# Functional Complexity of the Axonal Growth Cone: A Proteomic Analysis

**DOI:** 10.1371/journal.pone.0031858

**Published:** 2012-02-27

**Authors:** Adriana Estrada-Bernal, Staci D. Sanford, Lucas J. Sosa, Glenn C. Simon, Kirk C. Hansen, Karl H. Pfenninger

**Affiliations:** 1 Department of Pediatrics and Colorado Intellectual and Developmental Disabilities Research Center, University of Colorado School of Medicine, Aurora, Colorado, United States of America; 2 Department of Biochemistry and Molecular Genetics, University of Colorado School of Medicine, Aurora, Colorado, United States of America; Institut Jacque Monod, Centre National de la Recherche Scientifique, France

## Abstract

The growth cone, the tip of the emerging neurite, plays a crucial role in establishing the wiring of the developing nervous system. We performed an extensive proteomic analysis of axonal growth cones isolated from the brains of fetal Sprague-Dawley rats. Approximately 2000 proteins were identified at ≥99% confidence level. Using informatics, including functional annotation cluster and KEGG pathway analysis, we found great diversity of proteins involved in axonal pathfinding, cytoskeletal remodeling, vesicular traffic and carbohydrate metabolism, as expected. We also found a large and complex array of proteins involved in translation, protein folding, posttranslational processing, and proteasome/ubiquitination-dependent degradation. Immunofluorescence studies performed on hippocampal neurons in culture confirmed the presence in the axonal growth cone of proteins representative of these processes. These analyses also provide evidence for rough endoplasmic reticulum and reveal a reticular structure equipped with Golgi-like functions in the axonal growth cone. Furthermore, Western blot revealed the growth cone enrichment, relative to fetal brain homogenate, of some of the proteins involved in protein synthesis, folding and catabolism. Our study provides a resource for further research and amplifies the relatively recently developed concept that the axonal growth cone is equipped with proteins capable of performing a highly diverse range of functions.

## Introduction

The nerve growth cone is the enlarged leading edge of the growing neurite. It is the primary site of neurite formation, which involves plasmalemmal expansion as well as cytoskeletal assembly [1], [2], [3]. The growth cone advances through tissue by amoeboid movement while probing the microenvironment with its filopodia for molecular cues. *Axonal* growth cones, on which this report is focused, travel considerable distances through the central nervous system or peripheral tissues to reach their target cell(s) for synaptogenesis. Pathfinding is accomplished by detection of, and reaction to, multiple substrate-bound and soluble molecular signals, such as cell surface and extracellular matrix molecules, growth factors, growth cone attractants, and growth cone repellents [4], [5]. Once the axonal growth cone has reached and recognized an appropriate target cell synaptogenesis ensues. During this process the growth cone is replaced by a presynaptic nerve terminal. Thus, the nerve growth cone is a developmentally regulated structure specialized for neurite assembly, amoeboid movement, detection of growth and guidance signals, and target cell recognition for synaptogenesis. As such it plays a key role in neuronal network formation and modulation during development and plasticity. As our understanding of specific growth cone functions has increased so has the evidence for their complexity. Nevertheless, the axonal growth cone has been viewed traditionally as wholly dependent on the parent neuron's perikaryon for the supply of almost all macromolecular constituents.

Axonal growth cones can be isolated by subcellular fractionation from developing rodent brain with reasonable purity [6], [7]. Criteria for the identity and purity of the fraction include (a) electron microscopic analysis, (b) co-purification of growth cones micro-dissected from cultures, (c) the enrichment of “marker” molecules, such as growth-associated protein 43 (Gap43), known to be abundant in axonal growth cones, and (d) depletion of non-axonal proteins, such as dendritic and glial markers [6], [7], [8]. Thus, this “growth cone particle” (GCP) fraction can be used to determine the axonal growth cone's proteome. This was done successfully by Nozumi and co-workers [9], who validated the approach and described over 900 GCP proteins. They used the GCP preparation developed in our laboratory [7] and verified the presence of 131 GCP proteins in axonal growth cones of cultured cortical neurons by immunofluorescence microscopy. A major goal of their study was to identify potential new growth cone markers. Using a somewhat different approach and advanced instrumentation we identified over 2000 proteins at very high confidence level (≥99%) and subjected the identified species to extensive, broad-based informatics analysis. While our results are largely consistent with the data from the Nozumi et al. [9] study they reveal the presence in axonal growth cones of a highly complex machinery of biological processes. (Proteins are referred to by the official names of the genes encoding them. A list of all proteins and official gene names is provided in Table S1.)

## Results

### 1. Limitations and Validation of the Approach

GCPs are derived from whole forebrain and, thus, from a great variety of neuron types. Therefore, proteins identified in the GCP fraction may come from all or only from subsets of growth cones in the brain. However, the proteomic analysis favors the proteins shared by all growth cones because they are domineering. GCPs are recovered from a discontinuous density gradient as a band that also contains a large amount of cytosolic protein (essentially fetal brain high-speed supernatant [7]), and the GCPs cannot be washed by re-suspension and pelleting without disruption. To avoid GCP contamination with cytosolic proteins from other regions of the neuron or from other cells we plated GCPs on the physiologic substrate laminin, rinsed off soluble components and then harvested the GCP proteins. This procedure allowed us to remove the bulk of soluble proteins (and to isolate adhesive structures for separate analysis; Estrada et al., in preparation). To identify potentially contaminating cytosolic proteins we performed proteomic analysis of the fetal brain high-speed supernatant. In addition, we generated proteomic profiles from the laminin plating substrate (without GCPs), another potential contributor of non-GCP proteins. These two data sets represent “background” proteins.

Mass spectra were analyzed with Scaffold software, using the 99% confidence limit (minimum number peptides, 2; minimum confidence level for peptide identification, 95%). This identified a total of 2130 proteins in the plated GCP fraction (based on 139,158 spectra; Table S2). Spectral counts per protein reached from small numbers into the thousands. The number of identifying spectra per protein was used as a semi-quantitative measure of protein abundance (the limitations of this approach are considered in Discussion). Proteins whose spectral counts in the GCP preparation were equal or lower than in the background fractions were excluded from further analysis. This was done as follows: Spectral frequencies of proteins found in the laminin preparation were subtracted from those of the respective proteins in the GCP fraction. To determine how much high-speed supernatant protein to subtract as background we chose two test proteins, serum albumin (Alb) and α-fetoprotein (Afp), which are not growth cone components but present in fetal brain high-speed supernatant. We calculated the multipliers of their spectral numbers that equaled them to those of the GCP contamination levels so that, when subtracted, their spectral numbers in the GCP preparation would total zero. The two multipliers were 0.29 and 0.34 for Alb and Afp, respectively. We used the mean of these values (0.315) as a multiplier for all spectral counts in the high-speed supernatant and subtracted these numbers from the respective GCP spectral counts. Thus, the frequencies of identifying spectra of **net** GCP proteins were the result of subtracting the entire background [laminin fraction without GCPs+0.315×(high-speed supernatant fraction)]. This procedure almost completely eliminated from the GCP proteome not only Afp and Alb, but also 3 other serum proteins (transferrin, α2-macroglobulin and IgG subunits). This result validated the approach, at least within the limitation that contaminating proteins would be present in similar proportions. The background subtraction reduced the net GCP count by 313 to 1981 proteins.

Uploading the data into the DAVID informatics tool (DAVID Bioinformatics Resources 6.7; [10], [11], [12]) resulted in 1817 DAVID identifications (164 gi accession numbers, or <10%, were not recognized by DAVID). These 1817 GCP proteins formed the basis for all subsequent informatics analyses, together with their net (after background subtraction) spectral frequencies. These data are listed in Table S2. Protein names and accession numbers are listed as provided by Scaffold software. In a few cases, accession numbers refer to mouse or human, rather than rat, proteins. It should be noted that the spectral abundance of some proteins, especially those associated with adhesions (e.g., integrins; myristoylated alanine-rich C-kinase substrate), was much lower than expected. This likely is the result of incomplete solubilization of the laminin-adherent adhesion complexes.

**Figure 1:**
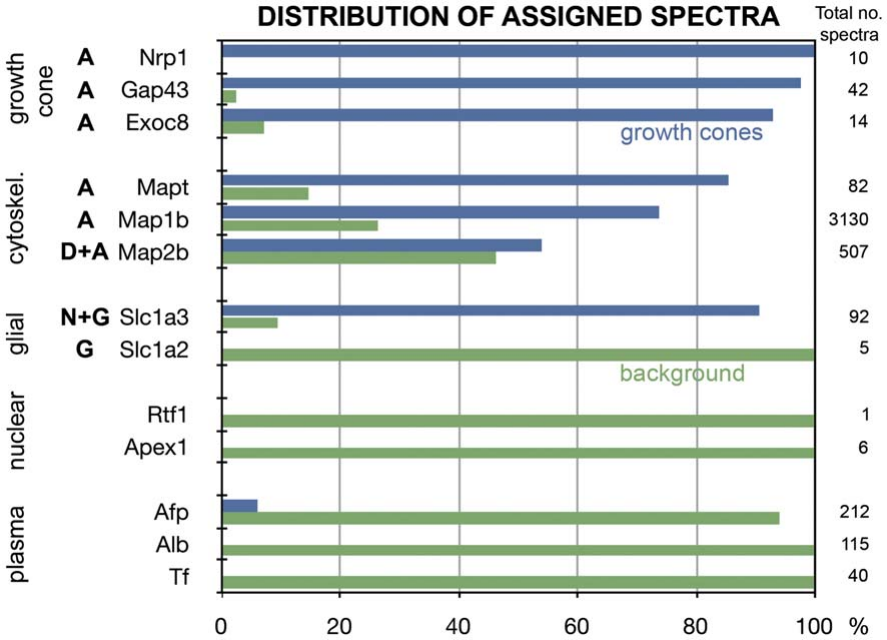
Relative distribution of assigned spectra between growth cones (blue) and background (green). Identified proteins (listed by gene name) are grouped as “markers” of axonal (A) growth cones, axonal or dendritic (D) cytoskeleton, neurons (N), glia (G), the nucleus or serum. Total numbers of identifying spectra detected for each protein are indicated on the right.

Figure 1 shows the distribution of selected assigned spectra between net GCPs (blue) and background (green) as % of total spectra detected in the crude GCP fraction, prior to background subtraction. The total number of spectra detected is shown on the right. Axonal growth cone markers, such as neuropilin 1 (Nrp1), Gap43 and exocyst component 8 (Exoc8) are essentially confined to the growth cone preparation. The axonal cytoskeletal components, microtubule-associated proteins tau (Mapt) and 1b (Map1b), also were detected primarily in the growth cone preparation, whereas Map2b, present in emerging (but not mature) axons and in dendrites [13], [14], [15], was about equally abundant in growth cones and background. The solute carrier family 1 (high-affinity glutamate transporter), member 3 (Slc1a3; also known as GLAST-1), present in both neurons and glia, was detected primarily in GCPs whereas member 2 (Slc1a2; also known as GLT-1), present in glia only, was confined to background [16]. Potential nuclear contaminants, an RNA polymerase II complex component (Rtf1) and APEX nuclease (Apex1), also were detected in the background only, as were the serum proteins Afp, Alb and transferrin (Tf), as mentioned above. Likewise, histones H2B (Hist1h2bl) and H4 were present in background but not in the net GCP preparation (Table S2). Except for Hist3h2ba no histones were seen in net GCPs, and nuclear lamins were not detected. These results strongly indicate that the growth cone preparation analyzed is primarily axonal and not significantly contaminated with extraneous proteins.

### 2. Overall Evaluation of the Proteome

**Figure 2:**
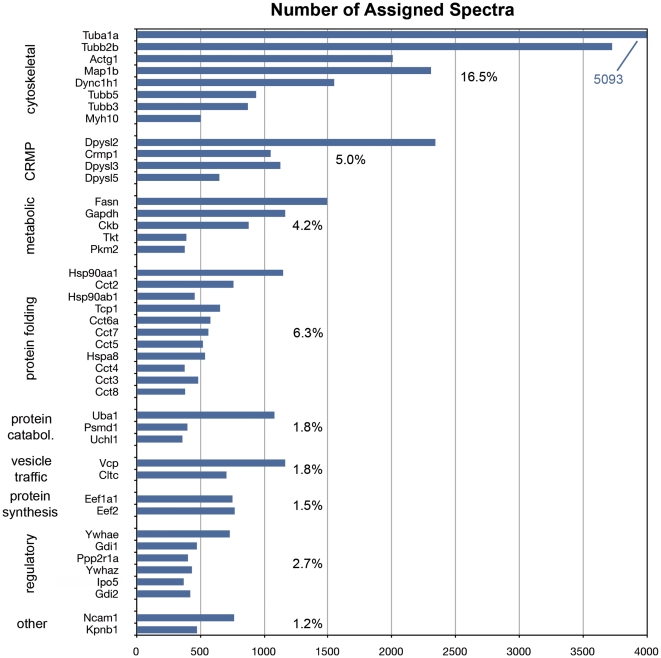
The 43 highest-ranked proteins based on identifying spectra (≥400) detected in the net GCP preparation. The proteins are grouped functionally. CRMP, collapsing response mediator proteins. Percent values indicate the cumulative number of spectra identifying the proteins shown in each group relative to the total number of spectra detected in growth cones.

To obtain an initial reading of the growth cone proteome we surveyed (a) the most abundant spectra and (b) the enrichment of functional annotations of cellular components in the net GCP preparation. All proteins with spectral frequencies of ≥400, the 43 most abundant proteins, are shown in Figure 2. They are presented in 8 functional groups. The % values indicate for each group the cumulative frequency of spectra relative to all spectra detected. As expected, cytoskeletal components and associated proteins were predominant and the 8 proteins in the group contributed 16.5% of all spectra. Among them was the microtubule motor dynein (Dync1h1) as the 6^th^ most abundant protein detected. This is consistent with the known importance of vesicular transport to the axonal growth cone. Collapsin response mediator proteins (CRMPs/dihydropyrimidinase-like proteins), involved in the regulation of microtubule function, cell polarity and growth cone repulsion [17], also form a highly prominent group of proteins. Metabolic proteins were abundant, as expected. Surprisingly, however, one of these was fatty acid synthase (Fasn). The vesicle-traffic-associated proteins valosin-containing protein (Vcp) and clathrin (Cltc, heavy chain) were heavily represented as well. Surprising, however, was the fact that 11 (or 25%) of the most abundant proteins were involved in protein folding, totaling 6.3% of all spectra detected. Also remarkable was the high frequency of spectra identifying 3 proteins involved in ubiquitin/proteasome-dependent protein catabolism and 2 translation elongation factors. Prominent proteins involved in cell regulatory functions included two 14-3-3 proteins (Ywhaz and Ywhae) and two Rab GDP dissociation inhibitors (Gdi1 and Gdi2), as well as two nuclear importins (Ipo5 and Kpnb1). Spectra of the Ig superfamily cell adhesion molecule Ncam1 also were detected with high frequency.

**Figure 3:**
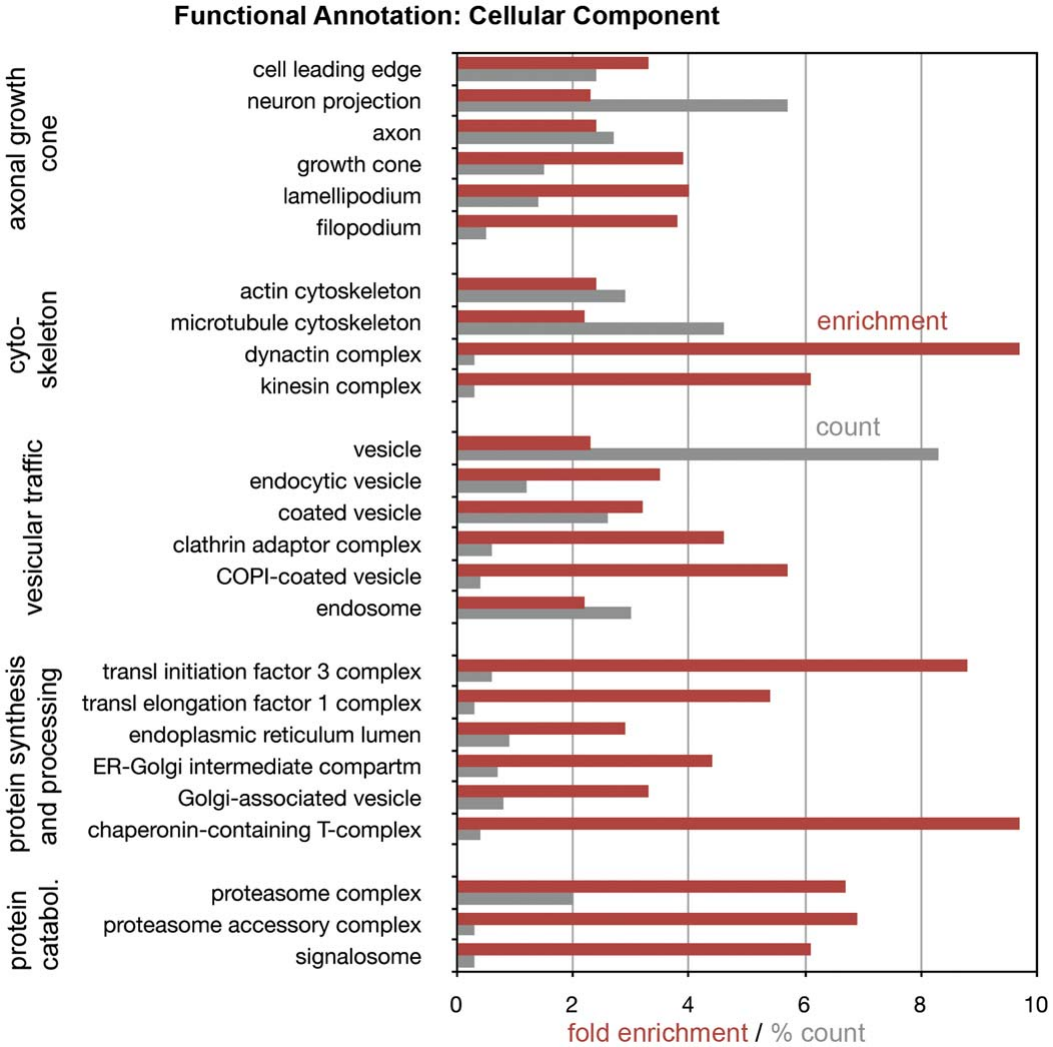
Functional annotation (“Cellular Component”) analysis of the growth cone proteome. GO Terms are listed in functional groups. Enrichment values (relative to random expression) are shown in red; % count (grey) indicates the number of protein species associated with each GO Term relative to the total number of protein species in net GCPs. For the enrichment values, p<0.01; Benjamini scores <0.05.

A complementary approach to the overall evaluation of the growth cone proteome is functional annotation using GO (Gene Ontology) Terms and the DAVID informatics tool. GO Terms fall into different categories, including “Cellular Component”. This category is particularly useful for describing functions of cellular organelles. Figure 3 shows examples of the most enriched cellular component GO Terms in the net growth cone proteome, grouped functionally. “Fold enrichment” refers not to relative protein abundance but to the number of relevant protein species present relative to random protein expression. “% count” indicates the percentage of protein species associated with each GO Term relative to the total number of protein species in the net GCP proteome. The results indicate the expected significant enrichment of GO Terms known to be associated with the axonal growth cone, such as “cytoskeleton” and “vesicular traffic”. Surprising, however, was the high *enrichment* of GO Term components associated with protein synthesis, processing (including folding) and degradation.

The relatively simple analyses just presented indicate the expected enrichment of growth cone markers (versus contaminants) and of other proteins associated with well-known growth cone functions in the GCP proteome. In addition, however, these analyses point to the prominent presence of highly developed machinery for protein synthesis, processing and degradation. These observations are explored in further detail below.

### 3. Functional Annotation Clusters (Biological Processes) and KEGG Pathways

**Figure 4:**
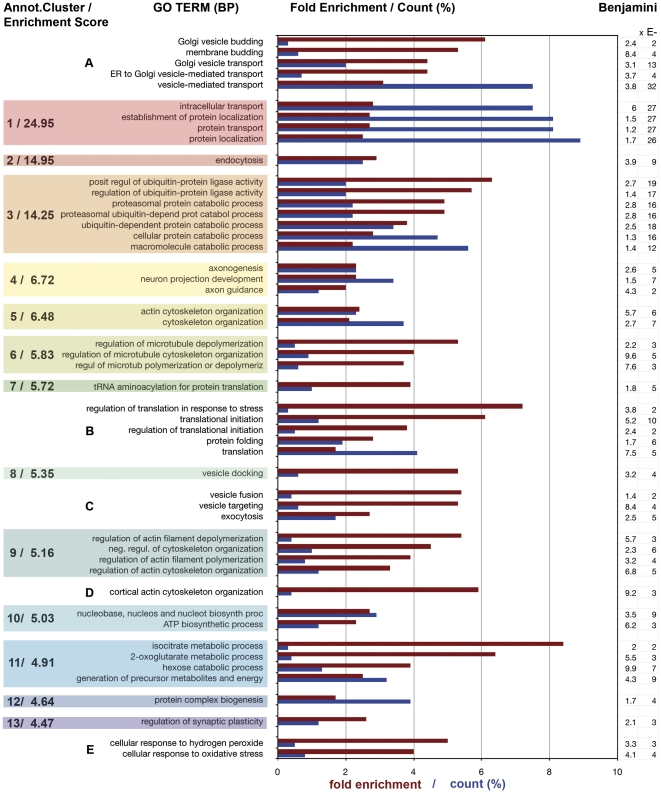
Functional annotation clusters (“Biological Process”, BP) in the growth cone proteome (DAVID). The 13 most enriched clusters and characteristic examples of their GO Terms are shown, together with their enrichment scores, in the colored boxes. Bars show GO Term enrichment (burgundy) and % count (number of included protein species relative to total number of protein species in GCPs; blue). Benjamini scores are shown on the far right. The DAVID annotation cluster analysis left out a number of highly enriched GO Terms that were grouped functionally (A–E) and are listed also.

GO Terms for biological processes and KEGG Pathways (Kyoto Encyclopedia of Genes and Genomes) describe ordered multi-step, multi-molecular operations in the cell. Similarly, annotation clusters, as provided by DAVID, describe functionally linked groups of biological-process GO Terms. Figure 4 illustrates the 13 most enriched annotation clusters (in terms of the number of protein species represented, relative to random expression of all genes in the genome). They are numbered 1–13, and highlighted by colored boxes. The enrichment score is indicated for each cluster; the fold enrichment (burgundy red) and % count (number of included protein species relative to total number of protein species present; blue) are shown for sample GO Terms in the bar graphs. Benjamini coefficients are shown for each GO Term on the far right. At <0.05 all values are significant, with most of them highly significant at <0.001. Some of the GO Terms that were strongly represented in the growth cone proteome were not included in any DAVID annotation clusters. We grouped these functionally and listed them also (A–E). The bar graph conveys the enrichment and the number of protein species participating in a specific biological process for a broad range of growth cone functions. Particularly striking are the high enrichment (>3-fold) of several biological processes and the large number of protein species (>7% of total number) participating in intracellular/vesicle-mediated transport (Annot. Cluster 1).

Most of the high-scoring annotation clusters and Groups C and D shown in Figure 4 were expected. However, Group A includes greatly enriched GO Terms involving Golgi functions, Annotation Cluster 3 deals with ubiquitin- and proteasome-dependent protein catabolism, Annotation Cluster 7 and Group B show high enrichment of components needed for protein synthesis (we detected 35 ribosomal proteins), and Annotation Cluster 12 is focused on protein complex assembly (almost all with highly significant Benjamini scores <0.001). Also of interest is the great enrichment of cellular responses to oxidative stress (Group E). Table 1 summarizes the data from the opposite end of the annotation cluster spectrum, clusters with very low “enrichment” scores and with GO Terms that are “de-enriched”. These clusters include, among obviously irrelevant GO Terms, such terms as “Regulation of transcription”, “Chromatin (dis)assembly”, and “Regulation of cell proliferation”. In other words, there is no evidence for the representation of these biological processes in the growth cone proteome.

**Table 1:** Excluded Annotation Clusters* and Sample GO Terms (BP FAT).

Cluster #	Cluster Subject	Enrichment Score	Prot. Count	Fold Change
**286**	Reproduction	0	-	-
*Sample GO Term*	*Gamete generation*	-	20	0.62
**285** **	Transcription	0	-	-
*Sample GO Term*	*Regulation of transcription*	-	72	0.45
**279**	Urogenital system	0.01	-	-
*Sample GO Term*	*Urogenital system development*	-	10	0.65
**268** **	Nucleus	0.02	-	-
*Sample GO Term*	*Chromatin (dis)assembly*	-	7	0.72
**264**	Limb development	0.02	-	-
*Sample GO Term*	*Limb morphogenesis*	-	6	0.64
**263** **	Cell proliferation	0.02	-	-
*Sample GO Term*	*Regulation of cell proliferation*	-	53	0.8
**261**	Immune response	0.03	-	-
*Sample GO Term*	*Regulation of leukocyte activation*	-	11	0.69
**256**	Immune/inflammatory response	0.05	-	-
*Sample GO Term*	*Inflammatory response*	-	13	0.6
**251**	Meiosis	0.06	-	-
*Sample GO Term*	*Meiosis*	-	5	0.8
**249**	Sensory perception	0.07	-	-
*Sample GO Term*	*Sensory perception*	-	18	0.12

*) Ten representative examples of the 50 least enriched (actually de-enriched) annotation clusters in the growth cone proteome (total number of annotation clusters identified, 290).

**) Involve key nuclear processes.

A complementary analysis of biological processes was performed with a search for KEGG pathways that are significantly enriched in the growth cone proteome. Table 2 lists the 20 pathways represented with the highest statistical significance. Four of these pathways (superscripted a-d) are represented by many components but lack key identifying elements: the Fcγ receptor (a); claudins and occludins (b); the insulin receptor (c); and ErbB (d). Therefore, they should be considered only to the extent that they overlap with other pathways operating in the growth cone (signaling that targets the cytoskeleton for a; cell junction/cell adhesion-related processes for b; growth factor signaling for c and d). The identification of the DNA replication pathway results mainly from the presence of mini-chromosome maintenance (Mcm) proteins 2–7 (components of the prereplication complex) and proliferating cell nuclear antigen (Pcna). This finding is surprising, but these proteins have been observed in the extranuclear cytoplasm after DNA replication [18], [19], [20] (for immunofluorescence data, see below). Among the other KEGG pathways, Proteasome, Aminoacyl-tRNA Biosynthesis, Ubiquitin-Mediated Proteolysis, and Terpenoid Backbone Biosynthesis are of particular interest because they confirm the significant enrichment of pathways involved in protein synthesis and degradation, as well as in the synthesis of cholesterol and the geranyl/farnesyl modifications of membrane proteins.

**Table 2:** KEGG Pathways in Growth Cones.

Term	Protein Count	%	Fold Enrichm.	P-value	Benjamini
Proteasome	31	1.7	5.0	1.6E-15	2.6E-13
Endocytosis	52	2.9	2.1	2.5E-7	2.1E-5
Aminoacyl-tRNA biosynthesis	19	1.0	3.8	3.2E-7	1.8E-5
Axon guidance	36	2.0	2.3	2.7E-6	1.1E-4
Ubiquitin mediated proteolysis	35	1.9	2.2	6.3E-6	2.1E-4
Regulation of actin cytoskeleton	46	2.5	1.8	1.2E-4	3.2E-3
Fc gamma R-mediated phagocytosis^a^	24	1.3	2.2	3.3E-4	7.8E-3
Tight junction^b^	31	1.7	1.9	4.7E-4	9.8E-3
Pyruvate metabolism	14	0.8	2.9	5.6E-4	1.0E-2
Citrate cycle (TCA cycle)	12	0.7	3.2	5.9E-4	9.8E-3
Terpenoid backbone biosynthesis	8	0.4	4.6	6.9E-4	1.0E-2
Long-term potentiation	19	1.0	2.3	1.0E-3	1.4E-2
Insulin signaling pathway^c^	30	1.7	1.8	1.3E-3	1.7E-2
Pentose phosphate pathway	10	0.6	3.3	1.6E-3	1.8E-2
Amino sugar and nucleotide sugar metabolism	14	0.8	2.6	1.6E-3	1.7E-2
Purine metabolism	33	1.8	1.7	2.1E-3	2.1E-2
Glutathione metabolism	15	0.8	2.4	2.4E-3	2.3E-2
DNA replication	12	0.7	2.8	2.5E-3	2.3E-2
Glycolysis/gluconeogenesis	21	1.2	2.0	2.7E-3	2.3E-2
ErbB signaling pathway^d^	21	1.2	2.0	3.1E-3	2.6E-2

(20 pathways identified with the highest statistical significance).

a–d : Pathways not included for further consideration because they share elements with several other pathways, and, especially, because their key identifying proteins [Fcγ receptor (a); claudins, occludins (b); insulin receptor (c); ErbB (d)] were not detected.

**Figure 5:**
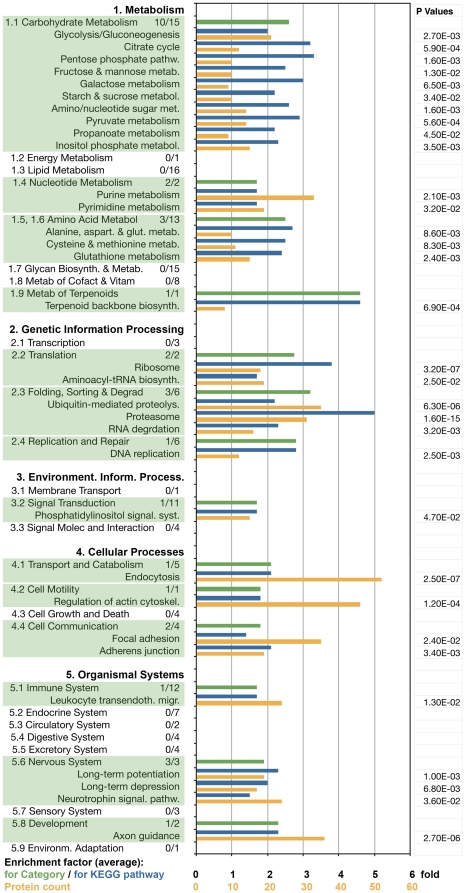
KEGG pathways in axonal growth cones. The five relevant KEGG pathway categories and their subcategories (enriched subcategories are shaded) are listed on the left. The numbers following each subcategory title indicate the number of pathways detected in GCPs/total number of identified pathways in mammals. Bars indicate the average enrichment factor for the subcategory (green), the enrichment factor for each pathway (blue), and the number of protein species of the pathway detected in GCPs (ochre). P values (<0.05) are shown on the right.

Figure 5 presents a more comprehensive view of known KEGG pathways that are significantly enriched in, or absent from, the growth cone proteome, listed by major category and subcategory (see KEGG Pathway Database). The numbers following the titles of each subcategory indicate the number of pathways detected in growth cones/total number of identified pathways in mammals. KEGG pathway subcategories that were detected in growth cones are highlighted by shading. The bar graph shows the average growth cone enrichment for each subcategory (green). Blue bars indicate enrichment of specific KEGG pathways, while ochre bars indicate the number of protein species of the pathway represented in growth cones. As in the previous analysis, there are both expected (typifying) and non-typifying results. Strong representation of carbohydrate (1.1), nucleotide (1.4) and amino acid (1.5, 1.6) metabolism, of several cellular processes (4.1, 4.2, 4.4), of nervous system-specific pathways (5.6) and axon guidance (5.8 Development) was expected (numbers refer to the KEGG pathway categories in Fig. 5). That none of the lipid metabolism pathways (1.3) were found to be enriched is surprising in view of the facts that extensive lipid re-modeling occurs at the growth cone [21], and that fatty acid synthase (Fasn) is one of the most abundant growth cone proteins (Fig. 2). One of the six defined Replication and Repair pathways was enriched (see also Table 2), but none of the Cell Growth and Death pathways (4.3) were found to be prominently represented.

Of special interest is the finding that two of the three enriched amino acid metabolism pathways (1.5, 1.6) involve cysteine, methionine and glutathione. This parallels the previous observation (Fig. 4, E) that the biological process GO Terms “Cellular response to hydrogen peroxide” and “Cellular response to oxidative stress” are highly enriched in the growth cone. The remarkable enrichment of the Terpenoid Backbone Biosynthesis pathway (1.9), the two Translation pathways (2.2) and, especially, 3 of the 6 Folding, Sorting and Degradation pathways (2.3) point again to the significance of protein synthesis, processing and degradation in the growth cone.

### 4. Protein Abundance by GO Term

**Figure 6:**
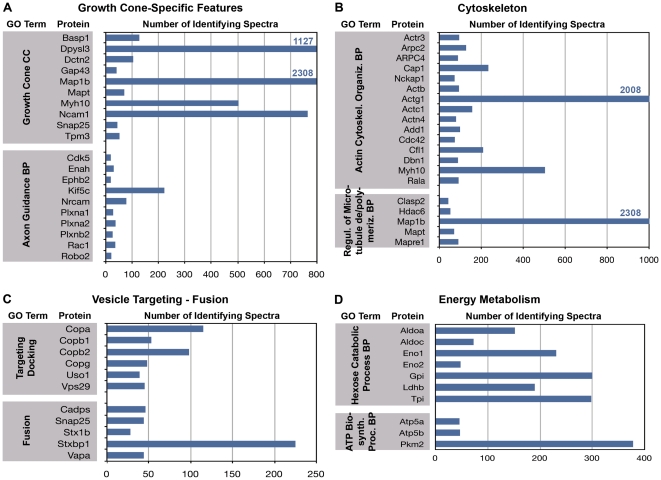
Proteins with the most abundant spectral counts under the following GO Terms: (A) “Growth Cone” (cytoplasmic component, CC) and “Axon Guidance” (biological process, BP); (B) “Actin Cytoskeletal Organization” (BP) and “Regulation of Microtubule Polymerization/Depolymerization” (BP); (C) “Vesicle Targeting/Docking” (BP) and “Vesicle Fusion” (BP); and (D) “Hexose Catabolic Process” (BP) and “ATP Biosynthetic Process” (BP). Note that scales are different for each panel.

**Figure 7:**
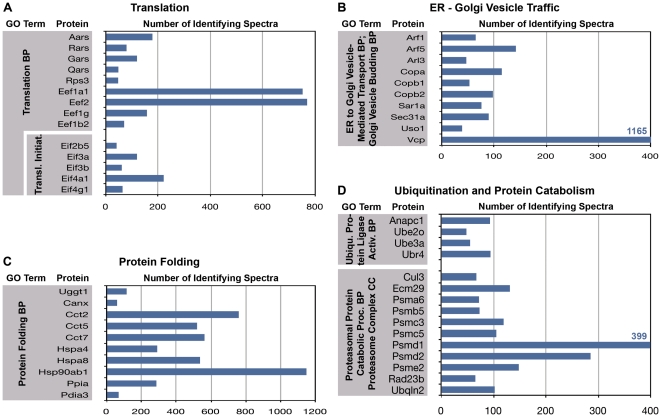
Frequencies of the most abundant spectra identifying proteins under the following GO Terms: (A) “Translation” and “Translational Initiation” (biological process, BP); (B) “ER to Golgi Vesicle-Mediated Transport” (BP)/“Golgi Vesicle Budding” (BP); (C) “Protein Folding” (BP); and (D) “Ubiquitin Protein Ligase Activity” (BP); “Proteasomal Protein Catabolic Process (BP)/“Proteasome Complex” (cellular component, CC).

**Figure 8:**
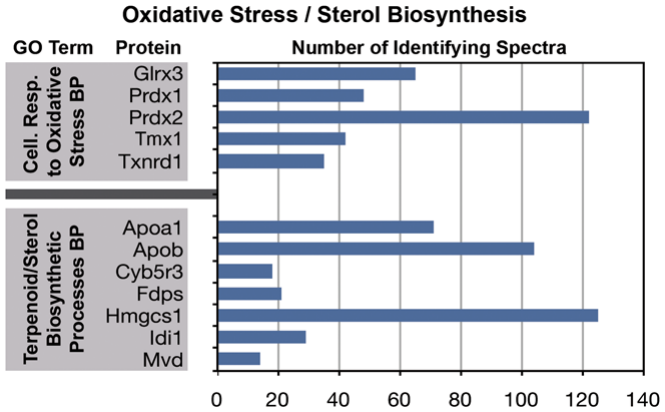
Frequencies of the most abundant spectra identifying proteins under the following GO Terms: “Cellular Response to Oxidative Stress” (BP) and “Terpenoid/Sterol Biosynthetic Processes” (BP).

The analyses presented above are focused on the diversity of protein species present in the growth cone. Here we address the question of the abundance of specific proteins in the functional clusters, as reflected semi-quantitatively in the relative number of identifying spectra. Figures 6, 7 and 8 relate the number of detected spectra to specific GO Terms, i.e., those that are typically prominent in the neuronal perikaryon (Figs. 7, 8) and, for comparison, those characteristic of growth cones (Fig. 6).

Figure 6A illustrates the most abundant protein-identifying spectra under the GO Terms “Growth cone“ (cytoplasmic component) and “Axon Guidance” (biological process). Spectral numbers for the “Growth Cone” proteins reach into the hundreds and thousands. Those involved in “Axon Guidance” were detected much less frequently, as would be expected, because many of these proteins are receptors or regulatory molecules that are present only on growth cone subsets and/or are generally not as abundant as, e.g., structural proteins. Figures 6B–D show the proteins with the highest spectral counts that are involved in cytoskeletal dynamics, vesicle targeting and fusion, and energy metabolism. Throughout, the proteins listed were detected with high frequency. The spectral frequencies shown in Figure 6 provide points of reference for the data shown in Figures 7 and 8. Figure 7 illustrates spectral frequencies for GO Terms related to translation (A), ER-to-Golgi vesicle traffic (B), protein folding (C), and ubiquitination and protein catabolism (D). Particularly striking is the abundance of certain elongation factors, valosin-containing protein (Vcp, a transitional endoplasmic reticulum ATPase), several species involved in protein folding, and proteasomal proteins.

Two other, somewhat unexpected biological-process GO Terms were enriched and robustly represented in axonal growth cones by multiple proteins identified with large numbers of spectra. These were “Cellular Response to Oxidative Stress” and “Terpenoid and Sterol Biosynthetic Process” (Fig. 8). These observations are consistent with the annotation cluster and KEGG pathway analyses. The robust number of identifying spectra indicates that these proteins are not contaminants but prominent elements of growth cone function.

Overall, linking the frequency of identifying spectra to annotation analysis signals the robust presence in axonal growth cones of the complete machinery of protein synthesis, folding, post-translational processing and proteasomal degradation. Furthermore these growth cones are enriched in proteins involved in the response to oxidative stress.

### 5. Western Blot and Immunolocalization of Selected Proteins

**Figure 9:**
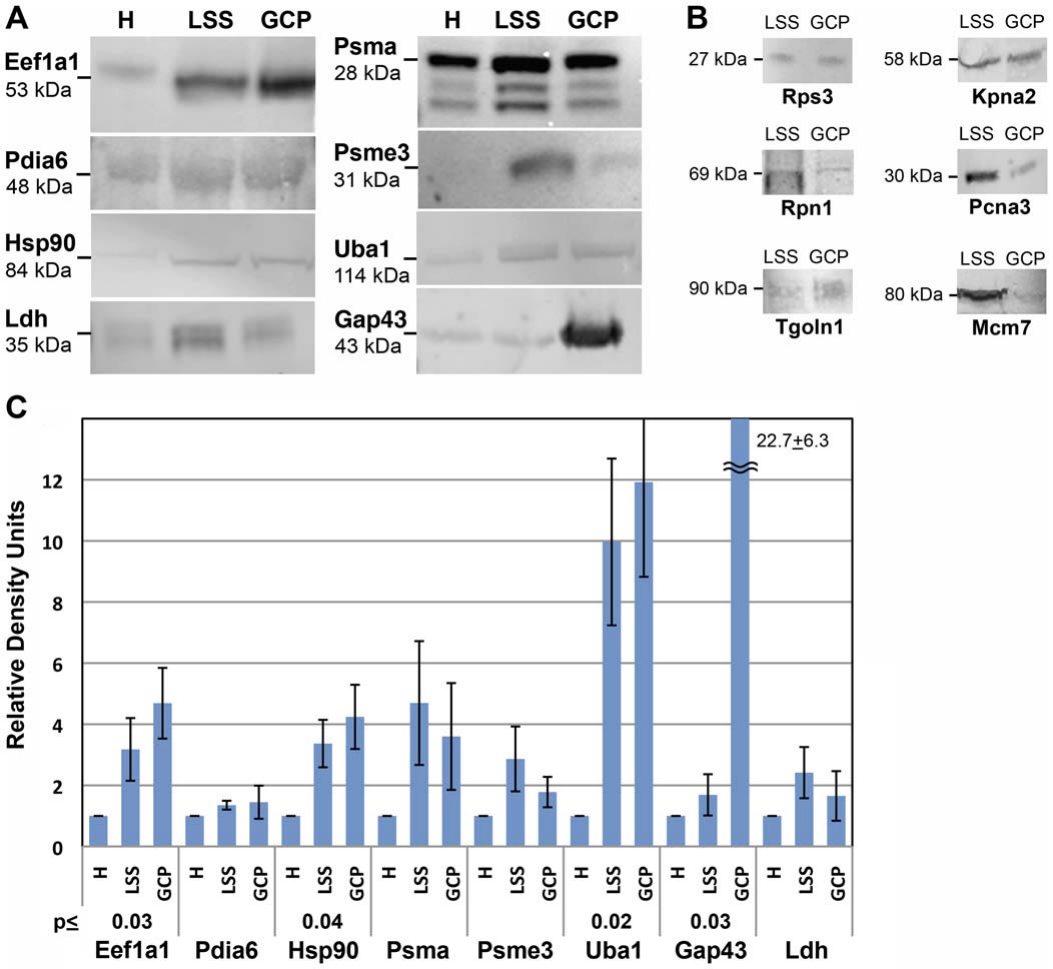
Abundance of non-typifying proteins in fetal brain fractions including GCPs, as determined by Western blot. **A** shows the relative abundance in fetal brain homogenate (H), low-speed supernatant (LSS) and GCPs (40 µg protein/lane) of six non-typifying proteins, a cytosolic marker (Ldh) and a growth cone marker (Gap43). Hsp90 is Hsp90ab1. **B** illustrates the presence of immunoreactivity of six additional proteins in fetal brain LSS and GCPs (equal amounts of protein loaded). **C** shows the quantitative analysis of the data in A. Net fluorescence intensities of the bands shown in A were normalized to H for each experiment and then averaged (experiments done in triplicate). The resulting relative density units shown equal the fold increase in immunoreactivity relative to H (means ± s.e.m.). p values are shown where GCP enrichment over H was significant.

To support the proteomic results with independent evidence we performed Western blot and immunolocalization analyses of proteins representative of the non-typifying functional categories of the growth cone. Western blots were carried out with fetal brain homogenate, its low-speed supernatant (LSS) and GCPs (derived from the LSS). The Western blots allow for semi-quantitative comparison of protein abundance across the fractions. To monitor gel loading and growth cone enrichment we probed blots of the three fractions (40 µg protein/lane; experiments performed in triplicate) for the cytosolic marker lactate dehydrogenase (Ldh) and the growth cone marker Gap43. Without exception Ldh seemed unevenly distributed, peaking in LSS (Figs. 9A and C), a result that was also obtained with antibody to another cytosolic metabolic enzyme, glyceraldehydes-3-phosphate dehydrogenase (Gapdh; data not shown). Even though the apparent differences were not significant statistically for n = 3, our observations indicated that these enzymes were not optimal as loading controls for this analysis. Gap43 was highly enriched in GCPs relative to homogenate in all experiments regardless of whether the numbers were based on protein loaded (constant) or the level of Ldh immunoreactivity (enrichment×22.7±6.3 versus 20.6±4.4, respectively; averages ± s.e.m; n = 3; Fig. 9A, C). Therefore, we show the data in Figure 9C with each antigen normalized to H levels and not to Ldh immunoreactivity (though results were similar). Our results indicated that protein disulfide isomerase (Pdia6), proteasome α subunits (Psma), and proteasome activator complex, subunit 3 (Psme3) were present but not enriched in GCPs relative to brain homogenate. However, some proteins involved in protein translation (eukaryotic elongation factor Eef1a1), folding (heat shock protein Hsp90ab1) and ubiquitin-dependent degradation (ubiquitin-like modifier-activating enzyme 1, Uba1), were significantly enriched in GCPs (Fig. 9A, C).

**Figure 10:**
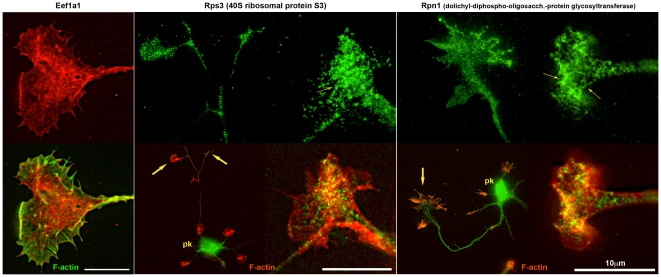
Hippocampal pyramidal neurons and/or their axonal growth cones labeled with antibodies to proteins involved in protein synthesis (top row). The antibody specificities are indicated above. Dual-fluorescence images including labeling for filamentous actin are shown in the bottom row. pk, neuronal perikaryon; large arrows, axonal growth cones; small arrows, reticular structures.

**Figure 11:**
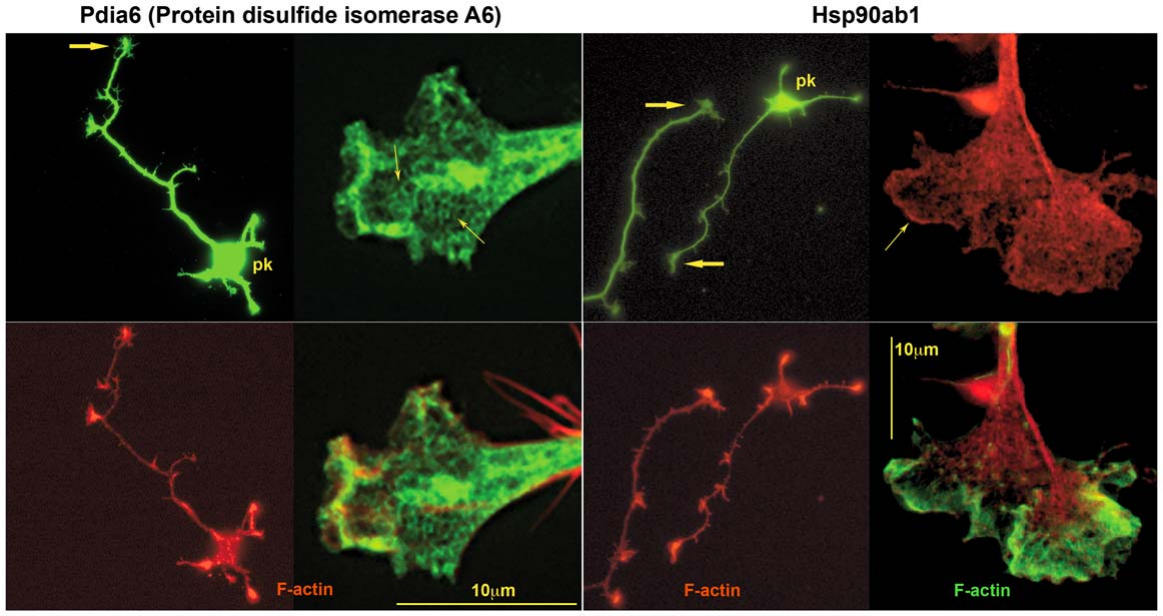
Hippocampal pyramidal neurons and their axonal growth cones labeled with antibodies to proteins involved in protein folding (top row). The antibody specificities are indicated above. Additional filamentous-actin label is shown in the bottom row. pk, neuronal perikaryon; large arrows, axonal growth cones. Small arrows indicate: left, reticular structures (Pdia6); right, edge labeling (Hsp90ab1).

**Figure 12:**
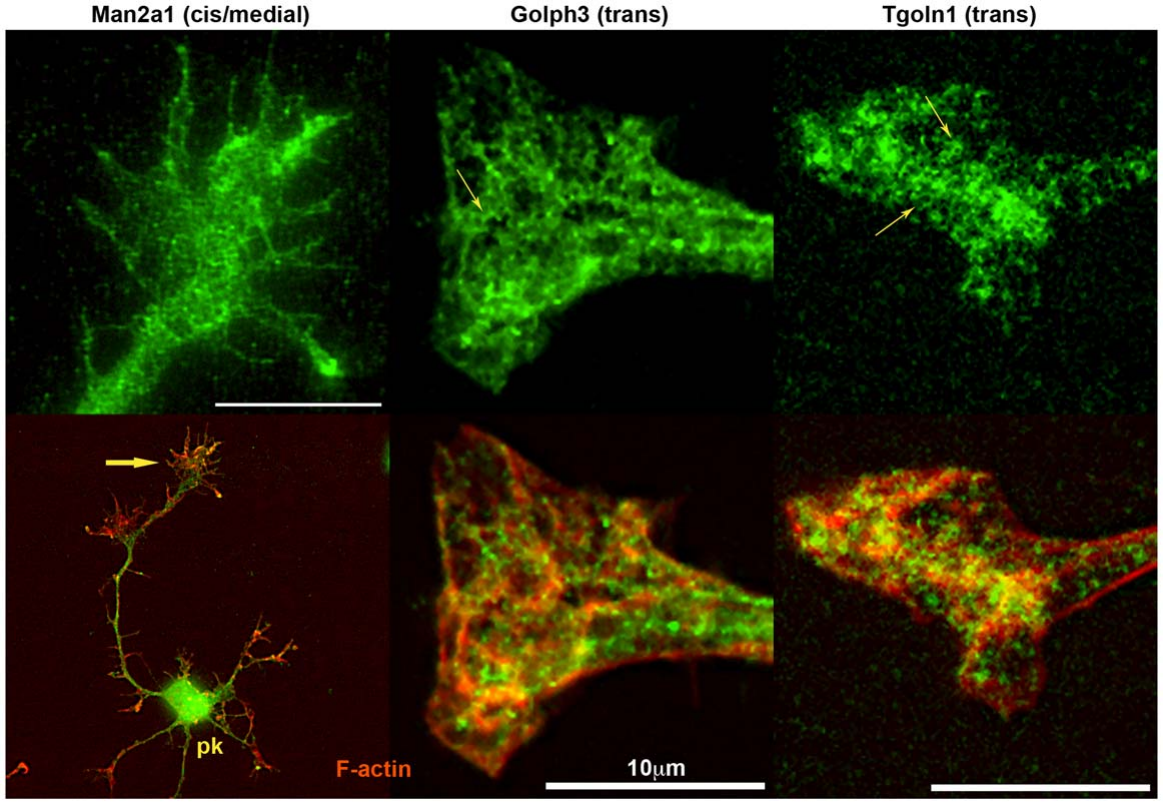
Hippocampal pyramidal neurons and/or their axonal growth cones labeled with antibodies to Golgi proteins (top row). The antibody specificities are indicated above. cis/medial/trans, known Golgi location of the antigens. pk, neuronal perikaryon; large arrow, axonal growth cone; small arrows, reticular structures.

**Figure 13:**
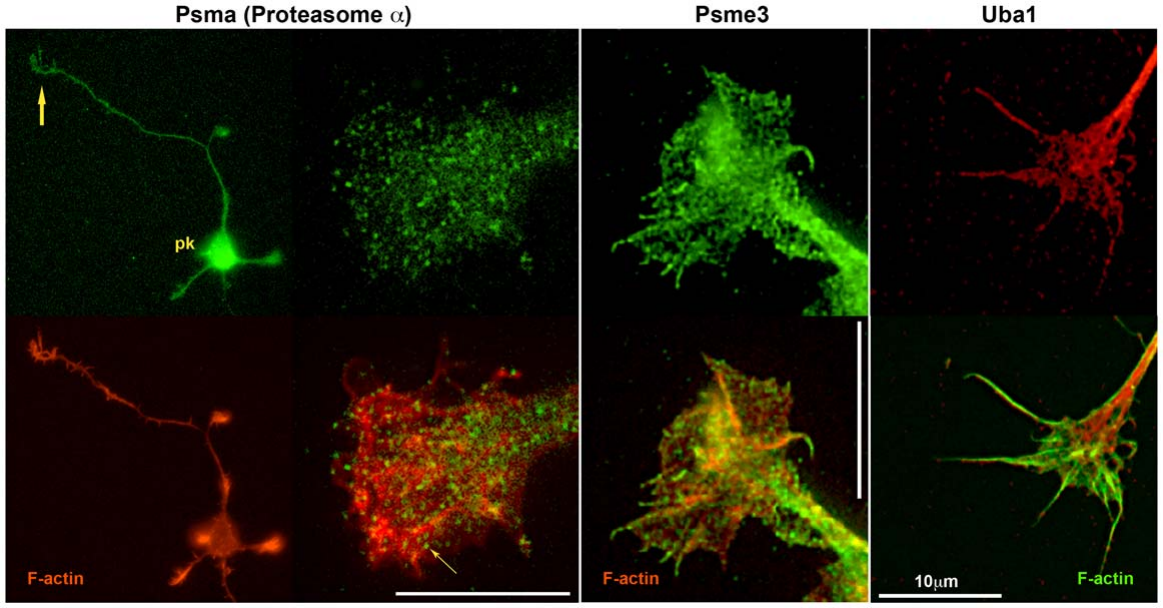
Hippocampal pyramidal neurons and/or their axonal growth cones labeled with antibodies to proteins involved in proteasomal degradation (top row). The antibody specificities are indicated above. Additional filamentous-actin label is shown in the bottom row. pk, neuronal perikaryon; large arrow, axonal growth cone; small arrow, Psma-positive, large puncta.

We localized sample proteins for each of the non-typifying functional categories by immunofluorescence in hippocampal pyramidal neurons sprouting in culture. These cultures, grown for 1–2 days, had been prepared from fetal brain at the same developmental age as the GCP fraction and, therefore, were comparable. Western blots to ascertain antibody specificity are shown in Figures 9A and B. Figure 10 illustrates the cellular location of elongation factor Eef1a1, of 40 S ribosomal protein S3 (Rps3), and of an endoplasmic-reticulum (ER) transmembrane protein, dolichyl-diphosphooligosaccharide protein glycosyltransferase subunit 1 (Rpn1; also known as ribophorin). Low-power images for Rps3 and Rpn1 ascertain the presence of immunoreactivity in *axonal* growth cones. The high-power images illustrate antigen distribution in greater detail (co-labeling with fluorescent phalloidin reveals filamentous actin). The three antigens of interest are non-homogeneously distributed. This is particularly evident for Rps3 and Rpn1, whose reactivity forms a reticulum of elongated, branching structures. Figure 11 illustrates presence and distribution of protein disulfide isomerase Pdia6 and heat shock protein Hsp90ab1 in axonal growth cones and throughout the hippocampal neuron. Pdia6, a rough-ER luminal component, occurs in the form of reticular structures, while the cytosolic Hsp90ab1 is more evenly distributed throughout the growth cone. Immunolocalization of the Golgi components α-mannosidase II (Man2a1), localized in cis/medial Golgi [22], [23], Golgi phosphoprotein 3 (Golph3, also known as GMx33), a cytosolic protein of the trans face of the Golgi matrix, and trans-Golgi network protein (Tgoln1; also known as TGN38), an integral membrane protein of the trans Golgi [24], [25], are illustrated in Figure 12. [The antibody to Man2a1 does not work in Western blots; specificity of the same anti-Golph3 (a single band of about 35 kDa) antibody was shown in [24]]. Immunoreactivity of the three Golgi components is evident in the axonal growth cones, as is the labeling of reticular patterns, especially for Golph3 and Tgoln1. However, organization of the label in Golgi-like stacks was not seen. Immunolocalization of proteins involved in protein catabolism (proteasome α subunits labeled with a pan-Psma antibody; Psme3 and Uba1) are shown in Figure 13. The low-power micrograph demonstrates presence of Psma in the axon and its growth cone, as well as in all other regions of the developing neuron. The labeling pattern observed at high magnification is punctate and, sometimes, forms larger clusters. Psme3 immunofluorescence is punctate as well and, together with Uba1, reaches into the filopodia.

**Figure 14:**
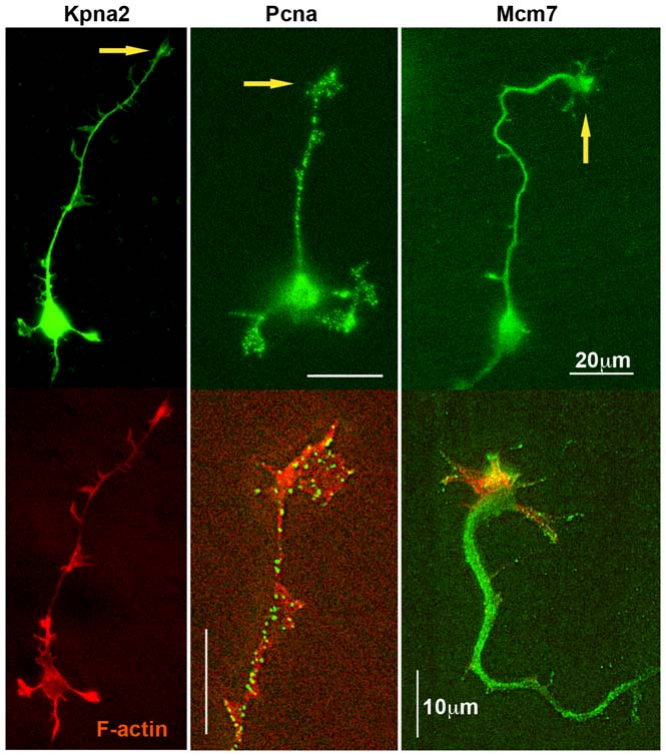
Hippocampal pyramidal neurons and their axonal growth cones labeled with antibodies to proteins involved in functions of the nucleus (top row). The antibody specificities are indicated above. Additional filamentous-actin label is shown in the bottom row. Large arrows, axonal growth cones.

The presence of “nuclear” proteins in the growth cone proteome deserves particular scrutiny. Figure 14 shows hippocampal pyramidal neurons and their axonal growth cones labeled with antibodies to an importin family protein, karyopherin α2 (Kpna2), to Pcna and to Mcm7, a DNA prereplication complex component. While the presence in the axonal growth cone of importins is consistent with recent reports [26], [27], [28], that of Mcm7 and Pcna is surprising but consistent with the proteomic data.

## Discussion

The axonal growth cone plays a critical role in the development of neuronal circuits so that its protein components and functions are of great interest. The goal of the present study was to generate extensive and robust data on the functional groups of proteins present in the axonal growth cone, at a time when the developing neuron is polarized and the growth cone widely separated from its perikaryon.

### 1. Data Validation

The starting material for the proteomic analysis was GCPs isolated from fetal rat brain, the preparation also used by Nozumi et al. [9]. Consistent with Nozumi's data, we found enrichment in the proteome of axonal growth cone “marker” proteins (such as Gap43) and abundance of proteins involved in well-established growth cone functions, such as vesicular traffic and cytoskeletal remodeling (see also [29]). We also observed a lack (after background subtraction) of proteins known to be absent from growth cones, such as serum proteins, glial markers and certain nuclear proteins [e.g., histones (H1, H2A, H2B, H3 and H4), Apex1 nuclease; but see below]. Several axonal guidance receptors (Ephb2, plexins, Robo2; see Fig. 6A), which are critical for growth cone function, also were evident in our analyses. Of the 17 candidate growth-associated proteins identified by Nozumi et al. [9], 14 were detected in our analysis as well. However, they detected only 12 of our 43 most abundant identifying spectra (≥400 spectra each). Among those not seen by them were Actg1 (actin, cytoplasmic 2 or γ-actin), Dpysl2 (dihydropyriminidase-related protein 2) and Tubb2b (tubulin beta-2B chain) (>2000 spectra each in our analysis). Some of the discrepancies may stem from differences in protein isoforms: We detected Tubb2b and Myh10 (myosin-10) whereas they identified Tubb2a (tubulin beta-2A chain) and Myh11 (myosin 11). Overall, however, there is substantial agreement except that our analysis revealed about twice the number of proteins reported earlier [9].

#### Nuclear proteins

It is evident that the nucleus is not a growth cone component. Indeed, we did not detect Apex1 and Rtf1 and identified only one of the core histones, Hist3h2ba, in our net preparation. Yet, two karyopherins/importins (Kpnb1 and Ipo5) were among the 43 most abundant proteins (≥400 spectra each in growth cones). The proteomic result was confirmed for Kpna2 by immunofluorescence (Fig. 14) and is consistent with reports showing their function in retrograde transport of signaling molecules from the axonal growth cone to the nucleus [26], [27]. More surprising was the detection of Mcm family DNA helicase subunits, Pcna and Hist3h2ba in the GCP preparation. However, Zivraj et al. [30] reported the presence of Mcm2, 6 and 7 transcripts in the mouse growth cone. Furthermore, immunofluorescence of intact neurons in culture (Fig. 14) clearly shows Pcna and Mcm7 in the axonal growth cone, a finding that may be related to the cytoplasmic occurrence of these proteins in other cells [18], [19]. We do not know whether these proteins, as well as Hist3h2ba, reached the growth cone without a functional role, whether they perform a function related to that in the nucleus (e.g., in conjunction with RNA), or whether they play an altogether different, unknown role as “moonlighting” proteins in the axonal growth cone [31].

Overall, evidence for the specificity of our data is strong, but each protein not previously shown to be in axonal growth cones should be immunolocalized before further conclusions can be drawn. The data in the Nozumi et al. [9] study suggest the same functional annotation groups we have identified, but the discrepancies show that sample amounts and preparation, as well as instrumentation, can substantially affect the outcome of a proteomic analysis.

### 2. Proteins Characteristic of Axonal Growth Cones

Our bioinformatics studies were performed and presented so as to minimize bias, using functional annotation clustering and KEGG pathway analyses as tools (DAVID). In addition, we present the numbers of identifying spectra as a semi-quantitative measure of protein abundance. Interpretation of these numbers requires caution because they are not dependent on protein abundance alone. Other factors influencing them are: protein solubility during sample preparation; protein size and cleavage efficiency; ionization efficiency of peptides. Furthermore, we used exclusion filters when sequencing peptides to optimize the number of unique peptides observed so that the most abundant proteins were undersampled. Nevertheless, the numbers of identifying spectra can provide a rough estimate of protein abundance.

As a motile structure distant from the perikaryon the axonal growth cone must have its own energy supply, and it is known to contain mitochondria. Not surprisingly, therefore, we detected 35 mitochondrial proteins, and the GO Terms “Hexose catabolic process” and “ATP biosynthetic process” are well represented by abundant spectra (Fig. 6; see also Figs. 2 and 4).

In view of the importance of vesicular transport to the growth cone, growth and dynamics of the microtubule cytoskeleton of the axon, and constant remodeling of the actin cytoskeleton during growth cone advance, we expected and found an abundance of proteins of the microtubule and actin cytoskeletons, as well as those involved in their regulation. Together with the collapsin response mediator proteins (CRMPs), which participate in growth cone steering and microtubule regulation [17], the most abundant species of these proteins comprise over 21% of all spectra detected (Fig. 2). Vesicle-based traffic (vesicle formation, endocytosis, transport, docking and fusion) is essential for plasmalemmal expansion and transfer of proteins from one cellular compartment to another. Proteins involved in traffic are highly prominent in every type of analysis conducted. For example, Vcp and Cltc are among the most frequent spectra detected (≫500). GO Terms describing vesicle traffic are highly enriched in all of the analyses (Figs. 3, 4, 6). The kinesin and dynactin complexes are enriched over six- and ten-fold, respectively, and vesicle-associated proteins amount to >8% of all proteins identified.

### 3. Non-Typifying Proteins in Axonal Growth Cones

The question of whether the axonal growth cone was capable of synthesizing proteins was a hotly debated issue not that long ago [32]. Yet, convincing evidence for protein synthesis in the axon and its growth cone has emerged over the past ten years [33], [34], [35], [36], [37], [38], [39], [40], [41], [42]. Some of the proteins synthesized in the axonal growth cone are known to be membrane proteins and may be glycosylated [35], [43]. However, our knowledge of posttranslational processing in the growth cone is limited [44]. Proteasomal protein degradation in the axonal growth cone has been reported and linked to axonal differentiation, pathfinding and regeneration [33], [45], [46], [47]. Our proteomic analysis confirms and extends these insights by demonstrating the presence of a very large number of proteins for protein synthesis, folding, processing and degradation. These issues are addressed below in greater detail.

#### Translation and Protein Folding

All of our analyses show strong representation, in terms of protein species diversity as well as numbers of identifying spectra detected, of the biological process of translation. The spectra identifying two elongation factors are among the 43 most abundant species detected in axonal growth cones (together accounting for 1.5% of spectra; Fig. 2). Functional annotation analysis for cellular components reveals 3- to nearly 10-fold enrichment of proteins involved in translational initiation, elongation, ER, Golgi-associated vesicle, and chaperonin-containing T-complex (Fig. 3). Annotation clusters (biological process) for tRNA aminoacylation and translation (cluster 7 and group B in Fig. 4) and KEGG Pathway analysis (Fig. 5; 2.2) show high enrichment of proteins involved in translation, including 35 ribosomal proteins (see also Fig. 7A). This was confirmed by immunolocalization in axonal growth cones in culture. The elongation factor Eef1a1 was concentrated primarily in the proximal growth cone, together with Rps3 and the rough-ER membrane protein Rpn1. Rps3 and Rpn1 are of particular interest because their labeling patterns are distinctly reticular, compatible with that of rough ER (Fig. 10). A similar pattern was observed with the ER lumen chaperone Pdia6 (Fig. 11), while the cytosolic Hsp90ab1 was diffusely distributed. Thus, immunofluorescence analysis confirms the proteomic data and identifies a rough ER-like reticular compartment in axonal growth cones.

Our analyses also reveal that 11 of the 43 most commonly detected spectra identify proteins involved in folding, such as heat shock proteins and chaperonins (Fig. 2). Together these 11 proteins represent 6.3% of all protein species identified (see also Fig. 7C). It is reasonable to assume that these chaperones and chaperonins are essential for folding and maintaining locally synthesized proteins (see also [38], [44]). Moreover, remodeling may serve to reactivate defunct proteins and to disassemble normal or abnormal protein aggregates [48], [49]. An increased need for protein remodeling may explain the abundance of the relevant proteins in the remote axonal growth cone.

#### Posttranslational Processing

At least one of the proteins known to be translated in the axonal growth cone, the kappa opioid receptor [43], has two potential N-glycosylation sites suggesting that a Golgi-like structure may be present in the axonal growth cone. Indeed, [44] presented evidence for rough ER- and Golgi-like compartments in the axon. Our functional annotation analysis (cellular component or biological process) showed high growth cone enrichment of proteins involved in ER-Golgi intermediate compartment, Golgi-associated vesicle (Fig. 3), Golgi vesicle budding, Golgi vesicle transport, and ER-Golgi vesicle-mediated transport (Fig. 4; Group A). Valosin-containing protein (Vcp), one of the most common axonal growth cone proteins (about 1200 spectra detected; Figs. 2 and 7B), seems to operate primarily in ER-to-Golgi vesicle traffic, but it may be involved in the regulation of protein homeostasis as well [50]. Immunolocalization of 3 Golgi “marker” proteins, Man2a1 (cis/medial Golgi), Golph3 (trans Golgi), and Tgoln1 (trans Golgi) showed that all three were readily detected in axonal growth cones. Man2a1 and, especially Golph3 and Tgoln1 were present in a distinctive reticular network. While there was no evidence for stacks of membrane sacs as seen in the classic Golgi apparatus, our data indicate the presence of a rather elaborate specialized compartment with Golgi-like functions in the axonal growth cone.

Farnesylation and geranylation are posttranslational protein modifications best known for GTP-binding proteins and depend on terpenoid and sterol biosynthetic processes. Interestingly, these processes are among the most highly enriched in the axonal growth cone (Table 2, Figs. 5 and 7). As farnesylation and geranylation seem to be permanent [51] this finding suggests that at least some of the proteins that carry such posttranslational modifications are synthesized *de novo* in the growth cone. This is consistent with the finding of mRNAs encoding small G proteins [30] and their translation products, and of Rab geranylgeranyl-transferases (present study) in the growth cone.

#### Growth Cone Proteome versus Transcriptome

The recently published transcriptome of axonal growth cones [30] (see also that of axons [52]) lists over 400 identified transcripts in mouse and Xenopus growth cones. Functional classification of messages suggests that the axon is equipped to synthesize a highly diverse range of proteins. Even though the systems used for the two studies are not identical (growth cones laser-capture microdissected from cultured retinal ganglion cells versus those isolated from fetal brain; different species) the comparison of mouse transcriptome and rat proteome is of interest. Overall, the functional categories of transcripts and proteins identified in the axonal growth cones overlap extensively. For a more detailed comparison of transcriptome and proteome we analyzed two protein families (Rab and Kif) and the subunits of a large protein complex, the proteasome (Table 3). For the 26 Rabs, 10 Kifs and 37 Psms detected in the proteome, Zivraj et al. [30] reported 4, 5 and 16 (or about 15%, 50% and 43%) corresponding messages, respectively. Thus, there is overlap, but the reported transcriptome of the axonal growth cone lacked the messages encoding a significant number of protein species, suggesting that these are synthesized in the perikaryon only. For Rabs, Kifs and Psms protein prevalence, as judged from the abundance of identifying spectra detected (Table 3), did not correlate with the presence or absence of corresponding transcripts (and presumably local translation) in the growth cone (see, e.g., Rab3b vs. Rab11b; Kif5c vs. Kif22; Psmd1 vs. Psmf1; Psmd12 vs. Psme4). Furthermore, both “structural” (Psma and Psmb; italicized in Table 3) and regulatory (Psmc – Psmf) subunits of the proteasome may or may not be represented in the transcriptome. Although we have only limited information about which messages are translated in the axon and its growth cone the comparison of proteome and transcriptome provides an important tool for hypothesis generation.

**Table 3:** Proteome of the Growth Cone versus Transcriptome of the Growing Axon.

PROTEIN ISOFORMS (Rab; Kif)			SUBUNITS OF A LARGE PROTEIN COMPLEX		
Rab	Proteome # spectra*	Transcriptome [30]	Psm**	Proteome # spectra*	Transcriptome [30]
**1a**	44	○	***Psma1***	57	X
**1b**	62	X	***Psma2***	57	○
**2a**	101	X	***Psma3(I)***	24	X
**2b**	17	○	***Psma4***	67	○
**3a**	56	○	***Psma5***	57	○
**3b**	5	○	***Psma6***	72	X
**3c**	58	○	***Psma7***	53	○
**4a**	9	○	***Psmb1***	70	○
**4b**	11	○	***Psmb2***	38	○
**5a**	52	○	***Psmb3***	47	X
**5b**	41	○	***Psmb4***	33	X
**5c**	113	○	***Psmb5***	73	○
**6a**	63	○	***Psmb6***	25	X
**6b**	30	X	***Psmb7***	50	X
**7a**	92	○	***Psmb8***	0	X
**8a**	5	○	**Psmc1**	84	○
**9a**	5	○	**Psmc2**	55	X
**9b**	8	○	**Psmc3**	119	○
**10**	83	○	**Psmc4**	103	X
**11b**	163	○	**Psmc5**	105	○
**14**	132	X	**Psmc6**	82	X
**21**	27	○	**Psmd1**	399	○
**23**	14	○	**Psmd2**	284	○
**31**	20	○	**Psmd3**	122	X
**33a**	11	○	**Psmd4**	102	○
**35**	30	○	**Psmd5**	47	○
*Entries*	*26*	*4*	**Psmd6**	94	X
**Kif**	**Proteome # spectra** *	**Transcriptome ** [**30**]	**Psmd7**	108	○
**1a**	76	X	**Psmd10**	6	○
**1b**	9	○	**Psmd11**	118	X
**2**	28	○	**Psmd12**	139	X
**3a**	20	○	**Psmd13**	150	○
**3b**	7	○	**Psmd14**	139	○
**5a**	6	○	**Pmse1**	21	○
**5b**	80	X	**Psme2**	148	○
**5c**	222	X	**Psme3**	42	○
**21a**	32	○	**Psme4**	28	X
**21b**	66	○	**Psmf1**	3	○
**22**	0	X	*-*	*-*	*-*
**c2**	0	X	*Entries*	*37*	*16*
*Entries*	*10*	*5*	**) Italicized symbols indicate structural proteasome subunits		

*) Net spectral numbers.

x) mRNA detected in mouse growth cones.

#### Protein Degradation

The most abundant assigned spectra in our analysis include 3 proteins involved in ubiquitination/proteasome function (Fig. 2). Functional annotation (Cellular Component) indicates ≥6-fold enrichment of proteasome and signalosome complexes (Fig. 3), and the third-most enriched annotation cluster (biological process) comprises functions associated with ubiquitin-dependent and/or proteasome-dependent protein catabolism (Fig. 4; see also Fig. 7D). KEGG analysis shows proteasome function as the most highly enriched pathway in the growth cone. Localization by immunofluorescence confirms these findings and shows a pattern of small puncta or larger clusters in the axonal growth cone for the structural proteasome α subunits. The patterns for the regulatory components, Psme3 and Uba1, are similar but, strikingly, reach into fine filopodia. In other words, our proteomic results augment the concept that axonal growth cones contain elaborate machinery for protein degradation [33], [45], [46], [47]. The need for local protein degradation is perhaps not so surprising once we consider the large amount of protein transported down the axon. However, ubiquitin- and proteasome-dependent protein catabolism has been implicated in highly selective degradation of proteins that affect the growth cone response during midline crossing and others involved in synaptogenesis and plasticity [33], [45], [46], [47], [53], [54]. Thus, such controlled and selective protein breakdown seems to play a major role in the regulation of many growth cone-specific functions.

### 4. Overall Conclusions

Our proteomic analysis of axonal growth cones isolated from fetal rat forebrain has identified >1800 proteins with a high level of confidence (≥99%). Many of these play roles in typical, well-understood growth cone functions and, thus, were expected. They include, first and foremost, actin and tubulin and regulators of the respective cytoskeletal structures, as well as proteins involved in vesicular traffic. Less abundant but prominent in their variety are axonal guidance receptors, adhesion molecules, and regulatory proteins, such as GTP-binding proteins. The data provided here should be a useful resource for further studies on growth cone functions, either new or well-established.

Perhaps of greater importance is the identification of a large number of proteins that define, by annotation and KEGG pathway analyses, machinery for protein synthesis, folding, posttranslational processing, complex assembly and regulated degradation in the axonal growth cone. These findings confirm and amplify previous studies that located these processes at the axonal growth cone [33], [38], [40], [41], [44]. However, (i) the preponderance of the relevant protein species, relative to random gene expression and based on the high numbers of identifying spectra detected, and (ii) the quantitative enrichment of a number of these proteins in the axonal growth cone was not expected. Furthermore, we describe for the first time (iii) discrete reticular structures with rough-ER and Golgi apparatus functions in the axonal growth cone. Especially striking are the amounts and variety of proteins involved in protein folding and catabolism. From an overall viewpoint, our study confirms and extends the concept that the axonal growth cone is much more broadly equipped with cellular functions than originally assumed and, thus, can function as a quasi-autonomous, distant outpost of the neuron.

## Materials and Methods

### Ethics Statement

Animal use: Pregnant Sprague-Dawley rats were purchased from Harlan Laboratories (Indianapolis, IN) and kept for a few days in the local animal facility accredited by the Association for Assessment and Accreditation of Laboratory Animal Care (Animal Welfare Assurance Number PHS A3269-01). The animals were handled in strict compliance with Protocol #21708(01)1B, approved by the University of Colorado Health Sciences Center Animal Care and Use Committee and with the Guidelines for the Care and Use of Laboratory Animals of the National Institutes of Health. For tissue collection rats were sacrificed under terminal isoflurane anesthesia, and every effort was made to minimize suffering.

### Materials

Antibody specificities, hosts and sources: Golph3 and Tgoln1 (rabbit polyclonal) were generous gifts of Dr. C. Wu, then at University of Colorado School of Medicine. Kpna2 (chicken polyclonal), Man2a1 (mouse monoclonal), Mcm7 (mouse monoclonal), Pcna (rabbit polyclonal), Pdia6 (chicken polyclonal), and Uba1 (rabbit polyclonal) from Abcam (Cambridge, MA). Rpn1 (rabbit polyclonal) from Abgent (San Diego, CA). Hsp90ab1 (rabbit polyclonal) from Affinity Bioreagents, Inc. (Golden, CO). Rps3 (rabbit polyclonal) from Cell Signaling Technologies, Inc. (Beverly, MA). Psme3 (rabbit polyclonal) from Enzo Life Sciences (Farmingdale, NY). Eef1a1 (mouse monoclonal) and Psma (anti-α 1, 2, 3, 5, 6, 7; mouse monoclonal) from EMD Millipore (Billerica, MA). Cell culture supplies were from Invitrogen/Life Technologies (Carlsbad, CA). Other chemicals, unless stated otherwise, were from Sigma-Aldrich (St. Louis, MO) or Thermo Fisher Scientific (Pittsburgh, PA) and of the highest quality available.

### Growth cone isolation

GCPs were prepared as described previously [6], [7]. Briefly, whole brains from gestation day-18 fetal rats were homogenized in 0.32 M sucrose containing 1 mM MgCl_2_, 2 mM TES buffer (pH 7.3), and 2 µM aprotinin. Low-speed (1660×g, 15 min) supernatant (LSS) of the homogenate was layered onto a discontinuous density gradient (0.83 and 2.66 M sucrose containing MgCl_2_ and TES buffer) and spun to equilibrium at 242,000×g at 4°C for 40 min in a vertical rotor (VTi50, Beckman). The GCP fraction at the 0.32/0.83 M-sucrose interface and a sample of the gradient's supernatant (0.32 M sucrose) were collected.

### Plating of GCPs in laminin-coated wells

24-well tissue culture plates were coated with 20 µg/ml of laminin in Hank's balanced salt solution for 3 h and rinsed three times with phosphate-buffered saline (PBS) supplemented with 0.9 mM CaCl_2_ and 0.5 mM MgCl_2_ (PBS-Ca^+2^/Mg^2+^). Wells were blocked with 5% non-fat dry milk in PBS for 30 minutes and rinsed again.

GCPs were gradually diluted with 2× modified Krebs buffer (0.05 M sucrose, 0.1 M NaCl, 5 mM KCl, 22 mM HEPES, 10 mM glucose, 1.2 mM NaH_2_PO_4_, 1.2 mM MgCl_2_, 2 mM CaCl_2_, pH 7.3) and incubated at 37°C for 10 min. After incubation equal aliquots of the GCP suspension were transferred into the 24-well culture dish, spun at 4,000 rpm for 15 min and incubated at 37°C for 30 min. Under these conditions the resealed and viable GCPs attached to the laminin-coated plastic.

Attached GCPs were extracted in two steps: (1) Brij Buffer (5 mM MgCl_2_, 1 mM CaCl_2_, 1% Brij 98 in PBS) was added for incubation at 37°C for 20 min, followed by 40 min at 4°C. The supernatant was collected as the Brij-soluble fraction. (2) Remaining adherent structures were rinsed 2 times with PBS-Ca^+2^/Mg^2+^ and lysed in 1% SDS. A chloroform/methanol (C/M) precipitation [55] was performed with all samples, and the pellets were resuspended in 5% SDS and used for electrophoresis.

Controls: (a) Laminin-coated wells without GCPs. These were extracted with 1% SDS and the supernatant C/M-precipitated. The pellets were solubilized in 5% SDS. (b) Aliquot of the gradient's supernatant (clear; containing soluble proteins of the brain homogenate). This sample was C/M precipitated and solubilized in 5% SDS.

### Gel electrophoresis and sample preparation for mass spectrometry

Protein samples (50–60 µg/lane), alongside with dual-colored Precision Plus Protein™ standards (Bio-Rad Laboratories, Inc., Hercules, CA), were resolved by SDS-polyacrylamide gel electrophoresis using NuPAGE Novex Bis-Tris gradient gels (Invitrogen, Carlsbad, CA). After separation, the gel was stained with Coomassie Blue (Invitrogen). Each lane of the gel was cut into 25 equal-sized pieces, which resulted in a range of 30–400 (average about 80) proteins per slice. Proteins in the gel were digested as follows. Bands were destained in 200 µl of 25 mM ammonium bicarbonate in 50% v/v acetonitrile (ACN) for 15 min and then incubated in 200 µl of 100% ACN for 15 min at room temperature. After addition of dithiothreitol (DTT) to a final concentration of 10 mM, slices were incubated at 65°C for 30 min to reduce the disulfide bonds. The reduced cysteines were alkylated with the addition of iodoacetamide (IAA) at a final concentration of 20 mM (30 min at room temperature in the dark). The IAA was removed, and washes were performed with 200 µl of distilled water followed by addition of 100 µl of ACN. ACN (i.e., all liquid) was removed in a vacuum concentrator to allow complete gel rehydration thereafter. We added 50 µl of 0.01 µg/µl trypsin in 25 mM ammonium bicarbonate (∼pH 7.4) to each sample and allowed the gel pieces to rehydrate at 4°C for 30 min, before incubation at 37°C overnight. The tryptic mixtures were acidified with formic acid (FA) up to a final concentration of 1%. Peptides were extracted three times from the gel slices using 50% ACN and 1% FA, concentrated by SpeedVac to a desired volume (∼18 µl), and subjected to LC-MS/MS analysis. If necessary, they were stored at −20°C.

### Liquid Chromatography–Tandem Mass Spectrometry

Nano-flow reverse phase LC-MS/MS was performed using a capillary HPLC system (Agilent 1200, Palo Alto, CA) coupled via an electrospray ionization source to a linear ion trap hybrid ion cyclotron resonance mass spectrometer (LTQ-FT Ultra, ThermoFisher; San Jose, CA).

Eight microliters of the tryptic peptides were pre-concentrated and desalted on a C_18_ trap column ZORBAX 300SB-C_18_, (5 µm i.d. ×5 mm, Agilent Technologies, Santa Clara, CA) with 5% ACN, 0.1% FA at a flow rate of 15 µl/min for 5 minutes. The separation of the tryptic peptides was performed at a flow rate of 380 nl/min on a C_18_ reverse phase column (75 µm ID×360 µm OD×100 mm length) packed in-house with 4-µm, 100-Å pore size C_18_ reversed-phase stationary phase (Synergy, Phenomenex, Torrance, CA), kept at a constant 40°C using an in-house built column heater. The mobile phases consisted of 5% ACN with 0.1% FA (A) and 95% ACN with 0.1% FA (B), respectively. A 90-min linear gradient from 5 to 50% B was typically used. Data acquisition was performed using the instrument-supplied Xcalibur (version 2.0.6) software. The LC runs were monitored in positive ion mode by sequentially recording survey MS scans (m/z 400–2000) in the ion cyclotron resonance cell, while three MS/MS were obtained in the ion trap via collision-induced dissociation for the most intense ions. Once ions were selected for MS/MS they were reacquired after 30 seconds and then excluded for 90 seconds.

### Database searching, protein identification

MS and MS/MS spectra were de-isotoped, centroided from raw data files and converted into “mgf” files using an in-house script. Mascot (version 2.2; Matrix Science Inc., London, UK) was used to perform database searches against rat sequences in the SwissProt database using the extracted data. Parent MS tolerance was set at ±10 ppm with MS/MS tolerance set at ±0.6 Da. Trypsin specificity was used allowing for 1 missed cleavage. The modifications of methionine oxidation, protein N-terminal acetylation, and peptide N-terminal pyroglutamic acid formation were allowed for, and cysteine carbamidomethylation was set as a fixed modification.

Scaffold version 2 (Proteome Software, Portland, OR, USA) was used to validate and compare MS/MS based peptide and protein identifications. All “.DAT” files (from Mascot) for the 25 gel bands were loaded together as one “biological sample” within Scaffold. Peptide identifications were accepted if they could be established at greater than 95.0% probability as specified by the Peptide Prophet algorithm. Protein identifications were accepted if they could be established at greater than 99.0% probability and contained at least two identified unique peptides. Proteins are referred to by their official encoding gene names (Entrez Gene; NCBI, U.S. National Library of Medicine). A list of abbreviations and protein names is provided in Table S1.

### Data analysis

Protein identifications and number of identifying spectra for each fraction were exported from Scaffold software. The following parameters were used: 99% confidence limit for protein identification; minimum number of peptides, 2; minimum confidence level for peptide identification, 95%. For the purposes of the present overall growth cone analysis, data for Brij-soluble and Brij-insoluble fractions were combined. “Background”, i.e., potentially contaminating polypeptides from the laminin plating matrix and the gradient supernatant, were subtracted as explained and illustrated in Results. The net growth cone (GCP) data were used for further analysis. The raw data are shown in Table S2.

For annotation analysis, gi protein accession numbers were uploaded into the DAVID (Database for Annotation, Visualization and Integrated Discovery) informatics tool (DAVID Bioinformatics Resources 6.7; [10], [11], [12]). For GO Term (Gene Ontology) analysis we studied the “Cellular Component” and “Biological Process” categories using the GO FAT default settings. At these settings the program uses a subset of GO Terms depleted of the broadest terms (primarily from the top 5 levels of the tree) to avoid overshadowing of the more specific terms (term specificity defined on the basis of the number of child terms in the hierarchy; see DAVID website). Use of GO FAT generated more informative results than that of any specific GO Term level. For functional annotation searches we set the following parameters: “Cellular Component”, threshold count 5, EASE 0.1 (resulting in 171 chart records); “Biological Process”, threshold count 5, EASE 0.1 (resulting in 481 chart records); for functional annotation *clusters* (“Biological Process”), medium stringency (resulting in 194 clusters). For KEGG pathway searches the parameters were: Threshold count 5 (resulting in 54 records).

Enrichment values (GO Terms), enrichment scores (annotation clusters), and statistical determinants (p values and Benjamini coefficients) are those calculated by DAVID software. The Benjamini coefficients are Benjamini-Hochberg corrected p values. They are adjusted for multiple comparisons to lower the family-wise false discovery rate and, thus, are more conservative than Fisher Exact p values.

### Western Blots

Fetal brain homogenate (H), LSS and pelleted GCPs (equal amounts of protein loaded) were analyzed by SDS gel electrophoresis as described above. Resolved proteins were transferred electrophoretically onto Immobilon P (EMD Millipore Co., Billerica, MA) membranes. Membranes quenched in Tris-buffered saline with 5% non-fat evaporated milk and 0.1% Tween-20 were probed with primary and fluorescent secondary antibodies (Alexa Fluor 647, 555 or 488; Life Technologies/Invitrogen Co., Carlsbad, CA) and analyzed with a Typhoon 9400 multi-mode imager (GE Healthcare, Pittsburgh, PA). Experiments were done in triplicate. After background subtraction, densities of antigen bands (determined with Imagequant software) were normalized to H levels and values averaged. Significance of H versus GCP levels of immunoreactivity was determined using Student's t test. To assess the specificity of the other antibodies used for immunofluorescence we followed the same electrophoresis and Western blot protocols.

### Immunofluorescence analysis of neurons in culture

Hippocampi from 18-day gestation Sprague-Dawley rat fetus were cut into explants or dissociated with trypsin. Explants or dissociated hippocampal pyramidal neurons were cultured on laminin-coated coverslips (Assistant brand) in B27 neurobasal medium supplemented with 10% v/v fetal bovine serum for 1–3 hours and then in serum-free B27 medium. After 1–2 days incubation at 37°C, 5% CO_2_ neurons were fully polarized and processed for indirect immunofluorescence.

Cultures were fixed using slow infusion of fixative [4% w/v paraformaldehyde, 0.1 M phosphate buffer (pH 7.4), 120 mM glucose, 0.4 mM CaCl_2_] as developed for electron microscopic analysis [56]. Cultures were rinsed (3×) with PBS containing 1 mM glycine, permeabilized with blocking buffer (PBS, 1% w/v bovine serum albumin) plus 1% (v/v) Brij™ 98 detergent for 2 min at room temperature, and washed (3×) with blocking buffer. Samples were labeled with primary antibody in blocking buffer for 1 hour at room temperature and washed 3× with blocking buffer. This process was repeated with Alexa Fluor 488-(green) or 594-(red) conjugated secondary antibodies and with red or green fluorescent phalloidin to label filamentous actin (Molecular Probes, Eugene, OR). These coverslips were mounted on slides using a polyvinyl alcohol/glycerol anti-fade medium. Samples were analyzed with a Zeiss Axiovert 200 M microscope operated by Metamorph Software (Molecular Dynamics, Sunnyvale, CA) and equipped with Zeiss lenses, epifluorescence and total internal reflection fluorescence (TIRF) optics, and a Cook Sensicam digital camera.

## Supporting Information

Table S1 **This table provides full protein names for the gene symbols used throughout the text.**
(PDF)

Table S2 **Proteins identified in isolated axonal growth cones, together with their gi accession numbers, molecular weights (Mol W) and numbers of identifying spectra detected in the gross GCP (isolated growth cones) preparation, background (Bkgd; composed of control and laminin coat of wells; see narrative), and net GCP preparation (values shown as≥0).** Note that, in a few cases, accession numbers refer to mouse or human rather than rat proteins.
(PDF)

## References

[1] Letourneau PC (1996). The cytoskeleton in nerve growth cone motility and axonal pathfinding.. Perspect Dev Neurobiol.

[2] Pfenninger KH (2009). Plasma membrane expansion: a neuron's Herculean task.. Nat Rev Neurosci.

[3] Conde C, Caceres A (2009). Microtubule assembly, organization and dynamics in axons and dendrites.. Nat Rev Neurosci.

[4] Tessier-Lavigne M, Goodman CS (1996). The molecular biology of axon guidance.. Science.

[5] O'Donnell M, Chance RK, Bashaw GJ (2009). Axon growth and guidance: receptor regulation and signal transduction.. Annu Rev Neurosci.

[6] Lohse K, Helmke SM, Wood MR, Quiroga S, de la Houssaye BA (1996). Axonal origin and purity of growth cones isolated from fetal rat brain.. Brain Res Dev Brain Res.

[7] Pfenninger KH, Ellis L, Johnson MP, Friedman LB, Somlo S (1983). Nerve growth cones isolated from fetal rat brain: subcellular fractionation and characterization.. Cell.

[8] Meiri KF, Pfenninger KH, Willard MB (1986). Growth-associated protein, GAP-43, a polypeptide that is induced when neurons extend axons, is a component of growth cones and corresponds to pp46, a major polypeptide of a subcellular fraction enriched in growth cones.. Proc Natl Acad Sci U S A.

[9] Nozumi M, Togano T, Takahashi-Niki K, Lu J, Honda A (2009). Identification of functional marker proteins in the mammalian growth cone.. Proc Natl Acad Sci U S A.

[10] Dennis G, Sherman BT, Hosack DA, Yang J, Gao W (2003). DAVID: Database for Annotation, Visualization, and Integrated Discovery.. Genome Biol.

[11] Huang da W, Sherman BT, Lempicki RA (2009). Systematic and integrative analysis of large gene lists using DAVID bioinformatics resources.. Nat Protoc.

[12] Huang da W, Sherman BT, Lempicki RA (2009). Bioinformatics enrichment tools: paths toward the comprehensive functional analysis of large gene lists.. Nucleic Acids Res.

[13] Dotti CG, Banker GA, Binder LI (1987). The expression and distribution of the microtubule-associated proteins tau and microtubule-associated protein 2 in hippocampal neurons in the rat in situ and in cell culture.. Neuroscience.

[14] Ferreira A, Busciglio J, Caceres A (1987). An immunocytochemical analysis of the ontogeny of the microtubule-associated proteins MAP-2 and Tau in the nervous system of the rat.. Brain Res.

[15] Kosik KS, Finch EA (1987). MAP2 and tau segregate into dendritic and axonal domains after the elaboration of morphologically distinct neurites: an immunocytochemical study of cultured rat cerebrum.. J Neurosci.

[16] Rothstein JD, Martin L, Levey AI, Dykes-Hoberg M, Jin L (1994). Localization of neuronal and glial glutamate transporters.. Neuron.

[17] Schmidt EF, Strittmatter SM (2007). The CRMP family of proteins and their role in Sema3A signaling.. Adv Exp Med Biol.

[18] Nguyen VQ, Co C, Irie K, Li JJ (2000). Clb/Cdc28 kinases promote nuclear export of the replication initiator proteins Mcm2-7.. Curr Biol.

[19] Naryzhny SN, Lee H (2004). The post-translational modifications of proliferating cell nuclear antigen: acetylation, not phosphorylation, plays an important role in the regulation of its function.. J Biol Chem.

[20] Vriz S, Lemaitre JM, Leibovici M, Thierry N, Mechali M (1992). Comparative analysis of the intracellular localization of c-Myc, c-Fos, and replicative proteins during cell cycle progression.. Mol Cell Biol.

[21] Negre-Aminou P, Pfenninger KH (1993). Arachidonic acid turnover and phospholipase A2 activity in neuronal growth cones.. J Neurochem.

[22] Kornfeld R, Kornfeld S (1985). Assembly of asparagine-linked oligosaccharides.. Annu Rev Biochem.

[23] Dunphy WG, Rothman JE (1983). Compartmentation of asparagine-linked oligosaccharide processing in the Golgi apparatus.. J Cell Biol.

[24] Wu CC, Taylor RS, Lane DR, Ladinsky MS, Weisz JA (2000). GMx33: a novel family of trans-Golgi proteins identified by proteomics.. Traffic.

[25] Jones SM, Crosby JR, Salamero J, Howell KE (1993). A cytosolic complex of p62 and rab6 associates with TGN38/41 and is involved in budding of exocytic vesicles from the trans-Golgi network.. J Cell Biol.

[26] Perry RB, Fainzilber M (2009). Nuclear transport factors in neuronal function.. Semin Cell Dev Biol.

[27] Otis KO, Thompson KR, Martin KC (2006). Importin-mediated nuclear transport in neurons.. Curr Opin Neurobiol.

[28] Harel A, Forbes DJ (2004). Importin beta: conducting a much larger cellular symphony.. Mol Cell.

[29] Pertz OC, Wang Y, Yang F, Wang W, Gay LJ (2008). Spatial mapping of the neurite and soma proteomes reveals a functional Cdc42/Rac regulatory network.. Proc Natl Acad Sci U S A.

[30] Zivraj KH, Tung YC, Piper M, Gumy L, Fawcett JW (2010). Subcellular profiling reveals distinct and developmentally regulated repertoire of growth cone mRNAs.. J Neurosci.

[31] Jeffery CJ (2003). Moonlighting proteins: old proteins learning new tricks.. Trends Genet.

[32] Giuditta A, Kaplan BB, van Minnen J, Alvarez J, Koenig E (2002). Axonal and presynaptic protein synthesis: new insights into the biology of the neuron.. Trends Neurosci.

[33] Campbell DS, Holt CE (2001). Chemotropic responses of retinal growth cones mediated by rapid local protein synthesis and degradation.. Neuron.

[34] Zheng JQ, Kelly TK, Chang B, Ryazantsev S, Rajasekaran AK (2001). A functional role for intra-axonal protein synthesis during axonal regeneration from adult sensory neurons.. J Neurosci.

[35] Brittis PA, Lu Q, Flanagan JG (2002). Axonal protein synthesis provides a mechanism for localized regulation at an intermediate target.. Cell.

[36] Brunet I, Weinl C, Piper M, Trembleau A, Volovitch M (2005). The transcription factor Engrailed-2 guides retinal axons.. Nature.

[37] Wu KY, Hengst U, Cox LJ, Macosko EZ, Jeromin A (2005). Local translation of RhoA regulates growth cone collapse.. Nature.

[38] Willis D, Li KW, Zheng JQ, Chang JH, Smit A (2005). Differential transport and local translation of cytoskeletal, injury-response, and neurodegeneration protein mRNAs in axons.. J Neurosci.

[39] Willis DE, Twiss JL (2006). The evolving roles of axonally synthesized proteins in regeneration.. Curr Opin Neurobiol.

[40] Hengst U, Jaffrey SR (2007). Function and translational regulation of mRNA in developing axons.. Semin Cell Dev Biol.

[41] Lin AC, Holt CE (2008). Function and regulation of local axonal translation.. Curr Opin Neurobiol.

[42] Bassell GJ, Zhang H, Byrd AL, Femino AM, Singer RH (1998). Sorting of beta-actin mRNA and protein to neurites and growth cones in culture.. J Neurosci.

[43] Bi J, Tsai NP, Lin YP, Loh HH, Wei LN (2006). Axonal mRNA transport and localized translational regulation of kappa-opioid receptor in primary neurons of dorsal root ganglia.. Proc Natl Acad Sci U S A.

[44] Merianda TT, Lin AC, Lam JS, Vuppalanchi D, Willis DE (2009). A functional equivalent of endoplasmic reticulum and Golgi in axons for secretion of locally synthesized proteins.. Mol Cell Neurosci.

[45] Mann F, Miranda E, Weinl C, Harmer E, Holt CE (2003). B-type Eph receptors and ephrins induce growth cone collapse through distinct intracellular pathways.. J Neurobiol.

[46] Verma P, Chierzi S, Codd AM, Campbell DS, Meyer RL (2005). Axonal protein synthesis and degradation are necessary for efficient growth cone regeneration.. J Neurosci.

[47] Drinjakovic J, Jung H, Campbell DS, Strochlic L, Dwivedy A (2010). E3 ligase Nedd4 promotes axon branching by downregulating PTEN.. Neuron.

[48] Mayer MP, Bukau B (2005). Hsp70 chaperones: cellular functions and molecular mechanism.. Cell Mol Life Sci.

[49] Doyle SM, Wickner S (2009). Hsp104 and ClpB: protein disaggregating machines.. Trends Biochem Sci.

[50] Koike M, Fukushi J, Ichinohe Y, Higashimae N, Fujishiro M (2010). Valosin-containing protein (VCP) in novel feedback machinery between abnormal protein accumulation and transcriptional suppression.. J Biol Chem.

[51] Casey PJ, Solski PA, Der CJ, Buss JE (1989). p21ras is modified by a farnesyl isoprenoid.. Proc Natl Acad Sci U S A.

[52] Gumy LF, Yeo GS, Tung YC, Zivraj KH, Willis D (2011). Transcriptome analysis of embryonic and adult sensory axons reveals changes in mRNA repertoire localization.. RNA.

[53] Fulga TA, Van Vactor D (2008). Synapses and growth cones on two sides of a highwire.. Neuron.

[54] Murphey RK, Godenschwege TA (2002). New roles for ubiquitin in the assembly and function of neuronal circuits.. Neuron.

[55] Wessel D, Flugge UI (1984). A method for the quantitative recovery of protein in dilute solution in the presence of detergents and lipids.. Anal Biochem.

[56] Pfenninger KH, Maylie-Pfenninger MF (1981). Lectin labeling of sprouting neurons. I. Regional distribution of surface glycoconjugates.. J Cell Biol.

