# Supplementary material for: Functional Complexity of the Axonal Growth Cone: A Proteomic Analysis
Source: PLoS One. 2012 Feb 27;7(2):e31858. doi: 10.1371/journal.pone.0031858 (PMC3288056; doi:10.1371/journal.pone.0031858)
Supplement: Table S1 — This table provides full protein names for the gene symbols used throughout the text. (PDF) [file pone.0031858.s001.pdf]

Table S1: Gene Symbols and Protein Names

Gene Symbol	Protein Name
Aars	alanyl-tRNA synthetase
Actb	actin, beta
Actc1	actin, alpha, cardiac muscle 1
Actg1	actin, gamma 1
Actn4	actinin alpha 4
Actr3	ARP3 actin-related protein 3 homolog (yeast)
Add1	adducin 1 (alpha)
Afp	alpha-fetoprotein
Alb	albumin
Aldoa	aldolase A, fructose-bisphosphate
Aldoc	aldolase C, fructose-bisphosphate
Anapc1	anaphase promoting complex subunit 1
Apex1	APEX nuclease (multifunctional DNA repair enzyme) 1
Apoa1	apolipoprotein A-I
Apob	apolipoprotein B
Arf1	ADP-ribosylation factor 1
Arf3	ADP-ribosylation factor 3
Arf5	ADP-ribosylation factor 5
Arpc2	actin related protein 2/3 complex, subunit 2
Arpc4	actin related protein 2/3 complex, subunit 4
Atp5a	ATP synthase H^+ transporting, mitochondrial F1 complex, alpha subunit 1 (human)
Atp5b	ATP synthase, H^+ transporting, mitochondrial F1 complex, beta polypeptide
Basp1	brain abundant, membrane attached signal protein 1
Cadps	Ca++-dependent secretion activator
Canx	calnexin
Cap1	CAP, adenylate cyclase-associated protein 1 (yeast)
Cct2	chaperonin containing TCP1, subunit 2 (beta)
Cct3	chaperonin containing Tcp1, subunit 3 (gamma)
Cct4	chaperonin containing Tcp1, subunit 4 (delta)
Cct5	chaperonin containing Tcp1, subunit 5 (epsilon)
Cct6a	chaperonin containing Tcp1, subunit 6A (zeta 1)
Cct7	chaperonin containing Tcp1, subunit 7 (eta)
Cct8	chaperonin containing Tcp1, subunit 8 (theta)
Cdc42	cell division cycle 42 (GTP binding protein)
Cdk5	cyclin-dependent kinase 5
Cfl1	cofilin 1, non-muscle
Ckb	creatine kinase, brain
Clasp2	cytoplasmic linker associated protein 2
Cltc	clathrin, heavy chain (Hc)
Copa	coatomer protein complex subunit alpha
Copb1	coatomer protein complex, subunit beta 1
Copb2	coatomer protein complex, subunit beta 2 (beta prime)
Copg	coatomer protein complex, subunit gamma
Crmp1	collapsin response mediator protein 1
Cul3	cullin 3

Gene Symbol	Protein Name
Cyb5r3	cytochrome b5 reductase 3
Dbn1	drebrin 1
Dctn2	dynactin 2
Dpysl2	dihydropyrimidinase-like 2
Dpysl3	dihydropyrimidinase-like 3
Dpysl5	dihydropyrimidinase-like 5
Dync1h1	dynein cytoplasmic 1 heavy chain 1
Ecm29	proteasome-associated protein ECM29 homolog
Eef1a1	eukaryotic translation elongation factor 1 alpha 1
Eef1b2	eukaryotic translation elongation factor 1 beta 2
Eef1g	eukaryotic translation elongation factor 1 ga
Eef2	eukaryotic translation elongation factor 2
Eif2b5	eukaryotic translation initiation factor 2B, subunit 5 epsilon
Eif3a	eukaryotic translation initiation factor 3, subunit A
Eif3b	eukaryotic translation initiation factor 3, subunit B
Eif4a1	eukaryotic translation initiation factor 4A1
Eif4g1	eukaryotic translation initiation factor 4 gamma, 1
Enah	enabled homolog (Drosophila)
Eno1	enolase 1, (alpha)
Eno2	enolase 2, gamma, neuronal
Ephb2	Eph receptor B2
Exoc8	exocyst complex component 8
Fasn	fatty acid synthase
Fdps	farnesyl diphosphate synthase (farnesyl pyrophosphate synthetase, dimethylallyltranstransferase, geranyltranstransferase)
Gap43	growth associated protein 43
Gapdh	glyceraldehyde-3-phosphate dehydrogenase
Gars	glycyl-tRNA synthetase
Gdi1	GDP dissociation inhibitor 1
Gdi2	GDP dissociation inhibitor 2
Glrx3	glutaredoxin 3
Golph3	Golgi phosphoprotein 3 (GMx33)
Gpi	glucose phosphate isomerase
Hdac6	histone deacetylase 6
Hist1h2bl	histone cluster 1, H2bl
Hist3h2ba	histone cluster 3, H2ba
Hmgcs1	3-hydroxy-3-methylglutaryl-Coenzyme A synthase 1 (soluble)
Hsp90aa1	heat shock protein 90, alpha (cytosolic), class A member 1
Hsp90ab1	heat shock protein 90kDa alpha (cytosolic), class B member 1
Hspa4	heat shock protein 4
Hspa8	heat shock protein A8
Idi1	isopentenyl-diphosphate delta isomerase 1
Ipo5	Importin 5
Kif1 (-21)	kinesin family member 1 (-21)
Kif5c	kinesin family member 5C
Kpna2	karyopherin α2
Kpnb1	karyopherin (importin) beta 1
Ldhb	lactate dehydrogenase B
Man2a1	mannosidase alpha, class 2A (member 1)

Gene Symbol	Protein Name
Map1b	microtubule-associated protein 1B
Map2b	Microtubule-associated protein 2 (isoform B)
Mapre1	microtubule-associated protein, RP/EB family, member 1
Mapt	microtubule-associated protein tau
Mcm7	minichromosome maintenance protein 7
Mvd	mevalonate (diphospho) decarboxylase
Myh10	myosin, heavy chain 10, non-muscle
Ncam1	neural cell adhesion molecule 1
Nckap1	NCK-associated protein 1
Nrcam	neuronal cell adhesion molecule
Nrp1	neuropilin 1
Pdia3	protein disulfide isomerase family A, member 3
Pdia6	protein disulfide isomerase A6
Pena	proliferating cell nuclear antigen
Pkm2	pyruvate kinase, muscle
Plxna1	Plexin A1 (mouse)
Plxna2	plexin A2
Plxnb2	plexin B2
Ppia	peptidylprolyl isomerase A (cyclophilin A)
Ppp2r1a	protein phosphatase 2 (formerly 2A), regulatory subunit A, alpha isoform
Prdx1	peroxiredoxin 1
Prdx2	peroxiredoxin 2
Psma1 - Psma7	proteasome (prosome, macropain) subunit, alpha type 1 (-7)
Psmb1 - Psmb7	proteasome (prosome, macropain) subunit, beta type 1 (-7)
Psmc1 - Psmc6	proteasome (prosome, macropain) 26S subunit, ATPase 1 (-6)
Psmd1	proteasome (prosome, macropain) 26S subunit, non-ATPase, 1
Psmd1 - Psmd14	proteasome (prosome, macropain) 26S subunit, non-ATPase, 1 (-14)
Psme1 - Psme4	proteasome (prosome, macropain) activator subunit 1 (-4)
Psme3	proteasome activator complex, subunit 3
Qars	glutaminyl-tRNA synthetase
Rab1 (-40)	Ras-related protein 1 (-40)
Rac1	ras-related C3 botulinum toxin substrate 1
Rad23b	RAD23 homolog B (S. cerevisiae)
Rala	v-ral simian leukemia viral oncogene homolog A (ras related)
Rars	arginyl-tRNA synthetase
Robo2	roundabout, axon guidance receptor, homolog 2 (Drosophila)
Rpn1	dolichyl-diphospho-oligosaccharide-protein glycosyltransferase (ribophorin)
Rps3	ribosomal protein S3
Rtf1	Rtf1, Paf1/RNA polymerase II complex component, homolog (S. cerevisiae)
Sar1a	SAR1 homolog A (S. cerevisiae)
Sec31a	SEC31 homolog A (S. cerevisiae)
Slc1a2	solute carrier family 1 (glial high affinity glutamate transporter), member 2
Slc1a3	solute carrier family 1 (glial high affinity glutamate transporter), member 3
Snap25	synaptosomal-associated protein 25
Stx1b	syntaxin 1B
Stxbp1	syntaxin binding protein 1
Tcp1	t-complex 1
Tf	transferrin
Tgoln1	trans-golgi network protein (TGN38)

Gene Symbol	Protein Name
Tkt	transketolase
Tmx1	Thioredoxin-related transmembrane protein 1 (Txndc1)
Tpi1	Triose phosphate isomerase 1
Tpm3	tropomyosin 3, gamma
Tuba1a	tubulin, alpha 1A
Tubb2b	tubulin, beta 2b
Tubb3	tubulin, beta 3
Tubb5	tubulin, beta 5
Txnrd1	thioredoxin reductase 1
Uba1	ubiquitin-like modifier activating enzyme 1
Ube2o	ubiquitin-conjugating enzyme E2O
Ube3a	ubiquitin protein ligase E3A
Ubqln2	ubiquilin 2
Ubr4	ubiquitin protein ligase E3 component n-recognin 4
Uchl1	ubiquitin carboxyl-terminal esterase L1 (ubiquitin thiolesterase)
Uggt1	UDP-glucose glycoprotein glucosyltransferase 1
Uso1	USO1 homolog, vesicle docking protein (yeast)
Vapa	VAMP (vesicle-associated membrane protein)-associated protein A
Vcp	valosin-containing protein
Vps29	vacuolar protein sorting 29 homolog (S. cerevisiae)
Ywhae	tyrosine 3-monooxygenase/tryptophan 5-monooxygenase activation protein, epsilon polypeptide
Ywhaz	tyrosine 3-monooxygenase/tryptophan 5-monooxygenase activation protein, zeta polypeptide
