# Supplementary material for: Functional Complexity of the Axonal Growth Cone: A Proteomic Analysis
Source: PLoS One. 2012 Feb 27;7(2):e31858. doi: 10.1371/journal.pone.0031858 (PMC3288056; doi:10.1371/journal.pone.0031858)
Supplement: Table S2 — Proteins identified in isolated axonal growth cones, together with their gi accession numbers, molecular weights (Mol W) and numbers of identifying spectra detected in the gross GCP (isolated growth cones) preparation, background (Bkgd; composed of control and laminin coat of wells; see narrative), and net GCP preparation (values shown as≥0). Note that, in a few cases, accession numbers refer to mouse or human rather than rat proteins. (PDF) [file pone.0031858.s002.pdf]

**Table S2: Proteins Identified in Isolated Axonal Growth Cones.**
(Mol W, molecular weight; GCP, isolated growth cones, Bkgd, background; Net GCP = gross GCP minus background, shown as $\geq$ 0).

| #20090521_Pfenninger_Scaffold_03, Samples Report created on 06/10/2010 | | | | ----- Number of identifying spectra ----- | | | | |
|---|---|---|---|---|---|---|---|---|
| **#** | **Identified Proteins (2130)** | **Accession Number** | **Mol W** | **Gross GCP** | **Control** | **Laminin** | **Bkgd** | **Net GCP** |
| 1 | tubulin, alpha 1 [Mus musculus], gi\|11560133\|ref\|NP_071634.1\| tubulin, alpha 1A [Rattus norvegicus], gi\|17986283\|ref\|NP_006000.2\| tubulin, alpha 1a [Homo sapiens], gi\|148727361\|ref\|NP_001092014.1\| tubulin, alpha 1a [Pan troglodytes], gi\|57106374\|ref\|XP_53 | gi\|6755901 | 50 | 7148 | 1596 | 1553 | 2056 | 5092 |
| 2 | tubulin T beta15 | gi\|224839 | 50 | 5263 | 1505 | 1063 | 1537 | 3726 |
| 3 | actin, gamma 1 propeptide [Homo sapiens], gi\|6752954\|ref\|NP_033739.1\| actin, gamma, cytoplasmic 1 [Mus musculus], gi\|75812932\|ref\|NP_001028790.1\| actin, gamma 1 [Bos taurus], gi\|147901470\|ref\|NP_001091279.1\| hypothetical protein LOC100037088 [Xenopus laev | gi\|4501887 | 42 | 3206 | 1109 | 849 | 1198 | 2008 |
| 4 | microtubule-associated protein 1b, isoform CRA_a [Rattus norvegicus] | gi\|149059169 | 256 | 3130 | 414 | 692 | 822 | 2308 |
| 5 | dihydropyrimidinase-like 2 [Mus musculus], gi\|157786744\|ref\|NP_001099187.1\| dihydropyrimidinase-like 2 [Rattus norvegicus], gi\|1351260\|sp\|P47942.1\|DPYL2_RAT RecName: Full=Dihydropyrimidinase-related protein 2; Short=DRP-2; AltName: Full=Turned on after di | gi\|40254595 | 62 | 3069 | 499 | 570 | 727 | 2342 |
| 6 | cytoplasmic dynein 1 heavy chain 1 [Rattus norvegicus], gi\|294543\|gb\|AAA41103.1\| dynein heavy chain, gi\|149044126\|gb\|EDL97508.1\| rCG27764, isoform CRA_a [Rattus norvegicus] | gi\|148491097 | 532 | 2434 | 172 | 831 | 885 | 1549 |
| 7 | RecName: Full=Fatty acid synthase; Includes: RecName: Full=[Acyl-carrier-protein] S-acetyltransferase; Includes: RecName: Full=[Acyl-carrier-protein] S-malonyltransferase; Includes: RecName: Full=3-oxoacyl-[acyl-carrier-protein] synthase; Includes: RecNam | gi\|2506136 | 273 | 2159 | 435 | 528 | 665 | 1494 |
| 8 | heat shock protein 90, alpha (cytosolic), class A member 1 [Rattus norvegicus], gi\|122065208\|sp\|P82995.3\|HS90A_RAT RecName: Full=Heat shock protein HSP 90-alpha; AltName: Full=HSP 86, gi\|14270366\|emb\|CAC39453.1\| heat shock protein 86 [Rattus norvegicus], | gi\|28467005 | 85 | 1575 | 384 | 305 | 426 | 1149 |
| 9 | ubiquitin-activating enzyme E1 [Rattus norvegicus], gi\|81889667\|sp\|Q5U300.1\|UBA1_RAT RecName: Full=Ubiquitin-like modifier-activating enzyme 1; AltName: Full=Ubiquitin-activating enzyme E1, gi\|55250575\|gb\|AAH85791.1\| Ubiquitin-like modifier activating enz | gi\|62078893 | 118 | 1549 | 472 | 320 | 469 | 1080 |
| 10 | collapsin response mediator protein | gi\|1518520 (+1) | 62 | 1470 | 229 | 347 | 419 | 1051 |
| 11 | tubulin, beta 5 [Mus musculus], gi\|27465535\|ref\|NP_775125.1\| tubulin, beta 5 [Rattus norvegicus], gi\|29788785\|ref\|NP_821133.1\| tubulin, beta [Homo sapiens], gi\|45384338\|ref\|NP_990646.1\| tubulin, beta 2 [Gallus gallus], gi\|74136187\|ref\|NP_001027985.1\| tubu | gi\|7106439 | 50 | 1398 | 351 | 351 | 462 | 936 |
| 12 | TUC-4b [Rattus norvegicus], gi\|149017450\|gb\|EDL76501.1\| dihydropyrimidinase-like 3, isoform CRA_d [Rattus norvegicus] | gi\|21666559 | 74 | 1436 | 214 | 242 | 309 | 1127 |
| 13 | glyceraldehyde-3-phosphate dehydrogenase [Rattus norvegicus], gi\|109475159\|ref\|XP_001062726.1\| PREDICTED: similar to glyceraldehyde-3-phosphate dehydrogenase [Rattus norvegicus], gi\|109476813\|ref\|XP_001059327.1\| PREDICTED: similar to glyceraldehyde-3-phos | gi\|8393418 | 36 | 1487 | 221 | 252 | 322 | 1165 |
| 14 | tubulin, beta 3 [Mus musculus], gi\|145966774\|ref\|NP_640347.2\| tubulin, beta 3 [Rattus norvegicus], gi\|20455323\|sp\|Q9ERD7.1\|TBB3_MOUSE RecName: Full=Tubulin beta-3 chain, gi\|81918226\|sp\|Q4QRB4.1\|TBB3_RAT RecName: Full=Tubulin beta-3 chain; AltName: Full=Ne | gi\|12963615 | 50 | 1229 | 292 | 264 | 356 | 873 |
| 15 | valosin-containing protein [Rattus norvegicus], gi\|1174637\|sp\|P46462.3\|TERA_RAT RecName: Full=Transitional endoplasmic reticulum ATPase; Short=TER ATPase; AltName: Full=15S Mg(2+)-ATPase p97 subunit; AltName: Full=Valosin-containing protein; Short=VCP, gi | gi\|17865351 | 89 | 1316 | 213 | 84 | 151 | 1165 |
| 16 | eukaryotic translation elongation factor 1 alpha 1 [Rattus norvegicus], gi\|126032329\|ref\|NP_034236.2\| eukaryotic translation elongation factor 1 alpha 1 [Mus musculus], gi\|50402095\|sp\|P62630.1\|EF1A1_RAT RecName: Full=Elongation factor 1-alpha 1; Short=EF- | gi\|28460696 | 50 | 1103 | 210 | 285 | 351 | 752 |
| 17 | RecName: Full=Creatine kinase B-type; AltName: Full=Creatine kinase B chain; AltName: Full=B-CK, gi\|127798304\|gb\|AAH65307.2\| Creatine kinase, brain [Rattus norvegicus], gi\|127799192\|gb\|AAH70955.2\| Creatine kinase, brain [Rattus norvegicus], gi\|127800417\|g | gi\|122065316 | 43 | 1148 | 261 | 187 | 269 | 879 |
| 18 | clathrin, heavy chain (Hc) [Rattus norvegicus], gi\|116514\|sp\|P11442.3\|CLH_RAT RecName: Full=Clathrin heavy chain 1, gi\|203302\|gb\|AAA40874.1\| clathrin heavy chain | gi\|9506497 | 192 | 1055 | 288 | 259 | 350 | 705 |
| 19 | tyrosine 3/tryptophan 5 -monooxygenase activation protein, epsilon polypeptide [Homo sapiens], gi\|27806197\|ref\|NP_776916.1\| tyrosine 3-monooxygenase/tryptophan 5-monooxygenase activation protein, epsilon polypeptide [Bos taurus], gi\|55741616\|ref\|NP_001006 | gi\|5803225 | 29 | 890 | 272 | 73 | 159 | 731 |
| 20 | eukaryotic translation elongation factor 2 [Rattus norvegicus], gi\|119176\|sp\|P05197.4\|EF2_RAT RecName: Full=Elongation factor 2; Short=EF-2, gi\|56082\|emb\|CAA68805.1\| unnamed protein product [Rattus norvegicus], gi\|44890252\|gb\|AAH66661.1\| Eukaryotic transl | gi\|8393296 | 95 | 943 | 221 | 104 | 174 | 769 |
| 21 | chaperonin containing TCP1, subunit 2 (beta) [Rattus norvegicus], gi\|81910374\|sp\|Q5XIM9.3\|TCPB_RAT RecName: Full=T-complex protein 1 subunit beta; Short=TCP-1-beta; AltName: Full=CCT-beta, gi\|53733839\|gb\|AAH83650.1\| Chaperonin containing TCP1, subunit 2 ( | gi\|54400730 | 57 | 1008 | 59 | 229 | 248 | 760 |
| 22 | myosin, heavy polypeptide 10, non-muscle, isoform CRA_b [Rattus norvegicus] | gi\|149052999 | 229 | 772 | 222 | 199 | 269 | 503 |
| 23 | dihydropyrimidinase-like 5 [Rattus norvegicus], gi\|20137606\|sp\|Q9JHU0.1\|DPYL5_RAT RecName: Full=Dihydropyrimidinase-related protein 5; Short=DRP-5; AltName: Full=ULIP6 protein, gi\|8671360\|emb\|CAB95193.1\| Ulip-like protein [Rattus norvegicus], gi\|149050805 | gi\|12711692 | 62 | 802 | 99 | 122 | 153 | 649 |
| 24 | heat shock protein 1, beta [Mus musculus], gi\|148747365\|ref\|NP_001004082.3\| heat shock protein 90kDa alpha (cytosolic), class B member 1 [Rattus norvegicus], gi\|122065211\|sp\|P34058.4\|HS90B_RAT RecName: Full=Heat shock protein HSP 90-beta; AltName: Full=HS | gi\|40556608 (+1) | 83 | 680 | 225 | 153 | 224 | 456 |
| 25 | neural cell adhesion molecule 1, isoform CRA_a [Rattus norvegicus] | gi\|149041605 | 108 | 871 | 85 | 79 | 106 | 765 |
| 26 | t-complex 1 [Rattus norvegicus], gi\|135539\|sp\|P28480.1\|TCPA_RAT RecName: Full=T-complex protein 1 subunit alpha; Short=TCP-1-alpha; AltName: Full=CCT-alpha, gi\|220915\|dbj\|BAA14357.1\| t complex polypeptide 1 [Rattus norvegicus], gi\|149027451\|gb\|EDL83041.1\| | gi\|6981642 | 60 | 850 | 28 | 186 | 195 | 655 |
| 27 | microtubule-associated protein 2, isoform CRA_b [Rattus norvegicus], gi\|149016021\|gb\|EDL75302.1\| microtubule-associated protein 2, isoform CRA_b [Rattus norvegicus] | gi\|149016020 | 198 | 507 | 345 | 125 | 234 | 273 |
| 28 | chaperonin containing Tcp1, subunit 6A (zeta 1) [Rattus norvegicus], gi\|75773247\|gb\|AAI04704.1\| Chaperonin containing Tcp1, subunit 6A (zeta 1) [Rattus norvegicus], gi\|149063164\|gb\|EDM13487.1\| chaperonin subunit 6a (zeta) [Rattus norvegicus] | gi\|76253725 | 58 | 753 | 32 | 164 | 174 | 579 |
| 29 | chaperonin containing Tcp1, subunit 7 (eta) [Rattus norvegicus], gi\|149036575\|gb\|EDL91193.1\| rCG55994, isoform CRA_c [Rattus norvegicus] | gi\|157819651 | 60 | 718 | 26 | 147 | 155 | 563 |
| 30 | chaperonin containing TCP1, subunit 5 (epsilon) [Rattus norvegicus], gi\|81910761\|sp\|Q68FQ0.1\|TCPE_RAT RecName: Full=T-complex protein 1 subunit epsilon; Short=TCP-1-epsilon; AltName: Full=CCT-epsilon, gi\|51260037\|gb\|AAH79441.1\| Chaperonin containing Tcp1, | gi\|51890219 | 60 | 702 | 33 | 172 | 182 | 520 |
| 31 | heat shock protein 8 [Rattus norvegicus], gi\|31981690\|ref\|NP_112442.2\| heat shock protein 8 [Mus musculus], gi\|148234651\|ref\|NP_001091238.1\| hypothetical protein LOC100037033 [Xenopus laevis], gi\|51702273\|sp\|P63018.1\|HSP7C_RAT RecName: Full=Heat shock cog | gi\|13242237 (+1) | 71 | 641 | 212 | 37 | 104 | 537 |
| 32 | alpha-fetoprotein, isoform CRA_a [Rattus norvegicus] | gi\|149033755 | 68 | 212 | 621 | 4 | 200 | 12 |
| 33 | GDP dissociation inhibitor 1 [Rattus norvegicus], gi\|1707888\|sp\|P50398.1\|GDIA_RAT RecName: Full=Rab GDP dissociation inhibitor alpha; Short=Rab GDI alpha; AltName: Full=Guanosine diphosphate dissociation inhibitor 1; Short=GDI-1, gi\|71122463\|gb\|AAH99763.1 | gi\|71534276 | 51 | 606 | 220 | 64 | 133 | 473 |
| 34 | alpha isoform of regulatory subunit A, protein phosphatase 2 [Mus musculus], gi\|47522870\|ref\|NP_999189.1\| alpha isoform of regulatory subunit A, protein phosphatase 2 [Sus scrofa], gi\|55926139\|ref\|NP_476481.1\| alpha isoform of regulatory subunit A, protei | gi\|8394027 | 65 | 557 | 68 | 133 | 154 | 403 |
| 35 | chaperonin subunit 4 (delta) [Rattus norvegicus], gi\|52000745\|sp\|Q7TPB1.3\|TCPD_RAT RecName: Full=T-complex protein 1 subunit delta; Short=TCP-1-delta; AltName: Full=CCT-delta, gi\|33149357\|gb\|AAP46161.1\| chaperonin delta subunit [Rattus norvegicus], gi\|509 | gi\|33414505 | 58 | 543 | 32 | 156 | 166 | 377 |
| 36 | tyrosine 3-monooxygenase/tryptophan 5-monooxygenase activation protein, zeta polypeptide [Mus musculus], gi\|62990183\|ref\|NP_037143.2\| tyrosine 3-monooxygenase/tryptophan 5-monooxygenase activation protein, zeta polypeptide [Rattus norvegicus], gi\|52000883 | gi\|6756041 | 28 | 546 | 157 | 63 | 112 | 434 |
| 37 | PREDICTED: similar to RAN binding protein 5 [Rattus norvegicus], gi\|109502869\|ref\|XP_001075101.1\| PREDICTED: similar to RAN binding protein 5 [Rattus norvegicus] | gi\|109502015 | 124 | 518 | 135 | 106 | 149 | 369 |
| 38 | proteasome (prosome, macropain) 26S subunit, non-ATPase, 1, isoform CRA_a [Rattus norvegicus] | gi\|149016321 | 106 | 555 | 13 | 152 | 156 | 399 |
| 39 | GDP dissociation inhibitor 2 [Rattus norvegicus], gi\|116089273\|ref\|NP_032138.3\| guanosine diphosphate (GDP) dissociation inhibitor 2 [Mus musculus], gi\|13626886\|sp\|Q61598.1\|GDIB_MOUSE RecName: Full=Rab GDP dissociation inhibitor beta; Short=Rab GDI beta; | gi\|40254781 | 51 | 510 | 205 | 25 | 90 | 420 |
| 40 | karyopherin (importin) beta 1 [Rattus norvegicus], gi\|1708485\|sp\|P52296.1\|IMB1_RAT RecName: Full=Importin subunit beta-1; AltName: Full=Karyopherin subunit beta-1; AltName: Full=Nuclear factor P97, gi\|712839\|gb\|AAC42047.1\| karyopherin beta, gi\|1095175\|prf | gi\|8393610 | 97 | 549 | 106 | 42 | 75 | 474 |
| 41 | RecName: Full=Transketolase; Short=TK | gi\|1729977 | 68 | 518 | 101 | 95 | 127 | 391 |
| 42 | chaperonin containing TCP1, subunit 3 (gamma) [Rattus norvegicus], gi\|81911258\|sp\|Q6P502.1\|TCPG_RAT RecName: Full=T-complex protein 1 subunit gamma; Short=TCP-1-gamma; AltName: Full=CCT-gamma, gi\|38969850\|gb\|AAH63178.1\| Chaperonin containing Tcp1, subunit | gi\|40018616 | 61 | 574 | 20 | 85 | 91 | 483 |
| 43 | rCG45607, isoform CRA_a [Rattus norvegicus] | gi\|149039134 | 289 | 456 | 116 | 95 | 132 | 324 |
| 44 | tubulin, alpha 1B [Mus musculus], gi\|57013276\|ref\|NP_006073.2\| tubulin, alpha, ubiquitous [Homo sapiens], gi\|112984124\|ref\|NP_001037735.1\| tubulin, alpha 1B [Rattus norvegicus], gi\|113205626\|ref\|NP_001038009.1\| tubulin alpha [Sus scrofa], gi\|166796019\|ref | gi\|34740335 | 50 | 460 | 81 | 140 | 166 | 294 |
| 45 | tubulin, beta 4 [Homo sapiens], gi\|31981939\|ref\|NP_033477.2\| tubulin, beta 4 [Mus musculus], gi\|77736339\|ref\|NP_001029869.1\| tubulin, beta 4 [Bos taurus], gi\|158262004\|ref\|NP_543158.1\| tubulin, beta 4 [Rattus norvegicus], gi\|75075849\|sp\|Q4R4X8.1\|TBB4_MACF | gi\|21361322 | 50 | 436 | 88 | 145 | 173 | 263 |
| 46 | ubiquitin carboxyl-terminal esterase L1 [Rattus norvegicus], gi\|188219614\|ref\|NP_035800.2\| ubiquitin carboxyl-terminal esterase L1 [Mus musculus], gi\|18203410\|sp\|Q9R0P9.1\|UCHL1_MOUSE RecName: Full=Ubiquitin carboxyl-terminal hydrolase isozyme L1; Short=UC | gi\|61098212 | 25 | 405 | 107 | 11 | 45 | 360 |
| 47 | PREDICTED: similar to Protein neurobeachin (Lysosomal trafficking regulator 2) [Rattus norvegicus] | gi\|109466685 | 327 | 330 | 111 | 104 | 139 | 191 |
| 48 | triosephosphate isomerase 1 [Rattus norvegicus], gi\|124056485\|sp\|P48500.2\|TPIS_RAT RecName: Full=Triosephosphate isomerase; Short=TIM; AltName: Full=Triose-phosphate isomerase, gi\|117558438\|gb\|AAI26088.1\| Triosephosphate isomerase 1 [Rattus norvegicus] | gi\|117935064 (+2) | 27 | 393 | 131 | 54 | 95 | 298 |
| 49 | proteasome (prosome, macropain) 26S subunit, non-ATPase, 2 [Rattus norvegicus], gi\|123785726\|sp\|Q4FZT9.1\|PSMD2_RAT RecName: Full=26S proteasome non-ATPase regulatory subunit 2, gi\|71051911\|gb\|AAH99135.1\| Proteasome (prosome, macropain) 26S subunit, non-AT | gi\|72255509 | 100 | 403 | 27 | 110 | 119 | 284 |

| | | | | | | | | |
|---|---|---|---|---|---|---|---|---|
| 50 | heat shock protein 4 [Rattus norvegicus], gi\|81886881\|sp\|O88600.1\|HSP74_RAT RecName: Full=Heat shock 70 kDa protein 4; AltName: Full=Ischemia responsive 94 kDa protein, gi\|3360521\|gb\|AAC27937.1\| ischemia responsive 94 kDa protein [Rattus norvegicus] | gi\|24025637 | 94 | 368 | 145 | 31 | 77 | 291 |
| 51 | M2 pyruvate kinase [Rattus norvegicus], gi\|149041863\|gb\|EDL95704.1\| rCG57843, isoform CRA_a [Rattus norvegicus] | gi\|206205 | 58 | 427 | 92 | 20 | 49 | 378 |
| 52 | glucose phosphate isomerase [Rattus norvegicus], gi\|81892272\|sp\|Q6P6V0.1\|G6PI_RAT RecName: Full=Glucose-6-phosphate isomerase; Short=GPI; AltName: Full=Phosphoglucose isomerase; Short=PGI; AltName: Full=Phosphohexose isomerase; Short=PHI; AltName: Full=Au | gi\|46485440 | 63 | 386 | 89 | 58 | 86 | 300 |
| 53 | albumin [Rattus norvegicus], gi\|55391508\|gb\|AAH85359.1\| Albumin [Rattus norvegicus] | gi\|158138568 | 69 | 115 | 400 | 0 | 126 | 0 |
| 54 | chaperonin subunit 8 (theta) (predicted), isoform CRA_a [Rattus norvegicus] | gi\|149059759 | 60 | 455 | 21 | 67 | 74 | 381 |
| 55 | reticulon 4 [Rattus norvegicus], gi\|17367410\|sp\|Q9JK11.1\|RTN4_RAT RecName: Full=Reticulon-4; AltName: Full=Neurite outgrowth inhibitor; Short=Nogo protein; AltName: Full=Foocen; AltName: Full=Glut4 vesicle 20 kDa protein, gi\|6822247\|emb\|CAB71027.1\| Nogo-A | gi\|13929188 | 126 | 388 | 33 | 78 | 88 | 300 |
| 56 | ATP citrate lyase isoform 2 [Rattus norvegicus], gi\|149054226\|gb\|EDM06043.1\| ATP citrate lyase, isoform CRA_b [Rattus norvegicus] | gi\|161760632 (+1) | 120 | 372 | 54 | 78 | 95 | 277 |
| 57 | keratin 10 [Rattus norvegicus], gi\|81891690\|sp\|Q6IFW6.1\|K1C10_RAT RecName: Full=Keratin, type I cytoskeletal 10; AltName: Full=Cytokeratin-10; Short=CK-10; AltName: Full=Keratin-10; Short=K10; AltName: Full=Type I keratin Ka10, gi\|46485058\|tpg\|DAA04466.1\| | gi\|57012436 | 57 | 286 | 151 | 22 | 70 | 216 |
| 58 | guanine nucleotide-binding protein, beta-1 subunit [Mus musculus], gi\|11321585\|ref\|NP_002065.1\| guanine nucleotide-binding protein, beta-1 subunit [Homo sapiens], gi\|50979020\|ref\|NP_001003236.1\| guanine nucleotide-binding protein, beta-1 subunit [Canis lu | gi\|6680045 | 37 | 388 | 33 | 100 | 110 | 278 |
| 59 | ARP1 actin-related protein 1 homolog A, centractin alpha [Homo sapiens], gi\|8392847\|ref\|NP_058556.1\| ARP1 actin-related protein 1 homolog A, centractin alpha [Mus musculus], gi\|50978894\|ref\|NP_001003164.1\| ARP1 actin-related protein 1 homolog A, centracti | gi\|5031569 | 43 | 367 | 18 | 98 | 104 | 263 |
| 60 | ubiquitin specific peptidase 9, X-linked isoform 1 [Rattus norvegicus], gi\|149044325\|gb\|EDL97646.1\| ubiquitin specific peptidase 9, X chromosome (predicted) [Rattus norvegicus] | gi\|209571464 | 289 | 287 | 76 | 78 | 102 | 185 |
| 61 | tyrosine 3-monooxygenase/tryptophan 5-monooxygenase activation protein, theta polypeptide [Mus musculus], gi\|6981712\|ref\|NP_037185.1\| tyrosine 3-monooxygenase/tryptophan 5-monooxygenase activation protein, theta polypeptide [Rattus norvegicus], gi\|1267233 | gi\|6756039 | 28 | 404 | 83 | 29 | 55 | 349 |
| 62 | cullin-associated and neddylation-dissociated 1 [Rattus norvegicus], gi\|67460104\|sp\|P97536.1\|CAND1_RAT RecName: Full=Cullin-associated NEDD8-dissociated protein 1; AltName: Full=Cullin-associated and neddylation-dissociated protein 1; AltName: Full=p120 C | gi\|16758920 | 136 | 309 | 170 | 4 | 58 | 251 |
| 63 | keratin 1 [Rattus norvegicus], gi\|119850981\|gb\|AAI27465.1\| Keratin 1 [Rattus norvegicus] | gi\|120474989 (+1) | 65 | 352 | 144 | 4 | 49 | 303 |
| 64 | chromosome segregation 1-like [Rattus norvegicus], gi\|149042854\|gb\|EDL96428.1\| chromosome segregation 1-like (S. cerevisiae) (predicted) [Rattus norvegicus] | gi\|157820325 | 110 | 322 | 109 | 34 | 68 | 254 |
| 65 | neurocan [Rattus norvegicus], gi\|1709256\|sp\|P55067.1\|NCAN_RAT RecName: Full=Neurocan core protein; AltName: Full=Chondroitin sulfate proteoglycan 3; AltName: Full=245 kDa early postnatal core glycoprotein; Contains: RecName: Full=150 kDa adult core glycop | gi\|13928904 (+1) | 136 | 336 | 51 | 34 | 50 | 286 |
| 66 | ATPase, Na+/K+ transporting, alpha 3 polypeptide, isoform CRA_a [Rattus norvegicus] | gi\|149056618 (+3) | 108 | 372 | 4 | 81 | 82 | 290 |
| 67 | dynactin 1, isoform CRA_a [Rattus norvegicus] | gi\|149036514 | 142 | 322 | 36 | 99 | 110 | 212 |
| 68 | cofilin 1 [Rattus norvegicus], gi\|1168996\|sp\|P45592.3\|COF1_RAT RecName: Full=Cofilin-1; AltName: Full=Cofilin, non-muscle isoform, gi\|509201\|emb\|CAA44694.1\| cofilin [Rattus norvegicus], gi\|37589844\|gb\|AAH59143.1\| Cofilin 1, non-muscle [Rattus norvegicus], | gi\|8393101 | 19 | 264 | 177 | 0 | 56 | 208 |
| 69 | adaptor-related protein complex 2, beta 1 subunit [Rattus norvegicus], gi\|71773106\|ref\|NP_001025177.1\| adaptor-related protein complex 2, beta 1 subunit isoform a [Homo sapiens], gi\|73966805\|ref\|XP_853723.1\| PREDICTED: similar to adaptor-related protein c | gi\|18034787 (+1) | 106 | 387 | 32 | 47 | 57 | 330 |
| 70 | tyrosine 3-monooxygenase/tryptophan 5-monooxygenase activation protein, gamma polypeptide [Rattus norvegicus], gi\|21464101\|ref\|NP_036611.2\| tyrosine 3-monooxygenase/tryptophan 5-monooxygenase activation protein, gamma polypeptide [Homo sapiens], gi\|315439 | gi\|9507245 | 28 | 326 | 93 | 22 | 51 | 275 |
| 71 | cytosolic peroxisome proliferator-induced acyl-coA thioesterase [Rattus norvegicus], gi\|1944428\|dbj\|BAA19626.1\| acyl-CoA hydrolase [Rattus norvegicus], gi\|56388796\|gb\|AAH87716.1\| Acyl-CoA thioesterase 7 [Rattus norvegicus] | gi\|1915948 | 38 | 284 | 73 | 53 | 76 | 208 |
| 72 | brain glycogen phosphorylase [Rattus norvegicus], gi\|149031112\|gb\|EDL86139.1\| brain glycogen phosphorylase, isoform CRA_b [Rattus norvegicus] | gi\|158187544 | 97 | 289 | 67 | 70 | 91 | 198 |
| 73 | tubulin, beta 6 [Rattus norvegicus], gi\|67678286\|gb\|AAH97977.1\| Tubulin, beta 6 [Rattus norvegicus], gi\|149064525\|gb\|EDM14728.1\| tubulin, beta 6 [Rattus norvegicus] | gi\|71043680 | 50 | 309 | 69 | 72 | 94 | 215 |
| 74 | ubiquitin specific peptidase 5 [Rattus norvegicus], gi\|149049468\|gb\|EDM01922.1\| ubiquitin specific protease 5 (isopeptidase T) (predicted) [Rattus norvegicus] | gi\|157819971 | 96 | 238 | 117 | 54 | 91 | 147 |
| 75 | guanine nucleotide binding protein, alpha inhibiting 2 [Rattus norvegicus], gi\|121025\|sp\|P04897.3\|GNAI2_RAT RecName: Full=Guanine nucleotide-binding protein G(i), alpha-2 subunit; AltName: Full=Adenylate cyclase-inhibiting G alpha protein, gi\|203166\|gb\|AA | gi\|13591955 | 41 | 327 | 7 | 82 | 84 | 243 |
| 76 | tubulin, beta, 2 [Homo sapiens], gi\|22165384\|ref\|NP_666228.1\| tubulin, beta 2C [Mus musculus], gi\|77736271\|ref\|NP_001029835.1\| tubulin, beta, 2 [Bos taurus], gi\|73967439\|ref\|XP_848461.1\| PREDICTED: similar to tubulin, beta, 2 isoform 1 [Canis familiaris], | gi\|5174735 | 50 | 270 | 91 | 59 | 88 | 182 |
| 77 | rCG42612, isoform CRA_b [Rattus norvegicus] | gi\|149034971 | 55 | 251 | 131 | 32 | 73 | 178 |
| 78 | kinesin family member 5C [Rattus norvegicus], gi\|149047848\|gb\|EDM00464.1\| kinesin family member 5C (predicted) [Rattus norvegicus] | gi\|157819777 | 109 | 306 | 67 | 63 | 84 | 222 |
| 79 | L-lactate dehydrogenase B [Rattus norvegicus], gi\|1170739\|sp\|P42123.2\|LDHB_RAT RecName: Full=L-lactate dehydrogenase B chain; Short=LDH-B; AltName: Full=LDH heart subunit; Short=LDH-H, gi\|473577\|gb\|AAA50439.1\| lactate dehydrogenase-B, gi\|37590241\|gb\|AAH59 | gi\|6981146 | 37 | 269 | 93 | 50 | 79 | 190 |
| 80 | heterogeneous nuclear ribonucleoprotein U [Rattus norvegicus], gi\|47938967\|gb\|AAH72529.1\| Heterogeneous nuclear ribonucleoprotein U [Rattus norvegicus], gi\|149040844\|gb\|EDL94801.1\| rCG20317, isoform CRA_b [Rattus norvegicus] | gi\|148747541 (+1) | 88 | 228 | 53 | 63 | 80 | 148 |
| 81 | ATPase, H+ transporting, lysosomal V1 subunit A [Rattus norvegicus], gi\|109493234\|ref\|XP_001058034.1\| PREDICTED: similar to ATPase, H+ transporting, V1 subunit A, isoform 1 isoform 1 [Rattus norvegicus], gi\|109493236\|ref\|XP_001058095.1\| PREDICTED: similar | gi\|157819953 | 68 | 286 | 20 | 64 | 70 | 216 |
| 82 | peptidylprolyl isomerase A (cyclophilin A) [Rattus norvegicus], gi\|118107\|sp\|P10111.2\|PPIA_RAT RecName: Full=Peptidyl-prolyl cis-trans isomerase A; Short=PPIase A; Short=Rotamase A; AltName: Full=Cyclophilin A; AltName: Full=Cyclosporin A-binding protein; | gi\|8394009 | 18 | 317 | 64 | 11 | 31 | 286 |
| 83 | alanyl-tRNA synthetase [Rattus norvegicus], gi\|149038198\|gb\|EDL92558.1\| alanyl-tRNA synthetase [Rattus norvegicus] | gi\|209862801 | 107 | 256 | 82 | 52 | 78 | 178 |
| 84 | syntaxin binding protein 1 [Rattus norvegicus], gi\|73760415\|ref\|NP_001027392.1\| syntaxin binding protein 1 isoform b [Homo sapiens], gi\|165972305\|ref\|NP_033321.2\| syntaxin binding protein 1 isoform b [Mus musculus], gi\|197097918\|ref\|NP_001126572.1\| syntax | gi\|6981602 | 68 | 297 | 6 | 70 | 72 | 225 |
| 85 | heterogeneous nuclear ribonucleoprotein K isoform a [Homo sapiens], gi\|14165439\|ref\|NP_002131.2\| heterogeneous nuclear ribonucleoprotein K isoform a [Homo sapiens], gi\|197101649\|ref\|NP_001126766.1\| heterogeneous nuclear ribonucleoprotein K [Pongo abelii], | gi\|14165437 | 51 | 244 | 72 | 48 | 71 | 173 |
| 86 | importin 7 [Rattus norvegicus], gi\|149068326\|gb\|EDM17878.1\| importin 7 (predicted), isoform CRA_c [Rattus norvegicus] | gi\|157820315 | 119 | 270 | 91 | 33 | 62 | 208 |
| 87 | exportin 1, CRM1 homolog [Rattus norvegicus], gi\|68052857\|sp\|Q80U96.1\|XPO1_RAT RecName: Full=Exportin-1; Short=Exp1; AltName: Full=Chromosome region maintenance 1 protein homolog, gi\|28971720\|dbj\|BAC65240.1\| nuclear export factor CRM1 [Rattus norvegicus], | gi\|29789299 | 123 | 274 | 58 | 57 | 75 | 199 |
| 88 | rCG55184, isoform CRA_a [Rattus norvegicus] | gi\|149059573 | 171 | 270 | 37 | 68 | 80 | 190 |
| 89 | eukaryotic translation initiation factor 4A isoform 1 [Homo sapiens], gi\|21450625\|ref\|NP_659207.1\| eukaryotic translation initiation factor 4A1 isoform 1 [Mus musculus], gi\|40786436\|ref\|NP_955404.1\| eukaryotic translation initiation factor 4A1 [Rattus nor | gi\|4503529 | 46 | 283 | 78 | 37 | 62 | 221 |
| 90 | L-lactate dehydrogenase A [Rattus norvegicus], gi\|126051\|sp\|P04642.1\|LDHA_RAT RecName: Full=L-lactate dehydrogenase A chain; Short=LDH-A; AltName: Full=LDH muscle subunit; Short=LDH-M, gi\|56544\|emb\|CAA26000.1\| unnamed protein product [Rattus norvegicus], | gi\|8393706 | 36 | 228 | 131 | 10 | 51 | 177 |
| 91 | vacuolar H+ATPase B2 [Rattus norvegicus], gi\|19705578\|ref\|NP_031535.2\| vacuolar H+ATPase B2 [Mus musculus], gi\|51338689\|sp\|P62815.1\|VATB2_RAT RecName: Full=V-type proton ATPase subunit B, brain isoform; Short=V-ATPase subunit B 2; AltName: Full=Vacuolar p | gi\|17105370 | 57 | 295 | 39 | 59 | 71 | 224 |
| 92 | RecName: Full=Dihydropyrimidinase-related protein 4; Short=DRP-4; AltName: Full=Collapsin response mediator protein 3; Short=CRMP-3; AltName: Full=UNC33-like phosphoprotein 4; Short=ULIP4 protein, gi\|1399540\|gb\|AAB03281.1\| rCRMP-3 [Rattus norvegicus] | gi\|3122037 | 61 | 305 | 15 | 56 | 61 | 244 |
| 93 | PREDICTED: similar to Alpha-enolase (2-phospho-D-glycerate hydro-lyase) (Non-neural enolase) (NNE) (Enolase 1) [Rattus norvegicus] | gi\|109468300 (+2) | 54 | 269 | 101 | 6 | 38 | 231 |
| 94 | aminopeptidase puromycin sensitive [Rattus norvegicus] | gi\|149054035 | 93 | 227 | 129 | 16 | 57 | 170 |
| 95 | spectrin beta 2, isoform CRA_a [Rattus norvegicus] | gi\|149044856 | 274 | 227 | 76 | 61 | 85 | 142 |
| 96 | protein arginine methyltransferase 1 [Rattus norvegicus], gi\|2499804\|sp\|Q63009.1\|ANM1_RAT RecName: Full=Protein arginine N-methyltransferase 1, gi\|7141328\|gb\|AAF37293.1\|AF232717_1 protein arginine N-methyltransferase 1 [Mus musculus], gi\|1390025\|gb\|AAC526 | gi\|13242255 (+1) | 41 | 259 | 64 | 35 | 55 | 204 |
| 97 | RecName: Full=AP-2 complex subunit alpha-2; AltName: Full=Adapter-related protein complex 2 alpha-2 subunit; AltName: Full=Adaptor protein complex AP-2 subunit alpha-2; AltName: Full=Alpha2-adaptin; AltName: Full=Plasma membrane adaptor HA2/AP2 adaptin al | gi\|113337 | 104 | 290 | 37 | 25 | 37 | 253 |
| 98 | RecName: Full=Microtubule-associated protein 1B; Short=MAP-1B; AltName: Full=Neuraxin; Contains: RecName: Full=MAP1 light chain LC1 | gi\|19856246 | 270 | 283 | 23 | 62 | 69 | 214 |
| 99 | phosphoglycerate kinase 1 [Rattus norvegicus], gi\|124106305\|sp\|P16617.2\|PGK1_RAT RecName: Full=Phosphoglycerate kinase 1, gi\|38649310\|gb\|AAH63161.1\| Phosphoglycerate kinase 1 [Rattus norvegicus], gi\|56585024\|gb\|AAH87651.1\| Phosphoglycerate kinase 1 [Rattu | gi\|40254752 | 45 | 248 | 91 | 25 | 54 | 194 |
| 100 | hydroxymethylglutaryl-CoA synthase 1 [Rattus norvegicus], gi\|123333\|sp\|P17425.1\|HMCS1_RAT RecName: Full=Hydroxymethylglutaryl-CoA synthase, cytoplasmic; Short=HMG-CoA synthase; AltName: Full=3-hydroxy-3-methylglutaryl coenzyme A synthase, gi\|55947\|emb\|CAA | gi\|8393538 | 57 | 211 | 87 | 59 | 86 | 125 |
| 101 | isocitrate dehydrogenase 1 (NADP+), soluble [Rattus norvegicus], gi\|1170478\|sp\|P41562.1\|IDHC_RAT RecName: Full=Isocitrate dehydrogenase [NADP] cytoplasmic; Short=IDH; AltName: Full=Cytosolic NADP-isocitrate dehydrogenase; AltName: Full=Oxalosuccinate deca | gi\|13928690 | 47 | 246 | 87 | 21 | 48 | 198 |
| 102 | RecName: Full=Dynamin-1-like protein; AltName: Full=Dynamin-like protein, gi\|2425052\|gb\|AAB72197.1\| dynamin-like protein [Rattus norvegicus] | gi\|68566301 | 84 | 269 | 33 | 72 | 82 | 187 |
| 103 | keratin 5 [Rattus norvegicus], gi\|33519156\|gb\|AAQ20893.1\| keratin 5 [Rattus norvegicus] | gi\|50233797 (+1) | 62 | 228 | 81 | 42 | 68 | 160 |
| 104 | cardiac muscle alpha actin 1 proprotein [Homo sapiens], gi\|14192922\|ref\|NP_033738.1\| actin, alpha, cardiac muscle 1 [Mus musculus], gi\|45360837\|ref\|NP_989094.1\| actin, alpha, cardiac muscle 1 [Xenopus (Silurana) tropicalis], gi\|77736117\|ref\|NP_001029757.1 | gi\|4885049 | 42 | 176 | 48 | 5 | 20 | 156 |
| 105 | 5-aminoimidazole-4-carboxamide ribonucleotide formyltransferase/IMP cyclohydrolase [Rattus norvegicus], gi\|122064939\|sp\|O35567.2\|PUR9_RAT RecName: | gi\|48675845 | 64 | 204 | 66 | 62 | 83 | 121 |

| | | | | | | | | |
|---|---|---|---|---|---|---|---|---|
| | Full=Bifunctional purine biosynthesis protein PURH; Includes: RecName: Full=Phosphoribosylaminoimidazolecarb | | | | | | | |
| 106 | rCG38907, isoform CRA_a [Rattus norvegicus] | gi\|149031313 | 343 | 172 | 56 | 82 | 100 | 72 |
| 107 | CAP, adenylate cyclase-associated protein 1 [Rattus norvegicus], gi\|124012084\|sp\|Q08163.3\|CAP1_RAT RecName: Full=Adenylyl cyclase-associated protein 1; Short=CAP 1, gi\|58477725\|gb\|AAH89801.1\| CAP, adenylate cyclase-associated protein 1 (yeast) [Rattus nor | gi\|59709467 | 52 | 265 | 26 | 24 | 32 | 233 |
| 108 | importin 9 [Rattus norvegicus], gi\|149058537\|gb\|EDM09694.1\| importin 9 (predicted) [Rattus norvegicus] | gi\|157821073 | 116 | 201 | 74 | 29 | 52 | 149 |
| 109 | glutamyl-prolyl-tRNA synthetase, isoform CRA_c [Rattus norvegicus] | gi\|149040965 | 164 | 206 | 22 | 57 | 64 | 142 |
| 110 | RecName: Full=E3 ubiquitin-protein ligase UBR4; AltName: Full=N-recognin-4; AltName: Full=Zinc finger UBR1-type protein 1 | gi\|147742911 (+1) | 574 | 184 | 13 | 86 | 90 | 94 |
| 111 | Dync1i2 protein [Rattus norvegicus], gi\|149022201\|gb\|EDL79095.1\| dynein, cytoplasmic, intermediate chain 2, isoform CRA_b [Rattus norvegicus] | gi\|50926121 | 71 | 198 | 9 | 56 | 59 | 139 |
| 112 | acetyl-Coenzyme A acetyltransferase 2 [Rattus norvegicus], gi\|81883683\|sp\|Q5XI22.1\|THIC_RAT RecName: Full=Acetyl-CoA acetyltransferase, cytosolic; AltName: Full=Cytosolic acetoacetyl-CoA thiolase, gi\|54035342\|gb\|AAH83872.1\| Acetyl-Coenzyme A acetyltransfe | gi\|55741502 | 41 | 200 | 74 | 38 | 61 | 139 |
| 113 | filamin, alpha (predicted), isoform CRA_b [Rattus norvegicus] | gi\|149029878 (+1) | 277 | 179 | 63 | 20 | 40 | 139 |
| 114 | nuclear autoantigenic sperm protein [Rattus norvegicus], gi\|81910679\|sp\|Q66HD3.1\|NASP_RAT RecName: Full=Nuclear autoantigenic sperm protein; Short=NASP, gi\|51858884\|gb\|AAH81913.1\| Nuclear autoantigenic sperm protein (histone-binding) [Rattus norvegicus], | gi\|53850614 | 84 | 149 | 112 | 15 | 50 | 99 |
| 115 | eukaryotic translation initiation factor 3, subunit 10 (theta) [Rattus norvegicus] | gi\|149040541 | 163 | 181 | 15 | 57 | 62 | 119 |
| 116 | phosphoribosyl pyrophosphate synthetase 1 [Homo sapiens], gi\|8394053\|ref\|NP_058939.1\| phosphoribosyl pyrophosphate synthetase 1 [Rattus norvegicus], gi\|10946854\|ref\|NP_067438.1\| phosphoribosyl pyrophosphate synthetase 1 [Mus musculus], gi\|114051283\|ref\|NP | gi\|4506127 | 35 | 237 | 8 | 30 | 33 | 204 |
| 117 | tubulin, beta [Mus musculus], gi\|29788768\|ref\|NP_821080.1\| tubulin, beta 2B [Homo sapiens], gi\|110347600\|ref\|NP_001013908.2\| tubulin, beta 2b [Rattus norvegicus], gi\|74003816\|ref\|XP_848443.1\| PREDICTED: similar to tubulin, beta isoform 1 [Canis familiaris | gi\|21746161 | 50 | 195 | 57 | 19 | 37 | 158 |
| 118 | GTP-binding protein alpha o [Rattus norvegicus], gi\|27808491\|sp\|P59215.2\|GNAO_RAT RecName: Full=Guanine nucleotide-binding protein G(o) subunit alpha, gi\|27808492\|sp\|P59216.2\|GNAO_CRILO RecName: Full=Guanine nucleotide-binding protein G(o) subunit alpha, | gi\|8394152 | 40 | 217 | 5 | 56 | 58 | 159 |
| 119 | heat shock 105kDa [Rattus norvegicus], gi\|81890517\|sp\|Q66HA8.1\|HS105_RAT RecName: Full=Heat shock protein 105 kDa; AltName: Full=Heat shock 110 kDa protein, gi\|51859173\|gb\|AAH81945.1\| Heat shock 105kDa/110kDa protein 1 [Rattus norvegicus], gi\|149034799\|gb | gi\|58865372 | 96 | 168 | 69 | 44 | 66 | 102 |
| 120 | coatomer protein complex subunit alpha [Rattus norvegicus], gi\|149040721\|gb\|EDL94678.1\| rCG20221, isoform CRA_b [Rattus norvegicus] | gi\|197384345 | 138 | 174 | 27 | 51 | 60 | 114 |
| 121 | Ncam1 protein [Rattus norvegicus] | gi\|74353679 | 94 | 232 | 21 | 9 | 16 | 216 |
| 122 | proteasome 26S subunit, non-ATPase 14 [Homo sapiens], gi\|71043862\|ref\|NP_001020860.1\| proteasome 26S subunit, non-ATPase 14 [Rattus norvegicus], gi\|115497090\|ref\|NP_001069535.1\| proteasome 26S subunit, non-ATPase 14 [Bos taurus], gi\|145966883\|ref\|NP_06750 | gi\|5031981 | 35 | 172 | 10 | 73 | 76 | 96 |
| 123 | protein phosphatase 2a, catalytic subunit, alpha isoform [Rattus norvegicus], gi\|9506983\|ref\|NP_062284.1\| protein phosphatase 2a, catalytic subunit, alpha isoform [Mus musculus], gi\|54038796\|sp\|P63331.1\|PP2AA_RAT RecName: Full=Serine/threonine-protein pho | gi\|8394018 | 36 | 193 | 54 | 25 | 42 | 151 |
| 124 | rCG27444, isoform CRA_a [Rattus norvegicus] | gi\|149016565 | 79 | 137 | 96 | 29 | 59 | 78 |
| 125 | PREDICTED: similar to p30 DBC protein [Rattus norvegicus], gi\|109502667\|ref\|XP_001069514.1\| PREDICTED: similar to p30 DBC protein [Rattus norvegicus] | gi\|109501857 | 103 | 142 | 47 | 55 | 70 | 72 |
| 126 | proteasome (prosome, macropain) 26S subunit, non-ATPase, 13 [Rattus norvegicus], gi\|229891500\|sp\|B0BN93.1\|PSD13_RAT RecName: Full=26S proteasome non-ATPase regulatory subunit 13; AltName: Full=26S proteasome regulatory subunit S11; AltName: Full=26S prote | gi\|157821581 | 43 | 209 | 8 | 56 | 59 | 150 |
| 127 | PREDICTED: similar to Kinesin-like protein KIF1A (Axonal transporter of synaptic vesicles) [Rattus norvegicus], gi\|149037532\|gb\|EDL91963.1\| kinesin family member 1A [Rattus norvegicus] | gi\|109487519 | 192 | 130 | 101 | 22 | 54 | 76 |
| 128 | inhibitor of kappa light polypeptide enhancer in B-cells, kinase complex-associated protein, isoform CRA_a [Rattus norvegicus] | gi\|149037159 | 149 | 194 | 28 | 37 | 46 | 148 |
| 129 | proteasome (prosome, macropain) 26S subunit, non-ATPase, 3 [Rattus norvegicus], gi\|55250442\|gb\|AAH85881.1\| Proteasome (prosome, macropain) 26S subunit, non-ATPase, 3 [Rattus norvegicus], gi\|149054124\|gb\|EDM05941.1\| proteasome (prosome, macropain) 26S subu | gi\|56605666 | 61 | 177 | 4 | 54 | 55 | 122 |
| 130 | isoleucine tRNA synthetase [Rattus norvegicus], gi\|149045018\|gb\|EDL98104.1\| isoleucine-tRNA synthetase (predicted) [Rattus norvegicus] | gi\|208022685 | 144 | 193 | 0 | 45 | 45 | 148 |
| 131 | Peroxiredoxin 2 [Rattus norvegicus], gi\|149037815\|gb\|EDL92175.1\| rCG51106, isoform CRA_d [Rattus norvegicus] | gi\|34849738 | 22 | 155 | 99 | 2 | 33 | 122 |
| 132 | glutaredoxin 3 [Rattus norvegicus], gi\|60416390\|sp\|Q9JLZ1.2\|GLRX3_RAT RecName: Full=Glutaredoxin-3; AltName: Full=Thioredoxin-like protein 2; AltName: Full=PKC-interacting cousin of thioredoxin; AltName: Full=PKC-theta-interacting protein; Short=PKCq-inte | gi\|78187979 | 38 | 135 | 66 | 49 | 70 | 65 |
| 133 | protease (prosome, macropain) 26S subunit, ATPase 1 [Mus musculus], gi\|16923972\|ref\|NP_476464.1\| proteasome (prosome, macropain) 26S subunit, ATPase, 1 [Rattus norvegicus], gi\|24430151\|ref\|NP_002793.2\| proteasome 26S ATPase subunit 1 [Homo sapiens], gi\|11 | gi\|6679501 | 49 | 169 | 5 | 83 | 85 | 84 |
| 134 | proteasome (prosome, macropain) 26S subunit, ATPase 3 [Mus musculus], gi\|13543237\|gb\|AAH05783.1\| Proteasome (prosome, macropain) 26S subunit, ATPase 3 [Mus musculus], gi\|38304011\|gb\|AAH62019.1\| Proteasome (prosome, macropain) 26S subunit, ATPase 3 [Rattus | gi\|228008337 (+1) | 50 | 191 | 3 | 71 | 72 | 119 |
| 135 | ubiquitin-like modifier activating enzyme 2 [Rattus norvegicus], gi\|149056217\|gb\|EDM07648.1\| rCG53609 [Rattus norvegicus], gi\|171847094\|gb\|AAI61985.1\| Uba2 protein [Rattus norvegicus] | gi\|197384571 | 71 | 128 | 73 | 30 | 53 | 75 |
| 136 | ADP-ribosylation factor 5 [Homo sapiens], gi\|6680722\|ref\|NP_031506.1\| ADP-ribosylation factor 5 [Mus musculus], gi\|13162339\|ref\|NP_077063.1\| ADP-ribosylation factor 5 [Rattus norvegicus], gi\|139948288\|ref\|NP_001077140.1\| ADP-ribosylation factor 5 [Bos tau | gi\|4502209 | 21 | 168 | 83 | 0 | 26 | 142 |
| 137 | transferrin [Rattus norvegicus], gi\|122066515\|sp\|P12346.3\|TRFE_RAT RecName: Full=Serotransferrin; Short=Transferrin; AltName: Full=Siderophilin; AltName: Full=Beta-1 metal-binding globulin; AltName: Full=Liver regeneration-related protein LRRG03; Flags: P | gi\|61556986 | 76 | 40 | 215 | 0 | 68 | 0 |
| 138 | myosin Va [Rattus norvegicus], gi\|13431673\|sp\|Q9QYF3.1\|MYO5A_RAT RecName: Full=Myosin-Va; AltName: Full=Dilute myosin heavy chain, non-muscle, gi\|6577099\|dbj\|BAA88350.1\| myosin-Va [Rattus norvegicus] | gi\|11559935 | 212 | 163 | 22 | 58 | 65 | 98 |
| 139 | PREDICTED: similar to cytoskeleton associated protein 5 isoform 3 [Rattus norvegicus] | gi\|109470239 (+2) | 227 | 173 | 24 | 32 | 40 | 133 |
| 140 | tyrosine 3-monooxygenase/tryptophan 5-monooxygenase activation protein, eta polypeptide [Mus musculus], gi\|6981710\|ref\|NP_037184.1\| tyrosine 3-monooxygenase/tryptophan 5-monooxygenase activation protein, eta polypeptide [Rattus norvegicus], gi\|194043292\|r | gi\|6756037 | 28 | 178 | 42 | 31 | 44 | 134 |
| 141 | protein phosphatase 1, catalytic subunit, alpha isoform 1 [Homo sapiens], gi\|13928710\|ref\|NP_113715.1\| protein phosphatase 1, catalytic subunit, alpha [Rattus norvegicus], gi\|78369446\|ref\|NP_001030393.1\| protein phosphatase 1, catalytic subunit, alpha [Bo | gi\|4506003 | 38 | 167 | 43 | 43 | 57 | 110 |
| 142 | ras-related nuclear protein [Homo sapiens], gi\|6678365\|ref\|NP_033417.1\| RAN, member RAS oncogene family [Mus musculus], gi\|16758182\|ref\|NP_445891.1\| RAN, member RAS oncogene family [Rattus norvegicus], gi\|50979248\|ref\|NP_001003375.1\| ras-related nuclear p | gi\|5453555 | 24 | 162 | 50 | 23 | 39 | 123 |
| 143 | vesicle amine transport protein 1 homolog (T californica) [Rattus norvegicus], gi\|75516467\|gb\|AAI01883.1\| Vesicle amine transport protein 1 homolog (T californica) [Rattus norvegicus], gi\|149054315\|gb\|EDM06132.1\| vesicle amine transport protein 1 homolog | gi\|76096306 | 43 | 225 | 17 | 7 | 12 | 213 |
| 144 | proteasome (prosome, macropain) 26S subunit, non-ATPase, 7 [Rattus norvegicus], gi\|149038124\|gb\|EDL92484.1\| proteasome (prosome, macropain) 26S subunit, non-ATPase, 7 (predicted) [Rattus norvegicus] | gi\|157818107 | 36 | 176 | 2 | 67 | 68 | 108 |
| 145 | kinesin light chain 1 isoform A [Rattus norvegicus] | gi\|126517461 (+2) | 62 | 169 | 32 | 26 | 36 | 133 |
| 146 | Dbn1 protein [Rattus norvegicus], gi\|149039865\|gb\|EDL93981.1\| drebrin 1, isoform CRA_b [Rattus norvegicus] | gi\|146218400 (+1) | 72 | 131 | 95 | 13 | 43 | 88 |
| 147 | eukaryotic translation elongation factor 1 gamma [Rattus norvegicus], gi\|55583762\|sp\|Q68FR6.3\|EF1G_RAT RecName: Full=Elongation factor 1-gamma; Short=EF-1-gamma; AltName: Full=eEF-1B gamma, gi\|51261278\|gb\|AAH79398.1\| Eukaryotic translation elongation fact | gi\|51948418 | 50 | 186 | 7 | 27 | 29 | 157 |
| 148 | leucyl-tRNA synthetase [Rattus norvegicus], gi\|56388592\|gb\|AAH87655.1\| Leucyl-tRNA synthetase [Rattus norvegicus] | gi\|57526927 | 134 | 156 | 9 | 57 | 60 | 96 |
| 149 | alcohol dehydrogenase 5 [Rattus norvegicus], gi\|134047695\|sp\|P12711.2\|ADHX_RAT RecName: Full=Alcohol dehydrogenase class-3; AltName: Full=Alcohol dehydrogenase class-III; AltName: Full=Alcohol dehydrogenase 5; AltName: Full=Alcohol dehydrogenase 2; AltNam | gi\|186972146 (+1) | 40 | 130 | 29 | 40 | 49 | 81 |
| 150 | RAB11B, member RAS oncogene family [Rattus norvegicus], gi\|78369332\|ref\|NP_001030468.1\| RAB11B, member RAS oncogene family [Bos taurus], gi\|190358517\|ref\|NP_004209.2\| RAB11B, member RAS oncogene family [Homo sapiens], gi\|226529709\|ref\|NP_001142168.1\| hypo | gi\|14249144 | 24 | 194 | 38 | 9 | 21 | 173 |
| 151 | rCG48597, isoform CRA_a [Rattus norvegicus] | gi\|149061779 (+1) | 43 | 172 | 44 | 23 | 37 | 135 |
| 152 | myosin, heavy polypeptide 9, non-muscle [Rattus norvegicus] | gi\|149066032 | 226 | 140 | 58 | 38 | 56 | 84 |
| 153 | valyl-tRNA synthetase [Rattus norvegicus], gi\|73920806\|sp\|Q04462.2\|SYVC_RAT RecName: Full=Valyl-tRNA synthetase; AltName: Full=Valine--tRNA ligase; Short=ValRS, gi\|46237603\|emb\|CAE83981.1\| valyl-tRNA synthetase 2 [Rattus norvegicus], gi\|149028031\|gb\|EDL83 | gi\|158186770 | 140 | 196 | 8 | 29 | 32 | 164 |
| 154 | adaptor-related protein complex 3, delta 1 subunit [Rattus norvegicus], gi\|149034506\|gb\|EDL89243.1\| adaptor-related protein complex 3, delta 1 subunit, isoform CRA_b [Rattus norvegicus], gi\|197246509\|gb\|AAI69097.1\| Ap3d1 protein [Rattus norvegicus] | gi\|198278523 | 136 | 153 | 35 | 47 | 58 | 95 |
| 155 | guanine nucleotide-binding protein, beta-2 subunit [Mus musculus], gi\|20357529\|ref\|NP_005264.2\| guanine nucleotide-binding protein, beta-2 subunit [Homo sapiens], gi\|29789261\|ref\|NP_112299.1\| guanine nucleotide-binding protein, beta 2 [Rattus norvegicus], | gi\|13937391 | 37 | 140 | 21 | 48 | 55 | 85 |
| 156 | WD repeat domain 1 [Rattus norvegicus], gi\|81910041\|sp\|Q5RKI0.3\|WDR1_RAT RecName: Full=WD repeat-containing protein 1, gi\|55715677\|gb\|AAH85864.1\| WD repeat domain 1 [Rattus norvegicus], gi\|62721147\|gb\|AAX94056.1\| WD repeat protein 1 [Rattus norvegicus] | gi\|62078997 | 66 | 129 | 51 | 29 | 45 | 84 |
| 157 | alpha actinin 4 [Rattus norvegicus], gi\|182705246\|sp\|Q9QXQ0.2\|ACTN4_RAT RecName: Full=Alpha-actinin-4; AltName: Full=Non-muscle alpha-actinin 4; AltName: Full=F-actin cross-linking protein, gi\|38197444\|gb\|AAH61788.1\| Actinin alpha 4 [Rattus norvegicus], g | gi\|77539778 | 105 | 122 | 93 | 14 | 43 | 79 |
| 158 | malate dehydrogenase 1, NAD (soluble) [Rattus norvegicus], gi\|81861572\|sp\|O88989.3\|MDHC_RAT RecName: Full=Malate dehydrogenase, cytoplasmic; AltName: Full=Cytosolic malate dehydrogenase, gi\|3747085\|gb\|AAC64180.1\| cytosolic malate dehydrogenase [Rattus nor | gi\|15100179 | 36 | 117 | 82 | 4 | 30 | 87 |
| 159 | carbamyl phosphatate synthetase 2 (mapped), isoform CRA_e [Rattus norvegicus] | gi\|149050766 | 251 | 162 | 8 | 49 | 52 | 110 |
| 160 | platelet-activating factor acetylhydrolase, isoform Ib, alpha subunit [Mus musculus], gi\|13929078\|ref\|NP_113951.1\| platelet-activating factor acetylhydrolase beta subunit [Rattus norvegicus], gi\|167234378\|ref\|NP_001107812.1\| platelet-activating factor ace | gi\|7305363 | 47 | 163 | 36 | 21 | 32 | 131 |

| | | | | | | | | |
|---|---|---|---|---|---|---|---|---|
| 161 | PREDICTED: similar to Putative pre-mRNA-splicing factor ATP-dependent RNA helicase DHX15 (DEAH box protein 15) [Rattus norvegicus], gi\|109500663\|ref\|XP_001054651.1\| PREDICTED: similar to Putative pre-mRNA-splicing factor ATP-dependent RNA helicase DHX15 ( | gi\|34878065 | 91 | 136 | 42 | 43 | 56 | 80 |
| 162 | proteasome, 26S, non-ATPase regulatory subunit 6 [Rattus norvegicus], gi\|37748489\|gb\|AAH59159.1\| Proteasome (prosome, macropain) 26S subunit, non-ATPase, 6 [Rattus norvegicus], gi\|149040032\|gb\|EDL94116.1\| proteasome (prosome, macropain) 26S subunit, non-A | gi\|38454206 | 46 | 155 | 6 | 59 | 61 | 94 |
| 163 | tripeptidyl peptidase II [Rattus norvegicus], gi\|9979103\|sp\|Q64560.3\|TPP2_RAT RecName: Full=Tripeptidyl-peptidase 2; AltName: Full=Tripeptidyl-peptidase II; Short=TPP-II; AltName: Full=Tripeptidyl aminopeptidase, gi\|1245343\|gb\|AAA93458.1\| tripeptidylpepti | gi\|13592121 | 138 | 143 | 0 | 34 | 34 | 109 |
| 164 | guanine monophosphate synthetase [Rattus norvegicus], gi\|81908630\|sp\|Q4V7C6.1\|GUAA_RAT RecName: Full=GMP synthase [glutamine-hydrolyzing]; AltName: Full=Glutamine amidotransferase; AltName: Full=GMP synthetase, gi\|66910916\|gb\|AAH98016.1\| Guanine monphosph | gi\|67078508 | 77 | 122 | 56 | 36 | 54 | 68 |
| 165 | neural precursor cell expressed, developmentally down-regulated gene 4 [Rattus norvegicus], gi\|149028788\|gb\|EDL84129.1\| neural precursor cell expressed, developmentally down-regulated gene 4A, isoform CRA_d [Rattus norvegicus] | gi\|158186672 | 103 | 92 | 64 | 32 | 52 | 40 |
| 166 | inducible carbonyl reductase [Rattus norvegicus] | gi\|1906812 | 31 | 147 | 58 | 15 | 33 | 114 |
| 167 | ubiquitin specific peptidase 7 [Rattus norvegicus], gi\|81908181\|sp\|Q4VSI4.1\|UBP7_RAT RecName: Full=Ubiquitin carboxyl-terminal hydrolase 7; AltName: Full=Ubiquitin thioesterase 7; AltName: Full=Ubiquitin-specific-processing protease 7; AltName: Full=Deubi | gi\|67078438 | 128 | 122 | 49 | 23 | 38 | 84 |
| 168 | anaphase promoting complex subunit 1 [Rattus norvegicus], gi\|149023243\|gb\|EDL80137.1\| anaphase promoting complex subunit 1 (predicted) [Rattus norvegicus] | gi\|157820993 | 216 | 138 | 18 | 39 | 45 | 93 |
| 169 | PREDICTED: similar to EPM2A (laforin) interacting protein 1 [Rattus norvegicus] | gi\|109485509 (+1) | 70 | 129 | 54 | 37 | 54 | 75 |
| 170 | adaptor protein complex AP-1, beta 1 subunit, isoform CRA_a [Rattus norvegicus] | gi\|149047589 (+1) | 105 | 175 | 5 | 28 | 30 | 145 |
| 171 | proteasome 26S non-ATPase subunit 12 [Rattus norvegicus], gi\|53733547\|gb\|AAH83758.1\| Proteasome (prosome, macropain) 26S subunit, non-ATPase, 12 [Rattus norvegicus] | gi\|54400716 | 53 | 177 | 3 | 37 | 38 | 139 |
| 172 | platelet-activating factor acetylhydrolase, isoform Ib, gamma subunit [Rattus norvegicus], gi\|6093641\|sp\|O35263.1\|PA1B3_RAT RecName: Full=Platelet-activating factor acetylhydrolase IB subunit gamma; AltName: Full=PAF acetylhydrolase 29 kDa subunit; Short= | gi\|16758470 | 26 | 139 | 41 | 3 | 16 | 123 |
| 173 | RecName: Full=Nucleolin; AltName: Full=Protein C23, gi\|205792\|gb\|AAA41732.1\| nucleolin | gi\|128844 (+2) | 77 | 163 | 24 | 24 | 32 | 131 |
| 174 | aldo-keto reductase family 1, member A1 (aldehyde reductase) [Rattus norvegicus], gi\|1703237\|sp\|P51635.2\|AK1A1_RAT RecName: Full=Alcohol dehydrogenase [NADP+]; AltName: Full=Aldehyde reductase; AltName: Full=Aldo-keto reductase family 1 member A1; AltName | gi\|13591894 | 37 | 129 | 44 | 23 | 37 | 92 |
| 175 | tyrosine 3-monooxgenase/tryptophan 5-monooxgenase activation protein, beta polypeptide [Rattus norvegicus], gi\|1345591\|sp\|P35213.3\|1433B_RAT RecName: Full=14-3-3 protein beta/alpha; AltName: Full=Protein kinase C inhibitor protein 1; Short=KCIP-1; AltName | gi\|9507243 | 28 | 150 | 36 | 19 | 30 | 120 |
| 176 | talin [Rattus norvegicus], gi\|109474844\|ref\|XP_001053379.1\| PREDICTED: similar to Talin-1 [Rattus norvegicus], gi\|109476401\|ref\|XP_001069865.1\| PREDICTED: similar to Talin-1 [Rattus norvegicus], gi\|149045752\|gb\|EDL98752.1\| rCG55135, isoform CRA_b [Rattus | gi\|189181726 | 270 | 95 | 78 | 28 | 53 | 42 |
| 177 | PREDICTED: similar to Proteasome-associated protein ECM29 homolog (Ecm29) [Rattus norvegicus] | gi\|109474974 (+1) | 216 | 158 | 10 | 24 | 27 | 131 |
| 178 | PREDICTED: similar to Ubiquitin conjugation factor E4 B (Ubiquitin-fusion degradation protein 2) (Ufd2a) [Rattus norvegicus] | gi\|62650035 | 133 | 100 | 60 | 15 | 34 | 66 |
| 179 | dimethylarginine dimethylaminohydrolase 2 [Rattus norvegicus], gi\|81911114\|sp\|Q6MG60.1\|DDAH2_RAT RecName: Full=N(G),N(G)-dimethylarginine dimethylaminohydrolase 2; Short=Dimethylarginine dimethylaminohydrolase 2; Short=DDAH-2; AltName: Full=DDAHII; AltNam | gi\|47087079 | 30 | 132 | 56 | 21 | 39 | 93 |
| 180 | rCG55067 [Rattus norvegicus] | gi\|149045627 | 98 | 133 | 64 | 4 | 24 | 109 |
| 181 | proteasome (prosome, macropain) 26S subunit, ATPase, 6 [Rattus norvegicus], gi\|149033503\|gb\|EDL88301.1\| rCG61291 [Rattus norvegicus] | gi\|213972598 (+1) | 46 | 140 | 11 | 55 | 58 | 82 |
| 182 | reticulon 1 [Rattus norvegicus], gi\|12643485\|sp\|Q64548.1\|RTN1_RAT RecName: Full=Reticulon-1; AltName: Full=Neuroendocrine-specific protein; AltName: Full=S-rex, gi\|1143719\|gb\|AAC53046.1\| rS-Rex-b | gi\|16758732 | 83 | 175 | 15 | 4 | 9 | 166 |
| 183 | fructose-bisphosphate aldolase A [Rattus norvegicus], gi\|113609\|sp\|P05065.2\|ALDOA_RAT RecName: Full=Fructose-bisphosphate aldolase A; AltName: Full=Muscle-type aldolase, gi\|202835\|gb\|AAA40714.1\| aldolase A (EC 4.1.2.13), gi\|1619605\|emb\|CAA27815.1\| aldolas | gi\|6978487 | 39 | 162 | 32 | 0 | 10 | 152 |
| 184 | acetyl-coenzyme A carboxylase alpha [Rattus norvegicus], gi\|116670\|sp\|P11497.1\|ACACA_RAT RecName: Full=Acetyl-CoA carboxylase 1; AltName: Full=ACC-alpha; Includes: RecName: Full=Biotin carboxylase, gi\|202645\|gb\|AAA40653.1\| acetyl-coenzyme A carboxylase (E | gi\|11559962 | 265 | 118 | 34 | 34 | 45 | 73 |
| 185 | proteasome 26S  ATPase subunit 4 [Rattus norvegicus], gi\|124248577\|ref\|NP_036004.2\| proteasome 26S  ATPase subunit 4 [Mus musculus], gi\|2492517\|sp\|Q63570.1\|PRS6B_RAT RecName: Full=26S protease regulatory subunit 6B; AltName: Full=Proteasome 26S subunit AT | gi\|25742677 | 47 | 141 | 4 | 37 | 38 | 103 |
| 186 | staphylococcal nuclease domain containing 1 [Rattus norvegicus], gi\|60415342\|sp\|Q66X93.1\|SND1_RAT RecName: Full=Staphylococcal nuclease domain-containing protein 1; AltName: Full=p100 co-activator; AltName: Full=100 kDa coactivator; AltName: Full=p105 coa | gi\|77404395 | 102 | 150 | 4 | 46 | 47 | 103 |
| 187 | PREDICTED: similar to glycyl-tRNA synthetase [Rattus norvegicus] | gi\|109472087 (+2) | 88 | 133 | 43 | 0 | 14 | 119 |
| 188 | protease (prosome, macropain) 26S subunit, ATPase 5 [Mus musculus], gi\|13592141\|ref\|NP_112411.1\| for proteasomal ATPase (SUG1) [Rattus norvegicus], gi\|24497435\|ref\|NP_002796.4\| proteasome 26S ATPase subunit 5 [Homo sapiens], gi\|27806101\|ref\|NP_776866.1\| p | gi\|7110703 | 46 | 143 | 4 | 37 | 38 | 105 |
| 189 | s-Afadin [Rattus norvegicus] | gi\|2555013 | 189 | 123 | 32 | 32 | 42 | 81 |
| 190 | Chain A, Rat Liver S-Adenosylhomocystein Hydrolase, gi\|4139572\|pdb\|1B3R\|B Chain B, Rat Liver S-Adenosylhomocystein Hydrolase, gi\|4139573\|pdb\|1B3R\|C Chain C, Rat Liver S-Adenosylhomocystein Hydrolase, gi\|4139574\|pdb\|1B3R\|D Chain D, Rat Liver S-Adenosylhomo | gi\|4139571 (+1) | 47 | 118 | 36 | 17 | 28 | 90 |
| 191 | ataxin 10 [Rattus norvegicus], gi\|18202870\|sp\|Q9ER24.1\|ATX10_RAT RecName: Full=Ataxin-10; AltName: Full=Spinocerebellar ataxia type 10 protein homolog; AltName: Full=Neuronal beta-catenin-like protein, gi\|11182352\|emb\|CAC16214.1\| neuronal beta-catenin lik | gi\|25742674 | 54 | 119 | 48 | 7 | 22 | 97 |
| 192 | beta actin [Homo sapiens], gi\|6671509\|ref\|NP_031419.1\| actin, beta [Mus musculus], gi\|13592133\|ref\|NP_112406.1\| actin, beta [Rattus norvegicus], gi\|45382927\|ref\|NP_990849.1\| beta actin [Gallus gallus], gi\|57619329\|ref\|NP_001009784.1\| beta actin [Ovis arie | gi\|4501885 | 42 | 137 | 32 | 34 | 44 | 93 |
| 193 | synaptosomal-associated protein, 91kDa homolog (mouse), isoform CRA_a [Rattus norvegicus], gi\|149018977\|gb\|EDL77618.1\| synaptosomal-associated protein, 91kDa homolog (mouse), isoform CRA_a [Rattus norvegicus], gi\|149018979\|gb\|EDL77620.1\| synaptosomal-asso | gi\|149018973 (+3) | 89 | 135 | 34 | 12 | 23 | 112 |
| 194 | singar2 [Rattus norvegicus] | gi\|145967404 | 55 | 117 | 38 | 15 | 27 | 90 |
| 195 | SEC31-like 1 (S. cerevisiae), isoform CRA_a [Rattus norvegicus] | gi\|149046798 (+2) | 135 | 123 | 23 | 26 | 33 | 90 |
| 196 | proteasome (prosome, macropain) 26S subunit, non-ATPase, 11 (predicted), isoform CRA_b [Rattus norvegicus] | gi\|149053606 | 56 | 141 | 3 | 22 | 23 | 118 |
| 197 | RAB14, member RAS oncogene family [Mus musculus], gi\|19923483\|ref\|NP_057406.2\| GTPase Rab14 [Homo sapiens], gi\|55926076\|ref\|NP_001007505.1\| rab14, member ras oncogene family [Xenopus (Silurana) tropicalis], gi\|61098298\|ref\|NP_001012814.1\| RAB14, member RA | gi\|18390323 | 24 | 148 | 29 | 7 | 16 | 132 |
| 198 | aspartyl-tRNA synthetase [Rattus norvegicus], gi\|135099\|sp\|P15178.1\|SYDC_RAT RecName: Full=Aspartyl-tRNA synthetase, cytoplasmic; AltName: Full=Aspartate--tRNA ligase; Short=AspRS, gi\|203066\|gb\|AAA40789.1\| aspartyl-tRNA synthetase, gi\|1773257\|gb\|AAC52981. | gi\|16758642 | 57 | 139 | 7 | 20 | 22 | 117 |
| 199 | arginyl-tRNA synthetase (predicted), isoform CRA_a [Rattus norvegicus] | gi\|149052278 | 67 | 123 | 6 | 42 | 44 | 79 |
| 200 | phospholipase C, gamma 1, isoform CRA_a [Rattus norvegicus] | gi\|149043040 (+2) | 148 | 112 | 40 | 27 | 40 | 72 |
| 201 | RecName: Full=Apolipoprotein B-100; Short=Apo B-100; Contains: RecName: Full=Apolipoprotein B-48; Short=Apo B-48; Flags: Precursor, gi\|32492562\|gb\|AAP85369.1\| Aa1064 [Rattus norvegicus], gi\|32527695\|gb\|AAP86249.1\| Ac1-060 [Rattus norvegicus] | gi\|81894378 | 536 | 121 | 51 | 1 | 17 | 104 |
| 202 | phosphofructokinase, liver [Rattus norvegicus], gi\|400095\|sp\|P30835.3\|K6PL_RAT RecName: Full=6-phosphofructokinase, liver type; AltName: Full=Phosphofructokinase 1; AltName: Full=Phosphohexokinase; AltName: Full=Phosphofructo-1-kinase isozyme B; Short=PFK | gi\|6981352 | 85 | 127 | 16 | 26 | 31 | 96 |
| 203 | dynein cytoplasmic 1 light intermediate chain 1 [Rattus norvegicus] | gi\|149018342 | 57 | 136 | 2 | 35 | 36 | 100 |
| 204 | tripartite motif-containing 2 [Rattus norvegicus], gi\|149048249\|gb\|EDM00825.1\| tripartite motif protein 2 [Rattus norvegicus] | gi\|157818847 | 83 | 122 | 18 | 25 | 31 | 91 |
| 205 | bisphosphoglycerate mutase 1 [Mus musculus], gi\|226496801\|ref\|NP_001141260.1\| hypothetical protein LOC100273347 [Zea mays], gi\|20178035\|sp\|Q9DBJ1.3\|PGAM1_MOUSE RecName: Full=Phosphoglycerate mutase 1; AltName: Full=Phosphoglycerate mutase isozyme B; Short | gi\|114326546 | 29 | 106 | 41 | 16 | 29 | 77 |
| 206 | suppressor of Ty 16 homolog [Rattus norvegicus], gi\|149033673\|gb\|EDL88471.1\| suppressor of Ty 16 homolog (S. cerevisiae) (predicted) [Rattus norvegicus] | gi\|157821845 | 120 | 84 | 51 | 17 | 33 | 51 |
| 207 | proteasome (prosome, macropain) 28 subunit, beta, isoform CRA_a [Rattus norvegicus] | gi\|149063974 (+1) | 26 | 157 | 14 | 5 | 9 | 148 |
| 208 | preproapolipoprotein A-I [Rattus norvegicus] | gi\|55747 (+1) | 30 | 95 | 76 | 0 | 24 | 71 |
| 209 | actin related protein 2/3 complex, subunit 2 [Rattus norvegicus], gi\|149016091\|gb\|EDL75337.1\| actin related protein 2/3 complex, subunit 2 (predicted), isoform CRA_a [Rattus norvegicus] | gi\|157817229 | 34 | 135 | 11 | 5 | 8 | 127 |
| 210 | stress-induced phosphoprotein 1 [Rattus norvegicus], gi\|54036435\|sp\|O35814.1\|STIP1_RAT RecName: Full=Stress-induced-phosphoprotein 1; Short=STI1; AltName: Full=Hsc70/Hsp90-organizing protein; Short=Hop, gi\|2511703\|emb\|CAA75351.1\| p60 protein [Rattus norve | gi\|20302113 | 63 | 88 | 59 | 0 | 19 | 69 |
| 211 | ubiquilin 2 [Rattus norvegicus], gi\|149031365\|gb\|EDL86363.1\| ubiquilin 2 (predicted) [Rattus norvegicus] | gi\|157818175 | 67 | 121 | 41 | 6 | 19 | 102 |
| 212 | microtubule-associated protein, RP/EB family, member 1 [Rattus norvegicus], gi\|60389848\|sp\|Q66HR2.3\|MARE1_RAT RecName: Full=Microtubule-associated protein RP/EB family member 1; AltName: Full=APC-binding protein EB1; AltName: Full=End-binding protein 1; S | gi\|78097100 | 30 | 107 | 41 | 4 | 17 | 90 |
| 213 | guanine nucleotide binding protein, alpha q polypeptide [Mus musculus], gi\|74184519\|dbj\|BAE27882.1\| unnamed protein product [Mus musculus], gi\|77386135\|gb\|ABA77547.1\| guanine nucleotide binding protein q polypeptide [Cricetulus griseus], gi\|117616372\|gb\|A | gi\|84662745 | 42 | 122 | 5 | 30 | 32 | 90 |
| 214 | rCG59785, isoform CRA_a [Rattus norvegicus] | gi\|149066599 | 103 | 118 | 0 | 36 | 36 | 82 |
| 215 | kinesin family member 21B [Rattus norvegicus], gi\|149058510\|gb\|EDM09667.1\| kinesin family member 21B (predicted) [Rattus norvegicus] | gi\|157821213 | 183 | 105 | 28 | 30 | 39 | 66 |
| 216 | rCG48740, isoform CRA_c [Rattus norvegicus] | gi\|149066999 (+2) | 45 | 104 | 22 | 25 | 32 | 72 |
| 217 | RAB7A, member RAS oncogene family [Rattus norvegicus], gi\|1710001\|sp\|P09527.2\|RAB7A_RAT RecName: Full=Ras-related protein Rab-7a; AltName: Full=Ras-related protein p23; AltName: Full=Ras-related protein BRL-RAS, gi\|51247924\|pdb\|1VG0\|B Chain B, The Crystal | gi\|13027392 | 24 | 109 | 32 | 7 | 17 | 92 |

| | | | | | | | | |
|---|---|---|---|---|---|---|---|---|
| 218 | prolyl endopeptidase [Rattus norvegicus], gi\|81861326\|sp\|O70196.1\|PPCE_RAT RecName: Full=Prolyl endopeptidase; Short=PE; AltName: Full=Post-proline cleaving enzyme; AltName: Full=rPop, gi\|3043760\|dbj\|BAA25544.1\| rPOP [Rattus norvegicus] | gi\|13786144 (+1) | 81 | 112 | 18 | 32 | 38 | 74 |
| 219 | rCG32615, isoform CRA_a [Rattus norvegicus], gi\|165970759\|gb\|AAI58857.1\| Rab5c protein [Rattus norvegicus] | gi\|149054244 | 23 | 129 | 32 | 6 | 16 | 113 |
| 220 | X-prolyl aminopeptidase (aminopeptidase P) 1, soluble, isoform CRA_a [Rattus norvegicus] | gi\|149040371 | 75 | 106 | 23 | 17 | 24 | 82 |
| 221 | otubain 1 [Mus musculus], gi\|157821731\|ref\|NP_001099802.1\| OTU domain, ubiquitin aldehyde binding 1 [Rattus norvegicus], gi\|149270309\|ref\|XP_001476636.1\| PREDICTED: similar to OTU domain, ubiquitin aldehyde binding 1 [Mus musculus], gi\|44888264\|sp\|Q7TQI3. | gi\|19527388 | 31 | 89 | 44 | 15 | 29 | 60 |
| 222 | PREDICTED: similar to ubiquitin-conjugating enzyme E2O [Rattus norvegicus] | gi\|109489413 | 143 | 79 | 70 | 9 | 31 | 48 |
| 223 | Dync1li2 protein [Rattus norvegicus] | gi\|60422784 | 54 | 107 | 3 | 32 | 33 | 74 |
| 224 | PREDICTED: similar to eukaryotic translation initiation factor 4, gamma 1 isoform a [Rattus norvegicus], gi\|109494661\|ref\|XP_001060756.1\| PREDICTED: similar to eukaryotic translation initiation factor 4, gamma 1 isoform a [Rattus norvegicus] | gi\|62658155 | 176 | 103 | 37 | 27 | 39 | 64 |
| 225 | Ca2+-dependent secretion activator, isoform CRA_a [Rattus norvegicus] | gi\|149040049 | 153 | 81 | 62 | 16 | 36 | 45 |
| 226 | maternal embryonic message 3 [Rattus norvegicus], gi\|149032606\|gb\|EDL87476.1\| vacuolar protein sorting 35 (mapped), isoform CRA_b [Rattus norvegicus] | gi\|205360969 | 92 | 134 | 6 | 28 | 30 | 104 |
| 227 | coxsackie-adenovirus-receptor homolog [Rattus norvegicus] | gi\|6013135 (+1) | 39 | 117 | 2 | 38 | 39 | 78 |
| 228 | COP9 signalosome subunit 4 [Rattus norvegicus], gi\|56405004\|sp\|Q68FS2.1\|CSN4_RAT RecName: Full=COP9 signalosome complex subunit 4; Short=Signalosome subunit 4; Short=SGN4; AltName: Full=JAB1-containing signalosome subunit 4, gi\|51259458\|gb\|AAH79384.1\| COP | gi\|51948518 | 46 | 123 | 10 | 16 | 19 | 104 |
| 229 | dipeptidyl peptidase III [Rattus norvegicus], gi\|13626316\|sp\|O55096.2\|DPP3_RAT RecName: Full=Dipeptidyl-peptidase 3; AltName: Full=Dipeptidyl-peptidase III; Short=DPP III; AltName: Full=Dipeptidyl aminopeptidase III; AltName: Full=Dipeptidyl arylamidase I | gi\|16758578 | 83 | 103 | 57 | 0 | 18 | 85 |
| 230 | damage-specific DNA binding protein 1 [Rattus norvegicus] | gi\|149062405 | 127 | 105 | 46 | 5 | 19 | 86 |
| 231 | radixin [Rattus norvegicus] | gi\|40804379 (+1) | 69 | 110 | 26 | 0 | 8 | 102 |
| 232 | capping protein (actin filament) muscle Z-line, alpha 2 [Mus musculus], gi\|57163991\|ref\|NP_001009180.1\| capping protein (actin filament) muscle Z-line, alpha 2 [Rattus norvegicus], gi\|1345696\|sp\|P47754.3\|CAZA2_MOUSE RecName: Full=F-actin-capping protein s | gi\|6671672 | 33 | 95 | 50 | 8 | 24 | 71 |
| 233 | acidic (leucine-rich) nuclear phosphoprotein 32 family, member E, isoform CRA_b [Rattus norvegicus] | gi\|149030622 (+3) | 29 | 113 | 33 | 25 | 35 | 78 |
| 234 | adaptor-related protein complex 3, beta 2 subunit (predicted), isoform CRA_b [Rattus norvegicus] | gi\|149057364 | 117 | 72 | 34 | 24 | 35 | 37 |
| 235 | coatomer protein complex, subunit beta 2 (beta prime) [Rattus norvegicus] | gi\|149018820 (+1) | 102 | 120 | 12 | 18 | 22 | 98 |
| 236 | protein arginine methyltransferase 5 [Rattus norvegicus], gi\|149063904\|gb\|EDM14174.1\| protein arginine N-methyltransferase 5 (predicted), isoform CRA_a [Rattus norvegicus] | gi\|184160976 | 73 | 118 | 5 | 19 | 21 | 97 |
| 237 | elongation protein 2 homolog [Rattus norvegicus], gi\|83305835\|sp\|Q496Z0.1\|ELP2_RAT RecName: Full=Elongator complex protein 2; Short=ELP2; AltName: Full=STAT3-interacting protein; AltName: Full=StIP1; AltName: Full=SHINC-2, gi\|72679582\|gb\|AAI00662.1\| Elong | gi\|77628045 | 92 | 117 | 3 | 21 | 22 | 95 |
| 238 | rCG48390, isoform CRA_b [Rattus norvegicus] | gi\|149062533 | 41 | 99 | 29 | 35 | 44 | 55 |
| 239 | N-acetylneuraminic acid synthase [Rattus norvegicus], gi\|149045842\|gb\|EDL98842.1\| N-acetylneuraminic acid synthase (sialic acid synthase) (predicted), isoform CRA_a [Rattus norvegicus], gi\|171847415\|gb\|AAI61979.1\| N-acetylneuraminic acid synthase [Rattus | gi\|164663874 | 40 | 70 | 42 | 33 | 46 | 24 |
| 240 | rCG23159, isoform CRA_a [Rattus norvegicus], gi\|149030115\|gb\|EDL85192.1\| rCG23159, isoform CRA_a [Rattus norvegicus] | gi\|149030114 | 41 | 74 | 51 | 30 | 46 | 28 |
| 241 | Psmd8 protein [Rattus norvegicus] | gi\|77748475 | 32 | 101 | 6 | 14 | 16 | 85 |
| 242 | tubulin folding cofactor B [Rattus norvegicus], gi\|109458545\|ref\|XP_001071286.1\| PREDICTED: similar to Tubulin-specific chaperone B (Tubulin folding cofactor B) (Cytoskeleton-associated protein 1) (Cytoskeleton-associated protein CKAPI) [Rattus norvegicus | gi\|93277119 | 27 | 107 | 28 | 18 | 27 | 80 |
| 243 | PREDICTED: similar to myosin XVIIIa [Rattus norvegicus] | gi\|109491389 (+1) | 233 | 102 | 15 | 31 | 36 | 66 |
| 244 | protein kinase, cAMP dependent regulatory, type II beta [Rattus norvegicus], gi\|400117\|sp\|P12369.3\|KAP3_RAT RecName: Full=cAMP-dependent protein kinase type II-beta regulatory subunit, gi\|149051089\|gb\|EDM03262.1\| protein kinase, cAMP dependent regulatory, | gi\|158081759 (+1) | 46 | 85 | 26 | 35 | 43 | 42 |
| 245 | protein kinase, cAMP dependent, catalytic, beta (predicted), isoform CRA_d [Rattus norvegicus] | gi\|149026195 (+3) | 46 | 86 | 25 | 30 | 38 | 48 |
| 246 | rCG50095 [Rattus norvegicus] | gi\|149064751 | 31 | 88 | 33 | 37 | 47 | 41 |
| 247 | SUMO1 activating enzyme subunit 1 [Rattus norvegicus], gi\|81884451\|sp\|Q6AXQ0.1\|SAE1_RAT RecName: Full=SUMO-activating enzyme subunit 1; AltName: Full=Ubiquitin-like 1-activating enzyme E1A, gi\|50925905\|gb\|AAH79411.1\| SUMO1 activating enzyme subunit 1 [Rat | gi\|58865692 | 39 | 81 | 59 | 14 | 33 | 48 |
| 248 | ribonucleotide reductase M1 [Rattus norvegicus], gi\|55250768\|gb\|AAH85906.1\| Ribonucleotide reductase M1 [Rattus norvegicus], gi\|149068638\|gb\|EDM18190.1\| ribonucleotide reductase M1 (mapped), isoform CRA_b [Rattus norvegicus] | gi\|61557388 | 90 | 125 | 22 | 6 | 13 | 112 |
| 249 | JTV1 [Rattus norvegicus], gi\|123780052\|sp\|Q32PX2.1\|MCA2_RAT RecName: Full=Multisynthetase complex auxiliary component p38; AltName: Full=Protein JTV-1, gi\|81294208\|gb\|AAI07948.1\| JTV1 gene [Rattus norvegicus] | gi\|82654218 | 35 | 97 | 1 | 46 | 46 | 51 |
| 250 | minichromosome maintenance complex component 7 [Rattus norvegicus], gi\|50925575\|gb\|AAH78973.1\| Minichromosome maintenance deficient 7 (S. cerevisiae) [Rattus norvegicus], gi\|149028524\|gb\|EDL83896.1\| minichromosome maintenance deficient 7 (S. cerevisiae), | gi\|148747275 (+1) | 81 | 99 | 8 | 44 | 47 | 52 |
| 251 | PREDICTED: similar to delta isoform of regulatory subunit B56, protein phosphatase 2A isoform 1 [Rattus norvegicus], gi\|109486753\|ref\|XP_001065844.1\| PREDICTED: similar to delta isoform of regulatory subunit B56, protein phosphatase 2A isoform 1 [Rattus n | gi\|109485787 (+1) | 77 | 88 | 22 | 22 | 29 | 59 |
| 252 | Acidic (leucine-rich) nuclear phosphoprotein 32 family, member A [Rattus norvegicus] | gi\|56388748 (+1) | 29 | 91 | 32 | 20 | 30 | 61 |
| 253 | adducin 1 (alpha), isoform CRA_a [Rattus norvegicus] | gi\|149047419 | 70 | 117 | 8 | 16 | 19 | 98 |
| 254 | Mapt protein [Rattus norvegicus], gi\|149054486\|gb\|EDM06303.1\| rCG32645, isoform CRA_g [Rattus norvegicus] | gi\|116487733 | 36 | 82 | 38 | 0 | 12 | 70 |
| 255 | RecName: Full=Protein SET; AltName: Full=Phosphatase 2A inhibitor I2PP2A; Short=I-2PP2A; AltName: Full=Template-activating factor I; Short=TAF-I; AltName: Full=Liver regeneration-related protein LRRGR00002, gi\|2144099\|pir\|\|I51908 Set alpha isoform - rat, | gi\|46396568 (+1) | 33 | 106 | 44 | 1 | 15 | 91 |
| 256 | vinculin (predicted), isoform CRA_a [Rattus norvegicus] | gi\|149031250 (+1) | 124 | 89 | 56 | 0 | 18 | 71 |
| 257 | nucleophosmin (nucleolar phosphoprotein B23, numatrin) [Rattus norvegicus], gi\|114763\|sp\|P13084.1\|NPM_RAT RecName: Full=Nucleophosmin; Short=NPM; AltName: Full=Nucleolar phosphoprotein B23; AltName: Full=Numatrin; AltName: Full=Nucleolar protein NO38, gi\| | gi\|7242160 | 33 | 95 | 23 | 23 | 30 | 65 |
| 258 | spectrin beta 3 [Rattus norvegicus] | gi\|149061992 (+1) | 271 | 67 | 34 | 36 | 47 | 20 |
| 259 | coatomer protein complex, subunit beta 1 [Rattus norvegicus], gi\|116923\|sp\|P23514.1\|COPB_RAT RecName: Full=Coatomer subunit beta; AltName: Full=Beta-coat protein; Short=Beta-COP, gi\|55819\|emb\|CAA40505.1\| beta COP [Rattus norvegicus], gi\|38197648\|gb\|AAH618 | gi\|18158449 | 107 | 81 | 13 | 24 | 28 | 53 |
| 260 | aplysia ras-related homolog A2 [Rattus norvegicus], gi\|31542143\|ref\|NP_058082.2\| ras homolog gene family, member A [Mus musculus], gi\|13633865\|sp\|Q9QUI0.1\|RHOA_MOUSE RecName: Full=Transforming protein RhoA; Flags: Precursor, gi\|47605935\|sp\|P61589.1\|RHOA_R | gi\|16923986 | 22 | 117 | 19 | 0 | 6 | 111 |
| 261 | neuronal cell adhesion molecule long isoform Nc1 [Rattus norvegicus] | gi\|46369969 (+5) | 133 | 97 | 36 | 8 | 19 | 78 |
| 262 | exportin 5 (predicted) [Rattus norvegicus] | gi\|149069362 | 103 | 78 | 47 | 15 | 30 | 48 |
| 263 | PREDICTED: similar to tubulin-specific chaperone d [Rattus norvegicus] | gi\|109492428 | 134 | 69 | 44 | 10 | 24 | 45 |
| 264 | rCG46625 [Rattus norvegicus] | gi\|149064321 | 26 | 100 | 11 | 27 | 30 | 70 |
| 265 | eukaryotic translation initiation factor 3, subunit 9 (eta) [Rattus norvegicus], gi\|88909158\|sp\|Q4G061.1\|EIF3B_RAT RecName: Full=Eukaryotic translation initiation factor 3 subunit B; Short=eIF3b; AltName: Full=Eukaryotic translation initiation factor 3 su | gi\|72255511 | 91 | 101 | 2 | 39 | 40 | 61 |
| 266 | histidyl-tRNA synthetase [Rattus norvegicus], gi\|67678042\|gb\|AAH97969.1\| Histidyl-tRNA synthetase [Rattus norvegicus], gi\|149017268\|gb\|EDL76319.1\| dead end homolog 1 (zebrafish) [Rattus norvegicus] | gi\|70794762 | 57 | 104 | 29 | 2 | 11 | 93 |
| 267 | PREDICTED: similar to FK506-binding protein 4 (Peptidyl-prolyl cis-trans isomerase) (PPIase) (Rotamase) (p59 protein) (HSP-binding immunophilin) (HBI) (FKBP52 protein) (52 kDa FK506-binding protein) (FKBP59) [Rattus norvegicus] | gi\|109472679 (+2) | 62 | 76 | 40 | 11 | 24 | 52 |
| 268 | heat shock 70kDa protein 12A [Rattus norvegicus], gi\|149040506\|gb\|EDL94544.1\| heat shock 70kDa protein 12A (predicted), isoform CRA_a [Rattus norvegicus] | gi\|157818467 | 75 | 101 | 21 | 20 | 27 | 74 |
| 269 | tumor rejection antigen gp96 [Rattus norvegicus], gi\|205716800\|sp\|Q66HD0.2\|ENPL_RAT RecName: Full=Endoplasmin; AltName: Full=Heat shock protein 90 kDa beta member 1; AltName: Full=94 kDa glucose-regulated protein; Short=GRP94; Flags: Precursor, gi\|1490673 | gi\|210032365 | 93 | 80 | 40 | 9 | 22 | 58 |
| 270 | cytoplasmic linker 2 [Rattus norvegicus] | gi\|149063106 | 116 | 94 | 10 | 15 | 18 | 76 |
| 271 | threonyl-tRNA synthetase, isoform CRA_a [Rattus norvegicus] | gi\|149027310 | 83 | 98 | 24 | 14 | 22 | 76 |
| 272 | dynactin 2 [Rattus norvegicus], gi\|81884599\|sp\|Q6AYH5.1\|DCTN2_RAT RecName: Full=Dynactin subunit 2, gi\|50926127\|gb\|AAH79042.1\| Dynactin 2 [Rattus norvegicus], gi\|149066609\|gb\|EDM16482.1\| dynactin 2, isoform CRA_a [Rattus norvegicus] | gi\|51948450 | 44 | 117 | 1 | 13 | 13 | 104 |
| 273 | dimethylarginine dimethylaminohydrolase 1 [Rattus norvegicus], gi\|6831527\|sp\|O08557.3\|DDAH1_RAT RecName: Full=N(G),N(G)-dimethylarginine dimethylaminohydrolase 1; Short=Dimethylarginine dimethylaminohydrolase 1; Short=DDAH-1; AltName: Full=DDAHI; AltName: | gi\|11560131 | 31 | 63 | 47 | 12 | 27 | 36 |
| 274 | peptidylprolyl isomerase D [Rattus norvegicus], gi\|66773787\|sp\|Q6DGG0.3\|PPID_RAT RecName: Full=40 kDa peptidyl-prolyl cis-trans isomerase; Short=PPIase; Short=Rotamase; AltName: Full=Cyclophilin-40; Short=CYP-40, gi\|49900877\|gb\|AAH76386.1\| Peptidylprolyl | gi\|51948528 | 41 | 71 | 34 | 20 | 31 | 40 |
| 275 | proteasome (prosome, macropain) 26S subunit, non-ATPase, 4 [Rattus norvegicus], gi\|3510374\|dbj\|BAA32596.1\| antisecretory factor [Rattus norvegicus], gi\|149030721\|gb\|EDL85758.1\| proteasome (prosome, macropain) 26S subunit, non-ATPase, 4, isoform CRA_a [Rat | gi\|13786158 | 41 | 108 | 2 | 24 | 25 | 83 |
| 276 | glypican 2 (cerebroglycan), isoform CRA_a [Rattus norvegicus] | gi\|149028539 (+1) | 62 | 112 | 4 | 20 | 21 | 91 |
| 277 | eukaryotic translation elongation factor 1 beta 2 [Rattus norvegicus], gi\|149046006\|gb\|EDL98899.1\| rCG22471, isoform CRA_b [Rattus norvegicus], gi\|197245749\|gb\|AAI68738.1\| Eukaryotic translation elongation factor 1 beta 2 [Rattus norvegicus] | gi\|157818179 | 25 | 96 | 13 | 22 | 26 | 70 |
| 278 | Chain A, New Crystal Forms Of A Mu Class Glutathione S-Transferase From Rat Liver, gi\|442968\|pdb\|1GSB\|B Chain B, New Crystal Forms Of A Mu Class | gi\|442967 (+1) | 26 | 69 | 50 | 4 | 20 | 49 |

| | | | | | | | | |
|---|---|---|---|---|---|---|---|---|
| | Glutathione S-Transferase From Rat Liver, gi\|442969\|pdb\|1GSB\|C Chain C, New Crystal Forms Of A Mu Class Glutat | | | | | | | |
| 279 | catenin (cadherin associated protein), beta 1, 88kDa [Mus musculus], gi\|399310\|sp\|Q02248.1\|CTNB1_MOUSE RecName: Full=Catenin beta-1; AltName: Full=Beta-catenin, gi\|192142\|gb\|AAA37280.1\| beta-catenin, gi\|15149346\|gb\|AAK85253.1\| beta-catenin [Rattus norvegi | gi\|6671684 | 85 | 98 | 5 | 37 | 39 | 59 |
| 280 | calpain 2 [Rattus norvegicus], gi\|543926\|sp\|Q07009.3\|CAN2_RAT RecName: Full=Calpain-2 catalytic subunit; AltName: Full=Calpain-2 large subunit; AltName: Full=Calcium-activated neutral proteinase 2; Short=CANP 2; AltName: Full=Calpain M-type; AltName: Full | gi\|8393038 (+1) | 80 | 86 | 30 | 12 | 21 | 65 |
| 281 | transportin 2 (importin 3, karyopherin beta 2b) [Rattus norvegicus], gi\|149037805\|gb\|EDL92165.1\| transportin 2 (importin 3, karyopherin beta 2b) (predicted) [Rattus norvegicus] | gi\|157821505 | 103 | 102 | 9 | 5 | 8 | 94 |
| 282 | EH-domain containing 1 [Mus musculus], gi\|58865448\|ref\|NP_001011939.1\| EH-domain containing 1 [Rattus norvegicus], gi\|109459775\|ref\|XP_001074569.1\| PREDICTED: similar to EH-domain-containing protein 1 (mPAST1) [Rattus norvegicus], gi\|109463451\|ref\|XP_0010 | gi\|7106303 | 61 | 100 | 2 | 18 | 19 | 81 |
| 283 | ADP-ribosylation factor 1 [Homo sapiens], gi\|6680716\|ref\|NP_031502.1\| ADP-ribosylation factor 1 [Mus musculus], gi\|11968098\|ref\|NP_071963.1\| ADP-ribosylation factor 1 [Rattus norvegicus], gi\|28603778\|ref\|NP_788826.1\| ADP-ribosylation factor 1 [Bos taurus] | gi\|4502201 | 21 | 82 | 54 | 0 | 17 | 65 |
| 284 | NCK-associated protein 1, isoform CRA_a [Rattus norvegicus] | gi\|149022387 (+2) | 130 | 97 | 7 | 23 | 25 | 72 |
| 285 | UDP-glucose ceramide glucosyltransferase-like 1 [Rattus norvegicus], gi\|7677176\|gb\|AAF67072.1\|AF200359_1 UDP-glucose glycoprotein:glucosyltransferase precursor [Rattus norvegicus] | gi\|19424302 (+1) | 174 | 119 | 9 | 1 | 4 | 115 |
| 286 | guanine nucleotide binding protein (G protein), alpha inhibiting 3 [Rattus norvegicus], gi\|120997\|sp\|P08753.3\|GNAI3_RAT RecName: Full=Guanine nucleotide-binding protein G(k) subunit alpha; AltName: Full=G(i) alpha-3, gi\|203164\|gb\|AAA40823.1\| GTP-binding p | gi\|6980964 (+1) | 41 | 88 | 1 | 42 | 42 | 46 |
| 287 | brain abundant, membrane attached signal protein 1 [Rattus norvegicus], gi\|730110\|sp\|Q05175.2\|BASP_RAT RecName: Full=Brain acid soluble protein 1; Short=BASP1 protein; AltName: Full=Neuronal axonal membrane protein NAP-22; AltName: Full=22 kDa neuronal ti | gi\|11560135 | 22 | 129 | 2 | 1 | 2 | 127 |
| 288 | proliferating cell nuclear antigen [Rattus norvegicus], gi\|129698\|sp\|P04961.1\|PCNA_RAT RecName: Full=Proliferating cell nuclear antigen; Short=PCNA; AltName: Full=Cyclin, gi\|56862\|emb\|CAA68261.1\| unnamed protein product [Rattus norvegicus], gi\|38014559\|gb | gi\|11693142 | 29 | 91 | 27 | 10 | 19 | 72 |
| 289 | asparaginyl-tRNA synthetase isoform 1 [Rattus norvegicus], gi\|149064471\|gb\|EDM14674.1\| rCG46601, isoform CRA_b [Rattus norvegicus] | gi\|219275589 | 64 | 95 | 11 | 20 | 23 | 72 |
| 290 | kinesin family member 5B [Rattus norvegicus], gi\|109892476\|sp\|Q2PQA9.1\|KINH_RAT RecName: Full=Kinesin-1 heavy chain; AltName: Full=Ubiquitous kinesin heavy chain; Short=UKHC; AltName: Full=Conventional kinesin heavy chain, gi\|83595210\|gb\|ABC25059.1\| kines | gi\|83776543 | 110 | 101 | 7 | 19 | 21 | 80 |
| 291 | hypothetical protein LOC679221 [Rattus norvegicus], gi\|108860788\|sp\|Q3B8Q0.1\|MARE2_RAT RecName: Full=Microtubule-associated protein RP/EB family member 2, gi\|77748365\|gb\|AAI05880.1\| Similar to Microtubule-associated protein RP/EB family member 2 (APC-bind | gi\|155369682 | 37 | 62 | 22 | 39 | 46 | 16 |
| 292 | ribosomal protein S27a [Mus musculus], gi\|13592077\|ref\|NP_112375.1\| ribosomal protein S27a [Rattus norvegicus], gi\|76443694\|ref\|NP_001029037.1\| ribosomal protein S27a [Mus musculus], gi\|82930467\|ref\|XP_889611.1\| PREDICTED: similar to fusion protein: ubiqu | gi\|13195690 | 18 | 85 | 37 | 0 | 12 | 73 |
| 293 | actin related protein 2/3 complex, subunit 1A [Rattus norvegicus], gi\|59797636\|sp\|Q99PD4.1\|ARC1A_RAT RecName: Full=Actin-related protein 2/3 complex subunit 1A, gi\|12667258\|gb\|AAK01364.1\|AF315378_1 suppressor of profilin/p41 of actin-related complex 2/3 [ | gi\|13592137 | 42 | 91 | 10 | 21 | 24 | 67 |
| 294 | COP9 constitutive photomorphogenic homolog subunit 3 [Rattus norvegicus], gi\|81884373\|sp\|Q68FW9.1\|CSN3_RAT RecName: Full=COP9 signalosome complex subunit 3; Short=Signalosome subunit 3; Short=SGN3, gi\|51260717\|gb\|AAH79143.1\| COP9 constitutive photomorphog | gi\|51948372 | 48 | 90 | 8 | 27 | 30 | 60 |
| 295 | splicing factor 3b, subunit 1 isoform 1 [Homo sapiens], gi\|153791358\|ref\|NP_112456.2\| splicing factor 3b, subunit 1 [Mus musculus], gi\|189339231\|ref\|NP_445878.1\| splicing factor 3b, subunit 1 [Rattus norvegicus], gi\|74005042\|ref\|XP_545578.2\| PREDICTED: si | gi\|54112117 | 146 | 83 | 6 | 24 | 26 | 57 |
| 296 | RAB2A, member RAS oncogene family [Rattus norvegicus], gi\|131789\|sp\|P05712.1\|RAB2A_RAT RecName: Full=Ras-related protein Rab-2A, gi\|206555\|gb\|AAA42007.1\| ras protein | gi\|13929006 | 24 | 106 | 10 | 2 | 5 | 101 |
| 297 | hypothetical protein LOC683313 [Rattus norvegicus], gi\|123781839\|sp\|Q4FZU2.1\|K2C6A_RAT RecName: Full=Keratin, type II cytoskeletal 6A; AltName: Full=Cytokeratin-6A; Short=CK 6A; AltName: Full=K6a keratin, gi\|71051822\|gb\|AAH99121.1\| LOC683313 protein [Ratt | gi\|155369696 | 59 | 85 | 40 | 4 | 17 | 68 |
| 298 | G1 to S phase transition 1 [Rattus norvegicus], gi\|50925679\|gb\|AAH79092.1\| G1 to S phase transition 1 [Rattus norvegicus], gi\|149042560\|gb\|EDL96197.1\| G1 to S phase transition 1 [Rattus norvegicus] | gi\|51571949 | 69 | 70 | 31 | 10 | 20 | 50 |
| 299 | hypothetical protein LOC309053 [Rattus norvegicus], gi\|149037235\|gb\|EDL91735.1\| similar to RIKEN cDNA 2310035C23 (predicted) [Rattus norvegicus] | gi\|197385435 | 135 | 81 | 28 | 23 | 32 | 49 |
| 300 | phosphatidylethanolamine binding protein [Rattus norvegicus], gi\|400734\|sp\|P31044.3\|PEBP1_RAT RecName: Full=Phosphatidylethanolamine-binding protein 1; Short=PEBP-1; AltName: Full=HCNPpp; AltName: Full=23 kDa morphine-binding protein; AltName: Full=P23K; | gi\|8393910 | 21 | 66 | 56 | 0 | 18 | 48 |
| 301 | RAB10, member RAS oncogene family [Mus musculus], gi\|61889071\|ref\|NP_059055.2\| RAB10, member RAS oncogene family [Rattus norvegicus], gi\|155372291\|ref\|NP_001094758.1\| ras-related GTP-binding protein RAB10 [Bos taurus], gi\|197099764\|ref\|NP_001126682.1\| RAB | gi\|7710086 | 23 | 94 | 16 | 6 | 11 | 83 |
| 302 | dynamin 1 [Rattus norvegicus], gi\|56054\|emb\|CAA38397.1\| D100 [Rattus norvegicus], gi\|123230379\|emb\|CAM15857.1\| dynamin 1 [Mus musculus], gi\|227123\|prf\|\|1614348A dynamin 1 D100 protein | gi\|18093102 | 96 | 98 | 4 | 22 | 23 | 75 |
| 303 | mitogen-activated protein kinase 1 [Mus musculus], gi\|16758698\|ref\|NP_446294.1\| mitogen-activated protein kinase 1 [Rattus norvegicus], gi\|84579909\|ref\|NP_001033752.1\| mitogen-activated protein kinase 1 [Mus musculus], gi\|52001075\|sp\|P63086.3\|MK01_RAT Rec | gi\|6754632 | 41 | 75 | 16 | 21 | 26 | 49 |
| 304 | glutaminyl-tRNA synthetase [Rattus norvegicus], gi\|51859305\|gb\|AAH82002.1\| Glutaminyl-tRNA synthetase [Rattus norvegicus], gi\|149018523\|gb\|EDL77164.1\| rCG25149, isoform CRA_a [Rattus norvegicus] | gi\|56090285 | 88 | 79 | 3 | 30 | 31 | 48 |
| 305 | protein kinase, cAMP-dependent, regulatory, type 2, alpha, isoform CRA_a [Rattus norvegicus], gi\|149018507\|gb\|EDL77148.1\| protein kinase, cAMP-dependent, regulatory, type 2, alpha, isoform CRA_a [Rattus norvegicus] | gi\|149018505 (+2) | 45 | 91 | 11 | 29 | 32 | 59 |
| 306 | meningioma expressed antigen 5 (hyaluronidase) [Rattus norvegicus], gi\|81867293\|sp\|Q8VIJ5.1\|NCOAT_RAT RecName: Full=Bifunctional protein NCOAT; AltName: Full=Nuclear cytoplasmic O-GlcNAcase and acetyltransferase; AltName: Full=Meningioma-expressed antigen | gi\|18777747 | 103 | 65 | 40 | 17 | 30 | 35 |
| 307 | PREDICTED: similar to transportin 1 [Rattus norvegicus] | gi\|109465974 (+1) | 128 | 78 | 23 | 6 | 13 | 65 |
| 308 | protein phosphatase 2 (formerly 2A), regulatory subunit B (PR 52), alpha isoform, isoform CRA_a [Rattus norvegicus] | gi\|149030343 (+1) | 43 | 75 | 13 | 18 | 22 | 53 |
| 309 | pyrophosphatase (mapped), isoform CRA_b [Rattus norvegicus] | gi\|149038730 | 34 | 46 | 72 | 1 | 24 | 22 |
| 310 | double cortin and calcium/calmodulin-dependent protein kinase-like 1, isoform CRA_c [Rattus norvegicus] | gi\|149064748 | 57 | 76 | 26 | 20 | 28 | 48 |
| 311 | heterogeneous nuclear ribonucleoprotein F [Mus musculus], gi\|25742579\|ref\|NP_071792.1\| heterogeneous nuclear ribonucleoprotein F [Rattus norvegicus], gi\|82546815\|ref\|NP_001032362.1\| heterogeneous nuclear ribonucleoprotein F [Rattus norvegicus], gi\|8254681 | gi\|19527048 | 46 | 72 | 36 | 13 | 24 | 48 |
| 312 | translation initiation factor eIF-2B subunit alpha/beta/delta-like protein [Rattus norvegicus], gi\|81882932\|sp\|Q5HZE4.1\|MTNA_RAT RecName: Full=Methylthioribose-1-phosphate isomerase; Short=MTR-1-P isomerase; Short=M1Pi; AltName: Full=S-methyl-5-thioribose | gi\|58219514 | 40 | 61 | 26 | 26 | 34 | 27 |
| 313 | rCG52086, isoform CRA_a [Rattus norvegicus] | gi\|149030303 | 62 | 99 | 0 | 17 | 17 | 82 |
| 314 | RecName: Full=Tropomyosin alpha-3 chain; AltName: Full=Tropomyosin-3; AltName: Full=Gamma-tropomyosin; AltName: Full=Tropomyosin-5, gi\|438878\|gb\|AAA21721.1\| tropomyosin, gi\|149048012\|gb\|EDM00588.1\| rCG62531, isoform CRA_b [Rattus norvegicus] | gi\|148840439 | 29 | 69 | 54 | 0 | 17 | 52 |
| 315 | calponin 3, acidic [Rattus norvegicus], gi\|584956\|sp\|P37397.1\|CNN3_RAT RecName: Full=Calponin-3; AltName: Full=Calponin, acidic isoform; AltName: Full=Calponin, non-muscle isoform, gi\|458454\|gb\|AAA18590.1\| acidic calponin, gi\|38303945\|gb\|AAH62020.1\| Calpo | gi\|9506501 | 36 | 72 | 34 | 10 | 21 | 51 |
| 316 | ARP3 actin-related protein 3 homolog [Mus musculus], gi\|70912366\|ref\|NP_112330.1\| ARP3 actin-related protein 3 homolog [Rattus norvegicus], gi\|47116573\|sp\|Q99JY9.3\|ARP3_MOUSE RecName: Full=Actin-related protein 3; AltName: Full=Actin-like protein 3, gi\|81 | gi\|23956222 | 47 | 103 | 12 | 5 | 9 | 94 |
| 317 | caspase 3 [Rattus norvegicus], gi\|2506263\|sp\|P55213.2\|CASP3_RAT RecName: Full=Caspase-3; Short=CASP-3; AltName: Full=Apopain; AltName: Full=Cysteine protease CPP32; Short=CPP-32; AltName: Full=Yama protein; AltName: Full=SREBP cleavage activity 1; Short=S | gi\|6978605 | 31 | 81 | 26 | 6 | 14 | 67 |
| 318 | peroxiredoxin 1 [Rattus norvegicus], gi\|2499470\|sp\|Q63716.1\|PRDX1_RAT RecName: Full=Peroxiredoxin-1; AltName: Full=Thioredoxin peroxidase 2; AltName: Full=Thioredoxin-dependent peroxide reductase 2; AltName: Full=Heme-binding 23 kDa protein; AltName: Full | gi\|16923958 | 22 | 65 | 47 | 2 | 17 | 48 |
| 319 | nuclear matrix protein SNEV [Mus musculus], gi\|74095899\|ref\|NP_647549.2\| PRP19/PSO4 pre-mRNA processing factor 19 homolog [Rattus norvegicus], gi\|55976574\|sp\|Q99KP6.1\|PRP19_MOUSE RecName: Full=Pre-mRNA-processing factor 19; AltName: Full=PRP19/PSO4 homolo | gi\|19527358 | 55 | 68 | 18 | 20 | 26 | 42 |
| 320 | fructose-bisphosphate aldolase C [Rattus norvegicus], gi\|113615\|sp\|P09117.3\|ALDOC_RAT RecName: Full=Fructose-bisphosphate aldolase C; AltName: Full=Brain-type aldolase, gi\|202842\|gb\|AAA40717.1\| aldolase C, gi\|4519576\|dbj\|BAA75659.1\| aldolase C [Rattus nor | gi\|6978489 | 39 | 88 | 28 | 6 | 15 | 73 |
| 321 | actin related protein 2/3 complex subunit 4 isoform a [Homo sapiens], gi\|13386054\|ref\|NP_080828.1\| actin related protein 2/3 complex, subunit 4 [Mus musculus], gi\|115495705\|ref\|NP_001069631.1\| actin related protein 2/3 complex, subunit 4 [Bos taurus], gi\| | gi\|5031595 | 20 | 94 | 18 | 0 | 6 | 88 |
| 322 | PREDICTED: similar to Myristoylated alanine-rich C-kinase substrate (MARCKS) (Protein kinase C substrate 80 kDa protein) [Rattus norvegicus] | gi\|109509584 (+1) | 30 | 102 | 7 | 0 | 2 | 100 |
| 323 | seryl-aminoacyl-tRNA synthetase 1, isoform CRA_a [Rattus norvegicus] | gi\|149025695 | 61 | 94 | 8 | 0 | 3 | 91 |
| 324 | G-protein signaling modulator 1 (AGS3-like, C. elegans) isoform a [Rattus norvegicus], gi\|149039294\|gb\|EDL93514.1\| G-protein signalling modulator 1 (AGS3-like, C. elegans), isoform CRA_c [Rattus norvegicus] | gi\|224451084 | 74 | 54 | 29 | 19 | 28 | 26 |
| 325 | ATPase, H+ transporting, V1 subunit E isoform 1, isoform CRA_a [Rattus norvegicus] | gi\|149049573 | 26 | 79 | 21 | 12 | 19 | 60 |
| 326 | basic leucine zipper and W2 domains 2 [Rattus norvegicus], gi\|81882072\|sp\|Q9WTT7.1\|BZW2_RAT RecName: Full=Basic leucine zipper and W2 domain-containing protein 2; AltName: Full=Brain development-related molecule 2, gi\|4426565\|gb\|AAD20436.1\| unknown [Rattu | gi\|19705527 | 48 | 94 | 12 | 7 | 11 | 83 |
| 327 | COP9 constitutive photomorphogenic homolog subunit 5 [Rattus norvegicus], gi\|68534551\|gb\|AAH98736.1\| COP9 constitutive photomorphogenic homolog subunit 5 (Arabidopsis) [Rattus norvegicus], gi\|149060940\|gb\|EDM11550.1\| COP9 (constitutive photomorphogenic) h | gi\|71043620 | 38 | 70 | 19 | 23 | 29 | 41 |
| 328 | ras related v-ral simian leukemia viral oncogene homolog A [Rattus norvegicus], gi\|34328471\|ref\|NP_062364.3\| ras related v-ral simian leukemia viral oncogene homolog A [Mus musculus], gi\|54038996\|sp\|P63322.1\|RALA_RAT RecName: Full=Ras-related protein Ral- | gi\|13592039 | 24 | 99 | 6 | 6 | 8 | 91 |
| 329 | PREDICTED: similar to DNA replication licensing factor MCM3 (DNA polymerase alpha holoenzyme-associated protein P1) (P1-MCM3) [Rattus norvegicus] | gi\|109486863 | 92 | 84 | 20 | 10 | 16 | 68 |

| | | | | | | | | |
|---|---|---|---|---|---|---|---|---|
| 330 | COP9 (constitutive photomorphogenic) homolog, subunit 7b (Arabidopsis thaliana) (predicted), isoform CRA_a [Rattus norvegicus], gi\|149016358\|gb\|EDL75604.1\| COP9 (constitutive photomorphogenic) homolog, subunit 7b (Arabidopsis thaliana) (predicted), isofor | gi\|149016354 | 30 | 92 | 11 | 14 | 17 | 75 |
| 331 | PREDICTED: similar to hect (homologous to the E6-AP (UBE3A) carboxyl terminus) domain and RCC1 (CHC1)-like domain (RLD) 2 [Rattus norvegicus] | gi\|109495994 | 492 | 59 | 1 | 32 | 32 | 27 |
| 332 | esterase D/formylglutathione hydrolase [Rattus norvegicus], gi\|193806001\|sp\|B0BNE5.1\|ESTD_RAT RecName: Full=S-formylglutathione hydrolase; Short=FGH; AltName: Full=Esterase D, gi\|149049957\|gb\|EDM02281.1\| esterase D/formylglutathione hydrolase (mapped), is | gi\|157823267 | 31 | 55 | 33 | 15 | 25 | 30 |
| 333 | thimet oligopeptidase 1 [Rattus norvegicus], gi\|189038855\|sp\|P24155.4\|THOP1_RAT RecName: Full=Thimet oligopeptidase; AltName: Full=Endo-oligopeptidase A; AltName: Full=Endopeptidase 24.15; AltName: Full=PZ-peptidase; AltName: Full=Soluble metallo-endopept | gi\|126723582 | 78 | 43 | 62 | 5 | 25 | 18 |
| 334 | Rho GDP dissociation inhibitor (GDI) alpha [Mus musculus], gi\|55742827\|ref\|NP_001007006.1\| Rho GDP dissociation inhibitor (GDI) alpha [Rattus norvegicus], gi\|21759130\|sp\|Q99PT1.3\|GDIR1_MOUSE RecName: Full=Rho GDP-dissociation inhibitor 1; Short=Rho GDI 1; | gi\|31982030 | 23 | 71 | 39 | 4 | 16 | 55 |
| 335 | catenin (cadherin associated protein), alpha 2 (predicted), isoform CRA_b [Rattus norvegicus] | gi\|149036451 | 102 | 90 | 1 | 11 | 11 | 79 |
| 336 | splicing factor 3b, subunit 3 [Rattus norvegicus], gi\|149038189\|gb\|EDL92549.1\| splicing factor 3b, subunit 3 (predicted) [Rattus norvegicus] | gi\|157822163 | 72 | 79 | 21 | 8 | 15 | 64 |
| 337 | cullin 3 [Rattus norvegicus], gi\|149016247\|gb\|EDL75493.1\| cullin 3 (predicted), isoform CRA_a [Rattus norvegicus] | gi\|157818315 | 86 | 87 | 8 | 17 | 20 | 67 |
| 338 | protein-L-isoaspartate (D-aspartate) O-methyltransferase 1, isoform CRA_b [Rattus norvegicus] | gi\|149039521 (+2) | 30 | 66 | 32 | 15 | 25 | 41 |
| 339 | coatomer protein complex, subunit gamma [Rattus norvegicus], gi\|123797612\|sp\|Q4AEF8.1\|COPG_RAT RecName: Full=Coatomer subunit gamma; AltName: Full=Gamma-coat protein; Short=Gamma-COP, gi\|71891597\|dbj\|BAE16997.1\| coatomer protein complex, subunit gamma [Ra | gi\|73532768 | 98 | 75 | 6 | 25 | 27 | 48 |
| 340 | lysyl-tRNA synthetase [Rattus norvegicus], gi\|53733461\|gb\|AAH83652.1\| Lysyl-tRNA synthetase [Rattus norvegicus], gi\|149038242\|gb\|EDL92602.1\| lysyl-tRNA synthetase, isoform CRA_b [Rattus norvegicus] | gi\|55741637 | 72 | 76 | 0 | 21 | 21 | 55 |
| 341 | prolyl endopeptidase-like [Rattus norvegicus], gi\|57921016\|gb\|AAH89111.1\| Prolyl endopeptidase-like [Rattus norvegicus], gi\|149050505\|gb\|EDM02678.1\| similar to RIKEN cDNA D030028O16 [Rattus norvegicus] | gi\|58219516 | 73 | 67 | 5 | 25 | 27 | 40 |
| 342 | squamous cell carcinoma antigen recognized by T-cells 3 [Rattus norvegicus], gi\|149063643\|gb\|EDM13966.1\| squamous cell carcinoma antigen recognized by T-cells 3 (predicted) [Rattus norvegicus] | gi\|157820585 | 109 | 59 | 16 | 24 | 29 | 30 |
| 343 | PREDICTED: similar to ubiquitin protein ligase E3A isoform 2 [Rattus norvegicus], gi\|109462056\|ref\|XP_001055915.1\| PREDICTED: similar to ubiquitin protein ligase E3A isoform 2 [Rattus norvegicus] | gi\|109458840 | 98 | 76 | 14 | 17 | 21 | 55 |
| 344 | phosphoribosyl pyrophosphate synthetase-associated protein 2 [Rattus norvegicus], gi\|73955994\|ref\|XP_546654.2\| PREDICTED: similar to phosphoribosyl pyrophosphate synthetase-associated protein 2 isoform 1 [Canis familiaris], gi\|24418482\|sp\|O08618.1\|KPRB_RA | gi\|16923984 | 41 | 86 | 8 | 13 | 16 | 70 |
| 345 | cell division cycle 42 isoform 1 [Homo sapiens], gi\|6753364\|ref\|NP_033991.1\| cell division cycle 42 [Mus musculus], gi\|55742784\|ref\|NP_001003254.1\| cell division cycle 42 [Canis lupus familiaris], gi\|61889112\|ref\|NP_741991.3\| cell division cycle 42 [Rattu | gi\|4757952 | 21 | 81 | 24 | 0 | 8 | 73 |
| 346 | ADP-ribosylation factor-like 8A [Mus musculus], gi\|157818451\|ref\|NP_001102541.1\| ADP-ribosylation factor-like 8A [Rattus norvegicus], gi\|81902053\|sp\|Q8VEH3.1\|ARL8A_MOUSE RecName: Full=ADP-ribosylation factor-like protein 8A; AltName: Full=ADP-ribosylation | gi\|23956194 | 21 | 94 | 15 | 0 | 5 | 89 |
| 347 | actin-related protein 10 homolog ARP10 actin-related protein 10 homolog [Rattus norvegicus], gi\|56541122\|gb\|AAH87143.1\| Actin-related protein 10 homolog (S. cerevisiae) [Rattus norvegicus], gi\|149051386\|gb\|EDM03559.1\| ARP10 actin-related protein 10 homolo | gi\|57527131 | 46 | 79 | 0 | 29 | 29 | 50 |
| 348 | testicular dynamin [Rattus norvegicus] | gi\|391872 | 96 | 79 | 2 | 14 | 15 | 64 |
| 349 | phosphoglycerate dehydrogenase [Rattus norvegicus], gi\|3122856\|sp\|O08651.3\|SERA_RAT RecName: Full=D-3-phosphoglycerate dehydrogenase; Short=3-PGDH, gi\|1944614\|emb\|CAA66374.1\| D-3-phosphoglycerate dehydrogenase [Rattus norvegicus], gi\|7688285\|emb\|CAB89828. | gi\|13928850 | 56 | 73 | 28 | 11 | 20 | 53 |
| 350 | rCG38346, isoform CRA_d [Rattus norvegicus] | gi\|149028082 (+1) | 110 | 61 | 30 | 12 | 21 | 40 |
| 351 | 6-phosphogluconolactonase (predicted), isoform CRA_a [Rattus norvegicus] | gi\|149036104 (+1) | 39 | 78 | 29 | 5 | 14 | 64 |
| 352 | RecName: Full=Hypoxia up-regulated protein 1; AltName: Full=150 kDa oxygen-regulated protein; AltName: Full=Orp150; Flags: Precursor, gi\|1480453\|gb\|AAB05672.1\| 150 kDa oxygen regulated protein | gi\|10720174 (+1) | 111 | 68 | 23 | 6 | 13 | 55 |
| 353 | contactin 2 [Rattus norvegicus] | gi\|149058636 | 113 | 73 | 22 | 3 | 10 | 63 |
| 354 | tubulin tyrosine ligase-like family, member 12 (predicted) [Rattus norvegicus] | gi\|149065748 (+1) | 79 | 54 | 20 | 18 | 24 | 30 |
| 355 | Rtn3 protein [Rattus norvegicus] | gi\|120538101 (+1) | 28 | 84 | 2 | 5 | 6 | 78 |
| 356 | eukaryotic translation initiation factor 3, subunit 6 interacting protein [Rattus norvegicus] | gi\|149065953 | 67 | 78 | 0 | 25 | 25 | 53 |
| 357 | RecName: Full=Aspartate aminotransferase, cytoplasmic; AltName: Full=Transaminase A; AltName: Full=Glutamate oxaloacetate transaminase 1, gi\|345752\|pir\|\|S29028 aspartate transaminase (EC 2.6.1.1) (clone 8C7) - human, gi\|38197390\|gb\|AAH61877.1\| Glutamic-ox | gi\|122065118 | 46 | 59 | 51 | 0 | 16 | 43 |
| 358 | RAB1B, member RAS oncogene family [Rattus norvegicus], gi\|109463007\|ref\|XP_001052971.1\| PREDICTED: similar to Ras-related protein Rab-1B [Rattus norvegicus], gi\|149042337\|gb\|EDL96044.1\| rCG63295 [Rattus norvegicus], gi\|149062037\|gb\|EDM12460.1\| rCG48195, i | gi\|158341664 | 22 | 76 | 11 | 11 | 14 | 62 |
| 359 | RAD23 homolog B [Rattus norvegicus], gi\|123789085\|sp\|Q4KMA2.1\|RD23B_RAT RecName: Full=UV excision repair protein RAD23 homolog B, gi\|68534740\|gb\|AAH98674.1\| RAD23 homolog B (S. cerevisiae) [Rattus norvegicus], gi\|149037170\|gb\|EDL91701.1\| RAD23b homolog (S | gi\|70778952 | 43 | 75 | 24 | 2 | 10 | 65 |
| 360 | cullin 5 [Rattus norvegicus], gi\|13124593\|sp\|Q9JJ31.3\|CUL5_RAT RecName: Full=Cullin-5; Short=CUL-5; AltName: Full=Vasopressin-activated calcium-mobilizing receptor; AltName: Full=VACM-1, gi\|7381163\|gb\|AAF61416.1\|AF135115_1 vasopressin-activated calcium-mo | gi\|12083629 (+1) | 91 | 71 | 11 | 14 | 17 | 54 |
| 361 | basic transcription factor 3 isoform B [Homo sapiens], gi\|56605700\|ref\|NP_001008310.1\| basic transcription factor 3 [Rattus norvegicus], gi\|109077568\|ref\|XP_001100502.1\| PREDICTED: basic transcription factor 3 isoform 3 [Macaca mulatta], gi\|114599709\|ref\| | gi\|20070130 | 18 | 78 | 34 | 0 | 11 | 67 |
| 362 | nucleoside diphosphate kinase B [Rattus norvegicus], gi\|127984\|sp\|P19804.1\|NDKB_RAT RecName: Full=Nucleoside diphosphate kinase B; Short=NDP kinase B; Short=NDK B; AltName: Full=P18, gi\|205662\|gb\|AAA41684.1\| nucleoside diphosphate kinase, gi\|55778652\|gb\|A | gi\|55926145 | 17 | 66 | 37 | 0 | 12 | 54 |
| 363 | casein kinase 2, alpha 1 polypeptide [Rattus norvegicus], gi\|729878\|sp\|P19139.2\|CSK21_RAT RecName: Full=Casein kinase II subunit alpha; Short=CK II, gi\|415716\|gb\|AAA74462.1\| casein kinase II alpha subunit, gi\|60551761\|gb\|AAH91130.1\| Csnk2a1 protein [Rattu | gi\|16758674 | 45 | 65 | 13 | 19 | 23 | 42 |
| 364 | importin 4 [Rattus norvegicus], gi\|149063984\|gb\|EDM14254.1\| importin 4 (predicted), isoform CRA_b [Rattus norvegicus] | gi\|157823031 | 119 | 87 | 11 | 6 | 9 | 78 |
| 365 | phosphoribosylglycinamide formyltransferase [Rattus norvegicus], gi\|62657641\|ref\|XP_573258.1\| PREDICTED: similar to Trifunctional purine biosynthetic protein adenosine-3 [Rattus norvegicus], gi\|109493898\|ref\|XP_001068637.1\| PREDICTED: similar to Trifuncti | gi\|197927388 | 108 | 57 | 22 | 14 | 21 | 36 |
| 366 | phosphoribosylaminoimidazole carboxylase, phosphoribosylaminoimidazole succinocarboxamide synthetase [Rattus norvegicus], gi\|1709928\|sp\|P51583.3\|PUR6_RAT RecName: Full=Multifunctional protein ADE2; Includes: RecName: Full=Phosphoribosylaminoimidazole-succ | gi\|18266726 | 47 | 86 | 4 | 11 | 12 | 74 |
| 367 | SAR1a gene homolog [Mus musculus], gi\|56090263\|ref\|NP_001007740.1\| SAR1a gene homolog 1 [Rattus norvegicus], gi\|13542685\|gb\|AAH05549.1\| SAR1 gene homolog A (S. cerevisiae) [Mus musculus], gi\|50925795\|gb\|AAH79228.1\| SAR1 homolog A (S. cerevisiae) [Rattus n | gi\|21703344 | 22 | 82 | 19 | 0 | 6 | 76 |
| 368 | Aldehyde dehydrogenase 1 family, member L1 [Rattus norvegicus] | gi\|57921067 | 99 | 60 | 17 | 23 | 28 | 32 |
| 369 | Obg-like ATPase 1 [Rattus norvegicus], gi\|215275692\|sp\|A0JPJ7.1\|OLA1_RAT RecName: Full=Obg-like ATPase 1, gi\|117558623\|gb\|AAI27458.1\| Obg-like ATPase 1 [Rattus norvegicus] | gi\|76677911 | 45 | 67 | 19 | 24 | 30 | 37 |
| 370 | cytoplasmic FMR1 interacting protein 1 [Rattus norvegicus], gi\|149031480\|gb\|EDL86460.1\| cytoplasmic FMR1 interacting protein 1 (predicted) [Rattus norvegicus] | gi\|157822937 | 145 | 74 | 6 | 17 | 19 | 55 |
| 371 | PREDICTED: hypothetical protein [Rattus norvegicus], gi\|109489722\|ref\|XP_001079214.1\| PREDICTED: hypothetical protein [Rattus norvegicus] | gi\|109487775 (+1) | 33 | 63 | 37 | 6 | 18 | 45 |
| 372 | erythrocyte protein band 4.1-like 1 isoform L [Rattus norvegicus], gi\|4587120\|dbj\|BAA76625.1\| rat brain 4.1(L) [Rattus norvegicus] | gi\|11067407 | 171 | 57 | 20 | 15 | 21 | 36 |
| 373 | Proteasome (prosome, macropain) subunit, beta type 1 [Rattus norvegicus], gi\|149047099\|gb\|EDL99819.1\| proteasome (prosome, macropain) subunit, beta type 1, isoform CRA_a [Rattus norvegicus] | gi\|37231712 | 26 | 76 | 22 | 0 | 7 | 69 |
| 374 | tumor protein, translationally-controlled 1 [Mus musculus], gi\|16758734\|ref\|NP_446319.1\| tumor protein, translationally-controlled 1 [Rattus norvegicus], gi\|149255389\|ref\|XP_001480708.1\| PREDICTED: hypothetical protein [Mus musculus], gi\|149255783\|ref\|XP_ | gi\|6678437 | 19 | 84 | 16 | 4 | 9 | 75 |
| 375 | tropomyosin 4 [Rattus norvegicus], gi\|136081\|sp\|P09495.3\|TPM4_RAT RecName: Full=Tropomyosin alpha-4 chain; AltName: Full=Tropomyosin-4; Short=TM-4, gi\|207504\|gb\|AAA42291.1\| tropomyosin TM4, gi\|763182\|emb\|CAA68360.1\| fibroblast tropomyosin 4 [Rattus norveg | gi\|6981672 | 29 | 51 | 45 | 0 | 14 | 37 |
| 376 | glypican, gi\|38181579\|gb\|AAH61572.1\| Gpc1 protein [Rattus norvegicus] | gi\|506417 | 62 | 95 | 0 | 0 | 0 | 95 |
| 377 | PREDICTED: similar to low density lipoprotein receptor-related protein 1 [Rattus norvegicus], gi\|109482078\|ref\|XP_001056970.1\| PREDICTED: similar to low density lipoprotein receptor-related protein 1 [Rattus norvegicus], gi\|149066587\|gb\|EDM16460.1\| rCG595 | gi\|62652278 | 505 | 86 | 0 | 0 | 0 | 86 |
| 378 | contactin 1 [Rattus norvegicus], gi\|17432928\|sp\|Q63198.2\|CNTN1_RAT RecName: Full=Contactin-1; AltName: Full=Neural cell surface protein F3; Flags: Precursor, gi\|1498194\|dbj\|BAA07504.1\| F3 [Rattus norvegicus], gi\|149017608\|gb\|EDL76612.1\| contactin 1 [Rattu | gi\|16923964 | 113 | 83 | 4 | 4 | 5 | 78 |
| 379 | guanine nucleotide binding protein (G protein), beta polypeptide 2-like 1 [Homo sapiens], gi\|6680047\|ref\|NP_032169.1\| guanine nucleotide binding protein (G protein), beta polypeptide 2 like 1 [Mus musculus], gi\|28461223\|ref\|NP_786996.1\| guanine nucleotide | gi\|5174447 | 35 | 81 | 7 | 12 | 14 | 67 |
| 380 | nascent-polypeptide-associated complex alpha polypeptide (predicted), isoform CRA_c [Rattus norvegicus] | gi\|149029725 (+1) | 15 | 52 | 45 | 8 | 22 | 30 |
| 381 | similar to Dendritic cell protein GA17 (predicted) [Rattus norvegicus] | gi\|149022814 | 43 | 65 | 0 | 35 | 35 | 30 |
| 382 | proteasome (prosome, macropain) subunit, alpha type 5 [Mus musculus], gi\|23110942\|ref\|NP_002781.2\| proteasome alpha 5 subunit [Homo sapiens], gi\|62751982\|ref\|NP_001015566.1\| proteasome (prosome, macropain) subunit, alpha type, 5 [Bos taurus], gi\|222136590 | gi\|7106387 | 26 | 66 | 17 | 4 | 9 | 57 |
| 383 | serine/threonine kinase receptor associated protein [Rattus norvegicus], gi\|81883769\|sp\|Q5XIG8.1\|STRAP_RAT RecName: Full=Serine-threonine kinase receptor-associated protein; AltName: Full=UNR-interacting protein, gi\|53733514\|gb\|AAH83714.1\| Serine/threonin | gi\|58865512 | 38 | 57 | 17 | 16 | 21 | 36 |
| 384 | glutathione S-transferase mu 5 [Rattus norvegicus], gi\|81917962\|sp\|Q9Z1B2.3\|GSTM5_RAT RecName: Full=Glutathione S-transferase Mu 5; AltName: Full=GST class-mu 5, gi\|4099365\|gb\|AAD00603.1\| glutathione s-transferase M5 [Rattus norvegicus], gi\|149025650\|gb\|E | gi\|25282395 | 27 | 50 | 23 | 7 | 14 | 36 |

| | | | | | | | | |
|---|---|---|---|---|---|---|---|---|
| 385 | similar to Pol(yrC)-binding protein 1 (Alpha-CP1) (hnRNP-E1) (predicted) [Rattus norvegicus] | gi\|149036630 (+1) | 36 | 51 | 40 | 10 | 23 | 28 |
| 386 | rCG20491, isoform CRA_a [Rattus norvegicus] | gi\|149040822 (+1) | 167 | 64 | 13 | 15 | 19 | 45 |
| 387 | proteasome alpha 4 subunit [Rattus norvegicus], gi\|130862\|sp\|P21670.1\|PSA4_RAT RecName: Full=Proteasome subunit alpha type-4; AltName: Full=Proteasome component C9; AltName: Full=Macropain subunit C9; AltName: Full=Multicatalytic endopeptidase complex sub | gi\|8394069 | 29 | 76 | 21 | 2 | 9 | 67 |
| 388 | RecName: Full=Excitatory amino acid transporter 1; AltName: Full=Sodium-dependent glutamate/aspartate transporter 1; Short=GLAST-1; AltName: Full=Glial glutamate transporter; AltName: Full=Solute carrier family 1 member 3, gi\|56269\|emb\|CAA45276.1\| glutama | gi\|232176 (+1) | 60 | 92 | 2 | 8 | 9 | 83 |
| 389 | keratin 17 [Rattus norvegicus], gi\|81891674\|sp\|Q6IFU8.1\|K1C17_RAT RecName: Full=Keratin, type I cytoskeletal 17; AltName: Full=Cytokeratin-17; Short=CK-17; AltName: Full=Keratin-17; Short=K17; AltName: Full=Type I keratin Ka17, gi\|46485094\|tpg\|DAA04484.1\| | gi\|47087085 | 48 | 46 | 27 | 0 | 9 | 37 |
| 390 | synapsin I isoform a [Rattus norvegicus], gi\|6686305\|sp\|P09951.3\|SYN1_RAT RecName: Full=Synapsin-1; AltName: Full=Synapsin I, gi\|206921\|gb\|AAA42145.1\| synapsin Ia, gi\|149044403\|gb\|EDL97724.1\| synapsin I, isoform CRA_a [Rattus norvegicus] | gi\|9507159 | 74 | 86 | 0 | 0 | 0 | 86 |
| 391 | RAB3A, member RAS oncogene family [Mus musculus], gi\|61098195\|ref\|NP_037150.2\| RAB3A, member RAS oncogene family [Rattus norvegicus], gi\|178056554\|ref\|NP_001116651.1\| RAB3A, member RAS oncogene family [Sus scrofa], gi\|187936991\|ref\|NP_001120756.1\| RAB3A, | gi\|6679593 | 25 | 72 | 18 | 10 | 16 | 56 |
| 392 | ATP synthase, H+ transporting, mitochondrial F1 complex, alpha subunit, isoform 1, isoform CRA_d [Rattus norvegicus] | gi\|149029483 (+4) | 55 | 68 | 1 | 22 | 22 | 46 |
| 393 | RAB6A, member RAS oncogene family [Mus musculus], gi\|13638404\|sp\|P35279.4\|RAB6A_MOUSE RecName: Full=Ras-related protein Rab-6A; Short=Rab-6, gi\|7670415\|dbj\|BAA95059.1\| unnamed protein product [Mus musculus], gi\|26341820\|dbj\|BAC34572.1\| unnamed protein pro | gi\|13195674 | 24 | 70 | 22 | 0 | 7 | 63 |
| 394 | rCG26771, isoform CRA_b [Rattus norvegicus], gi\|149022783\|gb\|EDL79677.1\| rCG26771, isoform CRA_b [Rattus norvegicus] | gi\|149022782 (+1) | 77 | 83 | 9 | 6 | 9 | 74 |
| 395 | PREDICTED: similar to destrin [Rattus norvegicus] | gi\|62665569 (+1) | 19 | 53 | 31 | 0 | 10 | 43 |
| 396 | A kinase (PRKA) anchor protein (gravin) 12, isoform CRA_a [Rattus norvegicus] | gi\|149038539 (+3) | 173 | 53 | 19 | 12 | 18 | 35 |
| 397 | similar to microfilament and actin filament cross-linker protein isoform b, isoform CRA_a [Rattus norvegicus] | gi\|149023883 (+1) | 609 | 38 | 14 | 17 | 21 | 17 |
| 398 | calcium/calmodulin-dependent protein kinase II beta isoform2 [Rattus norvegicus], gi\|226693349\|ref\|NP_031621.3\| calcium/calmodulin-dependent protein kinase II beta [Mus musculus], gi\|94730394\|sp\|P28652.2\|KCC2B_MOUSE RecName: Full=Calcium/calmodulin-depend | gi\|108796657 (+6) | 60 | 57 | 4 | 29 | 30 | 27 |
| 399 | calmodulin 2 [Homo sapiens], gi\|5901912\|ref\|NP_008819.1\| calmodulin 1 [Homo sapiens], gi\|6680832\|ref\|NP_031615.1\| calmodulin 2 [Mus musculus], gi\|6680834\|ref\|NP_031616.1\| calmodulin 3 [Mus musculus], gi\|6753244\|ref\|NP_033920.1\| calmodulin 1 [Mus musculus] | gi\|4502549 (+1) | 17 | 93 | 0 | 0 | 0 | 93 |
| 400 | heterogeneous nuclear ribonucleoprotein H1 [Mus musculus], gi\|46396669\|sp\|O35737.3\|HNRH1_MOUSE RecName: Full=Heterogeneous nuclear ribonucleoprotein H; Short=hnRNP H; Contains: RecName: Full=Heterogeneous nuclear ribonucleoprotein H, N-terminally process | gi\|10946928 | 49 | 71 | 12 | 14 | 18 | 53 |
| 401 | eukaryotic translation initiation factor 3 subunit D [Rattus norvegicus], gi\|125628629\|ref\|NP_061219.2\| eukaryotic translation initiation factor 3, subunit 7 (zeta) [Mus musculus], gi\|81884616\|sp\|Q6AYK8.1\|EIF3D_RAT RecName: Full=Eukaryotic translation ini | gi\|51948536 | 64 | 72 | 2 | 28 | 29 | 43 |
| 402 | aspartyl aminopeptidase, isoform CRA_a [Rattus norvegicus], gi\|149016179\|gb\|EDL75425.1\| aspartyl aminopeptidase, isoform CRA_a [Rattus norvegicus], gi\|149016181\|gb\|EDL75427.1\| aspartyl aminopeptidase, isoform CRA_a [Rattus norvegicus] | gi\|149016178 (+2) | 52 | 76 | 0 | 8 | 8 | 68 |
| 403 | neutral alpha-glucosidase AB [Rattus norvegicus], gi\|149062331\|gb\|EDM12754.1\| alpha glucosidase 2 alpha neutral subunit (predicted) [Rattus norvegicus] | gi\|157822919 | 91 | 84 | 1 | 6 | 6 | 78 |
| 404 | Parkinson disease protein 7 [Rattus norvegicus], gi\|56404680\|sp\|O88767.1\|PARK7_RAT RecName: Full=Protein DJ-1; AltName: Full=Parkinson disease protein 7 homolog; AltName: Full=Contraception-associated protein 1; Short=Protein CAP1; AltName: Full=Fertility | gi\|16924002 | 20 | 50 | 41 | 1 | 14 | 36 |
| 405 | PREDICTED: similar to DNA replication licensing factor MCM4 (CDC21 homolog) (P1-CDC21) [Rattus norvegicus], gi\|109494801\|ref\|XP_001068436.1\| PREDICTED: similar to DNA replication licensing factor MCM4 (CDC21 homolog) (P1-CDC21) [Rattus norvegicus], gi\|149 | gi\|34870013 | 97 | 64 | 7 | 21 | 23 | 41 |
| 406 | Proteasome (prosome, macropain) 26S subunit, ATPase 2 [Rattus norvegicus] | gi\|38181888 (+1) | 49 | 75 | 2 | 19 | 20 | 55 |
| 407 | adenine phosphoribosyl transferase (predicted), isoform CRA_a [Rattus norvegicus] | gi\|149038396 | 20 | 54 | 31 | 0 | 10 | 44 |
| 408 | pre-mRNA processing factor 8 [Mus musculus], gi\|62656550\|ref\|XP_213385.3\| PREDICTED: similar to Pre-mRNA-processing-splicing factor 8 (Splicing factor Prp8) (PRP8 homolog) (220 kDa U5 snRNP-specific protein) (p220) [Rattus norvegicus], gi\|109491316\|ref\|XP | gi\|115583687 | 274 | 66 | 3 | 16 | 17 | 49 |
| 409 | PREDICTED: similar to CLIP-associating protein 1 [Rattus norvegicus] | gi\|109498506 (+1) | 165 | 51 | 20 | 17 | 23 | 28 |
| 410 | glutathione S-transferase mu 7 [Rattus norvegicus], gi\|121721\|sp\|P08009.2\|GSTM4_RAT RecName: Full=Glutathione S-transferase Yb-3; AltName: Full=Chain 4; AltName: Full=GST Yb3; AltName: Full=GST class-mu 3, gi\|204513\|gb\|AAA41292.1\| glutathione-S-transferas | gi\|13592152 | 26 | 43 | 45 | 1 | 15 | 28 |
| 411 | similar to RIKEN cDNA 2610110G12, isoform CRA_e [Rattus norvegicus] | gi\|149031796 | 45 | 69 | 3 | 19 | 20 | 49 |
| 412 | unnamed protein product [Rattus norvegicus] | gi\|1334284 (+2) | 58 | 94 | 3 | 0 | 1 | 93 |
| 413 | RAB1A, member RAS oncogene family isoform 1 [Homo sapiens], gi\|6679587\|ref\|NP_033022.1\| RAB1, member RAS oncogene family [Mus musculus], gi\|45433570\|ref\|NP_112352.2\| RAB1, member RAS oncogene family [Rattus norvegicus], gi\|55741705\|ref\|NP_001003153.1\| RAB | gi\|4758988 | 23 | 66 | 15 | 17 | 22 | 44 |
| 414 | RecName: Full=General vesicular transport factor p115; AltName: Full=Protein USO1 homolog; AltName: Full=Transcytosis-associated protein; Short=TAP; AltName: Full=Vesicle-docking protein, gi\|538153\|gb\|AAA62632.1\| p115, gi\|149033810\|gb\|EDL88606.1\| vesicle | gi\|1171952 | 107 | 52 | 20 | 7 | 13 | 39 |
| 415 | proteasome subunit alpha type 2 [Rattus norvegicus], gi\|134031994\|ref\|NP_032970.2\| proteasome subunit alpha type 2 [Mus musculus], gi\|130851\|sp\|P17220.3\|PSA2_RAT RecName: Full=Proteasome subunit alpha type-2; AltName: Full=Proteasome component C3; AltName | gi\|8394063 | 26 | 66 | 24 | 1 | 9 | 57 |
| 416 | RecName: Full=Neural cell adhesion molecule L1; Short=N-CAM L1; AltName: Full=Nerve-growth factor-inducible large external glycoprotein; Short=NILE; AltName: CD_antigen=CD171; Flags: Precursor | gi\|20455467 (+1) | 141 | 67 | 1 | 16 | 16 | 51 |
| 417 | ARP2 actin-related protein 2 homolog [Rattus norvegicus], gi\|62899645\|sp\|Q5M7U6.1\|ARP2_RAT RecName: Full=Actin-related protein 2; AltName: Full=Actin-like protein 2, gi\|56789539\|gb\|AAH88442.1\| ARP2 actin-related protein 2 homolog (yeast) [Rattus norvegicu | gi\|57164143 | 45 | 75 | 7 | 9 | 11 | 64 |
| 418 | malate dehydrogenase, mitochondrial [Rattus norvegicus], gi\|122065494\|sp\|P04636.2\|MDHM_RAT RecName: Full=Malate dehydrogenase, mitochondrial; Flags: Precursor, gi\|38648863\|gb\|AAH63165.1\| Malate dehydrogenase 2, NAD (mitochondrial) [Rattus norvegicus], gi\| | gi\|42476181 | 36 | 81 | 13 | 0 | 4 | 77 |
| 419 | IQ motif containing GTPase activating protein 1 [Rattus norvegicus], gi\|149057332\|gb\|EDM08655.1\| IQ motif containing GTPase activating protein 1 (predicted), isoform CRA_b [Rattus norvegicus] | gi\|210032529 | 189 | 25 | 43 | 9 | 23 | 2 |
| 420 | chloride intracellular channel 4, isoform CRA_b [Rattus norvegicus] | gi\|149024253 | 29 | 44 | 30 | 13 | 22 | 22 |
| 421 | eukaryotic translation initiation factor 3, subunit 6 48kDa [Homo sapiens], gi\|45476573\|ref\|NP_032414.1\| eukaryotic translation initiation factor 3, subunit 6 [Mus musculus], gi\|58865556\|ref\|NP_001011990.1\| eukaryotic translation initiation factor 3, subu | gi\|4503521 | 52 | 77 | 0 | 15 | 15 | 62 |
| 422 | myosin regulatory light chain MRCL2 isoform A [Homo sapiens], gi\|21728376\|ref\|NP_075891.1\| myosin, light chain 12B, regulatory [Mus musculus], gi\|203097095\|ref\|NP_059039.2\| myosin light chain, regulatory B [Rattus norvegicus], gi\|222144324\|ref\|NP_00113841 | gi\|15809016 (+1) | 20 | 75 | 20 | 0 | 6 | 69 |
| 423 | adaptor-related protein complex 1, gamma 1 subunit [Rattus norvegicus], gi\|149038154\|gb\|EDL92514.1\| adaptor-related protein complex 1, gamma 1 subunit, isoform CRA_b [Rattus norvegicus], gi\|187469711\|gb\|AAI66845.1\| Ap1g1 protein [Rattus norvegicus] | gi\|189491695 | 91 | 63 | 3 | 18 | 19 | 44 |
| 424 | PREDICTED: similar to phosphoribosylformylglycinamidine synthase [Rattus norvegicus] | gi\|109491072 (+1) | 137 | 15 | 74 | 0 | 23 | 0 |
| 425 | FK506 binding protein 12-rapamycin associated protein 1 [Rattus norvegicus], gi\|1169736\|sp\|P42346.1\|FRAP_RAT RecName: Full=FKBP12-rapamycin complex-associated protein; AltName: Full=FK506-binding protein 12-rapamycin complex-associated protein 1; AltName: | gi\|9845251 | 289 | 43 | 6 | 26 | 28 | 15 |
| 426 | RAP1B, member of RAS oncogene family [Rattus norvegicus], gi\|51338715\|sp\|Q62636.2\|RAP1B_RAT RecName: Full=Ras-related protein Rap-1b; AltName: Full=GTP-binding protein smg p21B; Flags: Precursor, gi\|47716868\|gb\|AAT37620.1\| RAP1B [Rattus norvegicus], gi\|51 | gi\|52138628 | 21 | 77 | 5 | 0 | 2 | 75 |
| 427 | Bat1 [Rattus norvegicus] | gi\|19773876 (+1) | 49 | 55 | 25 | 3 | 11 | 44 |
| 428 | protein phosphatase 2A activator, regulatory subunit 4 [Rattus norvegicus], gi\|149039087\|gb\|EDL93307.1\| protein phosphatase 2A, regulatory subunit B (PR 53) (predicted) [Rattus norvegicus], gi\|187469721\|gb\|AAI66861.1\| Protein phosphatase 2A activator, reg | gi\|157819489 | 37 | 49 | 7 | 13 | 15 | 34 |
| 429 | Structure specific recognition protein 1 [Rattus norvegicus], gi\|83305804\|sp\|Q04931.2\|SSRP1_RAT RecName: Full=FACT complex subunit SSRP1; AltName: Full=Facilitates chromatin transcription complex subunit SSRP1; AltName: Full=Structure-specific recognition | gi\|54020668 | 81 | 44 | 20 | 20 | 26 | 18 |
| 430 | peroxiredoxin 6 [Rattus norvegicus], gi\|5902791\|sp\|O35244.3\|PRDX6_RAT RecName: Full=Peroxiredoxin-6; AltName: Full=Antioxidant protein 2; AltName: Full=1-Cys peroxiredoxin; Short=1-Cys PRX; AltName: Full=Acidic calcium-independent phospholipase A2; Short= | gi\|16758348 | 25 | 27 | 59 | 0 | 19 | 8 |
| 431 | eukaryotic translation initiation factor 3, subunit 8, 110kDa, isoform CRA_a [Rattus norvegicus] | gi\|149067892 (+1) | 73 | 50 | 0 | 14 | 14 | 36 |
| 432 | inosine monophosphate dehydrogenase 2 [Rattus norvegicus], gi\|38014713\|gb\|AAH60585.1\| IMP (inosine monophosphate) dehydrogenase 2 [Rattus norvegicus] | gi\|40018566 | 56 | 62 | 7 | 10 | 12 | 50 |
| 433 | NMDA receptor regulated 1 [Rattus norvegicus], gi\|149064834\|gb\|EDM14985.1\| NMDA receptor-regulated gene 1 (predicted), isoform CRA_b [Rattus norvegicus] | gi\|157818303 | 101 | 55 | 22 | 9 | 16 | 39 |
| 434 | small inducible cytokine subfamily E, member 1 [Rattus norvegicus], gi\|71051686\|gb\|AAH98675.1\| Small inducible cytokine subfamily E, member 1 [Rattus norvegicus], gi\|149025978\|gb\|EDL82221.1\| small inducible cytokine subfamily E, member 1 [Rattus norvegicu | gi\|75832035 | 35 | 58 | 0 | 15 | 15 | 43 |
| 435 | serine/threonine specific protein phosphatase [Rattus norvegicus], gi\|74138620\|dbj\|BAE27131.1\| unnamed protein product [Mus musculus], gi\|74219496\|dbj\|BAE29521.1\| unnamed protein product [Mus musculus], gi\|148680181\|gb\|EDL12128.1\| protein phosphatase 3, c | gi\|4584820 (+1) | 58 | 62 | 20 | 0 | 6 | 56 |
| 436 | quinoid dihydropteridine reductase [Rattus norvegicus], gi\|118601\|sp\|P11348.1\|DHPR_RAT RecName: Full=Dihydropteridine reductase; AltName: Full=HDHPR; AltName: Full=Quinoid dihydropteridine reductase, gi\|576086\|pdb\|1DIR\|A Chain A, Crystal Structure Of A Mo | gi\|11693160 (+1) | 26 | 70 | 10 | 2 | 5 | 65 |
| 437 | ADP-ribosylation factor-like 3 [Rattus norvegicus], gi\|584774\|sp\|P37996.2\|ARL3_RAT RecName: Full=ADP-ribosylation factor-like protein 3; AltName: Full=ARD3, gi\|560006\|gb\|AAA50861.1\| ADP-ribosylation factor-like protein 3, gi\|562785\|emb\|CAA54246.1\| ARF-li | gi\|12083661 | 20 | 56 | 26 | 1 | 9 | 47 |
| 438 | RNA terminal phosphate cyclase domain 1 [Rattus norvegicus], gi\|51261274\|gb\|AAH79374.1\| RNA terminal phosphate cyclase domain 1 [Rattus norvegicus], gi\|149025788\|gb\|EDL82031.1\| RNA terminal phosphate cyclase domain 1, isoform CRA_a [Rattus norvegicus] | gi\|51948426 | 39 | 32 | 21 | 16 | 23 | 9 |
| 439 | nardilysin, N-arginine dibasic convertase 1 [Rattus norvegicus] | gi\|149035699 (+2) | 133 | 33 | 49 | 0 | 15 | 18 |

| | | | | | | | | |
|---|---|---|---|---|---|---|---|---|
| 440 | cullin 1 [Mus musculus], gi\|157820743\|ref\|NP_001102097.1\| cullin 1 [Rattus norvegicus], gi\|224044991\|ref\|XP_002198372.1\| PREDICTED: hypothetical protein [Taeniopygia guttata], gi\|13124104\|sp\|Q9WTX6.1\|CUL1_MOUSE RecName: Full=Cullin-1; Short=CUL-1, gi\|5815 | gi\|7549752 | 90 | 67 | 7 | 12 | 14 | 53 |
| 441 | rCG41048, isoform CRA_a [Rattus norvegicus] | gi\|149029060 | 441 | 54 | 4 | 22 | 23 | 31 |
| 442 | ribosomal protein S3 [Mus musculus], gi\|57164151\|ref\|NP_001009239.1\| ribosomal protein S3 [Rattus norvegicus], gi\|57102362\|ref\|XP_534008.1\| PREDICTED: similar to 40S ribosomal protein S3 isoform 1 [Canis familiaris], gi\|73987935\|ref\|XP_849947.1\| PREDICTED | gi\|6755372 | 27 | 62 | 8 | 12 | 15 | 47 |
| 443 | hypothetical protein LOC223601 [Mus musculus], gi\|187282120\|ref\|NP_001119739.1\| hypothetical protein LOC299909 [Rattus norvegicus], gi\|52782789\|sp\|Q921M7.1\|FA49B_MOUSE RecName: Full=Protein FAM49B, gi\|15030175\|gb\|AAH11343.1\| Fam49b protein [Mus musculus], | gi\|21450053 | 37 | 34 | 50 | 2 | 18 | 16 |
| 444 | proteasome (prosome, macropain) subunit, beta type 5 [Rattus norvegicus], gi\|149063910\|gb\|EDM14180.1\| proteasome (prosome, macropain) subunit, beta type 5, isoform CRA_a [Rattus norvegicus] | gi\|160333293 | 29 | 76 | 9 | 0 | 3 | 73 |
| 445 | COP9 constitutive photomorphogenic homolog subunit 6 [Rattus norvegicus], gi\|149028519\|gb\|EDL83891.1\| COP9 (constitutive photomorphogenic) homolog, subunit 6 (Arabidopsis thaliana) (predicted), isoform CRA_a [Rattus norvegicus] | gi\|157821399 | 38 | 61 | 7 | 14 | 16 | 45 |
| 446 | Chain A, Annexin V Complex With Heparin Oligosaccharides, gi\|126030730\|pdb\|2IE6\|A Chain A, Annexin V Under 2.0 Mpa Pressure Of Xenon, gi\|126030731\|pdb\|2IE7\|A Chain A, Annexin V Under 2.0 Mpa Pressure Of Nitrous Oxide, gi\|157829732\|pdb\|1A8A\|A Chain A, Rat | gi\|14488466 (+2) | 36 | 48 | 11 | 21 | 24 | 24 |
| 447 | cullin-associated and neddylation-dissociated 2 (putative), isoform CRA_c [Rattus norvegicus] | gi\|149049691 (+1) | 136 | 37 | 33 | 6 | 16 | 21 |
| 448 | proteasome (prosome, macropain) subunit, alpha type 1 [Rattus norvegicus], gi\|130849\|sp\|P18420.2\|PSA1_RAT RecName: Full=Proteasome subunit alpha type-1; AltName: Full=Proteasome component C2; AltName: Full=Macropain subunit C2; AltName: Full=Multicatalyti | gi\|8394060 | 30 | 62 | 16 | 0 | 5 | 57 |
| 449 | PREDICTED: similar to Histone deacetylase 6 (HD6) (Histone deacetylase mHDA2) [Rattus norvegicus], gi\|109511550\|ref\|XP_001057931.1\| PREDICTED: similar to Histone deacetylase 6 (HD6) (Histone deacetylase mHDA2) [Rattus norvegicus] | gi\|109510484 | 125 | 62 | 14 | 6 | 10 | 52 |
| 450 | hypothetical protein LOC681996 [Rattus norvegicus], gi\|149025274\|gb\|EDL81641.1\| rCG20659, isoform CRA_b [Rattus norvegicus], gi\|165970860\|gb\|AAI58699.1\| LOC681996 protein [Rattus norvegicus] | gi\|169234816 | 38 | 53 | 22 | 7 | 14 | 39 |
| 451 | moesin, isoform CRA_a [Rattus norvegicus] | gi\|149042266 | 68 | 55 | 18 | 2 | 8 | 47 |
| 452 | RecName: Full=6-phosphogluconate dehydrogenase, decarboxylating, gi\|149024656\|gb\|EDL81153.1\| rCG31394, isoform CRA_a [Rattus norvegicus] | gi\|205686170 | 53 | 53 | 26 | 3 | 11 | 42 |
| 453 | proteasome (prosome, macropain) subunit, alpha type 6 [Rattus norvegicus], gi\|23110944\|ref\|NP_002782.1\| proteasome alpha 6 subunit [Homo sapiens], gi\|114053135\|ref\|NP_001039427.1\| proteasome (prosome, macropain) subunit, alpha type, 6 [Bos taurus], gi\|556 | gi\|8394076 | 27 | 75 | 3 | 2 | 3 | 72 |
| 454 | chloride intracellular channel 1 [Rattus norvegicus], gi\|81911115\|sp\|Q6MG61.1\|CLIC1_RAT RecName: Full=Chloride intracellular channel protein 1, gi\|46237607\|emb\|CAE83985.1\| chloride intracellular channel 1 [Rattus norvegicus], gi\|71122383\|gb\|AAH99823.1\| Ch | gi\|50657380 | 27 | 43 | 32 | 6 | 16 | 27 |
| 455 | eukaryotic translation initiation factor 2, subunit 1 alpha [Rattus norvegicus], gi\|13385624\|ref\|NP_080390.1\| eukaryotic translation initiation factor 2, subunit 1 alpha [Mus musculus], gi\|28461253\|ref\|NP_787007.1\| eukaryotic translation initiation factor | gi\|9506571 | 36 | 59 | 21 | 3 | 10 | 49 |
| 456 | synaptojanin 1, isoform CRA_a [Rattus norvegicus] | gi\|149059817 (+3) | 145 | 45 | 21 | 6 | 13 | 32 |
| 457 | ATP synthase, H+ transporting, mitochondrial F1 complex, beta polypeptide, isoform CRA_a [Rattus norvegicus] | gi\|149029718 (+1) | 56 | 58 | 2 | 10 | 11 | 47 |
| 458 | hypothetical protein LOC364073 [Rattus norvegicus], gi\|149040979\|gb\|EDL94936.1\| rCG20177 [Rattus norvegicus] | gi\|157819755 | 73 | 56 | 6 | 22 | 24 | 32 |
| 459 | heat shock protein 5 [Rattus norvegicus], gi\|121574\|sp\|P06761.1\|GRP78_RAT RecName: Full=78 kDa glucose-regulated protein; AltName: Full=GRP 78; AltName: Full=Heat shock 70 kDa protein 5; AltName: Full=Immunoglobulin heavy chain-binding protein; Short=BiP; | gi\|25742763 | 72 | 78 | 0 | 0 | 0 | 78 |
| 460 | F-actin capping protein alpha-1 subunit [Rattus norvegicus], gi\|109467357\|ref\|XP_001068917.1\| PREDICTED: similar to capping protein (actin filament) muscle Z-line, alpha 1 [Rattus norvegicus], gi\|205690878\|sp\|B2GUZ5.1\|CAZA1_RAT RecName: Full=F-actin-cappi | gi\|161086978 | 33 | 45 | 28 | 4 | 13 | 32 |
| 461 | RecName: Full=Protein disulfide-isomerase A3; AltName: Full=Disulfide isomerase ER-60; AltName: Full=ERp60; AltName: Full=58 kDa microsomal protein; AltName: Full=p58; AltName: Full=ERp57; AltName: Full=HIP-70; AltName: Full=Q-2; Flags: Precursor, gi\|3838 | gi\|1352384 (+1) | 57 | 70 | 2 | 0 | 1 | 69 |
| 462 | Dcps protein [Rattus norvegicus] | gi\|59808733 (+1) | 38 | 44 | 21 | 19 | 26 | 18 |
| 463 | calnexin [Rattus norvegicus], gi\|543922\|sp\|P35565.1\|CALX_RAT RecName: Full=Calnexin; Flags: Precursor, gi\|310085\|gb\|AAA21015.1\| calnexin [Rattus sp.], gi\|149052451\|gb\|EDM04268.1\| calnexin, isoform CRA_a [Rattus norvegicus], gi\|149052452\|gb\|EDM04269.1\| cal | gi\|25282419 | 67 | 69 | 2 | 8 | 9 | 60 |
| 464 | rCG25653, isoform CRA_g [Rattus norvegicus] | gi\|149018243 (+1) | 28 | 74 | 2 | 14 | 15 | 59 |
| 465 | hypothetical protein LOC301742 [Rattus norvegicus], gi\|149065190\|gb\|EDM15266.1\| similar to Coatomer gamma-2 subunit (Gamma-2 coat protein) (Gamma-2 COP) (predicted) [Rattus norvegicus] | gi\|157817775 | 98 | 63 | 2 | 13 | 14 | 49 |
| 466 | Phosphofructokinase, muscle [Rattus norvegicus], gi\|149032171\|gb\|EDL87083.1\| phosphofructokinase, muscle, isoform CRA_a [Rattus norvegicus], gi\|149032172\|gb\|EDL87084.1\| phosphofructokinase, muscle, isoform CRA_a [Rattus norvegicus], gi\|149032173\|gb\|EDL870 | gi\|62825891 | 85 | 74 | 2 | 4 | 5 | 69 |
| 467 | CDC23 (cell division cycle 23, yeast, homolog), isoform CRA_b [Rattus norvegicus] | gi\|149017186 (+1) | 66 | 49 | 0 | 26 | 26 | 23 |
| 468 | proteasome (prosome, macropain) 26S subunit, non-ATPase, 5 (predicted), isoform CRA_a [Rattus norvegicus] | gi\|149038941 | 56 | 61 | 6 | 12 | 14 | 47 |
| 469 | adenosine monophosphate deaminase 2 (isoform L) [Rattus norvegicus], gi\|226801521\|sp\|Q02356.2\|AMPD2_RAT RecName: Full=AMP deaminase 2; AltName: Full=AMP deaminase isoform L, gi\|149025670\|gb\|EDL81913.1\| similar to adenosine monophosphate deaminase 2 (isofo | gi\|156119591 | 95 | 52 | 4 | 12 | 13 | 39 |
| 470 | translin [Rattus norvegicus], gi\|6136059\|sp\|P97891.1\|TSN_CRIGR RecName: Full=Translin, gi\|9652067\|gb\|AAF91387.1\|AF262356_1 translin [Rattus norvegicus], gi\|1770102\|emb\|CAA66669.1\| translin [Cricetulus griseus], gi\|149033085\|gb\|EDL87903.1\| translin [Rattus | gi\|11120712 (+1) | 26 | 45 | 28 | 0 | 9 | 36 |
| 471 | actin, beta-like 2 [Rattus norvegicus], gi\|149059329\|gb\|EDM10336.1\| similar to cytoplasmic beta-actin (predicted) [Rattus norvegicus] | gi\|157823033 | 42 | 16 | 12 | 2 | 6 | 10 |
| 472 | rCG61772, isoform CRA_a [Rattus norvegicus] | gi\|149051518 (+3) | 84 | 71 | 3 | 2 | 3 | 68 |
| 473 | cytosolic branched chain aminotransferase [Rattus norvegicus], gi\|149048991\|gb\|EDM01445.1\| branched chain aminotransferase 1, cytosolic, isoform CRA_a [Rattus norvegicus] | gi\|13374912 (+4) | 45 | 53 | 16 | 8 | 13 | 40 |
| 474 | rCG38907, isoform CRA_b [Rattus norvegicus] | gi\|149031314 | 94 | 45 | 9 | 21 | 24 | 21 |
| 475 | Arhgef7 protein [Rattus norvegicus] | gi\|126362051 | 82 | 45 | 0 | 17 | 17 | 28 |
| 476 | RecName: Full=Farnesyl pyrophosphate synthetase; Short=FPP synthetase; Short=FPS; AltName: Full=Farnesyl diphosphate synthetase; AltName: Full=Cholesterol-regulated 39 kDa protein; Short=CR 39; Includes: RecName: Full=Dimethylallyltranstransferase; Includ | gi\|120478 (+2) | 41 | 36 | 48 | 0 | 15 | 21 |
| 477 | RAB5A, member RAS oncogene family [Rattus norvegicus], gi\|3309068\|gb\|AAC26004.1\| small GTP-binding protein rab5 [Rattus norvegicus], gi\|149027113\|gb\|EDL82849.1\| RAB5A, member RAS oncogene family [Rattus norvegicus], gi\|171847366\|gb\|AAI61848.1\| Rab5a prote | gi\|12083645 | 24 | 61 | 18 | 3 | 9 | 52 |
| 478 | RAP1, GTP-GDP dissociation stimulator 1 (predicted), isoform CRA_a [Rattus norvegicus] | gi\|149026086 (+1) | 58 | 46 | 11 | 15 | 18 | 28 |
| 479 | vesicle-fusing ATPase [Rattus norvegicus], gi\|81917630\|sp\|Q9QUL6.1\|NSF_RAT RecName: Full=Vesicle-fusing ATPase; AltName: Full=N-ethylmaleimide-sensitive fusion protein; Short=NEM-sensitive fusion protein; AltName: Full=Vesicular-fusion protein NSF, gi\|508 | gi\|13489067 (+2) | 83 | 58 | 0 | 10 | 10 | 48 |
| 480 | COP9 (constitutive photomorphogenic) homolog, subunit 7a (Arabidopsis thaliana) (predicted) [Rattus norvegicus] | gi\|149049450 | 30 | 66 | 5 | 14 | 16 | 50 |
| 481 | Cdc42 effector, short isoform [Rattus norvegicus] | gi\|87299586 | 64 | 70 | 0 | 4 | 4 | 66 |
| 482 | myosin Ib [Rattus norvegicus], gi\|13431669\|sp\|Q05096.1\|MYO1B_RAT RecName: Full=Myosin-Ib; AltName: Full=Myosin I alpha; AltName: Full=MMI-alpha; Short=MMIa; AltName: Full=Myosin heavy chain myr 1, gi\|56733\|emb\|CAA48287.1\| myosin I heavy chain [Rattus norv | gi\|16758890 (+1) | 132 | 46 | 20 | 11 | 17 | 29 |
| 483 | MAP-kinase activating death domain [Rattus norvegicus], gi\|81861041\|sp\|O08873.1\|MADD_RAT RecName: Full=MAP kinase-activating death domain protein; AltName: Full=Rab3 GDP/GTP exchange factor, gi\|1947050\|gb\|AAC53149.1\| Rab3 GDP/GTP exchange protein [Rattus | gi\|16758360 | 178 | 28 | 27 | 12 | 21 | 7 |
| 484 | RAB3C, member RAS oncogene family [Mus musculus], gi\|51338595\|sp\|P62824.1\|RAB3C_RAT RecName: Full=Ras-related protein Rab-3C, gi\|51338605\|sp\|P62823.1\|RAB3C_MOUSE RecName: Full=Ras-related protein Rab-3C, gi\|12597437\|gb\|AAG60047.1\|AF312037_1 small GTP bind | gi\|13470090 (+1) | 26 | 61 | 10 | 0 | 3 | 58 |
| 485 | hemoglobin beta chain complex [Rattus norvegicus], gi\|122514\|sp\|P02091.3\|HBB1_RAT RecName: Full=Hemoglobin subunit beta-1; AltName: Full=Hemoglobin beta-1 chain; AltName: Full=Beta-1-globin; AltName: Full=Hemoglobin beta chain, major-form, gi\|55823\|emb\|CA | gi\|17985949 | 16 | 54 | 15 | 0 | 5 | 49 |
| 486 | rCG27987, isoform CRA_a [Rattus norvegicus] | gi\|149065081 (+3) | 176 | 69 | 1 | 0 | 0 | 69 |
| 487 | heat shock protein 4 like [Rattus norvegicus], gi\|149048788\|gb\|EDM01329.1\| heat shock 70kDa protein 4-like (predicted), isoform CRA_b [Rattus norvegicus], gi\|195539870\|gb\|AAI68157.1\| Hspa4l protein [Rattus norvegicus] | gi\|197927441 | 94 | 32 | 40 | 6 | 19 | 13 |
| 488 | ATP-binding cassette, sub-family E (OABP), member 1 [Rattus norvegicus], gi\|197098652\|ref\|NP_001126534.1\| ATP-binding cassette, sub-family E (OABP), member 1 [Pongo abelii], gi\|55731829\|emb\|CAH92618.1\| hypothetical protein [Pongo abelii], gi\|149037946\|gb\| | gi\|157823309 | 67 | 58 | 1 | 20 | 20 | 38 |
| 489 | ribosomal protein S4 | gi\|227229 (+1) | 30 | 49 | 0 | 17 | 17 | 32 |
| 490 | eukaryotic translation elongation factor 1 delta [Rattus norvegicus], gi\|51259466\|gb\|AAH79391.1\| Eukaryotic translation elongation factor 1 delta (guanine nucleotide exchange protein) [Rattus norvegicus] | gi\|61556967 | 72 | 52 | 6 | 16 | 18 | 34 |
| 491 | acidic (leucine-rich) nuclear phosphoprotein 32 family, member B [Rattus norvegicus], gi\|81917438\|sp\|Q9EST6.1\|AN32B_RAT RecName: Full=Acidic leucine-rich nuclear phosphoprotein 32 family member B; AltName: Full=Proliferation-related acidic leucine-rich pr | gi\|18777770 | 31 | 45 | 22 | 3 | 10 | 35 |
| 492 | COP9 constitutive photomorphogenic homolog subunit 8 [Rattus norvegicus], gi\|55976171\|sp\|Q6P4Z9.1\|CSN8_RAT RecName: Full=COP9 signalosome complex subunit 8; Short=Signalosome subunit 8; Short=SGN8; AltName: Full=JAB1-containing signalosome subunit 8; AltN | gi\|61557351 | 23 | 59 | 9 | 4 | 7 | 52 |
| 493 | sterile alpha and TIR motif containing 1 [Rattus norvegicus], gi\|149053540\|gb\|EDM05357.1\| sterile alpha and TIR motif containing 1 (predicted) [Rattus norvegicus] | gi\|157786658 | 80 | 64 | 1 | 16 | 16 | 48 |
| 494 | neuroblastoma ras oncogene [Rattus norvegicus], gi\|464551\|sp\|Q04970.1\|RASN_RAT RecName: Full=GTPase NRas; AltName: Full=Transforming protein N-Ras; Flags: Precursor, gi\|56769\|emb\|CAA48460.1\| p21 protein [Rattus norvegicus], gi\|68534734\|gb\|AAH98659.1\| Neur | gi\|18158431 | 21 | 71 | 1 | 2 | 2 | 69 |

| | | | | | | | | |
|---|---|---|---|---|---|---|---|---|
| 495 | platelet-activating factor acetylhydrolase, isoform Ib, beta subunit [Rattus norvegicus], gi\|40254624\|ref\|NP_032801.2\| platelet-activating factor acetylhydrolase, isoform Ib, beta subunit [Mus musculus], gi\|6093640\|sp\|O35264.1\|PA1B2_RAT RecName: Full=Plat | gi\|11693154 | 26 | 48 | 26 | 2 | 10 | 38 |
| 496 | thioredoxin reductase 1, isoform CRA_a [Rattus norvegicus] | gi\|149067338 (+3) | 67 | 45 | 33 | 0 | 10 | 35 |
| 497 | phospholipase C, beta 1 [Rattus norvegicus], gi\|130223\|sp\|P10687.1\|PLCB1_RAT RecName: Full=1-phosphatidylinositol-4,5-bisphosphate phosphodiesterase beta-1; AltName: Full=Phosphoinositide phospholipase C; AltName: Full=Phospholipase C-beta-1; Short=PLC-be | gi\|117647200 (+1) | 138 | 67 | 9 | 0 | 3 | 64 |
| 498 | cytokine induced apoptosis inhibitor 1 [Rattus norvegicus], gi\|53733543\|gb\|AAH83753.1\| Cytokine induced apoptosis inhibitor 1 [Rattus norvegicus], gi\|149032432\|gb\|EDL87323.1\| cytokine induced apoptosis inhibitor 1 [Rattus norvegicus] | gi\|56090353 | 33 | 36 | 19 | 12 | 18 | 18 |
| 499 | Chain A, G Protein Heterotrimer Gi_alpha_1 Beta_1 Gamma_2 With Gdp Bound, gi\|2392128\|pdb\|1AGR\|A Chain A, Complex Of Alf4-Activated Gi-Alpha-1 With Rgs4, gi\|2392129\|pdb\|1AGR\|D Chain D, Complex Of Alf4-Activated Gi-Alpha-1 With Rgs4, gi\|4930007\|pdb\|1CIP\|A C | gi\|1942397 (+1) | 40 | 51 | 0 | 25 | 25 | 26 |
| 500 | PREDICTED: similar to GTPase activating protein and VPS9 domains 1 [Rattus norvegicus] | gi\|109468053 (+1) | 160 | 31 | 36 | 13 | 24 | 7 |
| 501 | O-sialoglycoprotein endopeptidase [Rattus norvegicus], gi\|149033627\|gb\|EDL88425.1\| O-sialoglycoprotein endopeptidase, isoform CRA_b [Rattus norvegicus], gi\|165971402\|gb\|AAI58593.1\| Osgep protein [Rattus norvegicus] | gi\|189303591 | 36 | 42 | 26 | 10 | 18 | 24 |
| 502 | ZW10 interacting protein-1 [Rattus norvegicus], gi\|21759469\|sp\|Q8VIL3.1\|ZWINT_RAT RecName: Full=ZW10 interactor; AltName: Full=ZW10-interacting protein 1; Short=Zwint-1; AltName: Full=SNAP25-interacting protein 30; AltName: Full=Scoilin, gi\|17063945\|gb\|AA | gi\|22129759 | 30 | 41 | 12 | 12 | 16 | 25 |
| 503 | PREDICTED: similar to cell adhesion molecule with homology to L1CAM [Rattus norvegicus] | gi\|109474014 | 137 | 33 | 29 | 0 | 9 | 24 |
| 504 | glutamate oxaloacetate transaminase 2 [Rattus norvegicus], gi\|112987\|sp\|P00507.2\|AATM_RAT RecName: Full=Aspartate aminotransferase, mitochondrial; AltName: Full=mAspAT; AltName: Full=Transaminase A; AltName: Full=Glutamate oxaloacetate transaminase 2; Alt | gi\|6980972 | 47 | 69 | 0 | 0 | 0 | 69 |
| 505 | protein phosphatase 1G (formerly 2C), magnesium-dependent, gamma isoform [Rattus norvegicus], gi\|22087629\|gb\|AAM90993.1\| protein phosphatase PP2C gamma [Rattus norvegicus], gi\|38303971\|gb\|AAH62083.1\| Protein phosphatase 1G (formerly 2C), magnesium-depende | gi\|22219444 | 59 | 59 | 9 | 3 | 6 | 53 |
| 506 | RecName: Full=Isopentenyl-diphosphate Delta-isomerase 1; AltName: Full=Isopentenyl pyrophosphate isomerase 1; Short=IPP isomerase 1; Short=IPPI1, gi\|127800114\|gb\|AAH89786.2\| Isopentenyl-diphosphate delta isomerase 1 [Rattus norvegicus] | gi\|147744566 (+1) | 26 | 43 | 17 | 9 | 14 | 29 |
| 507 | vimentin [Rattus norvegicus], gi\|401365\|sp\|P31000.2\|VIME_RAT RecName: Full=Vimentin, gi\|57480\|emb\|CAA44722.1\| vimentin [Rattus norvegicus], gi\|38197662\|gb\|AAH61847.1\| Vimentin [Rattus norvegicus] | gi\|14389299 (+1) | 54 | 47 | 5 | 15 | 17 | 30 |
| 508 | ankyrin repeat and FYVE domain containing 1 (predicted), isoform CRA_b [Rattus norvegicus] | gi\|149053305 | 112 | 31 | 30 | 8 | 17 | 14 |
| 509 | mevalonate (diphospho) decarboxylase [Rattus norvegicus], gi\|2498339\|sp\|Q62967.1\|ERG19_RAT RecName: Full=Diphosphomevalonate decarboxylase; AltName: Full=Mevalonate pyrophosphate decarboxylase; AltName: Full=Mevalonate (diphospho)decarboxylase, gi\|1322245 | gi\|13592005 (+1) | 44 | 32 | 28 | 9 | 18 | 14 |
| 510 | eukaryotic translation initiation factor 3, subunit I [Rattus norvegicus], gi\|187471122\|sp\|B0BNA7.1\|EIF3I_RAT RecName: Full=Eukaryotic translation initiation factor 3 subunit I; Short=eIF3i; AltName: Full=Eukaryotic translation initiation factor 3 subunit | gi\|169234840 | 36 | 47 | 2 | 15 | 16 | 31 |
| 511 | thioredoxin-like 1 [Rattus norvegicus], gi\|212722176\|ref\|NP_001131150.1\| hypothetical protein LOC100192458 [Zea mays], gi\|81916316\|sp\|Q920J4.3\|TXNL1_RAT RecName: Full=Thioredoxin-like protein 1; AltName: Full=Thioredoxin-related protein, gi\|15430626\|gb\|AA | gi\|18266686 | 32 | 26 | 37 | 4 | 16 | 10 |
| 512 | Na+/K+ -ATPase beta 3 subunit [Rattus norvegicus], gi\|3121778\|sp\|Q63377.1\|AT1B3_RAT RecName: Full=Sodium/potassium-transporting ATPase subunit beta-3; AltName: Full=Sodium/potassium-dependent ATPase subunit beta-3; Short=ATPB-3; AltName: CD_antigen=CD298, | gi\|6978553 | 32 | 56 | 0 | 18 | 18 | 38 |
| 513 | O-linked N-acetylglucosamine transferase [Rattus norvegicus], gi\|149042180\|gb\|EDL95887.1\| O-linked N-acetylglucosamine (GlcNAc) transferase (UDP-N-acetylglucosamine:polypeptide-N-acetylglucosaminyl transferase), isoform CRA_b [Rattus norvegicus], gi\|14904 | gi\|139948535 (+1) | 116 | 46 | 7 | 13 | 15 | 31 |
| 514 | RecName: Full=Apolipoprotein A-IV; Short=Apo-AIV; Short=ApoA-IV; Flags: Precursor, gi\|202941\|gb\|AAA40747.1\| preapolipoprotein A-IV, gi\|202943\|gb\|AAA40748.1\| apolipoprotein A-IV, gi\|60552712\|gb\|AAH91159.1\| Apolipoprotein A-IV [Rattus norvegicus], gi\|149041 | gi\|114008 (+1) | 44 | 40 | 35 | 0 | 11 | 29 |
| 515 | ATPase, Na+/K+ transporting, alpha 1 polypeptide, isoform CRA_a [Rattus norvegicus], gi\|149030486\|gb\|EDL85523.1\| ATPase, Na+/K+ transporting, alpha 1 polypeptide, isoform CRA_a [Rattus norvegicus] | gi\|149030485 (+2) | 112 | 54 | 6 | 9 | 11 | 43 |
| 516 | CTP synthase II [Rattus norvegicus], gi\|81883462\|sp\|Q5U2N0.1\|PYRG2_RAT RecName: Full=CTP synthase 2; AltName: Full=UTP--ammonia ligase 2; AltName: Full=CTP synthetase 2, gi\|55250782\|gb\|AAH85949.1\| CTP synthase II [Rattus norvegicus] | gi\|78042611 | 66 | 59 | 3 | 7 | 8 | 51 |
| 517 | PREDICTED: similar to Glucocorticoid receptor DNA-binding factor 1 (GAP-associated protein p190) [Rattus norvegicus] | gi\|109461308 | 168 | 32 | 16 | 14 | 19 | 13 |
| 518 | VAMP (vesicle-associated membrane protein)-associated protein A [Rattus norvegicus], gi\|122066704\|sp\|Q9Z270.3\|VAPA_RAT RecName: Full=Vesicle-associated membrane protein-associated protein A; Short=VAMP-associated protein A; Short=VAMP-A; Short=VAP-A; AltN | gi\|118142811 | 28 | 48 | 5 | 2 | 4 | 44 |
| 519 | minichromosome maintenance deficient 6 (MIS5 homolog, S. pombe) (S. cerevisiae), isoform CRA_b [Rattus norvegicus] | gi\|149058718 (+1) | 88 | 42 | 14 | 13 | 17 | 25 |
| 520 | PREDICTED: similar to HECT domain containing 1 [Rattus norvegicus] | gi\|109478203 | 289 | 30 | 15 | 12 | 17 | 13 |
| 521 | ribosomal protein S2 [Rattus norvegicus] | gi\|2920833 | 27 | 45 | 0 | 8 | 8 | 37 |
| 522 | NORBIN [Rattus norvegicus] | gi\|2564013 | 79 | 50 | 17 | 2 | 7 | 43 |
| 523 | proteasome beta 3 subunit [Rattus norvegicus], gi\|730380\|sp\|P40112.1\|PSB3_RAT RecName: Full=Proteasome subunit beta type-3; AltName: Full=Proteasome theta chain; AltName: Full=Proteasome chain 13; AltName: Full=Proteasome component C10-II, gi\|458731\|dbj\|B | gi\|8394082 | 23 | 53 | 14 | 2 | 6 | 47 |
| 524 | NSFL1 (p97) cofactor (p47) [Rattus norvegicus], gi\|41017427\|sp\|O35987.1\|NSF1C_RAT RecName: Full=NSFL1 cofactor p47; AltName: Full=p97 cofactor p47; AltName: Full=XY body-associated protein XY40, gi\|2285790\|dbj\|BAA21659.1\| p47 [Rattus norvegicus], gi\|25470 | gi\|14010837 | 41 | 46 | 22 | 0 | 7 | 39 |
| 525 | RecName: Full=Beta-adducin; AltName: Full=Erythrocyte adducin subunit beta; AltName: Full=Adducin-63 | gi\|10720378 (+2) | 81 | 44 | 3 | 17 | 18 | 26 |
| 526 | eukaryotic translation initiation factor 3, subunit H [Rattus norvegicus], gi\|81885459\|sp\|Q6P9U8.1\|EIF3H_RAT RecName: Full=Eukaryotic translation initiation factor 3 subunit H; Short=eIF3h; AltName: Full=Eukaryotic translation initiation factor 3 subunit | gi\|38454242 | 40 | 46 | 0 | 14 | 14 | 32 |
| 527 | eukaryotic translation initiation factor 5 [Rattus norvegicus], gi\|585303\|sp\|Q07205.1\|IF5_RAT RecName: Full=Eukaryotic translation initiation factor 5; Short=eIF-5, gi\|294545\|gb\|AAA41112.1\| eukaryotic initiation factor 5, gi\|38382855\|gb\|AAH62398.1\| Eukary | gi\|9910214 | 49 | 47 | 9 | 13 | 16 | 31 |
| 528 | proteasome activator subunit 3 [Mus musculus], gi\|30410794\|ref\|NP_005780.2\| proteasome activator subunit 3 isoform 1 [Homo sapiens], gi\|47523754\|ref\|NP_999513.1\| proteasome activator subunit 3 [Sus scrofa], gi\|58865366\|ref\|NP_001011894.1\| proteasome (pros | gi\|6755214 | 30 | 51 | 10 | 6 | 9 | 42 |
| 529 | synaptosomal-associated protein 25 isoform SNAP25A [Homo sapiens], gi\|207080112\|ref\|NP_001128960.1\| DKFZP459L0820 protein [Pongo abelii], gi\|73991295\|ref\|XP_849677.1\| PREDICTED: similar to synaptosomal-associated protein 25 isoform SNAP25A isoform 3 [Cani | gi\|18765733 | 23 | 52 | 0 | 8 | 8 | 44 |
| 530 | exportin 7, isoform CRA_b [Rattus norvegicus] | gi\|149049916 | 124 | 48 | 9 | 13 | 16 | 32 |
| 531 | RNA (guanine-7-) methyltransferase [Rattus norvegicus], gi\|81883509\|sp\|Q5U2U7.1\|MCES_RAT RecName: Full=mRNA cap guanine-N7 methyltransferase; AltName: Full=mRNA (guanine-N(7)-)-methyltransferase; AltName: Full=RG7MT1; AltName: Full=mRNA cap methyltransfer | gi\|56605632 | 53 | 37 | 22 | 3 | 10 | 27 |
| 532 | coronin, actin binding protein 1C [Rattus norvegicus], gi\|149063636\|gb\|EDM13959.1\| similar to Coronin, actin binding protein 1C (predicted) [Rattus norvegicus] | gi\|157818697 | 53 | 46 | 13 | 4 | 8 | 38 |
| 533 | solute carrier family 25, member 4 [Rattus norvegicus], gi\|461475\|sp\|Q05962.3\|ADT1_RAT RecName: Full=ADP/ATP translocase 1; AltName: Full=Adenine nucleotide translocator 1; Short=ANT 1; AltName: Full=ADP,ATP carrier protein 1; AltName: Full=Solute carrier | gi\|32189355 (+1) | 33 | 57 | 5 | 6 | 8 | 49 |
| 534 | phospholipase A2, activating protein, isoform CRA_a [Rattus norvegicus] | gi\|149044496 (+1) | 92 | 43 | 6 | 11 | 13 | 30 |
| 535 | cysteinyl-tRNA synthetase (predicted), isoform CRA_a [Rattus norvegicus] | gi\|149061783 (+1) | 95 | 51 | 2 | 10 | 11 | 40 |
| 536 | immunoglobulin superfamily, member 3 [Rattus norvegicus], gi\|149030487\|gb\|EDL85524.1\| immunoglobulin superfamily, member 3 (predicted) [Rattus norvegicus] | gi\|158631164 | 120 | 63 | 0 | 6 | 6 | 57 |
| 537 | ras-related C3 botulinum toxin substrate 1 isoform Rac1 [Homo sapiens], gi\|27806443\|ref\|NP_776588.1\| ras-related C3 botulinum toxin substrate 1 [Bos taurus], gi\|45384330\|ref\|NP_990348.1\| ras-related C3 botulinum toxin substrate 1 (rho family, small GTP bi | gi\|9845511 | 21 | 42 | 18 | 0 | 6 | 36 |
| 538 | PREDICTED: similar to baculoviral IAP repeat-containing 6 [Rattus norvegicus] | gi\|109478992 (+1) | 547 | 37 | 3 | 15 | 16 | 21 |
| 539 | RecName: Full=Alpha-1-antiproteinase; AltName: Full=Alpha-1-antitrypsin; AltName: Full=Alpha-1-proteinase inhibitor; Flags: Precursor | gi\|112889 (+2) | 46 | 6 | 58 | 0 | 18 | 0 |
| 540 | RecName: Full=Neurolysin, mitochondrial; AltName: Full=Neurotensin endopeptidase; AltName: Full=Mitochondrial oligopeptidase M; AltName: Full=Microsomal endopeptidase; Short=MEP; Flags: Precursor, gi\|987087\|emb\|CAA60630.1\| neurolysin; neurotensin-degradin | gi\|1171691 (+2) | 80 | 42 | 15 | 2 | 7 | 35 |
| 541 | casein kinase 2, beta subunit, isoform CRA_b [Rattus norvegicus] | gi\|149028072 (+1) | 27 | 42 | 13 | 9 | 13 | 29 |
| 542 | PREDICTED: similar to SLIT-ROBO Rho GTPase-activating protein 3 (srGAP3) (srGAP2) (WAVE-associated Rac GTPase-activating protein) (WRP) (Rho-GTPase-activating protein 14) [Rattus norvegicus], gi\|109473966\|ref\|XP_001076245.1\| PREDICTED: similar to SLIT-ROB | gi\|62648014 | 124 | 50 | 9 | 8 | 11 | 39 |
| 543 | PREDICTED: similar to heterogeneous nuclear ribonucleoprotein U isoform a [Rattus norvegicus], gi\|109463488\|ref\|XP_001073417.1\| PREDICTED: similar to heterogeneous nuclear ribonucleoprotein U isoform a [Rattus norvegicus] | gi\|109459838 (+1) | 97 | 27 | 23 | 13 | 20 | 7 |
| 544 | cullin 2 [Rattus norvegicus], gi\|149032583\|gb\|EDL87461.1\| cullin 2 (predicted), isoform CRA_a [Rattus norvegicus] | gi\|157817342 | 85 | 46 | 7 | 12 | 14 | 32 |
| 545 | eukaryotic translation initiation factor 5A isoform B [Homo sapiens], gi\|31712036\|ref\|NP_853613.1\| eukaryotic translation initiation factor 5A [Mus musculus], gi\|51036244\|ref\|NP_001003658.1\| eukaryotic translation initiation factor 5A [Bos taurus], gi\|760 | gi\|4503545 | 17 | 53 | 15 | 0 | 5 | 48 |
| 546 | CDC16 cell division cycle 16 homolog [Rattus norvegicus], gi\|66911485\|gb\|AAH97498.1\| CDC16 cell division cycle 16 homolog (S. cerevisiae) [Rattus norvegicus] | gi\|67078528 | 71 | 47 | 2 | 8 | 9 | 38 |
| 547 | proteasome (prosome, macropain) subunit, alpha type 7 [Rattus norvegicus], gi\|149034051\|gb\|EDL88834.1\| rCG38543, isoform CRA_a [Rattus norvegicus] | gi\|56550075 | 28 | 56 | 10 | 0 | 3 | 53 |
| 548 | protein phosphatase 1, catalytic subunit, beta isoform 1 [Homo sapiens], gi\|6981388\|ref\|NP_037197.1\| protein phosphatase 1, catalytic subunit, beta [Rattus norvegicus], gi\|45384112\|ref\|NP_990453.1\| protein phosphatase 1, catalytic subunit, beta isoform [G | gi\|4506005 | 37 | 56 | 12 | 2 | 6 | 50 |
| 549 | rCG57686 [Rattus norvegicus] | gi\|149040327 | 65 | 49 | 6 | 5 | 7 | 42 |
| 550 | PREDICTED: similar to Nidogen-1 precursor (Entactin) [Rattus norvegicus] | gi\|109505096 | 137 | 51 | 0 | 11 | 11 | 40 |

| | | | | | | | | |
|---|---|---|---|---|---|---|---|---|
| 551 | cofilin 2, muscle [Mus musculus], gi\|1168994\|sp\|P45591.1\|COF2_MOUSE RecName: Full=Cofilin-2; AltName: Full=Cofilin, muscle isoform, gi\|498017\|gb\|AAA37433.1\| cofilin, gi\|13938044\|gb\|AAH07138.1\| Cofilin 2, muscle [Mus musculus], gi\|74151236\|dbj\|BAE27737.1\| | gi\|6671746 | 19 | 32 | 32 | 0 | 10 | 22 |
| 552 | RecName: Full=Matrin-3; AltName: Full=Nuclear scaffold protein p130/MAT3, gi\|2276402\|gb\|AAB63955.1\| matrin 3 [Rattus norvegicus] | gi\|12643398 (+1) | 94 | 43 | 4 | 11 | 12 | 31 |
| 553 | PREDICTED: similar to Filamin-C (Gamma-filamin) (Filamin-2) (Protein FLNc) (Actin-binding-like protein) (ABP-L) (ABP-280-like protein) isoform 3 [Rattus norvegicus] | gi\|109473203 (+1) | 287 | 25 | 33 | 5 | 15 | 10 |
| 554 | PREDICTED: similar to Ubiquitin carboxyl-terminal hydrolase 24 (Ubiquitin thiolesterase 24) (Ubiquitin-specific-processing protease 24) (Deubiquitinating enzyme 24) [Rattus norvegicus], gi\|109476934\|ref\|XP_001064937.1\| PREDICTED: similar to Ubiquitin carb | gi\|109475211 | 235 | 36 | 13 | 12 | 16 | 20 |
| 555 | nuclear receptor binding protein [Rattus norvegicus], gi\|74356247\|gb\|AAI04695.1\| Nuclear receptor binding protein [Rattus norvegicus], gi\|149050746\|gb\|EDM02919.1\| nuclear receptor binding protein [Rattus norvegicus] | gi\|78042609 | 60 | 31 | 16 | 15 | 20 | 11 |
| 556 | PREDICTED: similar to Hook-related protein 1 [Rattus norvegicus] | gi\|109501126 | 212 | 28 | 23 | 3 | 10 | 18 |
| 557 | ADP-ribosylation factor-like 2 [Rattus norvegicus], gi\|3182914\|sp\|O08697.1\|ARL2_RAT RecName: Full=ADP-ribosylation factor-like protein 2, gi\|2062133\|emb\|CAA73245.1\| ADP-ribosylation factor-like protein [Rattus norvegicus] | gi\|13928996 (+1) | 21 | 41 | 25 | 0 | 8 | 33 |
| 558 | anaphase-promoting complex subunit 5 (predicted) [Rattus norvegicus] | gi\|149063341 | 83 | 37 | 4 | 18 | 19 | 18 |
| 559 | UDP-glucose pyrophosphorylase 2 [Rattus norvegicus], gi\|66910575\|gb\|AAH97369.1\| UDP-glucose pyrophosphorylase 2 [Rattus norvegicus], gi\|149044770\|gb\|EDL97956.1\| UDP-glucose pyrophosphorylase 2, isoform CRA_b [Rattus norvegicus] | gi\|67078526 | 57 | 55 | 5 | 2 | 4 | 51 |
| 560 | RAB5B, member RAS oncogene family [Rattus norvegicus], gi\|120537414\|gb\|AAI29102.1\| RAB5B, member RAS oncogene family [Rattus norvegicus], gi\|149029654\|gb\|EDL84825.1\| RAB5B, member RAS oncogene family (predicted), isoform CRA_c [Rattus norvegicus] | gi\|121583768 | 24 | 50 | 15 | 4 | 9 | 41 |
| 561 | glutathione transferase zeta 1 [Rattus norvegicus], gi\|149025260\|gb\|EDL81627.1\| rCG20683, isoform CRA_b [Rattus norvegicus], gi\|165971039\|gb\|AAI58834.1\| Glutathione transferase zeta 1 [Rattus norvegicus], gi\|208969735\|gb\|ACI32127.1\| glutathione S-transfer | gi\|157822229 | 24 | 41 | 20 | 7 | 13 | 28 |
| 562 | calcium channel, voltage-dependent, alpha2/delta subunit 1 isoform 2 [Rattus norvegicus], gi\|27450704\|gb\|AAO14652.1\|AF486276_1 calcium channel alpha-2 delta-1 subunit isoform e [Rattus norvegicus] | gi\|161086906 (+1) | 123 | 61 | 0 | 5 | 5 | 56 |
| 563 | N-ethylmaleimide-sensitive factor attachment protein, alpha [Rattus norvegicus], gi\|6094309\|sp\|P54921.2\|SNAA_RAT RecName: Full=Alpha-soluble NSF attachment protein; Short=SNAP-alpha; AltName: Full=N-ethylmaleimide-sensitive factor attachment protein alpha | gi\|18034791 | 33 | 52 | 2 | 8 | 9 | 43 |
| 564 | proteasome (prosome, macropain) subunit, beta type 7 [Rattus norvegicus], gi\|17380238\|sp\|Q9JHW0.1\|PSB7_RAT RecName: Full=Proteasome subunit beta type-7; AltName: Full=Proteasome subunit Z; AltName: Full=Macropain chain Z; AltName: Full=Multicatalytic endo | gi\|16758298 | 30 | 56 | 9 | 3 | 6 | 50 |
| 565 | rCG26466, isoform CRA_b [Rattus norvegicus] | gi\|149023210 | 143 | 44 | 0 | 10 | 10 | 34 |
| 566 | adaptor protein complex AP-2, mu1 [Mus musculus], gi\|14917109\|ref\|NP_004059.2\| adaptor-related protein complex 2, mu 1 subunit isoform a [Homo sapiens], gi\|16758938\|ref\|NP_446289.1\| adaptor-related protein complex 2, mu 1 subunit [Rattus norvegicus], gi\|7 | gi\|6753074 | 50 | 48 | 6 | 6 | 8 | 40 |
| 567 | lysophospholipase 2 [Rattus norvegicus], gi\|41017253\|sp\|Q9QYL8.1\|LYPA2_RAT RecName: Full=Acyl-protein thioesterase 2; AltName: Full=Lysophospholipase II; AltName: Full=Lysophospholipase 2, gi\|6518521\|dbj\|BAA87911.1\| lysophospholipase II [Rattus norvegicus | gi\|13786178 | 25 | 23 | 28 | 4 | 13 | 10 |
| 568 | laminin, gamma 1 [Rattus norvegicus] | gi\|149058392 (+1) | 171 | 59 | 0 | 7 | 7 | 52 |
| 569 | aldo-keto reductase family 1, member B1 [Rattus norvegicus], gi\|1168407\|sp\|P07943.3\|ALDR_RAT RecName: Full=Aldose reductase; Short=AR; AltName: Full=Aldehyde reductase, gi\|55759\|emb\|CAA29308.1\| unnamed protein product [Rattus norvegicus], gi\|202852\|gb\|AAA | gi\|6978491 | 36 | 32 | 20 | 0 | 6 | 26 |
| 570 | ARP1 actin-related protein 1 homolog B, centractin beta [Mus musculus], gi\|166157502\|ref\|NP_001034117.2\| ARP1 actin-related protein 1 homolog B [Rattus norvegicus], gi\|47115849\|sp\|Q8R5C5.1\|ACTY_MOUSE RecName: Full=Beta-centractin; AltName: Full=Actin-rela | gi\|22122615 | 42 | 47 | 0 | 13 | 13 | 34 |
| 571 | RAN binding protein 1 [Rattus norvegicus], gi\|149019779\|gb\|EDL77927.1\| rCG36598 [Rattus norvegicus] | gi\|157820113 | 24 | 34 | 12 | 0 | 4 | 30 |
| 572 | small glutamine-rich tetratricopeptide repeat (TPR)-containing, alpha [Rattus norvegicus], gi\|8134664\|sp\|O70593.1\|SGTA_RAT RecName: Full=Small glutamine-rich tetratricopeptide repeat-containing protein alpha; AltName: Full=Alpha-SGT; AltName: Full=Small g | gi\|12083667 | 34 | 43 | 24 | 0 | 8 | 35 |
| 573 | eukaryotic translation elongation factor 1 epsilon 1 [Rattus norvegicus], gi\|149045165\|gb\|EDL98251.1\| rCG43901 [Rattus norvegicus], gi\|187469479\|gb\|AAI66841.1\| Eukaryotic translation elongation factor 1 epsilon 1 [Rattus norvegicus] | gi\|157816879 | 20 | 54 | 3 | 0 | 1 | 53 |
| 574 | anaphase promoting complex subunit 4 [Rattus norvegicus], gi\|149047211\|gb\|EDL99880.1\| anaphase promoting complex subunit 4, isoform CRA_a [Rattus norvegicus] | gi\|157821679 | 92 | 49 | 0 | 9 | 9 | 40 |
| 575 | ADP-ribosylation factor-like 1 [Rattus norvegicus], gi\|153792526\|ref\|NP_080135.2\| ADP-ribosylation factor-like 1 [Mus musculus], gi\|47117641\|sp\|P61212.1\|ARL1_RAT RecName: Full=ADP-ribosylation factor-like protein 1, gi\|47117662\|sp\|P61211.1\|ARL1_MOUSE RecN | gi\|11693150 (+2) | 20 | 41 | 21 | 0 | 7 | 34 |
| 576 | Rab6 interacting protein 2, isoform CRA_c [Rattus norvegicus] | gi\|149049598 | 125 | 48 | 5 | 1 | 3 | 45 |
| 577 | glycoprotein m6a [Rattus norvegicus], gi\|28201133\|dbj\|BAC56699.1\| glycoprotein m6a [Rattus norvegicus], gi\|57032815\|gb\|AAH88862.1\| Glycoprotein m6a [Rattus norvegicus], gi\|149021493\|gb\|EDL78956.1\| glycoprotein m6a, isoform CRA_b [Rattus norvegicus] | gi\|30017437 | 31 | 56 | 6 | 0 | 2 | 54 |
| 578 | vacuolar protein sorting 53 homolog [Rattus norvegicus], gi\|149053432\|gb\|EDM05249.1\| vacuolar protein sorting 53 (yeast) (predicted), isoform CRA_a [Rattus norvegicus] | gi\|157786652 | 94 | 46 | 0 | 13 | 13 | 33 |
| 579 | Vdac1 protein [Rattus norvegicus] | gi\|38051979 (+1) | 32 | 49 | 2 | 6 | 7 | 42 |
| 580 | phenylalanyl-tRNA synthetase, alpha subunit [Rattus norvegicus], gi\|81887353\|sp\|Q505J8.1\|SYFA_RAT RecName: Full=Phenylalanyl-tRNA synthetase alpha chain; AltName: Full=Phenylalanine--tRNA ligase alpha chain; Short=PheRS, gi\|63101487\|gb\|AAH94515.1\| Phenyla | gi\|66730413 | 58 | 37 | 0 | 19 | 19 | 18 |
| 581 | hemoglobin alpha 2 chain [Rattus norvegicus], gi\|1304381\|gb\|AAA99054.1\| hemoglobin alpha chain, gi\|3367720\|emb\|CAA46476.1\| alpha-2-globin chain [Rattus norvegicus], gi\|3367722\|emb\|CAA39764.1\| 2-alpha globin [Rattus norvegicus], gi\|60688619\|gb\|AAH91567.1\| | gi\|60678292 (+1) | 15 | 62 | 1 | 0 | 0 | 62 |
| 582 | transportin 3 [Rattus norvegicus], gi\|149065154\|gb\|EDM15230.1\| transportin 3 [Rattus norvegicus] | gi\|157819279 | 104 | 30 | 16 | 6 | 11 | 19 |
| 583 | WAS protein family, member 1 [Rattus norvegicus], gi\|81882530\|sp\|Q5BJU7.1\|WASF1_RAT RecName: Full=Wiskott-Aldrich syndrome protein family member 1; Short=WASP family protein member 1; AltName: Full=Protein WAVE-1, gi\|60551504\|gb\|AAH91322.1\| WAS protein fa | gi\|68341973 | 62 | 50 | 1 | 9 | 9 | 41 |
| 584 | rCG59652, isoform CRA_b [Rattus norvegicus] | gi\|149065835 | 63 | 32 | 31 | 1 | 11 | 21 |
| 585 | spermidine synthase [Rattus norvegicus], gi\|13384156\|gb\|AAK21288.1\|AF337636_1 spermidine synthase [Rattus norvegicus], gi\|118763718\|gb\|AAI28734.1\| Spermidine synthase [Rattus norvegicus] | gi\|16758208 | 34 | 23 | 31 | 2 | 12 | 11 |
| 586 | aminopeptidase-B [Rattus norvegicus] | gi\|1754515 (+1) | 73 | 45 | 15 | 0 | 5 | 40 |
| 587 | DEAD (Asp-Glu-Ala-Asp) box polypeptide 1 [Rattus norvegicus], gi\|76364171\|sp\|Q641Y8.1\|DDX1_RAT RecName: Full=ATP-dependent RNA helicase DDX1; AltName: Full=DEAD box protein 1, gi\|51980333\|gb\|AAH82049.1\| DEAD (Asp-Glu-Ala-Asp) box polypeptide 1 [Rattus nor | gi\|61097941 | 82 | 33 | 2 | 15 | 16 | 17 |
| 588 | ribosomal protein S7 [Homo sapiens], gi\|6755376\|ref\|NP_035430.1\| ribosomal protein S7 [Mus musculus], gi\|57618938\|ref\|NP_001009832.1\| ribosomal protein S7 [Felis catus], gi\|149642623\|ref\|NP_001092344.1\| ribosomal protein S7 [Bos taurus], gi\|201862122\|ref\| | gi\|4506741 | 22 | 44 | 0 | 8 | 8 | 36 |
| 589 | prenylcysteine oxidase 1 [Rattus norvegicus], gi\|62286984\|sp\|Q99ML5.1\|PCYOX_RAT RecName: Full=Prenylcysteine oxidase; AltName: Full=Chloride ion pump-associated 55 kDa protein; Flags: Precursor, gi\|13242075\|gb\|AAK16548.1\|AF332142_1 chloride ion pump-assoc | gi\|21489987 | 56 | 62 | 0 | 0 | 0 | 62 |
| 590 | SEC13 homolog [Rattus norvegicus], gi\|81910340\|sp\|Q5XFW8.1\|SEC13_RAT RecName: Full=Protein SEC13 homolog; AltName: Full=SEC13-like protein 1, gi\|54261633\|gb\|AAH84705.1\| SEC13 homolog (S. cerevisiae) [Rattus norvegicus], gi\|149036942\|gb\|EDL91560.1\| SEC13-l | gi\|55741774 | 36 | 33 | 11 | 12 | 15 | 18 |
| 591 | poly(A) binding protein, cytoplasmic 1 [Rattus norvegicus], gi\|47605941\|sp\|Q9EPH8.1\|PABP1_RAT RecName: Full=Polyadenylate-binding protein 1; Short=Poly(A)-binding protein 1; Short=PABP 1, gi\|12188891\|emb\|CAC21554.1\| poly(A) binding protein [Rattus norvegi | gi\|19705459 | 71 | 52 | 4 | 6 | 7 | 45 |
| 592 | RecName: Full=UMP-CMP kinase; AltName: Full=Cytidylate kinase; AltName: Full=Deoxycytidylate kinase; AltName: Full=Cytidine monophosphate kinase; AltName: Full=Uridine monophosphate kinase; AltName: Full=Uridine monophosphate/cytidine monophosphate kinase | gi\|150383503 | 22 | 36 | 21 | 0 | 7 | 29 |
| 593 | glypican 4 [Rattus norvegicus], gi\|51980590\|gb\|AAH81962.1\| Glypican 4 [Rattus norvegicus] | gi\|62078949 | 63 | 53 | 0 | 5 | 5 | 48 |
| 594 | ezrin [Rattus norvegicus], gi\|68067388\|sp\|P31977.3\|EZRI_RAT RecName: Full=Ezrin; AltName: Full=p81; AltName: Full=Cytovillin; AltName: Full=Villin-2, gi\|51858695\|gb\|AAH81958.1\| Ezrin [Rattus norvegicus], gi\|149028296\|gb\|EDL83712.1\| villin 2 [Rattus norveg | gi\|52138521 | 69 | 44 | 12 | 0 | 4 | 40 |
| 595 | PREDICTED: similar to Ubiquitin-protein ligase EDD1 (Hyperplastic discs protein homolog) [Rattus norvegicus] | gi\|109480712 (+1) | 307 | 43 | 1 | 13 | 13 | 30 |
| 596 | PREDICTED: similar to talin 2 isoform 2 [Rattus norvegicus] | gi\|109484980 (+2) | 272 | 33 | 14 | 7 | 11 | 22 |
| 597 | acidic ribosomal phosphoprotein P0 [Rattus norvegicus], gi\|730581\|sp\|P19945.2\|RLA0_RAT RecName: Full=60S acidic ribosomal protein P0; AltName: Full=L10E, gi\|450370\|emb\|CAA82647.1\| acidic ribosomal protein P0 [Rattus norvegicus], gi\|38541406\|gb\|AAH62028.1\| | gi\|11693176 | 34 | 35 | 10 | 16 | 19 | 16 |
| 598 | biliverdin reductase A [Rattus norvegicus], gi\|1168674\|sp\|P46844.1\|BIEA_RAT RecName: Full=Biliverdin reductase A; Short=BVR A; AltName: Full=Biliverdin-IX alpha-reductase; Flags: Precursor, gi\|13096101\|pdb\|1GCU\|A Chain A, Crystal Structure Of Rat Biliverd | gi\|16758714 (+1) | 34 | 27 | 22 | 6 | 13 | 14 |
| 599 | amphiphysin [Rattus norvegicus], gi\|14916529\|sp\|O08838.1\|AMPH_RAT RecName: Full=Amphiphysin, gi\|2145467\|emb\|CAA73808.1\| amphiphysin [Rattus norvegicus] | gi\|11560002 | 75 | 48 | 2 | 10 | 11 | 37 |
| 600 | dynein cytoplasmic 1 intermediate chain 1, isoform CRA_a [Rattus norvegicus] | gi\|149064950 (+2) | 71 | 45 | 2 | 10 | 11 | 34 |
| 601 | PREDICTED: similar to Heterogeneous nuclear ribonucleoprotein L-like [Rattus norvegicus], gi\|109478941\|ref\|XP_001063027.1\| PREDICTED: similar to Heterogeneous nuclear ribonucleoprotein L-like [Rattus norvegicus] | gi\|109477851 | 85 | 36 | 3 | 14 | 15 | 21 |
| 602 | nuclear distribution gene C homolog [Rattus norvegicus], gi\|62286960\|sp\|Q63525.1\|NUDC_RAT RecName: Full=Nuclear migration protein nudC; AltName: Full=Nuclear distribution protein C homolog; AltName: Full=c15, gi\|619907\|emb\|CAA57825.1\| RnudC [Rattus norveg | gi\|8394272 | 38 | 37 | 28 | 1 | 10 | 27 |
| 603 | enolase 2, gamma, neuronal [Rattus norvegicus], gi\|119349\|sp\|P07323.2\|ENOG_RAT RecName: Full=Gamma-enolase; AltName: Full=2-phospho-D-glycerate hydro-lyase; AltName: Full=Neural enolase; AltName: Full=Neuron-specific enolase; Short=NSE; AltName: Full=Enol | gi\|26023949 | 47 | 51 | 8 | 0 | 3 | 48 |

| | | | | | | | | |
|---|---|---|---|---|---|---|---|---|---|
| 604 | karyopherin alpha 1 (importin alpha 5) [Rattus norvegicus], gi\|59800315\|gb\|AAX07452.1\| karyopherin alpha 1 [Rattus norvegicus], gi\|149060584\|gb\|EDM11298.1\| karyopherin (importin) alpha 1 [Rattus norvegicus] | gi\|148747406 (+1) | 60 | 47 | 6 | 4 | 6 | 41 |
| 605 | G protein pathway suppressor 1 [Mus musculus], gi\|42476092\|ref\|NP_446421.2\| G protein pathway suppressor 1 [Rattus norvegicus], gi\|47116574\|sp\|Q99LD4.1\|CSN1_MOUSE RecName: Full=COP9 signalosome complex subunit 1; Short=Signalosome subunit 1; Short=SGN1; A | gi\|21703742 (+1) | 53 | 32 | 9 | 13 | 16 | 16 |
| 606 | CLIP associating protein 2 [Rattus norvegicus], gi\|77416394\|sp\|Q99JD4.1\|CLAP2_RAT RecName: Full=CLIP-associating protein 2; AltName: Full=Cytoplasmic linker-associated protein 2, gi\|13508651\|emb\|CAC35166.1\| CLIP-associating protein CLASP2 [Rattus norvegic | gi\|16758540 | 141 | 48 | 4 | 5 | 6 | 42 |
| 607 | calcyclin binding protein [Rattus norvegicus], gi\|81884615\|sp\|Q6AYK6.1\|CYBP_RAT RecName: Full=Calcyclin-binding protein; Short=CacyBP, gi\|50926902\|gb\|AAH79007.1\| Calcyclin binding protein [Rattus norvegicus], gi\|149058281\|gb\|EDM09438.1\| calcyclin binding | gi\|51948388 | 27 | 31 | 24 | 0 | 8 | 23 |
| 608 | amyloid beta (A4) precursor protein [Rattus norvegicus], gi\|112930\|sp\|P08592.2\|A4_RAT RecName: Full=Amyloid beta A4 protein; AltName: Full=Alzheimer disease amyloid A4 protein homolog; AltName: Full=ABPP; Short=APP; AltName: Full=Amyloidogenic glycoprotei | gi\|27436861 (+1) | 87 | 42 | 5 | 5 | 7 | 35 |
| 609 | PREDICTED: similar to brefeldin A-inhibited guanine nucleotide-exchange protein 1 [Rattus norvegicus], gi\|109476047\|ref\|XP_001066125.1\| PREDICTED: similar to brefeldin A-inhibited guanine nucleotide-exchange protein 1 [Rattus norvegicus] | gi\|109474575 (+1) | 219 | 30 | 16 | 6 | 11 | 19 |
| 610 | glutathione S-transferase, pi [Rattus norvegicus], gi\|121749\|sp\|P04906.2\|GSTP1_RAT RecName: Full=Glutathione S-transferase P; AltName: Full=GST 7-7; AltName: Full=Chain 7; AltName: Full=GST class-pi, gi\|56336\|emb\|CAA26664.1\| unnamed protein product [Rattu | gi\|25453420 | 23 | 24 | 27 | 3 | 12 | 12 |
| 611 | arsA arsenite transporter, ATP-binding, homolog 1 [Mus musculus], gi\|213512072\|ref\|NP_001093975.1\| arsA arsenite transporter, ATP-binding, homolog 1 [Rattus norvegicus], gi\|14916955\|sp\|O54984.2\|ARSA1_MOUSE RecName: Full=Arsenical pump-driving ATPase; AltN | gi\|12025542 | 39 | 38 | 3 | 19 | 20 | 18 |
| 612 | kinesin family member 21A (predicted), isoform CRA_b [Rattus norvegicus] | gi\|149017592 (+2) | 139 | 40 | 11 | 5 | 8 | 32 |
| 613 | karyopherin (importin) alpha 3 [Mus musculus], gi\|62339392\|ref\|NP_001014792.1\| karyopherin (importin) alpha 3 [Rattus norvegicus], gi\|119904523\|ref\|XP_612814.3\| PREDICTED: similar to importin alpha Q2 [Bos taurus], gi\|3122277\|sp\|O35344.1\|IMA3_MOUSE RecNam | gi\|6680596 (+1) | 58 | 46 | 3 | 3 | 4 | 42 |
| 614 | RecName: Full=Haloacid dehalogenase-like hydrolase domain-containing protein 2, gi\|50925547\|gb\|AAH78941.1\| Haloacid dehalogenase-like hydrolase domain containing 2 [Rattus norvegicus], gi\|149029509\|gb\|EDL84723.1\| rCG41187, isoform CRA_a [Rattus norvegicus | gi\|81884645 | 29 | 36 | 14 | 6 | 10 | 26 |
| 615 | tyrosyl-tRNA synthetase [Rattus norvegicus] | gi\|149024033 (+1) | 63 | 45 | 6 | 10 | 12 | 33 |
| 616 | Fas-associated factor 1 [Rattus norvegicus] | gi\|149035673 | 74 | 33 | 18 | 5 | 11 | 22 |
| 617 | hypothetical protein LOC303238 [Rattus norvegicus], gi\|149053015\|gb\|EDM04832.1\| similar to novel protein (predicted), isoform CRA_b [Rattus norvegicus] | gi\|157822211 | 134 | 40 | 8 | 6 | 9 | 31 |
| 618 | thioredoxin domain containing 1 [Rattus norvegicus], gi\|62948112\|gb\|AAH94308.1\| Thioredoxin domain containing 1 [Rattus norvegicus], gi\|149051378\|gb\|EDM03551.1\| thioredoxin domain containing 1 [Rattus norvegicus] | gi\|67078422 | 31 | 44 | 4 | 1 | 2 | 42 |
| 619 | dynactin 4 [Rattus norvegicus], gi\|62899905\|sp\|Q9QUR2.1\|DCTN4_RAT RecName: Full=Dynactin subunit 4; AltName: Full=Dynactin subunit p62, gi\|6090977\|gb\|AAF03422.1\|AF192494_1 p62 dynactin subunit isoform 2 [Rattus norvegicus], gi\|6176556\|gb\|AAF05618.1\|AF1907 | gi\|16758134 | 53 | 46 | 0 | 13 | 13 | 33 |
| 620 | SAR1a gene homolog 2 [Rattus norvegicus], gi\|81889008\|sp\|Q5HZY2.1\|SAR1B_RAT RecName: Full=GTP-binding protein SAR1b, gi\|57032799\|gb\|AAH88842.1\| SAR1 homolog B (S. cerevisiae) [Rattus norvegicus], gi\|149052515\|gb\|EDM04332.1\| SAR1 gene homolog B (S. cerevis | gi\|57528164 | 22 | 37 | 13 | 0 | 4 | 33 |
| 621 | septin 7, isoform CRA_b [Rattus norvegicus] | gi\|149027897 (+2) | 51 | 48 | 2 | 4 | 5 | 43 |
| 622 | ganglioside-induced differentiation-associated protein 1-like 1 [Rattus norvegicus], gi\|149043007\|gb\|EDL96581.1\| ganglioside-induced differentiation-associated protein 1-like 1 (predicted) [Rattus norvegicus], gi\|195540220\|gb\|AAI68159.1\| Gdap1l1 protein [ | gi\|157821573 | 42 | 29 | 13 | 9 | 13 | 16 |
| 623 | ATPase, H+ transporting, lysosomal V1 subunit C1 [Rattus norvegicus], gi\|81882798\|sp\|Q5FVI6.1\|VATC1_RAT RecName: Full=V-type proton ATPase subunit C 1; Short=V-ATPase subunit C 1; AltName: Full=Vacuolar proton pump subunit C 1, gi\|58476568\|gb\|AAH89961.1\| | gi\|58865560 | 44 | 53 | 3 | 2 | 3 | 50 |
| 624 | RecName: Full=Serine/threonine-protein kinase PAK 2; AltName: Full=p21-activated kinase 2; Short=PAK-2; AltName: Full=Gamma-PAK; Contains: RecName: Full=PAK-2p27; Contains: RecName: Full=PAK-2p34, gi\|1016005\|gb\|AAA79064.1\| serine/threonine kinase, gi\|1245 | gi\|2499648 | 58 | 28 | 31 | 1 | 11 | 17 |
| 625 | RecName: Full=Pyridoxal phosphate phosphatase; Short=PLP phosphatase; AltName: Full=Reg I-binding protein 1 | gi\|85541051 | 33 | 41 | 18 | 0 | 6 | 35 |
| 626 | guanine nucleotide binding protein, alpha 11 [Rattus norvegicus], gi\|12585229\|sp\|Q9JID2.1\|GNA11_RAT RecName: Full=Guanine nucleotide-binding protein subunit alpha-11; Short=G-protein subunit alpha-11; Short=G alpha-11, gi\|8925964\|gb\|AAF81690.1\|AF239674_1 | gi\|13591951 (+1) | 42 | 42 | 0 | 16 | 16 | 26 |
| 627 | TIP41, TOR signaling pathway regulator-like [Rattus norvegicus], gi\|158512831\|sp\|A2VCX1.1\|TIPRL_RAT RecName: Full=TIP41-like protein, gi\|124504345\|gb\|AAI28781.1\| TIP41, TOR signaling pathway regulator-like (S. cerevisiae) [Rattus norvegicus] | gi\|198278573 | 31 | 27 | 30 | 0 | 9 | 18 |
| 628 | capping protein (actin filament) muscle Z-line, beta, isoform CRA_c [Rattus norvegicus] | gi\|149024411 (+2) | 28 | 34 | 11 | 7 | 10 | 24 |
| 629 | leukotriene A4 hydrolase [Rattus norvegicus], gi\|71122467\|gb\|AAH99819.1\| Leukotriene A4 hydrolase [Rattus norvegicus] | gi\|71795621 | 69 | 24 | 28 | 8 | 17 | 7 |
| 630 | similar to RIKEN cDNA 1110059P08 [Rattus norvegicus] | gi\|149039583 | 34 | 33 | 8 | 14 | 17 | 16 |
| 631 | rCG58358, isoform CRA_a [Rattus norvegicus] | gi\|149041694 | 320 | 35 | 2 | 17 | 18 | 17 |
| 632 | rCG45997 [Rattus norvegicus] | gi\|149058446 (+1) | 38 | 37 | 0 | 20 | 20 | 17 |
| 633 | HIV-1 Tat specific factor 1 [Rattus norvegicus], gi\|149015808\|gb\|EDL75132.1\| HIV TAT specific factor 1 (predicted) [Rattus norvegicus] | gi\|157818333 | 89 | 23 | 10 | 17 | 20 | 3 |
| 634 | hypothetical protein LOC302247 [Rattus norvegicus], gi\|45478224\|gb\|AAS66283.1\| LRRGT00192 [Rattus norvegicus] | gi\|114145722 (+1) | 34 | 43 | 4 | 10 | 11 | 32 |
| 635 | DnaJ (Hsp40) homolog, subfamily A, member 2 [Mus musculus], gi\|56799412\|ref\|NP_114468.2\| DnaJ (Hsp40) homolog, subfamily A, member 2 [Rattus norvegicus], gi\|14916553\|sp\|Q9QYJ0.1\|DNJA2_MOUSE RecName: Full=DnaJ homolog subfamily A member 2; Short=mDj3, gi\|6 | gi\|9789937 | 46 | 31 | 4 | 14 | 15 | 16 |
| 636 | rCG62728, isoform CRA_b [Rattus norvegicus] | gi\|149048221 (+1) | 27 | 44 | 0 | 9 | 9 | 35 |
| 637 | NADPH-cytochrome P-450 oxidoreductase | gi\|205660 | 77 | 44 | 0 | 8 | 8 | 36 |
| 638 | 3'-phosphoadenosine 5'-phosphosulfate synthase 1 [Rattus norvegicus], gi\|149025974\|gb\|EDL82217.1\| 3'-phosphoadenosine 5'-phosphosulfate synthase 1 (predicted) [Rattus norvegicus] | gi\|157823805 | 66 | 45 | 3 | 11 | 12 | 33 |
| 639 | CTP synthase [Rattus norvegicus], gi\|149023839\|gb\|EDL80336.1\| rCG30713, isoform CRA_a [Rattus norvegicus], gi\|149023840\|gb\|EDL80337.1\| rCG30713, isoform CRA_a [Rattus norvegicus], gi\|171846764\|gb\|AAI61954.1\| Ctps protein [Rattus norvegicus] | gi\|199560247 | 67 | 42 | 2 | 10 | 11 | 31 |
| 640 | hypothetical protein LOC684352 [Rattus norvegicus], gi\|149018687\|gb\|EDL77328.1\| protein tyrosine kinase 9-like (A6-related protein) (predicted), isoform CRA_b [Rattus norvegicus], gi\|165971615\|gb\|AAI58615.1\| Ptk9l protein [Rattus norvegicus] | gi\|207028435 | 39 | 38 | 3 | 15 | 16 | 22 |
| 641 | microtubule-associated protein 4 [Rattus norvegicus] | gi\|149018456 (+1) | 117 | 29 | 4 | 10 | 11 | 18 |
| 642 | leucine aminopeptidase 3 [Rattus norvegicus], gi\|81884348\|sp\|Q68FS4.1\|AMPL_RAT RecName: Full=Cytosol aminopeptidase; AltName: Full=Leucine aminopeptidase; AltName: Full=Leucyl aminopeptidase; AltName: Full=Leucine aminopeptidase 3; Short=LAP; AltName: Ful | gi\|58865398 | 56 | 34 | 3 | 14 | 15 | 19 |
| 643 | twinfilin, actin-binding protein, homolog 1 [Rattus norvegicus], gi\|81883342\|sp\|Q5RJR2.1\|TWF1_RAT RecName: Full=Twinfilin-1, gi\|55824728\|gb\|AAH86536.1\| Twinfilin, actin-binding protein, homolog 1 (Drosophila) [Rattus norvegicus], gi\|149017641\|gb\|EDL76645. | gi\|56606100 | 40 | 29 | 23 | 2 | 9 | 20 |
| 644 | RecName: Full=Protein disulfide-isomerase; Short=PDI; AltName: Full=Prolyl 4-hydroxylase subunit beta; AltName: Full=Cellular thyroid hormone-binding protein; Flags: Precursor, gi\|38197382\|gb\|AAH61857.1\| Prolyl 4-hydroxylase, beta polypeptide [Rattus norv | gi\|129731 (+1) | 57 | 41 | 2 | 4 | 5 | 36 |
| 645 | calreticulin [Rattus norvegicus], gi\|117505\|sp\|P18418.1\|CALR_RAT RecName: Full=Calreticulin; AltName: Full=CRP55; AltName: Full=Calregulin; AltName: Full=HACBP; AltName: Full=ERp60; AltName: Full=CALBP; AltName: Full=Calcium-binding protein 3; Short=CABP3 | gi\|11693172 | 48 | 39 | 2 | 2 | 3 | 36 |
| 646 | ubiquitin-conjugating enzyme E2-25K isoform 1 [Homo sapiens], gi\|31542956\|ref\|NP_058066.2\| ubiquitin-conjugating enzyme E2-25K [Mus musculus], gi\|118403852\|ref\|NP_001072139.1\| UBC1 [Sus scrofa], gi\|157822205\|ref\|NP_001099476.1\| ubiquitin-conjugating enzym | gi\|4885417 | 22 | 24 | 30 | 0 | 9 | 15 |
| 647 | archain [Rattus norvegicus], gi\|81884175\|sp\|Q66H80.1\|COPD_RAT RecName: Full=Coatomer subunit delta; AltName: Full=Delta-coat protein; Short=Delta-COP; Short=Archain, gi\|51858705\|gb\|AAH81979.1\| Archain 1 [Rattus norvegicus], gi\|149041491\|gb\|EDL95332.1\| arc | gi\|56090634 | 57 | 31 | 0 | 10 | 10 | 21 |
| 648 | annexin A2 [Rattus norvegicus], gi\|584760\|sp\|Q07936.2\|ANXA2_RAT RecName: Full=Annexin A2; AltName: Full=Annexin-2; AltName: Full=Annexin II; AltName: Full=Lipocortin II; AltName: Full=Calpactin I heavy chain; AltName: Full=Chromobindin-8; AltName: Full=p3 | gi\|9845234 | 39 | 54 | 0 | 0 | 0 | 54 |
| 649 | rCG35464, isoform CRA_b [Rattus norvegicus] | gi\|149054189 (+1) | 47 | 49 | 3 | 0 | 1 | 48 |
| 650 | regulation of nuclear pre-mRNA domain containing 1B [Rattus norvegicus], gi\|23813892\|sp\|Q9CSU0.2\|RPR1B_MOUSE RecName: Full=Regulation of nuclear pre-mRNA domain-containing protein 1B; AltName: Full=Cell cycle-related and expression-elevated protein in tum | gi\|149158731 (+1) | 37 | 30 | 10 | 13 | 16 | 14 |
| 651 | bridging integrator 1 [Rattus norvegicus], gi\|14916534\|sp\|O08839.1\|BIN1_RAT RecName: Full=Myc box-dependent-interacting protein 1; AltName: Full=Bridging integrator 1; AltName: Full=Amphiphysin-like protein; Short=Amphiphysin II, gi\|2145469\|emb\|CAA73807.1 | gi\|16758846 | 65 | 42 | 5 | 7 | 9 | 33 |
| 652 | nuclear protein localization 4 [Rattus norvegicus], gi\|48429023\|sp\|Q9ES54.3\|NPL4_RAT RecName: Full=Nuclear protein localization protein 4 homolog; Short=Protein NPL4, gi\|11037252\|gb\|AAG27534.1\|AF234600_1 NPL4 [Rattus norvegicus], gi\|74353675\|gb\|AAI01888.1 | gi\|18034779 | 68 | 39 | 2 | 9 | 10 | 29 |
| 653 | prostaglandin-E synthase 3 [Mus musculus], gi\|195976800\|ref\|NP_001124461.1\| prostaglandin-E synthase 3 [Rattus norvegicus], gi\|149255313\|ref\|XP_001480119.1\| PREDICTED: similar to Sid3177p [Mus musculus], gi\|149255697\|ref\|XP_001478909.1\| PREDICTED: similar | gi\|9790017 | 19 | 34 | 15 | 0 | 5 | 29 |
| 654 | delta-aminolevulinic acid dehydratase [Rattus norvegicus], gi\|122836\|sp\|P06214.1\|HEM2_RAT RecName: Full=Delta-aminolevulinic acid dehydratase; Short=ALADH; AltName: Full=Porphobilinogen synthase, gi\|55626\|emb\|CAA28621.1\| unnamed protein product [Rattus no | gi\|6978483 | 36 | 36 | 20 | 0 | 6 | 30 |
| 655 | alpha-2-macroglobulin, isoform CRA_a [Rattus norvegicus] | gi\|149049553 (+1) | 163 | 11 | 42 | 0 | 13 | 0 |
| 656 | drebrin-like [Rattus norvegicus], gi\|7248377\|dbj\|BAA92708.1\| SH3P7r2 [Rattus norvegicus], gi\|149047637\|gb\|EDM00307.1\| drebrin-like, isoform CRA_b [Rattus norvegicus] | gi\|13786198 (+3) | 48 | 27 | 24 | 3 | 11 | 16 |
| 657 | RecName: Full=Bleomycin hydrolase; Short=BLM hydrolase; Short=BMH; Short=BH, gi\|1526452\|dbj\|BAA13333.1\| bleomycin hydrolase [Rattus sp.] | gi\|2499877 | 52 | 45 | 6 | 1 | 3 | 42 |
| 658 | PREDICTED: similar to Kinesin-like protein KIF2 [Rattus norvegicus], gi\|109465911\|ref\|XP_001065173.1\| PREDICTED: similar to Kinesin-like protein KIF2 isoform 2 [Rattus norvegicus], gi\|149059280\|gb\|EDM10287.1\| rCG44775, isoform CRA_a [Rattus norvegicus] | gi\|109464350 | 84 | 38 | 1 | 10 | 10 | 28 |

| | | | | | | | | |
|---|---|---|---|---|---|---|---|---|
| 659 | GTP binding protein 1 (predicted), isoform CRA_a [Rattus norvegicus] | gi\|149065914 | 72 | 30 | 8 | 11 | 14 | 16 |
| 660 | biliverdin reductase B (flavin reductase (NADPH)) [Rattus norvegicus], gi\|149056526\|gb\|EDM07957.1\| biliverdin reductase B (flavin reductase (NADPH)) (predicted), isoform CRA_b [Rattus norvegicus], gi\|197246855\|gb\|AAI68943.1\| Biliverdin reductase B (flavin | gi\|157819619 | 22 | 29 | 19 | 6 | 12 | 17 |
| 661 | rCG51764, isoform CRA_d [Rattus norvegicus] | gi\|149030542 | 284 | 32 | 0 | 3 | 3 | 29 |
| 662 | hook homolog 3 [Rattus norvegicus], gi\|149057834\|gb\|EDM09077.1\| hook homolog 3 (Drosophila) [Rattus norvegicus] | gi\|209869999 | 83 | 40 | 4 | 11 | 12 | 28 |
| 663 | Sjogren's syndrome/scleroderma autoantigen 1 homolog [Rattus norvegicus], gi\|109463401\|ref\|XP_001074570.1\| PREDICTED: similar to C184L-22 [Rattus norvegicus], gi\|149062103\|gb\|EDM12526.1\| mammary tumor virus receptor 2, isoform CRA_a [Rattus norvegicus], g | gi\|157817574 | 21 | 30 | 8 | 12 | 15 | 15 |
| 664 | microtubule-associated protein 1 A, isoform CRA_c [Rattus norvegicus] | gi\|149023090 | 300 | 27 | 0 | 17 | 17 | 10 |
| 665 | histone cluster 3, H2ba [Mus musculus], gi\|162138978\|ref\|NP_001104597.1\| histone cluster 3, H2ba [Rattus norvegicus], gi\|109490813\|ref\|XP_001076753.1\| PREDICTED: similar to histone 3, H2ba [Rattus norvegicus], gi\|81881288\|sp\|Q9D2U9.3\|H2B3A_MOUSE RecName: | gi\|13386452 | 14 | 43 | 10 | 0 | 3 | 40 |
| 666 | ribosomal protein S10 [Mus musculus], gi\|13592069\|ref\|NP_112371.1\| ribosomal protein S10 [Rattus norvegicus], gi\|54039307\|sp\|P63326.1\|RS10_RAT RecName: Full=40S ribosomal protein S10, gi\|54039315\|sp\|P63325.1\|RS10_MOUSE RecName: Full=40S ribosomal protein | gi\|13399310 (+1) | 19 | 49 | 4 | 0 | 1 | 48 |
| 667 | adaptor-related protein complex 2, alpha 1 subunit [Rattus norvegicus], gi\|149056010\|gb\|EDM07441.1\| adaptor protein complex AP-2, alpha 1 subunit (predicted) [Rattus norvegicus] | gi\|157823677 | 108 | 43 | 1 | 8 | 8 | 35 |
| 668 | GNAS complex locus GNASL [Rattus norvegicus], gi\|117959930\|ref\|NP_963910.1\| GNAS complex locus GNASL [Mus musculus], gi\|52000956\|sp\|P63095.1\|GNAS2_RAT RecName: Full=Guanine nucleotide-binding protein G(s) subunit alpha isoforms short; AltName: Full=Adenyl | gi\|9506737 | 46 | 40 | 0 | 4 | 4 | 36 |
| 669 | karyopherin (importin) beta 1 [Rattus norvegicus] | gi\|149054034 | 81 | 43 | 3 | 3 | 4 | 39 |
| 670 | WD repeat domain 47 [Rattus norvegicus], gi\|149025707\|gb\|EDL81950.1\| rCG28460 [Rattus norvegicus] | gi\|215276960 | 102 | 24 | 7 | 12 | 14 | 10 |
| 671 | ubiquitin specific protease 14 [Rattus norvegicus], gi\|55250780\|gb\|AAH85947.1\| Ubiquitin specific peptidase 14 [Rattus norvegicus], gi\|149031713\|gb\|EDL86663.1\| ubiquitin specific protease 14 [Rattus norvegicus] | gi\|56605688 | 56 | 45 | 1 | 6 | 6 | 39 |
| 672 | RecName: Full=Glutathione S-transferase omega-1; AltName: Full=GSTO 1-1; AltName: Full=Glutathione-dependent dehydroascorbate reductase, gi\|208969725\|gb\|ACI32122.1\| glutathione S-transferase omega 1 [Rattus norvegicus] | gi\|12585231 (+1) | 28 | 25 | 23 | 0 | 7 | 18 |
| 673 | glycoprotein, synaptic 2 [Rattus norvegicus], gi\|20177913\|sp\|Q64232.1\|GPSN2_RAT RecName: Full=Synaptic glycoprotein SC2; AltName: Full=Trans-2,3-enoyl-CoA reductase; Short=TER, gi\|2144098\|pir\|\|I56573 synaptic glycoprotein SC2 [imported] - rat, gi\|256994\|g | gi\|19924091 (+1) | 36 | 13 | 0 | 4 | 4 | 9 |
| 674 | ADP-ribosylation factor 4 [Mus musculus], gi\|13162343\|ref\|NP_077065.1\| ADP-ribosylation factor 4 [Rattus norvegicus], gi\|48428789\|sp\|P61751.2\|ARF4_RAT RecName: Full=ADP-ribosylation factor 4, gi\|48428790\|sp\|P61750.2\|ARF4_MOUSE RecName: Full=ADP-ribosylati | gi\|6680720 | 20 | 25 | 25 | 0 | 8 | 17 |
| 675 | vacuolar protein sorting 29 [Rattus norvegicus], gi\|224493432\|sp\|B2RZ78.2\|VPS29_RAT RecName: Full=Vacuolar protein sorting-associated protein 29; AltName: Full=Vesicle protein sorting 29, gi\|149063370\|gb\|EDM13693.1\| vacuolar protein sorting 29 (S. pombe) | gi\|157786944 | 20 | 46 | 4 | 0 | 1 | 45 |
| 676 | PREDICTED: similar to DNA-directed RNA polymerase II largest subunit (RPB1) [Rattus norvegicus], gi\|109491096\|ref\|XP_001079162.1\| PREDICTED: similar to DNA-directed RNA polymerase II largest subunit (RPB1) [Rattus norvegicus] | gi\|109488292 | 217 | 40 | 0 | 16 | 16 | 24 |
| 677 | Abelson helper integration site 1 [Rattus norvegicus], gi\|73921661\|sp\|Q6DTM3.1\|AHI1_RAT RecName: Full=Jouberin; AltName: Full=Abelson helper integration site 1 protein homolog; Short=AHI-1, gi\|49615316\|gb\|AAT66919.1\| AHI1 [Rattus norvegicus] | gi\|50511316 | 120 | 35 | 4 | 12 | 13 | 22 |
| 678 | ubiquitin-activating enzyme 3 [Rattus norvegicus], gi\|50401223\|sp\|Q99MI7.1\|UBA3_RAT RecName: Full=NEDD8-activating enzyme E1 catalytic subunit; AltName: Full=Ubiquitin-like modifier-activating enzyme 3; Short=Ubiquitin-activating enzyme 3; AltName: Full=N | gi\|17105358 | 52 | 35 | 11 | 7 | 10 | 25 |
| 679 | ubiquitin-like modifier activating enzyme 6 [Rattus norvegicus], gi\|149035136\|gb\|EDL89840.1\| similar to RIKEN cDNA 5730469D23 (predicted) [Rattus norvegicus] | gi\|157821599 | 118 | 15 | 14 | 5 | 9 | 6 |
| 680 | RecName: Full=Purine nucleoside phosphorylase; Short=PNP; AltName: Full=Inosine phosphorylase, gi\|149033633\|gb\|EDL88431.1\| nucleoside phosphorylase (mapped), isoform CRA_c [Rattus norvegicus] | gi\|205829287 | 32 | 30 | 20 | 4 | 10 | 20 |
| 681 | cyclin-dependent kinase 5 [Rattus norvegicus], gi\|416783\|sp\|Q03114.1\|CDK5_RAT RecName: Full=Cell division protein kinase 5; AltName: Full=Cyclin-dependent kinase 5; AltName: Full=Tau protein kinase II catalytic subunit; Short=TPKII catalytic subunit; AltN | gi\|18266682 | 33 | 29 | 11 | 7 | 10 | 19 |
| 682 | PREDICTED: similar to plexin A1 [Rattus norvegicus] | gi\|109473842 (+1) | 211 | 33 | 0 | 5 | 5 | 28 |
| 683 | RuvB-like protein 2 [Mus musculus], gi\|149757860\|ref\|XP_001490235.1\| PREDICTED: similar to DNA helicase isoform 1 [Equus caballus], gi\|30316329\|sp\|Q9WTM5.3\|RUVB2_MOUSE RecName: Full=RuvB-like 2; AltName: Full=p47 protein, gi\|4521249\|dbj\|BAA76297.1\| DNA he | gi\|6755382 (+1) | 51 | 35 | 9 | 5 | 8 | 27 |
| 684 | immunity-related GTPase family, Q (predicted) [Rattus norvegicus] | gi\|149056658 | 34 | 29 | 26 | 0 | 8 | 21 |
| 685 | keratin 2 [Rattus norvegicus], gi\|81891699\|sp\|Q6IG02.1\|K22E_RAT RecName: Full=Keratin, type II cytoskeletal 2 epidermal; AltName: Full=Cytokeratin-2e; Short=CK 2e; Short=K2e; Short=keratin-2; AltName: Full=Type II keratin Kb2, gi\|46485118\|tpg\|DAA02228.1\| | gi\|57114290 | 69 | 24 | 24 | 0 | 8 | 16 |
| 686 | PREDICTED: similar to protein phosphatase methylesterase 1 [Rattus norvegicus], gi\|109462506\|ref\|XP_001066863.1\| PREDICTED: similar to protein phosphatase methylesterase 1 [Rattus norvegicus], gi\|149068800\|gb\|EDM18352.1\| protein phosphatase methylesterase | gi\|62641013 | 42 | 29 | 11 | 5 | 8 | 21 |
| 687 | triple functional domain (PTPRF interacting) [Rattus norvegicus], gi\|149026481\|gb\|EDL82631.1\| triple functional domain (PTPRF interacting) [Rattus norvegicus] | gi\|157821177 | 176 | 33 | 9 | 6 | 9 | 24 |
| 688 | leucine rich repeat containing 47 (predicted) [Rattus norvegicus] | gi\|149024755 | 61 | 33 | 0 | 12 | 12 | 21 |
| 689 | mitogen-activated protein kinase kinase 6 [Rattus norvegicus], gi\|14039930\|gb\|AAK53428.1\|AF369384_1 mitogen-activated protein kinase kinase 6 [Rattus norvegicus], gi\|56270321\|gb\|AAH87004.1\| Mitogen-activated protein kinase kinase 6 [Rattus norvegicus], gi | gi\|16758520 | 37 | 16 | 17 | 5 | 10 | 6 |
| 690 | malignant T cell amplified sequence 1 [Rattus norvegicus], gi\|123781859\|sp\|Q4G009.1\|MCTS1_RAT RecName: Full=Malignant T cell amplified sequence 1; Short=MCT-1, gi\|71051757\|gb\|AAH98848.1\| Malignant T cell amplified sequence 1 [Rattus norvegicus], gi\|742105 | gi\|112984244 (+1) | 21 | 38 | 10 | 0 | 3 | 35 |
| 691 | ubiquitin specific protease 47 (predicted), isoform CRA_b [Rattus norvegicus] | gi\|149068273 (+1) | 91 | 19 | 26 | 10 | 18 | 1 |
| 692 | S-phase kinase-associated protein 1 [Rattus norvegicus], gi\|158854016\|ref\|NP_035673.3\| S-phase kinase-associated protein 1A [Mus musculus], gi\|213512347\|ref\|NP_001134437.1\| S-phase kinase-associated protein 1 [Salmo salar], gi\|54036436\|sp\|Q6PEC4.3\|SKP1_RA | gi\|56090475 | 19 | 28 | 19 | 0 | 6 | 22 |
| 693 | proteasome (prosome, macropain) subunit, beta type 4 [Rattus norvegicus] | gi\|149030731 | 29 | 37 | 13 | 0 | 4 | 33 |
| 694 | growth associated protein 43 [Rattus norvegicus], gi\|128102\|sp\|P07936.1\|NEUM_RAT RecName: Full=Neuromodulin; AltName: Full=Axonal membrane protein GAP-43; AltName: Full=Growth-associated protein 43; AltName: Full=Protein F1, gi\|56128\|emb\|CAA29644.1\| unnam | gi\|8393415 | 24 | 42 | 0 | 1 | 1 | 41 |
| 695 | rCG61105 [Rattus norvegicus] | gi\|149043586 | 35 | 27 | 14 | 1 | 5 | 22 |
| 696 | COP9 constitutive photomorphogenic homolog subunit 2 isoform 1 [Homo sapiens], gi\|23463271\|ref\|NP_695209.1\| COP9 constitutive photomorphogenic homolog subunit 2 [Rattus norvegicus], gi\|70909327\|ref\|NP_034069.2\| COP9 (constitutive photomorphogenic) homolog | gi\|4759264 | 52 | 41 | 0 | 3 | 3 | 38 |
| 697 | zinc responsive protein ZD10B [Rattus norvegicus], gi\|51260031\|gb\|AAH79094.1\| DEAD (Asp-Glu-Ala-Asp) box polypeptide 19a [Rattus norvegicus] | gi\|77628154 | 54 | 26 | 9 | 9 | 12 | 14 |
| 698 | minichromosome maintenance complex component 5 [Rattus norvegicus], gi\|149032486\|gb\|EDL87377.1\| minichromosome maintenance deficient 5, cell division cycle 46 (S. cerevisiae) (predicted) [Rattus norvegicus] | gi\|157823053 | 40 | 36 | 3 | 9 | 10 | 26 |
| 699 | eukaryotic translation initiation factor 3, subunit J [Rattus norvegicus], gi\|109468723\|ref\|XP_001080279.1\| PREDICTED: similar to Eukaryotic translation initiation factor 3 subunit 1 (eIF-3 alpha) [Rattus norvegicus], gi\|187471123\|sp\|A0JPM9.1\|EIF3J_RAT Re | gi\|117940021 | 29 | 44 | 5 | 0 | 2 | 42 |
| 700 | RecName: Full=Tryptophanyl-tRNA synthetase, cytoplasmic; AltName: Full=Tryptophan--tRNA ligase; Short=TrpRS | gi\|125991198 (+1) | 54 | 22 | 28 | 0 | 9 | 13 |
| 701 | pannexin 1 [Rattus norvegicus], gi\|45476996\|sp\|P60570.1\|PANX1_RAT RecName: Full=Pannexin-1, gi\|39930152\|emb\|CAD89522.1\| Pannexin 1 [Rattus norvegicus], gi\|149020650\|gb\|EDL78455.1\| Pannexin 1 [Rattus norvegicus] | gi\|40786483 | 48 | 30 | 0 | 15 | 15 | 15 |
| 702 | mitogen-activated protein kinase kinase kinase kinase 4 [Rattus norvegicus], gi\|149046291\|gb\|EDL99184.1\| mitogen-activated protein kinase kinase kinase kinase 4 (predicted) [Rattus norvegicus] | gi\|157819181 | 135 | 16 | 11 | 7 | 10 | 6 |
| 703 | tropomodulin 2 [Rattus norvegicus], gi\|23396879\|sp\|P70566.1\|TMOD2_RAT RecName: Full=Tropomodulin-2; AltName: Full=Neuronal tropomodulin; Short=N-Tmod, gi\|1628559\|gb\|AAC52854.1\| N-tropomodulin, gi\|149019141\|gb\|EDL77782.1\| tropomodulin 2, isoform CRA_a [Rat | gi\|13928838 | 39 | 29 | 17 | 3 | 8 | 21 |
| 704 | NEDD8 activating enzyme E1 subunit 1 [Rattus norvegicus] | gi\|119850964 (+1) | 60 | 29 | 7 | 8 | 10 | 19 |
| 705 | fermitin family homolog 2 [Rattus norvegicus], gi\|53733599\|gb\|AAH83876.1\| Fermitin family homolog 2 (Drosophila) [Rattus norvegicus], gi\|149033509\|gb\|EDL88307.1\| rCG61183, isoform CRA_b [Rattus norvegicus] | gi\|58865400 | 78 | 34 | 7 | 8 | 10 | 24 |
| 706 | DnaJ (Hsp40) homolog, subfamily A, member 1 [Mus musculus], gi\|12621094\|ref\|NP_075223.1\| DnaJ-like protein 2 [Rattus norvegicus], gi\|51702257\|sp\|P63036.1\|DNJA1_RAT RecName: Full=DnaJ homolog subfamily A member 1; AltName: Full=DnaJ-like protein 1, gi\|5170 | gi\|6680297 | 45 | 40 | 0 | 9 | 9 | 31 |
| 707 | cysteine-rich protein 2 [Rattus norvegicus], gi\|544102\|sp\|P36201.1\|CRIP2_RAT RecName: Full=Cysteine-rich protein 2; Short=CRP2; AltName: Full=Protein ESP1, gi\|487284\|dbj\|BAA04464.1\| cysteine-rich protein 2 [Rattus norvegicus], gi\|38511602\|gb\|AAH61774.1\| C | gi\|11968068 | 23 | 30 | 12 | 7 | 11 | 19 |
| 708 | PREDICTED: similar to importin 11 isoform 2 [Rattus norvegicus] | gi\|109465901 (+1) | 113 | 40 | 4 | 5 | 6 | 34 |
| 709 | proteasome (prosome, macropain) subunit, beta type 2 [Rattus norvegicus], gi\|730377\|sp\|P40307.1\|PSB2_RAT RecName: Full=Proteasome subunit beta type-2; AltName: Full=Proteasome component C7-I; AltName: Full=Macropain subunit C7-I; AltName: Full=Multicataly | gi\|8394079 | 23 | 39 | 4 | 0 | 1 | 38 |
| 710 | enolase-phosphatase 1 [Rattus norvegicus], gi\|81883193\|sp\|Q5PPH0.1\|ENOPH_RAT RecName: Full=Enolase-phosphatase E1; AltName: Full=2,3-diketo-5-methylthio-1-phosphopentane phosphatase; AltName: Full=MASA homolog, gi\|56388604\|gb\|AAH87697.1\| Enolase-phosphata | gi\|57164119 | 29 | 25 | 18 | 0 | 6 | 19 |
| 711 | glyceraldehyde 3-phosphate dehydrogenase [Rattus norvegicus] | gi\|1675349 | 9 | 22 | 4 | 0 | 1 | 21 |
| 712 | keratin 83 [Rattus norvegicus], gi\|155676725\|dbj\|BAF75713.1\| LOC681126 [Rattus norvegicus] | gi\|156119593 | 55 | 41 | 4 | 0 | 1 | 40 |
| 713 | stathmin-like 2 [Rattus norvegicus], gi\|46397776\|sp\|P21818.2\|STMN2_RAT RecName: Full=Stathmin-2; AltName: Full=Superior cervical ganglion-10 protein; Short=Protein SCG10, gi\|11228978\|gb\|AAG33230.1\|AF306458_1 SCG10 [Rattus norvegicus], gi\|56585174\|gb\|AAH87 | gi\|16758184 | 21 | 31 | 10 | 2 | 5 | 26 |

| | | | | | | | | |
|---|---|---|---|---|---|---|---|---|
| 714 | solute carrier family 3 (activators of dibasic and neutral amino acid transport), member 2, isoform CRA_b [Rattus norvegicus] | gi\|149062279 (+1) | 62 | 30 | 0 | 10 | 10 | 20 |
| 715 | uridine monophosphate synthase [Rattus norvegicus], gi\|67678064\|gb\|AAH98033.1\| Uridine monophosphate synthetase [Rattus norvegicus], gi\|149060637\|gb\|EDM11351.1\| uridine monophosphate synthetase, isoform CRA_a [Rattus norvegicus] | gi\|70794780 | 52 | 24 | 15 | 11 | 16 | 8 |
| 716 | adaptor-related protein complex 1, mu 1 subunit [Rattus norvegicus], gi\|212274717\|ref\|NP_001130911.1\| hypothetical protein LOC100192015 [Zea mays], gi\|109940231\|sp\|Q32Q06.3\|AP1M1_RAT RecName: Full=AP-1 complex subunit mu-1; AltName: Full=Adaptor-related p | gi\|112984344 | 49 | 37 | 0 | 4 | 4 | 33 |
| 717 | statin-related protein | gi\|206440 (+1) | 50 | 26 | 5 | 8 | 10 | 16 |
| 718 | coatomer protein complex, subunit zeta 1 [Homo sapiens], gi\|9789913\|ref\|NP_062791.1\| coatomer protein complex, subunit zeta 1 [Mus musculus], gi\|157824117\|ref\|NP_001101587.1\| coatomer protein complex, subunit zeta 1 [Rattus norvegicus], gi\|197097816\|ref\|N | gi\|7706337 | 20 | 26 | 17 | 0 | 5 | 21 |
| 719 | ubiquitin carboxyl-terminal esterase L3 [Rattus norvegicus], gi\|109471423\|ref\|XP_001054346.1\| PREDICTED: similar to Ubiquitin carboxyl-terminal hydrolase isozyme L3 (UCH-L3) (Ubiquitin thioesterase L3) [Rattus norvegicus], gi\|68566104\|sp\|Q91Y78.1\|UCHL3_RA | gi\|158749588 | 26 | 24 | 15 | 5 | 10 | 14 |
| 720 | eukaryotic translation initiation factor 2B, subunit 5 epsilon [Rattus norvegicus], gi\|92090594\|sp\|Q64350.2\|EI2BE_RAT RecName: Full=Translation initiation factor eIF-2B subunit epsilon; AltName: Full=eIF-2B GDP-GTP exchange factor subunit epsilon, gi\|5571 | gi\|55741628 | 80 | 42 | 0 | 1 | 1 | 41 |
| 721 | PREDICTED: similar to basic transcription factor 3-like 4 [Rattus norvegicus] | gi\|109475294 (+2) | 31 | 25 | 21 | 0 | 7 | 18 |
| 722 | rCG40811, isoform CRA_b [Rattus norvegicus] | gi\|149028369 (+1) | 23 | 47 | 0 | 2 | 2 | 45 |
| 723 | EF hand domain containing 2, isoform CRA_a [Rattus norvegicus] | gi\|149024529 (+1) | 20 | 13 | 31 | 0 | 10 | 3 |
| 724 | expressed in non-metastatic cells 1, protein (NM23A) (nucleoside diphosphate kinase) [Rattus norvegicus], gi\|462690\|sp\|Q05982.1\|NDKA_RAT RecName: Full=Nucleoside diphosphate kinase A; Short=NDP kinase A; Short=NDK A; AltName: Full=Tumor metastatic process | gi\|19924089 | 17 | 37 | 11 | 0 | 3 | 34 |
| 725 | Inositol (myo)-1(or 4)-monophosphatase 1 [Rattus norvegicus], gi\|44888968\|sp\|P97697.2\|IMPA1_RAT RecName: Full=Inositol monophosphatase; AltName: Full=Inositol-1(or 4)-monophosphatase; Short=IMPase; Short=IMP; AltName: Full=Lithium-sensitive myo-inositol m | gi\|14091736 | 31 | 24 | 21 | 0 | 7 | 17 |
| 726 | phosphatidylinositol transfer protein [Rattus norvegicus], gi\|130771\|sp\|P16446.2\|PIPNA_RAT RecName: Full=Phosphatidylinositol transfer protein alpha isoform; Short=PtdIns transfer protein alpha; Short=PtdInsTP; Short=PI-TP-alpha, gi\|49259199\|pdb\|1T27\|A Ch | gi\|8393962 | 32 | 17 | 24 | 0 | 8 | 9 |
| 727 | keratin 4 [Rattus norvegicus], gi\|81891698\|sp\|Q6IG00.1\|K2C4_RAT RecName: Full=Keratin, type II cytoskeletal 4; AltName: Full=Cytokeratin-4; Short=CK-4; Short=Keratin-4; Short=K4; AltName: Full=Type II keratin Kb4, gi\|46485122\|tpg\|DAA02230.1\| TPA_exp: type | gi\|57012360 | 58 | 26 | 24 | 0 | 8 | 18 |
| 728 | developmentally regulated GTP binding protein 2 [Mus musculus], gi\|8928105\|sp\|Q9QXB9.1\|DRG2_MOUSE RecName: Full=Developmentally-regulated GTP-binding protein 2; Short=DRG-2, gi\|6688758\|emb\|CAB65258.1\| GTP-binding protein [Mus musculus], gi\|52221213\|gb\|AAH | gi\|10946678 | 41 | 27 | 9 | 13 | 16 | 11 |
| 729 | plakoglobin | gi\|1497985 (+1) | 82 | 19 | 13 | 5 | 9 | 10 |
| 730 | coatomer protein complex, subunit epsilon (predicted), isoform CRA_b [Rattus norvegicus] | gi\|149036008 (+1) | 38 | 29 | 5 | 12 | 14 | 15 |
| 731 | rCG25340, isoform CRA_b [Rattus norvegicus], gi\|149018948\|gb\|EDL77589.1\| rCG25340, isoform CRA_b [Rattus norvegicus] | gi\|149018945 (+1) | 52 | 29 | 0 | 3 | 3 | 26 |
| 732 | aconitase 2, mitochondrial [Rattus norvegicus], gi\|60391194\|sp\|Q9ER34.2\|ACON_RAT RecName: Full=Aconitate hydratase, mitochondrial; Short=Aconitase; AltName: Full=Citrate hydro-lyase; Flags: Precursor, gi\|38541404\|gb\|AAH61999.1\| Aconitase 2, mitochondrial | gi\|40538860 | 85 | 46 | 0 | 0 | 0 | 46 |
| 733 | RAB GTPase activating protein 1 [Rattus norvegicus], gi\|149047907\|gb\|EDM00523.1\| G protein-coupled receptor 21 (predicted), isoform CRA_a [Rattus norvegicus] | gi\|157822571 | 121 | 23 | 18 | 6 | 12 | 11 |
| 734 | C-terminal binding protein 1 [Rattus norvegicus], gi\|14194487\|sp\|Q9Z2F5.3\|CTBP1_RAT RecName: Full=C-terminal-binding protein 1; Short=CtBP1; AltName: Full=C-terminal-binding protein 3; Short=CtBP3; AltName: Full=50 kDa BFA-dependent ADP-ribosylation subst | gi\|15011859 (+3) | 47 | 32 | 6 | 6 | 8 | 24 |
| 735 | galactokinase 1 [Rattus norvegicus], gi\|55715922\|gb\|AAH85919.1\| Galactokinase 1 [Rattus norvegicus], gi\|149054819\|gb\|EDM06636.1\| galactokinase 1, isoform CRA_c [Rattus norvegicus] | gi\|56605662 | 42 | 22 | 11 | 1 | 4 | 18 |
| 736 | PREDICTED: similar to 116 kDa U5 small nuclear ribonucleoprotein component (U5 snRNP-specific protein, 116 kDa) (U5-116 kDa) (Elongation factor Tu GTP-binding domain protein 2) (hSNU114) [Rattus norvegicus], gi\|109491989\|ref\|XP_001081526.1\| PREDICTED: sim | gi\|62657153 | 109 | 33 | 1 | 10 | 10 | 23 |
| 737 | cannabinoid receptor interacting protein 1 [Rattus norvegicus], gi\|68564975\|sp\|Q5M7A7.1\|CNRP1_RAT RecName: Full=CB1 cannabinoid receptor-interacting protein 1; Short=CRIP-1, gi\|56788996\|gb\|AAH88754.1\| Cannabinoid receptor interacting protein 1 [Rattus nor | gi\|62079191 | 19 | 26 | 12 | 0 | 4 | 22 |
| 738 | pyrroline-5-carboxylate reductase-like [Rattus norvegicus], gi\|81883225\|sp\|Q5PQJ6.1\|P5CR3_RAT RecName: Full=Pyrroline-5-carboxylate reductase 3; Short=P5C reductase 3; Short=P5CR 3; AltName: Full=Pyrroline-5-carboxylate reductase-like protein, gi\|56268940 | gi\|58865554 | 29 | 13 | 16 | 9 | 14 | 0 |
| 739 | protein phosphatase 2, regulatory subunit B', epsilon isoform [Rattus norvegicus], gi\|149051461\|gb\|EDM03634.1\| protein phosphatase 2, regulatory subunit B (B56), epsilon isoform (predicted) [Rattus norvegicus] | gi\|157818441 | 50 | 41 | 2 | 0 | 1 | 40 |
| 740 | protease (prosome, macropain) 28 subunit, alpha [Rattus norvegicus], gi\|38014837\|gb\|AAH60574.1\| Proteasome (prosome, macropain) activator subunit 1 [Rattus norvegicus], gi\|149063971\|gb\|EDM14241.1\| rCG23530 [Rattus norvegicus] | gi\|61098214 | 29 | 25 | 16 | 0 | 5 | 20 |
| 741 | PREDICTED: similar to High mobility group protein 1 (HMG-1) (High mobility group protein B1) (Amphoterin) (Heparin-binding protein p30) [Rattus norvegicus] | gi\|109459982 (+3) | 25 | 6 | 37 | 0 | 12 | 0 |
| 742 | ribosomal protein S15 [Homo sapiens], gi\|6677799\|ref\|NP_033117.1\| ribosomal protein S15 [Mus musculus], gi\|8394212\|ref\|NP_058847.1\| ribosomal protein S15 [Rattus norvegicus], gi\|45382615\|ref\|NP_990793.1\| ribosomal protein S15 [Gallus gallus], gi\|47523728\| | gi\|4506687 | 17 | 43 | 0 | 0 | 0 | 43 |
| 743 | aldo-keto reductase family 7, member A2 (aflatoxin aldehyde reductase) [Rattus norvegicus], gi\|226500346\|ref\|NP_001142309.1\| hypothetical protein LOC100274478 [Zea mays], gi\|6815049\|dbj\|BAA90396.1\| androgen-inducible aldehyde reductase [Rattus norvegicus] | gi\|19705537 (+1) | 38 | 18 | 17 | 3 | 8 | 10 |
| 744 | protein phosphatase 1, regulatory (inhibitor) subunit 7 [Rattus norvegicus], gi\|81909701\|sp\|Q5HZV9.1\|PP1R7_RAT RecName: Full=Protein phosphatase 1 regulatory subunit 7; AltName: Full=Protein phosphatase 1 regulatory subunit 22, gi\|57032943\|gb\|AAH88868.1\| | gi\|57634526 | 41 | 36 | 4 | 6 | 7 | 29 |
| 745 | coronin, actin binding protein 1A, isoform CRA_c [Rattus norvegicus] | gi\|149067861 (+1) | 40 | 24 | 12 | 3 | 7 | 17 |
| 746 | sirtuin 5 [Rattus norvegicus], gi\|81884377\|sp\|Q68FX9.1\|SIRT5_RAT RecName: Full=NAD-dependent deacetylase sirtuin-5, gi\|51259345\|gb\|AAH78958.1\| Sirtuin (silent mating type information regulation 2 homolog) 5 (S. cerevisiae) [Rattus norvegicus], gi\|14904511 | gi\|51948488 | 34 | 31 | 6 | 8 | 10 | 21 |
| 747 | MOB1, Mps One Binder kinase activator-like 3 [Rattus norvegicus], gi\|40254521\|ref\|NP_079559.2\| preimplantation protein 3 [Mus musculus], gi\|41349449\|ref\|NP_056202.2\| Mps One Binder kinase activator-like 3 isoform 1 [Homo sapiens], gi\|74024909\|ref\|NP_00102 | gi\|19424180 | 26 | 31 | 5 | 8 | 10 | 21 |
| 748 | syntaxin 1B2 [Rattus norvegicus], gi\|13259378\|ref\|NP_077725.1\| syntaxin 1B [Mus musculus], gi\|16418379\|ref\|NP_443106.1\| syntaxin 1B [Homo sapiens], gi\|114662156\|ref\|XP_001145497.1\| PREDICTED: similar to syntaxin B [Pan troglodytes], gi\|47117086\|sp\|P61266. | gi\|6981600 | 33 | 33 | 2 | 4 | 5 | 28 |
| 749 | Ste-20 related kinase [Rattus norvegicus], gi\|12643480\|sp\|O88506.2\|STK39_RAT RecName: Full=STE20/SPS1-related proline-alanine-rich protein kinase; Short=Ste-20-related kinase; AltName: Full=Serine/threonine-protein kinase 39; AltName: Full=Pancreatic seri | gi\|9507133 | 60 | 28 | 5 | 13 | 15 | 13 |
| 750 | adenylate kinase 1, isoform CRA_c [Rattus norvegicus] | gi\|149039004 (+1) | 23 | 25 | 21 | 0 | 7 | 18 |
| 751 | atlastin GTPase 1 [Rattus norvegicus], gi\|46370380\|gb\|AAS89975.1\| atlastin-like protein [Rattus norvegicus], gi\|62203112\|gb\|AAH92645.1\| Spastic paraplegia 3A homolog (human) [Rattus norvegicus] | gi\|57770372 | 63 | 32 | 0 | 6 | 6 | 26 |
| 752 | inosine triphosphatase [Rattus norvegicus], gi\|149023313\|gb\|EDL80207.1\| inosine triphosphatase (nucleoside triphosphate pyrophosphatase) (mapped), isoform CRA_a [Rattus norvegicus] | gi\|157821079 | 22 | 17 | 28 | 0 | 9 | 8 |
| 753 | RecName: Full=Protein disulfide-isomerase A4; AltName: Full=Protein ERp-72; Short=ERp72; AltName: Full=Calcium-binding protein 2; Short=CaBP2; Flags: Precursor, gi\|38181882\|gb\|AAH61535.1\| Protein disulfide isomerase family A, member 4 [Rattus norvegicus] | gi\|78099786 | 73 | 41 | 2 | 0 | 1 | 40 |
| 754 | MARCKS-like 1 [Rattus norvegicus], gi\|76363234\|sp\|Q9EPH2.3\|MRP_RAT RecName: Full=MARCKS-related protein; AltName: Full=MARCKS-like protein 1; AltName: Full=Macrophage myristoylated alanine-rich C kinase substrate; Short=Mac-MARCKS; Short=MacMARCKS; AltNam | gi\|13540687 | 20 | 38 | 5 | 0 | 2 | 36 |
| 755 | insulin-like growth factor 2 receptor, isoform CRA_b [Rattus norvegicus] | gi\|149027462 (+1) | 274 | 40 | 0 | 0 | 0 | 40 |
| 756 | ATPase, H+ transporting, lysosomal V1 subunit D [Rattus norvegicus], gi\|38648872\|gb\|AAH63177.1\| ATPase, H+ transporting, lysosomal V1 subunit D [Rattus norvegicus], gi\|149051530\|gb\|EDM03703.1\| ATPase, H+ transporting, V1 subunit D, isoform CRA_c [Rattus n | gi\|40786463 | 28 | 29 | 10 | 3 | 6 | 23 |
| 757 | prune [Rattus norvegicus], gi\|81884591\|sp\|Q6AYG3.1\|PRUNE_RAT RecName: Full=Protein prune homolog, gi\|50925647\|gb\|AAH79054.1\| Prune homolog (Drosophila) [Rattus norvegicus] | gi\|56090437 | 50 | 29 | 14 | 2 | 6 | 23 |
| 758 | rCG40648, isoform CRA_a [Rattus norvegicus] | gi\|149067609 (+1) | 112 | 19 | 10 | 8 | 11 | 8 |
| 759 | phosphoribosylpyrophosphate synthetase-associated protein [Rattus norvegicus], gi\|149054853\|gb\|EDM06670.1\| phosphoribosyl pyrophosphate synthetase-associated protein 1, isoform CRA_a [Rattus norvegicus] | gi\|186700616 (+1) | 43 | 31 | 0 | 10 | 10 | 21 |
| 760 | adhesion regulating molecule 1 [Rattus norvegicus], gi\|7339666\|dbj\|BAA92929.1\| cell membrane glycoprotein 110000Mr (surface antigen) homolog [Rattus norvegicus] | gi\|13928990 (+1) | 42 | 27 | 2 | 5 | 6 | 21 |
| 761 | SWI/SNF-related, matrix-associated actin-dependent regulator of chromatin, subfamily a, containing DEAD/H box 1` (predicted), isoform CRA_a [Rattus norvegicus] | gi\|149037041 | 83 | 17 | 19 | 3 | 9 | 8 |
| 762 | hypoxanthine phosphoribosyltransferase 1 [Rattus norvegicus], gi\|123501\|sp\|P27605.1\|HPRT_RAT RecName: Full=Hypoxanthine-guanine phosphoribosyltransferase; Short=HGPRTase; Short=HGPRT, gi\|2117729\|pir\|\|I51842 hypoxanthine phosphoribosyltransferase - rat, gi | gi\|51092266 | 24 | 21 | 17 | 0 | 5 | 16 |
| 763 | TROVE domain family, member 2 [Rattus norvegicus], gi\|149058447\|gb\|EDM09604.1\| TROVE domain family, member 2 (predicted) [Rattus norvegicus] | gi\|157821127 | 60 | 33 | 11 | 2 | 5 | 28 |
| 764 | PREDICTED: similar to G protein-regulated inducer of neurite outgrowth 1 [Rattus norvegicus] | gi\|109504667 (+1) | 94 | 26 | 1 | 11 | 11 | 15 |
| 765 | PREDICTED: similar to CG2747-PB, isoform B [Rattus norvegicus] | gi\|109477832 | 224 | 26 | 4 | 7 | 8 | 18 |
| 766 | rCG53455 [Rattus norvegicus] | gi\|149044546 | 119 | 28 | 0 | 7 | 7 | 21 |
| 767 | calbindin 2 [Rattus norvegicus], gi\|1345670\|sp\|P47728.1\|CALB2_RAT RecName: Full=Calretinin; Short=CR, gi\|55853\|emb\|CAA47385.1\| calretinin [Rattus norvegicus], gi\|56541188\|gb\|AAH87603.1\| Calbindin 2 [Rattus norvegicus], gi\|149038172\|gb\|EDL92532.1\| calbindi | gi\|16758892 | 31 | 21 | 26 | 0 | 8 | 13 |

| | | | | | | | | |
|---|---|---|---|---|---|---|---|---|
| 768 | crystallin, mu [Rattus norvegicus], gi\|48474729\|sp\|Q9QYU4.1\|CRYM_RAT RecName: Full=Mu-crystallin homolog; AltName: Full=CDK108, gi\|5931745\|emb\|CAB56625.1\| mu crystallin [Rattus norvegicus], gi\|56789444\|gb\|AAH88121.1\| Crystallin, mu [Rattus norvegicus], gi | gi\|16758840 | 34 | 13 | 27 | 0 | 9 | 4 |
| 769 | hexokinase 1 [Rattus norvegicus], gi\|6226638\|sp\|P05708.4\|HXK1_RAT RecName: Full=Hexokinase-1; AltName: Full=Hexokinase type I; Short=HK I; AltName: Full=Brain form hexokinase, gi\|157838283\|pdb\|1BG3\|A Chain A, Rat Brain Hexokinase Type I Complex With Gluco | gi\|6981022 | 102 | 38 | 7 | 0 | 2 | 36 |
| 770 | prefoldin 5 [Rattus norvegicus], gi\|149031935\|gb\|EDL86847.1\| prefoldin 5 (predicted), isoform CRA_a [Rattus norvegicus], gi\|197246197\|gb\|AAI69128.1\| Prefoldin subunit 5 [Rattus norvegicus] | gi\|164565396 | 17 | 30 | 14 | 0 | 4 | 26 |
| 771 | Pgm1 protein [Rattus norvegicus] | gi\|118764083 (+2) | 63 | 18 | 24 | 0 | 8 | 10 |
| 772 | PREDICTED: similar to spermine synthase [Rattus norvegicus], gi\|109471106\|ref\|XP_001062228.1\| PREDICTED: similar to spermine synthase [Rattus norvegicus], gi\|149030940\|gb\|EDL85967.1\| rCG63119 [Rattus norvegicus] | gi\|109469027 (+1) | 41 | 25 | 13 | 5 | 9 | 16 |
| 773 | PREDICTED: similar to SLIT-ROBO Rho GTPase-activating protein 1 [Rattus norvegicus], gi\|109482032\|ref\|XP_001053311.1\| PREDICTED: similar to SLIT-ROBO Rho GTPase-activating protein 1 [Rattus norvegicus] | gi\|109480632 | 124 | 33 | 3 | 7 | 8 | 25 |
| 774 | CaBP1 [Rattus norvegicus] | gi\|488838 (+2) | 47 | 41 | 2 | 1 | 2 | 39 |
| 775 | voltage dependent anion channel [Rattus norvegicus] | gi\|4558734 | 30 | 37 | 0 | 3 | 3 | 34 |
| 776 | plexin B2 [Rattus norvegicus], gi\|149017545\|gb\|EDL76549.1\| plexin B2 [Rattus norvegicus] | gi\|157821883 | 206 | 29 | 0 | 3 | 3 | 26 |
| 777 | hypothetical protein LOC683630 [Rattus norvegicus], gi\|81883481\|sp\|Q5U2R0.1\|MAT2B_RAT RecName: Full=Methionine adenosyltransferase 2 subunit beta; AltName: Full=Methionine adenosyltransferase II beta; Short=MAT II beta; AltName: Full=Methionine adenosyltr | gi\|112983992 (+1) | 37 | 29 | 2 | 4 | 5 | 24 |
| 778 | similar to chromosome 11 open reading frame2 (predicted), isoform CRA_a [Rattus norvegicus] | gi\|149062135 | 86 | 35 | 2 | 6 | 7 | 28 |
| 779 | actin related protein 2/3 complex, subunit 3 [Rattus norvegicus], gi\|149063364\|gb\|EDM13687.1\| actin related protein 2/3 complex, subunit 3 (predicted), isoform CRA_b [Rattus norvegicus], gi\|183986078\|gb\|AAI66554.1\| Actin related protein 2/3 complex, subun | gi\|157786926 | 21 | 23 | 17 | 0 | 5 | 18 |
| 780 | transgelin 3 [Rattus norvegicus], gi\|78214339\|ref\|NP_113864.2\| transgelin 3 [Rattus norvegicus], gi\|124056477\|sp\|P37805.2\|TAGL3_RAT RecName: Full=Transgelin-3; AltName: Full=Neuronal protein NP25; AltName: Full=Neuronal protein 22; Short=NP22, gi\|18252579 | gi\|78214333 | 23 | 29 | 15 | 0 | 5 | 24 |
| 781 | similar to hypothetical protein FLJ20331, isoform CRA_b [Rattus norvegicus] | gi\|149026333 | 68 | 30 | 7 | 4 | 6 | 24 |
| 782 | glutamyl-prolyl-tRNA synthetase [Rattus norvegicus], gi\|37654316\|gb\|AAQ96263.1\| LRRGT00050 [Rattus norvegicus] | gi\|66793366 | 167 | 29 | 6 | 9 | 11 | 18 |
| 783 | glucose-6-phosphate dehydrogenase [Rattus norvegicus], gi\|120733\|sp\|P05370.3\|G6PD_RAT RecName: Full=Glucose-6-phosphate 1-dehydrogenase; Short=G6PD, gi\|56196\|emb\|CAA30355.1\| unnamed protein product [Rattus norvegicus], gi\|51980296\|gb\|AAH81820.1\| Glucose-6 | gi\|8393381 | 59 | 24 | 11 | 0 | 3 | 21 |
| 784 | CHMP family, member 7 [Rattus norvegicus], gi\|149049867\|gb\|EDM02191.1\| CHMP family, member 7 (predicted), isoform CRA_a [Rattus norvegicus] | gi\|157820115 | 51 | 30 | 8 | 4 | 7 | 23 |
| 785 | v-src sarcoma (Schmidt-Ruppin A-2) viral oncogene homolog [Rattus norvegicus], gi\|8885998\|gb\|AAF80335.1\|AF157016_1 neuronal C-SRC tyrosine-specific protein kinase [Rattus norvegicus], gi\|149043101\|gb\|EDL96675.1\| Rous sarcoma oncogene, isoform CRA_a [Rattu | gi\|14010835 (+1) | 61 | 35 | 0 | 7 | 7 | 28 |
| 786 | secretory carrier membrane protein 1 [Rattus norvegicus] | gi\|149059066 (+1) | 34 | 39 | 1 | 4 | 4 | 35 |
| 787 | calcium channel, voltage-dependent, alpha 2/delta subunit 2 [Rattus norvegicus] | gi\|149018615 (+1) | 131 | 28 | 0 | 10 | 10 | 18 |
| 788 | APEX nuclease 1 [Rattus norvegicus], gi\|1168468\|sp\|P43138.2\|APEX1_RAT RecName: Full=DNA-(apurinic or apyrimidinic site) lyase; AltName: Full=Apurinic-apyrimidinic endonuclease 1; Short=AP endonuclease 1; AltName: Full=APEX nuclease; Short=APEN, gi\|1187823 | gi\|13162337 | 36 | 6 | 31 | 3 | 13 | 0 |
| 789 | similar to receptor expression enhancing protein 2 [Rattus norvegicus], gi\|111493967\|gb\|AAI05631.1\| Similar to receptor expression enhancing protein 2 [Rattus norvegicus] | gi\|114158668 (+1) | 28 | 37 | 0 | 8 | 8 | 29 |
| 790 | transaldolase [Rattus norvegicus] | gi\|12002054 (+1) | 37 | 10 | 32 | 0 | 10 | 0 |
| 791 | proline synthetase co-transcribed homolog [Rattus norvegicus], gi\|149057846\|gb\|EDM09089.1\| proline synthetase co-transcribed (predicted) [Rattus norvegicus] | gi\|157823503 | 30 | 25 | 13 | 4 | 8 | 17 |
| 792 | serine racemase [Rattus norvegicus], gi\|81894104\|sp\|Q76EQ0.1\|SRR_RAT RecName: Full=Serine racemase, gi\|34419178\|dbj\|BAC84968.1\| serine racemase [Rattus norvegicus], gi\|51858725\|gb\|AAH82014.1\| Serine racemase [Rattus norvegicus], gi\|149053378\|gb\|EDM05195.1 | gi\|38454254 | 36 | 18 | 14 | 6 | 10 | 8 |
| 793 | Ras suppressor protein 1 [Mus musculus], gi\|157821061\|ref\|NP_001102874.1\| Ras suppressor protein 1 [Rattus norvegicus], gi\|109506192\|ref\|XP_001072534.1\| PREDICTED: similar to Ras suppressor protein 1 [Rattus norvegicus], gi\|12848246\|dbj\|BAB27884.1\| unname | gi\|31982028 | 31 | 21 | 8 | 4 | 7 | 14 |
| 794 | Coenzyme A synthase [Rattus norvegicus], gi\|54035300\|gb\|AAH83781.1\| Coenzyme A synthase [Rattus norvegicus] | gi\|55741469 | 62 | 15 | 7 | 4 | 6 | 9 |
| 795 | RecName: Full=Neogenin; Flags: Precursor, gi\|1785999\|gb\|AAB41100.1\| neogenin [Rattus norvegicus] | gi\|10720132 (+1) | 151 | 29 | 4 | 0 | 1 | 28 |
| 796 | tripartite motif protein 3, isoform CRA_a [Rattus norvegicus], gi\|149068479\|gb\|EDM18031.1\| tripartite motif protein 3, isoform CRA_a [Rattus norvegicus], gi\|149068480\|gb\|EDM18032.1\| tripartite motif protein 3, isoform CRA_a [Rattus norvegicus], gi\|1490684 | gi\|149068478 | 81 | 23 | 4 | 10 | 11 | 12 |
| 797 | cell division cycle 42 isoform 2 [Homo sapiens], gi\|56118450\|ref\|NP_001008027.1\| cell division cycle 42 (GTP binding protein, 25kDa) [Xenopus (Silurana) tropicalis], gi\|108998912\|ref\|XP_001100002.1\| PREDICTED: similar to Cell division control protein 42 h | gi\|16357472 | 21 | 27 | 11 | 2 | 5 | 22 |
| 798 | PREDICTED: similar to proteasome (prosome, macropain) activator subunit 4 [Rattus norvegicus], gi\|109501140\|ref\|XP_001066053.1\| PREDICTED: similar to proteasome (prosome, macropain) activator subunit 4 [Rattus norvegicus] | gi\|109500155 (+1) | 229 | 31 | 2 | 2 | 3 | 28 |
| 799 | Eph receptor B2 (predicted) [Rattus norvegicus] | gi\|149024325 | 103 | 31 | 0 | 12 | 12 | 19 |
| 800 | growth factor receptor-bound protein 2 isoform 1 [Homo sapiens], gi\|77539774\|ref\|NP_110473.2\| growth factor receptor bound protein 2 [Rattus norvegicus], gi\|77736207\|ref\|NP_001029802.1\| growth factor receptor-bound protein 2 [Bos taurus], gi\|197097744\|ref | gi\|4504111 | 25 | 11 | 21 | 0 | 7 | 4 |
| 801 | v-crk sarcoma virus CT10 oncogene homolog [Rattus norvegicus], gi\|2842661\|sp\|Q63768.1\|CRK_RAT RecName: Full=Proto-oncogene C-crk; AltName: Full=p38; AltName: Full=Adapter molecule crk, gi\|119389392\|pdb\|2EYZ\|A Chain A, Ct10-Regulated Kinase Isoform Ii, gi\| | gi\|9506515 | 34 | 15 | 25 | 0 | 8 | 7 |
| 802 | ADP-ribosylation-like factor 6 interacting protein 5 [Rattus norvegicus], gi\|57012987\|sp\|Q9ES40.1\|PRAF3_RAT RecName: Full=PRA1 family protein 3; AltName: Full=ADP-ribosylation factor-like protein 6-interacting protein 5; Short=ARL-6-interacting protein 5; | gi\|13027426 | 22 | 39 | 1 | 0 | 0 | 39 |
| 803 | vesicle-associated membrane protein 2, isoform CRA_b [Rattus norvegicus] | gi\|149053024 (+6) | 15 | 44 | 0 | 0 | 0 | 44 |
| 804 | protein tyrosine phosphatase | gi\|458333 (+1) | 68 | 29 | 5 | 1 | 3 | 26 |
| 805 | eukaryotic translation initiation factor 6 [Rattus norvegicus], gi\|76780089\|gb\|AAI05765.1\| Eukaryotic translation initiation factor 6 [Rattus norvegicus], gi\|149030867\|gb\|EDL85894.1\| rCG37307, isoform CRA_b [Rattus norvegicus] | gi\|82654198 | 27 | 17 | 16 | 2 | 7 | 10 |
| 806 | chromatin modifying protein 5 [Mus musculus], gi\|70912368\|ref\|NP_001020581.1\| chromatin modifying protein 5 [Rattus norvegicus], gi\|51702143\|sp\|Q9D7S9.1\|CHMP5_MOUSE RecName: Full=Charged multivesicular body protein 5; AltName: Full=Chromatin-modifying pro | gi\|13386442 | 25 | 22 | 10 | 4 | 7 | 15 |
| 807 | oxidative-stress responsive 1 [Rattus norvegicus], gi\|149018277\|gb\|EDL76918.1\| oxidative-stress responsive 1 (predicted) [Rattus norvegicus] | gi\|157824158 | 58 | 29 | 5 | 5 | 7 | 22 |
| 808 | v-crk sarcoma virus CT10 oncogene homolog (avian)-like [Rattus norvegicus], gi\|81889657\|sp\|Q5U2U2.1\|CRKL_RAT RecName: Full=Crk-like protein, gi\|55249763\|gb\|AAH85865.1\| V-crk sarcoma virus CT10 oncogene homolog (avian)-like [Rattus norvegicus], gi\|14901974 | gi\|56605658 | 34 | 28 | 13 | 0 | 4 | 24 |
| 809 | septin 2 [Rattus norvegicus], gi\|81902430\|sp\|Q91Y81.1\|SEPT2_RAT RecName: Full=Septin-2; AltName: Full=Vascular endothelial cell specific protein 11, gi\|13928415\|dbj\|BAB47151.1\| vascular endothelial cell specific protein 11 [Rattus norvegicus], gi\|51858574 | gi\|16924010 | 42 | 33 | 0 | 5 | 5 | 28 |
| 810 | PREDICTED: similar to coiled-coil domain containing 6 [Rattus norvegicus] | gi\|109509406 | 53 | 35 | 2 | 2 | 3 | 32 |
| 811 | suppression of tumorigenicity 13 [Rattus norvegicus], gi\|226501346\|ref\|NP_001141128.1\| hypothetical protein LOC100273214 [Zea mays], gi\|1708200\|sp\|P50503.1\|F10A1_RAT RecName: Full=Hsc70-interacting protein; Short=Hip; AltName: Full=Protein ST13 homolog; A | gi\|13592093 | 41 | 22 | 13 | 0 | 4 | 18 |
| 812 | cullin 4B [Rattus norvegicus], gi\|149060051\|gb\|EDM10867.1\| cullin 4B (predicted) [Rattus norvegicus] | gi\|157819149 | 111 | 29 | 2 | 9 | 10 | 19 |
| 813 | ubiquilin 1 [Rattus norvegicus], gi\|47939201\|gb\|AAH72477.1\| Ubiquilin 1 [Rattus norvegicus], gi\|149039802\|gb\|EDL93918.1\| ubiquilin 1, isoform CRA_a [Rattus norvegicus] | gi\|48675852 | 62 | 34 | 7 | 0 | 2 | 32 |
| 814 | WD-repeat protein [Mus musculus], gi\|84000227\|ref\|NP_001033214.1\| WD repeat domain 68 [Bos taurus], gi\|108936958\|ref\|NP_005819.3\| WD-repeat protein [Homo sapiens], gi\|118405186\|ref\|NP_001072972.1\| WD repeat domain 68 [Gallus gallus], gi\|157817648\|ref\|NP_0 | gi\|58037257 | 39 | 27 | 0 | 12 | 12 | 15 |
| 815 | nuclear cap binding protein subunit 1, 80kDa [Rattus norvegicus], gi\|81882418\|sp\|Q56A27.1\|NCBP1_RAT RecName: Full=Nuclear cap-binding protein subunit 1, gi\|62089490\|gb\|AAH92199.1\| Nuclear cap binding protein subunit 1, 80kDa [Rattus norvegicus], gi\|149045 | gi\|62339326 | 92 | 42 | 1 | 0 | 0 | 42 |
| 816 | eukaryotic translation initiation factor 4 gamma, 2 [Rattus norvegicus] | gi\|153267461 | 102 | 31 | 0 | 8 | 8 | 23 |
| 817 | protein phosphatase 4, catalytic subunit [Homo sapiens], gi\|9790175\|ref\|NP_062648.1\| protein phosphatase 4, catalytic subunit [Mus musculus], gi\|154707914\|ref\|NP_001092578.1\| protein phosphatase 4, catalytic subunit [Bos taurus], gi\|197101625\|ref\|NP_00112 | gi\|4506027 (+1) | 35 | 23 | 6 | 6 | 8 | 15 |
| 818 | PREDICTED: similar to Nucleolar phosphoprotein p130 (Nucleolar 130 kDa protein) (140 kDa nucleolar phosphoprotein) (Nopp140) (Nucleolar and coiled-body phosphoprotein 1) [Rattus norvegicus], gi\|109500535\|ref\|XP_001076105.1\| PREDICTED: similar to Nucleolar | gi\|109499599 | 151 | 8 | 21 | 2 | 9 | 0 |
| 819 | signal recognition particle 68 [Rattus norvegicus], gi\|149054841\|gb\|EDM06658.1\| signal recognition particle 68 (predicted) [Rattus norvegicus], gi\|187469045\|gb\|AAI66787.1\| Signal recognition particle 68 [Rattus norvegicus] | gi\|157819325 | 70 | 25 | 2 | 7 | 8 | 17 |
| 820 | RecName: Full=Estradiol 17-beta-dehydrogenase 12; AltName: Full=17-beta-hydroxysteroid dehydrogenase 12; Short=17-beta-HSD 12; AltName: Full=3-ketoacyl-CoA reductase; Short=KAR, gi\|38494210\|gb\|AAH61543.1\| Hsd17b12 protein [Rattus norvegicus], gi\|149022712 | gi\|81892292 | 35 | 31 | 0 | 6 | 6 | 25 |
| 821 | tyrosine hydroxylase [Rattus norvegicus], gi\|136577\|sp\|P04177.3\|TY3H_RAT RecName: Full=Tyrosine 3-monooxygenase; AltName: Full=Tyrosine 3-hydroxylase; Short=TH, gi\|207409\|gb\|AAA42257.1\| tyrosine hydroxylase (EC 1.14.16.2), gi\|349098\|gb\|AAA42258.1\| tyrosin | gi\|6981652 | 56 | 25 | 2 | 12 | 13 | 12 |
| 822 | rCG56483, isoform CRA_a [Rattus norvegicus] | gi\|149036421 (+4) | 47 | 30 | 1 | 7 | 7 | 23 |
| 823 | rCG57672 [Rattus norvegicus] | gi\|149040185 | 77 | 32 | 0 | 7 | 7 | 25 |
| 824 | PDGF associated protein, gi\|1589641\|prf\|\|2211382A platelet-derived growth factor-associated protein | gi\|1136586 (+1) | 21 | 29 | 10 | 0 | 3 | 26 |

| | | | | | | | | |
|---|---|---|---|---|---|---|---|---|
| 825 | RAB6B, member RAS oncogene family [Rattus norvegicus], gi\|194221614\|ref\|XP_001496020.2\| PREDICTED: similar to RAB6B, member RAS oncogene family [Equus caballus], gi\|149018752\|gb\|EDL77393.1\| RAB6B, member RAS oncogene family (predicted) [Rattus norvegicus] | gi\|157817539 | 20 | 32 | 6 | 0 | 2 | 30 |
| 826 | cell division cycle 37 homolog [Rattus norvegicus], gi\|1197180\|dbj\|BAA05618.1\| unnamed protein product [Rattus norvegicus] | gi\|16758570 (+1) | 44 | 31 | 9 | 0 | 3 | 28 |
| 827 | protein phosphatase 1, regulatory (inhibitor) subunit 14B [Rattus norvegicus], gi\|62122946\|ref\|NP_032915.2\| protein phosphatase 1, regulatory (inhibitor) subunit 14B [Mus musculus], gi\|55583766\|sp\|Q8K3F3.1\|PP14B_RAT RecName: Full=Protein phosphatase 1 reg | gi\|25282423 | 16 | 30 | 10 | 0 | 3 | 27 |
| 828 | RecName: Full=Neurexin-1-alpha; AltName: Full=Neurexin I-alpha; Flags: Precursor | gi\|124106288 (+1) | 168 | 28 | 2 | 0 | 1 | 27 |
| 829 | WW domain binding protein 2, isoform CRA_b [Rattus norvegicus] | gi\|149054826 | 28 | 12 | 22 | 0 | 7 | 5 |
| 830 | RecName: Full=Glycogen synthase kinase-3 beta; Short=GSK-3 beta; AltName: Full=Factor A; Short=FA, gi\|56334\|emb\|CAA37519.1\| unnamed protein product [Rattus norvegicus] | gi\|125374 (+1) | 47 | 31 | 1 | 7 | 7 | 24 |
| 831 | PREDICTED: similar to Signal recognition particle 72 kDa protein (SRP72) isoform 1 [Rattus norvegicus] | gi\|109500449 | 66 | 17 | 1 | 9 | 9 | 8 |
| 832 | dipeptidylpeptidase 6 [Rattus norvegicus], gi\|1169415\|sp\|P46101.1\|DPP6_RAT RecName: Full=Dipeptidyl aminopeptidase-like protein 6; AltName: Full=Dipeptidylpeptidase VI; AltName: Full=Dipeptidylpeptidase 6; AltName: Full=Dipeptidyl peptidase IV-like protei | gi\|12408298 (+1) | 97 | 33 | 0 | 4 | 4 | 29 |
| 833 | hypothetical protein LOC689074 [Rattus norvegicus], gi\|149015556\|gb\|EDL74937.1\| rCG58914 [Rattus norvegicus] | gi\|221325616 | 137 | 17 | 9 | 7 | 10 | 7 |
| 834 | PREDICTED: similar to E3 ubiquitin-protein ligase NEDD4-like protein (Nedd4-2) (NEDD4.2) [Rattus norvegicus] | gi\|109506712 (+1) | 109 | 16 | 8 | 6 | 9 | 7 |
| 835 | rCG51062, isoform CRA_b [Rattus norvegicus] | gi\|149038022 (+1) | 32 | 19 | 0 | 6 | 6 | 13 |
| 836 | plexin A2 [Rattus norvegicus], gi\|149041088\|gb\|EDL95045.1\| plexin A2 (predicted) [Rattus norvegicus] | gi\|157787093 | 126 | 39 | 0 | 2 | 2 | 37 |
| 837 | chloride channel, nucleotide-sensitive, 1A [Rattus norvegicus], gi\|431451\|gb\|AAC37642.1\| regulatory protein, gi\|38014703\|gb\|AAH60555.1\| Clns1a protein [Rattus norvegicus], gi\|149068915\|gb\|EDM18467.1\| chloride channel, nucleotide-sensitive, 1A [Rattus norv | gi\|13929008 | 27 | 33 | 8 | 0 | 3 | 30 |
| 838 | ADP-ribosylarginine hydrolase [Rattus norvegicus] | gi\|202759 (+1) | 40 | 19 | 13 | 5 | 9 | 10 |
| 839 | RecName: Full=Plasma membrane calcium-transporting ATPase 1; Short=PMCA1; AltName: Full=Plasma membrane calcium ATPase isoform 1; AltName: Full=Plasma membrane calcium pump isoform 1 | gi\|14286099 (+3) | 139 | 29 | 0 | 8 | 8 | 21 |
| 840 | ATPase, Ca++ transporting, slow twitch 2 isoform b [Rattus norvegicus], gi\|57303\|emb\|CAA33645.1\| sarcoplasmic reticulum 2+-Ca-ATPase [Rattus norvegicus], gi\|203059\|gb\|AAA40786.1\| non-muscle ATPase | gi\|158635975 (+1) | 110 | 27 | 0 | 9 | 9 | 18 |
| 841 | PREDICTED: similar to Ras-GTPase-activating protein binding protein 1 (ATP-dependent DNA helicase VIII) (GAP SH3-domain binding protein 1) (G3BP-1) (HDH-VIII) [Rattus norvegicus] | gi\|109488113 | 59 | 28 | 2 | 10 | 11 | 17 |
| 842 | PREDICTED: similar to golgi-specific brefeldin A-resistance factor 1 [Rattus norvegicus], gi\|109464034\|ref\|XP_001066196.1\| PREDICTED: similar to golgi-specific brefeldin A-resistance factor 1 [Rattus norvegicus] | gi\|109460176 (+1) | 207 | 25 | 4 | 6 | 7 | 18 |
| 843 | phosphofurin acidic cluster sorting protein 1 [Rattus norvegicus], gi\|52000740\|sp\|O88588.1\|PACS1_RAT RecName: Full=Phosphofurin acidic cluster sorting protein 1; Short=PACS-1, gi\|3347953\|gb\|AAC31815.1\| cytosolic sorting protein PACS-1a [Rattus norvegicus] | gi\|19705535 | 105 | 30 | 0 | 2 | 2 | 28 |
| 844 | rCG36369, isoform CRA_a [Rattus norvegicus] | gi\|149042203 (+2) | 24 | 24 | 10 | 0 | 3 | 21 |
| 845 | casein kinase 1, alpha 1, isoform CRA_e [Mus musculus], gi\|149064403\|gb\|EDM14606.1\| casein kinase 1, alpha 1, isoform CRA_b [Rattus norvegicus] | gi\|148677820 (+5) | 39 | 25 | 2 | 9 | 10 | 15 |
| 846 | synaptophysin [Rattus norvegicus], gi\|135163\|sp\|P07825.1\|SYPH_RAT RecName: Full=Synaptophysin; AltName: Full=Major synaptic vesicle protein p38, gi\|57322\|emb\|CAA29543.1\| unnamed protein product [Rattus norvegicus], gi\|57326\|emb\|CAA29685.1\| unnamed protein | gi\|6981622 | 33 | 25 | 2 | 11 | 12 | 13 |
| 847 | glutamine fructose-6-phosphate transaminase 1, isoform CRA_a [Rattus norvegicus], gi\|149036650\|gb\|EDL91268.1\| glutamine fructose-6-phosphate transaminase 1, isoform CRA_a [Rattus norvegicus] | gi\|149036649 (+1) | 79 | 25 | 2 | 11 | 12 | 13 |
| 848 | filamin, beta [Rattus norvegicus], gi\|149040087\|gb\|EDL94171.1\| filamin, beta (predicted) [Rattus norvegicus] | gi\|157818975 | 275 | 6 | 29 | 2 | 11 | 0 |
| 849 | ubiquitin specific peptidase 11 [Rattus norvegicus], gi\|60422774\|gb\|AAH90333.1\| Ubiquitin specific peptidase 11 [Rattus norvegicus], gi\|149044387\|gb\|EDL97708.1\| ubiquitin specific protease 11 [Rattus norvegicus] | gi\|76443681 | 105 | 22 | 5 | 3 | 5 | 17 |
| 850 | echinoderm microtubule associated protein like 4 (predicted), isoform CRA_b [Rattus norvegicus] | gi\|149050563 (+1) | 94 | 23 | 0 | 4 | 4 | 19 |
| 851 | family with sequence similarity 49, member A [Rattus norvegicus], gi\|149050934\|gb\|EDM03107.1\| similar to hypothetical protein DKFZp566A1524 (predicted) [Rattus norvegicus], gi\|165971268\|gb\|AAI58701.1\| Family with sequence similarity 49, member A [Rattus n | gi\|157821991 | 37 | 8 | 30 | 0 | 9 | 0 |
| 852 | acylpeptide hydrolase, isoform CRA_a [Rattus norvegicus] | gi\|149018550 (+2) | 81 | 18 | 16 | 0 | 5 | 13 |
| 853 | RAB21, member RAS oncogene family [Rattus norvegicus], gi\|81884468\|sp\|Q6AXT5.1\|RAB21_RAT RecName: Full=Ras-related protein Rab-21, gi\|50927400\|gb\|AAH79323.1\| RAB21, member RAS oncogene family [Rattus norvegicus], gi\|149066960\|gb\|EDM16693.1\| RAB21, member | gi\|51948448 | 24 | 31 | 6 | 2 | 4 | 27 |
| 854 | ribosomal protein S5, isoform CRA_b [Rattus norvegicus], gi\|149016578\|gb\|EDL75779.1\| ribosomal protein S5, isoform CRA_b [Rattus norvegicus], gi\|149016579\|gb\|EDL75780.1\| ribosomal protein S5, isoform CRA_b [Rattus norvegicus], gi\|165970894\|gb\|AAI58719.1\| | gi\|149016577 | 23 | 31 | 2 | 6 | 7 | 24 |
| 855 | PREDICTED: similar to Liprin-alpha-3 (Protein tyrosine phosphatase receptor type f polypeptide-interacting protein alpha-3) (PTPRF-interacting protein alpha-3) [Rattus norvegicus], gi\|109461924\|ref\|XP_001080725.1\| PREDICTED: similar to Liprin-alpha-3 (Pro | gi\|109458729 (+1) | 150 | 21 | 12 | 4 | 8 | 13 |
| 856 | PREDICTED: similar to formin-like 2 isoform B [Rattus norvegicus] | gi\|109468179 (+1) | 122 | 22 | 3 | 10 | 11 | 11 |
| 857 | insulin-degrading enzyme [Rattus norvegicus], gi\|547706\|sp\|P35559.1\|IDE_RAT RecName: Full=Insulin-degrading enzyme; AltName: Full=Insulin protease; Short=Insulinase; AltName: Full=Insulysin, gi\|56492\|emb\|CAA47689.1\| insulin-degrading enzyme [Rattus norveg | gi\|6981076 | 118 | 21 | 5 | 6 | 8 | 13 |
| 858 | eukaryotic translation initiation factor 4H [Rattus norvegicus], gi\|81910358\|sp\|Q5XI72.1\|IF4H_RAT RecName: Full=Eukaryotic translation initiation factor 4H; Short=eIF-4H; AltName: Full=Williams-Beuren syndrome chromosomal region 1 protein homolog, gi\|5403 | gi\|55741853 | 27 | 24 | 9 | 2 | 5 | 19 |
| 859 | protein tyrosine phosphatase, non-receptor type 23 [Rattus norvegicus], gi\|3598974\|gb\|AAC62959.1\| protein tyrosine phosphatase TD14 [Rattus norvegicus] | gi\|29789309 (+1) | 163 | 21 | 11 | 2 | 5 | 16 |
| 860 | rCG54570, isoform CRA_b [Rattus norvegicus] | gi\|149055917 (+1) | 21 | 6 | 31 | 0 | 10 | 0 |
| 861 | PREDICTED: similar to Programmed cell death 6-interacting protein (ALG-2-interacting protein X) (ALG-2-interacting protein 1) (E2F1-inducible protein) (Eig2) [Rattus norvegicus], gi\|205371813\|sp\|Q9QZA2.2\|PDC6I_RAT RecName: Full=Programmed cell death 6-int | gi\|109485522 (+1) | 97 | 17 | 14 | 0 | 4 | 13 |
| 862 | asparagine synthetase [Rattus norvegicus], gi\|91771192\|sp\|P49088.3\|ASNS_RAT RecName: Full=Asparagine synthetase [glutamine-hydrolyzing]; AltName: Full=Glutamine-dependent asparagine synthetase, gi\|51859420\|gb\|AAH81719.1\| Asns protein [Rattus norvegicus], | gi\|148747576 (+1) | 64 | 19 | 5 | 10 | 12 | 7 |
| 863 | early endosome antigen 1 [Rattus norvegicus], gi\|149067114\|gb\|EDM16847.1\| early endosome antigen 1 (predicted) [Rattus norvegicus] | gi\|157821387 | 161 | 10 | 20 | 0 | 6 | 4 |
| 864 | PREDICTED: similar to Protein FAM40A [Rattus norvegicus] | gi\|109465351 (+3) | 99 | 15 | 6 | 8 | 10 | 5 |
| 865 | PREDICTED: similar to Histidine triad nucleotide-binding protein 1 (Adenosine 5-monophosphoramidase) (Protein kinase C inhibitor 1) (Protein kinase C-interacting protein 1) (PKCI-1) [Rattus norvegicus] | gi\|109490737 (+1) | 21 | 36 | 0 | 0 | 0 | 36 |
| 866 | toll interacting protein [Rattus norvegicus], gi\|160184946\|sp\|A2RUW1.1\|TOLIP_RAT RecName: Full=Toll-interacting protein, gi\|124481578\|gb\|AAI33068.1\| Toll interacting protein [Rattus norvegicus], gi\|149061684\|gb\|EDM12107.1\| toll interacting protein (predic | gi\|157823763 | 30 | 26 | 6 | 5 | 7 | 19 |
| 867 | TBC1 domain family, member 17 [Rattus norvegicus], gi\|149056024\|gb\|EDM07455.1\| TBC1 domain family, member 17 (predicted) [Rattus norvegicus], gi\|169642259\|gb\|AAI60880.1\| TBC1 domain family, member 17 [Rattus norvegicus] | gi\|157820129 | 73 | 24 | 6 | 4 | 6 | 18 |
| 868 | phosphoribosyl pyrophosphate synthetase 2 [Rattus norvegicus], gi\|125584\|sp\|P09330.3\|PRPS2_RAT RecName: Full=Ribose-phosphate pyrophosphokinase 2; AltName: Full=Phosphoribosyl pyrophosphate synthetase II; Short=PRS-II, gi\|56979\|emb\|CAA34556.1\| unnamed pro | gi\|6981418 | 35 | 28 | 3 | 2 | 3 | 25 |
| 869 | APC-binding protein EB2 [Mus musculus], gi\|56090319\|ref\|NP_001007657.1\| microtubule-associated protein, RP/EB family, member 3 [Rattus norvegicus], gi\|60389846\|sp\|Q5XIT1.1\|MARE3_RAT RecName: Full=Microtubule-associated protein RP/EB family member 3; AltNa | gi\|39930509 | 32 | 29 | 6 | 2 | 4 | 25 |
| 870 | neurofibromin 1 [Rattus norvegicus], gi\|13959414\|sp\|P97526.1\|NF1_RAT RecName: Full=Neurofibromin; AltName: Full=Neurofibromatosis-related protein NF-1, gi\|1841314\|dbj\|BAA08141.1\| neurofibromin [Rattus norvegicus] | gi\|6981264 | 317 | 25 | 0 | 7 | 7 | 18 |
| 871 | coronin, actin-binding protein, 1B, isoform CRA_a [Rattus norvegicus] | gi\|149061951 (+1) | 54 | 23 | 8 | 6 | 9 | 14 |
| 872 | visinin-like 1 [Mus musculus], gi\|21361559\|ref\|NP_003376.2\| visinin-like 1 [Homo sapiens], gi\|27806195\|ref\|NP_776915.1\| visinin-like 1 [Bos taurus], gi\|45383948\|ref\|NP_990586.1\| visinin-like 1 [Gallus gallus], gi\|114576253\|ref\|XP_001135378.1\| PREDICTED: s | gi\|6755983 | 22 | 27 | 11 | 0 | 3 | 24 |
| 873 | similar to fibronectin type 3 and SPRY domain-containing protein, isoform CRA_a [Rattus norvegicus], gi\|169642779\|gb\|AAI60924.1\| Fsd1 protein [Rattus norvegicus] | gi\|149028240 | 56 | 25 | 3 | 8 | 9 | 16 |
| 874 | RecName: Full=Annexin A6; AltName: Full=Annexin-6; AltName: Full=Annexin VI; AltName: Full=Calcium-binding protein 65/67; Short=CBP 65/67, gi\|763181\|emb\|CAA60040.1\| annexin VI [Rattus norvegicus] | gi\|1351943 | 76 | 23 | 2 | 6 | 7 | 16 |
| 875 | basic leucine zipper and W2 domains 1 [Mus musculus], gi\|38454312\|ref\|NP_942084.1\| basic leucine zipper and W2 domains 1 [Rattus norvegicus], gi\|57110923\|ref\|XP_536025.1\| PREDICTED: similar to basic leucine zipper and W2 domains 1 isoform 2 [Canis familia | gi\|13385296 | 48 | 31 | 0 | 2 | 2 | 29 |
| 876 | thyroid hormone receptor interactor 12 [Rattus norvegicus], gi\|71681048\|gb\|AAI00625.1\| Thyroid hormone receptor interactor 12 [Rattus norvegicus] | gi\|72535136 | 218 | 14 | 16 | 5 | 10 | 4 |
| 877 | lin 7 homolog c [Mus musculus], gi\|8922944\|ref\|NP_060832.1\| lin-7 homolog C [Homo sapiens], gi\|11177888\|ref\|NP_068623.1\| lin-7 homolog C [Rattus norvegicus], gi\|115495593\|ref\|NP_001068899.1\| lin-7 homolog C [Bos taurus], gi\|197100117\|ref\|NP_001125773.1\| l | gi\|6755973 | 22 | 24 | 2 | 0 | 1 | 23 |
| 878 | WD repeat domain 61 [Rattus norvegicus], gi\|81907874\|sp\|Q4V7A0.1\|WDR61_RAT RecName: Full=WD repeat-containing protein 61, gi\|66910939\|gb\|AAH98059.1\| WD repeat domain 61 [Rattus norvegicus], gi\|149041708\|gb\|EDL95549.1\| WD repeat domain 61 [Rattus norvegicu | gi\|71043902 | 34 | 26 | 3 | 9 | 10 | 16 |
| 879 | ras homolog gene family, member B [Homo sapiens], gi\|6680726\|ref\|NP_031509.1\| ras homolog gene family, member B [Mus musculus], gi\|11968136\|ref\|NP_071987.1\| ras homolog gene family, member B [Rattus norvegicus], gi\|118150946\|ref\|NP_001071390.1\| ras homolo | gi\|4757764 | 22 | 33 | 2 | 0 | 1 | 32 |
| 880 | N-acetylglucosamine kinase [Rattus norvegicus], gi\|119364611\|sp\|P81799.4\|NAGK_RAT RecName: Full=N-acetyl-D-glucosamine kinase; Short=N- | gi\|83642834 | 37 | 14 | 17 | 6 | 11 | 3 |

| | | | | | | | | |
|---|---|---|---|---|---|---|---|---|
| | acetylglucosamine kinase; AltName: Full=GlcNAc kinase, gi\|78394964\|gb\|AAI07648.1\| N-acetylglucosamine kinase [Rattus nor | | | | | | | |
| 881 | PREDICTED: similar to U2 (RNU2) small nuclear RNA auxiliary factor 2 isoform b isoform 5 [Rattus norvegicus] | gi\|109461138 (+2) | 52 | 24 | 6 | 5 | 7 | 17 |
| 882 | ADP-ribosylation factor 6 [Homo sapiens], gi\|6680724\|ref\|NP_031507.1\| ADP-ribosylation factor 6 [Mus musculus], gi\|13162345\|ref\|NP_077066.1\| ADP-ribosylation factor 6 [Rattus norvegicus], gi\|118403828\|ref\|NP_001072145.1\| ADP-ribosylation factor 6 [Sus scr | gi\|4502211 | 20 | 27 | 7 | 0 | 2 | 25 |
| 883 | reticulon 1 isoform RTN1-C [Mus musculus], gi\|1143717\|gb\|AAC53045.1\| rS-Rex-s, gi\|38098630\|tpg\|DAA01938.1\| TPA_inf: RTN1-C [Mus musculus], gi\|149051415\|gb\|EDM03588.1\| reticulon 1, isoform CRA_a [Rattus norvegicus] | gi\|56090141 | 24 | 22 | 9 | 0 | 3 | 19 |
| 884 | prohibitin [Mus musculus], gi\|13937353\|ref\|NP_114039.1\| prohibitin [Rattus norvegicus], gi\|109490241\|ref\|XP_001053031.1\| PREDICTED: prohibitin [Rattus norvegicus], gi\|54038835\|sp\|P67779.1\|PHB_RAT RecName: Full=Prohibitin, gi\|54038837\|sp\|P67778.1\|PHB_MOUSE | gi\|6679299 | 30 | 29 | 0 | 6 | 6 | 23 |
| 885 | splicing factor 3a, subunit 1 [Rattus norvegicus], gi\|149047546\|gb\|EDM00216.1\| splicing factor 3a, subunit 1 (predicted) [Rattus norvegicus] | gi\|157821905 | 89 | 22 | 0 | 8 | 8 | 14 |
| 886 | ferritin light chain | gi\|204123 (+1) | 21 | 34 | 5 | 0 | 2 | 32 |
| 887 | Bwk1 leukemia-related [Rattus norvegicus], gi\|58037443\|ref\|NP_083883.1\| RIKEN cDNA 2310003C23 [Mus musculus], gi\|28201785\|sp\|Q9D7M1.1\|CT011_MOUSE RecName: Full=Protein C20orf11 homolog; AltName: Full=Two hybrid-associated protein 1 with RanBPM; Short=Twa1 | gi\|38454224 | 27 | 33 | 2 | 3 | 4 | 29 |
| 888 | PREDICTED: similar to guanine nucleotide exchange factor p532 [Rattus norvegicus] | gi\|109484871 | 532 | 19 | 0 | 9 | 9 | 10 |
| 889 | huntingtin-associated protein 1 isoform A [Rattus norvegicus], gi\|1103853\|gb\|AAC52327.1\| rHAP1-A, gi\|1585781\|prf\|\|2201491A huntingtin-associated protein:ISOTYPE=A | gi\|13162306 (+3) | 67 | 19 | 2 | 10 | 11 | 8 |
| 890 | RecName: Full=Keratin, type II cytoskeletal 75; AltName: Full=Cytokeratin-75; Short=CK-75; AltName: Full=Keratin-75; Short=K75; AltName: Full=Type II keratin-18; AltName: Full=Type II keratin-K6hf; AltName: Full=Keratin-6 hair follicle, gi\|149031973\|gb\|ED | gi\|166218811 (+1) | 59 | 15 | 19 | 0 | 6 | 9 |
| 891 | eukaryotic translation initiation factor 2, subunit 3, structural gene X-linked [Rattus norvegicus], gi\|74007205\|ref\|XP_537983.2\| PREDICTED: similar to eukaryotic translation initiation factor 2, subunit 3, structural gene X-linked [Canis familiaris], gi\| | gi\|189217906 | 51 | 26 | 4 | 2 | 3 | 23 |
| 892 | alanyl-tRNA synthetase domain containing 1 [Rattus norvegicus], gi\|81883726\|sp\|Q5XI97.1\|AASD1_RAT RecName: Full=Alanyl-tRNA synthetase domain-containing protein 1, gi\|54035303\|gb\|AAH83790.1\| Alanyl-tRNA synthetase domain containing 1 [Rattus norvegicus], | gi\|77627733 | 45 | 22 | 10 | 2 | 5 | 17 |
| 893 | rCG51948 [Rattus norvegicus], gi\|149030604\|gb\|EDL85641.1\| rCG51723 [Rattus norvegicus] | gi\|149030603 (+2) | 15 | 27 | 7 | 1 | 3 | 24 |
| 894 | syntaxin A, gi\|220902\|dbj\|BAA02089.1\| synaptotagmin associated 35kDa protein [Rattus norvegicus], gi\|251470\|gb\|AAB22525.1\| syntaxin, P35A [rats, brain, Peptide Partial, 285 aa] | gi\|207127 (+3) | 33 | 25 | 0 | 11 | 11 | 14 |
| 895 | SNAP-25 interacting protein hrs-2 [Rattus norvegicus] | gi\|1885385 (+3) | 103 | 25 | 10 | 0 | 3 | 22 |
| 896 | neurocalcin delta [Bos taurus], gi\|31324569\|ref\|NP_598855.2\| neurocalcin delta [Mus musculus], gi\|66730547\|ref\|NP_001019542.1\| neurocalcin delta [Rattus norvegicus], gi\|98985778\|ref\|NP_114430.2\| neurocalcin delta [Homo sapiens], gi\|98985783\|ref\|NP_0010357 | gi\|27806017 | 22 | 32 | 4 | 0 | 1 | 31 |
| 897 | hypothetical protein LOC360664 [Rattus norvegicus], gi\|149054837\|gb\|EDM06654.1\| rCG35178, isoform CRA_a [Rattus norvegicus] | gi\|157819633 | 21 | 19 | 5 | 9 | 11 | 8 |
| 898 | heterogeneous nuclear ribonucleoprotein D isoform a [Rattus norvegicus], gi\|149046816\|gb\|EDL99590.1\| heterogeneous nuclear ribonucleoprotein D, isoform CRA_a [Rattus norvegicus] | gi\|127138710 (+3) | 38 | 8 | 29 | 0 | 9 | 0 |
| 899 | calcium/calmodulin-dependent protein kinase II gamma [Rattus norvegicus], gi\|26667196\|ref\|NP_751909.1\| calcium/calmodulin-dependent protein kinase II gamma isoform 2 [Homo sapiens], gi\|73953041\|ref\|XP_863859.1\| PREDICTED: similar to Calcium/calmodulin-dep | gi\|19424316 (+2) | 59 | 22 | 0 | 16 | 16 | 6 |
| 900 | PTK2 protein tyrosine kinase 2 [Rattus norvegicus], gi\|3182997\|sp\|O35346.1\|FAK1_RAT RecName: Full=Focal adhesion kinase 1; Short=FADK 1; AltName: Full=pp125FAK, gi\|2465667\|gb\|AAB72203.1\| focal adhesion kinase; pp125FAK [Rattus norvegicus] | gi\|6981440 | 120 | 22 | 7 | 4 | 6 | 16 |
| 901 | tissue specific transplantation antigen P35B [Rattus norvegicus], gi\|149066159\|gb\|EDM16032.1\| tissue specific transplantation antigen P35B (predicted), isoform CRA_a [Rattus norvegicus], gi\|149066161\|gb\|EDM16034.1\| tissue specific transplantation antigen | gi\|188536096 | 36 | 25 | 5 | 5 | 7 | 18 |
| 902 | hypothetical protein LOC28088 [Mus musculus], gi\|81880226\|sp\|Q99LF4.1\|CV028_MOUSE RecName: Full=UPF0027 protein C22orf28 homolog, gi\|81884655\|sp\|Q6AYT3.1\|CV028_RAT RecName: Full=UPF0027 protein C22orf28 homolog; AltName: Full=p55, gi\|13096991\|gb\|AAH03288. | gi\|21703842 (+1) | 55 | 18 | 4 | 4 | 5 | 13 |
| 903 | glucosamine-6-phosphate deaminase 1 [Rattus norvegicus], gi\|149017396\|gb\|EDL76447.1\| rCG49489, isoform CRA_a [Rattus norvegicus], gi\|197246475\|gb\|AAI69009.1\| Gnpda1 protein [Rattus norvegicus] | gi\|201861364 | 33 | 25 | 3 | 3 | 4 | 21 |
| 904 | rCG44572 [Rattus norvegicus] | gi\|149059097 | 58 | 16 | 10 | 9 | 12 | 4 |
| 905 | phosphoglucomutase 2-like 1 [Rattus norvegicus], gi\|109462502\|ref\|XP_001066769.1\| PREDICTED: similar to phosphoglucomutase 2-like 1 [Rattus norvegicus], gi\|149068804\|gb\|EDM18356.1\| rCG40012, isoform CRA_a [Rattus norvegicus] | gi\|157822435 | 70 | 17 | 12 | 4 | 8 | 9 |
| 906 | ubiquitin specific peptidase 15, isoform CRA_a [Rattus norvegicus] | gi\|149066659 (+2) | 112 | 23 | 4 | 2 | 3 | 20 |
| 907 | limbic system-associated membrane protein [Rattus norvegicus], gi\|2497324\|sp\|Q62813.1\|LSAMP_RAT RecName: Full=Limbic system-associated membrane protein; Short=LSAMP; Flags: Precursor, gi\|951176\|gb\|AAA86120.1\| limbic system-associated membrane protein, gi\| | gi\|25742796 (+1) | 37 | 23 | 0 | 2 | 2 | 21 |
| 908 | calpain, small subunit 1 [Rattus norvegicus], gi\|83301638\|sp\|Q64537.3\|CPNS1_RAT RecName: Full=Calpain small subunit 1; Short=CSS1; AltName: Full=Calcium-dependent protease small subunit 1; AltName: Full=Calcium-dependent protease small subunit; Short=CDPS | gi\|67514516 | 29 | 15 | 14 | 5 | 9 | 6 |
| 909 | PREDICTED: similar to Prefoldin subunit 3 (Von Hippel-Lindau-binding protein 1) (VHL-binding protein 1) (VBP-1) [Rattus norvegicus] | gi\|109462368 (+1) | 21 | 16 | 17 | 0 | 5 | 11 |
| 910 | microtubule-associated protein 1S [Rattus norvegicus], gi\|160409990\|sp\|P0C5W1.1\|MAP1S_RAT RecName: Full=Microtubule-associated protein 1S; Short=MAP-1S; Contains: RecName: Full=MAP1S heavy chain; Contains: RecName: Full=MAP1S light chain, gi\|149036090\|gb\| | gi\|157823551 | 103 | 29 | 0 | 3 | 3 | 26 |
| 911 | PREDICTED: similar to Roundabout homolog 2 precursor [Rattus norvegicus] | gi\|109492782 (+2) | 161 | 26 | 1 | 5 | 5 | 21 |
| 912 | PREDICTED: similar to thyroid adenoma associated isoform 1 [Rattus norvegicus], gi\|109478909\|ref\|XP_001060686.1\| PREDICTED: similar to thyroid adenoma associated isoform 1 [Rattus norvegicus] | gi\|109477886 | 254 | 13 | 11 | 0 | 3 | 10 |
| 913 | striatin, calmodulin binding protein 4 [Rattus norvegicus], gi\|149056872\|gb\|EDM08303.1\| striatin, calmodulin binding protein 4 (predicted), isoform CRA_a [Rattus norvegicus] | gi\|157819497 | 69 | 27 | 0 | 11 | 11 | 16 |
| 914 | hypothetical protein LOC687575 [Rattus norvegicus], gi\|17368837\|sp\|Q9D883.4\|U2AF1_MOUSE RecName: Full=Splicing factor U2AF 35 kDa subunit; AltName: Full=U2 auxiliary factor 35 kDa subunit; AltName: Full=U2 snRNP auxiliary factor small subunit, gi\|12849864 | gi\|112983840 | 28 | 16 | 2 | 10 | 11 | 5 |
| 915 | ribosomal protein S8 [Homo sapiens], gi\|6677813\|ref\|NP_033124.1\| ribosomal protein S8 [Mus musculus], gi\|13928986\|ref\|NP_113894.1\| ribosomal protein S8 [Rattus norvegicus], gi\|70778956\|ref\|NP_001020488.1\| ribosomal protein S8 [Bos taurus], gi\|55586251\|ref | gi\|4506743 | 24 | 31 | 0 | 0 | 0 | 31 |
| 916 | rCG37481, isoform CRA_b [Rattus norvegicus], gi\|149030843\|gb\|EDL85870.1\| rCG37481, isoform CRA_b [Rattus norvegicus], gi\|149030845\|gb\|EDL85872.1\| rCG37481, isoform CRA_b [Rattus norvegicus], gi\|149030846\|gb\|EDL85873.1\| rCG37481, isoform CRA_b [Rattus norv | gi\|149030842 | 59 | 15 | 2 | 10 | 11 | 4 |
| 917 | flightless I homolog [Rattus norvegicus], gi\|55715671\|gb\|AAH85829.1\| Flightless I homolog (Drosophila) [Rattus norvegicus], gi\|149052840\|gb\|EDM04657.1\| flightless I homolog (Drosophila) [Rattus norvegicus] | gi\|56605668 | 145 | 14 | 9 | 8 | 11 | 3 |
| 918 | RAB35, member RAS oncogene family [Homo sapiens], gi\|37718983\|ref\|NP_937806.1\| RAB35, member RAS oncogene family [Mus musculus], gi\|61556789\|ref\|NP_001013064.1\| RAB35, member RAS oncogene family [Rattus norvegicus], gi\|148229407\|ref\|NP_001091596.1\| RAB35, | gi\|5803135 | 23 | 32 | 0 | 2 | 2 | 30 |
| 919 | D-tyrosyl-tRNA deacylase 1 [Rattus norvegicus], gi\|149041218\|gb\|EDL95151.1\| histidyl tRNA synthetase 2 (predicted) [Rattus norvegicus] | gi\|157819955 | 20 | 28 | 8 | 0 | 3 | 25 |
| 920 | leucine-rich PPR-motif containing, isoform CRA_b [Rattus norvegicus] | gi\|149050517 (+1) | 157 | 28 | 0 | 0 | 0 | 28 |
| 921 | nicotinamide phosphoribosyltransferase [Rattus norvegicus], gi\|68565643\|sp\|Q80Z29.1\|NAMPT_RAT RecName: Full=Nicotinamide phosphoribosyltransferase; Short=NAmPRTase; Short=Nampt; AltName: Full=Pre-B-cell colony-enhancing factor 1 homolog; Short=PBEF; AltNa | gi\|29293813 | 55 | 20 | 5 | 2 | 4 | 16 |
| 922 | rCG53488 [Rattus norvegicus] | gi\|149044483 | 25 | 13 | 15 | 4 | 9 | 4 |
| 923 | translin-associated factor X [Rattus norvegicus], gi\|62901121\|sp\|Q9JHB5.1\|TSNAX_RAT RecName: Full=Translin-associated protein X; AltName: Full=Translin-associated factor X, gi\|8515734\|gb\|AAF76149.1\|AF262357_1 trax [Rattus norvegicus], gi\|51858564\|gb\|AAH81 | gi\|11560081 | 33 | 22 | 5 | 6 | 8 | 14 |
| 924 | SH3-domain GRB2-like 1 [Rattus norvegicus], gi\|10720270\|sp\|O35964.1\|SH3G1_RAT RecName: Full=Endophilin-A2; AltName: Full=Endophilin-2; AltName: Full=SH3 domain-containing GRB2-like protein 1; AltName: Full=SH3 domain protein 2B; AltName: Full=SH3p8, gi\|22 | gi\|13676847 | 41 | 33 | 2 | 0 | 1 | 32 |
| 925 | PREDICTED: similar to Kinesin-like protein KIF3A (Microtubule plus end-directed kinesin motor 3A) isoform 6 [Rattus norvegicus], gi\|109490694\|ref\|XP_001073312.1\| PREDICTED: similar to Kinesin-like protein KIF3A (Microtubule plus end-directed kinesin motor | gi\|109488050 (+7) | 80 | 26 | 0 | 6 | 6 | 20 |
| 926 | glyoxalase domain containing 4 [Rattus norvegicus], gi\|81882968\|sp\|Q5I0D1.1\|GLOD4_RAT RecName: Full=Glyoxalase domain-containing protein 4, gi\|56970492\|gb\|AAH88458.1\| Glyoxalase domain containing 4 [Rattus norvegicus], gi\|149053440\|gb\|EDM05257.1\| similar | gi\|62079189 | 33 | 11 | 22 | 0 | 7 | 4 |
| 927 | DnaJ (Hsp40) homolog, subfamily C, member 7, isoform CRA_a [Rattus norvegicus] | gi\|149054233 (+1) | 56 | 20 | 1 | 9 | 9 | 11 |
| 928 | rCG45759 [Rattus norvegicus] | gi\|149039274 | 156 | 10 | 12 | 4 | 8 | 2 |
| 929 | DnaJ (Hsp40) homolog, subfamily C, member 5 [Mus musculus], gi\|13162361\|ref\|NP_077075.1\| cysteine string protein [Rattus norvegicus], gi\|46397406\|sp\|P60905.1\|DNJC5_RAT RecName: Full=DnaJ homolog subfamily C member 5; AltName: Full=Cysteine string protein; | gi\|7949027 | 22 | 27 | 0 | 7 | 7 | 20 |
| 930 | cytochrome b5 reductase 3 [Rattus norvegicus], gi\|127847\|sp\|P20070.2\|NB5R3_RAT RecName: Full=NADH-cytochrome b5 reductase 3; Short=Cytochrome b5 reductase; Short=B5R; AltName: Full=Diaphorase-1; Contains: RecName: Full=NADH-cytochrome b5 reductase 3 membr | gi\|20302049 | 34 | 24 | 0 | 6 | 6 | 18 |
| 931 | suppressor of Ty 5 homolog (S. cerevisiae), isoform CRA_b [Rattus norvegicus] | gi\|149056473 | 104 | 10 | 16 | 0 | 5 | 5 |
| 932 | post-GPI attachment to proteins 1 [Rattus norvegicus], gi\|81911808\|sp\|Q765A7.1\|PGAP1_RAT RecName: Full=GPI inositol-deacylase; AltName: Full=Post-GPI attachment to proteins factor 1, gi\|41386667\|dbj\|BAD08353.1\| GPI deacylase [Rattus norvegicus], gi\|149046 | gi\|42538976 | 104 | 30 | 0 | 0 | 0 | 30 |
| 933 | myosin, light polypeptide 6, alkali, smooth muscle and non-muscle [Mus musculus], gi\|88999583\|ref\|NP_524147.2\| myosin, light chain 6, alkali, smooth muscle and non-muscle isoform 2 [Homo sapiens], gi\|157823377\|ref\|NP_001102954.1\| myosin, light chain 6, al | gi\|33620739 | 17 | 35 | 0 | 0 | 0 | 35 |
| 934 | eukaryotic translation initiation factor 4A2 [Rattus norvegicus], gi\|77404209\|ref\|NP_001029216.1\| eukaryotic translation initiation factor 4A2 [Bos taurus], | gi\|56605748 | 46 | 22 | 8 | 2 | 5 | 17 |

| | | | | | | | | |
|---|---|---|---|---|---|---|---|---|
| | gi\|83700235\|ref\|NP_001958.2\| eukaryotic translation initiation factor 4A2 [Homo sapiens], gi\|15414 | | | | | | | |
| 935 | alpha-1,3-mannosyltransferase ALG2 [Rattus norvegicus], gi\|149020216\|gb\|EDL78205.1\| asparagine-linked glycosylation 2 homolog (yeast, alpha-1,3-mannosyltransferase), isoform CRA_a [Rattus norvegicus], gi\|149020217\|gb\|EDL78206.1\| asparagine-linked glycosyl | gi\|213511844 | 47 | 23 | 0 | 8 | 8 | 15 |
| 936 | dynactin 5 [Mus musculus], gi\|62900105\|sp\|Q9QZB9.1\|DCTN5_MOUSE RecName: Full=Dynactin subunit 5; AltName: Full=Dynactin subunit p25, gi\|6176550\|gb\|AAF05615.1\|AF190795_1 dynactin subunit p25 [Mus musculus], gi\|12845534\|dbj\|BAB26789.1\| unnamed protein produ | gi\|188219518 | 20 | 24 | 4 | 1 | 2 | 22 |
| 937 | vacuolar protein sorting 26 homolog B [Mus musculus], gi\|197333853\|ref\|NP_001100279.1\| vacuolar protein sorting 26 homolog B [Rattus norvegicus], gi\|81898294\|sp\|Q8C0E2.1\|VP26B_MOUSE RecName: Full=Vacuolar protein sorting-associated protein 26B; AltName: F | gi\|29825827 | 39 | 23 | 0 | 5 | 5 | 18 |
| 938 | influenza virus NS1A binding protein (predicted), isoform CRA_b [Rattus norvegicus] | gi\|149058418 | 72 | 22 | 0 | 9 | 9 | 13 |
| 939 | p21 (CDKN1A)-activated kinase 3 [Rattus norvegicus], gi\|2499645\|sp\|Q62829.1\|PAK3_RAT RecName: Full=Serine/threonine-protein kinase PAK 3; AltName: Full=p21-activated kinase 3; Short=PAK-3; AltName: Full=Beta-PAK; AltName: Full=p65-PAK, gi\|1039425\|gb\|AAC52 | gi\|9506947 | 61 | 26 | 4 | 0 | 1 | 25 |
| 940 | calcium/calmodulin-dependent serine protein kinase (MAGUK family), isoform CRA_a [Rattus norvegicus] | gi\|149044335 (+2) | 105 | 22 | 2 | 1 | 2 | 20 |
| 941 | PREDICTED: similar to SAPS domain family, member 3 [Rattus norvegicus] | gi\|109463290 (+3) | 89 | 15 | 5 | 5 | 7 | 8 |
| 942 | similar to mitotic control protein dis3 homolog (predicted) [Rattus norvegicus] | gi\|149050096 | 107 | 18 | 2 | 5 | 6 | 12 |
| 943 | microtubule-associated protein 2 [Rattus norvegicus], gi\|56625\|emb\|CAA38034.1\| microtubule associated protein 2 [Rattus norvegicus] | gi\|6981182 | 199 | 8 | 17 | 0 | 5 | 3 |
| 944 | secernin 1 [Rattus norvegicus], gi\|81891345\|sp\|Q6AY84.1\|SCRN1_RAT RecName: Full=Secernin-1, gi\|50926985\|gb\|AAH79152.1\| Secernin 1 [Rattus norvegicus], gi\|149033314\|gb\|EDL88115.1\| secernin 1, isoform CRA_a [Rattus norvegicus] | gi\|68163565 | 46 | 17 | 9 | 0 | 3 | 14 |
| 945 | proteasome subunit R-DELTA [Rattus sp.] | gi\|286248 (+2) | 22 | 26 | 2 | 0 | 1 | 25 |
| 946 | eukaryotic translation initiation factor 3, subunit 12 (predicted), isoform CRA_a [Rattus norvegicus] | gi\|149056429 | 25 | 30 | 0 | 2 | 2 | 28 |
| 947 | rCG27500, isoform CRA_b [Rattus norvegicus] | gi\|149041256 (+1) | 23 | 34 | 0 | 0 | 0 | 34 |
| 948 | adaptor-related protein complex 2, sigma 1 subunit [Rattus norvegicus], gi\|70906430\|ref\|NP_004060.2\| adaptor-related protein complex 2, sigma 1 subunit isoform AP17 [Homo sapiens], gi\|115497184\|ref\|NP_001069546.1\| adaptor-related protein complex 2, sigma | gi\|56961624 | 17 | 34 | 0 | 0 | 0 | 34 |
| 949 | PREDICTED: similar to regulator of nonsense transcripts 1 [Rattus norvegicus], gi\|149036014\|gb\|EDL90680.1\| rCG38820 [Rattus norvegicus] | gi\|109504000 | 123 | 16 | 6 | 5 | 7 | 9 |
| 950 | protein phosphatase 1E (PP2C domain containing) [Rattus norvegicus], gi\|81895459\|sp\|Q80Z30.1\|PPM1E_RAT RecName: Full=Protein phosphatase 1E; AltName: Full=Ca(2+)/calmodulin-dependent protein kinase phosphatase N; Short=CaMKP-N; AltName: Full=CaMKP-nucleus | gi\|38454284 | 83 | 22 | 7 | 0 | 2 | 20 |
| 951 | UNC-119 homolog [Rattus norvegicus], gi\|2498855\|sp\|Q62885.1\|U119A_RAT RecName: Full=Protein unc-119 homolog A; AltName: Full=Retinal protein 4; Short=rRG4, gi\|1161378\|gb\|AAC52389.1\| retinal protein, gi\|38303855\|gb\|AAH62057.1\| UNC-119 homolog (C. elegans) | gi\|8394513 | 27 | 14 | 9 | 4 | 7 | 7 |
| 952 | rCG51656 [Rattus norvegicus] | gi\|149038199 (+1) | 26 | 27 | 3 | 2 | 3 | 24 |
| 953 | glyoxalase 1 [Rattus norvegicus], gi\|81885359\|sp\|Q6P7Q4.3\|LGUL_RAT RecName: Full=Lactoylglutathione lyase; AltName: Full=Methylglyoxalase; AltName: Full=Aldoketomutase; AltName: Full=Glyoxalase I; Short=Glx I; AltName: Full=Ketone-aldehyde mutase; AltName | gi\|46485429 | 21 | 21 | 6 | 0 | 2 | 19 |
| 954 | similar to MJ0495-like protein SelB (predicted), isoform CRA_a [Rattus norvegicus], gi\|197246375\|gb\|AAI68694.1\| Eefsec protein [Rattus norvegicus] | gi\|149036692 | 64 | 22 | 2 | 6 | 7 | 15 |
| 955 | intersectin 1 isoform a [Rattus norvegicus], gi\|149059858\|gb\|EDM10741.1\| intersectin 1 [Rattus norvegicus] | gi\|209870013 | 194 | 16 | 10 | 0 | 3 | 13 |
| 956 | exosome component 7 [Rattus norvegicus], gi\|149018124\|gb\|EDL76765.1\| exosome component 7 [Rattus norvegicus] | gi\|213688370 | 32 | 18 | 2 | 8 | 9 | 9 |
| 957 | STIP1 homology and U-Box containing protein 1 [Rattus norvegicus], gi\|149052151\|gb\|EDM03968.1\| STIP1 homology and U-Box containing protein 1, isoform CRA_b [Rattus norvegicus] | gi\|158081745 | 35 | 12 | 7 | 2 | 4 | 8 |
| 958 | PREDICTED: similar to Chromobox protein homolog 1 (Heterochromatin protein 1 homolog beta) (HP1 beta) (Modifier 1 protein) (M31) (Heterochromatin protein p25) [Rattus norvegicus] | gi\|109488886 (+2) | 82 | 15 | 16 | 1 | 6 | 9 |
| 959 | PREDICTED: similar to zyg-11 homolog B (C. elegans)-like [Rattus norvegicus] | gi\|109476879 (+1) | 84 | 19 | 0 | 7 | 7 | 12 |
| 960 | Ac2-269 [Rattus norvegicus] | gi\|32527757 (+1) | 38 | 25 | 0 | 5 | 5 | 20 |
| 961 | syntaxin 7 [Rattus norvegicus], gi\|146345521\|sp\|O70257.4\|STX7_RAT RecName: Full=Syntaxin-7, gi\|55250720\|gb\|AAH85737.1\| Syntaxin 7 [Rattus norvegicus], gi\|149032910\|gb\|EDL87765.1\| syntaxin 7, isoform CRA_a [Rattus norvegicus], gi\|149032912\|gb\|EDL87767.1\| s | gi\|55741787 | 30 | 23 | 0 | 9 | 9 | 14 |
| 962 | ciliary neurotrophic factor receptor, isoform CRA_b [Rattus norvegicus] | gi\|149045684 | 41 | 18 | 2 | 4 | 5 | 13 |
| 963 | glycoprotein m6b [Mus musculus], gi\|148708793\|gb\|EDL40740.1\| glycoprotein m6b, isoform CRA_g [Mus musculus], gi\|149035875\|gb\|EDL90542.1\| rCG49687, isoform CRA_c [Rattus norvegicus] | gi\|123121340 (+3) | 36 | 18 | 0 | 3 | 3 | 15 |
| 964 | RecName: Full=Fatty acid-binding protein, epidermal; AltName: Full=E-FABP; AltName: Full=Fatty acid-binding protein 5; AltName: Full=Cutaneous fatty acid-binding protein; Short=C-FABP; AltName: Full=DA11, gi\|546420\|gb\|AAB30574.1\| C-FABP [Rattus sp.], gi\|1 | gi\|1706754 (+2) | 15 | 24 | 6 | 0 | 2 | 22 |
| 965 | RecName: Full=Histone-arginine methyltransferase CARM1; AltName: Full=Protein arginine N-methyltransferase 4; AltName: Full=Coactivator-associated arginine methyltransferase 1, gi\|71274083\|dbj\|BAE16334.1\| coactivator-associated arginine methyltransferase | gi\|114149244 (+3) | 70 | 24 | 1 | 0 | 0 | 24 |
| 966 | PREDICTED: similar to thioredoxin family Trp26 [Rattus norvegicus], gi\|109477511\|ref\|XP_001068778.1\| PREDICTED: similar to thioredoxin family Trp26 [Rattus norvegicus] | gi\|109475669 (+2) | 30 | 23 | 7 | 0 | 2 | 21 |
| 967 | karyopherin (importin) alpha 2 [Rattus norvegicus], gi\|4456770\|emb\|CAB37408.1\| importin alpha [Rattus norvegicus] | gi\|16758242 (+1) | 58 | 17 | 1 | 4 | 4 | 13 |
| 968 | protein phosphatase 6, catalytic subunit [Mus musculus], gi\|41055937\|ref\|NP_957299.1\| protein phosphatase 6, catalytic subunit [Danio rerio], gi\|51592096\|ref\|NP_598273.2\| protein phosphatase 6, catalytic subunit [Rattus norvegicus], gi\|62859577\|ref\|NP_001 | gi\|21312758 | 35 | 15 | 11 | 6 | 9 | 6 |
| 969 | RecName: Full=Thioredoxin domain-containing protein 9; AltName: Full=ES cell-related protein | gi\|50401221 (+1) | 26 | 20 | 5 | 0 | 2 | 18 |
| 970 | penta-EF hand domain containing 1 [Rattus norvegicus], gi\|81910619\|sp\|Q641Z8.1\|PEF1_RAT RecName: Full=Peflin; AltName: Full=PEF protein with a long N-terminal hydrophobic domain; AltName: Full=Penta-EF hand domain-containing protein 1, gi\|51980425\|gb\|AAH8 | gi\|56090329 | 30 | 14 | 15 | 4 | 9 | 5 |
| 971 | RecName: Full=Cadherin-2; AltName: Full=Neural cadherin; Short=N-cadherin; AltName: CD_antigen=CD325; Flags: Precursor, gi\|3983131\|gb\|AAC83818.1\| testicular N-cadherin [Rattus norvegicus] | gi\|13431334 (+2) | 100 | 18 | 5 | 5 | 7 | 11 |
| 972 | PREDICTED: similar to glucan (1,4-alpha-), branching enzyme 1 [Rattus norvegicus], gi\|149016757\|gb\|EDL75919.1\| glucan (1,4-alpha-), branching enzyme 1, isoform CRA_b [Rattus norvegicus] | gi\|109493745 | 80 | 11 | 6 | 4 | 6 | 5 |
| 973 | RecName: Full=Fumarate hydratase, mitochondrial; Short=Fumarase; Flags: Precursor, gi\|204188\|gb\|AAA41177.1\| fumarase precursor (EC 4.2.1.2) | gi\|120605 | 54 | 27 | 0 | 3 | 3 | 24 |
| 974 | programmed cell death 10 [Rattus norvegicus], gi\|77416557\|sp\|Q6NX65.1\|PDC10_RAT RecName: Full=Programmed cell death protein 10, gi\|45501042\|gb\|AAH67245.1\| Programmed cell death 10 [Rattus norvegicus], gi\|149048308\|gb\|EDM00884.1\| similar to programmed cell | gi\|57164017 | 24 | 12 | 1 | 2 | 2 | 10 |
| 975 | septin 9, isoform CRA_c [Rattus norvegicus] | gi\|149054900 | 65 | 26 | 2 | 3 | 4 | 22 |
| 976 | antidepressant-related protein ADRG123 splice variant [Rattus norvegicus], gi\|149033652\|gb\|EDL88450.1\| N-myc downstream regulated gene 2, isoform CRA_b [Rattus norvegicus], gi\|149033653\|gb\|EDL88451.1\| N-myc downstream regulated gene 2, isoform CRA_b [Ratt | gi\|18478484 (+1) | 39 | 21 | 4 | 1 | 2 | 19 |
| 977 | guanine nucleotide binding protein, alpha z polypeptide [Rattus norvegicus], gi\|121006\|sp\|P19627.3\|GNAZ_RAT RecName: Full=Guanine nucleotide-binding protein G(z) subunit alpha; AltName: Full=G(x) alpha chain; AltName: Full=Gz-alpha, gi\|204547\|gb\|AAA41304. | gi\|6980966 | 41 | 19 | 0 | 8 | 8 | 11 |
| 978 | receptor accessory protein 1 [Rattus norvegicus], gi\|149036387\|gb\|EDL91005.1\| receptor accessory protein 1 (predicted) [Rattus norvegicus] | gi\|157820891 | 22 | 29 | 0 | 0 | 0 | 29 |
| 979 | PREDICTED: similar to Peroxisomal biogenesis factor 19 (Peroxin-19) (Peroxisomal farnesylated protein) [Rattus norvegicus] | gi\|109507564 (+4) | 56 | 19 | 8 | 0 | 3 | 16 |
| 980 | N-acetyltransferase 13 [Rattus norvegicus], gi\|149060453\|gb\|EDM11167.1\| Mak3 homolog (S. cerevisiae) (predicted) [Rattus norvegicus] | gi\|157786904 | 19 | 16 | 10 | 0 | 3 | 13 |
| 981 | phosphatidylinositol binding clathrin assembly protein, isoform CRA_a [Rattus norvegicus] | gi\|149069002 (+3) | 71 | 28 | 2 | 0 | 1 | 27 |
| 982 | lymphocyte antigen 6 complex, locus H [Rattus norvegicus], gi\|149066190\|gb\|EDM16063.1\| lymphocyte antigen 6 complex, locus H (predicted), isoform CRA_a [Rattus norvegicus], gi\|149066191\|gb\|EDM16064.1\| lymphocyte antigen 6 complex, locus H (predicted), iso | gi\|198386345 | 15 | 30 | 0 | 0 | 0 | 30 |
| 983 | rCG51065, isoform CRA_a [Rattus norvegicus] | gi\|149038370 | 65 | 27 | 0 | 0 | 0 | 27 |
| 984 | catenin (cadherin-associated protein), alpha 1, 102kDa [Rattus norvegicus], gi\|55249717\|gb\|AAH85789.1\| Catenin (cadherin associated protein), alpha 1 [Rattus norvegicus], gi\|149017202\|gb\|EDL76253.1\| catenin (cadherin-associated protein), alpha 1, isoform | gi\|55742755 | 100 | 26 | 1 | 2 | 2 | 24 |
| 985 | similar to RIKEN cDNA 1110007L15 (predicted) [Rattus norvegicus] | gi\|149035059 | 37 | 20 | 4 | 5 | 6 | 14 |
| 986 | ATPase, H+ transporting, lysosomal V0 subunit a1 [Rattus norvegicus], gi\|82794742\|gb\|ABB91440.1\| v-H+ATPase subunit a1-I [Rattus norvegicus] | gi\|77627990 (+1) | 96 | 17 | 0 | 1 | 1 | 16 |
| 987 | PREDICTED: similar to MON2 homolog [Rattus norvegicus] | gi\|109480615 | 189 | 6 | 20 | 2 | 8 | 0 |
| 988 | hypothetical protein LOC300783 [Rattus norvegicus], gi\|149041969\|gb\|EDL95810.1\| similar to Butyrate-induced transcript 1 (predicted) [Rattus norvegicus] | gi\|157819829 | 43 | 17 | 0 | 3 | 3 | 14 |
| 989 | cytoplasmic FMR1 interacting protein 2 [Rattus norvegicus], gi\|149052349\|gb\|EDM04166.1\| cytoplasmic FMR1 interacting protein 2 (predicted) [Rattus norvegicus] | gi\|157819571 | 69 | 16 | 2 | 6 | 7 | 9 |
| 990 | karyopherin alpha 4 [Mus musculus], gi\|62339366\|ref\|NP_001014793.1\| karyopherin alpha 4 (importin alpha 3) [Rattus norvegicus], gi\|3122276\|sp\|O35343.1\|IMA4_MOUSE RecName: Full=Importin subunit alpha-4; AltName: Full=Karyopherin subunit alpha-4; AltName: F | gi\|6680598 | 58 | 23 | 6 | 0 | 2 | 21 |
| 991 | nitrilase family, member 2 [Rattus norvegicus], gi\|123789027\|sp\|Q497B0.1\|NIT2_RAT RecName: Full=Nitrilase homolog 2, gi\|72679567\|gb\|AAI00638.1\| Nitrilase family, member 2 [Rattus norvegicus], gi\|149060320\|gb\|EDM11034.1\| nitrilase family, member 2 [Rattus | gi\|77628000 | 31 | 24 | 3 | 1 | 2 | 22 |
| 992 | RuvB-like protein 1 [Mus musculus], gi\|22208848\|ref\|NP_671706.1\| RuvB-like 1 [Rattus norvegicus], gi\|38605681\|sp\|P60123.1\|RUVB1_RAT RecName: Full=RuvB-like 1; AltName: Full=49 kDa TATA box-binding protein-interacting protein; Short=49 kDa TBP-interacting | gi\|9790083 | 50 | 22 | 0 | 5 | 5 | 17 |
| 993 | prohibitin 2 [Rattus norvegicus] | gi\|149049492 (+1) | 32 | 17 | 1 | 5 | 5 | 12 |
| 994 | vacuolar protein sorting 52 homolog [Rattus norvegicus], gi\|81870466\|sp\|O55166.2\|VPS52_RAT RecName: Full=Vacuolar protein sorting-associated protein 52 homolog; AltName: Full=SAC2 suppressor of actin mutations 2-like protein, gi\|3850063\|emb\|CAA11566.1\| AR | gi\|14861866 | 82 | 21 | 0 | 5 | 5 | 16 |

| | | | | | | | | |
|---|---|---|---|---|---|---|---|---|
| 995 | dynactin 3 [Rattus norvegicus], gi\|149045686\|gb\|EDL98686.1\| rCG55152, isoform CRA_a [Rattus norvegicus] | gi\|157821779 | 21 | 26 | 2 | 0 | 1 | 25 |
| 996 | PREDICTED: similar to rap2 interacting protein x [Rattus norvegicus] | gi\|109500414 | 74 | 13 | 6 | 10 | 12 | 1 |
| 997 | valosin-containing protein (p97)/p47 complex-interacting protein p135 [Rattus norvegicus], gi\|55977741\|sp\|Q8CF97.2\|VCIP1_RAT RecName: Full=Deubiquitinating protein VCIP135; AltName: Full=Valosin-containing protein p97/p47 complex-interacting protein p135; | gi\|148747111 | 135 | 23 | 0 | 6 | 6 | 17 |
| 998 | PREDICTED: similar to breakpoint cluster region isoform 1 [Rattus norvegicus] | gi\|109509356 (+2) | 135 | 15 | 5 | 7 | 9 | 6 |
| 999 | endoplasmic reticulum protein 29 [Rattus norvegicus], gi\|2507015\|sp\|P52555.2\|ERP29_RAT RecName: Full=Endoplasmic reticulum protein ERp29; AltName: Full=ERp31; Flags: Precursor, gi\|1772594\|emb\|CAA71313.1\| ERp29 precursor [Rattus norvegicus], gi\|2317800\|gb\| | gi\|16758848 | 29 | 26 | 3 | 0 | 1 | 25 |
| 1000 | RecName: Full=40S ribosomal protein S17, gi\|37231603\|gb\|AAH58484.1\| Ribosomal protein S17 [Rattus norvegicus], gi\|149057361\|gb\|EDM08684.1\| rCG24688, isoform CRA_d [Rattus norvegicus] | gi\|54041951 | 16 | 31 | 0 | 0 | 0 | 31 |
| 1001 | enabled homolog [Rattus norvegicus], gi\|53733843\|gb\|AAH83927.1\| Enabled homolog (Drosophila) [Rattus norvegicus] | gi\|58865872 | 59 | 30 | 0 | 0 | 0 | 30 |
| 1002 | RecName: Full=Mitogen-activated protein kinase 3; AltName: Full=Extracellular signal-regulated kinase 1; Short=ERK-1; AltName: Full=Insulin-stimulated MAP2 kinase; AltName: Full=MAP kinase 1; Short=MAPK 1; AltName: Full=p44-ERK1; AltName: Full=ERT2; AltNa | gi\|115311606 (+4) | 43 | 18 | 5 | 3 | 5 | 13 |
| 1003 | S-adenosylhomocysteine hydrolase-like 1 [Rattus norvegicus], gi\|149025642\|gb\|EDL81885.1\| S-adenosylhomocysteine hydrolase-like 1 (predicted) [Rattus norvegicus] | gi\|157819079 | 53 | 19 | 3 | 3 | 4 | 15 |
| 1004 | RecName: Full=AP-3 complex subunit mu-1; AltName: Full=Adapter-related protein complex 3 mu-1 subunit; AltName: Full=AP-3 adapter complex mu3A subunit; AltName: Full=Mu-adaptin 3A; AltName: Full=Mu3A-adaptin; AltName: Full=Golgi adaptor AP-1 47 kDa protei | gi\|1703027 (+1) | 47 | 26 | 1 | 3 | 3 | 23 |
| 1005 | 3'(2'), 5'-bisphosphate nucleotidase 1 [Rattus norvegicus], gi\|46395575\|sp\|Q9Z1N4.1\|BPNT1_RAT RecName: Full=3'(2'),5'-bisphosphate nucleotidase 1; AltName: Full=Bisphosphate 3'-nucleotidase 1; AltName: Full=PAP-inositol-1,4-phosphatase; Short=PIP; AltName | gi\|25282455 | 33 | 15 | 14 | 0 | 4 | 11 |
| 1006 | polymerase (DNA directed), delta 1, catalytic subunit, isoform CRA_b [Rattus norvegicus] | gi\|149056044 (+1) | 123 | 15 | 12 | 2 | 6 | 9 |
| 1007 | AKT1 kinase [Rattus norvegicus], gi\|1346401\|sp\|P47196.1\|AKT1_RAT RecName: Full=RAC-alpha serine/threonine-protein kinase; AltName: Full=RAC-PK-alpha; AltName: Full=Protein kinase B; Short=PKB, gi\|485403\|dbj\|BAA06279.1\| RAC protein kinase alpha [Rattus nor | gi\|15100164 | 56 | 12 | 9 | 5 | 8 | 4 |
| 1008 | rCG22154, isoform CRA_a [Rattus norvegicus] | gi\|149046323 | 115 | 13 | 10 | 3 | 6 | 7 |
| 1009 | peptidylprolyl isomerase B [Rattus norvegicus], gi\|3288923\|gb\|AAC25590.1\| cyclophilin B [Rattus norvegicus] | gi\|11968126 (+1) | 23 | 22 | 2 | 0 | 1 | 21 |
| 1010 | Vac14 homolog (S. cerevisiae), isoform CRA_a [Rattus norvegicus] | gi\|149038181 (+1) | 87 | 24 | 1 | 0 | 0 | 24 |
| 1011 | rCG51513 [Rattus norvegicus] | gi\|149038259 | 43 | 21 | 9 | 0 | 3 | 18 |
| 1012 | Chain A, Crystal Structure Of A Mu-Like Calpain, gi\|42543460\|pdb\|1QXP\|B Chain B, Crystal Structure Of A Mu-Like Calpain | gi\|42543459 | 103 | 16 | 8 | 1 | 4 | 12 |
| 1013 | PREDICTED: similar to nischarin [Rattus norvegicus], gi\|109503762\|ref\|XP_001058707.1\| PREDICTED: similar to nischarin [Rattus norvegicus], gi\|205829312\|sp\|Q4G017.2\|NISCH_RAT RecName: Full=Nischarin; AltName: Full=Imidazoline receptor 1; Short=IR1; Short=I | gi\|109503020 | 167 | 11 | 15 | 0 | 5 | 6 |
| 1014 | rCG23277 [Rattus norvegicus] | gi\|149044802 | 385 | 15 | 8 | 4 | 7 | 8 |
| 1015 | chromobox homolog 3 [Homo sapiens], gi\|20544151\|ref\|NP_057671.2\| chromobox homolog 3 [Homo sapiens], gi\|56799436\|ref\|NP_001008314.2\| chromobox homolog 3 [Rattus norvegicus], gi\|108860695\|ref\|NP_031650.3\| chromobox homolog 3 [Mus musculus], gi\|155372117\|re | gi\|15082258 | 21 | 21 | 11 | 0 | 3 | 18 |
| 1016 | hippocalcin-like 1 [Mus musculus], gi\|8393864\|ref\|NP_059052.1\| hippocalcin-like 1 [Rattus norvegicus], gi\|51317363\|sp\|P62749.2\|HPCL1_RAT RecName: Full=Hippocalcin-like protein 1; AltName: Full=Visinin-like protein 3; Short=VILIP-3; AltName: Full=Neural vi | gi\|7949055 | 22 | 27 | 0 | 0 | 0 | 27 |
| 1017 | thioredoxin-related transmembrane protein 2 [Rattus norvegicus], gi\|81883793\|sp\|Q5XIK2.1\|TXD14_RAT RecName: Full=Thioredoxin domain-containing protein 14; AltName: Full=Thioredoxin-related transmembrane protein 2; Flags: Precursor, gi\|53733483\|gb\|AAH83678 | gi\|56090369 | 34 | 28 | 0 | 0 | 0 | 28 |
| 1018 | grp75 [Rattus sp.] | gi\|1000439 (+2) | 74 | 27 | 0 | 0 | 0 | 27 |
| 1019 | rCG42519, isoform CRA_a [Rattus norvegicus] | gi\|149029697 | 52 | 27 | 0 | 0 | 0 | 27 |
| 1020 | RecName: Full=Alpha-2-HS-glycoprotein; AltName: Full=Fetuin-A; AltName: Full=Glycoprotein PP63; AltName: Full=59 kDa bone sialic acid-containing protein; Short=BSP; Flags: Precursor, gi\|56140\|emb\|CAA45042.1\| fetuin precursor [Rattus norvegicus], gi\|220676 | gi\|231468 | 38 | 1 | 26 | 0 | 8 | 0 |
| 1021 | X-linked eukaryotic translation initiation factor 1A [Homo sapiens], gi\|31541824\|ref\|NP_079713.2\| eukaryotic translation initiation factor 1A, Y-linked [Mus musculus], gi\|52345532\|ref\|NP_001004814.1\| intracisternal A particle-promoted polypeptide [Xenopus | gi\|4503499 | 16 | 19 | 0 | 0 | 0 | 19 |
| 1022 | Sjogren syndrome antigen B, isoform CRA_b [Rattus norvegicus], gi\|149022179\|gb\|EDL79073.1\| Sjogren syndrome antigen B, isoform CRA_b [Rattus norvegicus] | gi\|149022176 (+1) | 46 | 18 | 8 | 2 | 5 | 13 |
| 1023 | Down syndrome critical region homolog 2 (human) (predicted), isoform CRA_b [Rattus norvegicus] | gi\|149017674 | 33 | 19 | 8 | 1 | 4 | 15 |
| 1024 | adenylosuccinate synthase [Rattus norvegicus], gi\|149040836\|gb\|EDL94793.1\| adenylosuccinate synthetase, non muscle (predicted) [Rattus norvegicus] | gi\|157787018 | 50 | 17 | 6 | 6 | 8 | 9 |
| 1025 | putative MAGE-like protein [Rattus norvegicus] | gi\|12055371 (+1) | 66 | 12 | 12 | 6 | 10 | 2 |
| 1026 | paralemmin, isoform CRA_b [Rattus norvegicus] | gi\|149034654 | 37 | 21 | 1 | 6 | 6 | 15 |
| 1027 | proteasome inhibitor subunit 1 [Rattus norvegicus], gi\|62510947\|sp\|Q5XIU5.1\|PSMF1_RAT RecName: Full=Proteasome inhibitor PI31 subunit, gi\|53734363\|gb\|AAH83575.1\| Similar to Proteasome inhibitor PI31 subunit [Rattus norvegicus], gi\|149031071\|gb\|EDL86098.1\| | gi\|155369692 | 30 | 12 | 4 | 8 | 9 | 3 |
| 1028 | p21 (CDKN1A)-activated kinase 1 [Rattus norvegicus], gi\|3915789\|sp\|P35465.3\|PAK1_RAT RecName: Full=Serine/threonine-protein kinase PAK 1; AltName: Full=p21-activated kinase 1; Short=PAK-1; AltName: Full=p68-PAK; AltName: Full=Alpha-PAK; AltName: Full=Prot | gi\|8393901 | 61 | 22 | 2 | 0 | 1 | 21 |
| 1029 | PREDICTED: similar to EH domain binding protein 1 [Rattus norvegicus] | gi\|109500095 (+2) | 190 | 15 | 7 | 0 | 2 | 13 |
| 1030 | cytochrome c oxidase subunit II [Rattus norvegicus], gi\|117043\|sp\|P00406.2\|COX2_RAT RecName: Full=Cytochrome c oxidase subunit 2; AltName: Full=Cytochrome c oxidase polypeptide II, gi\|13481\|emb\|CAA32957.1\| cytochrome-c oxidase II [Rattus norvegicus], gi\|1 | gi\|110189666 | 26 | 21 | 1 | 7 | 7 | 14 |
| 1031 | PREDICTED: heterogeneous nuclear ribonucleoprotein A1 [Equus caballus], gi\|74222222\|dbj\|BAE26919.1\| unnamed protein product [Mus musculus], gi\|149031878\|gb\|EDL86790.1\| rCG50547, isoform CRA_b [Rattus norvegicus], gi\|149031880\|gb\|EDL86792.1\| rCG50547, isof | gi\|149714872 (+3) | 39 | 15 | 3 | 6 | 7 | 8 |
| 1032 | exosome component 2 [Rattus norvegicus], gi\|149039049\|gb\|EDL93269.1\| exosome component 2 (predicted) [Rattus norvegicus] | gi\|157822341 | 33 | 23 | 2 | 2 | 3 | 20 |
| 1033 | initiation factor eIF-2B gamma subunit | gi\|1537015 | 50 | 20 | 0 | 3 | 3 | 17 |
| 1034 | Cdc42 binding protein kinase beta, isoform CRA_a [Rattus norvegicus] | gi\|149044094 (+4) | 192 | 19 | 0 | 2 | 2 | 17 |
| 1035 | PREDICTED: similar to alveolar soft part sarcoma chromosome region, candidate 1 long isoform isoform 5 [Rattus norvegicus] | gi\|109492391 (+2) | 60 | 24 | 0 | 4 | 4 | 20 |
| 1036 | programmed cell death 6 [Rattus norvegicus], gi\|149032826\|gb\|EDL87681.1\| programmed cell death 6 (predicted), isoform CRA_a [Rattus norvegicus] | gi\|157818653 | 22 | 10 | 14 | 0 | 4 | 6 |
| 1037 | G protein-coupled receptor kinase interacting ArfGAP 1 [Rattus norvegicus], gi\|18203659\|sp\|Q9Z272.1\|GIT1_RAT RecName: Full=ARF GTPase-activating protein GIT1; AltName: Full=G protein-coupled receptor kinase-interactor 1; AltName: Full=GRK-interacting prot | gi\|13929158 (+1) | 85 | 15 | 1 | 4 | 4 | 11 |
| 1038 | laminin, beta 1 [Rattus norvegicus], gi\|149051074\|gb\|EDM03247.1\| laminin, beta 1 (predicted) [Rattus norvegicus] | gi\|157818227 | 112 | 21 | 0 | 1 | 1 | 20 |
| 1039 | mitogen-activated protein kinase kinase 4 [Mus musculus], gi\|71795640\|ref\|NP_001025194.1\| mitogen activated protein kinase kinase 4 [Rattus norvegicus], gi\|2507202\|sp\|P47809.2\|MP2K4_MOUSE RecName: Full=Dual specificity mitogen-activated protein kinase kin | gi\|22095023 | 44 | 12 | 4 | 9 | 10 | 2 |
| 1040 | PREDICTED: similar to SH3-domain GRB2-like 2 [Rattus norvegicus], gi\|109476698\|ref\|XP_001070855.1\| PREDICTED: similar to SH3-domain GRB2-like 2 [Rattus norvegicus] | gi\|109475069 (+1) | 52 | 23 | 5 | 0 | 2 | 21 |
| 1041 | polymerase (RNA) II (DNA directed) polypeptide E [Mus musculus], gi\|157819761\|ref\|NP_001103084.1\| polymerase (RNA) II (DNA directed) polypeptide E, 25kDa [Rattus norvegicus], gi\|109481606\|ref\|XP_001076884.1\| PREDICTED: similar to DNA-directed RNA polymera | gi\|124249077 (+2) | 25 | 24 | 3 | 2 | 3 | 21 |
| 1042 | Na+/K+ -ATPase beta 1 subunit [Rattus norvegicus], gi\|114395\|sp\|P07340.1\|AT1B1_RAT RecName: Full=Sodium/potassium-transporting ATPase subunit beta-1; AltName: Full=Sodium/potassium-dependent ATPase subunit beta-1, gi\|203041\|gb\|AAA40781.1\| Na-, K- ATPase b | gi\|148747253 (+2) | 35 | 27 | 0 | 2 | 2 | 25 |
| 1043 | PREDICTED: similar to SH2 domain binding protein 1 (tetratricopeptide repeat containing) [Rattus norvegicus], gi\|109462689\|ref\|XP_001077817.1\| PREDICTED: similar to SH2 domain binding protein 1 (tetratricopeptide repeat containing) [Rattus norvegicus] | gi\|109459265 (+1) | 144 | 13 | 0 | 8 | 8 | 5 |
| 1044 | TPA: proteasome subunit alpha type 3-like [Rattus norvegicus] | gi\|47169488 (+1) | 28 | 25 | 2 | 0 | 1 | 24 |
| 1045 | rCG23030, isoform CRA_c [Rattus norvegicus] | gi\|149016889 (+2) | 18 | 18 | 0 | 10 | 10 | 8 |
| 1046 | rCG24640, isoform CRA_a [Rattus norvegicus] | gi\|149057093 | 26 | 20 | 4 | 0 | 1 | 19 |
| 1047 | RecName: Full=Carboxypeptidase E; Short=CPE; AltName: Full=Carboxypeptidase H; Short=CPH; AltName: Full=Enkephalin convertase; AltName: Full=Prohormone-processing carboxypeptidase; Flags: Precursor, gi\|203304\|gb\|AAA40875.1\| carboxypeptidase H precursor (E | gi\|115893 (+3) | 53 | 26 | 0 | 0 | 0 | 26 |
| 1048 | frequenin homolog (Drosophila), isoform CRA_a [Rattus norvegicus], gi\|149039058\|gb\|EDL93278.1\| frequenin homolog (Drosophila), isoform CRA_a [Rattus norvegicus] | gi\|149039057 (+1) | 21 | 28 | 0 | 0 | 0 | 28 |
| 1049 | LanC-like 1 [Rattus norvegicus], gi\|47115529\|sp\|Q9QX69.2\|LANC1_RAT RecName: Full=LanC-like protein 1; AltName: Full=40 kDa erythrocyte membrane protein; AltName: Full=p40, gi\|6625487\|emb\|CAB63943.1\| lanthionine synthetase C-like protein 1 [Rattus norvegic | gi\|39930499 | 45 | 15 | 0 | 4 | 4 | 11 |
| 1050 | PREDICTED: similar to Coronin-7 (70 kDa WD repeat tumor rejection antigen homolog) [Rattus norvegicus] | gi\|109487777 (+1) | 106 | 17 | 1 | 5 | 5 | 12 |
| 1051 | vacuolar ATPase subunit H, isoform CRA_a [Rattus norvegicus] | gi\|149060971 | 56 | 20 | 1 | 1 | 1 | 19 |
| 1052 | DNA directed RNA polymerase II polypeptide B [Homo sapiens], gi\|157822127\|ref\|NP_001099472.1\| DNA directed RNA polymerase II polypeptide B [Rattus norvegicus], gi\|114594586\|ref\|XP_517275.2\| PREDICTED: DNA directed RNA polymerase II polypeptide B isoform 2 | gi\|4505941 | 134 | 16 | 6 | 3 | 5 | 11 |
| 1053 | Epsin 1 [Rattus norvegicus], gi\|41016934\|sp\|O88339.1\|EPN1_RAT RecName: Full=Epsin-1; AltName: Full=EPS-15-interacting protein 1, gi\|3249559\|gb\|AAC33823.1\| EH domain binding protein Epsin [Rattus norvegicus], gi\|149016701\|gb\|EDL75887.1\| Epsin 1 [Rattus nor | gi\|16923990 | 60 | 16 | 11 | 1 | 4 | 12 |
| 1054 | putative breast adenocarcinoma marker [Mus musculus], gi\|157821085\|ref\|NP_001102376.1\| chromatin modifying protein 2A [Rattus norvegicus], gi\|73917741\|sp\|Q9DB34.1\|CHM2A_MOUSE RecName: Full=Charged multivesicular body protein 2a; AltName: Full=Chromatin-mo | gi\|21312151 | 25 | 12 | 5 | 2 | 4 | 8 |

| | | | | | | | | |
|---|---|---|---|---|---|---|---|---|
| 1055 | poly(rC) binding protein 2 isoform b [Homo sapiens], gi\|157041229\|ref\|NP_001096635.1\| poly(rC) binding protein 2 isoform 1 [Mus musculus], gi\|73996203\|ref\|XP_534789.2\| PREDICTED: similar to Poly(rC)-binding protein 2 (Alpha-CP2) (Putative heterogeneous nu | gi\|14141166 (+5) | 38 | 12 | 14 | 2 | 6 | 6 |
| 1056 | RecName: Full=Vacuolar protein-sorting-associated protein 25; AltName: Full=ESCRT-II complex subunit VPS25; AltName: Full=ELL-associated protein of 20 kDa | gi\|73920461 | 21 | 17 | 7 | 0 | 2 | 15 |
| 1057 | PREDICTED: similar to pad-1-like isoform 1 [Rattus norvegicus] | gi\|109492847 (+2) | 258 | 17 | 10 | 1 | 4 | 13 |
| 1058 | exosome component 3 (predicted), isoform CRA_a [Rattus norvegicus] | gi\|149045812 | 30 | 18 | 0 | 9 | 9 | 9 |
| 1059 | PREDICTED: similar to Potassium channel tetramerisation domain containing protein 12 (Pfetin) (Predominantly fetal expressed T1 domain) [Rattus norvegicus] | gi\|109501957 | 47 | 17 | 4 | 0 | 1 | 16 |
| 1060 | trafficking protein particle complex 5 [Mus musculus], gi\|157819521\|ref\|NP_001102320.1\| trafficking protein particle complex 5 [Rattus norvegicus], gi\|71153353\|sp\|Q9CQA1.1\|TPPC5_MOUSE RecName: Full=Trafficking protein particle complex subunit 5, gi\|128344 | gi\|157266281 | 21 | 23 | 0 | 2 | 2 | 21 |
| 1061 | casein kinase 2, alpha prime polypeptide [Rattus norvegicus], gi\|149032392\|gb\|EDL87283.1\| casein kinase II, alpha 2, polypeptide (predicted) [Rattus norvegicus] | gi\|157817807 | 41 | 14 | 0 | 9 | 9 | 5 |
| 1062 | cell division cycle 73, Paf1/RNA polymerase II complex component, homolog (S. cerevisiae) [Rattus norvegicus] | gi\|149058452 (+1) | 56 | 21 | 0 | 6 | 6 | 15 |
| 1063 | similar to Amyloid beta (A4) precursor-like protein 1 (predicted) [Rattus norvegicus] | gi\|149056329 | 73 | 19 | 3 | 0 | 1 | 18 |
| 1064 | Na+/Ca2+ exchanger [Rattus norvegicus] | gi\|14132792 (+12) | 107 | 22 | 0 | 5 | 5 | 17 |
| 1065 | RecName: Full=Neuronal-specific septin-3; AltName: Full=G-septin; AltName: Full=P40 | gi\|147732953 (+6) | 41 | 21 | 0 | 0 | 0 | 21 |
| 1066 | rCG30479, isoform CRA_a [Rattus norvegicus] | gi\|149060903 (+3) | 31 | 14 | 0 | 4 | 4 | 10 |
| 1067 | apolipoprotein E, isoform CRA_c [Rattus norvegicus] | gi\|149056721 (+3) | 27 | 6 | 21 | 0 | 7 | 0 |
| 1068 | ribosomal protein S11 [Homo sapiens], gi\|13592071\|ref\|NP_112372.1\| ribosomal protein S11 [Rattus norvegicus], gi\|21426889\|ref\|NP_038753.1\| ribosomal protein S11 [Mus musculus], gi\|55624058\|ref\|XP_517681.1\| PREDICTED: hypothetical protein isoform 3 [Pan tr | gi\|4506681 | 18 | 19 | 0 | 0 | 0 | 19 |
| 1069 | rCG36716, isoform CRA_e [Rattus norvegicus] | gi\|149019924 (+4) | 48 | 0 | 26 | 0 | 8 | 0 |
| 1070 | AMP-activated protein kinase gamma2 subunit [Rattus norvegicus], gi\|33867943\|gb\|AAQ55225.1\| AMP-activated protein kinase gamma 2 non-catalytic subunit [Rattus norvegicus] | gi\|34576559 | 37 | 18 | 5 | 2 | 4 | 14 |
| 1071 | splicing factor, arginine/serine-rich 2 [Mus musculus], gi\|47271443\|ref\|NP_003007.2\| splicing factor, arginine/serine-rich 2 [Homo sapiens], gi\|57528425\|ref\|NP_001009720.1\| splicing factor, arginine/serine-rich 2 [Rattus norvegicus], gi\|77735589\|ref\|NP_00 | gi\|6755478 | 25 | 8 | 9 | 4 | 7 | 1 |
| 1072 | LanC lantibiotic synthetase component C-like 2 [Rattus norvegicus], gi\|51261051\|gb\|AAH79412.1\| LanC lantibiotic synthetase component C-like 2 (bacterial) [Rattus norvegicus] | gi\|62079109 | 51 | 20 | 4 | 1 | 2 | 18 |
| 1073 | RecName: Full=Huntingtin; AltName: Full=Huntington disease protein homolog; Short=HD protein homolog | gi\|1708162 (+1) | 344 | 10 | 6 | 2 | 4 | 6 |
| 1074 | exosome component 5 [Rattus norvegicus], gi\|149056574\|gb\|EDM08005.1\| exosome component 5 (predicted), isoform CRA_a [Rattus norvegicus], gi\|149056575\|gb\|EDM08006.1\| exosome component 5 (predicted), isoform CRA_a [Rattus norvegicus], gi\|149056576\|gb\|EDM080 | gi\|157819675 | 11 | 21 | 1 | 6 | 6 | 15 |
| 1075 | WD repeat domain 7 [Rattus norvegicus], gi\|73920470\|sp\|Q9ERH3.1\|WDR7_RAT RecName: Full=WD repeat-containing protein 7; AltName: Full=TGF-beta resistance-associated protein TRAG, gi\|11127919\|gb\|AAG31140.1\|AF305813_1 TGF-beta resistance-associated protein [ | gi\|13027430 (+1) | 163 | 15 | 2 | 4 | 5 | 10 |
| 1076 | PREDICTED: similar to glycerol-3-phosphate dehydrogenase 1-like [Rattus norvegicus], gi\|109485458\|ref\|XP_001077897.1\| PREDICTED: similar to glycerol-3-phosphate dehydrogenase 1-like [Rattus norvegicus] | gi\|109484025 | 38 | 12 | 11 | 0 | 3 | 9 |
| 1077 | similar to cullin 4A (predicted), isoform CRA_b [Rattus norvegicus] | gi\|149057633 | 88 | 17 | 3 | 4 | 5 | 12 |
| 1078 | hypothetical protein LOC305031 [Rattus norvegicus], gi\|81907947\|sp\|Q4V8K5.1\|BROX_RAT RecName: Full=BRO1 domain-containing protein BROX, gi\|66910563\|gb\|AAH97348.1\| Similar to 0610010K06Rik protein [Rattus norvegicus], gi\|149040931\|gb\|EDL94888.1\| similar to | gi\|71043832 | 46 | 12 | 9 | 0 | 3 | 9 |
| 1079 | methionine sulfoxide reductase A [Rattus norvegicus], gi\|45477002\|sp\|Q923M1.1\|MSRA_RAT RecName: Full=Peptide methionine sulfoxide reductase; AltName: Full=Protein-methionine-S-oxide reductase; Short=PMSR; AltName: Full=Peptide-methionine (S)-S-oxide reduc | gi\|16758004 | 26 | 18 | 7 | 0 | 2 | 16 |
| 1080 | superkiller viralicidic activity 2-like 2 [Rattus norvegicus], gi\|149059353\|gb\|EDM10360.1\| similar to RIKEN cDNA 2610528A15 [Rattus norvegicus] | gi\|77539760 | 118 | 15 | 0 | 6 | 6 | 9 |
| 1081 | Rab geranylgeranyltransferase alpha [Rattus norvegicus], gi\|730316\|sp\|Q08602.1\|PGTA_RAT RecName: Full=Geranylgeranyl transferase type-2 subunit alpha; AltName: Full=Geranylgeranyl transferase type II subunit alpha; AltName: Full=Rab geranylgeranyltransfer | gi\|13928906 (+1) | 65 | 13 | 2 | 6 | 7 | 6 |
| 1082 | eukaryotic translation initiation factor 3, subunit G [Rattus norvegicus], gi\|81910034\|sp\|Q5RK09.1\|EIF3G_RAT RecName: Full=Eukaryotic translation initiation factor 3 subunit G; Short=eIF3g; AltName: Full=Eukaryotic translation initiation factor 3 subunit | gi\|61556927 | 36 | 11 | 1 | 6 | 6 | 5 |
| 1083 | dihydrofolate reductase [Rattus norvegicus], gi\|81871579\|sp\|Q920D2.3\|DYR_RAT RecName: Full=Dihydrofolate reductase, gi\|15982944\|gb\|AAL11500.1\|AF318150_1 dihydrofolate reductase [Rattus norvegicus], gi\|149059008\|gb\|EDM10015.1\| rCG44522 [Rattus norvegicus], | gi\|18426814 | 22 | 10 | 12 | 2 | 6 | 4 |
| 1084 | transmembrane protein [Rattus norvegicus] | gi\|1360136 (+1) | 23 | 21 | 2 | 0 | 1 | 20 |
| 1085 | LIM and SH3 protein 1 [Rattus norvegicus], gi\|57012886\|sp\|Q99MZ8.1\|LASP1_RAT RecName: Full=LIM and SH3 domain protein 1; Short=LASP-1, gi\|13506795\|gb\|AAK28338.1\| LASP-1 [Rattus norvegicus], gi\|71122452\|gb\|AAH99791.1\| LIM and SH3 protein 1 [Rattus norvegic | gi\|14249130 | 30 | 9 | 14 | 0 | 4 | 5 |
| 1086 | PREDICTED: similar to neuron navigator 1 [Rattus norvegicus] | gi\|109498680 (+1) | 202 | 8 | 4 | 4 | 5 | 3 |
| 1087 | hypothetical protein LOC287871 [Rattus norvegicus], gi\|149054982\|gb\|EDM06799.1\| similar to p150 target of rapamycin (TOR)-scaffold protein containing WD-repeats (predicted) [Rattus norvegicus] | gi\|197386276 | 150 | 17 | 0 | 3 | 3 | 14 |
| 1088 | pyruvate kinase, muscle [Rattus norvegicus], gi\|125601\|sp\|P11980.3\|KPYM_RAT RecName: Full=Pyruvate kinase isozymes M1/M2; AltName: Full=Pyruvate kinase muscle isozyme, gi\|56929\|emb\|CAA33799.1\| unnamed protein product [Rattus norvegicus], gi\|206204\|gb\|AAB9 | gi\|16757994 | 58 | 16 | 8 | 0 | 3 | 13 |
| 1089 | ubiquitin specific protease 4 isoform b [Rattus norvegicus], gi\|149018537\|gb\|EDL77178.1\| similar to Ubiquitin carboxyl-terminal hydrolase 4 (Ubiquitin thiolesterase 4) (Ubiquitin-specific processing protease 4) (Deubiquitinating enzyme 4) (Ubiquitous nucl | gi\|157824037 (+1) | 104 | 14 | 2 | 0 | 1 | 13 |
| 1090 | TP53 regulating kinase [Rattus norvegicus], gi\|109471347\|ref\|XP_001072110.1\| PREDICTED: similar to TP53-regulating kinase (p53-related protein kinase) (Nori-2) isoform 2 [Rattus norvegicus], gi\|109471349\|ref\|XP_001072076.1\| PREDICTED: similar to TP53-regu | gi\|157820293 | 27 | 13 | 4 | 7 | 8 | 5 |
| 1091 | COMM domain containing 9 [Rattus norvegicus], gi\|75516470\|gb\|AAI01874.1\| COMM domain containing 9 [Rattus norvegicus], gi\|149022733\|gb\|EDL79627.1\| COMM domain containing 9, isoform CRA_a [Rattus norvegicus], gi\|149022734\|gb\|EDL79628.1\| COMM domain contain | gi\|76096328 | 22 | 18 | 10 | 0 | 3 | 15 |
| 1092 | LUC7-like 2 [Rattus norvegicus], gi\|47115719\|sp\|Q7TNC4.1\|LC7L2_MOUSE RecName: Full=Putative RNA-binding protein Luc7-like 2; AltName: Full=CGI-74 homolog, gi\|33604140\|gb\|AAH56354.1\| LUC7-like 2 (S. cerevisiae) [Mus musculus], gi\|34784543\|gb\|AAH56970.1\| LU | gi\|157822829 | 47 | 11 | 4 | 7 | 8 | 3 |
| 1093 | septin 5, isoform CRA_b [Rattus norvegicus] | gi\|149019801 (+5) | 42 | 25 | 0 | 0 | 0 | 25 |
| 1094 | PREDICTED: similar to peptidylprolyl isomerase H [Rattus norvegicus] | gi\|109477123 (+1) | 26 | 4 | 11 | 0 | 3 | 1 |
| 1095 | restin (Reed-Steinberg cell-expressed intermediate filament-associated protein), isoform CRA_a [Rattus norvegicus] | gi\|149063304 (+2) | 135 | 1 | 20 | 0 | 6 | 0 |
| 1096 | peptidylprolyl cis/trans isomerase, NIMA-interacting 1 [Rattus norvegicus], gi\|149020551\|gb\|EDL78356.1\| protein (peptidyl-prolyl cis/trans isomerase) NIMA-interacting 1 (predicted), isoform CRA_a [Rattus norvegicus], gi\|165970767\|gb\|AAI58868.1\| Peptidylpr | gi\|157817696 | 18 | 22 | 3 | 0 | 1 | 21 |
| 1097 | nucleoredoxin [Rattus norvegicus], gi\|149053442\|gb\|EDM05259.1\| nucleoredoxin (predicted), isoform CRA_a [Rattus norvegicus] | gi\|157818993 | 41 | 20 | 5 | 0 | 2 | 18 |
| 1098 | Prpk (p53-related protein kinase)-binding protein [Rattus norvegicus], gi\|149036589\|gb\|EDL91207.1\| Tp53rk binding protein, isoform CRA_a [Rattus norvegicus], gi\|149036592\|gb\|EDL91210.1\| Tp53rk binding protein, isoform CRA_a [Rattus norvegicus] | gi\|62422579 | 20 | 18 | 6 | 2 | 4 | 14 |
| 1099 | rCG60920, isoform CRA_a [Rattus norvegicus] | gi\|149043815 (+2) | 41 | 11 | 15 | 0 | 5 | 6 |
| 1100 | eukaryotic translation initiation factor 2B, subunit 4 delta [Rattus norvegicus], gi\|2494308\|sp\|Q63186.1\|EI2BD_RAT RecName: Full=Translation initiation factor eIF-2B subunit delta; AltName: Full=eIF-2B GDP-GTP exchange factor subunit delta, gi\|667056\|emb\| | gi\|16758830 | 58 | 16 | 1 | 5 | 5 | 11 |
| 1101 | Rap guanine nucleotide exchange factor (GEF) 2 [Rattus norvegicus], gi\|149048299\|gb\|EDM00875.1\| Rap guanine nucleotide exchange factor (GEF) 2 (predicted) [Rattus norvegicus] | gi\|157818583 | 144 | 3 | 16 | 2 | 7 | 0 |
| 1102 | trafficking protein particle complex 3 [Mus musculus], gi\|56605828\|ref\|NP_001008377.1\| trafficking protein particle complex 3 [Rattus norvegicus], gi\|20177835\|sp\|O55013.1\|TPPC3_MOUSE RecName: Full=Trafficking protein particle complex subunit 3; AltName: F | gi\|7304929 | 20 | 21 | 3 | 0 | 1 | 20 |
| 1103 | synapsin III [Rattus norvegicus], gi\|6707725\|sp\|O70441.1\|SYN3_RAT RecName: Full=Synapsin-3; AltName: Full=Synapsin III, gi\|3170561\|gb\|AAC24521.1\| synapsin IIIa [Rattus norvegicus], gi\|149067390\|gb\|EDM17123.1\| synapsin III, isoform CRA_a [Rattus norvegicus | gi\|8394389 | 63 | 14 | 0 | 1 | 1 | 13 |
| 1104 | splicing factor 3b, subunit 2 (predicted), isoform CRA_a [Rattus norvegicus] | gi\|149062046 | 98 | 14 | 2 | 6 | 7 | 7 |
| 1105 | ADP-ribosylation factor 2, isoform CRA_c [Rattus norvegicus] | gi\|149054474 (+1) | 28 | 19 | 6 | 0 | 2 | 17 |
| 1106 | glutathione S-transferase alpha 4 [Rattus norvegicus], gi\|121714\|sp\|P14942.2\|GSTA4_RAT RecName: Full=Glutathione S-transferase alpha-4; AltName: Full=Glutathione S-transferase Yk; Short=GST Yk; AltName: Full=GST 8-8; AltName: Full=GST K; AltName: Full=GST | gi\|157820217 | 26 | 20 | 4 | 0 | 1 | 19 |
| 1107 | apoptotic peptidase activating factor 1 [Rattus norvegicus], gi\|20137192\|sp\|Q9EPV5.1\|APAF_RAT RecName: Full=Apoptotic protease-activating factor 1; Short=Apaf-1, gi\|11386007\|gb\|AAG35067.1\|AF320222_1 APAF1 [Rattus norvegicus], gi\|149067224\|gb\|EDM16957.1\| a | gi\|13027436 (+5) | 141 | 17 | 4 | 0 | 1 | 16 |
| 1108 | ATP synthase, H+ transporting, mitochondrial F1 complex, gamma subunit [Rattus norvegicus], gi\|37747914\|gb\|AAH59158.1\| ATP synthase, H+ transporting, mitochondrial F1 complex, gamma polypeptide 1 [Rattus norvegicus], gi\|149021018\|gb\|EDL78625.1\| ATP syntha | gi\|39930503 (+2) | 33 | 20 | 0 | 3 | 3 | 17 |
| 1109 | kinesin-associated protein 3 (predicted), isoform CRA_b [Rattus norvegicus] | gi\|149058216 (+1) | 86 | 19 | 0 | 7 | 7 | 12 |
| 1110 | calcium binding protein 39 [Rattus norvegicus], gi\|149016314\|gb\|EDL75560.1\| calcium binding protein 39 (predicted), isoform CRA_a [Rattus norvegicus], gi\|149016315\|gb\|EDL75561.1\| calcium binding protein 39 (predicted), isoform CRA_a [Rattus norvegicus], g | gi\|157818343 | 24 | 22 | 1 | 0 | 0 | 22 |
| 1111 | RecName: Full=Cytosolic 5'-nucleotidase III-like protein; Short=cN-III-like protein, gi\|50926874\|gb\|AAH78963.1\| 5'-nucleotidase, cytosolic III-like [Rattus norvegicus] | gi\|81884635 | 34 | 10 | 15 | 0 | 5 | 5 |
| 1112 | PREDICTED: similar to ribosomal protein S13 [Rattus norvegicus] | gi\|109476367 (+2) | 18 | 20 | 0 | 0 | 0 | 20 |

| | | | | | | | | |
|---|---|---|---|---|---|---|---|---|
| 1113 | PREDICTED: similar to desmoplakin isoform I isoform 2 [Rattus norvegicus], gi\|109505631\|ref\|XP_001058477.1\| PREDICTED: similar to desmoplakin isoform I isoform 2 [Rattus norvegicus] | gi\|109504778 (+1) | 332 | 8 | 17 | 0 | 5 | 3 |
| 1114 | prefoldin subunit 2 [Rattus norvegicus], gi\|109499039\|ref\|XP_001055378.1\| PREDICTED: similar to Prefoldin subunit 2 isoform 2 [Rattus norvegicus], gi\|187470922\|sp\|B0BN18.1\|PFD2_RAT RecName: Full=Prefoldin subunit 2, gi\|149040686\|gb\|EDL94643.1\| rCG20392, i | gi\|157823115 | 17 | 20 | 4 | 0 | 1 | 19 |
| 1115 | protein phosphatase 3, regulatory subunit B, alpha isoform (calcineurin B, type I), isoform CRA_a [Rattus norvegicus] | gi\|149044720 (+3) | 18 | 26 | 0 | 0 | 0 | 26 |
| 1116 | acetoacetyl-CoA synthetase [Rattus norvegicus], gi\|81906806\|sp\|Q9JMI1.1\|AACS_RAT RecName: Full=Acetoacetyl-CoA synthetase, gi\|6996616\|dbj\|BAA90828.1\| acetoacetyl-CoA synthetase [Rattus norvegicus], gi\|38197367\|gb\|AAH61803.1\| Acetoacetyl-CoA synthetase [Ra | gi\|12831227 | 75 | 7 | 5 | 4 | 6 | 1 |
| 1117 | myo-inositol 1-phosphate synthase A1 [Rattus norvegicus], gi\|172045900\|sp\|Q6AYK3.2\|INO1_RAT RecName: Full=Inositol-3-phosphate synthase 1; AltName: Full=Myo-inositol-1-phosphate synthase; Short=MI-1-P synthase; Short=MIP synthase; Short=IPS, gi\|149036044\| | gi\|189163481 | 61 | 16 | 3 | 1 | 2 | 14 |
| 1118 | vacuolar protein sorting 28 homolog [Rattus norvegicus], gi\|149066083\|gb\|EDM15956.1\| vacuolar protein sorting 28 (yeast) (predicted), isoform CRA_c [Rattus norvegicus] | gi\|194473628 | 25 | 12 | 4 | 5 | 6 | 6 |
| 1119 | RecName: Full=Ras-related protein M-Ras; AltName: Full=Ras-related protein R-Ras3; Flags: Precursor, gi\|2209075\|dbj\|BAA20531.1\| M-Ras [Rattus norvegicus] | gi\|2851409 (+1) | 24 | 19 | 1 | 2 | 2 | 17 |
| 1120 | adenylosuccinate lyase [Rattus norvegicus], gi\|149065868\|gb\|EDM15741.1\| adenylosuccinate lyase (predicted) [Rattus norvegicus] | gi\|194473622 | 55 | 14 | 4 | 0 | 1 | 13 |
| 1121 | ADP-ribosylation factor-like 6 (predicted), isoform CRA_a [Rattus norvegicus], gi\|171846753\|gb\|AAI62029.1\| Arl6 protein [Rattus norvegicus] | gi\|149060273 | 21 | 15 | 7 | 0 | 2 | 13 |
| 1122 | PREDICTED: similar to Rap guanine nucleotide exchange factor (GEF) 1 isoform 3 [Rattus norvegicus] | gi\|109469690 (+1) | 144 | 10 | 6 | 1 | 3 | 7 |
| 1123 | solute carrier family 25 (mitochondrial carrier; adenine nucleotide translocator), member 3, isoform CRA_a [Rattus norvegicus] | gi\|149067210 (+2) | 35 | 16 | 0 | 8 | 8 | 8 |
| 1124 | RecName: Full=Ras-related protein Rab-31; AltName: Full=GTP-binding protein Rab0 | gi\|62900806 (+2) | 21 | 21 | 2 | 0 | 1 | 20 |
| 1125 | PREDICTED: similar to ubiquitin protein ligase E3C [Rattus norvegicus], gi\|109472884\|ref\|XP_001055148.1\| PREDICTED: similar to ubiquitin protein ligase E3C [Rattus norvegicus] | gi\|62646604 | 124 | 17 | 0 | 2 | 2 | 15 |
| 1126 | SEC23A [Mus musculus], gi\|157786714\|ref\|NP_001099202.1\| SEC23 homolog A [Rattus norvegicus], gi\|27735257\|sp\|Q01405.2\|SC23A_MOUSE RecName: Full=Protein transport protein Sec23A; AltName: Full=SEC23-related protein A, gi\|21961650\|gb\|AAH34610.1\| SEC23A (S. c | gi\|67906177 | 86 | 14 | 2 | 7 | 8 | 6 |
| 1127 | PREDICTED: similar to CG4996-PA [Rattus norvegicus], gi\|109473023\|ref\|XP_001064458.1\| PREDICTED: similar to CG4996-PA [Rattus norvegicus] | gi\|62646814 | 111 | 15 | 0 | 5 | 5 | 10 |
| 1128 | phosphatidylinositol transfer protein, beta [Rattus norvegicus], gi\|1709739\|sp\|P53812.2\|PIPNB_RAT RecName: Full=Phosphatidylinositol transfer protein beta isoform; Short=PtdIns transfer protein beta; Short=PtdInsTP; Short=PI-TP-beta, gi\|829055\|dbj\|BAA0466 | gi\|16758568 (+2) | 31 | 5 | 9 | 2 | 5 | 0 |
| 1129 | PREDICTED: similar to KIAA0999 protein [Rattus norvegicus] | gi\|109484648 | 143 | 8 | 1 | 5 | 5 | 3 |
| 1130 | erythrocyte protein band 4.1-like 3, isoform CRA_e [Rattus norvegicus] | gi\|149036265 (+1) | 103 | 14 | 3 | 5 | 6 | 8 |
| 1131 | hypothetical protein LOC362483 [Rattus norvegicus], gi\|81884534\|sp\|Q6AY71.1\|CH037_RAT RecName: Full=Uncharacterized protein C8orf37 homolog, gi\|50925747\|gb\|AAH79168.1\| Similar to RIKEN cDNA 2610301B20; EST AI428449 [Rattus norvegicus], gi\|149061056\|gb\|EDM | gi\|56090403 | 24 | 10 | 10 | 2 | 5 | 5 |
| 1132 | SNIP-a [Rattus norvegicus], gi\|149054044\|gb\|EDM05861.1\| SNAP25-interacting protein, isoform CRA_b [Rattus norvegicus] | gi\|6693834 (+1) | 127 | 11 | 1 | 5 | 5 | 6 |
| 1133 | tripartite motif-containing 67 [Rattus norvegicus], gi\|109509065\|ref\|XP_001054626.1\| PREDICTED: similar to tripartite motif-containing 9 [Rattus norvegicus] | gi\|208973282 | 83 | 16 | 0 | 5 | 5 | 11 |
| 1134 | rCG55995 [Rattus norvegicus] | gi\|149036433 | 25 | 21 | 0 | 3 | 3 | 18 |
| 1135 | PREDICTED: similar to armadillo repeat containing 8 [Rattus norvegicus] | gi\|62654169 | 75 | 15 | 0 | 3 | 3 | 12 |
| 1136 | similar to RIKEN cDNA 2810002D13 gene, isoform CRA_a [Rattus norvegicus] | gi\|149023060 (+1) | 26 | 14 | 4 | 0 | 1 | 13 |
| 1137 | glutathione S-transferase, theta 2 [Rattus norvegicus], gi\|1170118\|sp\|P30713.3\|GSTT2_RAT RecName: Full=Glutathione S-transferase theta-2; AltName: Full=GST class-theta-2; AltName: Full=Glutathione S-transferase 12; AltName: Full=GST 12-12; AltName: Full=G | gi\|6980992 | 27 | 12 | 9 | 0 | 3 | 9 |
| 1138 | ATP synthase, H+ transporting, mitochondrial F1 complex, O subunit, isoform CRA_c [Rattus norvegicus] | gi\|149059861 (+1) | 24 | 19 | 0 | 2 | 2 | 17 |
| 1139 | golgi associated, gamma adaptin ear containing, ARF binding protein 1 [Rattus norvegicus], gi\|58477207\|gb\|AAH90031.1\| Golgi associated, gamma adaptin ear containing, ARF binding protein 1 [Rattus norvegicus] | gi\|58865564 | 62 | 16 | 6 | 0 | 2 | 14 |
| 1140 | integrin alpha FG-GAP repeat containing 1 [Rattus norvegicus], gi\|55250565\|gb\|AAH85706.1\| Itfg1 protein [Rattus norvegicus], gi\|149032625\|gb\|EDL87495.1\| rCG44261, isoform CRA_b [Rattus norvegicus], gi\|149032627\|gb\|EDL87497.1\| rCG44261, isoform CRA_b [Ratt | gi\|148227578 | 67 | 20 | 0 | 0 | 0 | 20 |
| 1141 | PREDICTED: hypothetical protein [Rattus norvegicus], gi\|109469732\|ref\|XP_001079909.1\| PREDICTED: hypothetical protein [Rattus norvegicus], gi\|81884031\|sp\|Q63ZV7.1\|CI119_RAT RecName: Full=Uncharacterized protein C9orf119 homolog, gi\|52350647\|gb\|AAH82799.1\| | gi\|109468044 | 10 | 22 | 0 | 0 | 0 | 22 |
| 1142 | RecName: Full=cAMP-dependent protein kinase catalytic subunit alpha; Short=PKA C-alpha, gi\|56912\|emb\|CAA41052.1\| cAMP-dependent protein kinase subunit C alpha [Rattus rattus] | gi\|125207 (+1) | 41 | 14 | 2 | 7 | 8 | 6 |
| 1143 | oxysterol binding protein [Rattus norvegicus], gi\|149062479\|gb\|EDM12902.1\| oxysterol binding protein (predicted) [Rattus norvegicus] | gi\|157821663 | 78 | 15 | 2 | 4 | 5 | 10 |
| 1144 | PREDICTED: similar to bromodomain and WD repeat domain containing 2 [Rattus norvegicus], gi\|109462988\|ref\|XP_001080483.1\| PREDICTED: similar to bromodomain and WD repeat domain containing 2 [Rattus norvegicus] | gi\|109459473 | 136 | 18 | 0 | 6 | 6 | 12 |
| 1145 | PREDICTED: similar to Zinc finger protein 294 (Zfp-294) [Rattus norvegicus], gi\|109493855\|ref\|XP_001066804.1\| PREDICTED: similar to Zinc finger protein 294 (Zfp-294) [Rattus norvegicus] | gi\|109492780 | 202 | 10 | 5 | 3 | 5 | 5 |
| 1146 | actinin, alpha 1 [Rattus norvegicus], gi\|13123942\|sp\|Q9Z1P2.1\|ACTN1_RAT RecName: Full=Alpha-actinin-1; AltName: Full=Alpha-actinin cytoskeletal isoform; AltName: Full=Non-muscle alpha-actinin-1; AltName: Full=F-actin cross-linking protein, gi\|4210985\|gb\|A | gi\|13591902 (+2) | 103 | 9 | 14 | 1 | 5 | 4 |
| 1147 | protein phosphatase 4, regulatory subunit 1, isoform CRA_c [Rattus norvegicus] | gi\|149037380 (+2) | 104 | 14 | 5 | 1 | 3 | 11 |
| 1148 | phenylalanyl-tRNA synthetase, beta subunit [Rattus norvegicus], gi\|51260793\|gb\|AAH79364.1\| Phenylalanyl-tRNA synthetase, beta subunit [Rattus norvegicus] | gi\|51948478 | 66 | 16 | 0 | 6 | 6 | 10 |
| 1149 | hypothetical protein LOC310362 [Rattus norvegicus], gi\|149016523\|gb\|EDL75741.1\| similar to expressed sequence AW549877 (predicted) [Rattus norvegicus] | gi\|197385810 | 29 | 12 | 8 | 5 | 8 | 4 |
| 1150 | mesoderm specific transcript [Rattus norvegicus], gi\|81885227\|sp\|Q6P5P5.1\|MEST_RAT RecName: Full=Mesoderm-specific transcript homolog protein, gi\|38541065\|gb\|AAH62800.1\| Mesoderm specific transcript [Rattus norvegicus] | gi\|57528148 | 39 | 15 | 0 | 4 | 4 | 11 |
| 1151 | RAB4A, member RAS oncogene family [Rattus norvegicus], gi\|38303943\|gb\|AAH62016.1\| RAB4A, member RAS oncogene family [Rattus norvegicus] | gi\|77404180 | 24 | 12 | 8 | 0 | 3 | 9 |
| 1152 | tenascin R, isoform CRA_a [Rattus norvegicus] | gi\|149058290 (+1) | 139 | 15 | 0 | 7 | 7 | 8 |
| 1153 | RecName: Full=Protein DPCD, gi\|149040272\|gb\|EDL94310.1\| similar to CG13901-PA [Rattus norvegicus] | gi\|172045909 | 23 | 20 | 0 | 2 | 2 | 18 |
| 1154 | lysophospholipase 1 [Rattus norvegicus], gi\|41017250\|sp\|P70470.1\|LYPA1_RAT RecName: Full=Acyl-protein thioesterase 1; AltName: Full=Lysophospholipase 1; AltName: Full=Lysophospholipase I, gi\|1552244\|dbj\|BAA09935.1\| lysophospholipase [Rattus norvegicus], g | gi\|6981362 | 25 | 13 | 9 | 0 | 3 | 10 |
| 1155 | methylenetetrahydrofolate dehydrogenase 1 [Rattus norvegicus], gi\|1345633\|sp\|P27653.3\|C1TC_RAT RecName: Full=C-1-tetrahydrofolate synthase, cytoplasmic; Short=C1-THF synthase; Includes: RecName: Full=Methylenetetrahydrofolate dehydrogenase; Includes: RecN | gi\|11968082 (+3) | 101 | 14 | 0 | 5 | 5 | 9 |
| 1156 | nudix-type motif 3 [Rattus norvegicus], gi\|68565629\|sp\|Q566C7.1\|NUDT3_RAT RecName: Full=Diphosphoinositol polyphosphate phosphohydrolase 1; Short=DIPP-1; AltName: Full=Diadenosine 5',5'''-P1,P6-hexaphosphate hydrolase 1; AltName: Full=Nucleoside diphospha | gi\|66730447 | 19 | 6 | 15 | 0 | 5 | 1 |
| 1157 | alpha fetoprotein | gi\|228784 | 68 | 6 | 11 | 0 | 3 | 3 |
| 1158 | p38 mitogen-activated protein kinase alpha1 [Rattus norvegicus] | gi\|13194758 (+3) | 41 | 14 | 0 | 11 | 11 | 3 |
| 1159 | polymerase (RNA) II (DNA directed) polypeptide C [Mus musculus], gi\|60223073\|ref\|NP_001012491.1\| polymerase II polypeptide C [Rattus norvegicus], gi\|12805131\|gb\|AAH02023.1\| Polymerase (RNA) II (DNA directed) polypeptide C [Mus musculus], gi\|59808391\|gb\|AA | gi\|29336059 | 31 | 17 | 0 | 7 | 7 | 10 |
| 1160 | reticulocalbin 2 [Rattus norvegicus], gi\|606968\|gb\|AAA80197.1\| taipoxin-associated calcium binding protein-49 precursor | gi\|8394171 | 37 | 22 | 0 | 0 | 0 | 22 |
| 1161 | serine (or cysteine) peptidase inhibitor, clade A, member 6 [Rattus norvegicus], gi\|205371742\|sp\|P31211.2\|CBG_RAT RecName: Full=Corticosteroid-binding globulin; Short=CBG; AltName: Full=Transcortin; AltName: Full=Serpin A6; Flags: Precursor, gi\|56789211\|g | gi\|57527135 | 45 | 9 | 11 | 0 | 3 | 6 |
| 1162 | stathmin 1 [Rattus norvegicus], gi\|134974\|sp\|P13668.2\|STMN1_RAT RecName: Full=Stathmin; AltName: Full=Phosphoprotein p19; Short=pp19; AltName: Full=Leukemia-associated phosphoprotein p18; AltName: Full=Oncoprotein 18; Short=Op18; AltName: Full=pp17; AltNa | gi\|8393696 | 17 | 12 | 9 | 0 | 3 | 9 |
| 1163 | RNA polymerase II, polypeptide H [Homo sapiens], gi\|21704118\|ref\|NP_663607.1\| polymerase (RNA) II (DNA directed) polypeptide H [Mus musculus], gi\|198278428\|ref\|NP_001128261.1\| polymerase (RNA) II (DNA directed) polypeptide H [Rattus norvegicus], gi\|507523 | gi\|14589953 | 17 | 23 | 1 | 0 | 0 | 23 |
| 1164 | target of myb1 homolog (chicken), isoform CRA_a [Rattus norvegicus] | gi\|149032481 (+1) | 55 | 14 | 3 | 5 | 6 | 8 |
| 1165 | hypothetical protein LOC362115 [Rattus norvegicus], gi\|109469579\|ref\|XP_001078459.1\| PREDICTED: similar to niban protein [Rattus norvegicus], gi\|149038983\|gb\|EDL93203.1\| similar to RIKEN cDNA 9130404D14 [Rattus norvegicus], gi\|195539744\|gb\|AAI68248.1\| Fam | gi\|158081781 | 85 | 11 | 5 | 5 | 7 | 4 |
| 1166 | WD repeat domain 37 [Rattus norvegicus], gi\|149031448\|gb\|EDL86438.1\| WD repeat domain 37 (predicted), isoform CRA_a [Rattus norvegicus], gi\|149031449\|gb\|EDL86439.1\| WD repeat domain 37 (predicted), isoform CRA_a [Rattus norvegicus], gi\|149031450\|gb\|EDL864 | gi\|157822497 | 50 | 14 | 1 | 7 | 7 | 7 |
| 1167 | acyl-Coenzyme A binding domain containing 3 [Rattus norvegicus] | gi\|149040873 (+2) | 60 | 14 | 6 | 4 | 6 | 8 |
| 1168 | F-box protein 21 [Rattus norvegicus], gi\|149063492\|gb\|EDM13815.1\| F-box only protein 21 (predicted) [Rattus norvegicus] | gi\|157820503 | 73 | 16 | 1 | 5 | 5 | 11 |
| 1169 | elongation factor Tu GTP binding domain containing 1 [Rattus norvegicus], gi\|149057408\|gb\|EDM08731.1\| rCG24931 [Rattus norvegicus] | gi\|157820011 | 126 | 15 | 5 | 0 | 2 | 13 |
| 1170 | anaphase promoting complex subunit 2 [Rattus norvegicus], gi\|149039398\|gb\|EDL93618.1\| anaphase promoting complex subunit 2 [Rattus norvegicus], gi\|187469179\|gb\|AAI66796.1\| Anapc2 protein [Rattus norvegicus] | gi\|189217530 | 95 | 16 | 0 | 4 | 4 | 12 |
| 1171 | GRIP1 associated protein 1 [Rattus norvegicus], gi\|76363169\|sp\|Q9JHZ4.1\|GRAP1_RAT RecName: Full=GRIP1-associated protein 1; Short=GRASP-1, gi\|8980841\|gb\|AAF82298.1\|AF274057_1 GRIP-associated protein 1 long form [Rattus norvegicus], gi\|149028442\|gb\|EDL8382 | gi\|16758652 (+1) | 96 | 10 | 7 | 4 | 6 | 4 |
| 1172 | Vps24p protein [Rattus norvegicus], gi\|73917767\|sp\|Q8CGS4.3\|CHMP3_RAT RecName: Full=Charged multivesicular body protein 3; AltName: Full=Chromatin-modifying protein 3; AltName: Full=Vacuolar protein-sorting-associated protein 24; Short=rVps24p, gi\|2598965 | gi\|27229308 | 25 | 9 | 5 | 3 | 5 | 4 |

| | | | | | | | | |
|---|---|---|---|---|---|---|---|---|
| 1173 | Paf1, RNA polymerase II associated factor, homolog [Rattus norvegicus], gi\|81908653\|sp\|Q4V886.1\|PAF1_RAT RecName: Full=RNA polymerase II-associated factor 1 homolog, gi\|66911741\|gb\|AAH97494.1\| Paf1, RNA polymerase II associated factor, homolog (S. cerevis | gi\|67846072 | 61 | 18 | 0 | 5 | 5 | 13 |
| 1174 | mitogen activated protein kinase kinase 2, isoform CRA_a [Rattus norvegicus] | gi\|149034454 (+2) | 41 | 11 | 6 | 5 | 7 | 4 |
| 1175 | dachsous 1 [Rattus norvegicus], gi\|149068442\|gb\|EDM17994.1\| dachsous 1 (Drosophila) (predicted) [Rattus norvegicus] | gi\|157823899 | 345 | 4 | 8 | 3 | 6 | 0 |
| 1176 | translocase of outer mitochondrial membrane 34 [Rattus norvegicus], gi\|212275276\|ref\|NP_001130928.1\| hypothetical protein LOC100192033 [Zea mays], gi\|123780638\|sp\|Q3KRD5.1\|TOM34_RAT RecName: Full=Mitochondrial import receptor subunit TOM34; AltName: Full= | gi\|112984308 | 34 | 3 | 17 | 0 | 5 | 0 |
| 1177 | sorting nexin 3 isoform a [Homo sapiens], gi\|31560433\|ref\|NP_059500.2\| sorting nexin 3 [Mus musculus], gi\|112983968\|ref\|NP_001037748.1\| hypothetical protein LOC684097 [Rattus norvegicus], gi\|119331196\|ref\|NP_001073246.1\| sorting nexin 3 [Bos taurus], gi\|1 | gi\|4507143 | 19 | 14 | 3 | 0 | 1 | 13 |
| 1178 | DnaJ (Hsp40) homolog, subfamily C, member 13 [Rattus norvegicus], gi\|149018720\|gb\|EDL77361.1\| DnaJ (Hsp40) homolog, subfamily C, member 13 (predicted) [Rattus norvegicus] | gi\|157817568 | 207 | 12 | 0 | 8 | 8 | 4 |
| 1179 | abl-interactor 1 [Rattus norvegicus], gi\|125858961\|gb\|AAI29093.1\| Abi1 protein [Rattus norvegicus] | gi\|126723350 (+1) | 52 | 15 | 0 | 9 | 9 | 6 |
| 1180 | cyclin-dependent kinase 4 [Rattus norvegicus], gi\|543972\|sp\|P35426.1\|CDK4_RAT RecName: Full=Cell division protein kinase 4; AltName: Full=Cyclin-dependent kinase 4; AltName: Full=PSK-J3, gi\|203392\|gb\|AAA40903.1\| cyclin-dependent kinase 4, gi\|299368\|gb\|AAB | gi\|16758944 | 34 | 15 | 2 | 2 | 3 | 12 |
| 1181 | WD repeat domain 26 [Rattus norvegicus], gi\|149040896\|gb\|EDL94853.1\| similar to myocardial ischemic preconditioning upregulated protein 2 (predicted) [Rattus norvegicus] | gi\|157818723 | 59 | 16 | 0 | 4 | 4 | 12 |
| 1182 | PREDICTED: similar to zinc finger, ZZ type with EF hand domain 1 [Rattus norvegicus], gi\|109491246\|ref\|XP_001080236.1\| PREDICTED: similar to zinc finger, ZZ type with EF hand domain 1 [Rattus norvegicus] | gi\|109488501 | 332 | 12 | 0 | 4 | 4 | 8 |
| 1183 | laminin, alpha 1 [Rattus norvegicus], gi\|149036255\|gb\|EDL90914.1\| laminin, alpha 1 (predicted) [Rattus norvegicus] | gi\|157816959 | 198 | 20 | 0 | 2 | 2 | 18 |
| 1184 | SH3-domain GRB2-like B1 [Rattus norvegicus], gi\|81910882\|sp\|Q6AYE2.1\|SHLB1_RAT RecName: Full=Endophilin-B1; AltName: Full=SH3 domain-containing GRB2-like protein B1, gi\|50925669\|gb\|AAH79085.1\| SH3-domain GRB2-like endophilin B1 [Rattus norvegicus] | gi\|58865428 | 41 | 17 | 1 | 0 | 0 | 17 |
| 1185 | dephosphin short form - rat (fragments) | gi\|480368 | 8 | 14 | 0 | 5 | 5 | 9 |
| 1186 | discs, large homolog-associated protein 4 (Drosophila) [Rattus norvegicus] | gi\|149030812 (+1) | 108 | 21 | 2 | 0 | 1 | 20 |
| 1187 | nitrilase 1 isoform a [Rattus norvegicus], gi\|56268926\|gb\|AAH87146.1\| Nit1 protein [Rattus norvegicus], gi\|149040683\|gb\|EDL94640.1\| nitrilase 1, isoform CRA_b [Rattus norvegicus] | gi\|128485833 (+1) | 36 | 13 | 5 | 0 | 2 | 11 |
| 1188 | RNA helicase [Rattus norvegicus], gi\|81890303\|sp\|Q62780.1\|DDX46_RAT RecName: Full=Probable ATP-dependent RNA helicase DDX46; AltName: Full=DEAD box protein 46; AltName: Full=Helicase of 117.4 kDa, gi\|897915\|gb\|AAC52210.1\| RNA helicase, gi\|78174325\|gb\|AAI0 | gi\|20806137 | 117 | 16 | 0 | 3 | 3 | 13 |
| 1189 | vacuolar protein sorting 45 [Rattus norvegicus], gi\|23396892\|sp\|O08700.1\|VPS45_RAT RecName: Full=Vacuolar protein sorting-associated protein 45; Short=rvps45, gi\|2047326\|gb\|AAB53041.1\| rvps45 [Rattus norvegicus], gi\|51858559\|gb\|AAH81705.1\| Vacuolar protei | gi\|25742604 | 65 | 13 | 0 | 9 | 9 | 4 |
| 1190 | glia maturation factor, beta [Rattus norvegicus], gi\|2842657\|sp\|Q63228.2\|GMFB_RAT RecName: Full=Glia maturation factor beta; Short=GMF-beta, gi\|4379406\|emb\|CAA77650.1\| glia maturation factor beta [Rattus norvegicus], gi\|51859444\|gb\|AAH81778.1\| Glia matura | gi\|13624295 | 17 | 18 | 5 | 0 | 2 | 16 |
| 1191 | golgi SNAP receptor complex member 2 [Rattus norvegicus], gi\|146345427\|sp\|O35165.2\|GOSR2_RAT RecName: Full=Golgi SNAP receptor complex member 2; AltName: Full=27 kDa Golgi SNARE protein; AltName: Full=Membrin, gi\|1907388\|gb\|AAC53131.1\| membrin [Rattus nor | gi\|13928950 | 25 | 19 | 0 | 0 | 0 | 19 |
| 1192 | rCG56755, isoform CRA_b [Rattus norvegicus] | gi\|149031440 (+1) | 27 | 24 | 0 | 0 | 0 | 24 |
| 1193 | isoamyl acetate-hydrolyzing esterase 1 homolog [Rattus norvegicus], gi\|166226260\|sp\|Q711G3.2\|IAH1_RAT RecName: Full=Isoamyl acetate-hydrolyzing esterase 1 homolog; AltName: Full=Hypertrophic agonist-responsive protein B64, gi\|149050995\|gb\|EDM03168.1\| hype | gi\|198278545 | 28 | 10 | 8 | 0 | 3 | 7 |
| 1194 | mevalonate kinase [Rattus norvegicus], gi\|125406\|sp\|P17256.1\|KIME_RAT RecName: Full=Mevalonate kinase; Short=MK, gi\|20151083\|pdb\|1KVK\|A Chain A, The Structure Of Binary Complex Between A Mammalian Mevalonate Kinase And Atp: Insights Into The Reaction Mech | gi\|13592007 | 42 | 15 | 1 | 6 | 6 | 9 |
| 1195 | 26S proteasome subunit S5a [Rattus norvegicus], gi\|38051982\|gb\|AAH60559.1\| Psmd4 protein [Rattus norvegicus] | gi\|9957071 | 41 | 21 | 0 | 2 | 2 | 19 |
| 1196 | synaptotagmin 1 [Rattus norvegicus], gi\|94730428\|sp\|P21707.3\|SYT1_RAT RecName: Full=Synaptotagmin-1; AltName: Full=Synaptotagmin I; Short=SytI; AltName: Full=p65, gi\|39918764\|emb\|CAE85101.1\| synaptotagmin 1 [Rattus rattus], gi\|74229928\|gb\|ABA00482.1\| syna | gi\|148356226 (+3) | 47 | 15 | 0 | 6 | 6 | 9 |
| 1197 | exosome component 4 [Rattus norvegicus], gi\|109482347\|ref\|XP_001071925.1\| PREDICTED: similar to Exosome complex exonuclease RRP41 (Ribosomal RNA-processing protein 41) (Exosome component 4) (p12A) [Rattus norvegicus] | gi\|198442889 | 26 | 19 | 0 | 4 | 4 | 15 |
| 1198 | tubulin folding cofactor E [Rattus norvegicus], gi\|81909645\|sp\|Q5FVQ9.1\|TBCE_RAT RecName: Full=Tubulin-specific chaperone E; AltName: Full=Tubulin-folding cofactor E, gi\|58476460\|gb\|AAH89833.1\| Tubulin folding cofactor E [Rattus norvegicus], gi\|149032547\| | gi\|58865894 | 59 | 13 | 2 | 4 | 5 | 8 |
| 1199 | guanidinoacetate methyltransferase, isoform CRA_a [Rattus norvegicus] | gi\|149034566 (+1) | 26 | 5 | 13 | 0 | 4 | 1 |
| 1200 | kinesin light chain 4 [Rattus norvegicus], gi\|56268906\|gb\|AAH87116.1\| Kinesin light chain 4 [Rattus norvegicus], gi\|149069389\|gb\|EDM18830.1\| kinesin light chain 4, isoform CRA_a [Rattus norvegicus], gi\|149069390\|gb\|EDM18831.1\| kinesin light chain 4, isofo | gi\|57527498 | 69 | 11 | 0 | 3 | 3 | 8 |
| 1201 | syndecan binding protein [Rattus norvegicus], gi\|20455280\|sp\|Q9JI92.1\|SDCB1_RAT RecName: Full=Syntenin-1; AltName: Full=Syndecan-binding protein 1, gi\|9295415\|gb\|AAF86960.1\|AF248548_1 syntenin [Rattus norvegicus], gi\|12214237\|emb\|CAC21602.1\| syntenin-1 pr | gi\|14010891 | 32 | 10 | 7 | 3 | 5 | 5 |
| 1202 | active BCR-related [Rattus norvegicus], gi\|149053445\|gb\|EDM05262.1\| active BCR-related gene (predicted) [Rattus norvegicus] | gi\|157786654 | 98 | 13 | 2 | 6 | 7 | 6 |
| 1203 | Cd200 molecule [Rattus norvegicus], gi\|129300\|sp\|P04218.1\|OX2G_RAT RecName: Full=OX-2 membrane glycoprotein; AltName: Full=MRC OX-2 antigen; AltName: CD_antigen=CD200; Flags: Precursor, gi\|56701\|emb\|CAA25925.1\| MRC OX-2 antigen [Rattus norvegicus], gi\|149 | gi\|13928702 | 31 | 19 | 0 | 2 | 2 | 17 |
| 1204 | DnaJ (Hsp40) homolog, subfamily B, member 1 (predicted), isoform CRA_b [Rattus norvegicus], gi\|166796507\|gb\|AAI59431.1\| Dnajb1 protein [Rattus norvegicus] | gi\|149037907 | 38 | 15 | 0 | 4 | 4 | 11 |
| 1205 | eukaryotic translation initiation factor 2B, subunit 1 alpha [Rattus norvegicus], gi\|2494304\|sp\|Q64270.1\|EI2BA_RAT RecName: Full=Translation initiation factor eIF-2B subunit alpha; AltName: Full=eIF-2B GDP-GTP exchange factor subunit alpha, gi\|623033\|gb\|A | gi\|25282453 | 34 | 19 | 0 | 2 | 2 | 17 |
| 1206 | Chain A, Structure Of Fpt Bound To Inhibitor Sch207736 | gi\|114793517 (+5) | 38 | 19 | 0 | 2 | 2 | 17 |
| 1207 | sorting nexin 6 [Rattus norvegicus], gi\|149051241\|gb\|EDM03414.1\| sorting nexin 6 (predicted) [Rattus norvegicus], gi\|197246598\|gb\|AAI68856.1\| Sorting nexin 6 [Rattus norvegicus] | gi\|157823311 | 47 | 14 | 3 | 0 | 1 | 13 |
| 1208 | RAP2C, member of RAS oncogene family [Mus musculus], gi\|32129209\|ref\|NP_067006.3\| RAP2C, member of RAS oncogene family [Homo sapiens], gi\|115496672\|ref\|NP_001069168.1\| RAP2C, member of RAS oncogene family [Bos taurus], gi\|157819091\|ref\|NP_001100420.1\| RAP | gi\|27369539 | 21 | 17 | 0 | 0 | 0 | 17 |
| 1209 | heterogeneous nuclear ribonucleoprotein A2/B1 isoform A2 [Homo sapiens], gi\|114051756\|ref\|NP_001039440.1\| heterogeneous nuclear ribonucleoprotein A2/B1 [Bos taurus], gi\|73976082\|ref\|XP_863847.1\| PREDICTED: similar to heterogeneous nuclear ribonucleoprotei | gi\|4504447 | 36 | 12 | 0 | 5 | 5 | 7 |
| 1210 | related RAS viral (r-ras) oncogene homolog 2 isoform a [Homo sapiens], gi\|61740635\|ref\|NP_001013452.1\| related RAS viral (r-ras) oncogene homolog 2 [Rattus norvegicus], gi\|149944707\|ref\|NP_001092480.1\| related RAS viral (r-ras) oncogene homolog 2 [Bos tau | gi\|21361416 | 23 | 16 | 0 | 5 | 5 | 11 |
| 1211 | PREDICTED: similar to N-terminal acetyltransferase complex ARD1 subunit homolog A [Rattus norvegicus] | gi\|109462338 | 27 | 11 | 0 | 6 | 6 | 5 |
| 1212 | ribosomal protein L8 [Homo sapiens], gi\|6755358\|ref\|NP_036183.1\| ribosomal protein L8 [Mus musculus], gi\|15431306\|ref\|NP_150644.1\| ribosomal protein L8 [Homo sapiens], gi\|77736197\|ref\|NP_001029797.1\| ribosomal protein L8 [Bos taurus], gi\|78214309\|ref\|NP_0 | gi\|4506663 (+1) | 28 | 9 | 0 | 3 | 3 | 6 |
| 1213 | hypothetical protein LOC499782 [Rattus norvegicus], gi\|109510396\|ref\|XP_001056336.1\| PREDICTED: similar to 60S ribosomal protein L12 [Rattus norvegicus], gi\|109511446\|ref\|XP_001054256.1\| PREDICTED: similar to 60S ribosomal protein L12 [Rattus norvegicus], | gi\|157822227 | 18 | 20 | 2 | 0 | 1 | 19 |
| 1214 | magnesium-dependent phosphatase 1 [Rattus norvegicus], gi\|149063990\|gb\|EDM14260.1\| similar to magnesium-dependent phosphatase-1 (predicted), isoform CRA_b [Rattus norvegicus] | gi\|157823059 | 19 | 9 | 11 | 0 | 3 | 6 |
| 1215 | Trafficking protein particle complex 4 [Rattus norvegicus], gi\|149041469\|gb\|EDL95310.1\| rCG58353, isoform CRA_e [Rattus norvegicus] | gi\|71051670 | 24 | 12 | 0 | 7 | 7 | 5 |
| 1216 | retinoic acid induced 12 [Rattus norvegicus], gi\|81863783\|sp\|Q6IUP3.1\|DERP6_RAT RecName: Full=Dermal papilla-derived protein 6 homolog, gi\|48237488\|gb\|AAT40590.1\| DERP6 [Rattus norvegicus], gi\|149053128\|gb\|EDM04945.1\| dermal papilla derived protein 6, iso | gi\|48843729 | 35 | 19 | 0 | 2 | 2 | 17 |
| 1217 | sulfotransferase family 4A, member 1 [Rattus norvegicus], gi\|34328449\|ref\|NP_038901.3\| sulfotransferase family 4A, member 1 [Mus musculus], gi\|51703303\|sp\|P63047.1\|ST4A1_RAT RecName: Full=Sulfotransferase 4A1; AltName: Full=Brain sulfotransferase-like pro | gi\|13928882 (+1) | 33 | 14 | 6 | 0 | 2 | 12 |
| 1218 | amylo-1,6-glucosidase, 4-alpha-glucanotransferase [Rattus norvegicus], gi\|149025802\|gb\|EDL82045.1\| amylo-1, 6-glucosidase, 4-alpha-glucanotransferase (glycogen debranching enzyme, glycogen storage disease type III) (predicted), isoform CRA_a [Rattus norve | gi\|157819187 | 174 | 16 | 4 | 0 | 1 | 15 |
| 1219 | phospholipid hydroperoxide glutathione peroxidase [Rattus norvegicus], gi\|1585295\|prf\|\|2124383A phospholipid hydroperoxide glutathione peroxidase | gi\|1041645 | 19 | 16 | 4 | 0 | 1 | 15 |
| 1220 | similar to hypothetical protein (predicted) [Rattus norvegicus] | gi\|149021445 (+1) | 128 | 13 | 0 | 8 | 8 | 5 |
| 1221 | PREDICTED: similar to protein kinase C-binding protein NELL2 [Rattus norvegicus] | gi\|109482804 (+4) | 56 | 19 | 2 | 0 | 1 | 18 |
| 1222 | heterogeneous nuclear ribonucleoprotein U-like 1 [Rattus norvegicus], gi\|149056565\|gb\|EDM07996.1\| heterogeneous nuclear ribonucleoprotein U-like 1 (predicted) [Rattus norvegicus] | gi\|157824105 | 96 | 14 | 1 | 0 | 0 | 14 |
| 1223 | transmembrane protein 2 [Rattus norvegicus], gi\|149062586\|gb\|EDM13009.1\| transmembrane protein 2 (predicted) [Rattus norvegicus] | gi\|157817531 | 153 | 15 | 0 | 0 | 0 | 15 |
| 1224 | hydroxysteroid (17-beta) dehydrogenase 10 [Rattus norvegicus], gi\|6523787\|gb\|AAF14853.1\|AF069770_1 17beta-hydroxysteroid dehydrogenase type 10/short chain L-3-hydroxyacyl-CoA dehydrogenase [Rattus norvegicus], gi\|149031312\|gb\|EDL86310.1\| hydroxyacyl-Coenz | gi\|13994225 (+2) | 27 | 22 | 0 | 0 | 0 | 22 |
| 1225 | lipocortin I [Rattus sp.] | gi\|235879 (+1) | 39 | 18 | 2 | 0 | 1 | 17 |
| 1226 | PREDICTED: similar to riboflavin kinase [Rattus norvegicus], gi\|109470376\|ref\|XP_001079189.1\| PREDICTED: similar to riboflavin kinase [Rattus norvegicus] | gi\|109468486 (+1) | 25 | 21 | 2 | 0 | 1 | 20 |
| 1227 | Ab1-205 [Rattus norvegicus] | gi\|33086478 (+1) | 83 | 22 | 0 | 0 | 0 | 22 |

| | | | | | | | | |
|---|---|---|---|---|---|---|---|---|
| 1228 | cadherin 13 [Rattus norvegicus], gi\|20162485\|gb\|AAM14607.1\|AF494095_1 T-cadherin [Rattus norvegicus], gi\|55249666\|gb\|AAH85699.1\| Cadherin 13 [Rattus norvegicus], gi\|149038298\|gb\|EDL92658.1\| cadherin 13 [Rattus norvegicus] | gi\|20302073 | 78 | 10 | 1 | 1 | 1 | 9 |
| 1229 | protein tyrosine phosphatase, receptor type, f polypeptide (PTPRF), interacting protein (liprin), alpha 2 [Rattus norvegicus], gi\|149067045\|gb\|EDM16778.1\| protein tyrosine phosphatase, receptor type, f polypeptide (PTPRF), interacting protein (liprin), al | gi\|157824053 | 143 | 15 | 3 | 3 | 4 | 11 |
| 1230 | aminoadipate-semialdehyde dehydrogenase-phosphopantetheinyl transferase (predicted), isoform CRA_b [Rattus norvegicus], gi\|187469697\|gb\|AAI66800.1\| Aasdhppt protein [Rattus norvegicus] | gi\|149030154 | 36 | 7 | 9 | 2 | 5 | 2 |
| 1231 | PREDICTED: similar to ATP-binding cassette sub-family F member 1 (ATP-binding cassette 50) [Rattus norvegicus] | gi\|109509122 | 93 | 10 | 1 | 4 | 4 | 6 |
| 1232 | replication protein A1 [Rattus norvegicus] | gi\|149053388 | 65 | 8 | 12 | 2 | 6 | 2 |
| 1233 | WD repeat domain 54 [Rattus norvegicus], gi\|149036510\|gb\|EDL91128.1\| similar to D3Mm3e (predicted), isoform CRA_a [Rattus norvegicus] | gi\|157823545 | 36 | 10 | 3 | 3 | 4 | 6 |
| 1234 | PREDICTED: similar to ADAMTS-9 precursor (A disintegrin and metalloproteinase with thrombospondin motifs 9) (ADAM-TS 9) (ADAM-TS9) [Rattus norvegicus] | gi\|109471763 (+1) | 184 | 18 | 0 | 2 | 2 | 16 |
| 1235 | phosducin-like 3 [Rattus norvegicus], gi\|110815908\|sp\|Q4KLJ8.1\|PDCL3_RAT RecName: Full=Phosducin-like protein 3, gi\|68533843\|gb\|AAH99162.1\| Phosducin-like 3 [Rattus norvegicus], gi\|149046312\|gb\|EDL99205.1\| phosducin-like 3, isoform CRA_b [Rattus norvegicu | gi\|71043634 | 28 | 16 | 0 | 4 | 4 | 12 |
| 1236 | exosome component 8 (predicted), isoform CRA_a [Rattus norvegicus] | gi\|149064770 | 30 | 15 | 2 | 3 | 4 | 11 |
| 1237 | Mannosidase, alpha, class 2C, member 1 [Rattus norvegicus] | gi\|56540863 | 116 | 12 | 0 | 5 | 5 | 7 |
| 1238 | histone acetyltransferase 1 [Rattus norvegicus], gi\|81883142\|sp\|Q5M939.1\|HAT1_RAT RecName: Full=Histone acetyltransferase type B catalytic subunit, gi\|56585068\|gb\|AAH87663.1\| Histone acetyltransferase 1 [Rattus norvegicus] | gi\|57527084 | 49 | 12 | 3 | 2 | 3 | 9 |
| 1239 | PREDICTED: hypothetical protein [Rattus norvegicus] | gi\|109464286 (+1) | 73 | 9 | 4 | 5 | 6 | 3 |
| 1240 | general transcription factor IIB [Rattus norvegicus], gi\|21704076\|ref\|NP_663521.1\| general transcription factor IIB [Mus musculus], gi\|51703316\|sp\|P62916.1\|TF2B_RAT RecName: Full=Transcription initiation factor IIB; AltName: Full=General transcription fac | gi\|13591965 | 35 | 12 | 6 | 4 | 6 | 6 |
| 1241 | four and a half LIM domains 1 isoform 1 [Rattus norvegicus], gi\|38512114\|gb\|AAH61782.1\| Four and a half LIM domains 1 [Rattus norvegicus], gi\|149015816\|gb\|EDL75140.1\| four and a half LIM domains 1, isoform CRA_b [Rattus norvegicus] | gi\|76781465 (+1) | 34 | 11 | 3 | 3 | 4 | 7 |
| 1242 | eukaryotic translation initiation factor 3, subunit 5 (epsilon) (predicted), isoform CRA_c [Rattus norvegicus], gi\|149068378\|gb\|EDM17930.1\| eukaryotic translation initiation factor 3, subunit 5 (epsilon) (predicted), isoform CRA_e [Rattus norvegicus] | gi\|149068376 | 26 | 11 | 0 | 1 | 1 | 10 |
| 1243 | unc-119 homolog B [Rattus norvegicus], gi\|149063583\|gb\|EDM13906.1\| similar to Unc-119 protein homolog (Retinal protein 4) (RRG4) (predicted), isoform CRA_a [Rattus norvegicus] | gi\|157786930 | 28 | 13 | 0 | 5 | 5 | 8 |
| 1244 | glycerophosphodiester phosphodiesterase domain containing 1 [Rattus norvegicus], gi\|115502209\|sp\|Q0VGK4.1\|GDPD1_RAT RecName: Full=Glycerophosphodiester phosphodiesterase domain-containing protein 1, gi\|111493981\|gb\|AAI05621.1\| Glycerophosphodiester phosph | gi\|112982914 | 36 | 17 | 0 | 1 | 1 | 16 |
| 1245 | type II keratin Kb15 [Rattus norvegicus], gi\|46485029\|tpg\|DAA02236.1\| TPA_exp: type II keratin Kb15 [Rattus norvegicus] | gi\|114145409 (+1) | 58 | 13 | 9 | 0 | 3 | 10 |
| 1246 | rCG30218, isoform CRA_a [Rattus norvegicus] | gi\|149049362 | 29 | 13 | 2 | 0 | 1 | 12 |
| 1247 | calcium modulating ligand [Rattus norvegicus], gi\|10644766\|gb\|AAG21394.1\|AF302085_1 calcium-modulating cyclophilin ligand [Rattus norvegicus] | gi\|16758044 (+1) | 33 | 17 | 0 | 3 | 3 | 14 |
| 1248 | lethal, Chr 7, Rinchik 6 [Mus musculus], gi\|81906188\|sp\|Q9DD02.1\|CK073_MOUSE RecName: Full=Uncharacterized protein C11orf73 homolog; AltName: Full=Lethal gene on chromosome 7 Rinchik 6 protein, gi\|12832191\|dbj\|BAB22001.1\| unnamed protein product [Mus musc | gi\|13399318 | 22 | 17 | 4 | 0 | 1 | 16 |
| 1249 | similar to Dual specificity protein phosphatase 3 (T-DSP11) (predicted), isoform CRA_b [Rattus norvegicus] | gi\|149054346 (+1) | 23 | 10 | 9 | 0 | 3 | 7 |
| 1250 | ubiquilin 4 [Rattus norvegicus], gi\|149048123\|gb\|EDM00699.1\| ubiquilin 4 (predicted) [Rattus norvegicus] | gi\|157818715 | 64 | 17 | 0 | 1 | 1 | 16 |
| 1251 | Ras-related GTP binding A [Homo sapiens], gi\|16758868\|ref\|NP_446425.1\| Ras-related GTP-binding protein ragA [Rattus norvegicus], gi\|31541819\|ref\|NP_848463.1\| Ras-related GTP binding A [Mus musculus], gi\|78369202\|ref\|NP_001030576.1\| Ras-related GTP binding | gi\|5729999 | 37 | 20 | 0 | 1 | 1 | 19 |
| 1252 | hypothetical protein LOC310358 [Rattus norvegicus], gi\|149016519\|gb\|EDL75737.1\| similar to RIKEN cDNA B130016O10 gene (predicted) [Rattus norvegicus] | gi\|157818229 | 36 | 9 | 10 | 0 | 3 | 6 |
| 1253 | rCG32122, isoform CRA_a [Rattus norvegicus] | gi\|149042824 (+1) | 36 | 15 | 3 | 0 | 1 | 14 |
| 1254 | sepiapterin reductase [Rattus norvegicus], gi\|134847\|sp\|P18297.1\|SPRE_RAT RecName: Full=Sepiapterin reductase; Short=SPR, gi\|149036562\|gb\|EDL91180.1\| rCG56371 [Rattus norvegicus], gi\|187469059\|gb\|AAI66809.1\| Sepiapterin reductase (7,8-dihydrobiopterin:NAD | gi\|158711683 (+1) | 28 | 5 | 13 | 0 | 4 | 1 |
| 1255 | sideroflexin 3 [Rattus norvegicus], gi\|20139873\|sp\|Q9JHY2.1\|SFXN3_RAT RecName: Full=Sideroflexin-3, gi\|8650526\|gb\|AAF78249.1\|AF276997_1 tricarboxylate carrier-like protein [Rattus norvegicus] | gi\|12621120 (+2) | 35 | 13 | 0 | 5 | 5 | 8 |
| 1256 | rCG45476, isoform CRA_a [Rattus norvegicus] | gi\|149039237 (+2) | 30 | 12 | 0 | 5 | 5 | 7 |
| 1257 | rCG44226 [Rattus norvegicus] | gi\|149045109 | 51 | 11 | 0 | 9 | 9 | 2 |
| 1258 | eukaryotic translation initiation factor 4E [Rattus norvegicus], gi\|83627717\|ref\|NP_031943.3\| eukaryotic translation initiation factor 4E [Mus musculus], gi\|52001071\|sp\|P63074.1\|IF4E_RAT RecName: Full=Eukaryotic translation initiation factor 4E; Short=eIF | gi\|16758870 | 25 | 10 | 4 | 0 | 1 | 9 |
| 1259 | 3-oxoacid CoA transferase 1 [Rattus norvegicus], gi\|205829936\|sp\|B2GV06.1\|SCOT1_RAT RecName: Full=Succinyl-CoA:3-ketoacid-coenzyme A transferase 1, mitochondrial; AltName: Full=3-oxoacid-CoA transferase 1; AltName: Full=Somatic-type succinyl CoA:3-oxoacid | gi\|189181716 | 56 | 19 | 0 | 0 | 0 | 19 |
| 1260 | cytochrome b5 type B (outer mitochondrial membrane) [Rattus norvegicus], gi\|12643974\|sp\|P04166.2\|CYB5B_RAT RecName: Full=Cytochrome b5 type B; AltName: Full=Cytochrome b5 outer mitochondrial membrane isoform; Flags: Precursor, gi\|2253161\|emb\|CAA73117.1\| c | gi\|13399338 | 16 | 22 | 0 | 0 | 0 | 22 |
| 1261 | transmembrane protein 103 [Rattus norvegicus], gi\|206557823\|sp\|B2RYG8.1\|CC075_RAT RecName: Full=UPF0405 protein C3orf75 homolog; AltName: Full=Protein TMEM103, gi\|149018446\|gb\|EDL77087.1\| transmembrane protein 103 (predicted) [Rattus norvegicus], gi\|18746 | gi\|157817755 | 29 | 21 | 0 | 0 | 0 | 21 |
| 1262 | hepatoma-derived growth factor [Rattus norvegicus], gi\|52788205\|sp\|Q8VHK7.2\|HDGF_RAT RecName: Full=Hepatoma-derived growth factor; Short=HDGF, gi\|14718640\|gb\|AAK72966.1\|AF389348_1 HDGF [Rattus norvegicus], gi\|47477847\|gb\|AAH70943.1\| Hepatoma-derived growt | gi\|16758528 | 26 | 0 | 20 | 0 | 6 | 0 |
| 1263 | PREDICTED: similar to ribosomal protein S15a [Rattus norvegicus], gi\|109462843\|ref\|XP_001079709.1\| PREDICTED: similar to ribosomal protein S15a [Rattus norvegicus] | gi\|109459378 (+2) | 14 | 16 | 0 | 0 | 0 | 16 |
| 1264 | phosphoprotein enriched in astrocytes 15 [Homo sapiens], gi\|61557370\|ref\|NP_001013249.1\| phosphoprotein enriched in astrocytes 15A [Rattus norvegicus], gi\|115495869\|ref\|NP_001068924.1\| phosphoprotein enriched in astrocytes 15 [Bos taurus], gi\|197100238\|re | gi\|4505705 | 15 | 22 | 0 | 0 | 0 | 22 |
| 1265 | glomulin, FKBP associated protein [Rattus norvegicus], gi\|149028634\|gb\|EDL83975.1\| FKBP-associated protein, isoform CRA_a [Rattus norvegicus], gi\|149028635\|gb\|EDL83976.1\| FKBP-associated protein, isoform CRA_a [Rattus norvegicus] | gi\|157821959 | 68 | 12 | 2 | 4 | 5 | 7 |
| 1266 | RAB3B, member RAS oncogene family [Rattus norvegicus], gi\|6093900\|sp\|Q63941.2\|RAB3B_RAT RecName: Full=Ras-related protein Rab-3B, gi\|2251196\|emb\|CAA74341.1\| Rab3B protein [Rattus norvegicus], gi\|149035702\|gb\|EDL90383.1\| RAB3B, member RAS oncogene family, | gi\|13592037 | 25 | 10 | 5 | 3 | 5 | 5 |
| 1267 | inositol polyphosphate-4-phosphatase, type 1 [Rattus norvegicus], gi\|73920061\|sp\|Q62784.1\|INP4A_RAT RecName: Full=Type I inositol-3,4-bisphosphate 4-phosphatase; AltName: Full=Inositol polyphosphate 4-phosphatase type I, gi\|944913\|gb\|AAB01069.1\| inositol | gi\|215272386 | 106 | 12 | 3 | 5 | 6 | 6 |
| 1268 | signal transducing adaptor molecule (SH3 domain and ITAM motif) 1 [Rattus norvegicus], gi\|149021124\|gb\|EDL78731.1\| rCG55706 [Rattus norvegicus], gi\|197246851\|gb\|AAI68933.1\| Signal transducing adaptor molecule (SH3 domain and ITAM motif) 1 [Rattus norvegic | gi\|157819901 | 60 | 13 | 2 | 1 | 2 | 11 |
| 1269 | fat mass and obesity associated [Rattus norvegicus], gi\|123779274\|sp\|Q2A121.1\|FTO_RAT RecName: Full=Protein fto; AltName: Full=Fat mass and obesity-associated protein, gi\|89179153\|emb\|CAJ80872.1\| Fatso protein [Rattus norvegicus], gi\|195539738\|gb\|AAI68239 | gi\|89337260 | 58 | 7 | 9 | 2 | 5 | 2 |
| 1270 | activated leukocyte cell adhesion molecule [Rattus norvegicus], gi\|47605356\|sp\|O35112.1\|CD166_RAT RecName: Full=CD166 antigen; AltName: Full=Activated leukocyte cell adhesion molecule; AltName: Full=Protein MEMD; AltName: Full=HB2; AltName: Full=SB-10 ant | gi\|13929058 (+1) | 65 | 17 | 0 | 2 | 2 | 15 |
| 1271 | Rho GTPase activating protein 1 (predicted), isoform CRA_b [Rattus norvegicus], gi\|149022644\|gb\|EDL79538.1\| Rho GTPase activating protein 1 (predicted), isoform CRA_b [Rattus norvegicus] | gi\|149022642 | 32 | 7 | 4 | 3 | 4 | 3 |
| 1272 | PREDICTED: similar to N-ethylmaleimide sensitive fusion protein attachment protein beta [Rattus norvegicus], gi\|109470977\|ref\|XP_001057340.1\| PREDICTED: similar to N-ethylmaleimide sensitive fusion protein attachment protein beta [Rattus norvegicus] | gi\|62645998 | 40 | 14 | 1 | 0 | 0 | 14 |
| 1273 | hypothetical protein LOC366311 [Rattus norvegicus], gi\|149061042\|gb\|EDM11652.1\| rCG30484, isoform CRA_a [Rattus norvegicus], gi\|149061044\|gb\|EDM11654.1\| rCG30484, isoform CRA_a [Rattus norvegicus], gi\|149061045\|gb\|EDM11655.1\| rCG30484, isoform CRA_a [Ratt | gi\|157822747 | 41 | 6 | 10 | 1 | 4 | 2 |
| 1274 | guanosine monophosphate reductase 2 [Rattus norvegicus] | gi\|149063995 (+1) | 38 | 13 | 0 | 1 | 1 | 12 |
| 1275 | translocated promoter region [Rattus norvegicus] | gi\|149058426 | 191 | 10 | 7 | 3 | 5 | 5 |
| 1276 | exocyst complex component 8 [Rattus norvegicus], gi\|81886761\|sp\|O54924.1\|EXOC8_RAT RecName: Full=Exocyst complex component 8; AltName: Full=Exocyst complex 84 kDa subunit, gi\|2827164\|gb\|AAC01581.1\| exo84 [Rattus norvegicus], gi\|149043214\|gb\|EDL96746.1\| ex | gi\|20514778 | 81 | 14 | 0 | 1 | 1 | 13 |
| 1277 | rCG62645, isoform CRA_b [Rattus norvegicus], gi\|165971324\|gb\|AAI58877.1\| Apoa1bp protein [Rattus norvegicus] | gi\|149048164 (+1) | 31 | 10 | 9 | 0 | 3 | 7 |
| 1278 | SEC22 vesicle trafficking protein-like 1 [Mus musculus], gi\|71043604\|ref\|NP_001020857.1\| SEC22 vesicle trafficking protein homolog B [Rattus norvegicus], gi\|197101735\|ref\|NP_001125720.1\| SEC22 vesicle trafficking protein homolog B [Pongo abelii], gi\|10901 | gi\|6755448 | 25 | 14 | 2 | 4 | 5 | 9 |
| 1279 | PREDICTED: similar to leucine rich repeat containing 4B [Rattus norvegicus], gi\|109461847\|ref\|XP_001077685.1\| PREDICTED: similar to leucine rich repeat containing 4B [Rattus norvegicus] | gi\|34856020 | 76 | 17 | 0 | 0 | 0 | 17 |
| 1280 | ribosomal protein L9 [Rattus norvegicus], gi\|133033\|sp\|P17077.1\|RL9_RAT RecName: Full=60S ribosomal protein L9, gi\|57706\|emb\|CAA36002.1\| unnamed protein product [Rattus rattus], gi\|55824704\|gb\|AAH86561.1\| Ribosomal protein L9 [Rattus norvegicus], gi\|14903 | gi\|145207974 (+2) | 22 | 15 | 0 | 6 | 6 | 9 |
| 1281 | kalirin, RhoGEF kinase [Rattus norvegicus], gi\|160380715\|sp\|P97924.3\|KALRN_RAT RecName: Full=Kalirin; AltName: Full=Huntingtin-associated protein-interacting protein; AltName: Full=Protein Duo; AltName: Full=Serine/threonine kinase with Dbl- and pleckstri | gi\|14091744 (+1) | 337 | 13 | 1 | 3 | 3 | 10 |
| 1282 | phosphatidylinositol glycan anchor biosynthesis, class S [Rattus norvegicus], gi\|62286959\|sp\|Q5XI31.3\|PIGS_RAT RecName: Full=GPI transamidase component PIG-S; AltName: Full=Phosphatidylinositol-glycan biosynthesis class S protein, gi\|54035333\|gb\|AAH83862. | gi\|54633340 | 62 | 17 | 0 | 3 | 3 | 14 |

| | | | | | | | | |
|---|---|---|---|---|---|---|---|---|
| 1283 | hypothetical protein LOC292139 [Rattus norvegicus], gi\|156637367\|sp\|A0MZ67.1\|SHOT1_RAT RecName: Full=Shootin-1, gi\|117667954\|gb\|ABK56023.1\| shootin2 [Rattus norvegicus], gi\|149040514\|gb\|EDL94552.1\| similar to 4930506M07Rik protein (predicted), isoform CRA | gi\|119508806 | 71 | 14 | 0 | 0 | 0 | 14 |
| 1284 | hypothetical protein LOC289400 [Rattus norvegicus], gi\|149058504\|gb\|EDM09661.1\| similar to KIAA1078 protein (predicted) [Rattus norvegicus] | gi\|197386168 | 166 | 4 | 5 | 3 | 5 | 0 |
| 1285 | hypothetical protein LOC308568 [Rattus norvegicus], gi\|38014715\|gb\|AAH60592.1\| Similar to RIKEN cDNA 2410002F23 [Rattus norvegicus] | gi\|62078767 | 28 | 16 | 0 | 5 | 5 | 11 |
| 1286 | ribophorin II, isoform CRA_a [Rattus norvegicus] | gi\|149043116 (+1) | 65 | 19 | 0 | 0 | 0 | 19 |
| 1287 | syntaxin 6 [Rattus norvegicus], gi\|2501094\|sp\|Q63635.1\|STX6_RAT RecName: Full=Syntaxin-6, gi\|1488683\|gb\|AAC52709.1\| syntaxin 6, gi\|51858907\|gb\|AAH81769.1\| Syntaxin 6 [Rattus norvegicus] | gi\|13928922 | 29 | 16 | 0 | 4 | 4 | 12 |
| 1288 | malic enzyme 1 [Rattus norvegicus] | gi\|149018984 (+2) | 55 | 18 | 0 | 0 | 0 | 18 |
| 1289 | PREDICTED: similar to TATA-binding protein-associated factor 172 (ATP-dependent helicase BTAF1) (TBP-associated factor 172) (TAF-172) (TAF(II)170) (B-TFIID transcription factor-associated 170 kDa subunit) [Rattus norvegicus], gi\|109463837\|ref\|XP_001080159 | gi\|109460086 | 207 | 4 | 15 | 0 | 5 | 0 |
| 1290 | cell adhesion molecule 4 [Rattus norvegicus], gi\|123778954\|sp\|Q1WIM1.1\|CADM4_RAT RecName: Full=Cell adhesion molecule 4; AltName: Full=Immunoglobulin superfamily member 4C; AltName: Full=Nectin-like protein 4; Flags: Precursor, gi\|82619338\|gb\|ABB85364.1\| | gi\|114052915 | 43 | 19 | 0 | 0 | 0 | 19 |
| 1291 | MOB1, Mps One Binder kinase activator-like 2A [Rattus norvegicus], gi\|149034510\|gb\|EDL89247.1\| MOB1, Mps One Binder kinase activator-like 2B (yeast) (predicted), isoform CRA_a [Rattus norvegicus], gi\|149034512\|gb\|EDL89249.1\| MOB1, Mps One Binder kinase ac | gi\|157823747 | 26 | 11 | 1 | 0 | 0 | 11 |
| 1292 | histone deacetylase 2 [Rattus norvegicus], gi\|149032950\|gb\|EDL87791.1\| histone deacetylase 2 [Rattus norvegicus] | gi\|208022663 | 55 | 12 | 0 | 7 | 7 | 5 |
| 1293 | FERM, RhoGEF (Arhgef) and pleckstrin domain protein 1 (chondrocyte-derived) [Rattus norvegicus], gi\|149050235\|gb\|EDM02559.1\| FERM, RhoGEF (Arhgef) and pleckstrin domain protein 1 (chondrocyte-derived) (predicted) [Rattus norvegicus] | gi\|157822917 | 119 | 9 | 0 | 3 | 3 | 6 |
| 1294 | Ras homolog enriched in brain [Rattus norvegicus], gi\|57097443\|ref\|XP_532768.1\| PREDICTED: similar to GTP-binding protein Rheb (Ras homolog enriched in brain) [Canis familiaris], gi\|194666609\|ref\|XP_001787150.1\| PREDICTED: hypothetical protein [Bos taurus | gi\|6981476 | 20 | 15 | 2 | 0 | 1 | 14 |
| 1295 | PRA1 domain family, member 2 [Rattus norvegicus], gi\|149028448\|gb\|EDL83833.1\| PRA1 domain family 2 (predicted) [Rattus norvegicus] | gi\|157823972 | 19 | 17 | 0 | 0 | 0 | 17 |
| 1296 | ubiquitin-conjugating enzyme E2N [Rattus norvegicus], gi\|77417616\|sp\|Q9EQX9.1\|UBE2N_RAT RecName: Full=Ubiquitin-conjugating enzyme E2 N; AltName: Full=Ubiquitin-protein ligase N; AltName: Full=Ubiquitin carrier protein N; AltName: Full=Bendless-like ubiqu | gi\|16758810 | 17 | 8 | 6 | 0 | 2 | 6 |
| 1297 | RecName: Full=G-protein coupled receptor 56; Flags: Precursor, gi\|22255986\|gb\|AAM94855.1\| G protein-coupled receptor 56 [Rattus norvegicus] | gi\|45476764 | 77 | 20 | 0 | 0 | 0 | 20 |
| 1298 | isocitrate dehydrogenase 3 (NAD+) alpha, isoform CRA_a [Rattus norvegicus] | gi\|149041699 (+4) | 41 | 19 | 0 | 0 | 0 | 19 |
| 1299 | BTB (POZ) domain containing 17 [Rattus norvegicus], gi\|149054729\|gb\|EDM06546.1\| similar to CG1841-PA, isoform A (predicted), isoform CRA_b [Rattus norvegicus] | gi\|197385182 | 55 | 4 | 0 | 4 | 4 | 0 |
| 1300 | saccharopine dehydrogenase (putative) [Rattus norvegicus], gi\|73919297\|sp\|Q6AY30.1\|SCPDH_RAT RecName: Full=Probable saccharopine dehydrogenase, gi\|50925783\|gb\|AAH79215.1\| Saccharopine dehydrogenase (putative) [Rattus norvegicus], gi\|149040855\|gb\|EDL94812. | gi\|62078699 | 47 | 20 | 0 | 0 | 0 | 20 |
| 1301 | 5'-AMP-activated protein kinase, gamma-1 subunit | gi\|1335860 (+2) | 37 | 12 | 4 | 1 | 2 | 10 |
| 1302 | rCG47917, isoform CRA_b [Rattus norvegicus] | gi\|149062107 (+1) | 82 | 12 | 4 | 2 | 3 | 9 |
| 1303 | dihydroxyacetone kinase 2 homolog [Rattus norvegicus], gi\|85718638\|sp\|Q4KLZ6.1\|DHAK_RAT RecName: Full=Bifunctional ATP-dependent dihydroxyacetone kinase/FAD-AMP lyase (cyclizing); Includes: RecName: Full=ATP-dependent dihydroxyacetone kinase; Short=DHA ki | gi\|84781664 | 59 | 13 | 5 | 0 | 2 | 11 |
| 1304 | cofactor of BRCA1 [Rattus norvegicus], gi\|149039415\|gb\|EDL93635.1\| similar to cofactor of BRCA1; negative elongation factor protein B (predicted), isoform CRA_b [Rattus norvegicus] | gi\|165377254 | 66 | 13 | 1 | 3 | 3 | 10 |
| 1305 | vacuolar protein sorting 37 homolog B [Rattus norvegicus], gi\|149063285\|gb\|EDM13608.1\| vacuolar protein sorting 37B (yeast) (predicted), isoform CRA_b [Rattus norvegicus] | gi\|157786918 | 31 | 15 | 2 | 3 | 4 | 11 |
| 1306 | RAB, member of RAS oncogene family-like 4 [Rattus norvegicus], gi\|149066017\|gb\|EDM15890.1\| RAB, member of RAS oncogene family-like 4 (predicted) [Rattus norvegicus], gi\|195539923\|gb\|AAI68150.1\| Rabl4 protein [Rattus norvegicus] | gi\|194473634 | 21 | 12 | 4 | 0 | 1 | 11 |
| 1307 | similar to KIAA2010 protein (predicted), isoform CRA_b [Rattus norvegicus] | gi\|149025363 | 94 | 8 | 8 | 3 | 6 | 2 |
| 1308 | ribosomal protein L5 [Rattus norvegicus], gi\|1173056\|sp\|P09895.3\|RL5_RAT RecName: Full=60S ribosomal protein L5, gi\|57125\|emb\|CAA29506.1\| unnamed protein product [Rattus norvegicus], gi\|38014831\|gb\|AAH60561.1\| Ribosomal protein L5 [Rattus norvegicus], gi\| | gi\|13592051 (+1) | 34 | 10 | 3 | 6 | 7 | 3 |
| 1309 | NOL1/NOP2/Sun domain family, member 2 [Rattus norvegicus], gi\|149032756\|gb\|EDL87611.1\| NOL1/NOP2/Sun domain family, member 2 (predicted) [Rattus norvegicus] | gi\|157822221 | 88 | 7 | 4 | 4 | 5 | 2 |
| 1310 | ribophorin I, isoform CRA_a [Rattus norvegicus] | gi\|149036687 (+2) | 68 | 16 | 0 | 1 | 1 | 15 |
| 1311 | guanine nucleotide binding protein (G protein), beta polypeptide 4 [Rattus norvegicus], gi\|62906844\|sp\|O35353.4\|GBB4_RAT RecName: Full=Guanine nucleotide-binding protein subunit beta-4; AltName: Full=Transducin beta chain 4, gi\|45359810\|gb\|AAS59142.1\| G-p | gi\|62078551 | 37 | 14 | 0 | 5 | 5 | 9 |
| 1312 | stromal cell derived factor receptor 1, isoform CRA_a [Rattus norvegicus] | gi\|149041842 (+4) | 44 | 14 | 0 | 1 | 1 | 13 |
| 1313 | target of myb1-like 2 (chicken) (predicted), isoform CRA_b [Rattus norvegicus], gi\|149052820\|gb\|EDM04637.1\| target of myb1-like 2 (chicken) (predicted), isoform CRA_b [Rattus norvegicus] | gi\|149052819 (+1) | 31 | 11 | 4 | 3 | 4 | 7 |
| 1314 | podocalyxin-like 2 [Rattus norvegicus], gi\|149036702\|gb\|EDL91320.1\| podocalyxin-like 2 (predicted) [Rattus norvegicus], gi\|171847413\|gb\|AAI61970.1\| Podocalyxin-like 2 [Rattus norvegicus] | gi\|157819739 | 65 | 16 | 1 | 1 | 1 | 15 |
| 1315 | heterogeneous nuclear ribonucleoprotein A/B [Rattus norvegicus], gi\|6911221\|gb\|AAF31437.1\|AF216753_1 CArG-binding factor A [Rattus norvegicus], gi\|6562847\|emb\|CAB62554.1\| heterogeneous nuclear ribonucleoprotein; type A/B hnRNP p38 [Rattus norvegicus], gi\| | gi\|140971918 (+3) | 31 | 9 | 10 | 0 | 3 | 6 |
| 1316 | PREDICTED: similar to calmodulin regulated spectrin-associated protein 1 [Rattus norvegicus], gi\|109469613\|ref\|XP_001078134.1\| PREDICTED: similar to calmodulin regulated spectrin-associated protein 1 [Rattus norvegicus] | gi\|109467903 | 179 | 2 | 8 | 2 | 5 | 0 |
| 1317 | down-regulator of transcription 1 [Rattus norvegicus], gi\|62900752\|sp\|Q5XI68.1\|NC2B_RAT RecName: Full=Protein Dr1; AltName: Full=Down-regulator of transcription 1; AltName: Full=TATA-binding protein-associated phosphoprotein; AltName: Full=Negative cofact | gi\|58865406 | 19 | 9 | 6 | 0 | 2 | 7 |
| 1318 | acyl-Coenzyme A binding domain containing 6 [Rattus norvegicus], gi\|81889400\|sp\|Q5RJK8.1\|ACBD6_RAT RecName: Full=Acyl-CoA-binding domain-containing protein 6, gi\|55778318\|gb\|AAH86598.1\| Acyl-Coenzyme A binding domain containing 6 [Rattus norvegicus], gi\|1 | gi\|58865390 | 31 | 11 | 3 | 2 | 3 | 8 |
| 1319 | protein tyrosine phosphatase 4a1 [Rattus norvegicus] | gi\|17528929 (+3) | 20 | 16 | 3 | 0 | 1 | 15 |
| 1320 | La ribonucleoprotein domain family, member 7 [Rattus norvegicus], gi\|53733605\|gb\|AAH83898.1\| La ribonucleoprotein domain family, member 7 [Rattus norvegicus] | gi\|112983842 (+1) | 56 | 19 | 0 | 0 | 0 | 19 |
| 1321 | eukaryotic translation termination factor 1 [Homo sapiens], gi\|56605766\|ref\|NP_001008345.1\| eukaryotic translation termination factor 1 [Rattus norvegicus], gi\|115495753\|ref\|NP_001069722.1\| eukaryotic translation termination factor 1 [Bos taurus], gi\|1242 | gi\|4759034 (+1) | 49 | 11 | 5 | 0 | 2 | 9 |
| 1322 | deleted in colorectal carcinoma, isoform CRA_b [Rattus norvegicus] | gi\|149064590 (+1) | 150 | 14 | 4 | 0 | 1 | 13 |
| 1323 | AKT interacting protein [Rattus norvegicus], gi\|58476597\|gb\|AAH89985.1\| Fused toes [Rattus norvegicus], gi\|149032679\|gb\|EDL87549.1\| rCG44356, isoform CRA_b [Rattus norvegicus], gi\|149032680\|gb\|EDL87550.1\| rCG44356, isoform CRA_b [Rattus norvegicus] | gi\|58865430 | 33 | 6 | 0 | 3 | 3 | 3 |
| 1324 | breast carcinoma amplified sequence 2 (predicted), isoform CRA_c [Rattus norvegicus] | gi\|149030454 (+1) | 24 | 16 | 0 | 4 | 4 | 12 |
| 1325 | YKT6 v-SNARE homolog [Rattus norvegicus], gi\|81870672\|sp\|Q5EGY4.1\|YKT6_RAT RecName: Full=Synaptobrevin homolog YKT6, gi\|58758764\|gb\|AAW81771.1\| SNARE protein Ykt6 [Rattus norvegicus], gi\|149047657\|gb\|EDM00327.1\| rCG35885, isoform CRA_a [Rattus norvegicus] | gi\|162138924 | 22 | 12 | 6 | 0 | 2 | 10 |
| 1326 | ubiquitin specific protease 10 [Rattus norvegicus] | gi\|149038330 (+1) | 87 | 9 | 0 | 3 | 3 | 6 |
| 1327 | serine/threonine kinase 25 [Rattus norvegicus], gi\|89337277\|ref\|NP_067512.3\| serine/threonine kinase 25 [Mus musculus], gi\|33943091\|gb\|AAQ55284.1\| serine/threonine kinase 25 [Rattus norvegicus], gi\|47937402\|gb\|AAH71218.1\| Serine/threonine kinase 25 (yeast | gi\|34576555 | 48 | 12 | 0 | 5 | 5 | 7 |
| 1328 | transmembrane emp24 protein transport domain containing 9 [Rattus norvegicus], gi\|56970465\|gb\|AAH88422.1\| Transmembrane emp24 protein transport domain containing 9 [Rattus norvegicus], gi\|149039848\|gb\|EDL93964.1\| transmembrane emp24 protein transport doma | gi\|57528337 | 27 | 17 | 2 | 0 | 1 | 16 |
| 1329 | strawberry notch homolog 1 [Rattus norvegicus], gi\|149063266\|gb\|EDM13589.1\| sno, strawberry notch homolog 1 (Drosophila), isoform CRA_a [Rattus norvegicus] | gi\|157821077 | 137 | 2 | 11 | 2 | 5 | 0 |
| 1330 | ribosomal protein L10a [Rattus norvegicus], gi\|15431288\|ref\|NP_009035.3\| ribosomal protein L10a [Homo sapiens], gi\|62751847\|ref\|NP_001015647.1\| ribosomal protein L10a [Bos taurus], gi\|73972648\|ref\|XP_863955.1\| PREDICTED: similar to ribosomal protein L10a | gi\|13592009 | 25 | 15 | 0 | 0 | 0 | 15 |
| 1331 | RecName: Full=Prothrombin; AltName: Full=Coagulation factor II; Contains: RecName: Full=Activation peptide fragment 1; Contains: RecName: Full=Activation peptide fragment 2; Contains: RecName: Full=Thrombin light chain; Contains: RecName: Full=Thrombin he | gi\|135809 (+1) | 70 | 2 | 15 | 0 | 5 | 0 |
| 1332 | resistance to inhibitors of cholinesterase 8 homolog A [Rattus norvegicus], gi\|149061518\|gb\|EDM11941.1\| resistance to inhibitors of cholinesterase 8 homolog A (C. elegans) [Rattus norvegicus], gi\|169642109\|gb\|AAI60852.1\| Ric8a protein [Rattus norvegicus] | gi\|198278525 | 60 | 6 | 4 | 6 | 7 | 0 |
| 1333 | gelsolin, isoform CRA_a [Rattus norvegicus] | gi\|149038928 (+2) | 86 | 2 | 13 | 0 | 4 | 0 |
| 1334 | cortactin, isoform CRA_c [Rattus norvegicus] | gi\|149061813 | 61 | 16 | 1 | 0 | 0 | 16 |
| 1335 | protein kinase, interferon inducible double stranded RNA dependent activator, isoform CRA_b [Rattus norvegicus] | gi\|149022336 (+1) | 34 | 10 | 0 | 6 | 6 | 4 |
| 1336 | protein kinase C, iota [Rattus norvegicus] | gi\|149048611 | 56 | 7 | 0 | 4 | 4 | 3 |
| 1337 | CTTNBP2 N-terminal like [Rattus norvegicus], gi\|149030408\|gb\|EDL85445.1\| CTTNBP2 N-terminal like (predicted), isoform CRA_a [Rattus norvegicus] | gi\|164565360 | 70 | 16 | 0 | 0 | 0 | 16 |
| 1338 | unnamed protein product [Rattus norvegicus] | gi\|55628 | 69 | 5 | 11 | 0 | 3 | 2 |
| 1339 | PREDICTED: similar to CG8379-PA, isoform A [Rattus norvegicus], gi\|109463946\|ref\|XP_001058595.1\| PREDICTED: similar to CG8379-PA, isoform A [Rattus | gi\|109460219 | 79 | 13 | 0 | 2 | 2 | 11 |

| | | | | | | | | |
|---|---|---|---|---|---|---|---|---|
| | norvegicus], gi\|149040289\|gb\|EDL94327.1\| rCG57700, isoform CRA_a [Rattus norvegicus] | | | | | | | |
| 1340 | collagen-binding factor Endo180 [Rattus norvegicus] | gi\|149054516 (+1) | 171 | 16 | 0 | 0 | 0 | 16 |
| 1341 | insulin-like growth factor 2 mRNA binding protein 1 [Rattus norvegicus], gi\|81866340\|sp\|Q8CGX0.1\|IF2B1_RAT RecName: Full=Insulin-like growth factor 2 mRNA-binding protein 1; Short=IGF2 mRNA-binding protein 1; Short=IMP-1; AltName: Full=IGF-II mRNA-binding | gi\|28212254 | 63 | 14 | 0 | 5 | 5 | 9 |
| 1342 | protein kinase C substrate 80K-H [Rattus norvegicus], gi\|149020438\|gb\|EDL78243.1\| protein kinase C substrate 80K-H (predicted), isoform CRA_b [Rattus norvegicus], gi\|171846648\|gb\|AAI61987.1\| Protein kinase C substrate 80K-H [Rattus norvegicus] | gi\|157818781 | 59 | 14 | 0 | 0 | 0 | 14 |
| 1343 | ubiquitin-conjugating enzyme E2I [Homo sapiens], gi\|6981690\|ref\|NP_037182.1\| ubiquitin-conjugating enzyme E2I [Rattus norvegicus], gi\|7549799\|ref\|NP_035795.1\| ubiquitin-conjugating enzyme E2I [Mus musculus], gi\|35493987\|ref\|NP_919235.1\| ubiquitin-conjugat | gi\|4507785 | 18 | 1 | 12 | 0 | 4 | 0 |
| 1344 | protein kinase (EC 2.7.1.37) raf - rat, gi\|206547\|gb\|AAA42002.1\| raf fusion protein | gi\|66766 | 69 | 6 | 7 | 1 | 3 | 3 |
| 1345 | PREDICTED: similar to secreted frizzled-related sequence protein 1 [Rattus norvegicus] | gi\|109503534 (+2) | 50 | 14 | 2 | 0 | 1 | 13 |
| 1346 | rCG33606, isoform CRA_b [Rattus norvegicus], gi\|149054023\|gb\|EDM05840.1\| rCG33606, isoform CRA_b [Rattus norvegicus] | gi\|149054022 (+1) | 47 | 13 | 4 | 1 | 2 | 11 |
| 1347 | rCG52996, isoform CRA_b [Rattus norvegicus] | gi\|149060331 (+1) | 38 | 1 | 8 | 0 | 3 | 0 |
| 1348 | rCG55411, isoform CRA_a [Rattus norvegicus] | gi\|149037422 (+1) | 134 | 14 | 0 | 1 | 1 | 13 |
| 1349 | guanine nucleotide binding protein-like 1 [Rattus norvegicus], gi\|81872343\|sp\|Q6MG06.1\|GNL1_RAT RecName: Full=Guanine nucleotide-binding protein-like 1, gi\|46237666\|emb\|CAE84041.1\| guanine nucleotide binding protein-like 1 [Rattus norvegicus], gi\|12585875 | gi\|47059187 (+1) | 69 | 10 | 2 | 3 | 4 | 6 |
| 1350 | myeloid leukemia factor 2 homolog [Mus musculus], gi\|157823873\|ref\|NP_001101359.1\| myeloid leukemia factor 2 [Rattus norvegicus], gi\|17368615\|sp\|Q99KX1.1\|MLF2_MOUSE RecName: Full=Myeloid leukemia factor 2; AltName: Full=Myelodysplasia-myeloid leukemia fac | gi\|21703770 | 28 | 9 | 2 | 2 | 3 | 6 |
| 1351 | similar to phospholysine phosphohistidine inorganic pyrophosphate phosphata (5M590) [Rattus norvegicus] | gi\|149061303 (+1) | 27 | 7 | 9 | 0 | 3 | 4 |
| 1352 | rCG21481, isoform CRA_a [Rattus norvegicus] | gi\|149063739 (+1) | 167 | 4 | 6 | 2 | 4 | 0 |
| 1353 | metallophosphoesterase domain containing 2 [Rattus norvegicus], gi\|149022845\|gb\|EDL79739.1\| putative C11orf8 homolog (human), isoform CRA_a [Rattus norvegicus], gi\|149022847\|gb\|EDL79741.1\| putative C11orf8 homolog (human), isoform CRA_a [Rattus norvegicus | gi\|158186724 | 33 | 6 | 11 | 0 | 3 | 3 |
| 1354 | RNA polymerase II associated protein 1 [Rattus norvegicus], gi\|123780902\|sp\|Q3T1I9.1\|RPAP1_RAT RecName: Full=RNA polymerase II-associated protein 1 | gi\|77404259 | 155 | 15 | 3 | 0 | 1 | 14 |
| 1355 | PREDICTED: similar to axonemal dynein light chain 1 [Rattus norvegicus], gi\|149025110\|gb\|EDL81477.1\| rCG20672 [Rattus norvegicus] | gi\|109479639 | 17 | 4 | 10 | 0 | 3 | 1 |
| 1356 | RecName: Full=UBX domain-containing protein 1; AltName: Full=SAPK substrate protein 1, gi\|149062320\|gb\|EDM12743.1\| similar to hypothetical protein MGC6696, isoform CRA_b [Rattus norvegicus], gi\|149062322\|gb\|EDM12745.1\| similar to hypothetical protein MGC6 | gi\|114152149 | 34 | 11 | 5 | 1 | 3 | 8 |
| 1357 | keratin 14 [Rattus norvegicus], gi\|81170667\|sp\|Q6IFV1.1\|K1C14_RAT RecName: Full=Keratin, type I cytoskeletal 14; AltName: Full=Cytokeratin-14; Short=CK-14; AltName: Full=Keratin-14; Short=K14; AltName: Full=Type I keratin Ka14, gi\|46485088\|tpg\|DAA04481.1\| | gi\|56912233 | 53 | 13 | 5 | 0 | 2 | 11 |
| 1358 | ribosomal protein L18 [Rattus norvegicus], gi\|132733\|sp\|P12001.2\|RL18_RAT RecName: Full=60S ribosomal protein L18, gi\|206724\|gb\|AAA42070.1\| ribosomal protein L18, gi\|54261673\|gb\|AAH84727.1\| Ribosomal protein L18 [Rattus norvegicus], gi\|149055876\|gb\|EDM073 | gi\|13592057 (+2) | 22 | 10 | 0 | 8 | 8 | 2 |
| 1359 | ubiquitin-conjugating enzyme E2G 1 [Homo sapiens], gi\|25742608\|ref\|NP_073181.1\| ubiquitin-conjugating enzyme E2G 1 [Rattus norvegicus], gi\|27754105\|ref\|NP_080261.2\| ubiquitin-conjugating enzyme E2G 1 [Mus musculus], gi\|124249326\|ref\|NP_001074352.1\| ubiqui | gi\|13489085 | 20 | 11 | 6 | 0 | 2 | 9 |
| 1360 | MAP/microtubule affinity-regulating kinase 1 [Rattus norvegicus], gi\|62510707\|sp\|O08678.1\|MARK1_RAT RecName: Full=Serine/threonine-protein kinase MARK1; AltName: Full=MAP/microtubule affinity-regulating kinase 1, gi\|2052189\|emb\|CAB06294.1\| serine/threonin | gi\|16758824 | 88 | 7 | 0 | 5 | 5 | 2 |
| 1361 | Chain A, Nmr Solution Structure Of Ca2+-Loaded Calbindin D28k, gi\|110591321\|pdb\|2F33\|A Chain A, Nmr Solution Structure Of Ca2+-Loaded Calbindin D28k | gi\|110590202 (+2) | 30 | 9 | 7 | 0 | 2 | 7 |
| 1362 | calcium regulated heat stable protein 1 [Rattus norvegicus], gi\|41016763\|sp\|Q9WU49.1\|CHSP1_RAT RecName: Full=Calcium-regulated heat stable protein 1; AltName: Full=Calcium-regulated heat-stable protein of 24 kDa; Short=CRHSP-24, gi\|4583309\|gb\|AAD25022.1\|A | gi\|22758142 | 16 | 5 | 10 | 0 | 3 | 2 |
| 1363 | acetylserotonin O-methyltransferase-like (predicted), isoform CRA_a [Rattus norvegicus] | gi\|149035096 | 14 | 8 | 9 | 0 | 3 | 5 |
| 1364 | transmembrane emp24 domain trafficking protein 2 [Rattus norvegicus], gi\|229577019\|ref\|NP_001153290.1\| hypothetical protein LOC100140040 [Bos taurus], gi\|73994416\|ref\|XP_857949.1\| PREDICTED: similar to coated vesicle membrane protein isoform 2 [Canis fami | gi\|13929014 | 23 | 14 | 0 | 0 | 0 | 14 |
| 1365 | neuroligin 3 [Rattus norvegicus], gi\|149042192\|gb\|EDL95899.1\| neuroligin 3, isoform CRA_a [Rattus norvegicus] | gi\|145966694 (+1) | 94 | 15 | 0 | 0 | 0 | 15 |
| 1366 | transgelin 2, isoform CRA_b [Rattus norvegicus] | gi\|149040761 (+1) | 30 | 2 | 15 | 0 | 5 | 0 |
| 1367 | CDP-diacylglycerol-inositol 3-phosphatidyltransferase [Rattus norvegicus], gi\|7674155\|sp\|P70500.1\|CDIPT_RAT RecName: Full=CDP-diacylglycerol--inositol 3-phosphatidyltransferase; AltName: Full=Phosphatidylinositol synthase; Short=PtdIns synthase; Short=PI | gi\|20302093 | 24 | 19 | 0 | 0 | 0 | 19 |
| 1368 | dolichyl-diphosphooligosaccharide-protein glycosyltransferase [Rattus norvegicus], gi\|81884080\|sp\|Q641Y0.1\|OST48_RAT RecName: Full=Dolichyl-diphosphooligosaccharide--protein glycosyltransferase 48 kDa subunit; Short=Oligosaccharyl transferase 48 kDa subun | gi\|58865778 | 49 | 14 | 0 | 0 | 0 | 14 |
| 1369 | protein phosphatase 1, catalytic subunit, gamma isoform [Rattus norvegicus], gi\|27807051\|ref\|NP_777006.1\| protein phosphatase 1, catalytic subunit, gamma isoform [Bos taurus], gi\|31980772\|ref\|NP_038664.2\| protein phosphatase 1, catalytic subunit, gamma is | gi\|11968062 (+1) | 37 | 9 | 5 | 1 | 3 | 6 |
| 1370 | doublecortin kinase-2 [Rattus norvegicus] | gi\|56269585 (+2) | 78 | 8 | 4 | 4 | 5 | 3 |
| 1371 | PREDICTED: similar to RNA-binding protein Nova-2 (Neuro-oncological ventral antigen 2) (Astrocytic NOVA1-like RNA-binding protein) [Rattus norvegicus] | gi\|109461339 | 56 | 12 | 0 | 3 | 3 | 9 |
| 1372 | Rho-associated coiled-coil containing protein kinase 2 [Rattus norvegicus], gi\|47605938\|sp\|Q62868.1\|ROCK2_RAT RecName: Full=Rho-associated protein kinase 2; AltName: Full=Rho-associated, coiled-coil-containing protein kinase 2; AltName: Full=p164 ROCK-2; | gi\|6981478 | 159 | 10 | 2 | 2 | 3 | 7 |
| 1373 | kinesin family member 1B [Rattus norvegicus], gi\|84028216\|sp\|O88658.2\|KIF1B_RAT RecName: Full=Kinesin-like protein KIF1B, gi\|19911181\|dbj\|BAB86917.1\| kinesin-family protein 1Bp204 [Rattus norvegicus] | gi\|29789307 | 204 | 12 | 3 | 2 | 3 | 9 |
| 1374 | protein kinase, cAMP dependent regulatory, type I, alpha [Rattus norvegicus], gi\|125196\|sp\|P09456.2\|KAP0_RAT RecName: Full=cAMP-dependent protein kinase type I-alpha regulatory subunit, gi\|206673\|gb\|AAB54276.1\| R-I subunit, gi\|149054655\|gb\|EDM06472.1\| pro | gi\|6981396 | 43 | 9 | 3 | 0 | 1 | 8 |
| 1375 | phosphoribosyl transferase domain containing 1 (predicted), isoform CRA_d [Rattus norvegicus] | gi\|149021191 (+1) | 22 | 11 | 5 | 0 | 2 | 9 |
| 1376 | MOB1, Mps One Binder kinase activator-like 1A [Homo sapiens], gi\|62412947\|ref\|NP_081011.1\| MOB1, Mps One Binder kinase activator-like 1A [Mus musculus], gi\|157820997\|ref\|NP_001101827.1\| MOB1, Mps One Binder kinase activator-like 1A [Rattus norvegicus], gi | gi\|27735029 | 25 | 8 | 4 | 0 | 1 | 7 |
| 1377 | PREDICTED: similar to density-regulated protein isoform 1 [Rattus norvegicus] | gi\|109495842 (+2) | 22 | 9 | 6 | 0 | 2 | 7 |
| 1378 | rCG47614, isoform CRA_f [Rattus norvegicus] | gi\|149061760 (+1) | 18 | 11 | 2 | 1 | 2 | 9 |
| 1379 | golgi associated, gamma adaptin ear containing, ARF binding protein 3 [Rattus norvegicus], gi\|149054778\|gb\|EDM06595.1\| golgi associated, gamma adaptin ear containing, ARF binding protein 3 (predicted) [Rattus norvegicus] | gi\|157819547 | 72 | 9 | 2 | 3 | 4 | 5 |
| 1380 | similar to RIKEN cDNA 4930429A22 (predicted), isoform CRA_c [Rattus norvegicus] | gi\|149016362 (+1) | 100 | 3 | 7 | 3 | 5 | 0 |
| 1381 | eukaryotic translation initiation factor 2B, subunit 2 beta, isoform CRA_d [Rattus norvegicus] | gi\|149025192 (+3) | 34 | 13 | 0 | 3 | 3 | 10 |
| 1382 | peroxiredoxin 4, isoform CRA_a [Rattus norvegicus] | gi\|149042395 (+1) | 32 | 12 | 0 | 0 | 0 | 12 |
| 1383 | c-K-ras2 protein [Rattus norvegicus], gi\|495534\|gb\|AAB60458.1\| p21, gi\|116487990\|gb\|AAI26087.1\| V-Ki-ras2 Kirsten rat sarcoma viral oncogene homolog [Rattus norvegicus], gi\|149048985\|gb\|EDM01439.1\| v-Ki-ras2 Kirsten rat sarcoma viral oncogene homolog [Rat | gi\|13928698 | 21 | 10 | 0 | 4 | 4 | 6 |
| 1384 | rCG57812, isoform CRA_a [Rattus norvegicus] | gi\|149040344 (+1) | 141 | 3 | 7 | 2 | 4 | 0 |
| 1385 | phosphodiesterase 1B [Rattus norvegicus], gi\|231819\|sp\|Q01066.1\|PDE1B_RAT RecName: Full=Calcium/calmodulin-dependent 3',5'-cyclic nucleotide phosphodiesterase 1B; Short=Cam-PDE 1B; AltName: Full=63 kDa Cam-PDE, gi\|13195526\|gb\|AAK15740.1\|AF327906_1 phospho | gi\|12083681 (+1) | 61 | 5 | 6 | 3 | 5 | 0 |
| 1386 | RNA binding motif protein 39 [Rattus norvegicus], gi\|60552170\|gb\|AAH91394.1\| RNA binding motif protein 39 [Rattus norvegicus], gi\|74196119\|dbj\|BAE32977.1\| unnamed protein product [Mus musculus], gi\|149030834\|gb\|EDL85861.1\| RNA-binding region (RNP1, RRM) c | gi\|61557287 (+1) | 59 | 12 | 1 | 5 | 5 | 7 |
| 1387 | RAB23, member RAS oncogene family [Rattus norvegicus], gi\|149046425\|gb\|EDL99318.1\| RAB23, member RAS oncogene family (predicted) [Rattus norvegicus] | gi\|157823749 | 27 | 15 | 2 | 0 | 1 | 14 |
| 1388 | ubiquitin specific protease 47 (predicted), isoform CRA_a [Rattus norvegicus] | gi\|149068272 | 19 | 2 | 11 | 2 | 5 | 0 |
| 1389 | PREDICTED: similar to Charged multivesicular body protein 4b (Chromatin-modifying protein 4b) (CHMP4b) [Rattus norvegicus], gi\|109472868\|ref\|XP_001054829.1\| PREDICTED: similar to Charged multivesicular body protein 4b (Chromatin-modifying protein 4b) (CHM | gi\|109469032 | 25 | 9 | 8 | 1 | 4 | 5 |
| 1390 | t-complex 11 like 1 [Rattus norvegicus], gi\|149022804\|gb\|EDL79698.1\| similar to RIKEN cDNA C130096D04 gene (predicted) [Rattus norvegicus] | gi\|157822343 | 56 | 10 | 6 | 0 | 2 | 8 |
| 1391 | RecName: Full=Adenosine kinase; Short=AK; AltName: Full=Adenosine 5'-phosphotransferase | gi\|122065124 (+3) | 40 | 3 | 13 | 0 | 4 | 0 |
| 1392 | hypothetical protein LOC365627 [Rattus norvegicus], gi\|149058942\|gb\|EDM09949.1\| similar to metallo-beta-lactamase superfamily protein like (XL884) (predicted) [Rattus norvegicus] | gi\|157821863 | 31 | 15 | 0 | 0 | 0 | 15 |
| 1393 | synapsin II, isoform CRA_a [Rattus norvegicus] | gi\|149049703 (+1) | 63 | 13 | 0 | 0 | 0 | 13 |
| 1394 | hemoglobin:SUBUNIT=epsilon1 | gi\|1587328 (+1) | 16 | 17 | 1 | 0 | 0 | 17 |
| 1395 | neurofascin, isoform CRA_a [Rattus norvegicus] | gi\|149058628 (+8) | 135 | 11 | 0 | 0 | 0 | 11 |
| 1396 | DNA directed RNA polymerase II polypeptide G [Homo sapiens], gi\|13385826\|ref\|NP_080605.1\| polymerase (RNA) II (DNA directed) polypeptide G [Mus musculus], gi\|16758826\|ref\|NP_446400.1\| polymerase (RNA) II (DNA directed) polypeptide G [Rattus norvegicus], g | gi\|4505947 | 19 | 13 | 2 | 0 | 1 | 12 |
| 1397 | ribosomal protein, large, P1 [Rattus norvegicus], gi\|133053\|sp\|P19944.1\|RLA1_RAT RecName: Full=60S acidic ribosomal protein P1, gi\|57710\|emb\|CAA33200.1\| unnamed protein product [Rattus rattus], gi\|34786057\|gb\|AAH58151.1\| Ribosomal protein, large, P1 [Ratt | gi\|56090277 | 11 | 16 | 1 | 0 | 0 | 16 |
| 1398 | complement component 1, q subcomponent binding protein [Rattus norvegicus], gi\|122065146\|sp\|O35796.2\|C1QBP_RAT RecName: Full=Complement component 1 Q subcomponent-binding protein, mitochondrial; AltName: Full=Glycoprotein gC1qBP; Short=C1qBP; AltName: Ful | gi\|48675371 | 31 | 18 | 0 | 0 | 0 | 18 |
| 1399 | kinesin family member 5A [Rattus norvegicus] | gi\|149066613 | 106 | 10 | 5 | 2 | 4 | 6 |

<table>
<tr><td></td><td></td><td></td><td></td><td></td><td></td><td></td><td></td><td></td></tr>
<tr><td>1400</td><td>acyl-CoA synthetase long-chain family member 3 [Rattus norvegicus], gi|13431600|sp|Q63151.1|ACSL3_RAT RecName: Full=Long-chain-fatty-acid--CoA ligase 3; AltName: Full=Long-chain acyl-CoA synthetase 3; Short=LACS 3; AltName: Full=Brain acyl-CoA synthetase</td><td>gi|16923952</td><td>80</td><td>9</td><td>0</td><td>2</td><td>2</td><td>7</td></tr>
<tr><td>1401</td><td>p32-subunit of replication protein A [Rattus norvegicus]</td><td>gi|1403534 (+1)</td><td>29</td><td>7</td><td>2</td><td>2</td><td>3</td><td>4</td></tr>
<tr><td>1402</td><td>crystallin, zeta (quinone reductase)-like 1 [Rattus norvegicus], gi|54035324|gb|AAH83853.1| Crystallin, zeta (quinone reductase)-like 1 [Rattus norvegicus]</td><td>gi|61556783</td><td>39</td><td>8</td><td>4</td><td>3</td><td>4</td><td>4</td></tr>
<tr><td>1403</td><td>ATG7 autophagy related 7 homolog [Rattus norvegicus], gi|62286472|sp|Q641Y5.1|ATG7_RAT RecName: Full=Autophagy-related protein 7; AltName: Full=APG7-like; AltName: Full=Ubiquitin-activating enzyme E1-like protein, gi|51980522|gb|AAH82059.1| ATG7 autophagy</td><td>gi|58865764</td><td>77</td><td>10</td><td>2</td><td>2</td><td>3</td><td>7</td></tr>
<tr><td>1404</td><td>carbonyl reductase [Rattus norvegicus]</td><td>gi|5032250 (+1)</td><td>7</td><td>9</td><td>6</td><td>2</td><td>4</td><td>5</td></tr>
<tr><td>1405</td><td>kinesin family member 3B [Rattus norvegicus], gi|149030979|gb|EDL86006.1| kinesin family member 3B (predicted) [Rattus norvegicus]</td><td>gi|157818101</td><td>85</td><td>9</td><td>0</td><td>2</td><td>2</td><td>7</td></tr>
<tr><td>1406</td><td>small glutamine rich protein with tetratricopeptide repeats 2 [Rattus norvegicus], gi|81912832|sp|Q80W98.1|SGTB_RAT RecName: Full=Small glutamine-rich tetratricopeptide repeat-containing protein beta; AltName: Full=Beta-SGT; AltName: Full=Small glutamine-</td><td>gi|31745160</td><td>33</td><td>9</td><td>4</td><td>0</td><td>1</td><td>8</td></tr>
<tr><td>1407</td><td>synaptic vesicle glycoprotein 2a [Rattus norvegicus], gi|108935908|sp|Q02563.2|SV2A_RAT RecName: Full=Synaptic vesicle glycoprotein 2A; Short=Synaptic vesicle protein 2A; Short=Synaptic vesicle protein 2, gi|62027591|gb|AAH92132.1| Synaptic vesicle glycop</td><td>gi|148747227 (+1)</td><td>83</td><td>14</td><td>0</td><td>1</td><td>1</td><td>13</td></tr>
<tr><td>1408</td><td>CTD (carboxy-terminal domain, RNA polymerase II, polypeptide A) phosphatase, subunit 1 [Rattus norvegicus], gi|149015915|gb|EDL75222.1| CTD (carboxy-terminal domain, RNA polymerase II, polypeptide A) phosphatase, subunit 1 (predicted), isoform CRA_a [Ratt</td><td>gi|157823025</td><td>106</td><td>10</td><td>4</td><td>3</td><td>4</td><td>6</td></tr>
<tr><td>1409</td><td>similar to hypothetical protein MGC34646 (predicted), isoform CRA_a [Rattus norvegicus]</td><td>gi|149032866</td><td>38</td><td>12</td><td>4</td><td>0</td><td>1</td><td>11</td></tr>
<tr><td>1410</td><td>inositol polyphosphate-1-phosphatase [Rattus norvegicus], gi|55778623|gb|AAH86600.1| Inositol polyphosphate-1-phosphatase [Rattus norvegicus], gi|149046211|gb|EDL99104.1| rCG22563 [Rattus norvegicus]</td><td>gi|58865832</td><td>43</td><td>10</td><td>7</td><td>0</td><td>2</td><td>8</td></tr>
<tr><td>1411</td><td>membrane bound C2 domain containing protein [Rattus norvegicus], gi|74355732|gb|AAI01858.1| Membrane bound C2 domain containing protein [Rattus norvegicus], gi|149029673|gb|EDL84844.1| membrane bound C2 domain containing protein [Rattus norvegicus]</td><td>gi|75832132 (+1)</td><td>121</td><td>12</td><td>0</td><td>3</td><td>3</td><td>9</td></tr>
<tr><td>1412</td><td>PREDICTED: similar to HECT domain containing 3 [Rattus norvegicus]</td><td>gi|109477004</td><td>97</td><td>10</td><td>0</td><td>6</td><td>6</td><td>4</td></tr>
<tr><td>1413</td><td>tuberous sclerosis 2, isoform CRA_a [Rattus norvegicus]</td><td>gi|149052029</td><td>189</td><td>12</td><td>0</td><td>3</td><td>3</td><td>9</td></tr>
<tr><td>1414</td><td>rCG36692 [Rattus norvegicus]</td><td>gi|149019851 (+1)</td><td>80</td><td>9</td><td>6</td><td>1</td><td>3</td><td>6</td></tr>
<tr><td>1415</td><td>RAB33A, member of RAS oncogene family [Rattus norvegicus], gi|149060103|gb|EDM10919.1| RAB33A, member of RAS oncogene family (predicted) [Rattus norvegicus]</td><td>gi|157818281</td><td>27</td><td>11</td><td>1</td><td>0</td><td>0</td><td>11</td></tr>
<tr><td>1416</td><td>RecName: Full=Membrane-associated progesterone receptor component 1; AltName: Full=Acidic 25 kDa protein; AltName: Full=25-DX; AltName: Full=Ventral midline antigen; Short=VEMA, gi|6572674|gb|AAF17359.1|AF163321_1 ventral midline antigen VEMA [Rattus norv</td><td>gi|6647578</td><td>22</td><td>16</td><td>0</td><td>0</td><td>0</td><td>16</td></tr>
<tr><td>1417</td><td>RAB4B, member RAS oncogene family [Rattus norvegicus], gi|21313012|ref|NP_083667.1| RAB4B, member RAS oncogene family [Mus musculus], gi|1710026|sp|P51146.1|RAB4B_RAT RecName: Full=Ras-related protein Rab-4B, gi|46577105|sp|Q91ZR1.2|RAB4B_MOUSE RecName: F</td><td>gi|8394136</td><td>24</td><td>11</td><td>0</td><td>0</td><td>0</td><td>11</td></tr>
<tr><td>1418</td><td>RecName: Full=Nestin, gi|23452508|gb|AAN33053.1| nestin [Rattus norvegicus]</td><td>gi|146345465 (+3)</td><td>209</td><td>5</td><td>4</td><td>4</td><td>5</td><td>0</td></tr>
<tr><td>1419</td><td>melanoma antigen, family E, 1 [Rattus norvegicus], gi|118763773|gb|AAI28755.1| Melanoma antigen, family E, 1 [Rattus norvegicus], gi|149055568|gb|EDM07152.1| melanoma antigen, family E, 1 (predicted) [Rattus norvegicus]</td><td>gi|120586971</td><td>102</td><td>6</td><td>2</td><td>4</td><td>5</td><td>1</td></tr>
<tr><td>1420</td><td>rCG38480, isoform CRA_a [Rattus norvegicus]</td><td>gi|149034053</td><td>46</td><td>11</td><td>3</td><td>1</td><td>2</td><td>9</td></tr>
<tr><td>1421</td><td>tubulin polyglutamylase complex subunit 1 [Rattus norvegicus], gi|109481628|ref|XP_001077329.1| PREDICTED: similar to gene trap ROSA b-geo 22 [Rattus norvegicus], gi|149034675|gb|EDL89412.1| rCG29268 [Rattus norvegicus]</td><td>gi|157819903</td><td>33</td><td>10</td><td>0</td><td>6</td><td>6</td><td>4</td></tr>
<tr><td>1422</td><td>catalase [Rattus norvegicus], gi|115707|sp|P04762.3|CATA_RAT RecName: Full=Catalase, gi|203335|gb|AAB42378.1| catalase [Rattus norvegicus], gi|203345|gb|AAA40884.1| catalase (EC 1.11.1.6), gi|51980301|gb|AAH81853.1| Catalase [Rattus norvegicus], gi|149022</td><td>gi|6978607</td><td>60</td><td>13</td><td>1</td><td>0</td><td>0</td><td>13</td></tr>
<tr><td>1423</td><td>arginine-rich, mutated in early stage tumors [Mus musculus], gi|158706420|sp|P0C5H9.1|ARMET_RAT RecName: Full=Protein ARMET; AltName: Full=Mesencephalic astrocyte-derived neurotrophic factor; Short=MANF; AltName: Full=Arginine-rich protein; Flags: Precurs</td><td>gi|110625813</td><td>20</td><td>11</td><td>1</td><td>0</td><td>0</td><td>11</td></tr>
<tr><td>1424</td><td>PREDICTED: similar to Vpr-binding protein [Rattus norvegicus], gi|109485261|ref|XP_001071998.1| PREDICTED: similar to Vpr-binding protein [Rattus norvegicus]</td><td>gi|109483860 (+1)</td><td>172</td><td>5</td><td>0</td><td>3</td><td>3</td><td>2</td></tr>
<tr><td>1425</td><td>ribosomal protein S9 [Homo sapiens], gi|33504483|ref|NP_084043.1| ribosomal protein S9-like [Mus musculus], gi|110347598|ref|NP_112370.2| ribosomal protein S9 [Rattus norvegicus], gi|155372029|ref|NP_001094622.1| ribosomal protein S9 [Bos taurus], gi|7394</td><td>gi|14141193 (+1)</td><td>23</td><td>8</td><td>0</td><td>5</td><td>5</td><td>3</td></tr>
<tr><td>1426</td><td>sorting nexin 1 [Rattus norvegicus], gi|17367373|sp|Q99N27.1|SNX1_RAT RecName: Full=Sorting nexin-1, gi|12581483|gb|AAG59616.1|AF218916_1 sorting nexin 1 [Rattus norvegicus], gi|62089576|gb|AAH92201.1| Sorting nexin 1 [Rattus norvegicus], gi|149042007|gb|</td><td>gi|16758148</td><td>59</td><td>14</td><td>0</td><td>0</td><td>0</td><td>14</td></tr>
<tr><td>1427</td><td>peroxiredoxin 5 precursor [Rattus norvegicus], gi|20138819|sp|Q9R063.1|PRDX5_RAT RecName: Full=Peroxiredoxin-5, mitochondrial; AltName: Full=Prx-V; AltName: Full=Peroxisomal antioxidant enzyme; AltName: Full=PLP; AltName: Full=Thioredoxin reductase; AltNa</td><td>gi|16758404 (+1)</td><td>22</td><td>10</td><td>4</td><td>0</td><td>1</td><td>9</td></tr>
<tr><td>1428</td><td>ribosomal protein L6 [Rattus norvegicus]</td><td>gi|1490384 (+3)</td><td>34</td><td>9</td><td>0</td><td>8</td><td>8</td><td>1</td></tr>
<tr><td>1429</td><td>N-myristoyltransferase 2, isoform CRA_a [Rattus norvegicus]</td><td>gi|149021095 (+1)</td><td>60</td><td>8</td><td>7</td><td>0</td><td>2</td><td>6</td></tr>
<tr><td>1430</td><td>protein kinase C, beta 1, isoform CRA_a [Rattus norvegicus], gi|149068004|gb|EDM17556.1| protein kinase C, beta 1, isoform CRA_a [Rattus norvegicus]</td><td>gi|149068002 (+2)</td><td>62</td><td>7</td><td>0</td><td>5</td><td>5</td><td>2</td></tr>
<tr><td>1431</td><td>rCG44996, isoform CRA_c [Rattus norvegicus]</td><td>gi|149028178</td><td>59</td><td>5</td><td>11</td><td>0</td><td>3</td><td>2</td></tr>
<tr><td>1432</td><td>isocitrate dehydrogenase 3, beta subunit [Rattus norvegicus], gi|68051964|sp|Q68FX0.1|IDH3B_RAT RecName: Full=Isocitrate dehydrogenase [NAD] subunit beta, mitochondrial; AltName: Full=Isocitric dehydrogenase; AltName: Full=NAD(+)-specific ICDH; Flags: Pre</td><td>gi|55926203</td><td>42</td><td>15</td><td>0</td><td>0</td><td>0</td><td>15</td></tr>
<tr><td>1433</td><td>profilin 1, isoform CRA_c [Rattus norvegicus]</td><td>gi|149053224 (+2)</td><td>22</td><td>4</td><td>8</td><td>0</td><td>3</td><td>1</td></tr>
<tr><td>1434</td><td>phospholipase C beta-3 [Rattus norvegicus]</td><td>gi|13177635 (+2)</td><td>138</td><td>17</td><td>0</td><td>0</td><td>0</td><td>17</td></tr>
<tr><td>1435</td><td>small nuclear ribonucleoprotein D1 polypeptide 16kDa [Homo sapiens], gi|6678055|ref|NP_033252.1| small nuclear ribonucleoprotein D1 [Mus musculus], gi|78050059|ref|NP_001030237.1| small nuclear ribonucleoprotein D1 polypeptide 16kDa [Bos taurus], gi|15781</td><td>gi|5902102</td><td>13</td><td>16</td><td>0</td><td>0</td><td>0</td><td>16</td></tr>
<tr><td>1436</td><td>RAB2, member RAS oncogene family [Rattus norvegicus]</td><td>gi|149061036</td><td>18</td><td>17</td><td>0</td><td>0</td><td>0</td><td>17</td></tr>
<tr><td>1437</td><td>keratin 85 [Rattus norvegicus], gi|46485104|tpg|DAA02221.1| TPA_exp: type II keratin Kb25 [Rattus norvegicus]</td><td>gi|57012382</td><td>53</td><td>12</td><td>0</td><td>0</td><td>0</td><td>12</td></tr>
<tr><td>1438</td><td>Chain A, Catechol O-Methyltransferase Bisubstrate-Inhibitor Complex, gi|34811344|pdb|1H1D|A Chain A, Catechol O-Methyltransferase, gi|110591018|pdb|2CL5|A Chain A, Catechol-O-Methyltransferase In Complex With An Inhibitor, gi|110591019|pdb|2CL5|B Chain B,</td><td>gi|23200180 (+1)</td><td>25</td><td>6</td><td>3</td><td>1</td><td>2</td><td>4</td></tr>
<tr><td>1439</td><td>eukaryotic translation initiation factor 4A, isoform 3 [Bos taurus], gi|154147644|ref|NP_001093663.1| eukaryotic translation initiation factor 4A, isoform 3 [Sus scrofa], gi|157821621|ref|NP_001093628.1| eukaryotic translation initiation factor 4A isoform</td><td>gi|114051321</td><td>47</td><td>10</td><td>2</td><td>3</td><td>4</td><td>6</td></tr>
<tr><td>1440</td><td>cytosolic iron-sulfur protein assembly 1 homolog [Rattus norvegicus], gi|81909842|sp|Q5M7T1.1|CIAO1_RAT RecName: Full=Protein CIAO1; AltName: Full=WD repeat-containing protein 39, gi|56788798|gb|AAH88474.1| Cytosolic iron-sulfur protein assembly 1 homolog</td><td>gi|56912225</td><td>38</td><td>9</td><td>0</td><td>3</td><td>3</td><td>6</td></tr>
<tr><td>1441</td><td>chromobox homolog 5 [Mus musculus], gi|116008461|ref|NP_001070257.1| chromobox homolog 5 [Mus musculus], gi|157818381|ref|NP_001100267.1| chromobox homolog 5 (HP1 alpha homolog, Drosophila) [Rattus norvegicus], gi|158966694|ref|NP_001103686.1| chromobox h</td><td>gi|6671694</td><td>22</td><td>3</td><td>9</td><td>2</td><td>5</td><td>0</td></tr>
<tr><td>1442</td><td>c-src tyrosine kinase [Rattus norvegicus], gi|417209|sp|P32577.1|CSK_RAT RecName: Full=Tyrosine-protein kinase CSK; AltName: Full=C-SRC kinase, gi|20150729|pdb|1K9A|A Chain A, Crystal Structure Analysis Of Full-Length Carboxyl-Terminal Src Kinase At 2.5 A</td><td>gi|71795633</td><td>51</td><td>10</td><td>4</td><td>2</td><td>3</td><td>7</td></tr>
<tr><td>1443</td><td>amyloid beta (A4) precursor protein-binding, family A, member 1 [Rattus norvegicus], gi|6225060|sp|O35430.1|APBA1_RAT RecName: Full=Amyloid beta A4 precursor protein-binding family A member 1; AltName: Full=Neuron-specific X11 protein; AltName: Full=Neuro</td><td>gi|13929100 (+1)</td><td>93</td><td>10</td><td>2</td><td>1</td><td>2</td><td>8</td></tr>
<tr><td>1444</td><td>PNMA-like 2 [Rattus norvegicus], gi|149056860|gb|EDM08291.1| similar to KIAA1183 protein (predicted) [Rattus norvegicus]</td><td>gi|157819095</td><td>72</td><td>4</td><td>4</td><td>2</td><td>3</td><td>1</td></tr>
<tr><td>1445</td><td>phosphatidylinositol-5-phosphate 4-kinase, type II, beta [Rattus norvegicus], gi|47605934|sp|O88377.1|PI42B_RAT RecName: Full=Phosphatidylinositol-5-phosphate 4-kinase type-2 beta; AltName: Full=Phosphatidylinositol-5-phosphate 4-kinase type II beta; AltN</td><td>gi|16758316</td><td>47</td><td>13</td><td>0</td><td>3</td><td>3</td><td>10</td></tr>
<tr><td>1446</td><td>transforming, acidic coiled-coil containing protein 2, isoform CRA_b [Rattus norvegicus]</td><td>gi|149067590 (+3)</td><td>94</td><td>6</td><td>8</td><td>1</td><td>4</td><td>2</td></tr>
<tr><td>1447</td><td>OTU domain containing 6B [Rattus norvegicus], gi|149045466|gb|EDL98466.1| OTU domain containing 6B (predicted), isoform CRA_b [Rattus norvegicus]</td><td>gi|157820311</td><td>37</td><td>10</td><td>1</td><td>4</td><td>4</td><td>6</td></tr>
<tr><td>1448</td><td>ATP binding domain 1 family, member C, isoform CRA_b [Rattus norvegicus]</td><td>gi|149063366 (+1)</td><td>33</td><td>8</td><td>2</td><td>5</td><td>6</td><td>2</td></tr>
<tr><td>1449</td><td>ubiquitin-conjugating enzyme E2M [Homo sapiens], gi|21704162|ref|NP_663553.1| ubiquitin-conjugating enzyme E2M [Mus musculus], gi|134085789|ref|NP_001076972.1| ubiquitin-conjugating enzyme E2M [Bos taurus], gi|157817518|ref|NP_001101941.1| ubiquitin-conju</td><td>gi|4507791</td><td>21</td><td>5</td><td>6</td><td>0</td><td>2</td><td>3</td></tr>
<tr><td>1450</td><td>SGT1, suppressor of G2 allele of SKP1 [Rattus norvegicus], gi|187629783|sp|B0BN85.1|SUGT1_RAT RecName: Full=Suppressor of G2 allele of SKP1 homolog, gi|149050043|gb|EDM02367.1| SGT1, suppressor of G2 allele of SKP1 (S. cerevisiae), isoform CRA_b [Rattus n</td><td>gi|198278555</td><td>38</td><td>7</td><td>7</td><td>0</td><td>2</td><td>5</td></tr>
<tr><td>1451</td><td>PREDICTED: similar to BCSC-1 [Rattus norvegicus]</td><td>gi|109483334 (+2)</td><td>103</td><td>13</td><td>1</td><td>1</td><td>1</td><td>12</td></tr>
<tr><td>1452</td><td>karyopherin alpha 6 [Rattus norvegicus]</td><td>gi|59800339 (+1)</td><td>60</td><td>12</td><td>0</td><td>1</td><td>1</td><td>11</td></tr>
<tr><td>1453</td><td>nudix-type motif 5 [Rattus norvegicus], gi|81891344|sp|Q6AY63.1|NUDT5_RAT RecName: Full=ADP-sugar pyrophosphatase; AltName: Full=Nucleoside diphosphate-linked moiety X motif 5; Short=Nudix motif 5, gi|50927368|gb|AAH79176.1| Nudix (nucleoside diphosphate</td><td>gi|56090301</td><td>24</td><td>5</td><td>10</td><td>0</td><td>3</td><td>2</td></tr>
<tr><td>1454</td><td>rCG47395, isoform CRA_a [Rattus norvegicus]</td><td>gi|149062543</td><td>125</td><td>7</td><td>6</td><td>0</td><td>2</td><td>5</td></tr>
<tr><td>1455</td><td>COMM domain containing 3 [Rattus norvegicus], gi|51315701|sp|Q6P9U3.1|COMD3_RAT RecName: Full=COMM domain-containing protein 3, gi|38051994|gb|AAH60591.1| COMM domain containing 3 [Rattus norvegicus]</td><td>gi|38454210</td><td>22</td><td>13</td><td>2</td><td>0</td><td>1</td><td>12</td></tr>
<tr><td>1456</td><td>hypothetical protein LOC298384 [Rattus norvegicus], gi|109476873|ref|XP_001059650.1| PREDICTED: similar to CG4646-PA isoform 2 [Rattus norvegicus], gi|119368230|sp|Q498R7.1|CA123_RAT RecName: Full=UPF0587 protein C1orf123 homolog, gi|72679311|gb|AAI00101.</td><td>gi|77627996</td><td>18</td><td>2</td><td>10</td><td>0</td><td>3</td><td>0</td></tr>
<tr><td>1457</td><td>voltage-dependent anion channel 2 [Rattus norvegicus], gi|46397780|sp|P81155.2|VDAC2_RAT RecName: Full=Voltage-dependent anion-selective channel protein</td><td>gi|13786202 (+1)</td><td>32</td><td>10</td><td>0</td><td>1</td><td>1</td><td>9</td></tr>
</table>

| | | | | | | | | |
|---|---|---|---|---|---|---|---|---|
| | 2; Short=VDAC-2; AltName: Full=Outer mitochondrial membrane protein porin 2; AltName: Full=B36-VDAC, g | | | | | | | |
| 1458 | PREDICTED: similar to tubulin, gamma 2 [Rattus norvegicus] | gi\|109489030 (+3) | 53 | 11 | 0 | 2 | 2 | 9 |
| 1459 | ADP-ribosylation factor-like 10C [Homo sapiens], gi\|13385518\|ref\|NP_080287.1\| ADP-ribosylation factor-like 10C [Mus musculus], gi\|66730258\|ref\|NP_001019503.1\| ADP-ribosylation factor-like 8B [Rattus norvegicus], gi\|73985044\|ref\|XP_853013.1\| PREDICTED: sim | gi\|8922601 | 22 | 8 | 1 | 0 | 0 | 8 |
| 1460 | calcium-binding protein P23K beta [Rattus norvegicus] | gi\|2160456 (+1) | 17 | 11 | 3 | 0 | 1 | 10 |
| 1461 | rabaptin [Rattus norvegicus] | gi\|1575770 (+1) | 99 | 8 | 6 | 2 | 4 | 4 |
| 1462 | RAB, member of RAS oncogene family-like 2B [Rattus norvegicus], gi\|53734206\|gb\|AAH83624.1\| RAB, member of RAS oncogene family-like 2B [Rattus norvegicus] | gi\|61557322 | 25 | 7 | 5 | 2 | 4 | 3 |
| 1463 | similar to autoantigen [Rattus norvegicus] | gi\|149038054 (+2) | 153 | 3 | 0 | 0 | 0 | 3 |
| 1464 | COMM domain containing 7, isoform CRA_a [Rattus norvegicus] | gi\|149030970 (+1) | 19 | 16 | 0 | 0 | 0 | 16 |
| 1465 | selenophosphate synthetase 1, isoform CRA_b [Rattus norvegicus] | gi\|149021069 (+1) | 39 | 10 | 3 | 0 | 1 | 9 |
| 1466 | leucine zipper transcription factor-like 1 [Rattus norvegicus], gi\|81888016\|sp\|Q562C6.1\|LZTL1_RAT RecName: Full=Leucine zipper transcription factor-like protein 1, gi\|62530993\|gb\|AAH92585.1\| Leucine zipper transcription factor-like 1 [Rattus norvegicus], | gi\|66730445 | 35 | 14 | 1 | 0 | 0 | 14 |
| 1467 | hypothetical protein LOC216551 [Mus musculus], gi\|197927125\|ref\|NP_001128202.1\| galectin-related protein [Rattus norvegicus], gi\|119903733\|ref\|XP_594786.3\| PREDICTED: similar to RIKEN cDNA 1110067D22 [Bos taurus], gi\|81902047\|sp\|Q8VED9.1\|LEGLA_MOUSE RecNa | gi\|29611654 | 19 | 1 | 10 | 0 | 3 | 0 |
| 1468 | PREDICTED: similar to SET binding factor 1 isoform a [Rattus norvegicus] | gi\|109481181 (+1) | 209 | 13 | 1 | 0 | 0 | 13 |
| 1469 | RNA binding motif protein 8A [Homo sapiens], gi\|162287296\|ref\|NP_001095877.1\| RNA binding motif protein 8a isoform a [Mus musculus], gi\|27693451\|ref\|XP_215637.1\| PREDICTED: similar to RNA-binding protein 8A (RNA-binding motif protein 8A) (Ribonucleoprotei | gi\|4826972 | 20 | 14 | 0 | 0 | 0 | 14 |
| 1470 | crystallin, zeta [Rattus norvegicus], gi\|71152023\|sp\|Q6AYT0.1\|QOR_RAT RecName: Full=Quinone oxidoreductase; AltName: Full=NADPH:quinone reductase; AltName: Full=Zeta-crystallin, gi\|50926853\|gb\|AAH78927.1\| Crystallin, zeta [Rattus norvegicus], gi\|149026296 | gi\|58865938 | 35 | 5 | 6 | 0 | 2 | 3 |
| 1471 | solute carrier family 25, member 5 [Rattus norvegicus], gi\|728810\|sp\|Q09073.3\|ADT2_RAT RecName: Full=ADP/ATP translocase 2; AltName: Full=Adenine nucleotide translocator 2; Short=ANT 2; AltName: Full=ADP,ATP carrier protein 2; AltName: Full=Solute carrier | gi\|32189350 | 33 | 13 | 0 | 0 | 0 | 13 |
| 1472 | calcium/calmodulin-dependent protein kinase II, delta, isoform CRA_c [Rattus norvegicus] | gi\|149025903 (+2) | 54 | 7 | 0 | 4 | 4 | 3 |
| 1473 | kelch-like 9 [Rattus norvegicus], gi\|149044480\|gb\|EDL97739.1\| kelch-like 9 (Drosophila) (predicted) [Rattus norvegicus], gi\|197246771\|gb\|AAI68717.1\| Kelch-like 9 (Drosophila) [Rattus norvegicus] | gi\|157817981 | 69 | 7 | 0 | 3 | 3 | 4 |
| 1474 | diaphanous homolog 1 (Drosophila) (predicted), isoform CRA_b [Rattus norvegicus] | gi\|149017360 | 81 | 15 | 0 | 0 | 0 | 15 |
| 1475 | transmembrane protein 33 isoform 1 [Rattus norvegicus], gi\|10719974\|sp\|Q9Z142.1\|TMM33_RAT RecName: Full=Transmembrane protein 33; AltName: Full=Protein DB83, gi\|4579688\|dbj\|BAA75068.1\| db83 [Rattus norvegicus], gi\|149035325\|gb\|EDL90029.1\| transmembrane pr | gi\|11067391 (+1) | 28 | 13 | 2 | 0 | 1 | 12 |
| 1476 | hypothetical protein LOC291750 [Rattus norvegicus], gi\|149017047\|gb\|EDL76098.1\| similar to TRS85 homolog (predicted) [Rattus norvegicus] | gi\|157818025 | 160 | 12 | 0 | 0 | 0 | 12 |
| 1477 | insulin-like growth factor 2, binding protein 3 [Rattus norvegicus] | gi\|149033413 | 64 | 8 | 0 | 6 | 6 | 2 |
| 1478 | COMM domain containing 10 [Rattus norvegicus], gi\|51259449\|gb\|AAH79373.1\| COMM domain containing 10 [Rattus norvegicus], gi\|149064206\|gb\|EDM14409.1\| COMM domain containing 10, isoform CRA_a [Rattus norvegicus] | gi\|51948526 | 23 | 13 | 0 | 0 | 0 | 13 |
| 1479 | sorting nexin 2 (predicted), isoform CRA_c [Rattus norvegicus] | gi\|149064254 | 38 | 13 | 0 | 0 | 0 | 13 |
| 1480 | similar to RIKEN cDNA 1700012G19 gene (predicted) [Rattus norvegicus] | gi\|149052016 | 35 | 2 | 12 | 0 | 4 | 0 |
| 1481 | COMM domain containing 2 (predicted), isoform CRA_b [Rattus norvegicus] | gi\|149064733 (+1) | 20 | 12 | 0 | 0 | 0 | 12 |
| 1482 | rCG35703, isoform CRA_a [Rattus norvegicus] | gi\|149047664 | 41 | 9 | 1 | 1 | 1 | 8 |
| 1483 | vacuolar protein sorting 4 homolog B [Rattus norvegicus], gi\|68533827\|gb\|AAH99128.1\| Vacuolar protein sorting 4 homolog B (S. cerevisiae) [Rattus norvegicus], gi\|149037249\|gb\|EDL91749.1\| vacuolar protein sorting 4b (yeast) [Rattus norvegicus] | gi\|71043636 | 49 | 9 | 2 | 2 | 3 | 6 |
| 1484 | RAS p21 protein activator 1, isoform CRA_a [Rattus norvegicus] | gi\|149058970 (+3) | 117 | 8 | 3 | 1 | 2 | 6 |
| 1485 | CaM kinase-like vesicle-associated [Rattus norvegicus], gi\|81910540\|sp\|Q63092.1\|CAMKV_RAT RecName: Full=CaM kinase-like vesicle-associated protein; AltName: Full=1G5, gi\|349075\|gb\|AAA16633.1\| calmodulin-binding protein | gi\|13027458 | 54 | 9 | 0 | 2 | 2 | 7 |
| 1486 | upstream of NRAS, isoform CRA_a [Rattus norvegicus] | gi\|149030462 (+2) | 86 | 9 | 2 | 2 | 3 | 6 |
| 1487 | rCG61079, isoform CRA_b [Rattus norvegicus] | gi\|149043902 (+1) | 118 | 5 | 4 | 2 | 3 | 2 |
| 1488 | PREDICTED: similar to C06A6.3 [Rattus norvegicus] | gi\|109468066 (+2) | 46 | 11 | 1 | 2 | 2 | 9 |
| 1489 | rCG39172 [Rattus norvegicus] | gi\|149015719 | 70 | 6 | 5 | 1 | 3 | 3 |
| 1490 | rCG47487, isoform CRA_a [Rattus norvegicus] | gi\|149062420 (+1) | 99 | 11 | 0 | 2 | 2 | 9 |
| 1491 | brain and reproductive organ-expressed protein [Rattus norvegicus], gi\|81885356\|sp\|Q6P7Q1.1\|BRE_RAT RecName: Full=BRCA1-A complex subunit BRE; AltName: Full=Brain and reproductive organ-expressed protein; AltName: Full=BRCA1/BRCA2-containing complex subun | gi\|40538868 | 44 | 6 | 4 | 3 | 4 | 2 |
| 1492 | soluble guanylyl cyclase beta 1 subunit [Rattus norvegicus] | gi\|27374983 (+2) | 70 | 10 | 1 | 4 | 4 | 6 |
| 1493 | RecName: Full=Acetyl-CoA acetyltransferase, mitochondrial; AltName: Full=Acetoacetyl-CoA thiolase; Flags: Precursor, gi\|220655\|dbj\|BAA03016.1\| mitochondrial acetoacetyl-CoA thiolase [Rattus norvegicus], gi\|149041679\|gb\|EDL95520.1\| acetyl-coenzyme A acetyl | gi\|135757 (+1) | 45 | 14 | 0 | 1 | 1 | 13 |
| 1494 | calcium channel, voltage-dependent, alpha 2/delta 3 subunit, isoform CRA_a [Rattus norvegicus] | gi\|149034245 (+2) | 105 | 9 | 0 | 2 | 2 | 7 |
| 1495 | rCG38798, isoform CRA_a [Rattus norvegicus], gi\|187469067\|gb\|AAI66821.1\| Armc6 protein [Rattus norvegicus] | gi\|149035997 | 51 | 11 | 2 | 0 | 1 | 10 |
| 1496 | syntaxin 13 [Rattus norvegicus] | gi\|3184552 (+2) | 30 | 8 | 0 | 4 | 4 | 4 |
| 1497 | mitochondrial carrier homolog 2 [Rattus norvegicus], gi\|149022589\|gb\|EDL79483.1\| mitochondrial carrier homolog 2 (C. elegans) (predicted), isoform CRA_a [Rattus norvegicus], gi\|165971637\|gb\|AAI58688.1\| Mitochondrial carrier homolog 2 (C. elegans) [Rattus | gi\|158819029 | 34 | 9 | 0 | 5 | 5 | 4 |
| 1498 | mannose phosphate isomerase [Rattus norvegicus], gi\|62510703\|sp\|Q68FX1.1\|MPI_RAT RecName: Full=Mannose-6-phosphate isomerase; AltName: Full=Phosphomannose isomerase; Short=PMI; AltName: Full=Phosphohexomutase, gi\|51259370\|gb\|AAH79111.1\| Mannose phosphate | gi\|51854221 | 46 | 2 | 11 | 0 | 3 | 0 |
| 1499 | eukaryotic translation initiation factor 2, subunit 2 beta, 38kDa [Rattus norvegicus], gi\|38382861\|gb\|AAH62402.1\| Eukaryotic translation initiation factor 2, subunit 2 (beta) [Rattus norvegicus], gi\|149030917\|gb\|EDL85944.1\| eukaryotic translation initiati | gi\|40786451 | 38 | 10 | 2 | 0 | 1 | 9 |
| 1500 | r-sly1 | gi\|1144569 (+2) | 74 | 7 | 0 | 4 | 4 | 3 |
| 1501 | RecName: Full=UDP-glucose 4-epimerase; AltName: Full=UDP-galactose 4-epimerase; AltName: Full=Galactowaldenase, gi\|57792\|emb\|CAA37897.1\| unnamed protein product [Rattus sp.] | gi\|120922 (+1) | 38 | 6 | 2 | 3 | 4 | 2 |
| 1502 | hypothetical protein LOC500988 [Rattus norvegicus], gi\|149041488\|gb\|EDL95329.1\| rCG58047 [Rattus norvegicus] | gi\|157817692 | 54 | 10 | 1 | 0 | 0 | 10 |
| 1503 | glutamate cysteine ligase, modifier subunit [Rattus norvegicus], gi\|1346189\|sp\|P48508.1\|GSH0_RAT RecName: Full=Glutamate--cysteine ligase regulatory subunit; AltName: Full=Gamma-glutamylcysteine synthetase regulatory subunit; AltName: Full=Gamma-ECS regul | gi\|8393446 | 31 | 7 | 3 | 2 | 3 | 4 |
| 1504 | myotubularin related protein 9 [Rattus norvegicus], gi\|53733831\|gb\|AAH83644.1\| Myotubularin related protein 9 [Rattus norvegicus], gi\|149030268\|gb\|EDL85324.1\| myotubularin related protein 9 [Rattus norvegicus] | gi\|54114991 | 63 | 8 | 1 | 0 | 0 | 8 |
| 1505 | receptor accessory protein 5 (predicted), isoform CRA_b [Rattus norvegicus] | gi\|149017173 | 14 | 14 | 0 | 0 | 0 | 14 |
| 1506 | protein phosphatase 3, catalytic subunit, beta [Rattus norvegicus], gi\|129343\|sp\|P20651.1\|PP2BB_RAT RecName: Full=Serine/threonine-protein phosphatase 2B catalytic subunit beta isoform; AltName: Full=Calmodulin-dependent calcineurin A subunit beta isoform | gi\|8394033 | 59 | 9 | 3 | 0 | 1 | 8 |
| 1507 | guanylate kinase 1 [Rattus norvegicus], gi\|149052768\|gb\|EDM04585.1\| guanylate kinase 1, isoform CRA_a [Rattus norvegicus] | gi\|226874873 (+1) | 24 | 9 | 6 | 0 | 2 | 7 |
| 1508 | bisphosphoglycerate mutase [Rattus norvegicus], gi\|38328489\|gb\|AAH62240.1\| 2,3-bisphosphoglycerate mutase [Rattus norvegicus], gi\|149065234\|gb\|EDM15310.1\| 2,3-bisphosphoglycerate mutase [Rattus norvegicus] | gi\|40786455 | 30 | 11 | 1 | 0 | 0 | 11 |
| 1509 | myotubularin related protein 2 (predicted), isoform CRA_a [Rattus norvegicus], gi\|149020686\|gb\|EDL78491.1\| myotubularin related protein 2 (predicted), isoform CRA_a [Rattus norvegicus] | gi\|149020685 (+1) | 66 | 10 | 0 | 4 | 4 | 6 |
| 1510 | PREDICTED: similar to Ribonuclease P protein subunit p30 (RNaseP protein p30) (RNase P subunit 2) [Rattus norvegicus], gi\|109463823\|ref\|XP_001080083.1\| PREDICTED: similar to Ribonuclease P protein subunit p30 (RNaseP protein p30) (RNase P subunit 2) [Ratt | gi\|109460073 | 29 | 14 | 0 | 0 | 0 | 14 |
| 1511 | nudix-type motif 2 [Rattus norvegicus], gi\|67460101\|sp\|Q6PEC0.3\|AP4A_RAT RecName: Full=Bis(5'-nucleosyl)-tetraphosphatase [asymmetrical]; AltName: Full=Diadenosine 5',5'''-P1,P4-tetraphosphate asymmetrical hydrolase; Short=Diadenosine tetraphosphatase; Sh | gi\|46485421 | 17 | 9 | 6 | 0 | 2 | 7 |
| 1512 | rCG21247, isoform CRA_b [Rattus norvegicus] | gi\|149063116 (+4) | 108 | 9 | 0 | 1 | 1 | 8 |
| 1513 | ribosomal protein L11 [Mus musculus], gi\|15431290\|ref\|NP_000966.2\| ribosomal protein L11 [Homo sapiens], gi\|115497634\|ref\|NP_001069049.1\| ribosomal protein L11 [Bos taurus], gi\|149695161\|ref\|XP_001504267.1\| PREDICTED: similar to 60S ribosomal protein L11 | gi\|13385408 (+1) | 20 | 13 | 0 | 0 | 0 | 13 |
| 1514 | cell adhesion molecule 3 [Rattus norvegicus], gi\|114149321\|sp\|Q1WIM3.1\|CADM3_RAT RecName: Full=Cell adhesion molecule 3; AltName: Full=Immunoglobulin superfamily member 4B; AltName: Full=Nectin-like protein 1; Flags: Precursor, gi\|82619334\|gb\|ABB85362.1\| | gi\|114052921 (+1) | 43 | 11 | 0 | 0 | 0 | 11 |
| 1515 | DCN1, defective in cullin neddylation 1, domain containing 1 (S. cerevisiae) (predicted), isoform CRA_b [Rattus norvegicus] | gi\|149048716 (+1) | 22 | 13 | 2 | 0 | 1 | 12 |
| 1516 | RecName: Full=MAM domain-containing glycosylphosphatidylinositol anchor protein 1; AltName: Full=Ig6M; Flags: Precursor | gi\|150384462 (+1) | 106 | 14 | 0 | 0 | 0 | 14 |
| 1517 | guanosine monophosphate reductase [Rattus norvegicus], gi\|8928123\|sp\|Q9Z244.1\|GMPR1_RAT RecName: Full=GMP reductase 1; AltName: Full=Guanosine 5'-monophosphate oxidoreductase 1; Short=Guanosine monophosphate reductase 1, gi\|3907579\|gb\|AAC78657.1\| guanosin | gi\|17105340 | 37 | 13 | 0 | 0 | 0 | 13 |

| | | | | | | | | |
|---|---|---|---|---|---|---|---|---|
| 1518 | retinoblastoma binding protein 7 [Homo sapiens], gi\|13929162\|ref\|NP_114004.1\| retinoblastoma binding protein 7 [Rattus norvegicus], gi\|109130031\|ref\|XP_001103891.1\| PREDICTED: retinoblastoma binding protein 7 isoform 5 [Macaca mulatta], gi\|2494891\|sp\|Q165 | gi\|4506439 | 48 | 7 | 4 | 0 | 1 | 6 |
| 1519 | Parkinson disease 7 domain containing 1 (predicted), isoform CRA_b [Rattus norvegicus] | gi\|149061618 (+1) | 22 | 13 | 1 | 0 | 0 | 13 |
| 1520 | sterol-C4-methyl oxidase-like [Rattus norvegicus], gi\|28558102\|sp\|O35532.1\|ERG25_RAT RecName: Full=C-4 methylsterol oxidase; AltName: Full=Methylsterol monooxygenase; AltName: Full=Neuropep 1; AltName: Full=RANP-1, gi\|2605606\|dbj\|BAA23329.1\| RANP-1 [Rattu | gi\|18266684 | 35 | 13 | 0 | 0 | 0 | 13 |
| 1521 | 3-mercaptopyruvate sulfurtransferase [Rattus norvegicus], gi\|3122930\|sp\|P97532.3\|THTM_RAT RecName: Full=3-mercaptopyruvate sulfurtransferase; Short=MST, gi\|1741864\|dbj\|BAA09127.1\| mercaptopyruvate sulfurtransferase [Rattus norvegicus], gi\|55824737\|gb\|AAH8 | gi\|20304123 | 33 | 4 | 11 | 0 | 3 | 1 |
| 1522 | PREDICTED: similar to mago-nashi homolog [Rattus norvegicus], gi\|109474348\|ref\|XP_001069775.1\| PREDICTED: similar to mago-nashi homolog [Rattus norvegicus] | gi\|109472730 (+2) | 23 | 13 | 0 | 0 | 0 | 13 |
| 1523 | ubiquitin-conjugating enzyme E2R 2 [Mus musculus], gi\|22212943\|ref\|NP_060281.2\| ubiquitin-conjugating enzyme UBC3B [Homo sapiens], gi\|189011626\|ref\|NP_001121045.1\| hypothetical protein LOC689226 [Rattus norvegicus], gi\|73971749\|ref\|XP_854606.1\| PREDICTED: | gi\|13385778 | 27 | 7 | 4 | 1 | 2 | 5 |
| 1524 | PRP31 pre-mRNA processing factor 31 homolog [Rattus norvegicus], gi\|149029802\|gb\|EDL84934.1\| PRP31 pre-mRNA processing factor 31 homolog (yeast) (predicted) [Rattus norvegicus] | gi\|157819227 | 55 | 4 | 4 | 0 | 1 | 3 |
| 1525 | rCG40565, isoform CRA_a [Rattus norvegicus] | gi\|149067975 | 38 | 7 | 1 | 1 | 1 | 6 |
| 1526 | ligase I, DNA, ATP-dependent, isoform CRA_a [Rattus norvegicus], gi\|149028348\|gb\|EDL83754.1\| ligase I, DNA, ATP-dependent, isoform CRA_a [Rattus norvegicus] | gi\|149028347 (+1) | 104 | 8 | 3 | 1 | 2 | 6 |
| 1527 | ZW10, kinetochore associated, homolog [Rattus norvegicus], gi\|81908675\|sp\|Q4V8C2.3\|ZW10_RAT RecName: Full=Centromere/kinetochore protein zw10 homolog, gi\|66911467\|gb\|AAH97452.1\| ZW10, kinetochore associated, homolog (Drosophila) [Rattus norvegicus], gi\|14 | gi\|67078414 | 88 | 7 | 3 | 2 | 3 | 4 |
| 1528 | rCG39108, isoform CRA_a [Rattus norvegicus] | gi\|149032456 (+1) | 94 | 11 | 2 | 1 | 2 | 9 |
| 1529 | PREDICTED: similar to polymerase (RNA) III (DNA directed) polypeptide A, 155kDa [Rattus norvegicus] | gi\|109502980 (+1) | 153 | 11 | 0 | 3 | 3 | 8 |
| 1530 | candidate tumor suppressor in ovarian cancer 2 [Rattus norvegicus], gi\|149053384\|gb\|EDM05201.1\| rCG34508, isoform CRA_a [Rattus norvegicus] | gi\|157817508 | 24 | 7 | 1 | 4 | 4 | 3 |
| 1531 | similar to D7Wsu128e protein [Rattus norvegicus] | gi\|149068020 (+2) | 40 | 10 | 3 | 1 | 2 | 8 |
| 1532 | similar to expressed sequence AI317237 (predicted) [Rattus norvegicus] | gi\|149032048 (+1) | 168 | 10 | 0 | 2 | 2 | 8 |
| 1533 | similar to 4933407C03Rik protein (predicted), isoform CRA_b [Rattus norvegicus], gi\|149038292\|gb\|EDL92652.1\| similar to 4933407C03Rik protein (predicted), isoform CRA_b [Rattus norvegicus] | gi\|149038291 (+1) | 75 | 10 | 0 | 4 | 4 | 6 |
| 1534 | plexin A3 [Rattus norvegicus], gi\|149029861\|gb\|EDL84973.1\| plexin A3 (mapped), isoform CRA_b [Rattus norvegicus] | gi\|164518900 | 208 | 10 | 2 | 1 | 2 | 8 |
| 1535 | rCG58801 [Rattus norvegicus] | gi\|149059731 (+2) | 81 | 9 | 0 | 1 | 1 | 8 |
| 1536 | PREDICTED: similar to retinoblastoma binding protein 4 [Rattus norvegicus], gi\|109466758\|ref\|XP_001062166.1\| PREDICTED: similar to retinoblastoma binding protein 4 [Rattus norvegicus] | gi\|109464838 (+3) | 47 | 8 | 3 | 0 | 1 | 7 |
| 1537 | rCG57303, isoform CRA_c [Rattus norvegicus] | gi\|149039651 (+2) | 75 | 3 | 5 | 2 | 4 | 0 |
| 1538 | septin 11 [Rattus norvegicus], gi\|148673287\|gb\|EDL05234.1\| septin 11, isoform CRA_b [Mus musculus], gi\|149033859\|gb\|EDL88655.1\| septin 6 (predicted), isoform CRA_a [Rattus norvegicus], gi\|189484058\|gb\|ACE00321.1\| septin 11 isoform I [Rattus norvegicus], g | gi\|157821523 (+1) | 50 | 12 | 0 | 0 | 0 | 12 |
| 1539 | tRNA(m1A58)-methyltransferase subunit TRM61 [Rattus norvegicus], gi\|81910870\|sp\|Q6AY46.1\|TRM61_RAT RecName: Full=tRNA (adenine-N(1)-)-methyltransferase catalytic subunit TRMT61A; AltName: Full=tRNA(m1A58)-methyltransferase subunit TRMT61A; Short=tRNA(m1A5 | gi\|56090395 | 32 | 8 | 0 | 2 | 2 | 6 |
| 1540 | minichromosome maintenance deficient 2 mitotin (S. cerevisiae) (predicted) [Rattus norvegicus] | gi\|149036703 | 55 | 8 | 2 | 2 | 3 | 5 |
| 1541 | rCG46446, isoform CRA_b [Rattus norvegicus] | gi\|149058104 (+1) | 29 | 9 | 4 | 0 | 1 | 8 |
| 1542 | PREDICTED: similar to cDNA sequence BC021395 [Rattus norvegicus] | gi\|109506377 (+2) | 49 | 5 | 5 | 3 | 5 | 0 |
| 1543 | beta-adrenergic receptor kinase; beta-ARK [Rattus sp.], gi\|382661\|prf\|\|1819488A beta adrenergic receptor kinase | gi\|260172 (+1) | 80 | 11 | 0 | 3 | 3 | 8 |
| 1544 | PREDICTED: similar to Catenin delta-2 (Neural plakophilin-related ARM-repeat protein) (NPRAP) (Neurojungin) [Rattus norvegicus] | gi\|109464562 (+1) | 134 | 9 | 0 | 3 | 3 | 6 |
| 1545 | succinate dehydrogenase complex, subunit A, flavoprotein (Fp) [Rattus norvegicus], gi\|52782765\|sp\|Q920L2.1\|DHSA_RAT RecName: Full=Succinate dehydrogenase [ubiquinone] flavoprotein subunit, mitochondrial; AltName: Full=Flavoprotein subunit of complex II; S | gi\|18426858 | 72 | 12 | 0 | 2 | 2 | 10 |
| 1546 | osteoclast stimulating factor 1, isoform CRA_b [Rattus norvegicus] | gi\|149062558 (+1) | 19 | 5 | 6 | 3 | 5 | 0 |
| 1547 | hypothetical protein LOC363485 [Rattus norvegicus], gi\|149055570\|gb\|EDM07154.1\| similar to RIKEN cDNA 2610029G23 (predicted), isoform CRA_a [Rattus norvegicus] | gi\|157818635 | 22 | 3 | 8 | 0 | 3 | 0 |
| 1548 | similar to RIKEN cDNA 4931406C07, isoform CRA_b [Rattus norvegicus] | gi\|149020644 (+1) | 31 | 6 | 7 | 0 | 2 | 4 |
| 1549 | similar to KIAA0456 protein (predicted) [Rattus norvegicus] | gi\|149058671 | 58 | 4 | 1 | 2 | 2 | 2 |
| 1550 | protein kinase N1 [Rattus norvegicus], gi\|38197376\|gb\|AAH61836.1\| Protein kinase N1 [Rattus norvegicus], gi\|149037900\|gb\|EDL92260.1\| protein kinase N1 [Rattus norvegicus] | gi\|166063985 (+2) | 104 | 7 | 3 | 3 | 4 | 3 |
| 1551 | rCG33798, isoform CRA_a [Rattus norvegicus] | gi\|149052838 (+2) | 113 | 10 | 3 | 0 | 1 | 9 |
| 1552 | RecName: Full=Hsp70-binding protein 1; AltName: Full=Heat shock protein-binding protein 1; Short=HspBP1; AltName: Full=Hsp70-interacting protein 1, gi\|47939891\|gb\|AAH72541.1\| HSPA (heat shock 70kDa) binding protein, cytoplasmic cochaperone 1 [Rattus norve | gi\|78098997 | 39 | 3 | 7 | 0 | 2 | 1 |
| 1553 | ubiquitin-like domain containing CTD phosphatase 1 [Rattus norvegicus], gi\|81882910\|sp\|Q5FWT7.1\|UBCP1_RAT RecName: Full=Ubiquitin-like domain-containing CTD phosphatase 1, gi\|58400827\|gb\|AAH89210.1\| Ubiquitin-like domain containing CTD phosphatase 1 [Ratt | gi\|62078971 | 37 | 7 | 0 | 2 | 2 | 5 |
| 1554 | RAP1A, member of RAS oncogene family [Homo sapiens], gi\|21704066\|ref\|NP_663516.1\| RAS-related protein-1a [Mus musculus], gi\|27806115\|ref\|NP_776873.1\| RAP1A, member of RAS oncogene family [Bos taurus], gi\|54114993\|ref\|NP_001005765.1\| RAP1A, member of RAS o | gi\|4506413 | 21 | 12 | 0 | 0 | 0 | 12 |
| 1555 | NADH dehydrogenase (ubiquinone) 1 alpha subcomplex, 9 [Rattus norvegicus], gi\|149049372\|gb\|EDM01826.1\| rCG30092 [Rattus norvegicus], gi\|197246398\|gb\|AAI68777.1\| Ndufa9 protein [Rattus norvegicus] | gi\|198278533 (+1) | 43 | 5 | 0 | 3 | 3 | 2 |
| 1556 | dihydrolipoamide dehydrogenase [Rattus norvegicus], gi\|81885266\|sp\|Q6P6R2.1\|DLDH_RAT RecName: Full=Dihydrolipoyl dehydrogenase, mitochondrial; AltName: Full=Dihydrolipoamide dehydrogenase; Flags: Precursor, gi\|38303871\|gb\|AAH62069.1\| Dihydrolipoamide dehy | gi\|40786469 | 54 | 14 | 0 | 0 | 0 | 14 |
| 1557 | leucine rich repeat and Ig domain containing 1 [Rattus norvegicus], gi\|33305422\|gb\|AAQ02775.1\|AF373780_1 putative transmembrane protein mV/BamHI#3 [Mus musculus], gi\|149041742\|gb\|EDL95583.1\| leucine rich repeat neuronal 6A, isoform CRA_a [Rattus norvegicu | gi\|213688408 (+1) | 70 | 10 | 0 | 0 | 0 | 10 |
| 1558 | TBC1 domain family, member 24 [Rattus norvegicus], gi\|149051996\|gb\|EDM03813.1\| similar to CG9339-PA (predicted) [Rattus norvegicus] | gi\|157786588 | 63 | 5 | 6 | 0 | 2 | 3 |
| 1559 | Fas (TNFRSF6)-associated via death domain [Rattus norvegicus], gi\|22535903\|gb\|AAN01113.1\|AF406779_1 FADD/MORT1 protein with death effector domain [Rattus norvegicus], gi\|20145614\|emb\|CAD29628.1\| Fas death domain associated protein [Rattus norvegicus], gi\| | gi\|23097354 | 23 | 4 | 9 | 0 | 3 | 1 |
| 1560 | glutamate receptor, ionotropic, AMPA2, isoform CRA_b [Rattus norvegicus] | gi\|149048284 | 99 | 8 | 0 | 0 | 0 | 8 |
| 1561 | PREDICTED: similar to dendritic cell-derived ubiquitin-like protein [Rattus norvegicus], gi\|109490468\|ref\|XP_001063674.1\| PREDICTED: similar to dendritic cell-derived ubiquitin-like protein [Rattus norvegicus] | gi\|109487949 (+1) | 27 | 12 | 0 | 0 | 0 | 12 |
| 1562 | PREDICTED: similar to Serine/threonine-protein phosphatase 2A 56 kDa regulatory subunit gamma isoform (PP2A, B subunit, B gamma isoform) (PP2A, B subunit, B56 gamma isoform) (PP2A, B subunit, PR61 gamma isoform) (PP2A, B subunit, R5 gamma isoform) (PP2A, | gi\|109478743 | 74 | 10 | 0 | 0 | 0 | 10 |
| 1563 | mediator of cell motility 1 [Rattus norvegicus], gi\|81918134\|sp\|Q4QQR9.1\|MEMO1_RAT RecName: Full=Protein MEMO1; AltName: Full=Mediator of ErbB2-driven cell motility 1; Short=Protein memo, gi\|67678458\|gb\|AAH98061.1\| Mediator of cell motility 1 [Rattus norv | gi\|71361623 | 34 | 7 | 3 | 0 | 1 | 6 |
| 1564 | serine (or cysteine) peptidase inhibitor, clade F, member 2 [Rattus norvegicus], gi\|51259429\|gb\|AAH79362.1\| Serine (or cysteine) peptidase inhibitor, clade F, member 2 [Rattus norvegicus], gi\|149053393\|gb\|EDM05210.1\| rCG33981, isoform CRA_a [Rattus norveg | gi\|58865362 | 55 | 0 | 12 | 0 | 4 | 0 |
| 1565 | lysosomal ATPase [Rattus norvegicus] | gi\|17529569 (+1) | 12 | 13 | 0 | 0 | 0 | 13 |
| 1566 | ribosomal protein L4 [Rattus norvegicus], gi\|1710511\|sp\|P50878.3\|RL4_RAT RecName: Full=60S ribosomal protein L4; AltName: Full=L1, gi\|1165139\|emb\|CAA57671.1\| ribosomal protein L4 [Rattus norvegicus] | gi\|11968086 (+1) | 47 | 7 | 0 | 7 | 7 | 0 |
| 1567 | dynactin 1, isoform CRA_b [Rattus norvegicus] | gi\|149036515 | 141 | 5 | 0 | 5 | 5 | 0 |
| 1568 | immunoglobulin heavy chain [Rattus sp.] | gi\|243866 | 51 | 2 | 9 | 0 | 3 | 0 |
| 1569 | 7-dehydrocholesterol reductase [Rattus norvegicus], gi\|81882109\|sp\|Q9Z2Z8.1\|DHCR7_RAT RecName: Full=7-dehydrocholesterol reductase; Short=7-DHC reductase; AltName: Full=Sterol Delta(7)-reductase, gi\|4877562\|gb\|AAD31383.1\|AF071500_1 7-dehydrocholesterol re | gi\|11693158 | 54 | 13 | 0 | 0 | 0 | 13 |
| 1570 | PREDICTED: similar to Secretory carrier-associated membrane protein 3 (Secretory carrier membrane protein 3) [Rattus norvegicus] | gi\|109465077 (+1) | 46 | 11 | 0 | 0 | 0 | 11 |
| 1571 | hematological and neurological expressed 1 [Rattus norvegicus], gi\|81884473\|sp\|Q6AXU6.3\|HN1_RAT RecName: Full=Hematological and neurological expressed 1 protein, gi\|50927765\|gb\|AAH79311.1\| Hematological and neurological expressed 1 [Rattus norvegicus], gi | gi\|54312084 | 16 | 10 | 0 | 0 | 0 | 10 |
| 1572 | glucose-6-phosphatase catalytic subunit-related protein [Rattus norvegicus] | gi\|28395063 (+1) | 39 | 12 | 0 | 0 | 0 | 12 |
| 1573 | synaptosomal-associated protein 29 [Rattus norvegicus], gi\|7769720\|gb\|AAF69517.1\|AF260577_1 SNAP-29 protein [Rattus norvegicus], gi\|60649723\|gb\|AAH91693.1\| Synaptosomal-associated protein 29 [Rattus norvegicus], gi\|149019741\|gb\|EDL77889.1\| synaptosomal-as | gi\|62751974 (+1) | 29 | 11 | 0 | 1 | 1 | 10 |
| 1574 | enhancer factor-1-alpha | gi\|203999 (+3) | 36 | 7 | 2 | 1 | 2 | 5 |
| 1575 | dedicator of cytokinesis 7 [Rattus norvegicus] | gi\|71362801 | 55 | 9 | 0 | 3 | 3 | 6 |
| 1576 | PREDICTED: similar to component of oligomeric golgi complex 5 isoform 1 [Rattus norvegicus] | gi\|109479252 | 96 | 5 | 4 | 1 | 2 | 3 |
| 1577 | tRNA-intron endonuclease 34 [Rattus norvegicus], gi\|53733960\|gb\|AAH83767.1\| TRNA splicing endonuclease 34 homolog (S. cerevisiae) [Rattus norvegicus], | gi\|55741514 | 34 | 6 | 4 | 2 | 3 | 3 |

| | | | | | | | | |
|---|---|---|---|---|---|---|---|---|
| | gi\|149029795\|gb\|EDL84927.1\| tRNA splicing endonuclease 34 homolog, isoform CRA_b [Rattus norvegicus] | | | | | | | |
| 1578 | cytochrome P450, family 51, subfamily A, polypeptide 1 [Rattus norvegicus], gi\|2493384\|sp\|Q64654.1\|CP51A_RAT RecName: Full=Cytochrome P450 51A1; AltName: Full=CYPLI; AltName: Full=P450LI; AltName: Full=Sterol 14-alpha demethylase; AltName: Full=Lanosterol | gi\|6978751 (+1) | 57 | 4 | 0 | 3 | 3 | 1 |
| 1579 | peptidase D [Rattus norvegicus], gi\|81889014\|sp\|Q5I0D7.1\|PEPD_RAT RecName: Full=Xaa-Pro dipeptidase; Short=X-Pro dipeptidase; AltName: Full=Proline dipeptidase; Short=Prolidase; AltName: Full=Imidodipeptidase; AltName: Full=Peptidase D, gi\|56970766\|gb\|AAH | gi\|57528238 | 55 | 10 | 2 | 0 | 1 | 9 |
| 1580 | uroporphyrinogen decarboxylase [Rattus norvegicus], gi\|149035558\|gb\|EDL90239.1\| uroporphyrinogen decarboxylase, isoform CRA_a [Rattus norvegicus], gi\|165970690\|gb\|AAI58691.1\| Uroporphyrinogen decarboxylase [Rattus norvegicus] | gi\|158749613 | 41 | 7 | 1 | 0 | 0 | 7 |
| 1581 | COMM domain containing 5, isoform CRA_a [Rattus norvegicus], gi\|149066059\|gb\|EDM15932.1\| COMM domain containing 5, isoform CRA_a [Rattus norvegicus], gi\|149066060\|gb\|EDM15933.1\| COMM domain containing 5, isoform CRA_a [Rattus norvegicus] | gi\|149066058 (+1) | 25 | 12 | 1 | 0 | 0 | 12 |
| 1582 | hypothetical protein LOC606294 [Rattus norvegicus], gi\|149038680\|gb\|EDL92969.1\| rCG21965, isoform CRA_c [Rattus norvegicus] | gi\|72384369 | 71 | 11 | 0 | 1 | 1 | 10 |
| 1583 | rCG32194 [Rattus norvegicus] | gi\|149043066 | 114 | 10 | 0 | 1 | 1 | 9 |
| 1584 | golgi reassembly stacking protein 2 [Rattus norvegicus], gi\|51259254\|gb\|AAH78731.1\| Golgi reassembly stacking protein 2 [Rattus norvegicus], gi\|149022191\|gb\|EDL79085.1\| golgi reassembly stacking protein 2 [Rattus norvegicus] | gi\|56090463 | 47 | 5 | 8 | 0 | 3 | 2 |
| 1585 | RNA polymerase II associated protein 3 [Rattus norvegicus], gi\|81910765\|sp\|Q68FQ7.1\|RPAP3_RAT RecName: Full=RNA polymerase II-associated protein 3, gi\|51260045\|gb\|AAH79414.1\| RNA polymerase II associated protein 3 [Rattus norvegicus] | gi\|51948458 | 75 | 9 | 0 | 1 | 1 | 8 |
| 1586 | ubiquitin-like 7 (bone marrow stromal cell-derived) [Rattus norvegicus], gi\|50927376\|gb\|AAH79221.1\| Ubiquitin-like 7 (bone marrow stromal cell-derived) [Rattus norvegicus], gi\|149041816\|gb\|EDL95657.1\| bone marrow stromal cell-derived ubiquitin-like protei | gi\|51948464 | 41 | 9 | 0 | 2 | 2 | 7 |
| 1587 | similar to RIKEN cDNA 2400003C14, isoform CRA_b [Rattus norvegicus] | gi\|149038149 (+1) | 32 | 8 | 2 | 0 | 1 | 7 |
| 1588 | LRRGT00111 [Rattus norvegicus] | gi\|37361908 (+1) | 45 | 9 | 0 | 2 | 2 | 7 |
| 1589 | PREDICTED: similar to Phosphatidylinositol-4,5-bisphosphate 3-kinase catalytic subunit alpha isoform (PI3-kinase p110 subunit alpha) (PtdIns-3-kinase p110) (PI3K) isoform 3 [Rattus norvegicus] | gi\|109466432 (+1) | 124 | 9 | 0 | 0 | 0 | 9 |
| 1590 | dihydrouridine synthase 3-like (S. cerevisiae), isoform CRA_c [Rattus norvegicus] | gi\|149028168 (+1) | 71 | 7 | 0 | 5 | 5 | 2 |
| 1591 | Emd protein [Rattus norvegicus], gi\|149029876\|gb\|EDL84988.1\| emerin [Rattus norvegicus] | gi\|37589620 | 30 | 7 | 0 | 4 | 4 | 3 |
| 1592 | kinetochore associated 1 [Rattus norvegicus], gi\|149063297\|gb\|EDM13620.1\| kinetochore associated 1 (predicted) [Rattus norvegicus] | gi\|157821131 | 233 | 8 | 0 | 2 | 2 | 6 |
| 1593 | immunoglobulin superfamily, member 4D [Rattus norvegicus], gi\|225543378\|ref\|NP_001139449.1\| immunoglobulin superfamily, member 4D isoform 2 [Mus musculus], gi\|26338381\|dbj\|BAC32876.1\| unnamed protein product [Mus musculus], gi\|82619336\|gb\|ABB85363.1\| nect | gi\|114052913 (+1) | 44 | 11 | 0 | 0 | 0 | 11 |
| 1594 | PREDICTED: similar to THUMP domain containing 3 [Rattus norvegicus], gi\|109474037\|ref\|XP_001078607.1\| PREDICTED: similar to THUMP domain containing 3 [Rattus norvegicus] | gi\|109472464 (+1) | 70 | 7 | 2 | 0 | 1 | 6 |
| 1595 | vacuolar protein sorting 33 homolog A [Rattus norvegicus], gi\|77415393\|gb\|AAI05753.1\| Vacuolar protein sorting 33 homolog A (S. cerevisiae) [Rattus norvegicus], gi\|149063306\|gb\|EDM13629.1\| vacuolar protein sorting 33A (yeast), isoform CRA_a [Rattus norveg | gi\|155369738 (+1) | 67 | 7 | 0 | 5 | 5 | 2 |
| 1596 | melanocyte proliferating gene 1 [Rattus norvegicus], gi\|81884070\|sp\|Q641W2.1\|MYG1_RAT RecName: Full=UPF0160 protein MYG1, mitochondrial; Flags: Precursor, gi\|51980347\|gb\|AAH82112.1\| Melanocyte proliferating gene 1 [Rattus norvegicus], gi\|149031933\|gb\|EDL8 | gi\|53850618 | 43 | 7 | 5 | 0 | 2 | 5 |
| 1597 | RecName: Full=Complement C3; Contains: RecName: Full=Complement C3 beta chain; Contains: RecName: Full=Complement C3 alpha chain; Contains: RecName: Full=C3a anaphylatoxin; Contains: RecName: Full=Complement C3b alpha' chain; Contains: RecName: Full=Compl | gi\|116597 (+1) | 186 | 6 | 4 | 0 | 1 | 5 |
| 1598 | integrin beta 1 [Rattus norvegicus], gi\|124298010\|gb\|AAI31846.1\| Integrin beta 1 (fibronectin receptor beta) [Rattus norvegicus], gi\|149043255\|gb\|EDL96787.1\| integrin beta 1 (fibronectin receptor beta) [Rattus norvegicus] | gi\|158303324 | 88 | 12 | 0 | 0 | 0 | 12 |
| 1599 | leucine zipper and CTNNBIP1 domain containing [Rattus norvegicus], gi\|81883247\|sp\|Q5PQN7.1\|LZIC_RAT RecName: Full=Protein LZIC; AltName: Full=Leucine zipper and ICAT homologous domain-containing protein; AltName: Full=Leucine zipper and CTNNBIP1 domain-co | gi\|61557405 | 21 | 11 | 2 | 0 | 1 | 10 |
| 1600 | hypothetical protein LOC362643 [Rattus norvegicus], gi\|149024425\|gb\|EDL80922.1\| similar to KIAA0090 protein (predicted), isoform CRA_a [Rattus norvegicus] | gi\|157822661 | 111 | 11 | 0 | 2 | 2 | 9 |
| 1601 | rCG39515, isoform CRA_a [Rattus norvegicus] | gi\|149068862 (+3) | 101 | 7 | 0 | 2 | 2 | 5 |
| 1602 | transmembrane protein 35 [Rattus norvegicus], gi\|81863796\|sp\|Q6JAM9.1\|TMM35_RAT RecName: Full=Transmembrane protein 35; AltName: Full=Spinal cord expression protein 4, gi\|47013847\|gb\|AAT08467.1\| spinal cord expression protein 4 [Rattus norvegicus], gi\|149 | gi\|49169970 | 18 | 10 | 0 | 0 | 0 | 10 |
| 1603 | tetratricopeptide repeat domain 3 (predicted), isoform CRA_b [Rattus norvegicus] | gi\|149017707 (+3) | 224 | 6 | 0 | 6 | 6 | 0 |
| 1604 | hydroxysteroid dehydrogenase like 1 [Rattus norvegicus], gi\|81908672\|sp\|Q4V8B7.1\|HSDL1_RAT RecName: Full=Hydroxysteroid dehydrogenase-like protein 1, gi\|66911469\|gb\|AAH97457.1\| Hydroxysteroid dehydrogenase like 1 [Rattus norvegicus], gi\|149038311\|gb\|EDL92 | gi\|67846070 | 37 | 9 | 0 | 0 | 0 | 9 |
| 1605 | ribosomal protein S18 [Mus musculus], gi\|11968182\|ref\|NP_072045.1\| ribosomal protein S18 [Homo sapiens], gi\|47087103\|ref\|NP_998722.1\| ribosomal protein S18 [Rattus norvegicus], gi\|47522714\|ref\|NP_999105.1\| ribosomal protein S18 [Sus scrofa], gi\|75812924\|r | gi\|6755368 | 18 | 10 | 0 | 0 | 0 | 10 |
| 1606 | synuclein, gamma, isoform CRA_b [Rattus norvegicus] | gi\|149034107 (+3) | 16 | 11 | 0 | 0 | 0 | 11 |
| 1607 | small nuclear ribonucleoprotein polypeptide D3 [Homo sapiens], gi\|13385598\|ref\|NP_080371.1\| small nuclear ribonucleoprotein D3 [Mus musculus], gi\|56119060\|ref\|NP_001007839.1\| small nuclear ribonucleoprotein D3 polypeptide 18kDa [Gallus gallus], gi\|6285924 | gi\|4759160 | 14 | 12 | 0 | 0 | 0 | 12 |
| 1608 | Bg135360 [Rattus norvegicus] | gi\|33086696 | 68 | 11 | 0 | 0 | 0 | 11 |
| 1609 | heat shock protein 70kDa family, member 13 [Rattus norvegicus], gi\|67461586\|sp\|O35162.2\|HSP13_RAT RecName: Full=Heat shock 70 kDa protein 13; AltName: Full=Stress 70 protein chaperone microsome-associated 60 kDa protein; AltName: Full=Microsomal stress 70 | gi\|148747550 | 52 | 7 | 0 | 0 | 0 | 7 |
| 1610 | neuronal growth regulator 1 [Rattus norvegicus], gi\|8134522\|sp\|Q9Z0J8.1\|NEGR1_RAT RecName: Full=Neuronal growth regulator 1; AltName: Full=Kindred of IgLON; Short=Kilon protein; Flags: Precursor, gi\|4519558\|dbj\|BAA75649.1\| Kilon [Rattus norvegicus], gi\|14 | gi\|11067409 (+2) | 38 | 11 | 0 | 0 | 0 | 11 |
| 1611 | leucine rich repeat containing 59 [Rattus norvegicus], gi\|81910029\|sp\|Q5RJR8.1\|LRC59_RAT RecName: Full=Leucine-rich repeat-containing protein 59; AltName: Full=Protein p34, gi\|480379\|pir\|\|S36779 ribosome-binding protein p34 - rat, gi\|534876\|dbj\|BAA02786.1 | gi\|56605670 | 35 | 8 | 0 | 0 | 0 | 8 |
| 1612 | N-myc downstream regulated gene 4 [Rattus norvegicus], gi\|8927964\|sp\|Q9Z2L9.1\|NDRG4_RAT RecName: Full=Protein NDRG4; AltName: Full=Brain development-related molecule 1, gi\|4105412\|gb\|AAD02415.1\| development-related protein [Rattus norvegicus], gi\|31249540 | gi\|14010841 (+1) | 38 | 12 | 0 | 0 | 0 | 12 |
| 1613 | hypothetical protein LOC308869 [Rattus norvegicus], gi\|81885329\|sp\|Q6P791.1\|CK059_RAT RecName: Full=UPF0404 protein C11orf59 homolog, gi\|38197700\|gb\|AAH61783.1\| Hypothetical LOC308869 [Rattus norvegicus], gi\|149068692\|gb\|EDM18244.1\| Unknown (protein for M | gi\|40018580 | 18 | 12 | 0 | 0 | 0 | 12 |
| 1614 | phosphohistidine phosphatase 1 [Rattus norvegicus], gi\|149039343\|gb\|EDL93563.1\| phosphohistidine phosphatase 1 (predicted), isoform CRA_a [Rattus norvegicus] | gi\|164663846 | 14 | 9 | 0 | 0 | 0 | 9 |
| 1615 | keratin 35 [Rattus norvegicus], gi\|46485078\|tpg\|DAA04476.1\| TPA_exp: type I hair keratin KA30 [Rattus norvegicus], gi\|149054194\|gb\|EDM06011.1\| rCG35153 [Rattus norvegicus] | gi\|57012430 | 51 | 11 | 0 | 0 | 0 | 11 |
| 1616 | PREDICTED: similar to Temporarily Assigned Gene name family member (tag-241) [Rattus norvegicus] | gi\|109457682 (+2) | 179 | 11 | 0 | 1 | 1 | 10 |
| 1617 | zinc finger protein 259 [Rattus norvegicus], gi\|149041558\|gb\|EDL95399.1\| similar to zinc finger protein (predicted) [Rattus norvegicus] | gi\|212549649 | 51 | 6 | 4 | 1 | 2 | 4 |
| 1618 | similar to PDZ-domain protein scribble (predicted), isoform CRA_c [Rattus norvegicus] | gi\|149066146 (+2) | 177 | 4 | 2 | 3 | 4 | 0 |
| 1619 | DnaJ (Hsp40) homolog, subfamily C, member 6 [Rattus norvegicus], gi\|149044571\|gb\|EDL97830.1\| DnaJ (Hsp40) homolog, subfamily C, member 6 (predicted) [Rattus norvegicus] | gi\|157822123 | 99 | 7 | 0 | 2 | 2 | 5 |
| 1620 | NADH dehydrogenase (ubiquinone) Fe-S protein 3 (predicted), isoform CRA_b [Rattus norvegicus] | gi\|149022594 (+1) | 34 | 10 | 0 | 2 | 2 | 8 |
| 1621 | PREDICTED: similar to calsyntenin 1 [Rattus norvegicus] | gi\|109475855 (+3) | 123 | 5 | 3 | 0 | 1 | 4 |
| 1622 | rCG27700, isoform CRA_b [Rattus norvegicus] | gi\|149044024 | 81 | 8 | 0 | 3 | 3 | 5 |
| 1623 | dynactin 6 [Rattus norvegicus], gi\|149057926\|gb\|EDM09169.1\| dynactin 6 (predicted), isoform CRA_b [Rattus norvegicus] | gi\|157823924 | 21 | 9 | 0 | 2 | 2 | 7 |
| 1624 | rCG30605, isoform CRA_a [Rattus norvegicus], gi\|149024765\|gb\|EDL81262.1\| rCG30605, isoform CRA_a [Rattus norvegicus] | gi\|149024764 | 14 | 9 | 0 | 1 | 1 | 8 |
| 1625 | RNA binding motif protein 7 (predicted), isoform CRA_b [Rattus norvegicus] | gi\|149041583 | 30 | 8 | 0 | 1 | 1 | 7 |
| 1626 | synaptogyrin 3 (predicted), isoform CRA_b [Rattus norvegicus] | gi\|149052040 (+2) | 25 | 9 | 0 | 2 | 2 | 7 |
| 1627 | hypothetical protein LOC498890 [Rattus norvegicus], gi\|226501156\|ref\|NP_001141256.1\| hypothetical protein LOC100273343 [Zea mays], gi\|117558375\|gb\|AAI27541.1\| Similar to DNA segment, Chr 18, Wayne State University 98, expressed [Rattus norvegicus], gi\|149 | gi\|117940037 | 25 | 7 | 2 | 3 | 4 | 3 |
| 1628 | N-acetylneuraminic acid phosphatase [Rattus norvegicus], gi\|81883158\|sp\|Q5M969.1\|NANP_RAT RecName: Full=N-acylneuraminate-9-phosphatase; AltName: Full=Neu5Ac-9-Pase; AltName: Full=Haloacid dehalogenase-like hydrolase domain-containing protein 4, gi\|565410 | gi\|57863283 | 28 | 5 | 5 | 2 | 4 | 1 |
| 1629 | kinesin family member 15, isoform CRA_a [Rattus norvegicus] | gi\|149018140 (+2) | 160 | 3 | 7 | 1 | 3 | 0 |
| 1630 | phosphatidylinositol-4-phosphate 5-kinase, type I, gamma isoform c [Rattus norvegicus] | gi\|111185932 (+2) | 76 | 6 | 2 | 1 | 2 | 4 |
| 1631 | similar to Traf2 and NCK interacting kinase, splice variant 4 (predicted), isoform CRA_b [Rattus norvegicus] | gi\|149048594 (+1) | 154 | 7 | 0 | 3 | 3 | 4 |
| 1632 | protein phosphatase 2, regulatory subunit B', beta isoform [Rattus norvegicus], gi\|30575399\|gb\|AAP33143.1\| protein phosphatase 2A regulatory subunit B' beta isoform [Rattus norvegicus], gi\|60422755\|gb\|AAH90324.1\| Protein phosphatase 2, regulatory subunit | gi\|31077118 | 57 | 9 | 3 | 0 | 1 | 8 |
| 1633 | PREDICTED: similar to vacuolar protein sorting 13C protein isoform 1A [Rattus norvegicus] | gi\|109483673 | 400 | 5 | 1 | 4 | 4 | 1 |
| 1634 | similar to Epidermal growth factor receptor substrate 15-like 1 (Eps15-related protein) (Eps15R) (Epidermal growth factor receptor pathway substrate 15 related sequence) (Eps15-rs) [Rattus norvegicus] | gi\|149036175 (+1) | 99 | 8 | 2 | 0 | 1 | 7 |
| 1635 | WD repeat domain 81 [Rattus norvegicus], gi\|149053395\|gb\|EDM05212.1\| similar to hypothetical protein (predicted) [Rattus norvegicus] | gi\|197252058 | 212 | 9 | 0 | 1 | 1 | 8 |

| | | | | | | | | |
|---|---|---|---|---|---|---|---|---|
| 1636 | LOC498793 protein [Rattus norvegicus] | gi\|72679950 | 63 | 6 | 5 | 0 | 2 | 4 |
| 1637 | similar to RIKEN cDNA 0710008K08 (predicted), isoform CRA_a [Rattus norvegicus] | gi\|149057580 (+1) | 39 | 5 | 4 | 0 | 1 | 4 |
| 1638 | rCG22366, isoform CRA_a [Rattus norvegicus] | gi\|149046039 (+2) | 52 | 6 | 0 | 3 | 3 | 3 |
| 1639 | BRCA2 and CDKN1A interacting protein (predicted), isoform CRA_b [Rattus norvegicus] | gi\|149061343 | 36 | 9 | 2 | 0 | 1 | 8 |
| 1640 | PREDICTED: similar to jumonji domain containing 1B [Rattus norvegicus], gi\|149017194\|gb\|EDL76245.1\| rCG49501 [Rattus norvegicus] | gi\|109507127 | 191 | 2 | 9 | 0 | 3 | 0 |
| 1641 | PREDICTED: similar to Peroxisomal targeting signal 1 receptor (Peroxismore receptor 1) (Peroxisomal C-terminal targeting signal import receptor) (PTS1-BP) (Peroxin-5) (PTS1 receptor) (PXR1P) (PTS1R) [Rattus norvegicus] | gi\|109474193 (+2) | 81 | 5 | 2 | 0 | 1 | 4 |
| 1642 | PREDICTED: similar to tRNA nucleotidyl transferase, CCA-adding, 1 isoform 2 [Rattus norvegicus], gi\|109462731\|ref\|XP_001075311.1\| PREDICTED: similar to tRNA nucleotidyl transferase, CCA-adding, 1 isoform 3 [Rattus norvegicus] | gi\|109462729 (+1) | 50 | 3 | 5 | 0 | 2 | 1 |
| 1643 | exocyst complex component 5 [Rattus norvegicus], gi\|24418658\|sp\|P97878.1\|EXOC5_RAT RecName: Full=Exocyst complex component 5; AltName: Full=Exocyst complex component Sec10; AltName: Full=71 kDa component of rsec6/8 secretory complex; AltName: Full=p71, gi | gi\|11559976 | 82 | 6 | 0 | 2 | 2 | 4 |
| 1644 | N-myc downstream regulated gene 3 [Rattus norvegicus], gi\|81884642\|sp\|Q6AYR2.1\|NDRG3_RAT RecName: Full=Protein NDRG3, gi\|50926863\|gb\|AAH78946.1\| N-myc downstream regulated gene 3 [Rattus norvegicus] | gi\|62078575 | 42 | 6 | 3 | 0 | 1 | 5 |
| 1645 | solute carrier family 1 (glutamate/neutral amino acid transporter), member 4 [Rattus norvegicus], gi\|37718695\|dbj\|BAC99093.1\| neutral amino acid transporter ASCT1 [Rattus norvegicus], gi\|149044752\|gb\|EDL97938.1\| solute carrier family 1 (glutamate/neutral | gi\|38454264 | 56 | 6 | 0 | 3 | 3 | 3 |
| 1646 | hypothetical protein LOC362676 [Rattus norvegicus], gi\|149024781\|gb\|EDL81278.1\| similar to RIKEN cDNA 2810405K02 (predicted) [Rattus norvegicus] | gi\|157822867 | 22 | 8 | 1 | 0 | 0 | 8 |
| 1647 | rCG39653, isoform CRA_e [Rattus norvegicus] | gi\|149068166 (+1) | 15 | 6 | 1 | 0 | 0 | 6 |
| 1648 | survival motor neuron 1 [Rattus norvegicus], gi\|3024613\|sp\|O35876.1\|SMN_RAT RecName: Full=Survival motor neuron protein, gi\|2393771\|gb\|AAB96377.1\| survival of motor neuron [Rattus norvegicus] | gi\|11968084 (+3) | 31 | 5 | 0 | 3 | 3 | 2 |
| 1649 | neuropilin [Rattus norvegicus] | gi\|2407643 (+1) | 103 | 10 | 0 | 0 | 0 | 10 |
| 1650 | gi\|109483673-R | gi\|109483673-R (+1) | ? | 6 | 1 | 0 | 0 | 6 |
| 1651 | PREDICTED: similar to RIKEN cDNA 1110018J12 [Rattus norvegicus] | gi\|109478862 (+1) | 94 | 10 | 0 | 0 | 0 | 10 |
| 1652 | PREDICTED: similar to Versican core protein precursor (Large fibroblast proteoglycan) (Chondroitin sulfate proteoglycan core protein 2) (PG-M) [Rattus norvegicus] | gi\|109465804 (+3) | 367 | 10 | 0 | 0 | 0 | 10 |
| 1653 | RecName: Full=Mitochondrial fission 1 protein; AltName: Full=Fis1 homolog; Short=rFis1; AltName: Full=Tetratricopeptide repeat protein 11; AltName: Full=TPR repeat protein 11, gi\|149062977\|gb\|EDM13300.1\| fission 1 (mitochondrial outer membrane) homolog (y | gi\|94711371 | 17 | 9 | 2 | 0 | 1 | 8 |
| 1654 | engulfment and cell motility 1 isoform 1 [Mus musculus], gi\|157822513\|ref\|NP_001101885.1\| engulfment and cell motility 1 [Rattus norvegicus], gi\|30913074\|sp\|Q8BPU7.2\|ELMO1_MOUSE RecName: Full=Engulfment and cell motility protein 1; AltName: Full=CED-12 ho | gi\|17933766 (+1) | 84 | 4 | 0 | 3 | 3 | 1 |
| 1655 | rCG32197, isoform CRA_a [Rattus norvegicus] | gi\|149042757 | 15 | 9 | 1 | 0 | 0 | 9 |
| 1656 | N-myc downstream regulated gene 1 [Rattus norvegicus], gi\|81884862\|sp\|Q6JE36.1\|NDRG1_RAT RecName: Full=Protein NDRG1; AltName: Full=N-myc downstream-regulated gene 1 protein; Short=Protein Ndr1, gi\|45861619\|gb\|AAS78638.1\| N-myc downstream regulated 1 [Rat | gi\|58865550 | 43 | 10 | 1 | 0 | 0 | 10 |
| 1657 | RWD domain containing 1 [Rattus norvegicus], gi\|34098350\|sp\|Q99ND9.1\|RWDD1_RAT RecName: Full=RWD domain-containing protein 1; AltName: Full=Small androgen receptor-interacting protein, gi\|12331287\|emb\|CAC24710.1\| small androgen receptor-interacting protei | gi\|22129753 | 28 | 10 | 2 | 0 | 1 | 9 |
| 1658 | glycolipid transfer protein [Rattus norvegicus], gi\|205779849\|sp\|B0BNM9.1\|GLTP_RAT RecName: Full=Glycolipid transfer protein; Short=GLTP, gi\|149063607\|gb\|EDM13930.1\| glycolipid transfer protein (predicted) [Rattus norvegicus], gi\|165971328\|gb\|AAI58885.1\| | gi\|197313643 | 24 | 2 | 10 | 0 | 3 | 0 |
| 1659 | SAPS domain family, member 1 [Rattus norvegicus], gi\|149016659\|gb\|EDL75845.1\| SAPS domain family, member 1 (predicted) [Rattus norvegicus] | gi\|209529660 | 95 | 10 | 0 | 0 | 0 | 10 |
| 1660 | uridine-cytidine kinase 2 [Mus musculus], gi\|109498991\|ref\|XP_001076056.1\| PREDICTED: similar to uridine-cytidine kinase 2 [Rattus norvegicus], gi\|20455355\|sp\|Q99PM9.1\|UCK2_MOUSE RecName: Full=Uridine-cytidine kinase 2; Short=UCK 2; AltName: Full=Uridine | gi\|13507680 (+1) | 29 | 7 | 3 | 0 | 1 | 6 |
| 1661 | 107 kDa sialoglycoprotein [Rattus norvegicus] | gi\|56578 | 42 | 8 | 0 | 0 | 0 | 8 |
| 1662 | PREDICTED: similar to cyclin-dependent kinase 6 [Rattus norvegicus], gi\|109473069\|ref\|XP_001068770.1\| PREDICTED: similar to cyclin-dependent kinase 6 [Rattus norvegicus], gi\|149029083\|gb\|EDL84377.1\| cyclin-dependent kinase 6 [Rattus norvegicus] | gi\|62646797 | 37 | 6 | 4 | 0 | 1 | 5 |
| 1663 | hypothetical protein LOC304285 [Rattus norvegicus], gi\|123785689\|sp\|Q3ZBA0.1\|TCPR1_RAT RecName: Full=Tectonin beta-propeller repeat-containing protein 1, gi\|73695308\|gb\|AAI03479.1\| Similar to RIKEN cDNA 2210010N04 gene [Rattus norvegicus] | gi\|81295339 | 130 | 8 | 0 | 0 | 0 | 8 |
| 1664 | polymerase (RNA) III (DNA directed) polypeptide F, 39 kDa [Rattus norvegicus], gi\|149041225\|gb\|EDL95158.1\| polymerase (RNA) III (DNA directed) polypeptide F (predicted), isoform CRA_a [Rattus norvegicus] | gi\|157821329 | 42 | 9 | 0 | 0 | 0 | 9 |
| 1665 | anti-NGF30 antibody light-chain [Rattus norvegicus] | gi\|4096754 | 24 | 0 | 11 | 0 | 3 | 0 |
| 1666 | PREDICTED: similar to chloride ion pump-associated 55 kDa protein [Rattus norvegicus] | gi\|109507428 | 41 | 11 | 0 | 0 | 0 | 11 |
| 1667 | sodium-dependent high affinity glutamate transporter GLT-1A [Rattus norvegicus] | gi\|10121878 (+9) | 62 | 5 | 0 | 5 | 5 | 0 |
| 1668 | globin, alpha [Rattus norvegicus], gi\|1778047\|gb\|AAB40623.1\| alpha globin, gi\|149052190\|gb\|EDM04007.1\| rCG33691 [Rattus norvegicus] | gi\|62078447 | 16 | 11 | 0 | 0 | 0 | 11 |
| 1669 | phosphoserine phosphatase [Rattus norvegicus], gi\|62900749\|sp\|Q5M819.1\|SERB_RAT RecName: Full=Phosphoserine phosphatase; Short=PSPase; Short=PSP; AltName: Full=O-phosphoserine phosphohydrolase, gi\|56789499\|gb\|AAH88310.1\| Phosphoserine phosphatase [Rattus | gi\|57527332 | 25 | 2 | 9 | 0 | 3 | 0 |
| 1670 | transcription elongation factor B (SIII), polypeptide 2 [Mus musculus], gi\|13592105\|ref\|NP_112391.1\| transcription elongation factor B (SIII), polypeptide 2 [Rattus norvegicus], gi\|51317294\|sp\|P62870.1\|ELOB_RAT RecName: Full=Transcription elongation facto | gi\|13385800 | 13 | 9 | 0 | 0 | 0 | 9 |
| 1671 | electron-transfer-flavoprotein, alpha polypeptide [Rattus norvegicus], gi\|122065179\|sp\|P13803.4\|ETFA_RAT RecName: Full=Electron transfer flavoprotein subunit alpha, mitochondrial; Short=Alpha-ETF; Flags: Precursor, gi\|56789726\|gb\|AAH88412.1\| Electron-tran | gi\|57527204 | 35 | 10 | 1 | 0 | 0 | 10 |
| 1672 | lon peptidase 1, mitochondrial [Rattus norvegicus], gi\|81916424\|sp\|Q924S5.1\|LONM_RAT RecName: Full=Lon protease homolog, mitochondrial; AltName: Full=Lon protease-like protein; Short=LONP; AltName: Full=Mitochondrial ATP-dependent protease Lon; AltName: F | gi\|19173766 | 106 | 10 | 0 | 0 | 0 | 10 |
| 1673 | rCG57161 [Rattus norvegicus] | gi\|149035271 | 300 | 5 | 3 | 1 | 2 | 3 |
| 1674 | RAN binding protein 6 [Rattus norvegicus], gi\|149062682\|gb\|EDM13105.1\| rCG47920 [Rattus norvegicus] | gi\|157821101 | 125 | 8 | 1 | 2 | 2 | 6 |
| 1675 | rCG62367, isoform CRA_b [Rattus norvegicus] | gi\|149051499 (+1) | 61 | 6 | 2 | 1 | 2 | 4 |
| 1676 | similar to hypothetical protein (predicted) [Rattus norvegicus] | gi\|149055579 (+1) | 32 | 5 | 1 | 2 | 2 | 3 |
| 1677 | PREDICTED: similar to Myosin-6 (Myosin VI) [Rattus norvegicus] | gi\|109483723 (+1) | 148 | 3 | 4 | 1 | 2 | 1 |
| 1678 | PREDICTED: similar to ribosomal protein S6 kinase polypeptide 3 [Rattus norvegicus], gi\|109511791\|ref\|XP_001054840.1\| PREDICTED: similar to ribosomal protein S6 kinase polypeptide 3 [Rattus norvegicus] | gi\|109510713 (+1) | 97 | 5 | 1 | 2 | 2 | 3 |
| 1679 | FYN oncogene related to SRC, FGR, YES [Rattus norvegicus], gi\|126310436\|ref\|XP_001368891.1\| PREDICTED: similar to FYN oncogene related to SRC, FGR, YES isoform 2 [Monodelphis domestica], gi\|81863183\|sp\|Q62844.1\|FYN_RAT RecName: Full=Proto-oncogene tyrosin | gi\|6978863 | 61 | 6 | 0 | 0 | 0 | 6 |
| 1680 | coiled-coil domain containing 22 (predicted) [Rattus norvegicus] | gi\|149028461 (+1) | 72 | 5 | 0 | 2 | 2 | 3 |
| 1681 | ribonuclease/angiogenin inhibitor 1, isoform CRA_b [Rattus norvegicus] | gi\|149061561 (+1) | 54 | 5 | 5 | 0 | 2 | 3 |
| 1682 | rCG55996 [Rattus norvegicus] | gi\|149036645 | 96 | 4 | 1 | 0 | 0 | 4 |
| 1683 | interleukin enhancer binding factor 2 [Mus musculus], gi\|24234747\|ref\|NP_004506.2\| interleukin enhancer binding factor 2 [Homo sapiens], gi\|207079937\|ref\|NP_001128737.1\| DKFZP469C1132 protein [Pongo abelii], gi\|57089135\|ref\|XP_537263.1\| PREDICTED: similar | gi\|13385872 | 43 | 7 | 0 | 4 | 4 | 3 |
| 1684 | PREDICTED: similar to glycogen synthase 1, muscle isoform 3 [Rattus norvegicus] | gi\|109461930 (+3) | 84 | 5 | 0 | 4 | 4 | 1 |
| 1685 | phosphodiesterase 12 [Rattus norvegicus], gi\|37361834\|gb\|AAQ91030.1\| LRRGT00074 [Rattus norvegicus] | gi\|148539973 (+1) | 79 | 7 | 0 | 0 | 0 | 7 |
| 1686 | PREDICTED: similar to germinal histone H4 gene [Rattus norvegicus] | gi\|109504921 (+1) | 11 | 2 | 7 | 0 | 2 | 0 |
| 1687 | rCG30986, isoform CRA_a [Rattus norvegicus] | gi\|149024626 | 101 | 3 | 0 | 2 | 2 | 1 |
| 1688 | RAB9B, member RAS oncogene family [Rattus norvegicus], gi\|149033183\|gb\|EDL87990.1\| rCG56886 [Rattus norvegicus] | gi\|157824109 | 23 | 8 | 0 | 0 | 0 | 8 |
| 1689 | ASF1 anti-silencing function 1 homolog A [Rattus norvegicus], gi\|149038636\|gb\|EDL92925.1\| ASF1 anti-silencing function 1 homolog A (S. cerevisiae) (predicted), isoform CRA_a [Rattus norvegicus] | gi\|213512016 | 23 | 6 | 0 | 0 | 0 | 6 |
| 1690 | regulator of G-protein signalling 3, isoform CRA_c [Rattus norvegicus] | gi\|149059606 (+1) | 106 | 6 | 4 | 0 | 1 | 5 |
| 1691 | ErbB3-binding protein 1 [Rattus norvegicus], gi\|50925681\|gb\|AAH79095.1\| Proliferation-associated 2G4 [Rattus norvegicus], gi\|149029665\|gb\|EDL84836.1\| proliferation-associated 2G4, isoform CRA_a [Rattus norvegicus] | gi\|51948384 | 44 | 11 | 0 | 0 | 0 | 11 |
| 1692 | rCG25584 [Rattus norvegicus] | gi\|149018515 | 122 | 6 | 0 | 3 | 3 | 3 |
| 1693 | hypothetical protein LOC682999 [Rattus norvegicus], gi\|74196430\|dbj\|BAE34358.1\| unnamed protein product [Mus musculus], gi\|149040933\|gb\|EDL94890.1\| rCG20102, isoform CRA_a [Rattus norvegicus], gi\|171846582\|gb\|AAI61899.1\| LOC682999 protein [Rattus norvegic | gi\|189027115 | 35 | 7 | 0 | 3 | 3 | 4 |
| 1694 | PREDICTED: similar to GCN1 general control of amino-acid synthesis 1-like 1 isoform 2 [Rattus norvegicus] | gi\|109496005 (+1) | 293 | 7 | 2 | 0 | 1 | 6 |
| 1695 | alpha-internexin [Rattus norvegicus] | gi\|55622 (+1) | 56 | 6 | 0 | 5 | 5 | 1 |
| 1696 | exportin, tRNA (nuclear export receptor for tRNAs) (predicted), isoform CRA_b [Rattus norvegicus] | gi\|149066813 (+1) | 79 | 0 | 5 | 0 | 2 | 0 |
| 1697 | PREDICTED: similar to Transcription elongation factor SPT6 [Rattus norvegicus], gi\|109491416\|ref\|XP_001080796.1\| PREDICTED: similar to Transcription elongation factor SPT6 [Rattus norvegicus] | gi\|62656585 | 199 | 3 | 5 | 0 | 2 | 1 |

| | | | | | | | | |
|---|---|---|---|---|---|---|---|---|
| 1698 | zinc finger, CCHC domain containing 8 (predicted), isoform CRA_b [Rattus norvegicus] | gi\|149063302 (+1) | 77 | 6 | 0 | 5 | 5 | 1 |
| 1699 | PREDICTED: similar to Protein C9orf64 [Rattus norvegicus], gi\|109505424\|ref\|XP_001067219.1\| PREDICTED: similar to Protein C9orf64 [Rattus norvegicus], gi\|149039795\|gb\|EDL93911.1\| similar to CG9752-PA [Rattus norvegicus] | gi\|62663171 | 39 | 7 | 3 | 0 | 1 | 6 |
| 1700 | PREDICTED: similar to SEC24 related gene family, member C isoform 5 [Rattus norvegicus] | gi\|109502089 (+3) | 121 | 10 | 0 | 0 | 0 | 10 |
| 1701 | nitric oxide synthase interacting protein (predicted), isoform CRA_a [Rattus norvegicus] | gi\|149055986 (+1) | 30 | 2 | 0 | 2 | 2 | 0 |
| 1702 | Inositol (myo)-1(or 4)-monophosphatase 1, isoform CRA_a [Rattus norvegicus], gi\|149048448\|gb\|EDM00989.1\| Inositol (myo)-1(or 4)-monophosphatase 1, isoform CRA_a [Rattus norvegicus] | gi\|149048447 | 26 | 6 | 4 | 0 | 1 | 5 |
| 1703 | sorting nexin 17 [Rattus norvegicus], gi\|226506310\|ref\|NP_001142236.1\| hypothetical protein LOC100274405 [Zea mays], gi\|81884652\|sp\|Q6AYS6.1\|SNX17_RAT RecName: Full=Sorting nexin-17, gi\|50926143\|gb\|AAH78931.1\| Sorting nexin 17 [Rattus norvegicus], gi\|1490 | gi\|58865530 | 53 | 11 | 0 | 0 | 0 | 11 |
| 1704 | tripartite motif-containing 32 [Rattus norvegicus], gi\|51858707\|gb\|AAH81980.1\| Tripartite motif protein 32 [Rattus norvegicus], gi\|60551553\|gb\|AAH91385.1\| Tripartite motif protein 32 [Rattus norvegicus], gi\|149059569\|gb\|EDM10507.1\| tripartite motif protei | gi\|58865776 | 72 | 7 | 0 | 3 | 3 | 4 |
| 1705 | plectin 1 [Rattus norvegicus], gi\|1709655\|sp\|P30427.2\|PLEC1_RAT RecName: Full=Plectin-1; Short=PLTN; Short=PCN, gi\|1561642\|emb\|CAA42169.1\| plectin [Rattus norvegicus] | gi\|13540714 (+12) | 534 | 3 | 0 | 3 | 3 | 0 |
| 1706 | DNA directed RNA polymerase II polypeptide D [Homo sapiens], gi\|21312246\|ref\|NP_081278.1\| polymerase (RNA) II (DNA directed) polypeptide D isoform 1 [Mus musculus], gi\|157820569\|ref\|NP_001102356.1\| polymerase (RNA) II (DNA directed) polypeptide D [Rattus | gi\|4758574 | 16 | 10 | 0 | 0 | 0 | 10 |
| 1707 | agrin [Rattus norvegicus] | gi\|149024867 (+3) | 206 | 7 | 0 | 0 | 0 | 7 |
| 1708 | carbonic anhydrase II [Rattus norvegicus], gi\|115459\|sp\|P27139.2\|CAH2_RAT RecName: Full=Carbonic anhydrase 2; AltName: Full=Carbonic anhydrase II; Short=CA-II; AltName: Full=Carbonate dehydratase II, gi\|55838\|emb\|CAA41227.1\| carbonic dehydratase [Rattus n | gi\|9506445 | 29 | 0 | 8 | 0 | 3 | 0 |
| 1709 | G protein-coupled receptor kinase interacting ArfGAP 2 [Rattus norvegicus], gi\|51859181\|gb\|AAH81967.1\| G protein-coupled receptor kinase interacting ArfGAP 2 [Rattus norvegicus] | gi\|53850634 | 85 | 11 | 0 | 0 | 0 | 11 |
| 1710 | unknown [Rattus norvegicus] | gi\|109692276 (+1) | 23 | 4 | 4 | 0 | 1 | 3 |
| 1711 | PREDICTED: similar to Huntingtin interacting protein K [Equus caballus], gi\|194676962\|ref\|XP_001789959.1\| PREDICTED: similar to Huntingtin interacting protein K [Bos taurus], gi\|149023106\|gb\|EDL80000.1\| similar to RIKEN cDNA 2310003F16 (predicted) [Rattus | gi\|149692007 | 14 | 8 | 0 | 0 | 0 | 8 |
| 1712 | PREDICTED: similar to Phosphoribosylformylglycinamidine synthase (FGAM synthase) (FGAMS) (Formylglycinamide ribotide amidotransferase) (FGARAT) (Formylglycinamide ribotide synthetase) [Rattus norvegicus] | gi\|109516321 | 37 | 4 | 7 | 0 | 2 | 2 |
| 1713 | PREDICTED: similar to SWI/SNF-related matrix-associated actin-dependent regulator of chromatin c2 isoform b isoform 2 [Rattus norvegicus] | gi\|109480098 (+2) | 125 | 8 | 1 | 0 | 0 | 8 |
| 1714 | latexin [Rattus norvegicus], gi\|61227509\|sp\|Q64361.1\|LXN_RAT RecName: Full=Latexin; AltName: Full=Endogenous carboxypeptidase inhibitor; Short=ECI; AltName: Full=Tissue carboxypeptidase inhibitor; Short=TCI, gi\|440576\|emb\|CAA54290.1\| latexin [Rattus norve | gi\|14269568 (+1) | 26 | 6 | 5 | 0 | 2 | 4 |
| 1715 | adaptor-related protein complex 3, sigma 1 subunit [Rattus norvegicus], gi\|149064199\|gb\|EDM14402.1\| adaptor-related protein complex 3, sigma 1 subunit (predicted), isoform CRA_a [Rattus norvegicus] | gi\|157818103 (+1) | 18 | 9 | 0 | 0 | 0 | 9 |
| 1716 | PREDICTED: similar to actin related protein 2/3 complex, subunit 5-like [Rattus norvegicus], gi\|109487031\|ref\|XP_001059654.1\| PREDICTED: similar to actin related protein 2/3 complex, subunit 5-like [Rattus norvegicus], gi\|143802284\|sp\|A1L108.2\|ARP5L_RAT R | gi\|109486016 (+2) | 17 | 7 | 0 | 0 | 0 | 7 |
| 1717 | procollagen, type V, alpha 1 [Rattus norvegicus] | gi\|149039207 (+1) | 184 | 0 | 8 | 0 | 3 | 0 |
| 1718 | phosphorylated adaptor for RNA export, isoform CRA_a [Rattus norvegicus] | gi\|149064291 (+2) | 40 | 9 | 0 | 0 | 0 | 9 |
| 1719 | coiled-coil domain containing 25 [Rattus norvegicus], gi\|149030311\|gb\|EDL85367.1\| coiled-coil domain containing 25 (predicted) [Rattus norvegicus] | gi\|157821639 | 24 | 2 | 0 | 0 | 0 | 2 |
| 1720 | 5'-nucleotidase, cytosolic III [Rattus norvegicus], gi\|149033245\|gb\|EDL88046.1\| 5'-nucleotidase, cytosolic III (predicted), isoform CRA_a [Rattus norvegicus], gi\|149033247\|gb\|EDL88048.1\| 5'-nucleotidase, cytosolic III (predicted), isoform CRA_a [Rattus no | gi\|157823103 (+1) | 34 | 10 | 0 | 0 | 0 | 10 |
| 1721 | PREDICTED: similar to SH3-domain GRB2-like (endophilin) interacting protein 1 [Rattus norvegicus], gi\|109476819\|ref\|XP_001058167.1\| PREDICTED: similar to SH3-domain GRB2-like (endophilin) interacting protein 1 [Rattus norvegicus] | gi\|62649304 | 86 | 6 | 2 | 0 | 1 | 5 |
| 1722 | fatty acid binding protein FABP-1 {N-terminal} [rats, Sprague-Dawley, liver, Peptide, 35 aa] | gi\|452977 | 4 | 10 | 0 | 0 | 0 | 10 |
| 1723 | secretogranin III, isoform CRA_a [Rattus norvegicus] | gi\|149019137 (+2) | 53 | 8 | 0 | 0 | 0 | 8 |
| 1724 | mitogen-activated protein kinase kinase 1 [Rattus norvegicus], gi\|266566\|sp\|Q01986.2\|MP2K1_RAT RecName: Full=Dual specificity mitogen-activated protein kinase kinase 1; Short=MAP kinase kinase 1; Short=MAPKK 1; AltName: Full=ERK activator kinase 1; AltNam | gi\|13928886 | 43 | 3 | 2 | 2 | 3 | 0 |
| 1725 | PREDICTED: similar to CG32732-PA [Rattus norvegicus], gi\|109479948\|ref\|XP_001069129.1\| PREDICTED: similar to CG32732-PA [Rattus norvegicus], gi\|149044195\|gb\|EDL97577.1\| rCG27725, isoform CRA_a [Rattus norvegicus] | gi\|109478621 (+1) | 67 | 8 | 1 | 0 | 0 | 8 |
| 1726 | catenin (cadherin associated protein), delta 1 (predicted), isoform CRA_b [Rattus norvegicus] | gi\|149022412 (+1) | 102 | 5 | 0 | 2 | 2 | 3 |
| 1727 | pallidin homolog [Rattus norvegicus], gi\|66910642\|gb\|AAH97469.1\| Pallidin homolog (mouse) [Rattus norvegicus], gi\|149023154\|gb\|EDL80048.1\| pallidin [Rattus norvegicus] | gi\|71043868 | 20 | 8 | 0 | 2 | 2 | 6 |
| 1728 | GABA transporter protein [Rattus norvegicus], gi\|30520131\|ref\|NP_848818.1\| solute carrier family 6 (neurotransmitter transporter, GABA), member 1 [Mus musculus], gi\|128609\|sp\|P23978.1\|SC6A1_RAT RecName: Full=Sodium- and chloride-dependent GABA transporter | gi\|13242269 | 67 | 9 | 0 | 0 | 0 | 9 |
| 1729 | timeless homolog [Rattus norvegicus], gi\|61216883\|sp\|Q9Z2Y1.1\|TIM_RAT RecName: Full=Protein timeless homolog; Short=rTIM; AltName: Full=Timeless-like protein; Short=rTLP, gi\|3869211\|dbj\|BAA34400.1\| rTIM [Rattus norvegicus] | gi\|13786174 (+1) | 139 | 4 | 3 | 1 | 2 | 2 |
| 1730 | PREDICTED: similar to chromodomain helicase DNA binding protein 4 [Rattus norvegicus] | gi\|109472581 (+2) | 206 | 4 | 1 | 2 | 2 | 2 |
| 1731 | rCG61894, isoform CRA_a [Rattus norvegicus] | gi\|149050639 (+1) | 86 | 5 | 1 | 2 | 2 | 3 |
| 1732 | ATG3 autophagy related 3 homolog [Rattus norvegicus], gi\|9957034\|gb\|AAG09182.1\|AF175224_1 preconditioning-inducible gene 1 protein [Rattus norvegicus] | gi\|19705511 (+1) | 36 | 6 | 0 | 4 | 4 | 2 |
| 1733 | copine III (predicted), isoform CRA_b [Rattus norvegicus] | gi\|149045496 | 60 | 6 | 0 | 0 | 0 | 6 |
| 1734 | adaptor-related protein complex 1, sigma 1 subunit [Bos taurus], gi\|114615097\|ref\|XP_001152893.1\| PREDICTED: similar to clathrin-associated protein 19 isoform 1 [Pan troglodytes], gi\|114615099\|ref\|XP_001152948.1\| PREDICTED: similar to clathrin-associated | gi\|116004439 (+1) | 19 | 10 | 0 | 0 | 0 | 10 |
| 1735 | similar to dJ202D23.2 (novel protein similar to C21ORF5 (KIAA0933)) (predicted), isoform CRA_a [Rattus norvegicus] | gi\|149018991 (+2) | 212 | 5 | 0 | 4 | 4 | 1 |
| 1736 | Nedd8 ultimate buster-1 [Rattus norvegicus], gi\|53733414\|gb\|AAH83586.1\| Negative regulator of ubiquitin-like proteins 1 [Rattus norvegicus] | gi\|62078579 | 70 | 7 | 0 | 1 | 1 | 6 |
| 1737 | RAB geranylgeranyltransferase, beta subunit [Rattus norvegicus], gi\|730317\|sp\|Q08603.1\|PGTB2_RAT RecName: Full=Geranylgeranyl transferase type-2 subunit beta; AltName: Full=Geranylgeranyl transferase type II subunit beta; Short=GGTase-II-beta; AltName: Fu | gi\|20177500 (+2) | 37 | 3 | 5 | 0 | 2 | 1 |
| 1738 | asparagine synthetase domain containing 1 [Rattus norvegicus], gi\|149046228\|gb\|EDL99121.1\| similar to HCV NS3-transactivated protein 1 (predicted), isoform CRA_b [Rattus norvegicus] | gi\|157823035 | 69 | 6 | 2 | 2 | 3 | 3 |
| 1739 | PREDICTED: similar to DEAH (Asp-Glu-Ala-Asp/His) box polypeptide 57 homolog [Rattus norvegicus], gi\|109478937\|ref\|XP_001062787.1\| PREDICTED: similar to DEAH (Asp-Glu-Ala-Asp/His) box polypeptide 57 homolog [Rattus norvegicus] | gi\|109477854 | 156 | 4 | 1 | 4 | 4 | 0 |
| 1740 | farnesyl diphosphate farnesyl transferase 1 [Rattus norvegicus], gi\|399484\|sp\|Q02769.1\|FDFT_RAT RecName: Full=Squalene synthetase; Short=SQS; Short=SS; AltName: Full=Farnesyl-diphosphate farnesyltransferase; AltName: Full=FPP:FPP farnesyltransferase, gi\|2 | gi\|9506591 | 48 | 7 | 0 | 0 | 0 | 7 |
| 1741 | polymerase (RNA) III (DNA directed) polypeptide C (62kD) [Rattus norvegicus], gi\|81883800\|sp\|Q5XIL3.1\|RPC3_RAT RecName: Full=DNA-directed RNA polymerase III subunit RPC3; Short=RNA polymerase III subunit C3; AltName: Full=DNA-directed RNA polymerase III s | gi\|58865732 | 61 | 9 | 0 | 0 | 0 | 9 |
| 1742 | rCG40279 [Rattus norvegicus] | gi\|149067611 | 65 | 9 | 0 | 0 | 0 | 9 |
| 1743 | thyroid hormone receptor interactor 13 [Rattus norvegicus], gi\|81883667\|sp\|Q5XHZ9.1\|TRP13_RAT RecName: Full=Thyroid receptor-interacting protein 13; AltName: Full=Thyroid hormone receptor interactor 13; Short=TRIP-13, gi\|53733607\|gb\|AAH83900.1\| Thyroid ho | gi\|58865438 | 48 | 8 | 0 | 0 | 0 | 8 |
| 1744 | tubulin folding cofactor C [Rattus norvegicus], gi\|149069425\|gb\|EDM18866.1\| tubulin-specific chaperone c (predicted) [Rattus norvegicus], gi\|183986260\|gb\|AAI66467.1\| Tubulin folding cofactor C [Rattus norvegicus] | gi\|157816975 | 38 | 8 | 2 | 0 | 1 | 7 |
| 1745 | COMM domain containing 4 (predicted), isoform CRA_c [Rattus norvegicus] | gi\|149041769 (+1) | 22 | 8 | 0 | 0 | 0 | 8 |
| 1746 | peroxisomal multifunctional enzyme type II | gi\|1592545 (+2) | 79 | 7 | 0 | 0 | 0 | 7 |
| 1747 | PREDICTED: similar to Rap1 GTPase-activating protein 1 (Rap1GAP) [Rattus norvegicus], gi\|109477546\|ref\|XP_001070178.1\| PREDICTED: similar to Rap1 GTPase-activating protein 1 (Rap1GAP) [Rattus norvegicus] | gi\|109475701 (+2) | 83 | 2 | 5 | 0 | 2 | 0 |
| 1748 | hypothetical protein LOC292267 [Rattus norvegicus], gi\|81884656\|sp\|Q6AYT5.1\|CF211_RAT RecName: Full=UPF0364 protein C6orf211 homolog, gi\|50927323\|gb\|AAH78920.1\| Similar to RIKEN cDNA 1700052N19 [Rattus norvegicus], gi\|149038532\|gb\|EDL92862.1\| similar to R | gi\|62945250 | 50 | 4 | 3 | 0 | 1 | 3 |
| 1749 | kelch-like 22 [Rattus norvegicus], gi\|149019756\|gb\|EDL77904.1\| kelch-like 22 (Drosophila) (predicted) [Rattus norvegicus] | gi\|157819093 | 72 | 8 | 0 | 2 | 2 | 6 |
| 1750 | proline, glutamic acid and leucine rich protein 1 [Rattus norvegicus] | gi\|149053184 (+1) | 67 | 5 | 0 | 2 | 2 | 3 |
| 1751 | EH-domain containing 3 [Rattus norvegicus], gi\|81915054\|sp\|Q8R491.2\|EHD3_RAT RecName: Full=EH domain-containing protein 3, gi\|34452241\|gb\|AAM14604.2\|AF494093_1 EH-domain containing protein 2 [Rattus norvegicus], gi\|149050680\|gb\|EDM02853.1\| rCG61532 [Rattu | gi\|34536836 | 61 | 4 | 0 | 2 | 2 | 2 |
| 1752 | PTK7 protein tyrosine kinase 7 [Rattus norvegicus], gi\|149069388\|gb\|EDM18829.1\| serum response factor (predicted) [Rattus norvegicus] | gi\|157818047 | 86 | 3 | 0 | 1 | 1 | 2 |
| 1753 | pyroglutamyl-peptidase I [Rattus norvegicus], gi\|81870932\|sp\|Q76IC5.1\|PGPI_RAT RecName: Full=Pyroglutamyl-peptidase 1; AltName: Full=Pyroglutamyl-peptidase I; Short=PGP-I; AltName: Full=Pyrrolidone-carboxylate peptidase; AltName: Full=5-oxoprolyl-peptidas | gi\|42558275 | 23 | 8 | 2 | 0 | 1 | 7 |
| 1754 | cell line NK14 derived transforming oncogene [Mus musculus], gi\|162287395\|ref\|NP_446450.2\| RAB8A, member RAS oncogene family [Rattus norvegicus], gi\|82592519\|sp\|P55258.2\|RAB8A_MOUSE RecName: Full=Ras-related protein Rab-8A; AltName: Full=Oncogene c-mel, g | gi\|38372905 (+1) | 24 | 5 | 0 | 0 | 0 | 5 |

| | | | | | | | | |
|---|---|---|---|---|---|---|---|---|
| 1755 | keratin 42 [Rattus norvegicus], gi\|81891673\|sp\|Q6IFU7.1\|K1C42_RAT RecName: Full=Keratin, type I cytoskeletal 42; AltName: Full=Cytokeratin-42; Short=CK-42; AltName: Full=Keratin-42; Short=K42; AltName: Full=Type I keratin Ka22, gi\|46485096\|tpg\|DAA04485.1\| | gi\|57012446 | 50 | 9 | 0 | 0 | 0 | 9 |
| 1756 | rCG45963 [Rattus norvegicus] | gi\|149058268 (+1) | 49 | 1 | 7 | 0 | 2 | 0 |
| 1757 | rCG20046 [Rattus norvegicus], gi\|149030792\|gb\|EDL85823.1\| G protein-coupled receptor associated sorting protein 1 [Rattus norvegicus] | gi\|149030791 (+1) | 152 | 5 | 0 | 5 | 5 | 0 |
| 1758 | a disintegrin and metalloprotease domain 8 (predicted), isoform CRA_e [Rattus norvegicus] | gi\|149061448 (+1) | 89 | 3 | 0 | 3 | 3 | 0 |
| 1759 | deoxycytidine kinase [Rattus norvegicus] | gi\|149033733 | 31 | 2 | 5 | 0 | 2 | 0 |
| 1760 | KDEL (Lys-Asp-Glu-Leu) endoplasmic reticulum protein retention receptor 1, isoform CRA_a [Rattus norvegicus] | gi\|149055856 (+2) | 25 | 8 | 0 | 0 | 0 | 8 |
| 1761 | PREDICTED: similar to High mobility group protein 4 (HMG-4) (High mobility group protein 2a) (HMG-2a) [Rattus norvegicus], gi\|109514150\|ref\|XP_001067329.1\| PREDICTED: similar to High mobility group protein 4 (HMG-4) (High mobility group protein 2a) (HMG-2 | gi\|109511369 (+1) | 62 | 0 | 9 | 0 | 3 | 0 |
| 1762 | proteasome (prosome, macropain) 26S subunit, non-ATPase, 10, isoform CRA_b [Rattus norvegicus] | gi\|149022066 (+1) | 25 | 6 | 0 | 0 | 0 | 6 |
| 1763 | enoyl Coenzyme A hydratase, short chain, 1, mitochondrial, isoform CRA_b [Rattus norvegicus] | gi\|149061475 (+4) | 16 | 8 | 0 | 0 | 0 | 8 |
| 1764 | serine (or cysteine) proteinase inhibitor, clade H, member 1, isoform CRA_a [Rattus norvegicus], gi\|149068860\|gb\|EDM18412.1\| serine (or cysteine) proteinase inhibitor, clade H, member 1, isoform CRA_a [Rattus norvegicus] | gi\|149068858 (+2) | 45 | 9 | 0 | 0 | 0 | 9 |
| 1765 | RecName: Full=Cathepsin D; Contains: RecName: Full=Cathepsin D 12 kDa light chain; Contains: RecName: Full=Cathepsin D 9 kDa light chain; Contains: RecName: Full=Cathepsin D 34 kDa heavy chain; Contains: RecName: Full=Cathepsin D 30 kDa heavy chain; Flags | gi\|115720 (+1) | 45 | 9 | 0 | 0 | 0 | 9 |
| 1766 | leucine zipper-EF-hand containing transmembrane protein 1 [Rattus norvegicus], gi\|62510718\|sp\|Q5XIN6.1\|LETM1_RAT RecName: Full=LETM1 and EF-hand domain-containing protein 1, mitochondrial; AltName: Full=Leucine zipper-EF-hand-containing transmembrane prot | gi\|54400736 | 83 | 10 | 0 | 0 | 0 | 10 |
| 1767 | calcitonin gene-related peptide-receptor component protein [Rattus norvegicus], gi\|20532031\|sp\|Q8VHM6.1\|RPC9_RAT RecName: Full=DNA-directed RNA polymerase III subunit RPC9; Short=RNA polymerase III subunit C9; AltName: Full=Calcitonin gene-related peptide | gi\|19526763 | 17 | 9 | 0 | 0 | 0 | 9 |
| 1768 | PREDICTED: similar to Complement C5 precursor (Hemolytic complement) [Rattus norvegicus] | gi\|109468076 (+2) | 190 | 9 | 1 | 0 | 0 | 9 |
| 1769 | pregnancy-zone protein [Rattus norvegicus], gi\|205384\|gb\|AAA41591.1\| alpha-1-macroglobulin | gi\|21955142 (+1) | 167 | 6 | 2 | 0 | 1 | 5 |
| 1770 | nuclear casein kinase and cyclin-dependent kinase substrate 1 [Rattus norvegicus], gi\|13631502\|sp\|Q9EPJ0.1\|NUCKS_RAT RecName: Full=Nuclear ubiquitous casein and cyclin-dependent kinases substrate, gi\|12054818\|emb\|CAC20862.1\| nuclear casein kinase and cycl | gi\|12248185 | 27 | 0 | 7 | 0 | 2 | 0 |
| 1771 | roundabout 1 [Rattus norvegicus], gi\|49036462\|sp\|O55005.1\|ROBO1_RAT RecName: Full=Roundabout homolog 1; Flags: Precursor, gi\|2811216\|gb\|AAC39960.1\| transmembrane receptor Robo1 [Rattus norvegicus] | gi\|11559953 | 181 | 8 | 0 | 0 | 0 | 8 |
| 1772 | transmembrane emp24 protein transport domain containing 7 [Rattus norvegicus], gi\|109507281\|ref\|XP_001054517.1\| PREDICTED: similar to transmembrane emp24 protein transport domain containing 7 [Rattus norvegicus], gi\|149064190\|gb\|EDM14393.1\| toll-like rece | gi\|157787208 | 25 | 9 | 0 | 0 | 0 | 9 |
| 1773 | synuclein, beta, isoform CRA_a [Rattus norvegicus], gi\|149039914\|gb\|EDL94030.1\| synuclein, beta, isoform CRA_a [Rattus norvegicus] | gi\|149039913 (+2) | 14 | 9 | 0 | 0 | 0 | 9 |
| 1774 | exocyst complex component 6B [Rattus norvegicus], gi\|149036560\|gb\|EDL91178.1\| rCG56008 [Rattus norvegicus] | gi\|157823569 | 94 | 6 | 0 | 0 | 0 | 6 |
| 1775 | rCG62592, isoform CRA_a [Rattus norvegicus] | gi\|149048118 (+2) | 117 | 4 | 1 | 2 | 2 | 2 |
| 1776 | ash2 (absent, small, or homeotic)-like [Rattus norvegicus], gi\|149057828\|gb\|EDM09071.1\| ash2 (absent, small, or homeotic)-like (Drosophila) (predicted) [Rattus norvegicus] | gi\|157821569 | 68 | 3 | 4 | 2 | 3 | 0 |
| 1777 | PREDICTED: similar to Endoplasmic reticulum-Golgi intermediate compartment protein 1 (ER-Golgi intermediate compartment 32 kDa protein) (ERGIC-32) [Rattus norvegicus] | gi\|109487945 (+2) | 40 | 6 | 0 | 1 | 1 | 5 |
| 1778 | tuberous sclerosis 1 [Rattus norvegicus], gi\|9297064\|sp\|Q9Z136.1\|TSC1_RAT RecName: Full=Hamartin; AltName: Full=Tuberous sclerosis 1 protein homolog, gi\|4512344\|dbj\|BAA75254.1\| Hamartin [Rattus norvegicus], gi\|149039183\|gb\|EDL93403.1\| tuberous sclerosis 1 | gi\|11177894 | 129 | 5 | 0 | 1 | 1 | 4 |
| 1779 | ubiquitin-conjugating enzyme E2C [Rattus norvegicus], gi\|149042923\|gb\|EDL96497.1\| rCG32249 [Rattus norvegicus] | gi\|157818349 | 20 | 3 | 2 | 0 | 1 | 2 |
| 1780 | tetratricopeptide repeat domain 9C, isoform CRA_b [Rattus norvegicus] | gi\|149062307 (+1) | 10 | 5 | 2 | 0 | 1 | 4 |
| 1781 | zuotin related factor 2 [Rattus norvegicus], gi\|57032822\|gb\|AAH88838.1\| Dnajc2 protein [Rattus norvegicus], gi\|149046589\|gb\|EDL99414.1\| DnaJ (Hsp40) homolog, subfamily C, member 2 [Rattus norvegicus] | gi\|158138509 (+3) | 72 | 5 | 1 | 3 | 3 | 2 |
| 1782 | L-iditol 2-dehydrogenase [Rattus norvegicus] | gi\|397357 (+2) | 43 | 6 | 0 | 2 | 2 | 4 |
| 1783 | PREDICTED: similar to WAS protein family, member 3 [Rattus norvegicus], gi\|149034868\|gb\|EDL89588.1\| rCG42551 [Rattus norvegicus] | gi\|109496483 | 55 | 7 | 0 | 2 | 2 | 5 |
| 1784 | signal-induced proliferation-associated 1 like 2 [Rattus norvegicus], gi\|81889098\|sp\|Q5JCS6.1\|SI1L2_RAT RecName: Full=Signal-induced proliferation-associated 1-like protein 2; Short=SIPA1-like protein 2; AltName: Full=Serine-rich synapse-associated protei | gi\|57527530 | 190 | 3 | 0 | 2 | 2 | 1 |
| 1785 | phosphoinositide-3-kinase, regulatory subunit 4 [Rattus norvegicus], gi\|149018701\|gb\|EDL77342.1\| phosphoinositide-3-kinase, regulatory subunit 4, p150 (predicted) [Rattus norvegicus] | gi\|157817604 (+1) | 149 | 2 | 0 | 2 | 2 | 0 |
| 1786 | RecName: Full=Vesicle transport through interaction with t-SNAREs homolog 1A; AltName: Full=Vesicle transport v-SNARE protein Vti1-like 2; AltName: Full=Vti1-rp2, gi\|9719422\|gb\|AAF97791.1\|AF262222_1 SNARE Vti1a-beta protein [Rattus norvegicus], gi\|1490404 | gi\|13124592 | 26 | 7 | 0 | 2 | 2 | 5 |
| 1787 | ADP-ribosylation factor guanine nucleotide-exchange factor 2 (brefeldin A-inhibited), isoform CRA_a [Rattus norvegicus] | gi\|149042855 (+1) | 200 | 5 | 0 | 2 | 2 | 3 |
| 1788 | protein phosphatase 1A, magnesium dependent, alpha isoform, isoform CRA_b [Rattus norvegicus] | gi\|149051427 (+1) | 30 | 5 | 4 | 0 | 1 | 4 |
| 1789 | aquarius (predicted), isoform CRA_b [Rattus norvegicus] | gi\|149022936 (+1) | 169 | 6 | 1 | 0 | 0 | 6 |
| 1790 | contactin 4 [Rattus norvegicus], gi\|55976164\|sp\|Q62845.1\|CNTN4_RAT RecName: Full=Contactin-4; AltName: Full=Brain-derived immunoglobulin superfamily protein 2; Short=BIG-2; Flags: Precursor, gi\|1016012\|gb\|AAC52262.1\| neural cell adhesion protein BIG-2 pre | gi\|16758746 | 113 | 7 | 0 | 1 | 1 | 6 |
| 1791 | similar to transmembrane protein induced by tumor necrosis factor alpha, isoform CRA_c [Rattus norvegicus] | gi\|149063036 (+1) | 22 | 8 | 0 | 0 | 0 | 8 |
| 1792 | tumor protein D52-like 2, isoform CRA_b [Rattus norvegicus] | gi\|149033926 (+5) | 25 | 4 | 3 | 0 | 1 | 3 |
| 1793 | adaptin ear-binding clathrin-associated protein [Rattus norvegicus], gi\|62286965\|sp\|P69682.1\|NECP1_RAT RecName: Full=Adaptin ear-binding coat-associated protein 1; AltName: Full=NECAP endocytosis-associated protein 1; Short=NECAP-1, gi\|67678331\|gb\|AAH9749 | gi\|71361625 | 30 | 8 | 0 | 0 | 0 | 8 |
| 1794 | alpha thalassemia/mental retardation syndrome X-linked (RAD54 homolog, S. cerevisiae) [Rattus norvegicus], gi\|149055561\|gb\|EDM07145.1\| alpha thalassemia/mental retardation syndrome X-linked homolog (human) [Rattus norvegicus] | gi\|157786740 | 101 | 4 | 4 | 0 | 1 | 3 |
| 1795 | nuclear assembly factor 1 homolog [Rattus norvegicus], gi\|81909058\|sp\|Q52KK4.1\|NAF1_RAT RecName: Full=H/ACA ribonucleoprotein complex non-core subunit NAF1, gi\|62871622\|gb\|AAH94302.1\| Nuclear assembly factor 1 homolog (S. cerevisiae) [Rattus norvegicus] | gi\|67078478 | 50 | 9 | 0 | 0 | 0 | 9 |
| 1796 | cyclin G associated kinase [Rattus norvegicus], gi\|2829846\|sp\|P97874.1\|GAK_RAT RecName: Full=Cyclin G-associated kinase, gi\|1902913\|dbj\|BAA18911.1\| cyclinG-associated kinase [Rattus norvegicus] | gi\|13591947 | 144 | 3 | 1 | 3 | 3 | 0 |
| 1797 | PREDICTED: similar to peptidylprolyl isomerase (cyclophilin)-like 1 [Rattus norvegicus], gi\|149043508\|gb\|EDL96959.1\| peptidylprolyl isomerase (cyclophilin)-like 1, isoform CRA_b [Rattus norvegicus] | gi\|109509766 (+1) | 16 | 5 | 1 | 0 | 0 | 5 |
| 1798 | ubiquitin fusion degradation 1-like [Rattus norvegicus], gi\|81881868\|sp\|Q9ES53.1\|UFD1_RAT RecName: Full=Ubiquitin fusion degradation protein 1 homolog; Short=UB fusion protein 1, gi\|11037254\|gb\|AAG27535.1\|AF234601_1 UFD1 [Rattus norvegicus], gi\|149019812\| | gi\|16758158 | 34 | 7 | 2 | 0 | 1 | 6 |
| 1799 | pyridoxine 5'-phosphate oxidase [Rattus norvegicus], gi\|37082100\|sp\|O88794.1\|PNPO_RAT RecName: Full=Pyridoxine-5'-phosphate oxidase; AltName: Full=Pyridoxamine-phosphate oxidase, gi\|3237304\|gb\|AAC23707.1\| pyridoxine 5'-phosphate oxidase [Rattus norvegicus | gi\|12018270 | 30 | 6 | 2 | 0 | 1 | 5 |
| 1800 | retinaldehyde binding protein 1 [Rattus norvegicus], gi\|149057252\|gb\|EDM08575.1\| retinaldehyde binding protein 1 (predicted), isoform CRA_a [Rattus norvegicus], gi\|149057253\|gb\|EDM08576.1\| retinaldehyde binding protein 1 (predicted), isoform CRA_a [Rattus | gi\|157820375 | 36 | 5 | 2 | 0 | 1 | 4 |
| 1801 | profilin 2 [Rattus norvegicus], gi\|9547318\|gb\|AAF86616.1\| profilin II [Rattus norvegicus] | gi\|13540707 (+2) | 16 | 3 | 4 | 0 | 1 | 2 |
| 1802 | ubiquitin-like 4 [Rattus norvegicus], gi\|149029858\|gb\|EDL84970.1\| ubiquitin-like 4a (predicted) [Rattus norvegicus], gi\|183985864\|gb\|AAI66513.1\| Ubiquitin-like 4 [Rattus norvegicus] | gi\|157822017 | 18 | 9 | 0 | 0 | 0 | 9 |
| 1803 | immunoglobulin binding protein 1 [Rattus norvegicus], gi\|14285494\|sp\|O08836.2\|IGBP1_RAT RecName: Full=Immunoglobulin-binding protein 1; AltName: Full=CD79a-binding protein 1; AltName: Full=Alpha4 phosphoprotein, gi\|7109702\|gb\|AAD05364.2\| alpha4 phosphopro | gi\|13928860 | 39 | 2 | 6 | 0 | 2 | 0 |
| 1804 | rCG30854 [Rattus norvegicus] | gi\|149023940 (+1) | 31 | 8 | 0 | 0 | 0 | 8 |
| 1805 | rCG46917, isoform CRA_c [Rattus norvegicus] | gi\|149064442 (+2) | 35 | 5 | 0 | 0 | 0 | 5 |
| 1806 | rCG47908 [Rattus norvegicus] | gi\|149062249 (+1) | 46 | 6 | 0 | 1 | 1 | 5 |
| 1807 | zinc finger protein 162 [Rattus norvegicus] | gi\|149062177 | 63 | 2 | 5 | 0 | 2 | 0 |
| 1808 | phosphate cytidylyltransferase 2, ethanolamine [Rattus norvegicus], gi\|30580471\|sp\|O88637.1\|PCY2_RAT RecName: Full=Ethanolamine-phosphate cytidylyltransferase; AltName: Full=Phosphorylethanolamine transferase; AltName: Full=CTP:phosphoethanolamine cytidyl | gi\|16758340 (+1) | 45 | 1 | 4 | 0 | 1 | 0 |
| 1809 | rCG20735, isoform CRA_a [Rattus norvegicus] | gi\|149025102 (+1) | 46 | 7 | 1 | 0 | 0 | 7 |
| 1810 | rCG48290 [Rattus norvegicus] | gi\|149062546 | 163 | 5 | 0 | 0 | 0 | 5 |
| 1811 | proteasome maturation protein [Rattus norvegicus], gi\|146160676\|gb\|ABQ08567.1\| voltage-gated potassium channel beta subunit 4.1 [Rattus norvegicus], gi\|149034831\|gb\|EDL89551.1\| similar to chromosome 13 open reading frame 12 (predicted) [Rattus norvegicus] | gi\|155369273 | 16 | 7 | 0 | 0 | 0 | 7 |
| 1812 | amino-terminal enhancer of split, isoform CRA_b [Rattus norvegicus] | gi\|149034403 (+1) | 16 | 8 | 0 | 0 | 0 | 8 |
| 1813 | arrestin, beta 1 [Rattus norvegicus], gi\|114213\|sp\|P29066.1\|ARRB1_RAT RecName: Full=Beta-arrestin-1; AltName: Full=Arrestin beta-1, gi\|203102\|gb\|AAA74459.1\| beta-arrestin 1 | gi\|6978537 | 47 | 3 | 4 | 0 | 1 | 2 |
| 1814 | LSM1 homolog, U6 small nuclear RNA associated [Rattus norvegicus], gi\|149057826\|gb\|EDM09069.1\| LSM1 homolog, U6 small nuclear RNA associated (S. | gi\|157820263 | 15 | 8 | 0 | 0 | 0 | 8 |

| | | | | | | | | |
|---|---|---|---|---|---|---|---|---|
| | cerevisiae) (predicted) [Rattus norvegicus] | | | | | | | |
| 1815 | apolipoprotein H (beta-2-glycoprotein I) [Rattus norvegicus], gi\|56971279\|gb\|AAH88180.1\| Apolipoprotein H (beta-2-glycoprotein I) [Rattus norvegicus], gi\|149054630\|gb\|EDM06447.1\| apolipoprotein H [Rattus norvegicus] | gi\|57528174 (+1) | 38 | 8 | 0 | 0 | 0 | 8 |
| 1816 | RAB28, member RAS oncogene family, isoform CRA_a [Rattus norvegicus], gi\|149047306\|gb\|EDL99975.1\| RAB28, member RAS oncogene family, isoform CRA_a [Rattus norvegicus] | gi\|149047303 (+4) | 22 | 2 | 6 | 0 | 2 | 0 |
| 1817 | similar to Hypothetical protein 9030012M21 (predicted) [Rattus norvegicus] | gi\|149055134 | 15 | 0 | 2 | 0 | 1 | 0 |
| 1818 | transmembrane protein 43 [Rattus norvegicus], gi\|81883820\|sp\|Q5XIP9.1\|TMM43_RAT RecName: Full=Transmembrane protein 43; AltName: Full=Protein LUMA, gi\|53733447\|gb\|AAH83626.1\| Transmembrane protein 43 [Rattus norvegicus], gi\|149036751\|gb\|EDL91369.1\| transm | gi\|56090383 | 45 | 7 | 0 | 0 | 0 | 7 |
| 1819 | PREDICTED: similar to poliovirus receptor-related 1 [Rattus norvegicus], gi\|109484587\|ref\|XP_001066246.1\| PREDICTED: similar to poliovirus receptor-related 1 [Rattus norvegicus], gi\|149041428\|gb\|EDL95269.1\| poliovirus receptor-related 1 [Rattus norvegicus | gi\|62653566 | 57 | 8 | 0 | 0 | 0 | 8 |
| 1820 | eukaryotic translation initiation factor 4B [Rattus norvegicus], gi\|55715687\|gb\|AAH85933.1\| Eukaryotic translation initiation factor 4B [Rattus norvegicus], gi\|149031954\|gb\|EDL86866.1\| eukaryotic translation initiation factor 4B [Rattus norvegicus] | gi\|56605726 | 69 | 9 | 0 | 0 | 0 | 9 |
| 1821 | NudC domain containing 2 [Rattus norvegicus], gi\|67460619\|sp\|Q5M823.1\|NUDC2_RAT RecName: Full=NudC domain-containing protein 2, gi\|56789209\|gb\|AAH88299.1\| NudC domain containing 2 [Rattus norvegicus], gi\|149052297\|gb\|EDM04114.1\| NudC domain containing 2, | gi\|57526823 | 18 | 8 | 0 | 0 | 0 | 8 |
| 1822 | secretory isoform of TSLC1 [Rattus norvegicus] | gi\|110349989 (+4) | 37 | 9 | 0 | 0 | 0 | 9 |
| 1823 | golgi phosphoprotein 3 [Mus musculus], gi\|62461580\|ref\|NP_076467.2\| golgi phosphoprotein 3 (coat-protein) [Rattus norvegicus], gi\|50400632\|sp\|Q9CRA5.1\|GOLP3_MOUSE RecName: Full=Golgi phosphoprotein 3; AltName: Full=Coat protein GPP34, gi\|12838867\|dbj\|BAB2 | gi\|14140240 (+1) | 34 | 8 | 0 | 0 | 0 | 8 |
| 1824 | THUMP domain containing 1 [Rattus norvegicus], gi\|56585045\|gb\|AAH87659.1\| THUMP domain containing 1 [Rattus norvegicus] | gi\|57527375 | 39 | 0 | 8 | 0 | 3 | 0 |
| 1825 | centrin 2, isoform CRA_a [Rattus norvegicus] | gi\|149027095 (+2) | 20 | 7 | 2 | 0 | 1 | 6 |
| 1826 | exocyst complex component 4, isoform CRA_a [Rattus norvegicus] | gi\|149065217 (+2) | 109 | 2 | 0 | 0 | 0 | 2 |
| 1827 | RecName: Full=60S ribosomal protein L13a, gi\|460776\|emb\|CAA48343.1\| rat ribosomal protein L13a [Rattus norvegicus] | gi\|548747 (+1) | 23 | 6 | 0 | 0 | 0 | 6 |
| 1828 | RNA binding motif protein 16 [Rattus norvegicus], gi\|1438530\|gb\|AAC52656.1\| rA8 | gi\|20806133 | 140 | 0 | 9 | 0 | 3 | 0 |
| 1829 | Cu/Zn superoxide dismutase [Rattus norvegicus] | gi\|1213217 (+4) | 16 | 7 | 0 | 0 | 0 | 7 |
| 1830 | rCG41948, isoform CRA_a [Rattus norvegicus] | gi\|149032877 (+1) | 28 | 5 | 2 | 0 | 1 | 4 |
| 1831 | RecName: Full=Neurexin-2-alpha; AltName: Full=Neurexin II-alpha; Flags: Precursor | gi\|124106289 (+5) | 185 | 5 | 0 | 2 | 2 | 3 |
| 1832 | WD repeat domain 48 (predicted) [Rattus norvegicus] | gi\|149018256 | 80 | 6 | 0 | 0 | 0 | 6 |
| 1833 | rCG52465, isoform CRA_a [Rattus norvegicus] | gi\|149033399 (+1) | 63 | 7 | 0 | 1 | 1 | 6 |
| 1834 | oxysterol binding protein-like 2 [Rattus norvegicus], gi\|60552731\|gb\|AAH91208.1\| Oxysterol binding protein-like 2 [Rattus norvegicus], gi\|149034041\|gb\|EDL88824.1\| oxysterol binding protein-like 2, isoform CRA_b [Rattus norvegicus] | gi\|61556891 | 55 | 5 | 2 | 0 | 1 | 4 |
| 1835 | IMP (inosine monophosphate) dehydrogenase 1 (predicted), isoform CRA_b [Rattus norvegicus] | gi\|149065132 (+1) | 55 | 4 | 0 | 1 | 1 | 3 |
| 1836 | PREDICTED: neural cell adhesion molecule L1 [Rattus norvegicus], gi\|149029901\|gb\|EDL85013.1\| rCG43874 [Rattus norvegicus] | gi\|109462307 | 126 | 4 | 3 | 0 | 1 | 3 |
| 1837 | Rtf1, Paf1/RNA polymerase II complex component, homolog [Rattus norvegicus], gi\|149023028\|gb\|EDL79922.1\| Rtf1, Paf1/RNA polymerase II complex component, homolog (S. cerevisiae) (predicted) [Rattus norvegicus] | gi\|157822517 | 49 | 1 | 6 | 0 | 2 | 0 |
| 1838 | geranylgeranyl diphosphate synthase 1 [Rattus norvegicus], gi\|81891588\|sp\|Q6F596.1\|GGPPS_RAT RecName: Full=Geranylgeranyl pyrophosphate synthetase; Short=GGPP synthetase; Short=GGPPSase; AltName: Full=Geranylgeranyl diphosphate synthase; Includes: RecName | gi\|56090562 | 35 | 3 | 3 | 0 | 1 | 2 |
| 1839 | Chain A, Crystal Structure Of The Conserved Core Of Protein Arginine Methyltransferase Prmt3 | gi\|13787051 (+1) | 36 | 5 | 0 | 2 | 2 | 3 |
| 1840 | phosphatidylinositol 4-kinase a [Rattus norvegicus], gi\|1944499\|dbj\|BAA19614.1\| 230kDa phosphatidylinositol 4-kinase [Rattus norvegicus] | gi\|25742825 | 231 | 6 | 0 | 2 | 2 | 4 |
| 1841 | DEAH (Asp-Glu-Ala-His) box polypeptide 38 [Rattus norvegicus], gi\|149038137\|gb\|EDL92497.1\| DEAH (Asp-Glu-Ala-His) box polypeptide 38 (predicted), isoform CRA_a [Rattus norvegicus] | gi\|157822135 | 139 | 5 | 2 | 1 | 2 | 3 |
| 1842 | PREDICTED: similar to Endoribonuclease Dicer (Double-strand-specific ribonuclease mDCR-1) [Rattus norvegicus], gi\|109479876\|ref\|XP_001069041.1\| PREDICTED: similar to Endoribonuclease Dicer (Double-strand-specific ribonuclease mDCR-1) [Rattus norvegicus], | gi\|109478606 | 217 | 6 | 1 | 1 | 1 | 5 |
| 1843 | LUC7-like (S. cerevisiae), isoform CRA_a [Rattus norvegicus] | gi\|149052188 (+2) | 38 | 4 | 2 | 1 | 2 | 2 |
| 1844 | similar to LIM and senescent cell antigen-like domains 1 (predicted), isoform CRA_c [Rattus norvegicus] | gi\|149038802 | 38 | 5 | 0 | 3 | 3 | 2 |
| 1845 | ephrin B3 [Rattus norvegicus], gi\|149053051\|gb\|EDM04868.1\| ephrin B3 (predicted) [Rattus norvegicus] | gi\|158303312 (+1) | 36 | 6 | 0 | 0 | 0 | 6 |
| 1846 | tumor susceptibility gene 101 [Rattus norvegicus], gi\|71153364\|sp\|Q6IRE4.1\|TS101_RAT RecName: Full=Tumor susceptibility gene 101 protein; AltName: Full=ESCRT-I complex subunit TSG101, gi\|47718036\|gb\|AAH70951.1\| Tumor susceptibility gene 101 [Rattus norveg | gi\|48374087 | 44 | 6 | 2 | 0 | 1 | 5 |
| 1847 | kinesin light chain 2 (predicted), isoform CRA_b [Rattus norvegicus], gi\|183985977\|gb\|AAI66555.1\| Klc2 protein [Rattus norvegicus] | gi\|149062042 | 69 | 5 | 0 | 0 | 0 | 5 |
| 1848 | neurexin 3 [Rattus norvegicus], gi\|17367337\|sp\|Q07310.1\|NRX3A_RAT RecName: Full=Neurexin-3-alpha; AltName: Full=Neurexin III-alpha; Flags: Precursor, gi\|294601\|gb\|AAA02857.1\| neurexin III-alpha [Rattus norvegicus] | gi\|168823431 (+7) | 174 | 7 | 0 | 0 | 0 | 7 |
| 1849 | RecName: Full=Fibronectin; Short=FN; Flags: Precursor, gi\|56164\|emb\|CAA34020.1\| precursor polypeptide (AA -32 to 2445) [Rattus norvegicus] | gi\|120178 (+5) | 273 | 4 | 2 | 0 | 1 | 3 |
| 1850 | component of oligomeric golgi complex 7 [Rattus norvegicus], gi\|123780891\|sp\|Q3T1G7.1\|COG7_RAT RecName: Full=Conserved oligomeric Golgi complex subunit 7; Short=COG complex subunit 7; AltName: Full=Component of oligomeric Golgi complex 7, gi\|74354456\|gb\|A | gi\|76559913 | 86 | 6 | 0 | 0 | 0 | 6 |
| 1851 | coiled-coil domain containing 104 [Rattus norvegicus], gi\|81908690\|sp\|Q4V8E4.1\|CC104_RAT RecName: Full=Coiled-coil domain-containing protein 104, gi\|66910609\|gb\|AAH97425.1\| Coiled-coil domain containing 104 [Rattus norvegicus], gi\|149044840\|gb\|EDL98026.1\| | gi\|67846002 | 40 | 4 | 2 | 0 | 1 | 3 |
| 1852 | dynamin 2, isoform CRA_b [Rattus norvegicus] | gi\|149020487 (+3) | 97 | 8 | 0 | 0 | 0 | 8 |
| 1853 | rCG25777, isoform CRA_a [Rattus norvegicus] | gi\|149018671 (+2) | 42 | 3 | 5 | 0 | 2 | 1 |
| 1854 | AKT1 substrate 1 (proline-rich) (predicted), isoform CRA_b [Rattus norvegicus] | gi\|149056020 (+1) | 26 | 7 | 0 | 0 | 0 | 7 |
| 1855 | inositol polyphosphate-5-phosphatase K [Rattus norvegicus], gi\|53734361\|gb\|AAH83572.1\| Inositol polyphosphate-5-phosphatase K [Rattus norvegicus], gi\|149053417\|gb\|EDM05234.1\| similar to putative phosphoinositide 5-phosphatase type II; C62, isoform CRA_b [ | gi\|62078453 | 52 | 6 | 0 | 0 | 0 | 6 |
| 1856 | vitamin K epoxide reductase complex, subunit 1-like 1 [Rattus norvegicus], gi\|46309457\|ref\|NP_081397.1\| vitamin K epoxide reductase complex, subunit 1-like 1 isoform 1 [Mus musculus], gi\|62511142\|sp\|Q6TEK3.1\|VKORL_RAT RecName: Full=Vitamin K epoxide reduc | gi\|42627869 | 20 | 7 | 0 | 0 | 0 | 7 |
| 1857 | zinc binding alcohol dehydrogenase, domain containing 1, isoform CRA_d [Rattus norvegicus] | gi\|149025118 (+2) | 36 | 4 | 2 | 0 | 1 | 3 |
| 1858 | golgi autoantigen, golgin subfamily a, 2 [Rattus norvegicus], gi\|6226622\|sp\|Q62839.2\|GOGA2_RAT RecName: Full=Golgin subfamily A member 2; AltName: Full=Cis-Golgi matrix protein GM130, gi\|4583537\|gb\|AAB53335.2\| cis-Golgi matrix protein GM130 [Rattus norveg | gi\|12018260 (+3) | 111 | 2 | 5 | 0 | 2 | 0 |
| 1859 | ras homolog gene family, member C precursor [Homo sapiens], gi\|111494248\|ref\|NP_001036143.1\| ras homolog gene family, member C precursor [Homo sapiens], gi\|111494251\|ref\|NP_001036144.1\| ras homolog gene family, member C precursor [Homo sapiens], gi\|114051 | gi\|28395033 | 22 | 7 | 0 | 0 | 0 | 7 |
| 1860 | ras homolog gene family, member E [Homo sapiens], gi\|13386354\|ref\|NP_083086.1\| Rho family GTPase 3 [Mus musculus], gi\|56090636\|ref\|NP_001007642.1\| Rho family GTPase 3 [Rattus norvegicus], gi\|149642559\|ref\|NP_001092574.1\| ras homolog gene family, member E | gi\|4885069 | 27 | 5 | 0 | 0 | 0 | 5 |
| 1861 | 4-nitrophenylphosphatase domain and non-neuronal SNAP25-like protein homolog 1 (C. elegans), isoform CRA_e [Rattus norvegicus] | gi\|149047581 (+2) | 31 | 7 | 0 | 0 | 0 | 7 |
| 1862 | golgi associated, gamma adaptin ear containing, ARF binding protein 2, isoform CRA_c [Rattus norvegicus], gi\|149068025\|gb\|EDM17577.1\| golgi associated, gamma adaptin ear containing, ARF binding protein 2, isoform CRA_c [Rattus norvegicus] | gi\|149068024 (+2) | 38 | 4 | 0 | 4 | 4 | 0 |
| 1863 | basophilic leukemia expressed protein BLES03 [Rattus norvegicus], gi\|149062065\|gb\|EDM12488.1\| rCG47431 [Rattus norvegicus] | gi\|208431797 (+1) | 31 | 3 | 4 | 0 | 1 | 2 |
| 1864 | sorting nexin 2 (predicted), isoform CRA_d [Rattus norvegicus] | gi\|149064255 | 16 | 6 | 0 | 0 | 0 | 6 |
| 1865 | similar to RIKEN cDNA 2810037C14, isoform CRA_a [Rattus norvegicus] | gi\|149046597 | 27 | 7 | 0 | 0 | 0 | 7 |
| 1866 | tropomodulin 3 [Rattus norvegicus], gi\|50926223\|gb\|AAH79290.1\| Tropomodulin 3 [Rattus norvegicus], gi\|149019151\|gb\|EDL77792.1\| rCG25684, isoform CRA_a [Rattus norvegicus] | gi\|58865562 | 39 | 7 | 0 | 0 | 0 | 7 |
| 1867 | rCG22798 [Rattus norvegicus] | gi\|149027867 | 38 | 5 | 0 | 0 | 0 | 5 |
| 1868 | Bcl2-associated athanogene 1 (predicted), isoform CRA_a [Rattus norvegicus] | gi\|149045648 | 25 | 4 | 4 | 0 | 1 | 3 |
| 1869 | Dr1 associated protein 1 (negative cofactor 2 alpha) [Rattus norvegicus], gi\|160380648\|sp\|A0JPP1.1\|NC2A_RAT RecName: Full=Dr1-associated corepressor; AltName: Full=Dr1-associated protein 1; AltName: Full=Negative co-factor 2-alpha; Short=NC2-alpha, gi\|117 | gi\|117940017 (+1) | 22 | 4 | 4 | 0 | 1 | 3 |
| 1870 | synuclein SYN2 [Rattus sp.], gi\|149037058\|gb\|EDL91619.1\| synuclein, alpha, isoform CRA_b [Rattus norvegicus] | gi\|241081 (+1) | 16 | 8 | 0 | 0 | 0 | 8 |
| 1871 | RecName: Full=Protein Shroom2; AltName: Full=Protein Apxl; AltName: Full=Liver regeneration-related protein LRRG167, gi\|33086604\|gb\|AAP92614.1\| Ab2-404 [Rattus norvegicus] | gi\|81865305 | 158 | 4 | 3 | 0 | 1 | 3 |
| 1872 | RAB, member RAS oncogene family-like 5 [Rattus norvegicus], gi\|81882802\|sp\|Q5FVJ7.1\|RABL5_RAT RecName: Full=Rab-like protein 5, gi\|58476859\|gb\|AAH89940.1\| RAB, member RAS oncogene family-like 5 [Rattus norvegicus], gi\|149062983\|gb\|EDM13306.1\| rCG21413, is | gi\|58865382 | 21 | 6 | 2 | 0 | 1 | 5 |
| 1873 | PREDICTED: similar to Sortilin-related receptor precursor (Sorting protein-related receptor containing LDLR class A repeats) (mSorLA) (SorLA-1) (Low-density lipoprotein receptor relative with 11 ligand-binding repeats) (LDLR relative with 11 ligand-bindin | gi\|109484566 | 257 | 6 | 0 | 0 | 0 | 6 |
| 1874 | PREDICTED: similar to spindle pole body component 24 homolog [Rattus norvegicus], gi\|109484372\|ref\|XP_001077474.1\| PREDICTED: similar to spindle pole body component 24 homolog [Rattus norvegicus] | gi\|62653328 | 23 | 0 | 7 | 0 | 2 | 0 |

| | | | | | | | | | |
|---|---|---|---|---|---|---|---|---|---|
| 1875 | ATP synthase, H+ transporting, mitochondrial F0 complex, subunit B1 [Rattus norvegicus], gi\|114625\|sp\|P19511.1\|AT5F1_RAT RecName: Full=ATP synthase subunit b, mitochondrial; Flags: Precursor, gi\|207089\|gb\|AAA42187.1\| F-0-ATPase subunit b precursor, gi\|396 | gi\|19705465 | 29 | 7 | 0 | 0 | 0 | 7 |
| 1876 | RecName: Full=60S ribosomal protein L26, gi\|57692\|emb\|CAA32801.1\| unnamed protein product [Rattus rattus], gi\|226411\|prf\|\|1511091A ribosomal protein L26 | gi\|132827 (+2) | 17 | 5 | 0 | 0 | 0 | 5 |
| 1877 | rCG34221, isoform CRA_a [Rattus norvegicus], gi\|149052511\|gb\|EDM04328.1\| rCG34221, isoform CRA_a [Rattus norvegicus], gi\|149052512\|gb\|EDM04329.1\| rCG34221, isoform CRA_a [Rattus norvegicus] | gi\|149052510 (+1) | 16 | 8 | 0 | 0 | 0 | 8 |
| 1878 | brain lipid binding protein [Rattus norvegicus], gi\|1706751\|sp\|P55051.2\|FABP7_RAT RecName: Full=Fatty acid-binding protein, brain; AltName: Full=B-FABP; AltName: Full=Fatty acid-binding protein 7; AltName: Full=Brain lipid-binding protein; Short=BLBP, gi\| | gi\|13540630 (+1) | 15 | 0 | 7 | 0 | 2 | 0 |
| 1879 | acyl-CoA synthetase short-chain family member 2 [Rattus norvegicus], gi\|149030886\|gb\|EDL85913.1\| acyl-CoA synthetase short-chain family member 2 (predicted) [Rattus norvegicus] | gi\|157823265 | 79 | 3 | 2 | 1 | 2 | 1 |
| 1880 | AP1 gamma subunit binding protein 1 [Rattus norvegicus], gi\|119351043\|gb\|ABL63417.1\| AP1 gamma subunit binding protein 1 [Rattus norvegicus] | gi\|119351041 (+4) | 122 | 5 | 0 | 1 | 1 | 4 |
| 1881 | PREDICTED: similar to Ia related protein isoform 2 [Rattus norvegicus] | gi\|109488127 (+2) | 127 | 5 | 0 | 0 | 0 | 5 |
| 1882 | guanine nucleotide binding protein, alpha 13 [Rattus norvegicus], gi\|45439860\|gb\|AAS64389.1\| Galpha13 [Rattus norvegicus], gi\|45439862\|gb\|AAS64390.1\| Galpha13 [Rattus norvegicus], gi\|71089931\|gb\|AAZ23820.1\| guanine nucleotide binding protein alpha 13 [Rat | gi\|61557003 | 44 | 4 | 0 | 0 | 0 | 4 |
| 1883 | aldehyde dehydrogenase family 9, subfamily A1 [Rattus norvegicus] | gi\|149058126 | 56 | 3 | 3 | 0 | 1 | 2 |
| 1884 | phosphodiesterase 4B, cAMP specific, isoform CRA_b [Rattus norvegicus] | gi\|149044592 (+5) | 82 | 4 | 0 | 3 | 3 | 1 |
| 1885 | RAB9A, member RAS oncogene family [Rattus norvegicus], gi\|12240243\|gb\|AAG49586.1\|AF325692_1 small GTP binding protein Rab9 [Rattus norvegicus] | gi\|16758200 (+1) | 23 | 5 | 0 | 0 | 0 | 5 |
| 1886 | methyl-CpG binding domain protein 3 (predicted), isoform CRA_c [Rattus norvegicus], gi\|149034546\|gb\|EDL89283.1\| methyl-CpG binding domain protein 3 (predicted), isoform CRA_c [Rattus norvegicus] | gi\|149034545 (+2) | 28 | 6 | 0 | 0 | 0 | 6 |
| 1887 | phosphomannomutase 1 [Rattus norvegicus], gi\|55778535\|gb\|AAH86346.1\| Phosphomannomutase 1 [Rattus norvegicus], gi\|149065812\|gb\|EDM15685.1\| phosphomannomutase 1, isoform CRA_c [Rattus norvegicus] | gi\|56605724 | 30 | 5 | 2 | 0 | 1 | 4 |
| 1888 | tubulin polymerization-promoting protein family member 3 [Rattus norvegicus], gi\|81883217\|sp\|Q5PPN5.1\|TPPP3_RAT RecName: Full=Tubulin polymerization-promoting protein family member 3, gi\|56270104\|gb\|AAH87585.1\| Tubulin polymerization-promoting protein fam | gi\|57526937 | 19 | 4 | 3 | 0 | 1 | 3 |
| 1889 | protein phosphatase 1, regulatory subunit 9B [Rattus norvegicus], gi\|13431725\|sp\|O35274.1\|NEB2_RAT RecName: Full=Neurabin-2; AltName: Full=Neurabin-II; AltName: Full=Spinophilin; AltName: Full=Protein phosphatase 1 regulatory subunit 9B; AltName: Full=Neu | gi\|16758226 | 90 | 4 | 3 | 0 | 1 | 3 |
| 1890 | PIH1 domain containing 1 [Rattus norvegicus], gi\|81908644\|sp\|Q4V7F5.1\|PIHD1_RAT RecName: Full=PIH1 domain-containing protein 1, gi\|66910894\|gb\|AAH97946.1\| PIH1 domain containing 1 [Rattus norvegicus] | gi\|67846006 | 32 | 5 | 0 | 0 | 0 | 5 |
| 1891 | protein phosphatase 5, catalytic subunit [Rattus norvegicus], gi\|1709745\|sp\|P53042.1\|PPP5_RAT RecName: Full=Serine/threonine-protein phosphatase 5; Short=PP5; AltName: Full=Protein phosphatase T; Short=PPT, gi\|663080\|emb\|CAA54454.1\| protein phosphatase T | gi\|13929024 (+2) | 57 | 2 | 4 | 0 | 1 | 1 |
| 1892 | rCG29232, isoform CRA_a [Rattus norvegicus] | gi\|149034494 (+1) | 36 | 3 | 0 | 3 | 3 | 0 |
| 1893 | PREDICTED: hypothetical protein [Rattus norvegicus] | gi\|109471980 (+2) | 113 | 5 | 1 | 0 | 0 | 5 |
| 1894 | acid phosphatase 1, soluble isoform A [Rattus norvegicus], gi\|46577669\|sp\|P41498.3\|PPAC_RAT RecName: Full=Low molecular weight phosphotyrosine protein phosphatase; Short=LMW-PTPase; Short=LMW-PTP; AltName: Full=Low molecular weight cytosolic acid phosphat | gi\|10863989 (+4) | 18 | 3 | 4 | 0 | 1 | 2 |
| 1895 | p34 protein [Rattus norvegicus], gi\|62286958\|sp\|Q9R0Z7.1\|P34_RAT RecName: Full=Alpha- and gamma-adaptin-binding protein p34, gi\|5802951\|gb\|AAD51852.1\|AF178669_1 p34 [Rattus norvegicus], gi\|149041934\|gb\|EDL95775.1\| p34 protein [Rattus norvegicus] | gi\|19705519 | 34 | 7 | 0 | 0 | 0 | 7 |
| 1896 | serine/threonine kinase 38 [Rattus norvegicus] | gi\|149043496 | 54 | 4 | 0 | 0 | 0 | 4 |
| 1897 | GRIP and coiled-coil domain containing 2 [Rattus norvegicus], gi\|149038803\|gb\|EDL93092.1\| LIM and senescent cell antigen-like domains 1 (predicted) [Rattus norvegicus] | gi\|157821653 | 195 | 0 | 4 | 0 | 1 | 0 |
| 1898 | Chain A, Tao2 Kinase Domain-Staurosporine Structure, gi\|114794046\|pdb\|2GCD\|B Chain B, Tao2 Kinase Domain-Staurosporine Structure | gi\|114794045 (+5) | 35 | 6 | 0 | 1 | 1 | 5 |
| 1899 | similar to RIKEN cDNA 1810043G02; DNA segment, Chr 10, Johns Hopkins University 13, expressed, isoform CRA_a [Rattus norvegicus] | gi\|149043618 (+1) | 38 | 4 | 0 | 2 | 2 | 2 |
| 1900 | capping protein (actin filament), gelsolin-like [Rattus norvegicus], gi\|81884568\|sp\|Q6AYC4.1\|CAPG_RAT RecName: Full=Macrophage-capping protein; AltName: Full=Actin regulatory protein CAP-G, gi\|50926963\|gb\|AAH79104.1\| Capping protein (actin filament), gels | gi\|61556900 | 39 | 2 | 3 | 0 | 1 | 1 |
| 1901 | rCG32280, isoform CRA_a [Rattus norvegicus] | gi\|149042791 (+2) | 29 | 5 | 0 | 1 | 1 | 4 |
| 1902 | Vascular cell adhesion molecule 1 [Rattus norvegicus] | gi\|57472 (+1) | 81 | 6 | 0 | 0 | 0 | 6 |
| 1903 | tRNA methyltransferase 1 [Rattus norvegicus], gi\|54648673\|gb\|AAH85126.1\| TRM1 tRNA methyltransferase 1 homolog (S. cerevisiae) [Rattus norvegicus] | gi\|62078475 | 48 | 5 | 2 | 0 | 1 | 4 |
| 1904 | hypothetical protein LOC304554 [Rattus norvegicus], gi\|149063670\|gb\|EDM13993.1\| similar to Seizure 6-like protein precursor (predicted) [Rattus norvegicus] | gi\|197386835 | 105 | 5 | 2 | 0 | 1 | 4 |
| 1905 | component of oligomeric golgi complex 8 [Rattus norvegicus], gi\|149038104\|gb\|EDL92464.1\| component of oligomeric golgi complex 8 (predicted), isoform CRA_b [Rattus norvegicus], gi\|149038106\|gb\|EDL92466.1\| component of oligomeric golgi complex 8 (predicted | gi\|157818521 | 71 | 6 | 1 | 0 | 0 | 6 |
| 1906 | ribulose-5-phosphate-3-epimerase [Mus musculus], gi\|34924979\|sp\|Q8VEE0.1\|RPE_MOUSE RecName: Full=Ribulose-phosphate 3-epimerase; AltName: Full=Ribulose-5-phosphate-epimerase, gi\|17512311\|gb\|AAH19126.1\| Ribulose-5-phosphate-3-epimerase [Mus musculus], gi\|2 | gi\|27532955 (+1) | 25 | 4 | 2 | 0 | 1 | 3 |
| 1907 | replication factor C (activator 1) 5 [Rattus norvegicus], gi\|149063502\|gb\|EDM13825.1\| replication factor C (activator 1) 5 (predicted) [Rattus norvegicus], gi\|197245812\|gb\|AAI68902.1\| Replication factor C (activator 1) 5 [Rattus norvegicus] | gi\|157821267 | 38 | 7 | 0 | 0 | 0 | 7 |
| 1908 | similar to CG9643-PA (predicted), isoform CRA_a [Rattus norvegicus] | gi\|149061310 | 27 | 2 | 4 | 0 | 1 | 1 |
| 1909 | DNA-damage inducible protein 2 [Rattus norvegicus], gi\|66910571\|gb\|AAH97361.1\| DNA-damage inducible protein 2 [Rattus norvegicus] | gi\|77628010 | 34 | 4 | 2 | 0 | 1 | 3 |
| 1910 | tumor protein p53 inducible protein 11 (predicted), isoform CRA_a [Rattus norvegicus] | gi\|149022691 (+1) | 22 | 7 | 0 | 0 | 0 | 7 |
| 1911 | EAP30 subunit of ELL complex [Rattus norvegicus], gi\|73919325\|sp\|Q5RK19.1\|SNF8_RAT RecName: Full=Vacuolar-sorting protein SNF8; AltName: Full=ESCRT-II complex subunit VPS22; AltName: Full=ELL-associated protein of 30 kDa, gi\|55778429\|gb\|AAH86364.1\| SNF8, | gi\|56119120 | 29 | 4 | 0 | 0 | 0 | 4 |
| 1912 | hypothetical protein LOC313551 [Rattus norvegicus], gi\|149035452\|gb\|EDL90133.1\| similar to chromosome 1 open reading frame 50 (predicted), isoform CRA_a [Rattus norvegicus] | gi\|157818445 | 22 | 4 | 2 | 0 | 1 | 3 |
| 1913 | alpha-1-microglobulin/bikunin precursor [Rattus norvegicus], gi\|2497697\|sp\|Q64240.1\|AMBP_RAT RecName: Full=Protein AMBP; Contains: RecName: Full=Alpha-1-microglobulin; Contains: RecName: Full=Inter-alpha-trypsin inhibitor light chain; Short=ITI-LC; AltNam | gi\|6978497 | 39 | 0 | 7 | 0 | 2 | 0 |
| 1914 | rCG36987 [Rattus norvegicus] | gi\|149049964 (+1) | 54 | 0 | 7 | 0 | 2 | 0 |
| 1915 | type II keratin Kb23 [Rattus norvegicus], gi\|46485106\|tpg\|DAA02222.1\| TPA_exp: type II keratin Kb23 [Rattus norvegicus] | gi\|57012376 | 62 | 5 | 1 | 0 | 0 | 5 |
| 1916 | Nucleoside 2-deoxyribosyltransferase domain containing protein RGD620382 [Rattus norvegicus], gi\|71162354\|sp\|O35820.1\|RCL_RAT RecName: Full=c-Myc-responsive protein Rcl, gi\|2228586\|gb\|AAB95314.1\| RCL [Rattus norvegicus], gi\|149069385\|gb\|EDM18826.1\| Nucleo | gi\|19424174 | 18 | 1 | 6 | 0 | 2 | 0 |
| 1917 | PREDICTED: similar to NIPSNAP-related protein isoform 2 [Rattus norvegicus] | gi\|109476499 (+2) | 28 | 5 | 0 | 0 | 0 | 5 |
| 1918 | solute carrier family 25 (mitochondrial carrier; oxoglutarate carrier), member 11, isoform CRA_b [Rattus norvegicus] | gi\|149053212 (+1) | 34 | 6 | 0 | 0 | 0 | 6 |
| 1919 | PREDICTED: similar to ribosomal protein L30 [Rattus norvegicus], gi\|109499117\|ref\|XP_001059720.1\| PREDICTED: similar to ribosomal protein L30 [Rattus norvegicus] | gi\|62659829 | 13 | 7 | 0 | 0 | 0 | 7 |
| 1920 | Tu translation elongation factor, mitochondrial (predicted), isoform CRA_b [Rattus norvegicus] | gi\|149067903 (+2) | 42 | 6 | 0 | 0 | 0 | 6 |
| 1921 | seizure related 6 homolog (mouse)-like 2 [Rattus norvegicus], gi\|149067784\|gb\|EDM17336.1\| rCG40132, isoform CRA_a [Rattus norvegicus], gi\|149067785\|gb\|EDM17337.1\| rCG40132, isoform CRA_a [Rattus norvegicus] | gi\|157820407 | 99 | 5 | 0 | 0 | 0 | 5 |
| 1922 | synaptogyrin 1, isoform CRA_b [Rattus norvegicus] | gi\|149065893 (+3) | 18 | 4 | 0 | 0 | 0 | 4 |
| 1923 | mannose-P-dolichol utilization defect 1 [Rattus norvegicus], gi\|149053064\|gb\|EDM04881.1\| mannose-P-dolichol utilization defect 1 [Rattus norvegicus] | gi\|157822325 | 27 | 5 | 0 | 0 | 0 | 5 |
| 1924 | ribosomal protein S12 [Homo sapiens], gi\|40254577\|ref\|NP_035425.2\| ribosomal protein S12 [Mus musculus], gi\|47523784\|ref\|NP_999528.1\| ribosomal protein S12 [Sus scrofa], gi\|62461611\|ref\|NP_001014409.1\| ribosomal protein S12 [Bos taurus], gi\|78126139\|ref\|N | gi\|14277700 (+1) | 15 | 7 | 0 | 0 | 0 | 7 |
| 1925 | PREDICTED: similar to ribosomal protein S24 [Rattus norvegicus], gi\|109482126\|ref\|XP_001059563.1\| PREDICTED: similar to ribosomal protein S24 [Rattus norvegicus] | gi\|109480703 (+6) | 13 | 6 | 0 | 0 | 0 | 6 |
| 1926 | putatative 28 kDa protein, isoform CRA_b [Rattus norvegicus] | gi\|149044723 | 28 | 5 | 0 | 0 | 0 | 5 |
| 1927 | neurabin 1, isoform CRA_b [Rattus norvegicus] | gi\|149064939 (+1) | 146 | 2 | 5 | 0 | 2 | 0 |
| 1928 | PREDICTED: similar to opioid binding protein/cell adhesion molecule-like [Rattus norvegicus] | gi\|109458654 (+1) | 38 | 6 | 0 | 0 | 0 | 6 |
| 1929 | acireductone dioxygenase 1, isoform CRA_c [Rattus norvegicus] | gi\|149051048 (+2) | 18 | 4 | 3 | 0 | 1 | 3 |
| 1930 | PREDICTED: similar to Zwilch [Rattus norvegicus] | gi\|109483626 | 67 | 6 | 0 | 0 | 0 | 6 |
| 1931 | PREDICTED: similar to microsomal glutathione S-transferase 3 [Rattus norvegicus], gi\|109500416\|ref\|XP_001073485.1\| PREDICTED: similar to microsomal glutathione S-transferase 3 [Rattus norvegicus] | gi\|62660299 (+2) | 17 | 6 | 0 | 0 | 0 | 6 |
| 1932 | PREDICTED: similar to High mobility group protein 2 (HMG-2) [Rattus norvegicus], gi\|109494281\|ref\|XP_001063065.1\| PREDICTED: similar to High mobility group protein 2 (HMG-2) [Rattus norvegicus] | gi\|109493208 (+2) | 26 | 0 | 4 | 0 | 1 | 0 |
| 1933 | CDP-diacylglycerol synthase (phosphatidate cytidylyltransferase) 2 [Rattus norvegicus] | gi\|149023367 (+1) | 51 | 7 | 0 | 0 | 0 | 7 |
| 1934 | presenilin-1 [Rattus norvegicus], gi\|1589007\|prf\|\|2209430A presenilin 1 | gi\|1777326 (+1) | 53 | 7 | 0 | 0 | 0 | 7 |
| 1935 | PREDICTED: similar to cullin 7 isoform 1 [Rattus norvegicus], gi\|109485721\|ref\|XP_001056520.1\| PREDICTED: similar to cullin 7 isoform 3 [Rattus norvegicus], gi\|109486679\|ref\|XP_001062759.1\| PREDICTED: similar to cullin 7 isoform 1 [Rattus norvegicus], gi\| | gi\|109485719 (+2) | 193 | 2 | 1 | 0 | 0 | 2 |

| | | | | | | | | |
|---|---|---|---|---|---|---|---|---|
| 1936 | PREDICTED: similar to opposite strand transcription unit to Stag3 [Rattus norvegicus], gi\|109497231\|ref\|XP_001077942.1\| PREDICTED: similar to opposite strand transcription unit to Stag3 [Rattus norvegicus], gi\|149063124\|gb\|EDM13447.1\| opposite strand tran | gi\|34871966 | 36 | 1 | 3 | 0 | 1 | 0 |
| 1937 | F-box protein 30 [Rattus norvegicus], gi\|61212353\|sp\|Q5XI67.1\|FBX30_RAT RecName: Full=F-box only protein 30, gi\|53734553\|gb\|AAH83823.1\| F-box protein 30 [Rattus norvegicus], gi\|149039554\|gb\|EDL93716.1\| F-box protein 30 [Rattus norvegicus] | gi\|56090568 | 82 | 2 | 3 | 1 | 2 | 0 |
| 1938 | eukaryotic translation elongation factor 1 delta (guanine nucleotide exchange protein), isoform CRA_c [Rattus norvegicus] | gi\|149066166 | 31 | 3 | 1 | 0 | 0 | 3 |
| 1939 | PREDICTED: similar to Protein MICAL-3 [Rattus norvegicus] | gi\|109472576 | 119 | 2 | 2 | 2 | 3 | 0 |
| 1940 | ubiquitin-activating enzyme 5 [Rattus norvegicus], gi\|76363460\|sp\|Q5M7A4.1\|UBA5_RAT RecName: Full=Ubiquitin-like modifier-activating enzyme 5; Short=Ubiquitin-activating enzyme 5; AltName: Full=Ubiquitin-activating enzyme E1 domain-containing protein 1; A | gi\|57527209 | 45 | 6 | 0 | 0 | 0 | 6 |
| 1941 | ATG16 autophagy related 16-like 1 (S. cerevisiae) (predicted), isoform CRA_c [Rattus norvegicus] | gi\|149016413 | 46 | 6 | 0 | 0 | 0 | 6 |
| 1942 | DPH2 homolog [Rattus norvegicus], gi\|81882362\|sp\|Q568Y2.1\|DPH2_RAT RecName: Full=Diphthamide biosynthesis protein 2; AltName: Full=DPH2 homolog, gi\|62203109\|gb\|AAH92656.1\| DPH2 homolog (S. cerevisiae) [Rattus norvegicus], gi\|149035519\|gb\|EDL90200.1\| DPH2 | gi\|62543509 | 52 | 5 | 0 | 1 | 1 | 4 |
| 1943 | protein kinase C and casein kinase substrate in neurons 1 [Rattus norvegicus], gi\|22256946\|sp\|Q9Z0W5.1\|PACN1_RAT RecName: Full=Protein kinase C and casein kinase substrate in neurons protein 1; AltName: Full=Synaptic, dynamin-associated protein I; AltName | gi\|8393896 | 50 | 6 | 0 | 0 | 0 | 6 |
| 1944 | PREDICTED: similar to ATP binding domain 1 family, member B [Rattus norvegicus], gi\|109477454\|ref\|XP_001066907.1\| PREDICTED: similar to ATP binding domain 1 family, member B [Rattus norvegicus] | gi\|34871770 | 34 | 6 | 0 | 0 | 0 | 6 |
| 1945 | PREDICTED: similar to WD repeat domain 44 protein [Rattus norvegicus], gi\|109512480\|ref\|XP_001061437.1\| PREDICTED: similar to WD repeat domain 44 protein [Rattus norvegicus] | gi\|109510388 (+1) | 102 | 2 | 3 | 0 | 1 | 1 |
| 1946 | methyltransferase like 11A [Rattus norvegicus], gi\|81170406\|sp\|Q5BJX0.3\|ME11A_RAT RecName: Full=Methyltransferase-like protein 11A, gi\|60551491\|gb\|AAH91294.1\| Methyltransferase like 11A [Rattus norvegicus], gi\|149039075\|gb\|EDL93295.1\| rCG45829, isoform CR | gi\|68163475 | 25 | 0 | 5 | 0 | 2 | 0 |
| 1947 | PREDICTED: similar to transferrin receptor [Rattus norvegicus], gi\|109494513\|ref\|XP_001072774.1\| PREDICTED: similar to transferrin receptor [Rattus norvegicus] | gi\|109493400 (+2) | 86 | 6 | 0 | 0 | 0 | 6 |
| 1948 | N-myristoyltransferase 1 [Rattus norvegicus], gi\|81914601\|sp\|Q8K1Q0.1\|NMT1_RAT RecName: Full=Glycylpeptide N-tetradecanoyltransferase 1; AltName: Full=Peptide N-myristoyltransferase 1; AltName: Full=Myristoyl-CoA:protein N-myristoyltransferase 1; Short=NM | gi\|22507314 | 57 | 3 | 3 | 0 | 1 | 2 |
| 1949 | ribosomal protein S6 kinase polypeptide 1, isoform CRA_a [Rattus norvegicus] | gi\|149024192 (+1) | 82 | 3 | 0 | 3 | 3 | 0 |
| 1950 | peptidylprolyl isomerase-like 4 [Rattus norvegicus], gi\|149039530\|gb\|EDL93692.1\| peptidylprolyl isomerase (cyclophilin)-like 4 (predicted) [Rattus norvegicus] | gi\|157823523 | 41 | 3 | 0 | 3 | 3 | 0 |
| 1951 | mCG9827 [Mus musculus], gi\|149027869\|gb\|EDL83329.1\| rCG22771, isoform CRA_a [Rattus norvegicus] | gi\|148693377 (+3) | 38 | 5 | 0 | 0 | 0 | 5 |
| 1952 | cytokine induced protein 29 kDa [Rattus norvegicus], gi\|84029305\|sp\|Q498U4.3\|HCC1_RAT RecName: Full=Nuclear protein Hcc-1, gi\|71680097\|gb\|AAI00071.1\| Cytokine induced protein 29 kDa [Rattus norvegicus], gi\|149029621\|gb\|EDL84792.1\| similar to RIKEN cDNA 11 | gi\|74271863 | 24 | 0 | 5 | 0 | 2 | 0 |
| 1953 | neuroligin 2 [Rattus norvegicus] | gi\|149053086 (+1) | 93 | 5 | 0 | 0 | 0 | 5 |
| 1954 | RecName: Full=Alpha-crystallin A chain; Contains: RecName: Full=Alpha-crystallin A chain, short form, gi\|1245160\|gb\|AAA93366.1\| alpha A (insert)-crystallin, gi\|149043576\|gb\|EDL97027.1\| crystallin, alpha A, isoform CRA_a [Rattus norvegicus] | gi\|117333 (+1) | 22 | 4 | 0 | 0 | 0 | 4 |
| 1955 | homer homolog 1 [Rattus norvegicus], gi\|38604638\|sp\|Q9Z214.2\|HOME1_RAT RecName: Full=Homer protein homolog 1; AltName: Full=PSD-Zip45; AltName: Full=VASP/Ena-related gene up-regulated during seizure and LTP, gi\|3834627\|gb\|AAC71032.1\| homer-1c [Rattus norv | gi\|13928988 (+5) | 41 | 5 | 0 | 0 | 0 | 5 |
| 1956 | mitogen activated protein kinase kinase kinase 4 [Rattus norvegicus], gi\|149027485\|gb\|EDL83075.1\| mitogen activated protein kinase kinase kinase 4 (predicted) [Rattus norvegicus] | gi\|157822757 | 157 | 2 | 0 | 0 | 0 | 2 |
| 1957 | PREDICTED: similar to tripartite motif protein 33 isoform 2 [Rattus norvegicus] | gi\|109467304 (+3) | 124 | 1 | 4 | 0 | 1 | 0 |
| 1958 | PREDICTED: similar to golgi autoantigen, golgin subfamily a, 4 [Rattus norvegicus] | gi\|109484079 (+2) | 272 | 0 | 4 | 0 | 1 | 0 |
| 1959 | nucleotide binding protein 1 [Rattus norvegicus], gi\|81889029\|sp\|Q5I0L4.1\|NUBP1_RAT RecName: Full=Nucleotide binding protein 1; Short=NBP 1, gi\|56971804\|gb\|AAH88221.1\| Nucleotide binding protein 1 [Rattus norvegicus], gi\|149042586\|gb\|EDL96223.1\| nucleotid | gi\|57528160 | 34 | 0 | 6 | 0 | 2 | 0 |
| 1960 | golgi apparatus protein 1 [Rattus norvegicus] | gi\|149038203 (+1) | 134 | 3 | 0 | 0 | 0 | 3 |
| 1961 | soc-2 (suppressor of clear) homolog [Rattus norvegicus], gi\|81910889\|sp\|Q6AYI5.1\|SHOC2_RAT RecName: Full=Leucine-rich repeat protein SHOC-2, gi\|50925627\|gb\|AAH79032.1\| Soc-2 (suppressor of clear) homolog (C. elegans) [Rattus norvegicus], gi\|149040408\|gb\|E | gi\|61557121 | 65 | 6 | 0 | 0 | 0 | 6 |
| 1962 | rCG46129, isoform CRA_b [Rattus norvegicus] | gi\|149058721 (+1) | 51 | 6 | 0 | 0 | 0 | 6 |
| 1963 | purine rich element binding protein B [Rattus norvegicus], gi\|81863556\|sp\|Q68A21.3\|PURB_RAT RecName: Full=Transcriptional activator protein Pur-beta; AltName: Full=Purine-rich element-binding protein B, gi\|51775933\|dbj\|BAD38899.1\| transcription factor Pur | gi\|62945366 | 33 | 5 | 0 | 0 | 0 | 5 |
| 1964 | unnamed protein product [Rattus norvegicus] | gi\|56200 (+1) | 61 | 5 | 0 | 0 | 0 | 5 |
| 1965 | CNDP dipeptidase 2 [Rattus norvegicus], gi\|81892734\|sp\|Q6Q0N1.1\|CNDP2_RAT RecName: Full=Cytosolic non-specific dipeptidase; AltName: Full=CNDP dipeptidase 2, gi\|45680894\|gb\|AAS75316.1\| non-specific dipeptidase [Rattus norvegicus], gi\|63100240\|gb\|AAH95904. | gi\|58219062 | 53 | 2 | 4 | 0 | 1 | 1 |
| 1966 | RecName: Full=ADP-ribosylation factor-like protein 2-binding protein; Short=ARF-like 2-binding protein; AltName: Full=Binder of ARF2 protein 1, gi\|149032442\|gb\|EDL87333.1\| ADP-ribosylation factor-like 2 binding protein, isoform CRA_c [Rattus norvegicus] | gi\|147742930 (+3) | 19 | 6 | 0 | 0 | 0 | 6 |
| 1967 | PREDICTED: similar to dual specificity phosphatase 23 [Rattus norvegicus] | gi\|34880952 | 17 | 6 | 0 | 0 | 0 | 6 |
| 1968 | vacuolar protein sorting 26 (yeast), isoform CRA_d [Rattus norvegicus] | gi\|149038687 (+2) | 33 | 5 | 0 | 0 | 0 | 5 |
| 1969 | rCG26543, isoform CRA_d [Rattus norvegicus] | gi\|149022969 (+1) | 10 | 6 | 0 | 0 | 0 | 6 |
| 1970 | rCG39045, isoform CRA_b [Rattus norvegicus] | gi\|149032329 (+1) | 14 | 5 | 0 | 0 | 0 | 5 |
| 1971 | Chain A, Casein Kinase I Delta Truncation Mutant Containing Residues 1-317 Complex With Bound Tungstate, gi\|1311055\|pdb\|1CKJ\|B Chain B, Casein Kinase I Delta Truncation Mutant Containing Residues 1-317 Complex With Bound Tungstate, gi\|1311056\|pdb\|1CKI\|A C | gi\|1311054 (+9) | 37 | 6 | 0 | 0 | 0 | 6 |
| 1972 | similar to RIKEN cDNA 3200002M19 (predicted), isoform CRA_a [Rattus norvegicus] | gi\|149068694 (+2) | 17 | 6 | 0 | 0 | 0 | 6 |
| 1973 | CCR4-NOT transcription complex, subunit 7 [Mus musculus], gi\|77735577\|ref\|NP_001029484.1\| CCR4-NOT transcription complex, subunit 7 [Bos taurus], gi\|157823359\|ref\|NP_001100783.1\| CCR4-NOT transcription complex, subunit 7 [Rattus norvegicus], gi\|3219782\|sp | gi\|6755126 | 33 | 6 | 0 | 0 | 0 | 6 |
| 1974 | PREDICTED: similar to SET domain, bifurcated 1 [Rattus norvegicus] | gi\|109467185 (+1) | 144 | 1 | 5 | 0 | 2 | 0 |
| 1975 | carbonyl reductase 3 [Rattus norvegicus], gi\|149017736\|gb\|EDL76737.1\| carbonyl reductase 3 (predicted) [Rattus norvegicus], gi\|183986573\|gb\|AAI66553.1\| Carbonyl reductase 3 [Rattus norvegicus] | gi\|157819653 | 31 | 0 | 6 | 0 | 2 | 0 |
| 1976 | fibrinogen beta chain [Rattus norvegicus], gi\|124106312\|sp\|P14480.4\|FIBB_RAT RecName: Full=Fibrinogen beta chain; AltName: Full=Liver regeneration-related protein LRRG036/LRRG043/LRRG189; Contains: RecName: Full=Fibrinopeptide B; Flags: Precursor, gi\|5697 | gi\|158186678 (+1) | 54 | 4 | 1 | 0 | 0 | 4 |
| 1977 | glucosamine-6-phosphate deaminase 2 [Rattus norvegicus], gi\|149035307\|gb\|EDL90011.1\| glucosamine-6-phosphate deaminase 2 (predicted), isoform CRA_a [Rattus norvegicus], gi\|149035308\|gb\|EDL90012.1\| glucosamine-6-phosphate deaminase 2 (predicted), isoform C | gi\|157822725 | 31 | 0 | 5 | 0 | 2 | 0 |
| 1978 | ceruloplasmin, isoform CRA_a [Rattus norvegicus] | gi\|149048530 (+2) | 121 | 3 | 0 | 0 | 0 | 3 |
| 1979 | PREDICTED: similar to leucine-rich repeats and calponin homology (CH) domain containing 2 [Rattus norvegicus], gi\|109512463\|ref\|XP_001059866.1\| PREDICTED: similar to leucine-rich repeats and calponin homology (CH) domain containing 2 [Rattus norvegicus] | gi\|109510544 | 85 | 3 | 0 | 0 | 0 | 3 |
| 1980 | serine/arginine-rich protein-specific kinase 2 [Rattus norvegicus], gi\|149046572\|gb\|EDL99397.1\| serine/arginine-rich protein specific kinase 2 (predicted), isoform CRA_b [Rattus norvegicus], gi\|171846868\|gb\|AAI61879.1\| SFRS protein kinase 2 [Rattus norveg | gi\|157819063 | 77 | 4 | 0 | 0 | 0 | 4 |
| 1981 | rCG36664 [Rattus norvegicus] | gi\|149019925 (+1) | 48 | 0 | 5 | 0 | 2 | 0 |
| 1982 | tumor suppressing subtransferable candidate 1 [Rattus norvegicus], gi\|81909943\|sp\|Q5PPK9.1\|TSSC1_RAT RecName: Full=Protein TSSC1, gi\|56388622\|gb\|AAH87633.1\| Tumor suppressing subtransferable candidate 1 [Rattus norvegicus] | gi\|58865956 | 43 | 4 | 0 | 0 | 0 | 4 |
| 1983 | SH3-domain GRB2-like endophilin B2, isoform CRA_a [Rattus norvegicus] | gi\|149039097 (+2) | 44 | 5 | 0 | 0 | 0 | 5 |
| 1984 | ribosomal protein L15 [Mus musculus], gi\|15431293\|ref\|NP_002939.2\| ribosomal protein L15 [Homo sapiens], gi\|20806169\|ref\|NP_620814.1\| ribosomal protein L15 [Rattus norvegicus], gi\|118150852\|ref\|NP_001071334.1\| ribosomal protein L15 [Bos taurus], gi\|197102 | gi\|13385036 | 24 | 3 | 0 | 0 | 0 | 3 |
| 1985 | deoxythymidylate kinase (thymidylate kinase) [Rattus norvegicus], gi\|149037469\|gb\|EDL91900.1\| deoxythymidylate kinase (predicted), isoform CRA_b [Rattus norvegicus] | gi\|157817466 | 24 | 6 | 0 | 0 | 0 | 6 |
| 1986 | neuroserpin [Rattus norvegicus] | gi\|7861758 | 46 | 5 | 0 | 0 | 0 | 5 |
| 1987 | DCN1, defective in cullin neddylation 1, domain containing 5 (S. cerevisiae), isoform CRA_c [Rattus norvegicus] | gi\|149020736 (+1) | 30 | 4 | 0 | 0 | 0 | 4 |
| 1988 | PREDICTED: similar to Phospholipid transfer protein precursor (Lipid transfer protein II) [Rattus norvegicus] | gi\|109469241 | 65 | 3 | 0 | 0 | 0 | 3 |
| 1989 | Rho, GDP dissociation inhibitor (GDI) beta [Rattus norvegicus], gi\|56789330\|gb\|AAH88209.1\| Rho, GDP dissociation inhibitor (GDI) beta [Rattus norvegicus], gi\|149049138\|gb\|EDM01592.1\| Rho, GDP dissociation inhibitor (GDI) beta, isoform CRA_a [Rattus norveg | gi\|57527565 | 23 | 0 | 6 | 0 | 2 | 0 |
| 1990 | ubiquitin-conjugating enzyme E2D 3 isoform 2 [Homo sapiens], gi\|21755161\|dbj\|BAC04632.1\| unnamed protein product [Homo sapiens], gi\|149026019\|gb\|EDL82262.1\| ubiquitin-conjugating enzyme E2D 3 (UBC4/5 homolog, yeast), isoform CRA_b [Rattus norvegicus] | gi\|33149322 (+3) | 17 | 6 | 0 | 0 | 0 | 6 |
| 1991 | myocardial ischemic preconditioning associated protein 7 [Rattus norvegicus], gi\|149063552\|gb\|EDM13875.1\| rCG21114, isoform CRA_b [Rattus norvegicus] | gi\|55228663 (+1) | 61 | 6 | 0 | 0 | 0 | 6 |
| 1992 | transmembrane protein 49 [Rattus norvegicus], gi\|81902504\|sp\|Q91ZQ0.1\|TMM49_RAT RecName: Full=Transmembrane protein 49; AltName: Full=Vacuole membrane protein 1, gi\|15705883\|gb\|AAL05859.1\|AF411216_1 vacuole membrane protein 1 [Rattus norvegicus], gi\|38197 | gi\|20301974 | 46 | 5 | 0 | 0 | 0 | 5 |
| 1993 | rCG33654, isoform CRA_b [Rattus norvegicus], gi\|149054762\|gb\|EDM06579.1\| rCG33654, isoform CRA_b [Rattus norvegicus] | gi\|149054761 (+2) | 16 | 6 | 0 | 0 | 0 | 6 |

| | | | | | | | | |
|---|---|---|---|---|---|---|---|---|
| 1994 | vitamin D binding protein prepeptide | gi\|203927 (+3) | 54 | 0 | 6 | 0 | 2 | 0 |
| 1995 | PREDICTED: similar to SET binding factor 2 [Rattus norvegicus] | gi\|109462683 (+1) | 221 | 3 | 0 | 1 | 1 | 2 |
| 1996 | 5'-3' exoribonuclease 2 (predicted), isoform CRA_a [Rattus norvegicus] | gi\|149041185 | 109 | 1 | 4 | 0 | 1 | 0 |
| 1997 | nuclear receptor coactivator 1 (predicted), isoform CRA_b [Rattus norvegicus] | gi\|149050860 (+1) | 152 | 1 | 3 | 0 | 1 | 0 |
| 1998 | PREDICTED: similar to Myotubularin-related protein 1 [Rattus norvegicus], gi\|109506891\|ref\|XP_001059230.1\| PREDICTED: similar to Myotubularin-related protein 1 [Rattus norvegicus] | gi\|109506273 | 75 | 4 | 0 | 1 | 1 | 3 |
| 1999 | inositol polyphosphate-5-phosphatase F [Rattus norvegicus], gi\|149067614\|gb\|EDM17166.1\| inositol polyphosphate-5-phosphatase F (predicted), isoform CRA_c [Rattus norvegicus] | gi\|198442866 | 127 | 2 | 1 | 0 | 0 | 2 |
| 2000 | cct-5 protein [Rattus norvegicus] | gi\|51012386 | 17 | 3 | 1 | 0 | 0 | 3 |
| 2001 | dynein cytoplasmic 2 heavy chain 1 [Rattus norvegicus], gi\|81917503\|sp\|Q9JJ79.1\|DYHC2_RAT RecName: Full=Cytoplasmic dynein 2 heavy chain 1; AltName: Full=Cytoplasmic dynein 2 heavy chain; AltName: Full=Dynein cytoplasmic heavy chain 2; AltName: Full=Dynei | gi\|12711694 | 492 | 3 | 0 | 0 | 0 | 3 |
| 2002 | PREDICTED: similar to Nonsyndromic hearing impairment protein 5 homolog [Rattus norvegicus] | gi\|109472039 (+1) | 58 | 0 | 4 | 0 | 1 | 0 |
| 2003 | post-synaptic density protein 95 [Rattus norvegicus], gi\|400891\|sp\|P31016.1\|DLG4_RAT RecName: Full=Disks large homolog 4; AltName: Full=Postsynaptic density protein 95; Short=PSD-95; AltName: Full=Synapse-associated protein 90; Short=SAP90, gi\|206455\|gb\|A | gi\|9665227 | 80 | 4 | 0 | 0 | 0 | 4 |
| 2004 | ribosomal protein S23 [Homo sapiens], gi\|13195604\|ref\|NP_077137.1\| ribosomal protein S23 [Mus musculus], gi\|17530965\|ref\|NP_511172.1\| ribosomal protein S23 [Rattus norvegicus], gi\|77736325\|ref\|NP_001029862.1\| ribosomal protein S23 [Bos taurus], gi\|1547592 | gi\|4506701 (+1) | 16 | 4 | 0 | 0 | 0 | 4 |
| 2005 | rab acceptor 1 (prenylated) [Rattus norvegicus], gi\|56404679\|sp\|O35394.1\|PRAF1_RAT RecName: Full=Prenylated Rab acceptor protein 1; AltName: Full=PRA1 family protein 1, gi\|2564205\|gb\|AAB81721.1\| prenylated rab acceptor 1 [Rattus norvegicus], gi\|55562836\|g | gi\|13929090 (+1) | 21 | 3 | 1 | 0 | 0 | 3 |
| 2006 | Rho guanine nucleotide exchange factor (GEF) 12 [Rattus norvegicus], gi\|60549645\|gb\|AAX24128.1\| leukemia-associated Rho guanine nucleotide exchange factor [Rattus norvegicus] | gi\|61557421 | 173 | 1 | 3 | 0 | 1 | 0 |
| 2007 | PREDICTED: similar to Casitas B-lineage lymphoma [Rattus norvegicus], gi\|109484591\|ref\|XP_001066453.1\| PREDICTED: similar to Casitas B-lineage lymphoma [Rattus norvegicus] | gi\|109483387 (+1) | 112 | 3 | 0 | 0 | 0 | 3 |
| 2008 | PREDICTED: similar to Protein C9orf126 homolog [Rattus norvegicus] | gi\|109468136 (+3) | 78 | 2 | 0 | 0 | 0 | 2 |
| 2009 | Smndc1 protein [Rattus norvegicus] | gi\|68533649 (+1) | 14 | 4 | 0 | 0 | 0 | 4 |
| 2010 | PREDICTED: similar to jumonji, AT rich interactive domain 1C (Rbp2 like) [Rattus norvegicus] | gi\|109511645 (+2) | 175 | 0 | 5 | 0 | 2 | 0 |
| 2011 | aldehyde dehydrogenase 1A2 [Rattus norvegicus], gi\|92087021\|sp\|Q63639.2\|AL1A2_RAT RecName: Full=Retinal dehydrogenase 2; Short=RALDH 2; Short=RalDH2; AltName: Full=Aldehyde dehydrogenase family 1 member A2; AltName: Full=Retinaldehyde-specific dehydrogena | gi\|225735575 | 57 | 0 | 5 | 0 | 2 | 0 |
| 2012 | RecName: Full=Brevican core protein; AltName: Full=Brain-enriched hyaluronan-binding protein; Short=Protein BEHAB; Contains: RecName: Full=Brevican core protein isoform 1; Contains: RecName: Full=Brevican core protein isoform 2; Flags: Precursor, gi\|50939 | gi\|2506815 (+1) | 96 | 5 | 0 | 0 | 0 | 5 |
| 2013 | RecName: Full=Echinoderm microtubule-associated protein-like 1; Short=EMAP-1 | gi\|145558904 (+3) | 90 | 5 | 0 | 0 | 0 | 5 |
| 2014 | RGM domain family, member A (predicted), isoform CRA_b [Rattus norvegicus], gi\|149057182\|gb\|EDM08505.1\| RGM domain family, member A (predicted), isoform CRA_b [Rattus norvegicus] | gi\|149057181 (+1) | 47 | 4 | 0 | 0 | 0 | 4 |
| 2015 | hypothetical protein LOC498100 [Rattus norvegicus], gi\|149019987\|gb\|EDL78135.1\| rCG36622, isoform CRA_a [Rattus norvegicus] | gi\|197333822 | 56 | 4 | 0 | 0 | 0 | 4 |
| 2016 | HEAT repeat containing 6 [Rattus norvegicus], gi\|189042487\|sp\|A1EC95.1\|HEAT6_RAT RecName: Full=HEAT repeat-containing protein 6, gi\|119351091\|gb\|ABL63441.1\| 2700008B19Rik-like protein [Rattus norvegicus], gi\|119351093\|gb\|ABL63442.1\| 2700008B19Rik-like pro | gi\|120586991 (+1) | 137 | 1 | 4 | 0 | 1 | 0 |
| 2017 | biogenesis of lysosome-related organelles complex-1, subunit 2 isoform 2 [Homo sapiens], gi\|109090245\|ref\|XP_001107659.1\| PREDICTED: similar to biogenesis of lysosome-related organelles complex-1, subunit 2 isoform 3 [Macaca mulatta], gi\|18088217\|gb\|AAH20 | gi\|47717129 (+1) | 11 | 5 | 0 | 0 | 0 | 5 |
| 2018 | BH3 interacting domain death agonist [Rattus norvegicus], gi\|88909592\|sp\|Q9JLT6.2\|BID_RAT RecName: Full=BH3-interacting domain death agonist; AltName: Full=p22 BID; Short=BID; Contains: RecName: Full=BH3-interacting domain death agonist p15; AltName: Full | gi\|12083631 | 22 | 2 | 3 | 0 | 1 | 1 |
| 2019 | similar to hypothetical protein FLJ21103, isoform CRA_b [Rattus norvegicus] | gi\|149027815 (+1) | 31 | 2 | 2 | 0 | 1 | 1 |
| 2020 | similar to hypothetical protein FLJ30656 (predicted), isoform CRA_a [Rattus norvegicus] | gi\|149054357 (+1) | 20 | 5 | 0 | 0 | 0 | 5 |
| 2021 | nucleoporin 205 [Rattus norvegicus], gi\|149065249\|gb\|EDM15325.1\| nucleoporin 205kDa (predicted) [Rattus norvegicus] | gi\|157820537 | 227 | 5 | 0 | 0 | 0 | 5 |
| 2022 | transmembrane protein 111 [Mus musculus], gi\|56605786\|ref\|NP_001008356.1\| transmembrane protein 111 [Rattus norvegicus], gi\|71153384\|sp\|Q99KI3.3\|TM111_MOUSE RecName: Full=Transmembrane protein 111, gi\|71153386\|sp\|Q5U2V8.3\|TM111_RAT RecName: Full=Transmemb | gi\|28827824 | 30 | 4 | 0 | 0 | 0 | 4 |
| 2023 | serine (or cysteine) proteinase inhibitor, clade B (ovalbumin), member 6 [Rattus norvegicus], gi\|38014574\|gb\|AAH60594.1\| Serine (or cysteine) peptidase inhibitor, clade B, member 6a [Rattus norvegicus], gi\|149045246\|gb\|EDL98332.1\| serine (or cysteine) pep | gi\|40018548 | 43 | 1 | 3 | 0 | 1 | 0 |
| 2024 | glyoxylate reductase/hydroxypyruvate reductase [Rattus norvegicus], gi\|149045798\|gb\|EDL98798.1\| rCG54768, isoform CRA_a [Rattus norvegicus], gi\|165971635\|gb\|AAI58681.1\| Grhpr protein [Rattus norvegicus] | gi\|166091519 | 36 | 0 | 5 | 0 | 2 | 0 |
| 2025 | similar to RIKEN cDNA 1810060J02 [Rattus norvegicus] | gi\|149048939 (+1) | 46 | 3 | 1 | 0 | 0 | 3 |
| 2026 | copper metabolism (Murr1) domain containing 1 [Rattus norvegicus], gi\|149044792\|gb\|EDL97978.1\| rCG23272, isoform CRA_b [Rattus norvegicus], gi\|165970730\|gb\|AAI58753.1\| Commd1 protein [Rattus norvegicus] | gi\|169234820 | 21 | 4 | 1 | 0 | 0 | 4 |
| 2027 | rCG31475, isoform CRA_a [Rattus norvegicus] | gi\|149023878 (+1) | 72 | 3 | 0 | 0 | 0 | 3 |
| 2028 | similar to hypothetical protein FLJ20546, isoform CRA_a [Rattus norvegicus] | gi\|149052342 (+1) | 25 | 5 | 0 | 0 | 0 | 5 |
| 2029 | phosphoglucomutase 3 (predicted), isoform CRA_b [Rattus norvegicus], gi\|187469167\|gb\|AAI66838.1\| Pgm3 protein [Rattus norvegicus] | gi\|149018988 (+1) | 60 | 0 | 4 | 0 | 1 | 0 |
| 2030 | RecName: Full=Sodium/potassium-transporting ATPase subunit beta-2; AltName: Full=Sodium/potassium-dependent ATPase subunit beta-2, gi\|203043\|gb\|AAA40782.1\| (Na+, K+)-ATPase-beta-2 subunit, gi\|1314365\|gb\|AAC52918.1\| Na,K-ATPase beta 2 subunit | gi\|114400 (+2) | 33 | 4 | 0 | 0 | 0 | 4 |
| 2031 | deoxyhypusine synthase [Rattus norvegicus], gi\|54035741\|sp\|Q6AY53.1\|DHYS_RAT RecName: Full=Deoxyhypusine synthase; Short=DHS, gi\|50927729\|gb\|AAH79188.1\| Deoxyhypusine synthase [Rattus norvegicus], gi\|149037798\|gb\|EDL92158.1\| deoxyhypusine synthase, isofor | gi\|51948386 | 41 | 0 | 5 | 0 | 2 | 0 |
| 2032 | ATPase, H transporting, lysosomal V1 subunit G1 [Rattus norvegicus], gi\|149059584\|gb\|EDM10522.1\| rCG55259, isoform CRA_c [Rattus norvegicus], gi\|183985826\|gb\|AAI66422.1\| ATPase, H transporting, lysosomal V1 subunit G1 [Rattus norvegicus] | gi\|157820847 | 14 | 4 | 0 | 0 | 0 | 4 |
| 2033 | dedicator of cyto-kinesis 3 [Rattus norvegicus], gi\|149018624\|gb\|EDL77265.1\| dedicator of cyto-kinesis 3 (predicted) [Rattus norvegicus] | gi\|157823849 | 153 | 5 | 0 | 0 | 0 | 5 |
| 2034 | RecName: Full=Cysteine and glycine-rich protein 2; Short=Cysteine-rich protein 2; Short=CRP2; AltName: Full=Smooth muscle cell LIM protein; Short=SmLIM, gi\|1314351\|gb\|AAC52554.1\| smooth muscle cell LIM protein, gi\|3184549\|gb\|AAC18966.1\| smooth muscle LIM | gi\|2497675 (+1) | 21 | 5 | 0 | 0 | 0 | 5 |
| 2035 | PREDICTED: similar to 60S ribosomal protein L27a [Rattus norvegicus], gi\|109461766\|ref\|XP_001079874.1\| PREDICTED: similar to 60S ribosomal protein L27a [Rattus norvegicus] | gi\|62639430 (+3) | 17 | 5 | 0 | 0 | 0 | 5 |
| 2036 | RER1 homolog [Mus musculus], gi\|84781666\|ref\|NP_001034101.1\| RER1 retention in endoplasmic reticulum 1 homolog [Rattus norvegicus], gi\|76363869\|sp\|Q9CQU3.1\|RER1_MOUSE RecName: Full=Protein RER1, gi\|118573308\|sp\|Q498C8.1\|RER1_RAT RecName: Full=Protein RER1 | gi\|13385882 | 23 | 5 | 0 | 0 | 0 | 5 |
| 2037 | DPPY splice variant c [Rattus norvegicus] | gi\|112363659 (+2) | 91 | 4 | 0 | 0 | 0 | 4 |
| 2038 | immunoglobulin light chain [Rattus sp.] | gi\|243868 (+1) | 26 | 3 | 0 | 0 | 0 | 3 |
| 2039 | PREDICTED: similar to slit homolog 1 [Rattus norvegicus] | gi\|109484799 (+1) | 81 | 4 | 0 | 0 | 0 | 4 |
| 2040 | PREDICTED: similar to periodic tryptophan protein 1 homolog [Rattus norvegicus], gi\|109481734\|ref\|XP_001075998.1\| PREDICTED: similar to periodic tryptophan protein 1 homolog [Rattus norvegicus] | gi\|34862412 | 56 | 3 | 0 | 0 | 0 | 3 |
| 2041 | RecName: Full=Glutaminase kidney isoform, mitochondrial; Short=GLS; AltName: Full=L-glutamine amidohydrolase; AltName: Full=K-glutaminase; Contains: RecName: Full=Glutaminase kidney isoform 68 kDa chain; Contains: RecName: Full=Glutaminase kidney isoform | gi\|121447 (+3) | 74 | 5 | 0 | 0 | 0 | 5 |
| 2042 | PREDICTED: similar to ribosomal protein L22 like 1 [Rattus norvegicus], gi\|109470813\|ref\|XP_001081142.1\| PREDICTED: similar to ribosomal protein L22 like 1 [Rattus norvegicus] | gi\|109468809 (+1) | 14 | 5 | 0 | 0 | 0 | 5 |
| 2043 | rCG38662, isoform CRA_a [Rattus norvegicus] | gi\|149036085 (+2) | 26 | 4 | 0 | 0 | 0 | 4 |
| 2044 | isocitrate dehydrogenase 3 (NAD), gamma, isoform CRA_a [Rattus norvegicus] | gi\|149029905 (+4) | 37 | 5 | 0 | 0 | 0 | 5 |
| 2045 | homolog of zebrafish ES1 [Rattus norvegicus] | gi\|149043607 (+1) | 25 | 5 | 0 | 0 | 0 | 5 |
| 2046 | ATP synthase, H+ transporting, mitochondrial F1 complex, delta subunit, isoform CRA_b [Rattus norvegicus] | gi\|149034587 | 18 | 3 | 0 | 0 | 0 | 3 |
| 2047 | acyl-Coenzyme A dehydrogenase family, member 9, isoform CRA_c [Rattus norvegicus] | gi\|149048722 (+2) | 69 | 5 | 0 | 0 | 0 | 5 |
| 2048 | signal recognition particle 19 [Rattus norvegicus], gi\|149017170\|gb\|EDL76221.1\| signal recognition particle 19 (predicted), isoform CRA_a [Rattus norvegicus], gi\|187469798\|gb\|AAI67043.1\| Signal recognition particle 19 [Rattus norvegicus] | gi\|157817949 | 16 | 5 | 0 | 0 | 0 | 5 |
| 2049 | beta-glo [Rattus norvegicus], gi\|34849742\|gb\|AAH58502.1\| Beta-glo [Rattus norvegicus] | gi\|40445397 (+1) | 16 | 5 | 0 | 0 | 0 | 5 |
| 2050 | PREDICTED: similar to ubiquitin protein ligase E3 component n-recognin 2 isoform 3 [Rattus norvegicus] | gi\|109486657 (+1) | 199 | 0 | 4 | 0 | 1 | 0 |
| 2051 | PREDICTED: similar to WD repeat and FYVE domain containing 3 [Rattus norvegicus] | gi\|109499467 | 396 | 3 | 0 | 1 | 1 | 2 |
| 2052 | vacuolar protein sorting 33 homolog B [Rattus norvegicus], gi\|23396894\|sp\|Q63616.1\|VP33B_RAT RecName: Full=Vacuolar protein sorting-associated protein 33B; Short=r-vps33b, gi\|1477470\|gb\|AAC52986.1\| vacuolar protein sorting homolog r-vps33b, gi\|51859108\|gb | gi\|25742586 | 71 | 3 | 0 | 1 | 1 | 2 |
| 2053 | ankyrin repeat and sterile alpha motif domain containing 1A [Rattus norvegicus], gi\|149043458\|gb\|EDL96909.1\| ankyrin repeat and SAM domain containing 1 (predicted) [Rattus norvegicus] | gi\|157820667 | 122 | 1 | 2 | 0 | 1 | 0 |

| | | | | | | | | |
|---|---|---|---|---|---|---|---|---|
| 2054 | Surfeit 2 [Rattus norvegicus] | gi\|37747910 (+1) | 29 | 3 | 1 | 0 | 0 | 3 |
| 2055 | similar to RIKEN cDNA 1190002C06, isoform CRA_a [Rattus norvegicus] | gi\|149064303 (+1) | 46 | 3 | 0 | 0 | 0 | 3 |
| 2056 | PREDICTED: similar to KARP-1 binding protein 1 [Rattus norvegicus] | gi\|109478779 (+1) | 203 | 2 | 2 | 0 | 1 | 1 |
| 2057 | NADH dehydrogenase (ubiquinone) 1 alpha subcomplex, 8 [Rattus norvegicus] | gi\|149038913 | 20 | 4 | 0 | 0 | 0 | 4 |
| 2058 | PREDICTED: hypothetical protein [Rattus norvegicus], gi\|109492384\|ref\|XP_001081787.1\| PREDICTED: hypothetical protein [Rattus norvegicus] | gi\|109489500 (+1) | 95 | 3 | 0 | 0 | 0 | 3 |
| 2059 | similar to 5730405I09Rik protein (predicted), isoform CRA_a [Rattus norvegicus] | gi\|149028381 (+3) | 24 | 4 | 0 | 0 | 0 | 4 |
| 2060 | MON1 homolog b (yeast) (predicted), isoform CRA_a [Rattus norvegicus] | gi\|149038249 (+1) | 63 | 3 | 0 | 0 | 0 | 3 |
| 2061 | RecName: Full=Lysosome-associated membrane glycoprotein 2; Short=LAMP-2; AltName: Full=Lysosomal membrane glycoprotein type B; Short=LGP-B; AltName: Full=LGP-96; AltName: Full=LGP-110; AltName: Full=CD107 antigen-like family member B; AltName: CD_antigen= | gi\|126382 (+5) | 45 | 4 | 0 | 0 | 0 | 4 |
| 2062 | signal recognition particle 54 [Rattus norvegicus] | gi\|149051248 (+1) | 30 | 3 | 0 | 0 | 0 | 3 |
| 2063 | BCL2-associated athanogene 5 [Rattus norvegicus], gi\|226509196\|ref\|NP_001140774.1\| hypothetical protein LOC100272849 [Zea mays], gi\|68051940\|sp\|Q5QJC9.1\|BAG5_RAT RecName: Full=BAG family molecular chaperone regulator 5; Short=BAG-5; AltName: Full=Bcl-2-as | gi\|56606102 | 51 | 3 | 0 | 0 | 0 | 3 |
| 2064 | PREDICTED: similar to CG13957-PA [Rattus norvegicus], gi\|109481736\|ref\|XP_001076152.1\| PREDICTED: similar to CG13957-PA [Rattus norvegicus] | gi\|62651891 | 136 | 4 | 0 | 0 | 0 | 4 |
| 2065 | PREDICTED: similar to Thiopurine S-methyltransferase (Thiopurine methyltransferase) [Rattus norvegicus], gi\|12585364\|sp\|Q9Z0T0.1\|TPMT_RAT RecName: Full=Thiopurine S-methyltransferase; AltName: Full=Thiopurine methyltransferase, gi\|4325177\|gb\|AAD17293.1\| t | gi\|109505576 (+2) | 28 | 2 | 2 | 0 | 1 | 1 |
| 2066 | RecName: Full=PDZ and LIM domain protein 1; AltName: Full=Elfin; AltName: Full=LIM domain protein CLP-36; AltName: Full=C-terminal LIM domain protein 1, gi\|47940150\|gb\|AAH72465.1\| PDZ and LIM domain 1 [Rattus norvegicus], gi\|149040142\|gb\|EDL94180.1\| PDZ a | gi\|78099788 (+1) | 36 | 2 | 0 | 0 | 0 | 2 |
| 2067 | oxidation resistance 1, isoform CRA_a [Rattus norvegicus] | gi\|149066454 (+1) | 87 | 0 | 3 | 0 | 1 | 0 |
| 2068 | NHL repeat containing 2 [Rattus norvegicus], gi\|149040462\|gb\|EDL94500.1\| NHL repeat containing 2 (predicted) [Rattus norvegicus] | gi\|157818439 | 79 | 0 | 4 | 0 | 1 | 0 |
| 2069 | F-box protein 22 [Rattus norvegicus], gi\|78394960\|gb\|AAI07671.1\| F-box protein 22 [Rattus norvegicus], gi\|149041725\|gb\|EDL95566.1\| rCG58340, isoform CRA_b [Rattus norvegicus] | gi\|83642814 | 44 | 0 | 4 | 0 | 1 | 0 |
| 2070 | DnaJ (Hsp40) homolog, subfamily B, member 11 [Rattus norvegicus], gi\|81885840\|sp\|Q6TUG0.1\|DJB11_RAT RecName: Full=DnaJ homolog subfamily B member 11; AltName: Full=ER-associated dnaJ protein 3; AltName: Full=ERj3p; AltName: Full=ERdj3; AltName: Full=ER-as | gi\|62543491 | 40 | 3 | 0 | 0 | 0 | 3 |
| 2071 | 5'-nucleotidase domain containing 3 [Rattus norvegicus], gi\|109481805\|ref\|XP_001079805.1\| PREDICTED: similar to CG1814-PA, isoform A [Rattus norvegicus] | gi\|199562203 | 63 | 3 | 0 | 0 | 0 | 3 |
| 2072 | calcium channel, voltage-dependent, beta 1 subunit, isoform CRA_c [Mus musculus], gi\|149054083\|gb\|EDM05900.1\| calcium channel, voltage-dependent, beta 1 subunit, isoform CRA_c [Rattus norvegicus] | gi\|148684164 (+4) | 28 | 2 | 0 | 0 | 0 | 2 |
| 2073 | PREDICTED: similar to Dystroglycan precursor (Dystrophin-associated glycoprotein 1) [Rattus norvegicus] | gi\|34865933 | 97 | 3 | 0 | 0 | 0 | 3 |
| 2074 | RecName: Full=Ig gamma-2A chain C region | gi\|121052 (+11) | 35 | 0 | 4 | 0 | 1 | 0 |
| 2075 | rCG38922 [Rattus norvegicus] | gi\|149031320 | 186 | 0 | 4 | 0 | 1 | 0 |
| 2076 | glucosidase, alpha, acid, isoform CRA_a [Rattus norvegicus], gi\|149054965\|gb\|EDM06782.1\| glucosidase, alpha, acid, isoform CRA_a [Rattus norvegicus] | gi\|149054964 | 106 | 3 | 0 | 0 | 0 | 3 |
| 2077 | histone H2B [Bos taurus], gi\|114605792\|ref\|XP_001172848.1\| PREDICTED: similar to histone H2B isoform 1 [Pan troglodytes], gi\|67968669\|dbj\|BAE00693.1\| unnamed protein product [Macaca fascicularis], gi\|149029303\|gb\|EDL84570.1\| rCG63091 [Rattus norvegicus], | gi\|157785601 | 7 | 0 | 4 | 0 | 1 | 0 |
| 2078 | potassium channel tetramerisation domain containing 5 [Rattus norvegicus], gi\|149051987\|gb\|EDM03804.1\| potassium channel tetramerisation domain containing 5 (predicted) [Rattus norvegicus], gi\|197246769\|gb\|AAI68712.1\| Potassium channel tetramerisation dom | gi\|157786778 | 26 | 4 | 0 | 0 | 0 | 4 |
| 2079 | PREDICTED: similar to hematopoietic stem/progenitor cells 176 [Rattus norvegicus], gi\|109513532\|ref\|XP_001075285.1\| PREDICTED: similar to hematopoietic stem/progenitor cells 176 [Rattus norvegicus] | gi\|109507775 (+2) | 18 | 4 | 0 | 0 | 0 | 4 |
| 2080 | NADH dehydrogenase (ubiquinone) 1 beta subcomplex, 6 [Rattus norvegicus], gi\|149045633\|gb\|EDL98633.1\| NADH dehydrogenase (ubiquinone) 1 beta subcomplex, 6 (predicted) [Rattus norvegicus] | gi\|157820465 | 16 | 4 | 0 | 0 | 0 | 4 |
| 2081 | Chain A, 1.7 Angstrom Crystal Structure Of The Rat Vap-A Msp Homology Domain | gi\|73535844 | 14 | 4 | 0 | 0 | 0 | 4 |
| 2082 | RecName: Full=Sigma 1-type opioid receptor; Short=Sigma1-receptor; Short=Sigma1R, gi\|6434855\|gb\|AAF08342.1\|AF067769_1 sigma1 receptor [Rattus norvegicus], gi\|38541101\|gb\|AAH61978.1\| Opioid receptor, sigma 1 [Rattus norvegicus], gi\|149045689\|gb\|EDL98689.1\| | gi\|81882051 | 25 | 3 | 0 | 0 | 0 | 3 |
| 2083 | mitochondrial trifunctional protein, alpha subunit [Rattus norvegicus], gi\|172045972\|sp\|Q64428.2\|ECHA_RAT RecName: Full=Trifunctional enzyme subunit alpha, mitochondrial; AltName: Full=TP-alpha; Includes: RecName: Full=Long-chain enoyl-CoA hydratase; Incl | gi\|148747393 (+1) | 83 | 4 | 0 | 0 | 0 | 4 |
| 2084 | cystatin C, isoform CRA_a [Rattus norvegicus] | gi\|149041145 (+4) | 17 | 0 | 4 | 0 | 1 | 0 |
| 2085 | similar to adipocyte-specific protein 4 [Rattus norvegicus] | gi\|149054551 (+1) | 56 | 3 | 0 | 0 | 0 | 3 |
| 2086 | RN protein [Rattus norvegicus], gi\|119371017\|sp\|Q2TA68.1\|OPA1_RAT RecName: Full=Dynamin-like 120 kDa protein, mitochondrial; AltName: Full=Optic atrophy protein 1 homolog; Contains: RecName: Full=Dynamin-like 120 kDa protein, form S1; Flags: Precursor, gi | gi\|148747459 (+2) | 111 | 4 | 0 | 0 | 0 | 4 |
| 2087 | PREDICTED: similar to 60S ribosomal protein L32 [Rattus norvegicus], gi\|109476925\|ref\|XP_001064496.1\| PREDICTED: similar to 60S ribosomal protein L32 [Rattus norvegicus] | gi\|109475208 (+1) | 15 | 3 | 0 | 0 | 0 | 3 |
| 2088 | similar to chromosome 9 open reading frame 5 (predicted) [Rattus norvegicus] | gi\|149037154 (+1) | 88 | 4 | 0 | 0 | 0 | 4 |
| 2089 | similar to oxoglutarate dehydrogenase (lipoamide), isoform CRA_d [Rattus norvegicus] | gi\|149047673 (+1) | 118 | 4 | 0 | 0 | 0 | 4 |
| 2090 | myosin light chain, regulatory B-like [Mus musculus], gi\|203097140\|ref\|NP_001128489.1\| similar to Myosin regulatory light chain 2-A, smooth muscle isoform (Myosin RLC-A) [Rattus norvegicus], gi\|127170\|sp\|P13832.2\|MRLCA_RAT RecName: Full=Myosin regulatory | gi\|71037403 | 20 | 4 | 0 | 0 | 0 | 4 |
| 2091 | rCG33672, isoform CRA_c [Rattus norvegicus] | gi\|149052162 (+1) | 21 | 0 | 4 | 0 | 1 | 0 |
| 2092 | Enah/Vasp-like [Rattus norvegicus], gi\|25090259\|sp\|O08719.1\|EVL_RAT RecName: Full=Ena/VASP-like protein; AltName: Full=Ena/vasodilator-stimulated phosphoprotein-like, gi\|2058462\|gb\|AAC53322.1\| RNB6 [Rattus norvegicus] | gi\|13162335 (+2) | 42 | 2 | 1 | 0 | 0 | 2 |
| 2093 | adapter protein RUK-m1 [Rattus norvegicus], gi\|149042433\|gb\|EDL96140.1\| SH3-domain kinase binding protein 1, isoform CRA_f [Rattus norvegicus] | gi\|14009486 (+9) | 44 | 3 | 0 | 0 | 0 | 3 |
| 2094 | serine/threonine kinase 32C [Rattus norvegicus], gi\|149061417\|gb\|EDM11840.1\| serine/threonine kinase 32C (predicted) [Rattus norvegicus] | gi\|157821485 | 55 | 2 | 1 | 0 | 0 | 2 |
| 2095 | protein-tyrosine-phosphatase (EC 3.1.3.48), receptor-linked form P1 precursor - rat | gi\|1083771 (+5) | 168 | 2 | 0 | 0 | 0 | 2 |
| 2096 | PREDICTED: similar to transformation related protein 53 binding protein 1 [Rattus norvegicus] | gi\|109470711 | 177 | 0 | 2 | 0 | 1 | 0 |
| 2097 | coagulation factor XIII, A1 subunit [Rattus norvegicus], gi\|149045192\|gb\|EDL98278.1\| coagulation factor XIII, A1 subunit, isoform CRA_a [Rattus norvegicus], gi\|149045193\|gb\|EDL98279.1\| coagulation factor XIII, A1 subunit, isoform CRA_a [Rattus norvegicus] | gi\|158186711 (+1) | 83 | 3 | 0 | 0 | 0 | 3 |
| 2098 | cyclic nucleotide phosphodiesterase 1, isoform CRA_b [Rattus norvegicus], gi\|149054232\|gb\|EDM06049.1\| cyclic nucleotide phosphodiesterase 1, isoform CRA_b [Rattus norvegicus] | gi\|149054231 (+2) | 45 | 3 | 0 | 0 | 0 | 3 |
| 2099 | PREDICTED: hypothetical protein [Rattus norvegicus], gi\|109508796\|ref\|XP_001075785.1\| PREDICTED: hypothetical protein [Rattus norvegicus] | gi\|109508061 (+2) | 144 | 2 | 0 | 0 | 0 | 2 |
| 2100 | sortilin 1, isoform CRA_a [Rattus norvegicus] | gi\|149025687 (+3) | 73 | 2 | 0 | 0 | 0 | 2 |
| 2101 | calcium/calmodulin-dependent protein kinase I [Rattus norvegicus], gi\|3122310\|sp\|Q63450.2\|KCC1A_RAT RecName: Full=Calcium/calmodulin-dependent protein kinase type 1; AltName: Full=CaM kinase I; Short=CaM-KI; AltName: Full=CaM kinase I alpha; Short=CaMKI-a | gi\|19745200 (+1) | 42 | 3 | 0 | 0 | 0 | 3 |
| 2102 | poly(A) polymerase alpha [Rattus norvegicus], gi\|149044213\|gb\|EDL97595.1\| poly (A) polymerase alpha (predicted), isoform CRA_a [Rattus norvegicus] | gi\|157820829 (+1) | 82 | 0 | 3 | 0 | 1 | 0 |
| 2103 | small nuclear ribonucleoprotein D2 isoform 1 [Homo sapiens], gi\|58037145\|ref\|NP_081219.1\| small nuclear ribonucleoprotein D2 [Mus musculus], gi\|77735903\|ref\|NP_001029648.1\| small nuclear ribonucleoprotein D2 [Bos taurus], gi\|157820925\|ref\|NP_001102869.1\| | gi\|4759158 | 14 | 3 | 0 | 0 | 0 | 3 |
| 2104 | transmembrane protein 85 [Mus musculus], gi\|157817408\|ref\|NP_001099965.1\| transmembrane protein 85 [Rattus norvegicus], gi\|81904578\|sp\|Q9CZX9.1\|TMM85_MOUSE RecName: Full=Transmembrane protein 85, gi\|12848544\|dbj\|BAB27992.1\| unnamed protein product [Mus mu | gi\|13386014 | 20 | 3 | 0 | 0 | 0 | 3 |
| 2105 | fibulin 1 [Rattus norvegicus], gi\|149065711\|gb\|EDM15584.1\| fibulin 1 (predicted) [Rattus norvegicus] | gi\|189011620 | 78 | 0 | 3 | 0 | 1 | 0 |
| 2106 | neuron derived neurotrophic factor, isoform CRA_a [Rattus norvegicus] | gi\|149041043 (+1) | 16 | 3 | 0 | 0 | 0 | 3 |
| 2107 | mesoderm development candidate 2 [Rattus norvegicus], gi\|81883487\|sp\|Q5U2R7.1\|MESD2_RAT RecName: Full=Mesoderm development candidate 2, gi\|55250764\|gb\|AAH85892.1\| Mesoderm development candidate 2 [Rattus norvegicus], gi\|149057436\|gb\|EDM08759.1\| mesoderm d | gi\|56605768 | 25 | 2 | 0 | 0 | 0 | 2 |
| 2108 | rCG42593, isoform CRA_b [Rattus norvegicus] | gi\|149034849 | 15 | 3 | 0 | 0 | 0 | 3 |
| 2109 | rCG22435 [Rattus norvegicus] | gi\|149046424 | 17 | 3 | 0 | 0 | 0 | 3 |
| 2110 | hypothetical protein LOC287929 [Rattus norvegicus], gi\|149019693\|gb\|EDL77841.1\| similar to RIKEN cDNA 2610001E06 (predicted), isoform CRA_a [Rattus norvegicus], gi\|149019694\|gb\|EDL77842.1\| similar to RIKEN cDNA 2610001E06 (predicted), isoform CRA_a [Rattu | gi\|157786734 | 16 | 3 | 0 | 0 | 0 | 3 |
| 2111 | retinoblastoma binding protein 5 [Rattus norvegicus], gi\|149058638\|gb\|EDM09795.1\| retinoblastoma binding protein 5 (predicted) [Rattus norvegicus] | gi\|157820961 | 59 | 3 | 0 | 0 | 0 | 3 |
| 2112 | zinc finger, FYVE domain containing 1 [Rattus norvegicus], gi\|149025078\|gb\|EDL81445.1\| zinc finger, FYVE domain containing 1 (predicted), isoform CRA_a [Rattus norvegicus] | gi\|157822531 | 87 | 3 | 0 | 0 | 0 | 3 |
| 2113 | coiled-coil domain containing 124 [Rattus norvegicus], gi\|149036079\|gb\|EDL90745.1\| similar to hypothetical protein BC013949 (predicted), isoform CRA_a [Rattus norvegicus], gi\|149036080\|gb\|EDL90746.1\| similar to hypothetical protein BC013949 (predicted), i | gi\|157823579 | 25 | 2 | 0 | 0 | 0 | 2 |
| 2114 | tropomyosin alpha isoform [Rattus norvegicus] | gi\|14134101 (+6) | 29 | 0 | 3 | 0 | 1 | 0 |
| 2115 | RecName: Full=Cold shock domain-containing protein C2; AltName: Full=RNA-binding protein PIPPin, gi\|149065816\|gb\|EDM15689.1\| cold shock domain containing C2, RNA binding [Rattus norvegicus] | gi\|32129826 | 17 | 0 | 2 | 0 | 1 | 0 |

<table>
<tr><td>2116</td><td>DnaJ (Hsp40) homolog, subfamily C, member 8, isoform CRA_b [Rattus norvegicus]</td><td>gi|149024145 (+1)</td><td>15</td><td>0</td><td>3</td><td>0</td><td>1</td><td>0</td></tr>
<tr><td>2117</td><td>superoxide dismutase 2 [Rattus norvegicus], gi|134678|sp|P07895.2|SODM_RAT RecName: Full=Superoxide dismutase [Mn], mitochondrial; Flags: Precursor, gi|57273|emb|CAA39937.1| manganese containing superoxide dismutase [Rattus norvegicus], gi|47477896|gb|AAH</td><td>gi|8394331</td><td>25</td><td>3</td><td>0</td><td>0</td><td>0</td><td>3</td></tr>
<tr><td>2118</td><td>PREDICTED: similar to coronin, actin binding protein, 2B [Rattus norvegicus], gi|109484821|ref|XP_001074062.1| PREDICTED: similar to coronin, actin binding protein, 2B [Rattus norvegicus], gi|149041915|gb|EDL95756.1| similar to KIAA0925 protein, isoform C</td><td>gi|109483534</td><td>55</td><td>2</td><td>0</td><td>0</td><td>0</td><td>2</td></tr>
<tr><td>2119</td><td>rCG20801, isoform CRA_a [Rattus norvegicus]</td><td>gi|149025266 (+5)</td><td>19</td><td>2</td><td>0</td><td>0</td><td>0</td><td>2</td></tr>
<tr><td>2120</td><td>sorting nexin 5 (predicted), isoform CRA_b [Rattus norvegicus]</td><td>gi|149041242</td><td>30</td><td>3</td><td>0</td><td>0</td><td>0</td><td>3</td></tr>
<tr><td>2121</td><td>adaptor-related protein complex 3, mu 2 subunit [Rattus norvegicus], gi|1703029|sp|P53678.1|AP3M2_RAT RecName: Full=AP-3 complex subunit mu-2; AltName: Full=Adapter-related protein complex 3 mu-2 subunit; AltName: Full=Mu3B-adaptin; AltName: Full=Golgi ad</td><td>gi|18959246</td><td>47</td><td>2</td><td>0</td><td>0</td><td>0</td><td>2</td></tr>
<tr><td>2122</td><td>antagonist of mitotic exit network 1 homolog [Rattus norvegicus], gi|158563899|sp|Q5U201.2|AMN1_RAT RecName: Full=Protein AMN1 homolog, gi|149048911|gb|EDM01365.1| similar to F-box protein FBL2, isoform CRA_a [Rattus norvegicus]</td><td>gi|164663789</td><td>28</td><td>0</td><td>2</td><td>0</td><td>1</td><td>0</td></tr>
<tr><td>2123</td><td>thyroid hormone receptor associated protein 6 (predicted), isoform CRA_a [Rattus norvegicus]</td><td>gi|149066398 (+1)</td><td>20</td><td>0</td><td>2</td><td>0</td><td>1</td><td>0</td></tr>
<tr><td>2124</td><td>procollagen, type VI, alpha 3 (predicted), isoform CRA_b [Rattus norvegicus]</td><td>gi|149037630 (+4)</td><td>311</td><td>0</td><td>2</td><td>0</td><td>1</td><td>0</td></tr>
<tr><td>2125</td><td>leucine rich repeat neuronal 1 [Rattus norvegicus], gi|85701140|sp|Q32Q07.1|LRRN1_RAT RecName: Full=Leucine-rich repeat neuronal protein 1; AltName: Full=Neuronal leucine-rich repeat protein 1; Short=NLRR-1; Flags: Precursor, gi|79152324|gb|AAI07903.1| Le</td><td>gi|82654226</td><td>81</td><td>2</td><td>0</td><td>0</td><td>0</td><td>2</td></tr>
<tr><td>2126</td><td>polyglutamine binding protein 1, isoform CRA_a [Rattus norvegicus]</td><td>gi|149028428 (+1)</td><td>31</td><td>2</td><td>0</td><td>0</td><td>0</td><td>2</td></tr>
<tr><td>2127</td><td>immunoglobulin superfamily, member 8, isoform CRA_a [Rattus norvegicus], gi|149040745|gb|EDL94702.1| immunoglobulin superfamily, member 8, isoform CRA_a [Rattus norvegicus], gi|149040747|gb|EDL94704.1| immunoglobulin superfamily, member 8, isoform CRA_a [</td><td>gi|149040743 (+1)</td><td>64</td><td>2</td><td>0</td><td>0</td><td>0</td><td>2</td></tr>
<tr><td>2128</td><td>glutamine synthetase 1 [Rattus norvegicus], gi|121376|sp|P09606.3|GLNA_RAT RecName: Full=Glutamine synthetase; Short=GS; AltName: Full=Glutamate--ammonia ligase, gi|57577|emb|CAA30754.1| unnamed protein product [Rattus norvegicus], gi|204402|gb|AAA65095.1</td><td>gi|142349612 (+3)</td><td>42</td><td>2</td><td>0</td><td>0</td><td>0</td><td>2</td></tr>
<tr><td>2129</td><td>plastin 3 (T-isoform) [Rattus norvegicus], gi|149030088|gb|EDL85165.1| plastin 3 (T-isoform), isoform CRA_a [Rattus norvegicus], gi|149030089|gb|EDL85166.1| plastin 3 (T-isoform), isoform CRA_a [Rattus norvegicus]</td><td>gi|209954804 (+2)</td><td>71</td><td>0</td><td>2</td><td>0</td><td>1</td><td>0</td></tr>
<tr><td>2130</td><td>myosin 9b [Rattus norvegicus]</td><td>gi|65305715 (+2)</td><td>229</td><td>0</td><td>2</td><td>0</td><td>1</td><td>0</td></tr>
<tr><td>END OF FILE</td><td></td><td></td><td></td><td></td><td></td><td></td><td></td><td></td></tr>
<tr><td></td><td></td><td></td><td></td><td></td><td></td><td></td><td></td><td></td></tr>
<tr><td></td><td></td><td></td><td></td><td></td><td></td><td></td><td></td><td></td></tr>
<tr><td></td><td></td><td></td><td></td><td></td><td></td><td></td><td></td><td></td></tr>
</table>
